# Identifying the causal relationship between immune factors and osteonecrosis: a two-sample Mendelian randomization study

**DOI:** 10.1038/s41598-024-59810-0

**Published:** 2024-04-23

**Authors:** Chao Wang, Yong Zhu, Ding Pan

**Affiliations:** Department of Orthopaedics, Xiangya Hospital, Central South University, Changsha, 410008 Hunan China

**Keywords:** Immune factors, Osteonecrosis, Drug induced osteonecrosis, Osteoimmunology, Mendelian randomization, Causal relationship, Genetics, Immunology, Risk factors

## Abstract

A wealth of evidence intimates a profound connection between the immune system and osteonecrosis, albeit the specific immune factors underlying this connection remain largely veiled. A bidirectional Mendelian randomization (MR) study was conducted based on genome-wide association study summary data to identify causal links between 731 immune factors and osteonecrosis including drug-induced osteonecrosis. Preliminary MR analysis was accomplished utilizing the inverse-variance weighted method under a multiplicative random effects model, and heterogeneity and potential horizontal pleiotropy were evaluated through Cochrane's Q-test, MR-Egger intercept test, MR-PRESSO global test, and leave-one-out analysis. Upon false discovery rate correction, the gene-predicted level of one immune factor (CD62L − monocyte %monocyte) exhibited a significant positive correlation with osteonecrosis, while eight immune traits associated with monocytes, dendritic cells, and NK cells demonstrated significant causal effects with drug-induced osteonecrosis. Reverse MR revealed no significant correlations. This MR research provides genetic evidence for the causal associations between a broad spectrum of immune factors and osteonecrosis. Such a study aids in unraveling the intricate interaction patterns between the immune and skeletal systems, elucidating the pathogenesis of osteonecrosis, and identifying potential novel therapeutic approaches.

## Introduction

Osteonecrosis, also designated as ischemic necrosis, aseptic necrosis, and avascular necrosis, is most commonly manifested as osteonecrosis of the femoral head (ONFH)^1^. Other bones like the humeral head, knee, small bones of foot and hand could also be afflicted^2–4^. While trauma often induces osteonecrosis through direct injury to bone blood supply, the prevalence of various non-traumatic osteonecrosis including steroid, alcohol and idiopathic osteonecrosis is also on the rise. According to epidemiological surveys, more than 20,000 new patients suffer from total hip arthroplasties (THAs) annually in the U.S. due to the disease, which constitutes 5–12% of all THAs^5,6^. By 2013, the cumulative number of ONFH patients in China reached 8.12 million^7^. The ultimate change in osteonecrosis is the death of the affected bone owing to the cessation of circulation, leading to the deprivation of nutrients. However, a series of complex pathophysiological changes may appear in the early stages of the disease, including ischemia, apoptosis, dysfunction of adipocytes, and coagulation changes^8,9^. Presently, the pathogenic mechanism of non-traumatic osteonecrosis remains perplexing and calls for further research and careful investigation.

The immune system and skeletal system have traditionally been considered as intimately linked. On one hand, the bone marrow serves as the primary generation and development site for immune cells, and skeletal cells can influence the formation and function of immune cells via the secretion of factors. On the other, the immune system plays a key role in skeletal health. For instance, immune cells such as T cells and B cells can produce factors affecting the function of skeletal cells like osteoblasts and osteoclasts, thereby impacting bone formation and remodeling^10,11^. Various bone diseases, including rheumatoid arthritis and osteoporosis, have been proven to be associated with immune factors^12,13^. During the onset of osteonecrosis, there are often accompanying damages to endothelial cells, bone marrow cell autophagy, apoptosis and necrosis, and fibrous repair in necrotic areas^1,14,15^. Similarly, osteonecrosis also occurs during the progression of certain autoimmune or inflammatory diseases^16–18^. All these suggest a close link between the immune system and osteonecrosis, but the specific immune factors underlying the link remain largely unknown.

In steroid users and alcoholics, only a fraction ultimately develops osteonecrosis, implying that genetic factors play an essential role in the onset of osteonecrosis^19,20^. Mendelian randomization (MR) is a method that uses single nucleotide polymorphisms (SNPs) as instrumental variables (IVs) to infer the potential causal relationship of exposure to outcomes. Since genetic variations are randomly allocated during meiosis and fertilization and are determined before the onset of disease, this mitigates the common confounding factors and risk of reverse causation in traditional observational studies^21^. In this study, we aimed to strengthen the early identification of osteonecrosis by implementing a forward MR of immune factors and osteonecrosis (ON, including osteonecrosis and drug-induced osteonecrosis) to identify immune factors potentially causally related to osteonecrosis. Reverse MR was also utilized to explore the causal relationship between osteonecrosis and immune factors, potentially assisting in prognosticating the severity of osteonecrosis.

## Materials and methods

### Study design

For the causality estimation of an MR to be valid, three assumptions^22^ must hold: (1) the genetic variant is strongly associated with the exposure, (2) the genetic variant is independent of any potential confounder of the exposure-outcome relationship, and (3) the variant does not affect the outcome independently of the exposure. We first conducted a two-sample bi-directional MR to evaluate the possible connection between immune factors and the risk of osteonecrosis as well as drug-induced osteonecrosis. Ethical approval was obtained for all original studies.

### Data Sources

We obtained the genome-wide association study (GWAS) summary statistics data related to the peripheral blood immune phenotypes from the public GWAS catalog^23^. Based on 3757 European individual samples, this GWAS estimated SNPs using the reference panel of Sardinian sequences, adjusted for covariates (sex, age, and age^2^) ^24^, and accommodated a total of 731 immune phenotypes (GWAS ID GCST90001391 to GCST90002121). These phenotypes encompass four major categories: absolute cell numbers (AC) (n = 118), median fluorescence intensity (MFI) reflecting the level of surface antigen (n = 389), relative cell numbers (RC) (n = 192), and morphological parameters (MP) (n = 32), and include seven groups of immune cells based on their type: B cells, cDCs, mature T cells, monocytes, myeloid cells, TBNK cells, and Treg cells. Summary statistics data for osteonecrosis and drug induced osteonecrosis were acquired from the FinnGen consortium R9 release data^25^. Osteonecrosis cases were defined by ICD 10 code M87 and ICD-9 code 7334, with GWAS data comprising 359,399 Finnish participants, consisting of 1385 cases and 358,014 controls. Drug induced osteonecrosis cases were defined by ICD 10 code M87.1, and the data from the ninth release of the FinnGen consortium were used for 264 cases and 377,013 non-cases, with adjustments made for gender, age, and genotyping batches during the analysis process.

### Genetic instruments selection

We followed the procedure below to select genetic variants as IVs: (1) Adopting a threshold for genome-wide significance (*P* ≤ 1 × 10^–5^), (2) SNPs with minor allele frequencies (MAFs) under 0.05 were excluded, (3) SNPs with mismatched alleles (C/T and C/A) were excluded, (4) Palindromic variants with ambiguous strands (C/G or A/T) were excluded, (5) With the 1000 genome project's European sample data serving as a reference panel in Plink, lead SNPs with parameters (window size = 500kb, r^2^ < 0.1) were pruned from the selected genetic variants above. For the characteristic of osteonecrosis, we adjusted parameters to r^2^ < 0.001 (clumping window size = 10,000 kb). The strength of IVs was evaluated by gauging the F-statistic, calculated using the formula F = R^2^ × (N-1-K)/(1-R^2^) × K, which correlates with the proportion of the exposure's variance explained (PVE) by the genetic variance (R^2^), the sample size (N), and the number of instruments (K). From our analysis, we expelled IVs with low F-statistics (< 10) thus averting significant weak instrument bias^26^.

### Statistical analysis

A spectrum of methodologies was employed in this study to infer bi-directional causality. Primary MR analysis was conducted under a multiplicative random-effects model using the IVW method, which aggregates each SNP's Wald ratio estimates into one causality estimate for each risk factor; this is considered to offer the most accurate estimate provided all IVs are valid. We resorted to a further four MR analyses, encompassing MR-Egger regression, simple mode, weighted median, and weighted mode, to conduct sensitivity testing. The Cochran Q test was employed to examine the heterogeneity of IVW estimates with *P* < 0.05 indicating significant heterogeneity. We conducted the MR-Egger interception test to detect potential directional pleiotropy, where the intercept *P* < 0.05. We also executed the MR-PRESSO method, inclusive of global and outlier tests to identify and rectify pleiotropy and potential outliers, offering causality estimates after the respective outlier's exclusion. The “leave-one-out” method and the funnel and scatter plots of the MR analysis were employed for an intuitive evaluation of directional pleiotropy and heterogeneity (Supplemental materials). Moreover, reverse MR was executed to deduce the causal link between osteonecrosis and immune factors.

We defined evidence of a significant causal effect when the following criteria were met: the MR results of IVW passed multiple comparisons with *q*-value < 0.2 after false discovery rate (FDR) correction; the remaining MR methods’ result showed a similar size and direction to IVW; there was no evidence of heterogeneity and directional pleiotropy after potential outliers were excluded; *p*-value was two-sided. MR and other analyses were conducted using the “TwoSampleMR” R package in R (version 4.3.1). Simultaneously, when the MR *p*-value of IVW was < 0.05, the remaining MR methods showed the same direction as IVW, and there was no heterogeneity and directional pleiotropy, the results were considered to potentially imply causality.

### Ethical approval and consent to participate

All analyses were based on publicly shared databases and no additional ethical approvals were required.

## Results

### Overview of the effect of immune factors on ON

**Figure 1:**
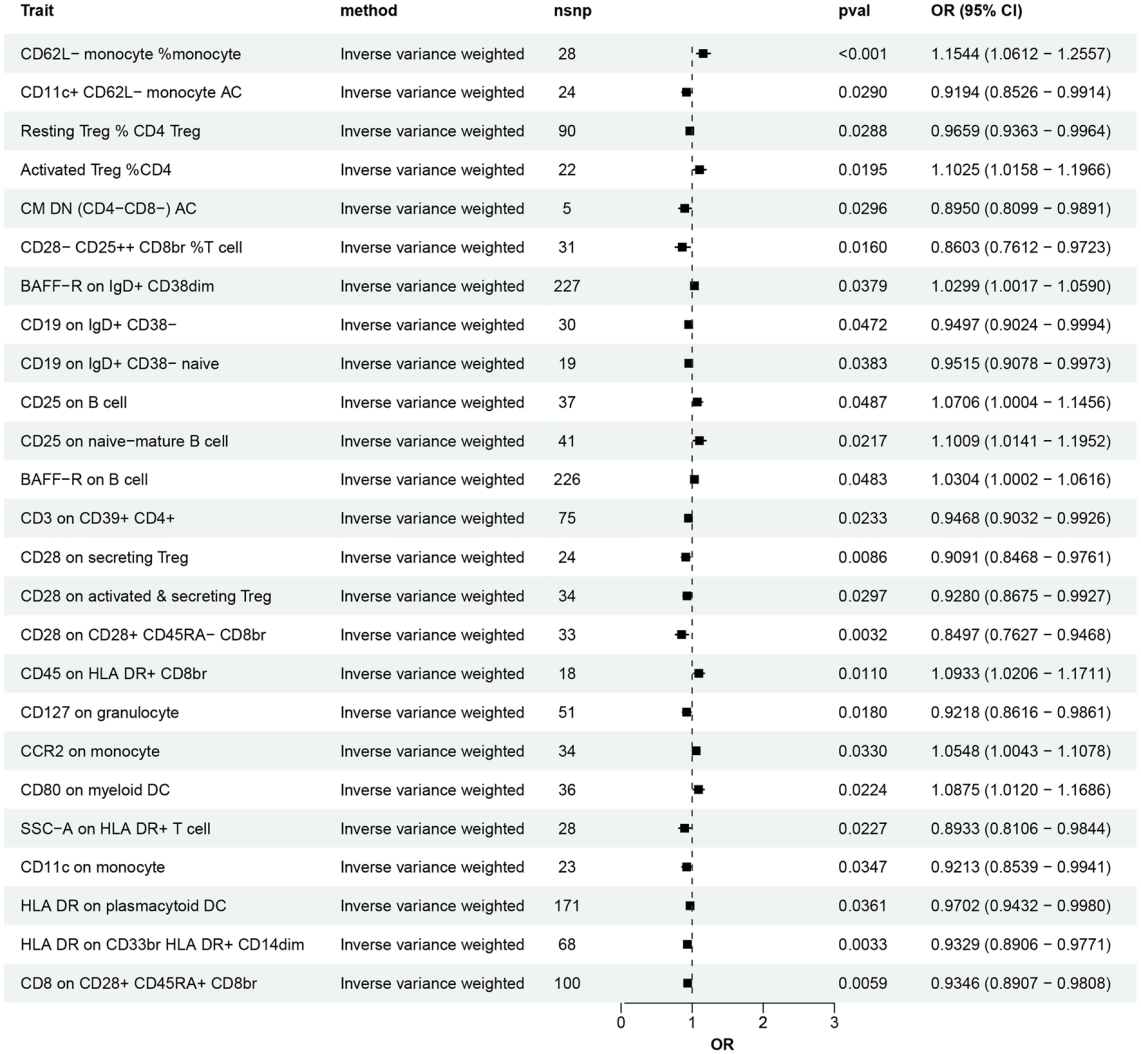
Effects of immune factors on osteonecrosis. Results from MR analyses showing the potential casual effects of 25 immune factors on osteonecrosis with IVW method.

**Figure 2:**
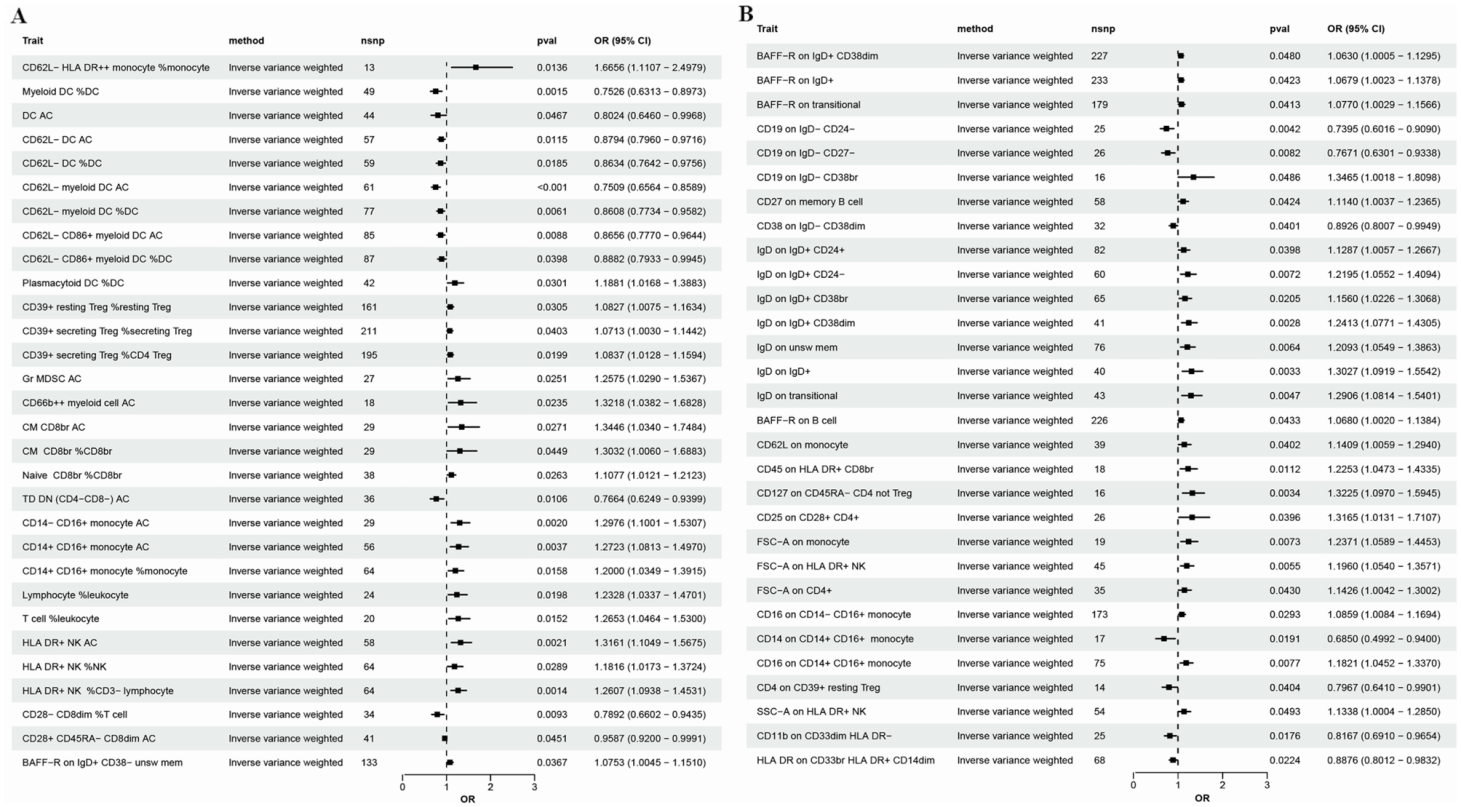
Effects of immune factors on drug induced osteonecrosis. Results from MR analyses showing the potential casual effects of 60 immune factors on drug induced osteonecrosis with IVW method.

**Figure 3:**
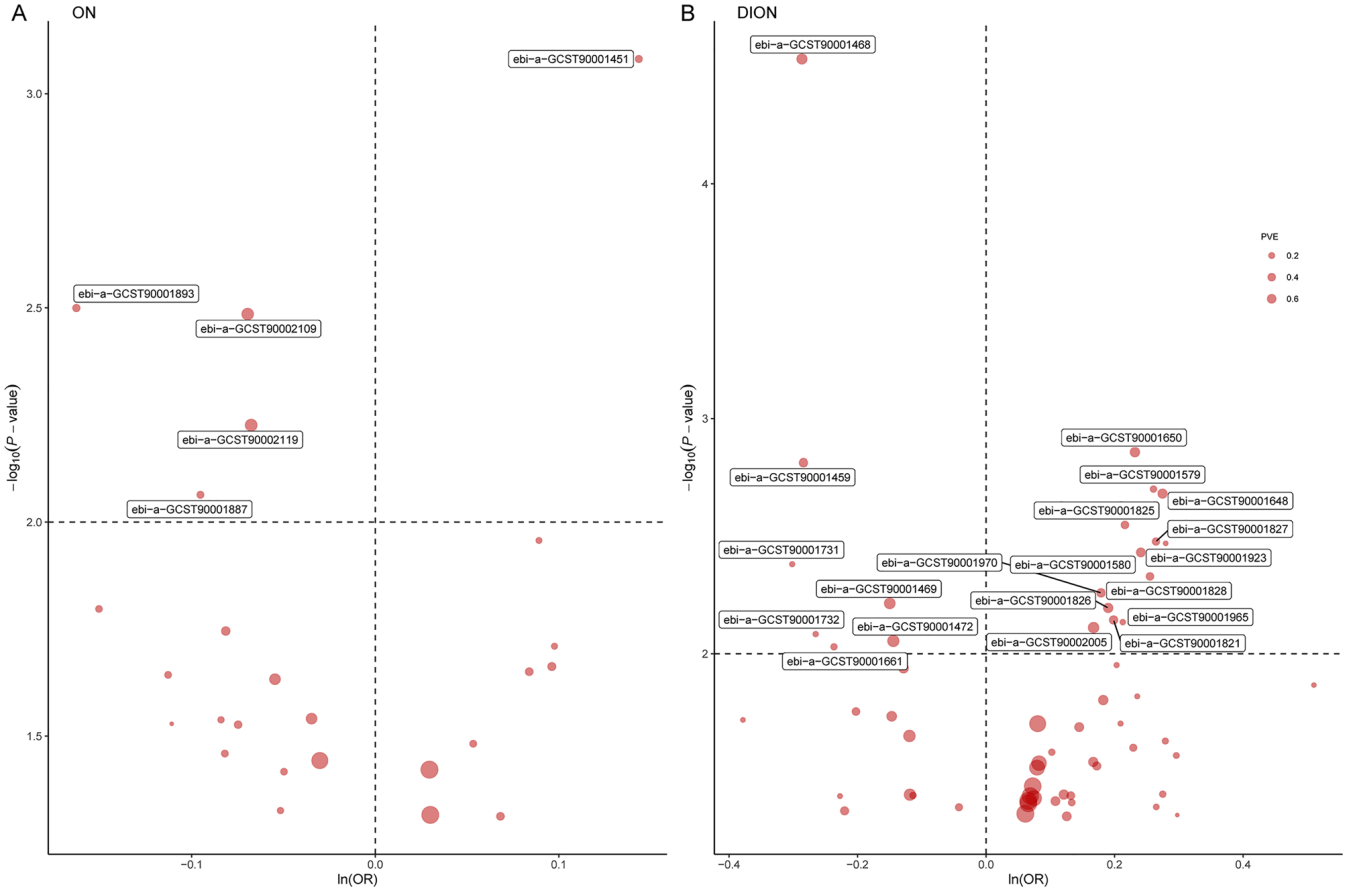
MR results for immune factors and the risk of ON and DION. Volcano plots of the MR results for potential immune factors on the risk of (A) ON and (B) DION. Dashed horizontal black line corresponded to *P* = 0.01. ln = natural logarithm; PVE = proportion of variance explained.

All MR analysis results between the 731 immune traits and the outcomes (ON, including osteonecrosis and drug induced osteonecrosis) are listed in tables S1 and S2. Regarding osteonecrosis, a total of 25 immune traits with potential causal effects were identified (Fig. 1). We classified all immune traits into four types of phenotypes, namely, absolute count AC, median fluorescence intensity MFI, relative count RC, and morphological parameters MP. The detected potential immune traits related to osteonecrosis can be summarized into 2 AC traits, 18 MFI traits, 4 RC traits, and 1 MP trait. Similarly, 60 immune traits were identified to have potential causal relationships with drug-induced osteonecrosis (Fig. 2), including 12 AC traits, 27 MFI traits, 17 RC traits, and 4 MP traits. Volcano plots also showed the MR results for potential immune factors on the risk of ON (Fig. 3).

### The absolute cell counts and ON

In our principal MR IVW analysis, results indicated that the levels of two count-related immune traits (CD11c + CD62L − monocyte and CM DN (CD4 − CD8 −)) have a negative correlation with the risk of osteonecrosis. The direction of the remaining four MR analyses aligns with IVW, while the Q-test, MR-Egger interception, and MR-PRESSO did not detect potential directional pleiotropy. Nevertheless, these correlations were no longer significant after the FDR correction (*q* > 0.2, Tables S1, S3).

**Figure 4:**
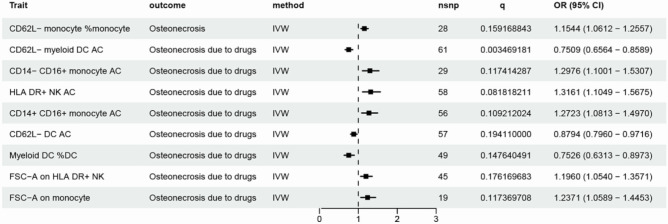
Significant causal effects of immune factors on ON. Results from MR analyses showing the significant casual effects of 9 immune factors on ON with IVW method after FDR correction. ON: osteonecrosis (including osteonecrosis and drug-induced osteonecrosis). FDR: false discovery rate.

Concerning drug-induced osteonecrosis, we identified 12 potentially causally associated count-related immune traits. Among these include 4 in the cDC cell group, 2 in the mature T cell group, 2 in the monocyte group, 2 in the myeloid cell group, 1 in the TBNK cell group, and 1 in the Treg cell group. Following the FDR method's multiple testing adjustment and sensitivity analysis, five immune traits still displayed a significant causal effect under 0.20 significance. As illustrated in Fig. 4 and Table S4, according to the IVW method, both CD62L − myeloid DC (OR: 0.751, *P* = 2.94 × 10^−5^, *q* = 0.003) and CD62L − DC (OR: 0.879, *P* = 1.15 × 10^−2^, *q* = 0.194) consistently exhibited a negative correlation with drug-induced osteonecrosis risk. Yet, features (namely CD14 − CD16 + monocyte, CD14 + CD16 + monocyte, and HLA DR + NK) were positively correlated with drug-induced osteonecrosis.

### Median fluorescence intensities and ON

Suggestive significances between 18 MFI traits and osteonecrosis and 27 and drug-induced osteonecrosis are illustrated in Fig. 1 and Tables S1 and S2 (*P* < 0.05). Regarding osteonecrosis, the traits of the B-cell group and Treg cell group bear the highest number of significant associations compared to other groups, while as many as 17 pairs of features derive from the B-cell group in drug-induced osteonecrosis. In the B-cell group, each subgroup was further differentiated into various immune traits according to the expression of different immune factors. Among these findings, CD19, IgD, and BAFF-R were the most frequently expressed molecules in different types of B-cell subgroups.

### The relative cell counts or the morphological parameter and ON

Utilizing IVW-MR analysis, suggestive associations (*P* < 0.05) were attained between four pairs of RC/osteonecrosis and seventeen pairs of RC/drug induced osteonecrosis. Among these, horizontal pleiotropy was detected in MR-Egger regression with regard to two features related to drug-induced osteonecrosis (CD62L − HLA DR + + monocyte %monocyte and CD62L − CD86 + myeloid DC %DC). For the remaining results, Cochran’s Q test for IVW did not yield any significant heterogeneity among these IVs. Moreover, no significant directional horizontal pleiotropy was observed based on MR-Egger regression intercept analysis. Furthermore, subsequent MR-PRESSO analysis detected no substantial outliers (global test *P* > 0.05, Tables S5, S6). After FDR adjustment, supporting evidence was found for the correlation between elevated immunologic feature CD62L − monocyte %monocyte and an increased osteonecrosis risk (OR:1.154, *q* = 0.159), while the immunologic feature Myeloid DC %DC was negatively associated with the risk of drug-induced osteonecrosis (OR:0.753, *q* = 0.148).

In the MP group, potential causal significances were observed between one pair and four pairs of immune features with osteonecrosis and drug-induced osteonecrosis respectively. Following FDR multiple corrections, two immune features, namely FSC-A on HLA DR + NK (OR:1.196, *q* = 0.176) and FSC-A on monocyte (OR:1.237, *q* = 0.117), remained significantly associated with drug-induced osteonecrosis risk.

### The Venn diagram

**Figure 5:**
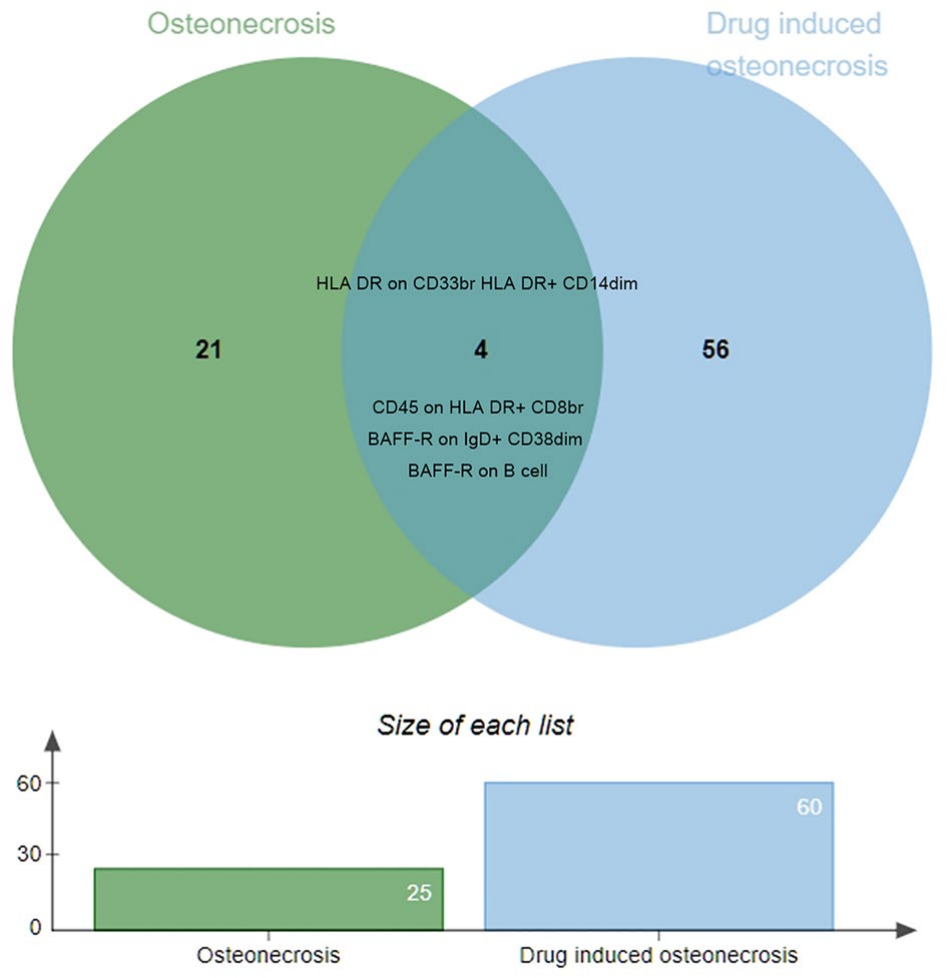
The Venn diagram of the intersection of all potential causative immune factors within the two data sets.

An online tool, http://www.bioinformatics.com.cn/static/others/jvenn/, was utilized to determine the intersection of all potential causative immune factors within the two data sets. The results indicated that CD45 on HLA DR + CD8br, BAFF-R on IgD + CD38dim and BAFF-R on B cell are common risk factors for osteonecrosis and drug-induced osteonecrosis, while HLA DR on CD33br HLA DR + CD14dim serves as a common protective feature for both (Fig. 5).

### Causal effect of ON on immune factors

To examine reverse causal effects, 19/13 SNPs that were robustly and independently associated with osteonecrosis/drug-induced osteonecrosis were extracted. Tables S7 and S8 showcase the MR analysis results of the relationship between ON and immune factors. The IVW method depicted potential causal associations between osteonecrosis and 31 immune factors, and between drug-induced osteonecrosis and 25 immune factors. No heterogeneity or horizontal pleiotropy was detected among these results (Tables S9, S10). However, post FDR correction, these correlations were no longer significant (*q* > 0.2).

## Discussion

In the realm of osteonecrosis, early intervention typically yields more conspicuous results^27^. However, diagnosing it during its initial stages is difficult due to the lack of overt clinical symptoms. Predominantly, osteonecrosis tends to affect younger patients, and in such cases, treatment ought to aim for joint preservation whilst postponing a joint replacement procedure. Thus, early diagnosis and prompt treatment of osteonecrosis stands paramount^1^. Our study is the first to employ MR analysis based on extensive GWAS data to delve into the potential causal relationship between 731 immune factors and osteonecrosis/drug-induced osteonecrosis. It was discovered that after correction for FDR, genetic prediction of level for a distinct immune factor (CD62L − monocyte %monocyte) showed a notable positive correlation with osteonecrosis, whereas eight other immune factors bore a significant causal effect with drug-induced osteonecrosis. Identification of these immune factors could potentially contribute towards early diagnosis, treatment and prevention of the disease. In this manuscript, we will primarily focus our discussion on these immune factors. In addition to this, although no significant causal links were discovered post FDR adjustments, reverse MR identified potential causal effects between osteonecrosis/drug-induced osteonecrosis and 31/25 immune factors, which may bear significance in disease prognosis.

Osteoimmunology is a relatively new branch within immunology and research regarding the link between the immune system and osteonecrosis is scarce^28^. Previous reports have associated macrophage presence with bisphosphonate-related osteonecrosis of the jaw and steroid-induced osteonecrosis^29,30^. Adapala et al., employing a piglet model of Legg-Calvé-Perthes disease, found that necrotic bone triggered an inflammatory response in macrophages by activating TLR4^31^. A retrospective clinical control study depicted a significant increase in total lymphocyte count, CD3 + T cells, T cells (CD3 + CD8 +), B-1 cell count and B-1 cells (CD5 + CD19 +) in peripheral blood of ONFH patients^32^. However, observational studies often succumb to confounding factors, reverse causation, limited follow-up duration and small sample size^33^. Concurrently, existing research lacks detailed classification of immune cells and their subclasses, thereby making comprehensive understanding a challenge. In our estimation, monitoring changes in different immune cell subclasses is pivotal to fully understanding peripheral immune abnormalities in osteonecrosis.

Monocytes perform pivotal roles within the body; from immune surveillance, inflammatory responses to tissues repair. Our research reveals significant positive correlation between the immune trait CD62L − monocyte %monocyte and risk of osteonecrosis in relative count groups—this relative count reflects its proportion vis-à-vis the corresponding progenitor cell lineage, essentially providing a ratio of CD62L − monocytes to monocytes. CD62L (the L-selectin), an I-type transmembrane glycoprotein and cell adhesion molecule, facilitates recognition and adhesion between white cells and vascular endothelial cells, enabling white cells to traverse through vascular walls to tissues, thus participating in immune responses^34^. Studies suggest that aberrations in the expression or functionality of CD62L could potentially trigger immune disorders and onset of numerous medical conditions, spanning autoimmune diseases (such as rheumatoid arthritis, systemic lupus erythematosus) and infectious illnesses (like AIDS, tuberculosis) ^35–38^. Consequently, an amplified ratio of CD62L − monocytes to monocytes might induce monocyte migration impediments, compromising the normal functionality of the immune system, eventually accelerating the onset of osteonecrosis. Within the absolute count group, monocyte-related immune traits (CD14 − CD16 + monocyte AC, CD14 + CD16 + monocyte AC) have been identified as risk parameters for drug-induced osteonecrosis. Human monocytes are classified into three distinct types based on surface receptors: classic monocytes (CD14 + CD16 −), intermediate monocytes (CD14 + CD16 +) and non-classic monocytes (CD14 − CD16 +)^39^. The intermediate and non-classic monocytes, representing two major monocyte subclasses, exhibit diverse functionalities in immune responses and inflammatory procedures. Intermediate monocytes (CD14 + CD16 +) possess a profound capacity to produce inflammatory factors, such as TNF-α and IL-1β, these cytokines might exacerbate the damages in bone tissue, thereby precipitating the occurrence of osteonecrosis^40^. Additionally, intermediate monocytes could also potentially differentiate into osteoclast precursors, participating in bone resorption procedures^12,41,42^. The role of non-classic monocytes (CD14 − CD16 +) in immune regulation and angiogenesis appears to be a contentious area. Whilst some studies suggest that these cells primarily maintain vascular homeostasis and promote inflammation resolution^43,44^, others have demonstrated contrasting implications in chronic inflammations and immune diseases, including atherosclerosis, systemic lupus erythematosus^45,46^, which aligns with our findings. Forward scatter area (FSC-A) commonly employed in flow cytometry reflects cell size and complexity^47^, our findings depict positive correlation between FSC-A on monocytes and drug induced osteonecrosis within the morphological parameters group. This research substantiates the significant role of monocytes in pathogenesis of osteonecrosis. Future research warrant thorough exploration into the specific molecular mechanisms of these cell subclasses in osteonecrosis, as well as examining their interplay with other immune cells and cytokines.

In this study, through MR, we observed a negative correlation between myeloid dendritic cells (Myeloid DC %DC), CD62L − myeloid dendritic cells (CD62L − myeloid DC AC) and CD62L − dendritic cells (CD62L − DC AC) and drug induced osteonecrosis. Myeloid dendritic cells, a significant class of antigen presentation cells, hold the capacity to activate T cells and regulate immune responses which potentially inhibit inflammatory responses and defend bone tissue during the pathogenesis of osteonecrosis^48^. Moreover, these cells can secrete a slew of cytokines, such as IL-10 and TGF-β, that bear anti-inflammatory properties and could thereby mitigate inflammation-related damage arising from osteonecrosis^49^. As a pivotal component of the immune system, dysfunction of DCs has a profound correlation with the incidence and progression of multiple diseases. In osteonecrosis, functionality deterioration of DCs could lead to a dysregulation of the immune response, subsequently impairing bone tissue repair and reconstruction^50^. Thus, our revelations imply maintaining the normal functionality of DCs could be critical in preventing or slowing down the development of osteonecrosis. These findings insinuate ameliorating the functioning of myeloid DCs and DCs could be a potential strategy in osteonecrosis treatment. Future research should delve deeper into the specific roles these cells play in osteonecrosis, and how their functionality could be manipulated to alleviate clinical symptoms of osteonecrosis.

Natural killer (NK) cells represent one of the three major lymphocyte lineages (T, B, and NK cells) in humans. Within the bone microenvironment, NK cells, by secreting various pro-inflammatory and chemotactic factors, engage in interactions with other immune cells, hematopoietic stem cells, and stromal cells^51^. Numerous studies have espoused an intimate link between NK cells and osseous pathology^52,53^. Our study intimates a positive correlation between both the absolute count (HLA DR + NK AC) and forward scatter area (FSC-A on HLA DR + NK) of HLA DR + NK cells and drug-induced osteonecrosis. Implying an activated cell state, HLA DR + NK cells, a subclass of NK cells, were found to be prolific in cytokine production, such as IFN-γ and TNF-α, which play a role in bone remodeling and osteoclast activation^54,55^. Moreover, activation of these HLA DR + NK cells might potentially trigger or exacerbate an inflammatory cascade^56^, thereby disrupting bone homeostasis and vascular systems, leading to bone ischemia and necrosis. In summary, the relationship between these HLA DR + NK cells and osteonecrosis could be multi-faceted, involving both direct and indirect impacts on bone tissue integrity, blood supply, and immune regulation. Additional research is necessary to delineate precise pathways and determine whether these mechanisms are the prime cause or secondary influences of osteonecrosis in light of other potential conditions.

Among all immune markers with potential causal effects, four were identified as having causal effects in the same direction for both osteonecrosis and drug-induced osteonecrosis. The results suggests that immune traits CD45 on HLA DR + CD8br, BAFF-R on IgD + CD38dim and BAFF-R on B cell are shared risk factors for both osteonecrosis and drug-induced osteonecrosis, whereas HLA DR on CD33br HLA DR + CD14dim is a shared protective factor. These findings may provide a novel perspective for understanding the common immune regulation mechanisms between osteonecrosis and drug-induced osteonecrosis and may inspire the development of new therapeutic strategies.

However, this investigation is subject to several limitations. Primarily, in MR studies, data from diverse racial, ethnic, or geographic backgrounds may introduce bias due to potential differences in genetic backgrounds, environmental exposures, lifestyles, and health conditions, leading to population stratification bias^57^. Although the cohorts in our study predominantly consist of European ancestry, data related to ON and drug-induced ON were chiefly derived from Finnish participants, which still poses a risk of population stratification bias. Moreover, our findings might not be applicable across other racial backgrounds, necessitating validation through GWAS data from other populations or within-family GWAS analyses in the future^57,58^. Secondly, the ON GWAS data employed in our study spanned various types of ON, and the absence of consolidated GWAS data for each specific type hindered the validation of our discoveries across additional independent GWAS, potentially constraining the interpretability and generalizability of our results. Thirdly, despite selecting highly correlated SNPs to represent the genetic variation of exposure, as demonstrated by the PVE in Fig. 3 and Supplementary Tables S5, S6, it only explained a fraction of the 731 immune factors and should not be considered an exact substitute for exposure. Furthermore, although bidirectional MR provides an important alternative method for validating causal effects, determining the direction of causal relationships remains challenging, thus confirmation may still necessitate randomized controlled trials. Future research endeavors necessitate the adoption of more comprehensive strategies to further corroborate our findings and address these potential confounding issues.

## Conclusion

All in all, through the use of genetic variations as IVs, the study provides genetic evidence for a causal link between a wide range of immune factors and ON. It help unravels the intricate interaction patterns between the immune and skeletal systems, elucidates the pathogenesis of osteonecrosis, and identifies potential new treatments. The potential mechanistic underpinnings of the detected causal associations necessitate further foundational and clinical research to bridge the theory–practice gap.

### Supplementary Information

Supplementary Table S1.

Supplementary Table S2.

Supplementary Table S3.

Supplementary Table S4.

Supplementary Table S5.

Supplementary Table S6.

Supplementary Table S7.

Supplementary Table S8.

Supplementary Table S9.

Supplementary Table S10.

Supplementary Information 11.

Supplementary Information 12.

## Data Availability

The data underlying this article are available in the article and in its online supplementary material.

## Abbreviations

MR: Mendelian randomization
GWAS: Genome-wide association study
IVW: Inverse-variance weighted
ONFH: Osteonecrosis of the femoral head
THAs: Total hip arthroplasties
SNPs: Single nucleotide polymorphisms
IVs: Instrumental variables
AC: Absolute cell numbers
MFI: Median fluorescence intensity
RC: Relative cell numbers
MP: Morphological parameters
MAFs: Minor allele frequencies
FDR: False discovery rate
PVE: Proportion of variance explained
