# Supplementary material for: Identifying the causal relationship between immune factors and osteonecrosis: a two-sample Mendelian randomization study
Source: Sci Rep. 2024 Apr 23;14:9371. doi: 10.1038/s41598-024-59810-0 (PMC11039661; doi:10.1038/s41598-024-59810-0)
Supplement: Supplementary file 11 — Supplementary Information 11. [file 41598_2024_59810_MOESM11_ESM.pdf]

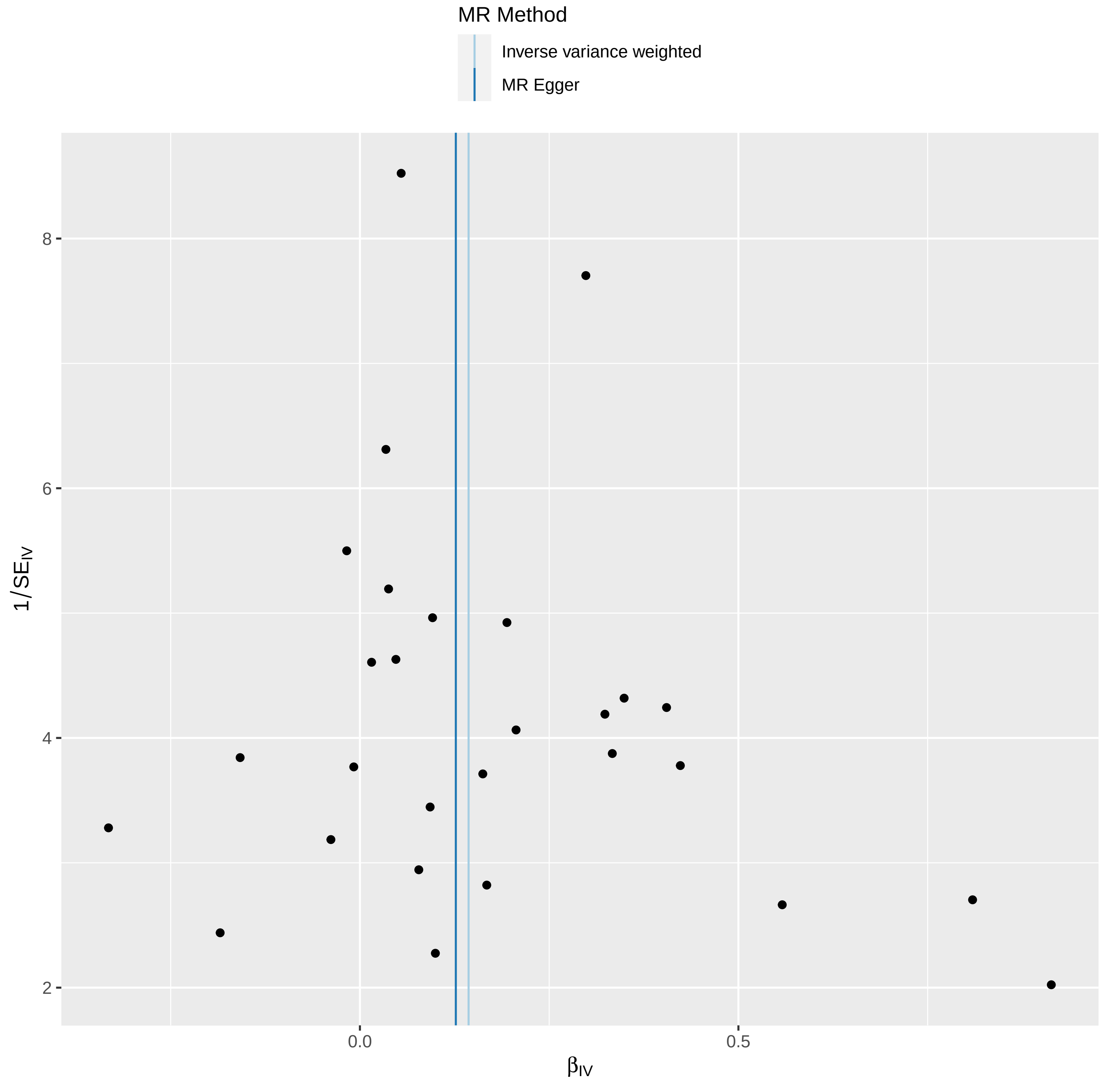

MR Method
Inverse variance weighted
MR Egger
8
6
4
2
$1/SE_{IV}$
0.0
0.5
β_{IV}

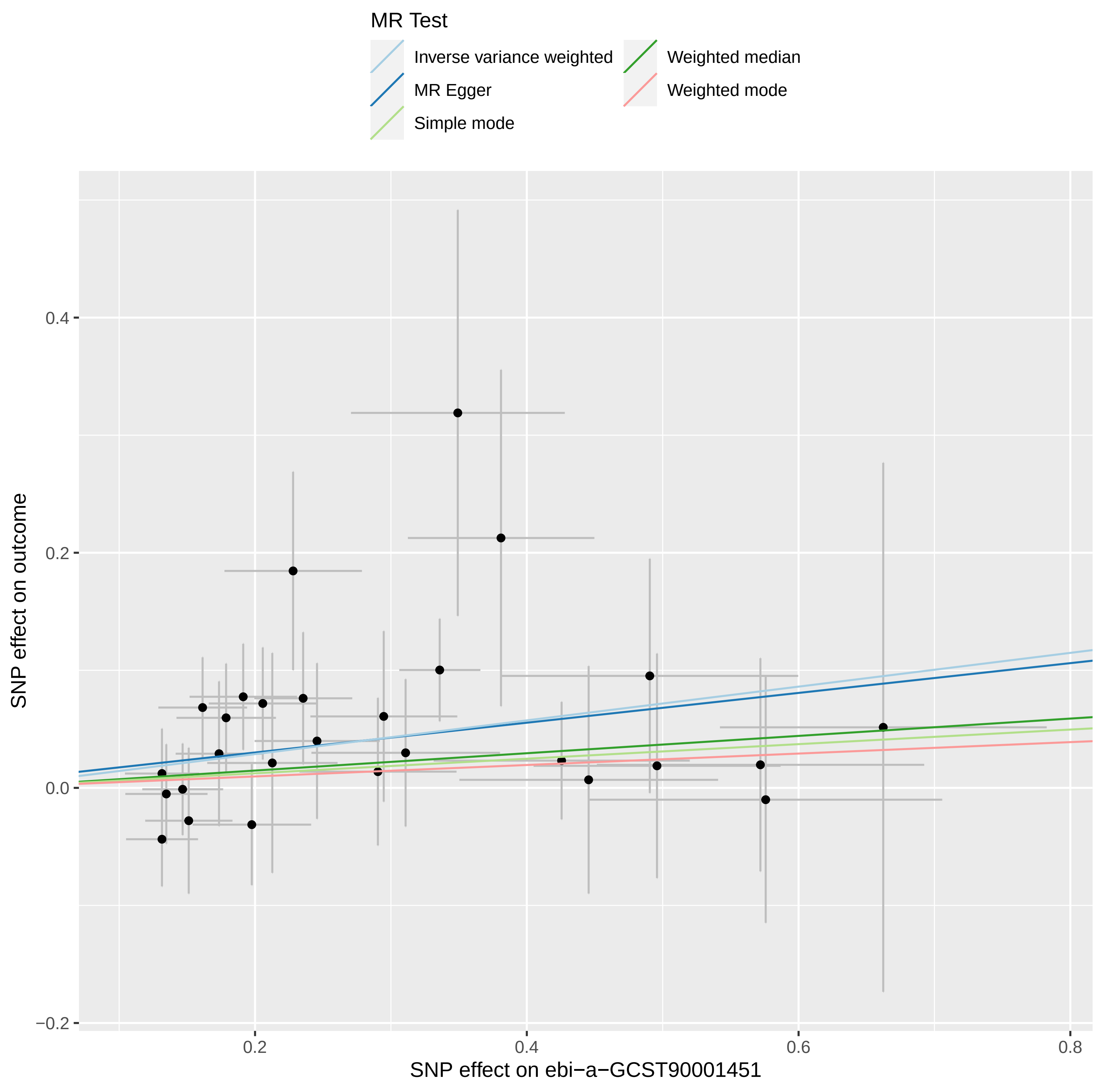
MR Test
Inverse variance weighted
MR Egger
Simple mode
Weighted median
Weighted mode
SNP effect on outcome
0.4
0.2
0.0
−0.2
0.2
0.4
0.6
0.8
SNP effect on ebi−a−GCST90001451

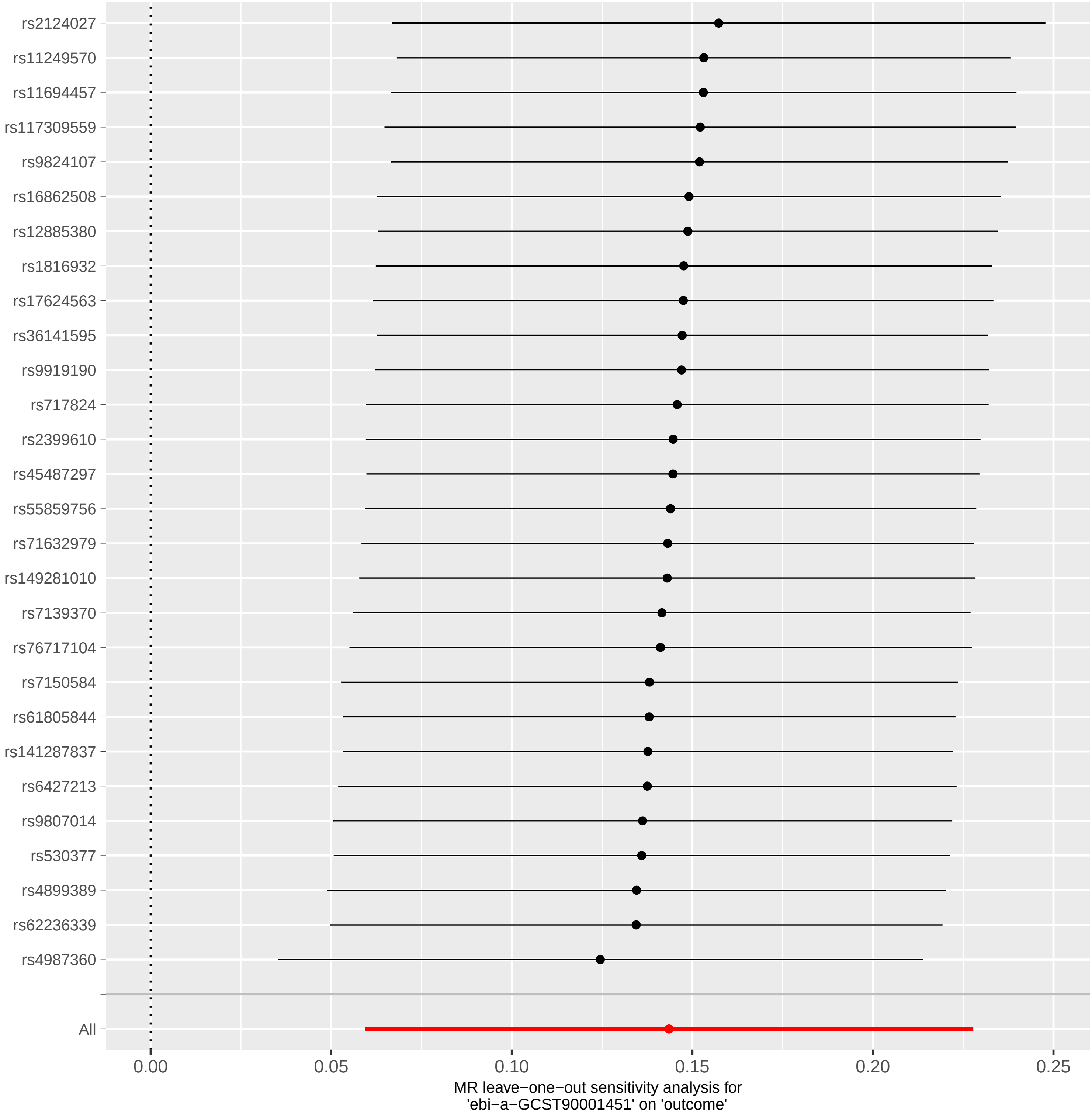
rs2124027
rs11249570
rs11694457
rs117309559
rs9824107
rs16862508
rs12885380
rs1816932
rs17624563
rs36141595
rs9919190
rs717824
rs2399610
rs45487297
rs55859756
rs71632979
rs149281010
rs7139370
rs76717104
rs7150584
rs61805844
rs141287837
rs6427213
rs9807014
rs530377
rs4899389
rs62236339
rs4987360
All
0.00
0.05
0.10
0.15
0.20
0.25
MR leave−one−out sensitivity analysis for
'ebi−a−GCST90001451' on 'outcome'

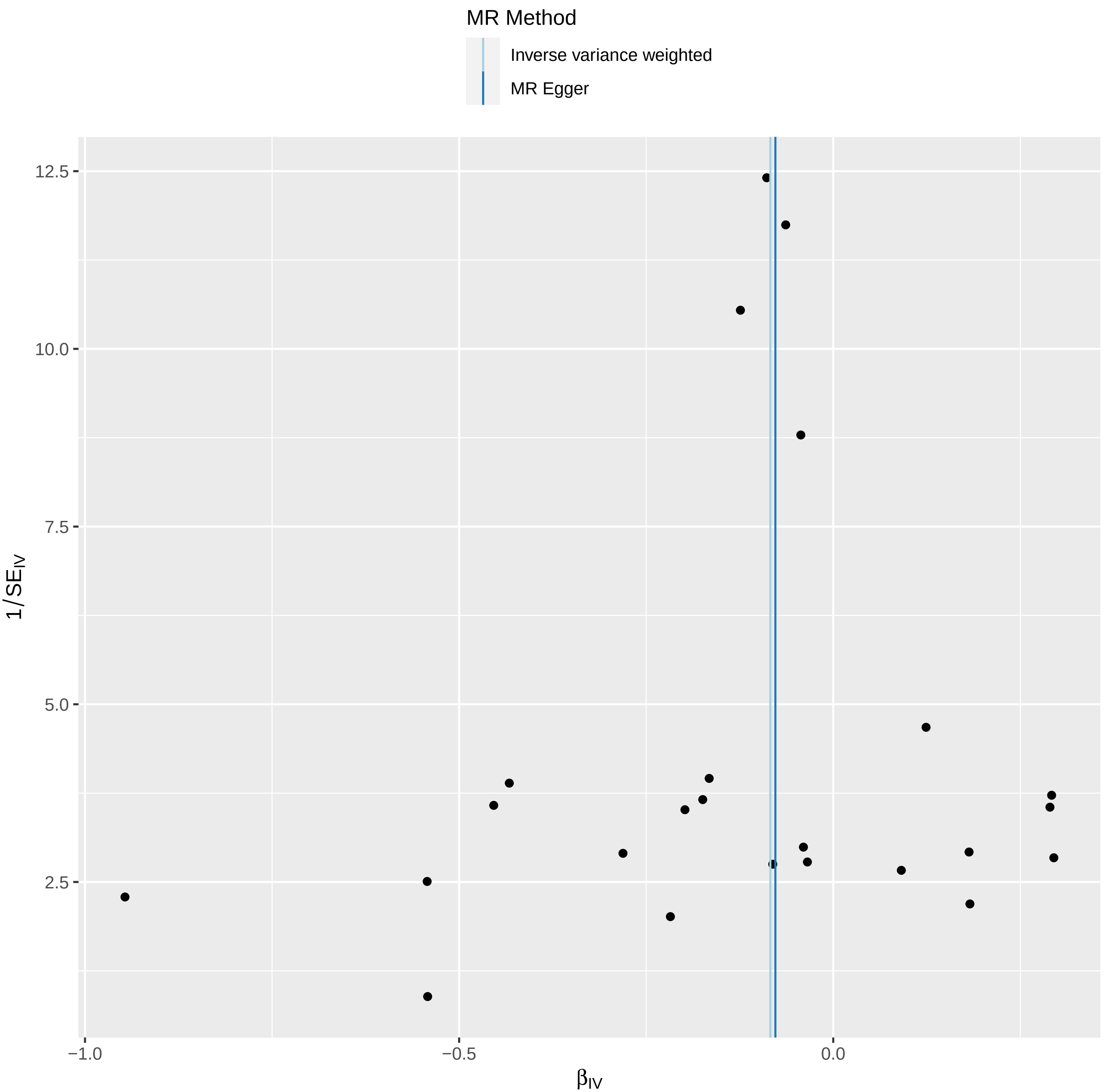

MR Method
Inverse variance weighted
MR Egger
12.5
10.0
7.5
5.0
2.5
1/SE$_{IV}$
−1.0
−0.5
0.0
β$_{IV}$

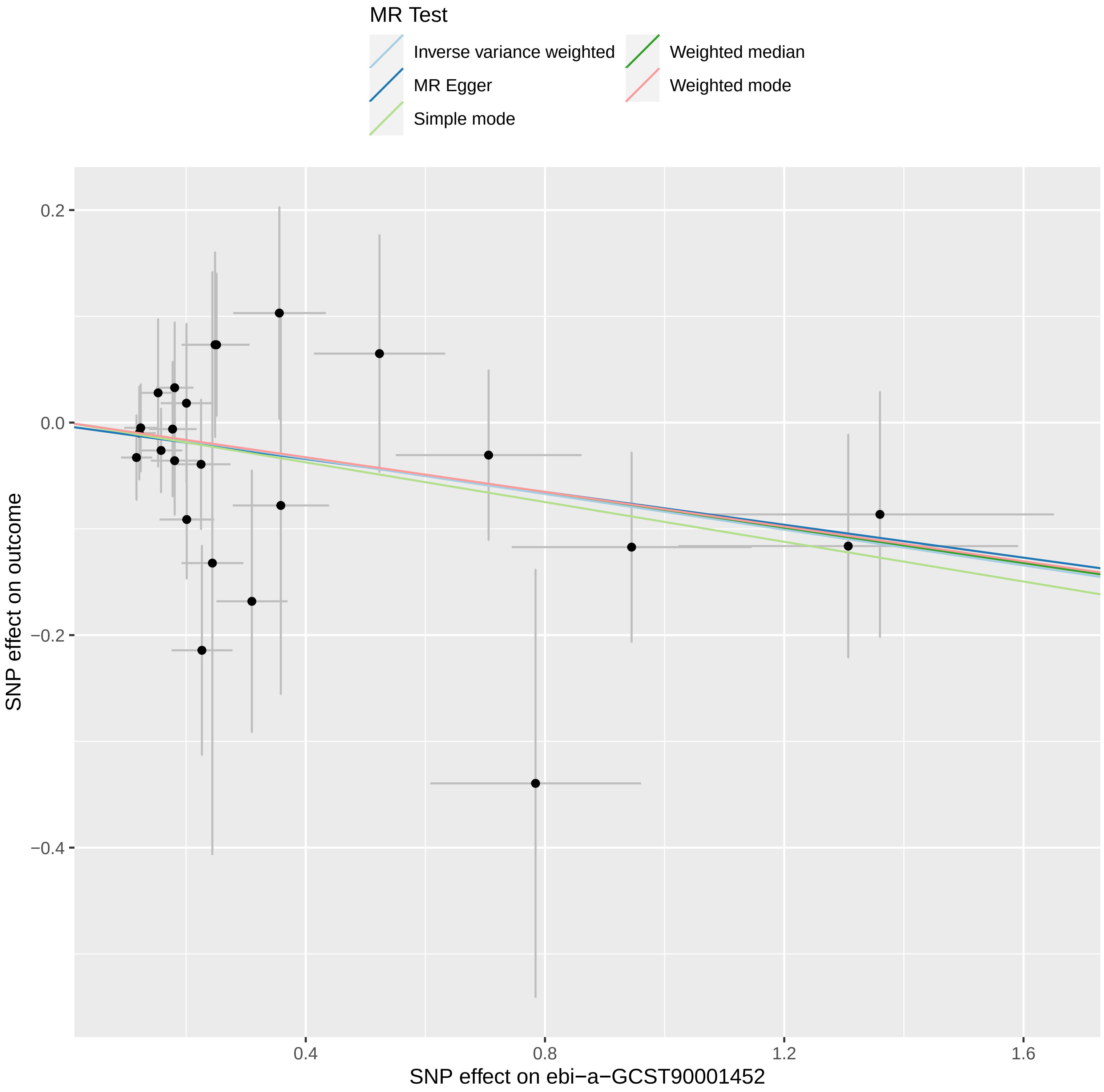
MR Test
Inverse variance weighted
MR Egger
Simple mode
Weighted median
Weighted mode
0.2
0.0
−0.2
−0.4
0.4
0.8
1.2
1.6
SNP effect on outcome
SNP effect on ebi−a−GCST90001452

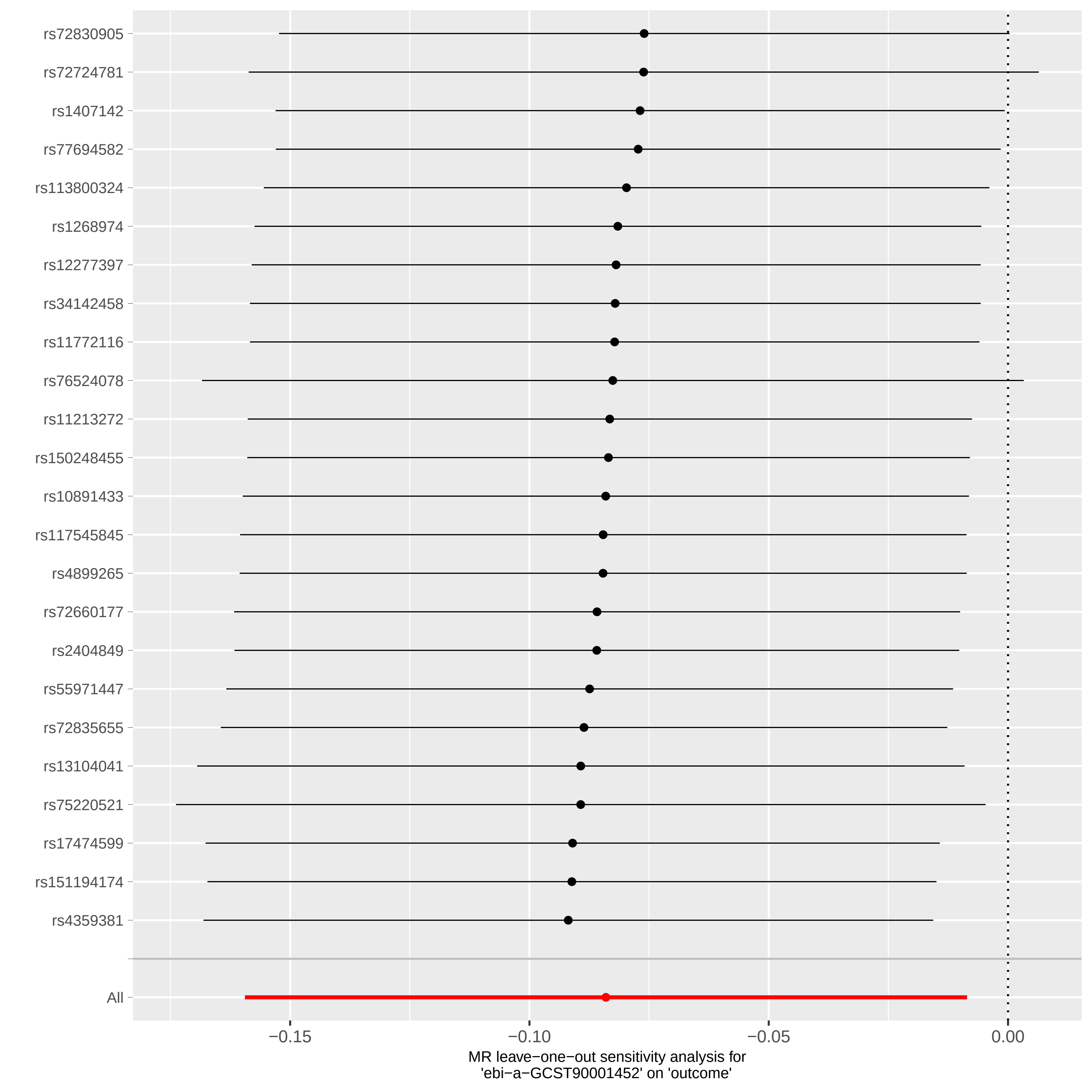
rs72830905
rs72724781
rs1407142
rs77694582
rs113800324
rs1268974
rs12277397
rs34142458
rs11772116
rs76524078
rs11213272
rs150248455
rs10891433
rs117545845
rs4899265
rs72660177
rs2404849
rs55971447
rs72835655
rs13104041
rs75220521
rs17474599
rs151194174
rs4359381
All
−0.15
−0.10
−0.05
0.00
MR leave−one−out sensitivity analysis for
'ebi−a−GCST90001452' on 'outcome'

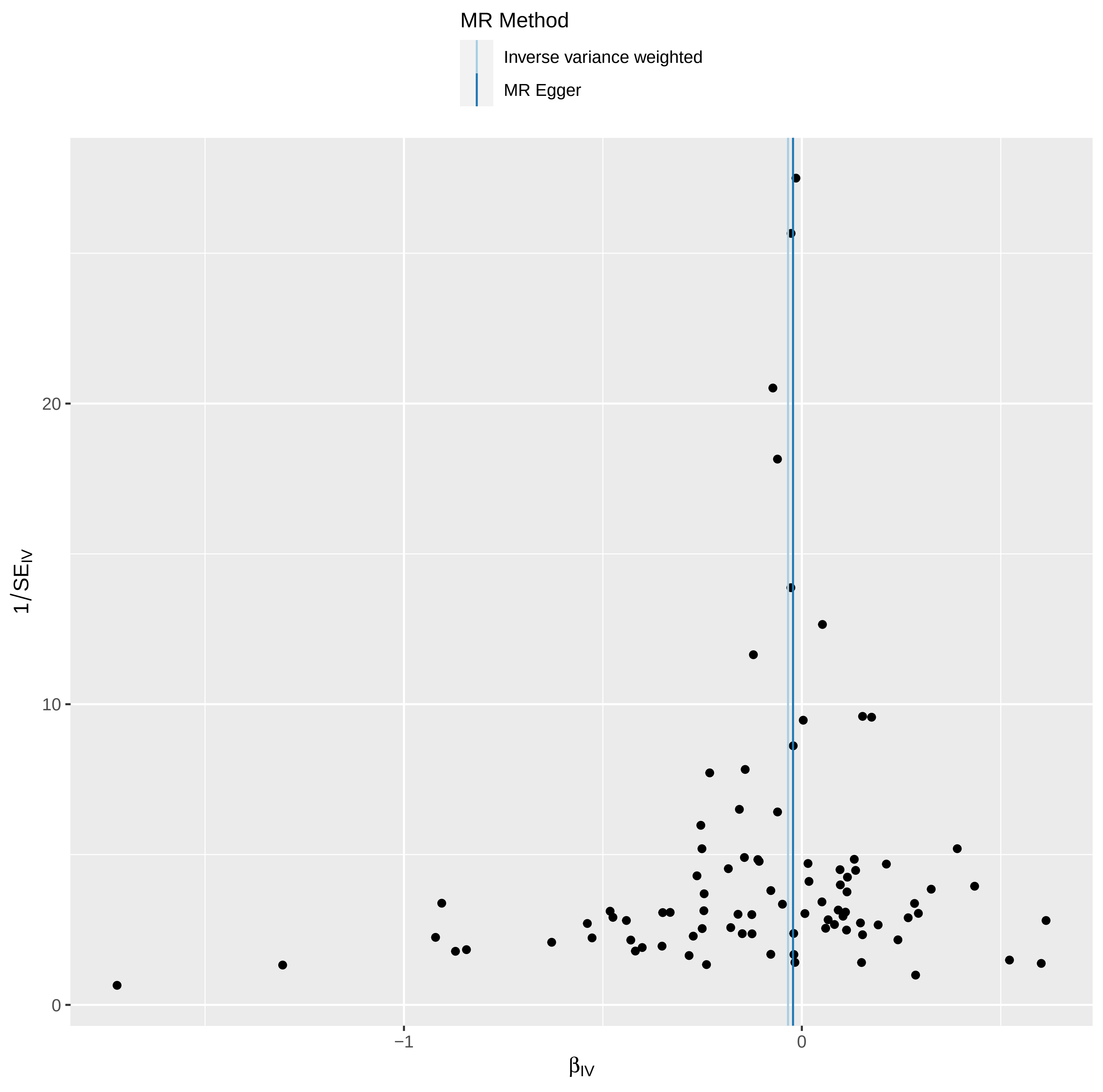
MR Method
Inverse variance weighted
MR Egger
20
10
0
−1
0
1/SE$_{IV}$
β$_{IV}$

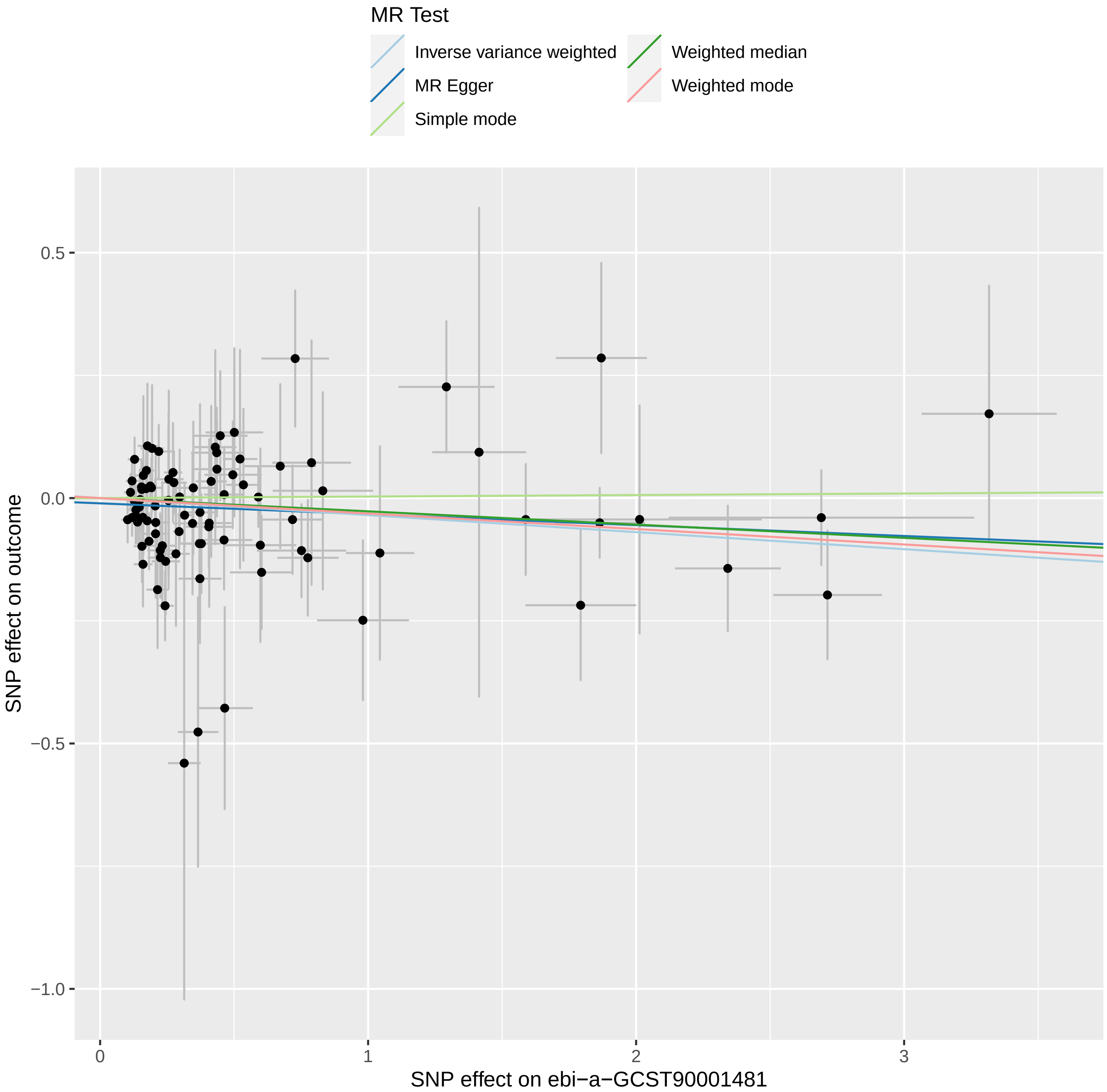
MR Test
Inverse variance weighted
MR Egger
Simple mode
Weighted median
Weighted mode
0.5
0.0
−0.5
−1.0
0
1
2
3
SNP effect on outcome
SNP effect on ebi−a−GCST90001481

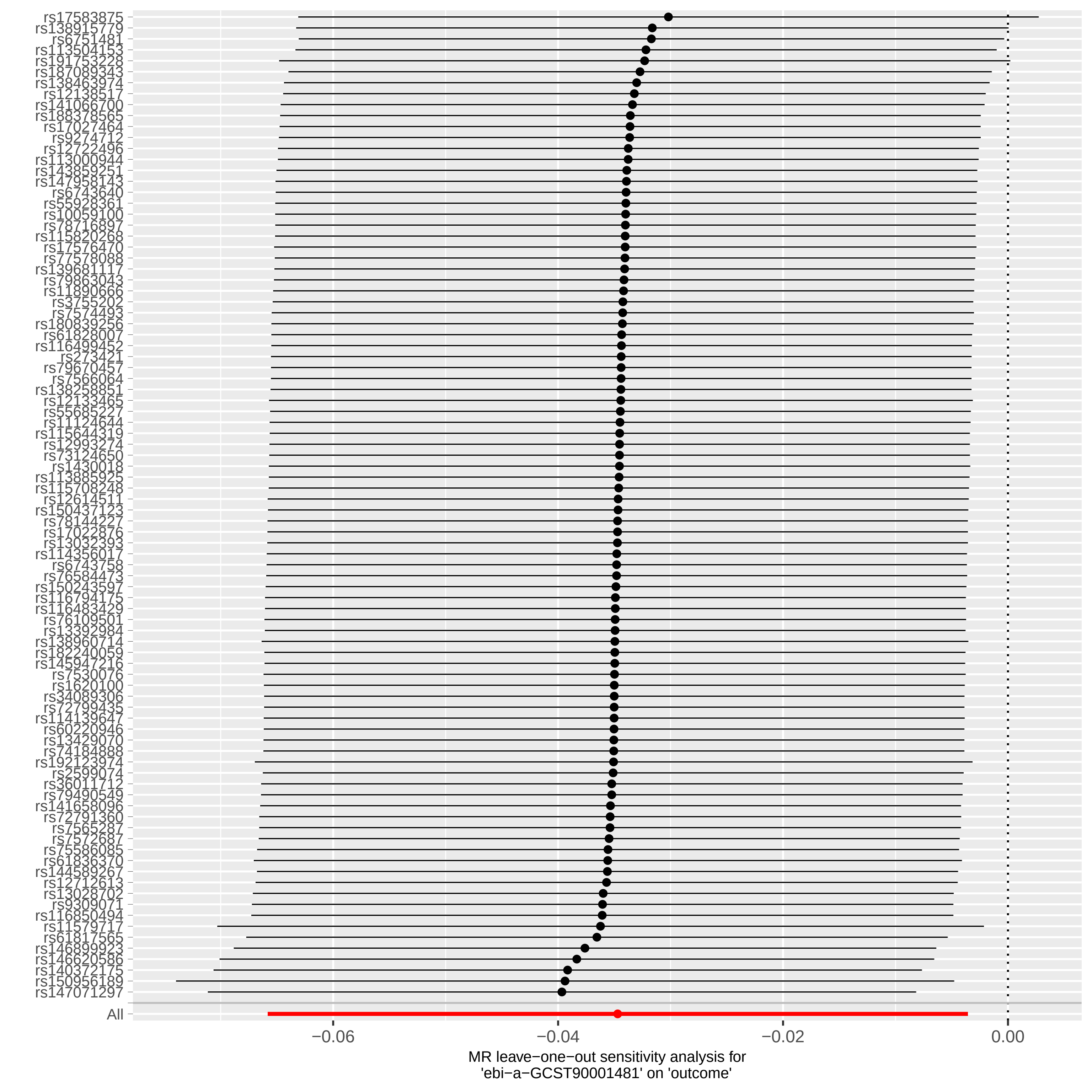
rs17583875
rs138915779
rs6751481
rs113504153
rs191753228
rs187089343
rs138463974
rs12138517
rs141066700
rs188378565
rs17027464
rs9274712
rs12722496
rs113000944
rs143859251
rs147958143
rs6743640
rs55928361
rs10059100
rs78716897
rs115820268
rs17576470
rs77578088
rs139681117
rs79863043
rs11890666
rs3755202
rs7574493
rs180839256
rs61828007
rs116499452
rs273421
rs79670457
rs7566064
rs138258851
rs12133465
rs55685227
rs11124644
rs115644319
rs12993274
rs73124650
rs1430018
rs113885925
rs115708248
rs12614511
rs150437123
rs78144227
rs17022876
rs13032393
rs114356017
rs6743758
rs76584473
rs150243597
rs116794175
rs116483429
rs76109501
rs13392984
rs138960714
rs182240059
rs145947216
rs7530076
rs1620100
rs34089306
rs72799435
rs114139647
rs60220946
rs13429070
rs74184888
rs192123974
rs2599074
rs36011712
rs79490549
rs141658096
rs72791360
rs7565287
rs7572687
rs75586085
rs61836370
rs144589267
rs12712613
rs13028702
rs9309071
rs116850494
rs11579717
rs61817565
rs146899923
rs146620586
rs140372175
rs150956189
rs147071297
All
−0.06
−0.04
−0.02
0.00
MR leave−one−out sensitivity analysis for
'ebi−a−GCST90001481' on 'outcome'

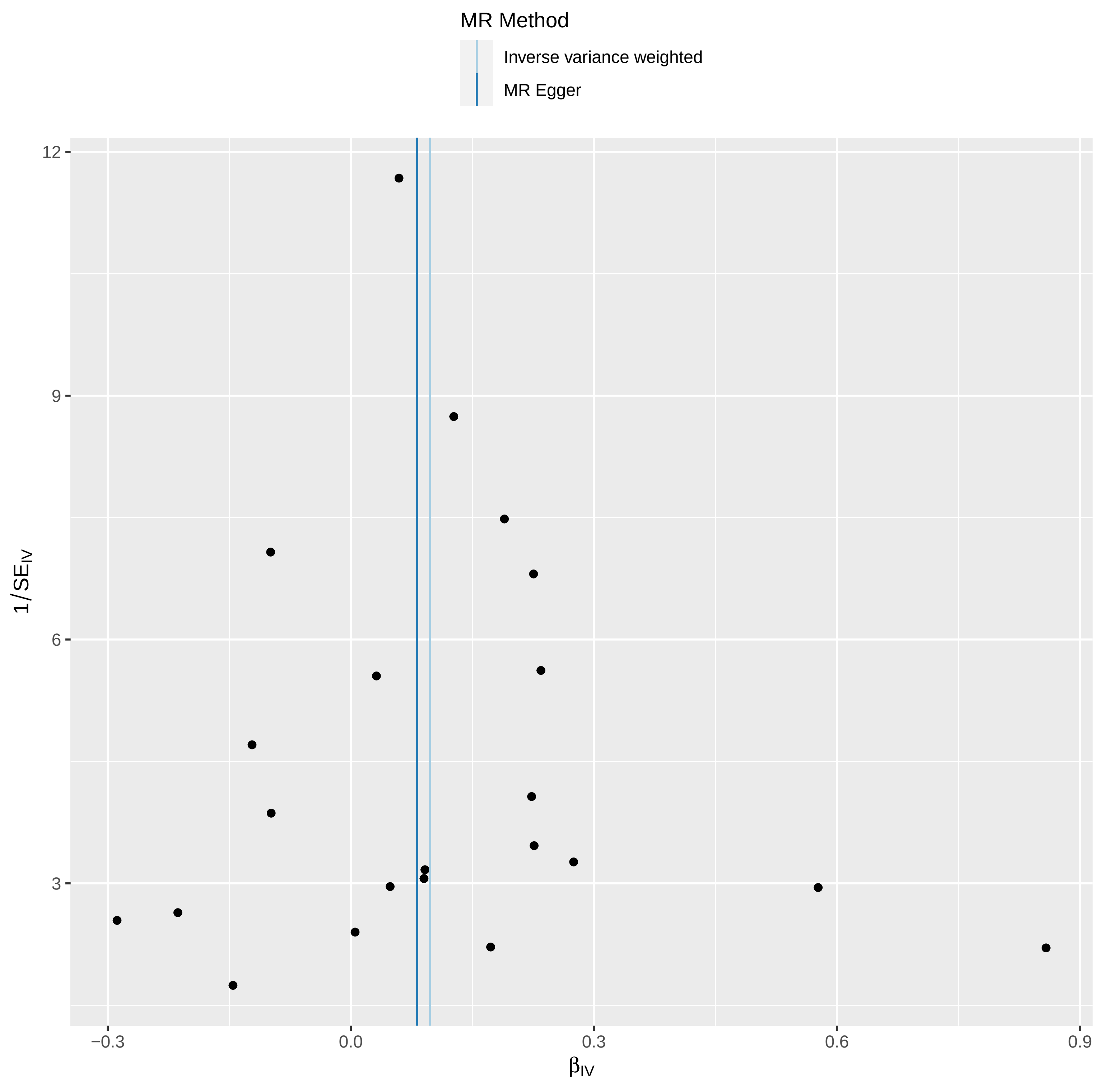

MR Method
Inverse variance weighted
MR Egger
12
9
6
3
1/SE_IV
−0.3
0.0
0.3
0.6
0.9
β_IV

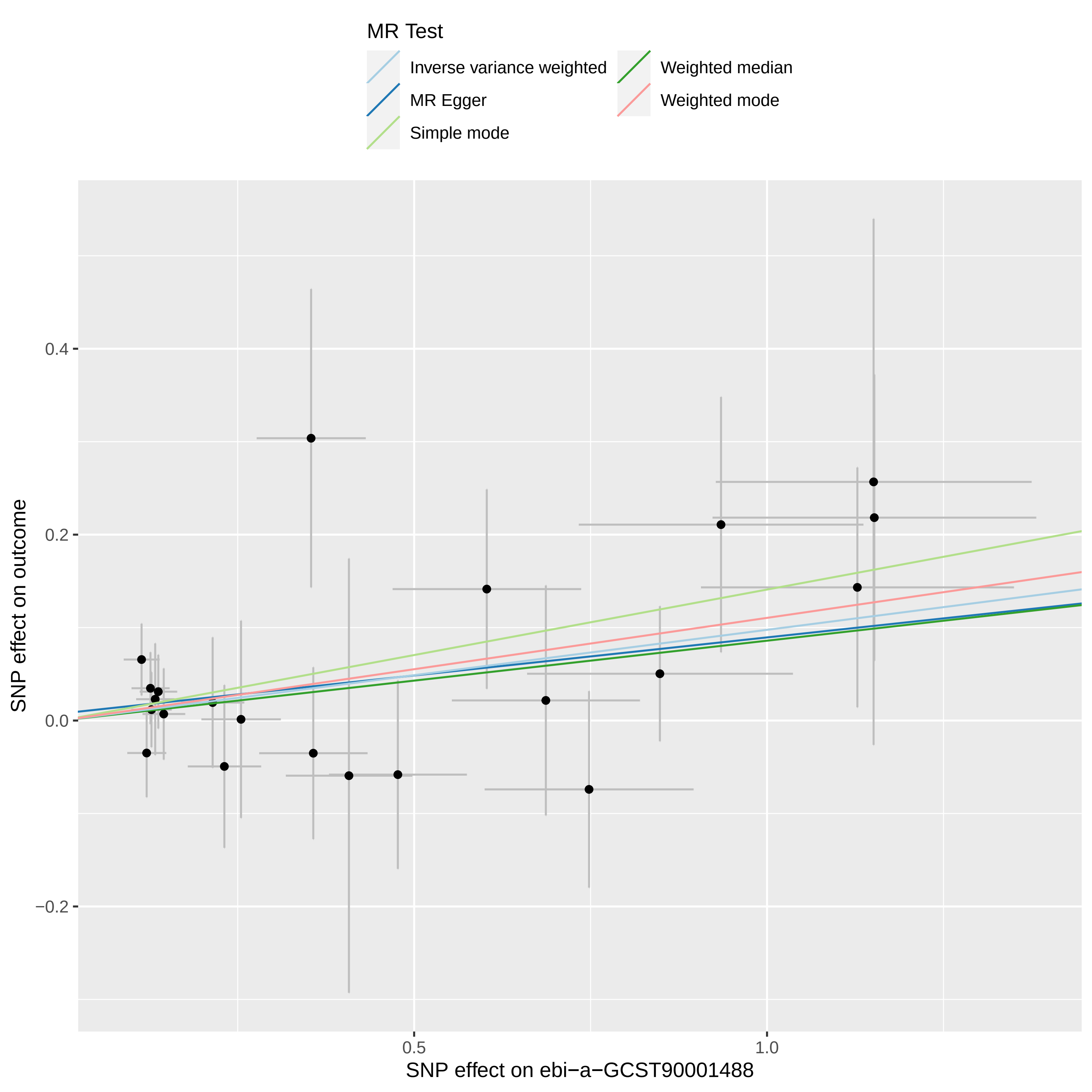

MR Test
Inverse variance weighted
MR Egger
Simple mode
Weighted median
Weighted mode
0.4
0.2
0.0
−0.2
SNP effect on outcome
0.5
1.0
SNP effect on ebi−a−GCST90001488

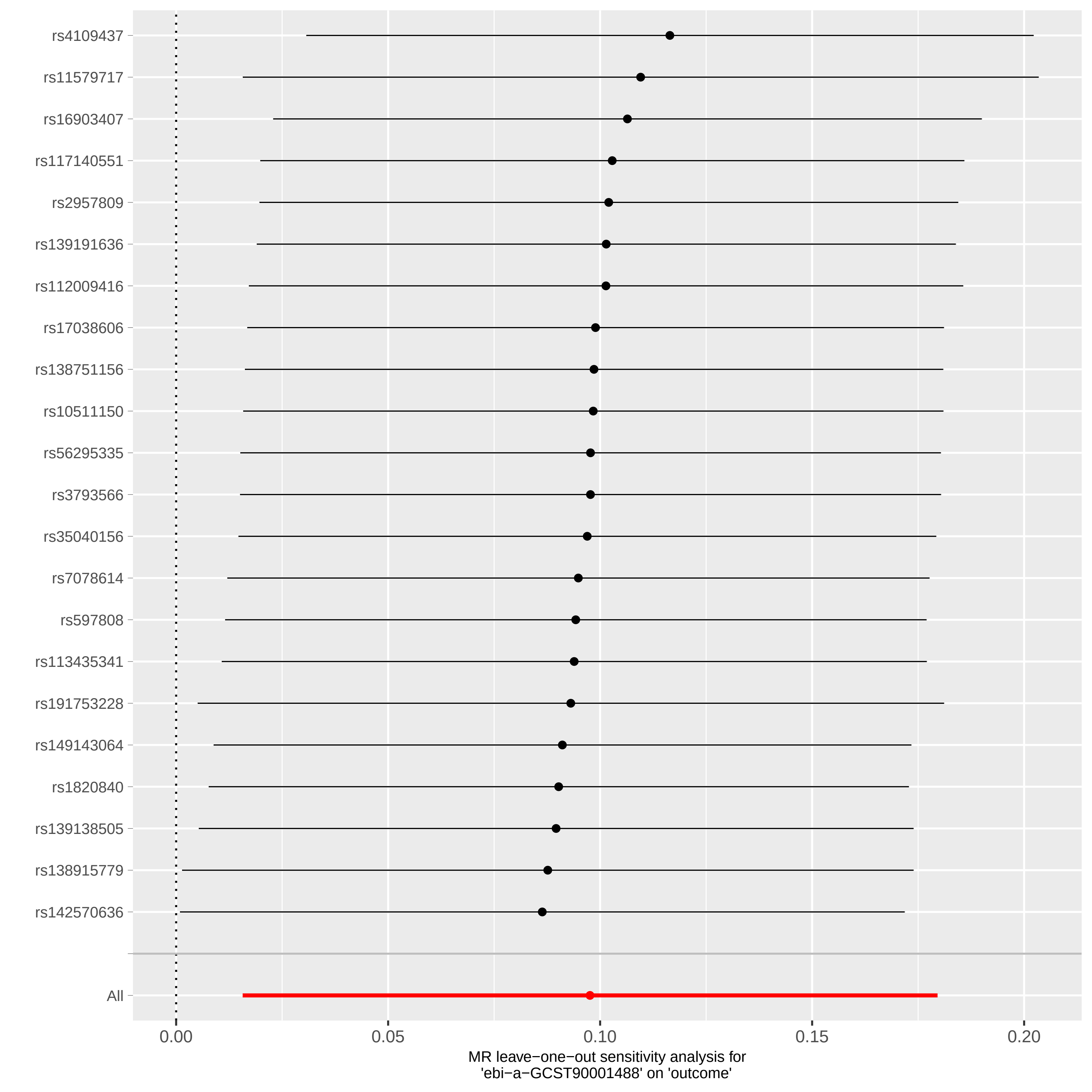
rs4109437
rs11579717
rs16903407
rs117140551
rs2957809
rs139191636
rs112009416
rs17038606
rs138751156
rs10511150
rs56295335
rs3793566
rs35040156
rs7078614
rs597808
rs113435341
rs191753228
rs149143064
rs1820840
rs139138505
rs138915779
rs142570636
All
0.00
0.05
0.10
0.15
0.20
MR leave−one−out sensitivity analysis for
'ebi−a−GCST90001488' on 'outcome'

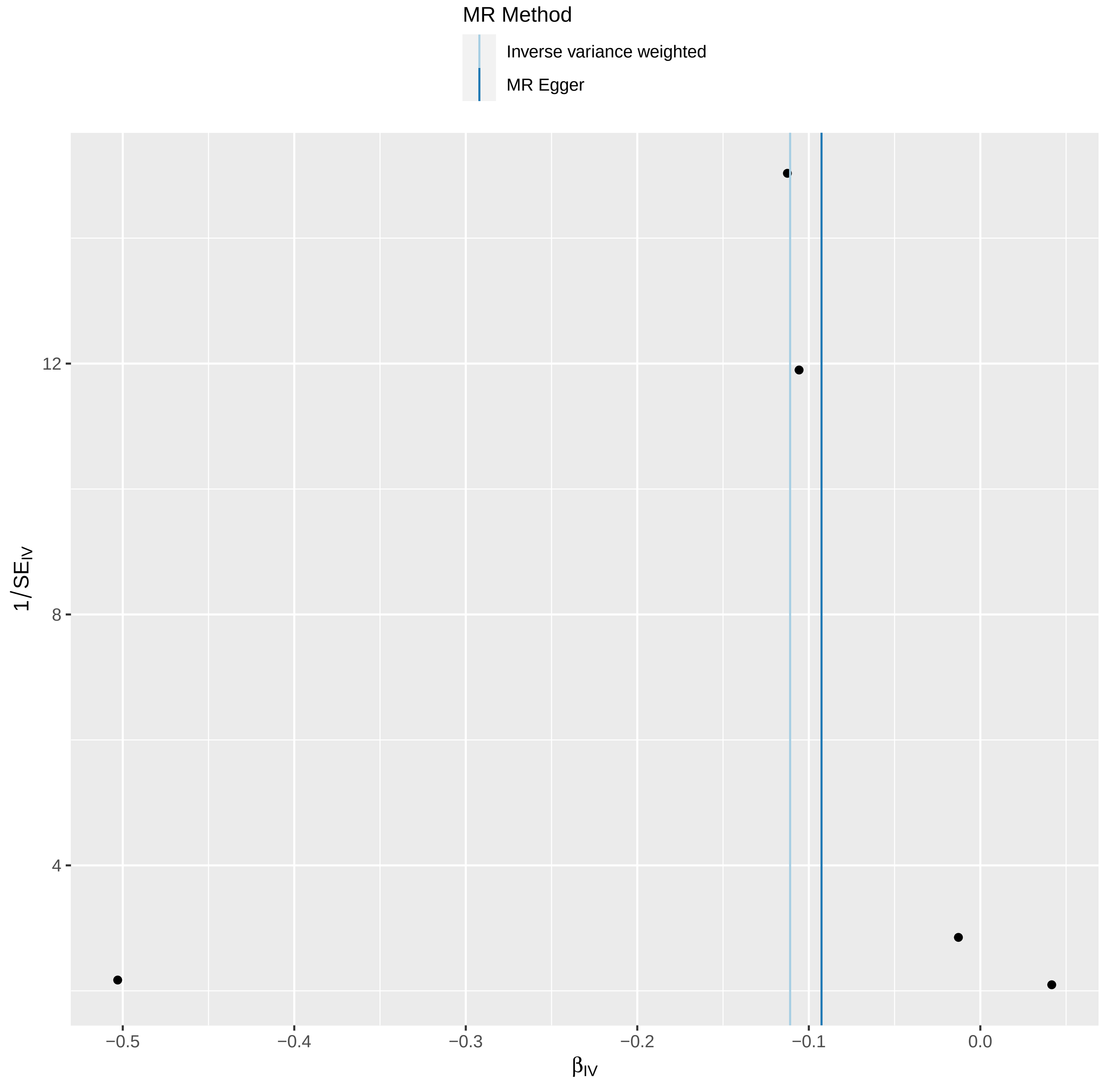
MR Method
Inverse variance weighted
MR Egger
12
8
4
1/SE$_{IV}$
−0.5
−0.4
−0.3
−0.2
−0.1
0.0
β$_{IV}$

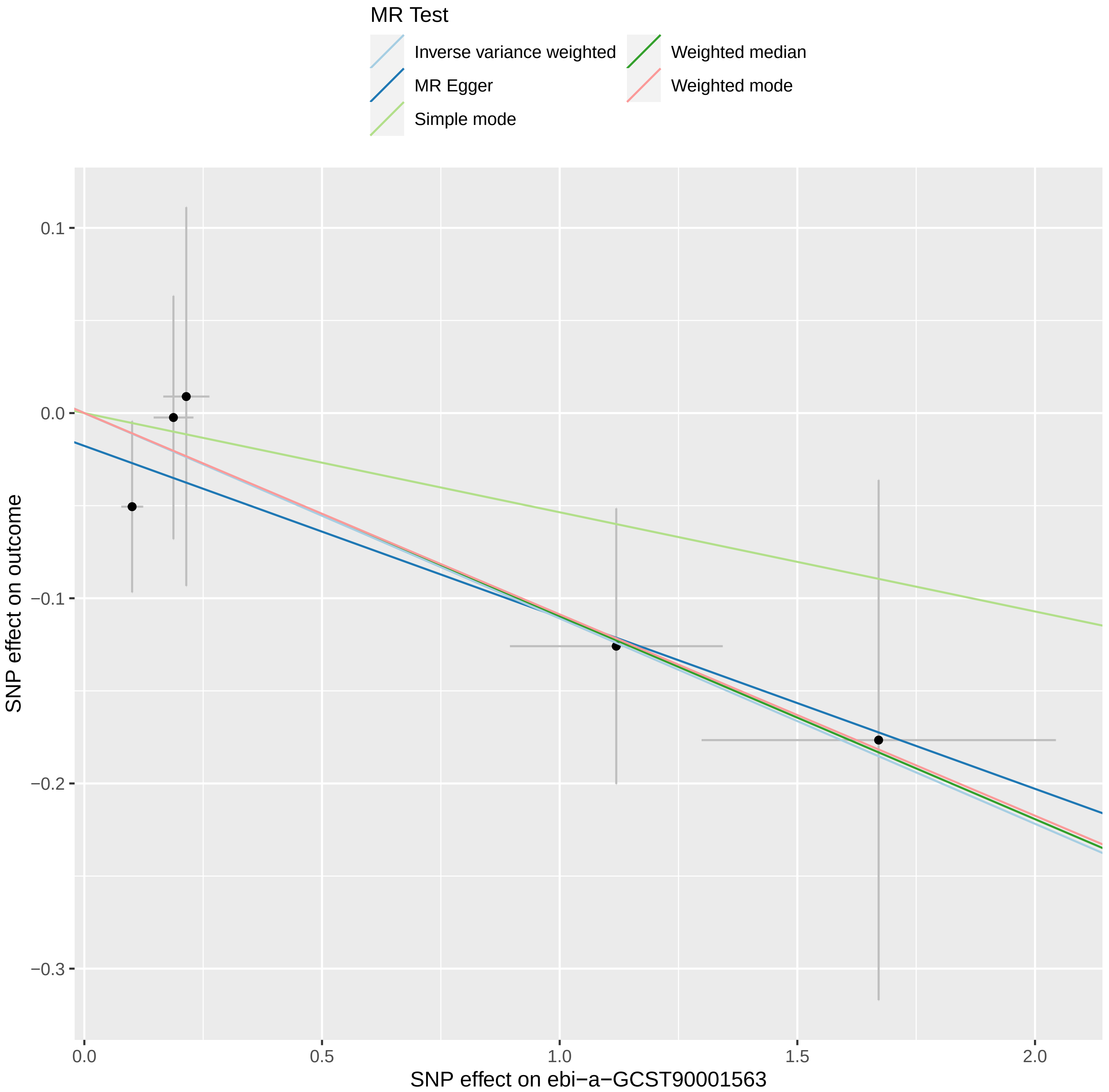
MR Test
Inverse variance weighted
MR Egger
Simple mode
Weighted median
Weighted mode
0.1
0.0
−0.1
−0.2
−0.3
SNP effect on outcome
0.0
0.5
1.0
1.5
2.0
SNP effect on ebi−a−GCST90001563

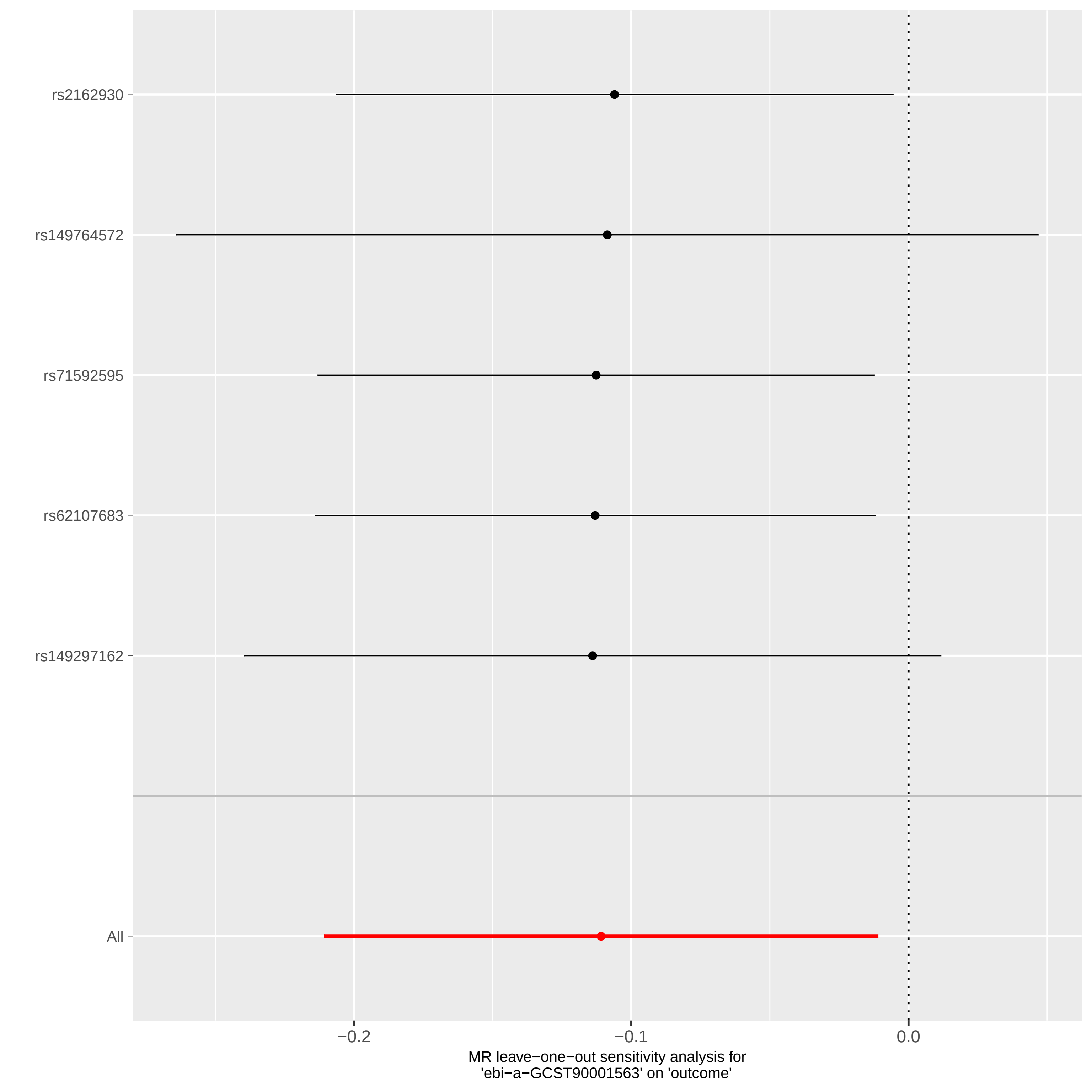
rs2162930
rs149764572
rs71592595
rs62107683
rs149297162
All
−0.2
−0.1
0.0
MR leave−one−out sensitivity analysis for
'ebi−a−GCST90001563' on 'outcome'

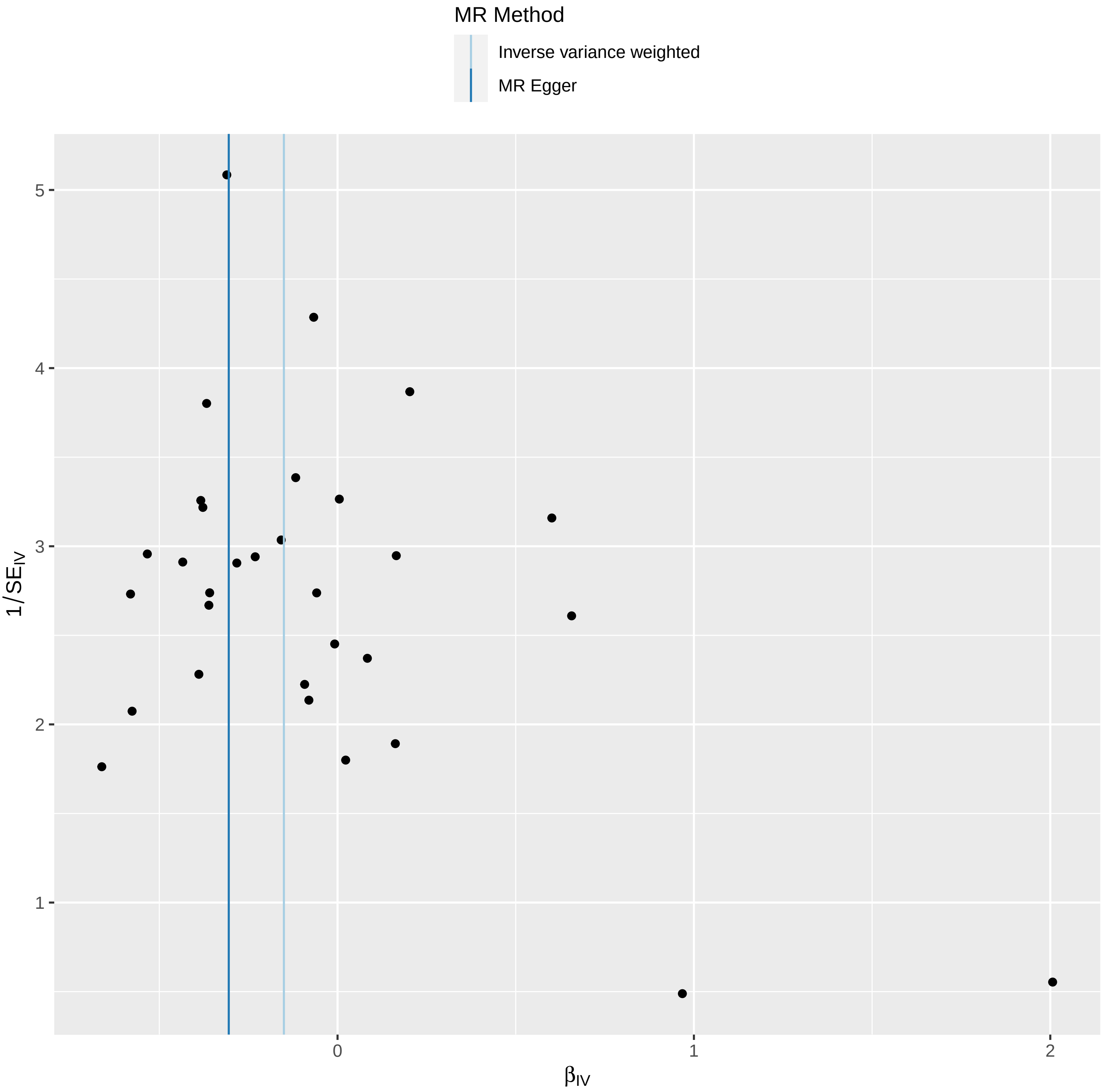
MR Method
Inverse variance weighted
MR Egger
1/SE_IV
5
4
3
2
1
0
1
2
β_IV

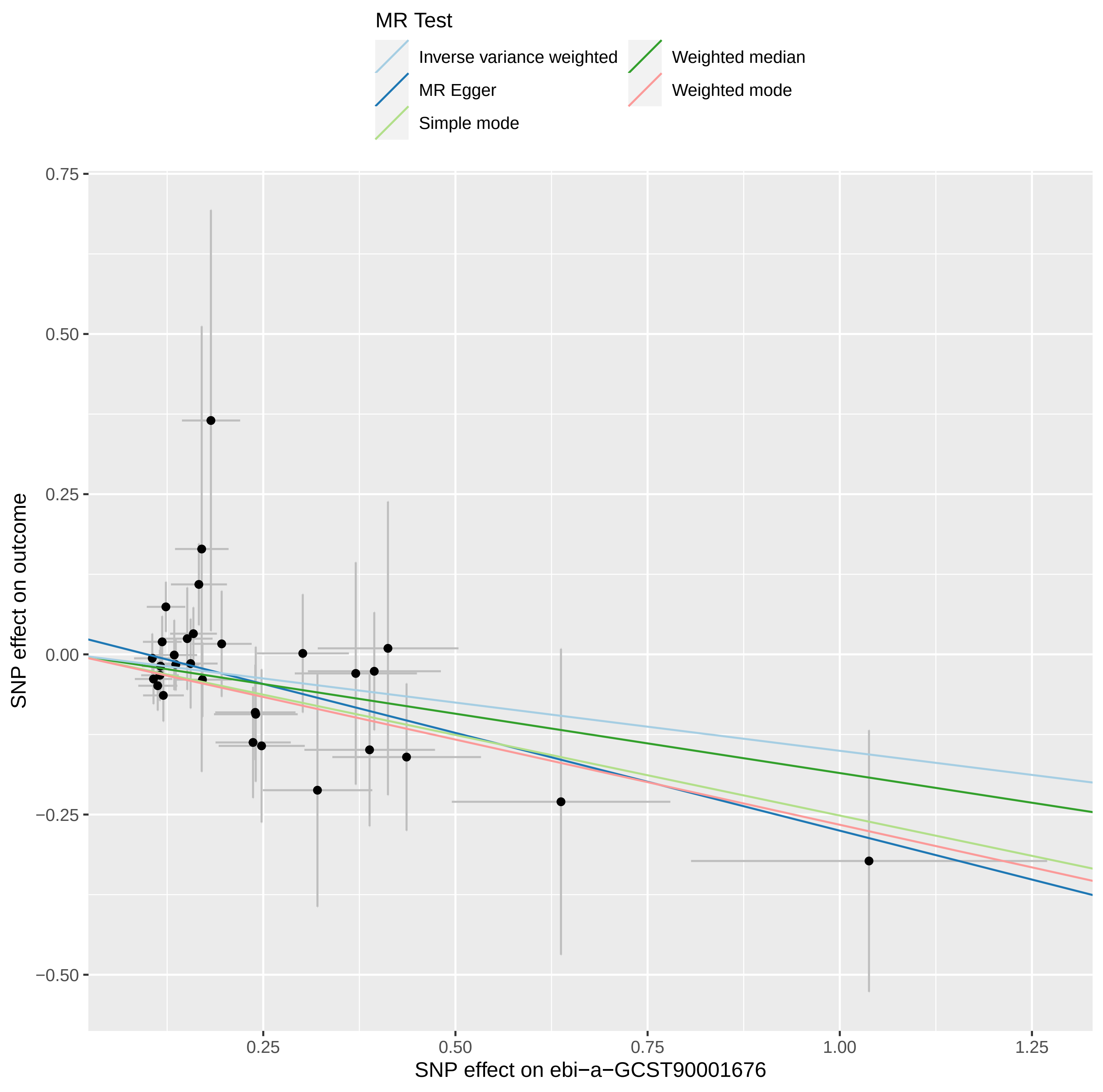

MR Test
Inverse variance weighted
MR Egger
Simple mode
Weighted median
Weighted mode
0.75
0.50
0.25
0.00
−0.25
−0.50
SNP effect on outcome
0.25
0.50
0.75
1.00
1.25
SNP effect on ebi−a−GCST90001676

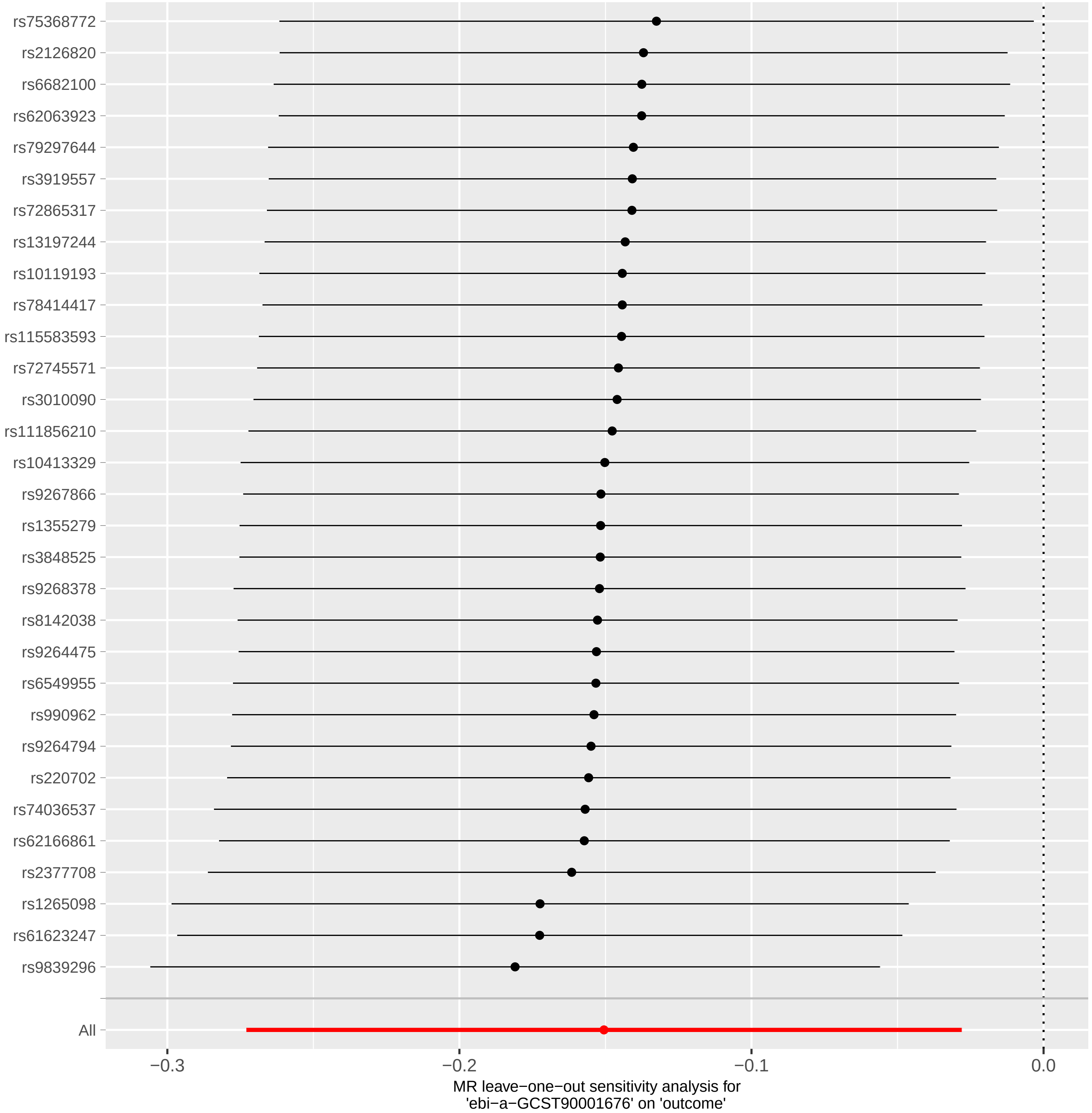
rs75368772
rs2126820
rs6682100
rs62063923
rs79297644
rs3919557
rs72865317
rs13197244
rs10119193
rs78414417
rs115583593
rs72745571
rs3010090
rs111856210
rs10413329
rs9267866
rs1355279
rs3848525
rs9268378
rs8142038
rs9264475
rs6549955
rs990962
rs9264794
rs220702
rs74036537
rs62166861
rs2377708
rs1265098
rs61623247
rs9839296
All
−0.3
−0.2
−0.1
0.0
MR leave−one−out sensitivity analysis for
'ebi−a−GCST90001676' on 'outcome'

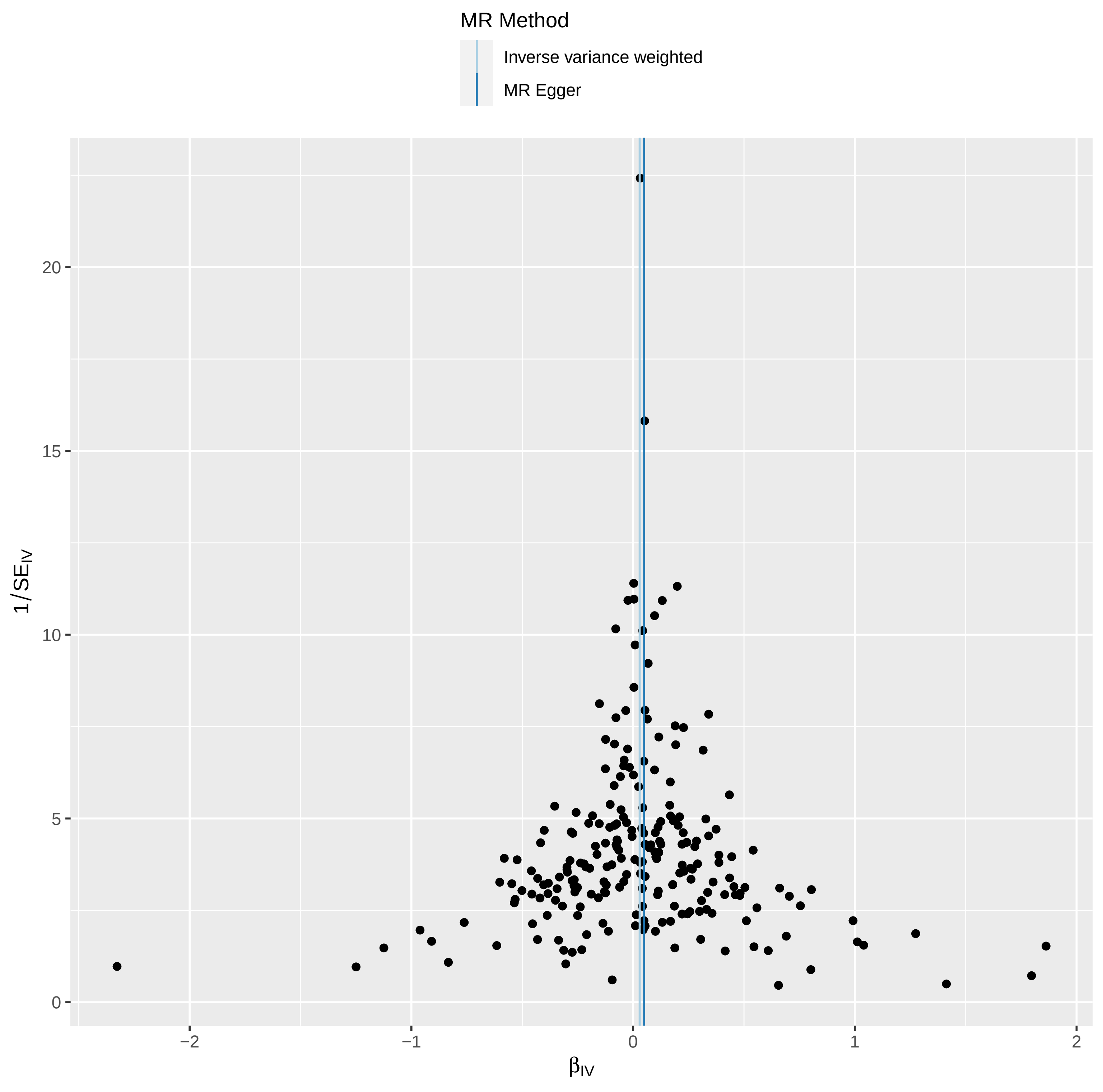
MR Method
Inverse variance weighted
MR Egger
$1/SE_{IV}$
0
5
10
15
20
β_{IV}
−2
−1
0
1
2

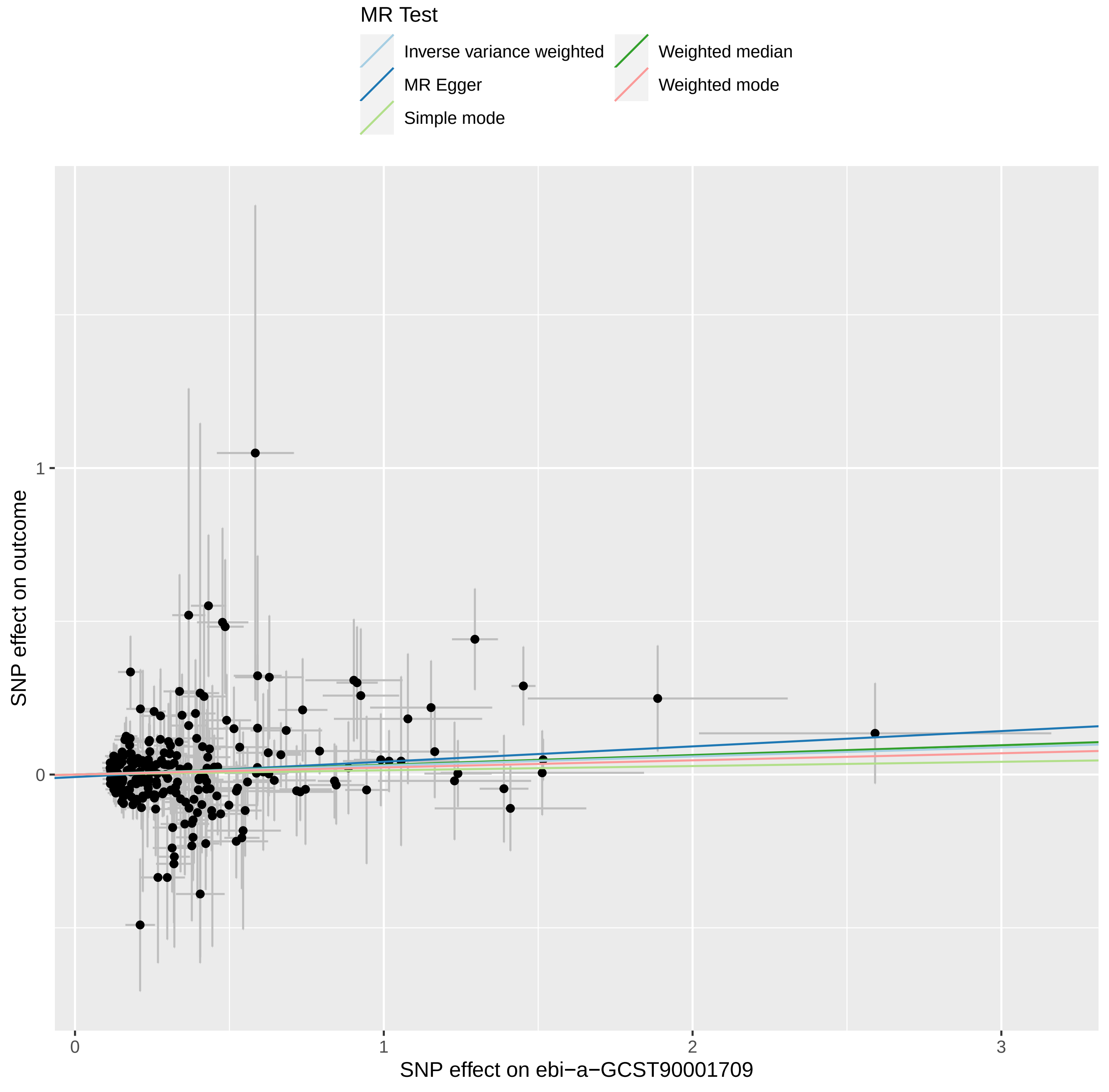
MR Test
Inverse variance weighted
MR Egger
Simple mode
Weighted median
Weighted mode
1
0
0
1
2
3
SNP effect on outcome
SNP effect on ebi−a−GCST90001709

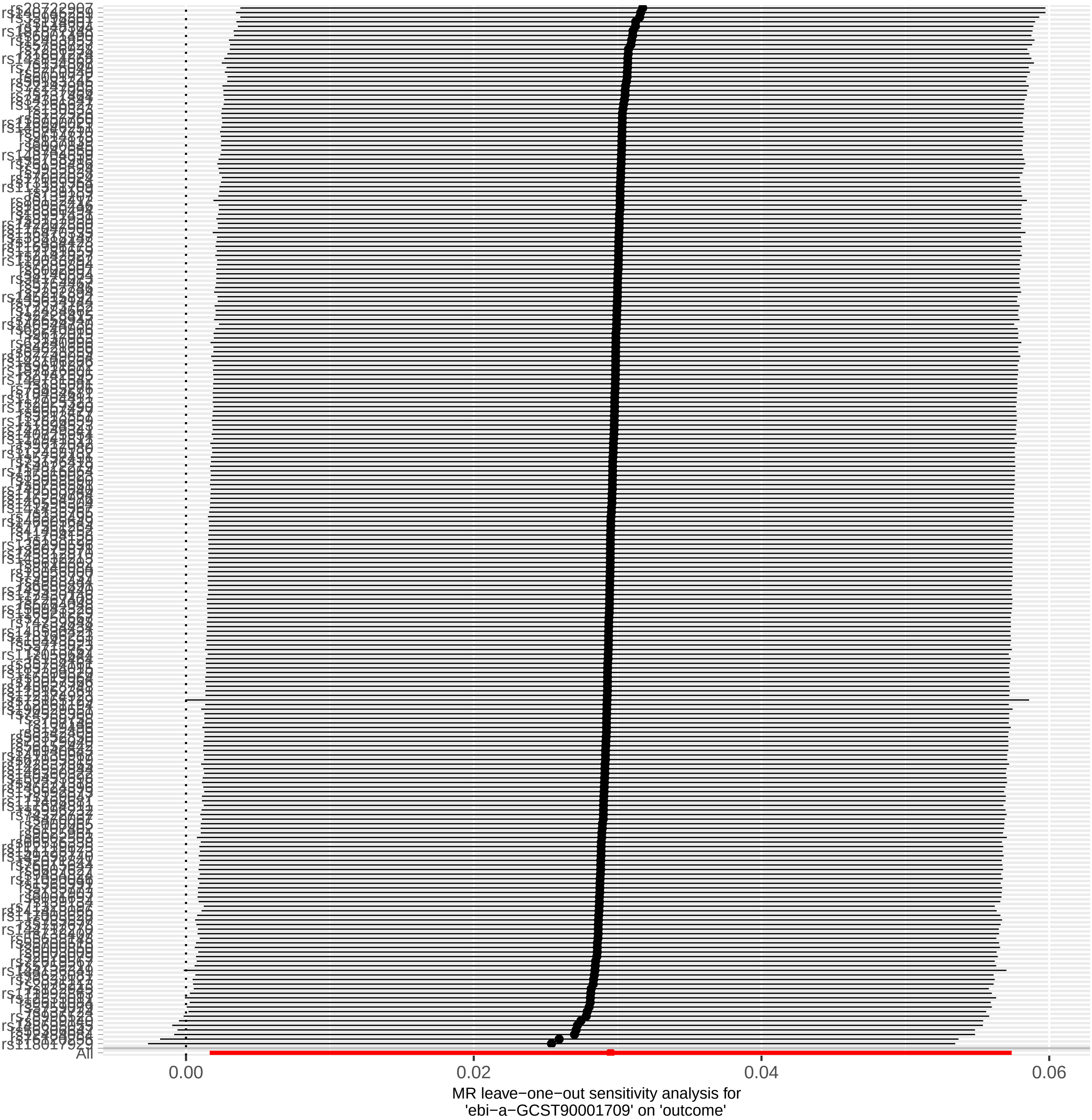
All
0.00
0.02
0.04
0.06
MR leave−one−out sensitivity analysis for
'ebi−a−GCST90001709' on 'outcome'

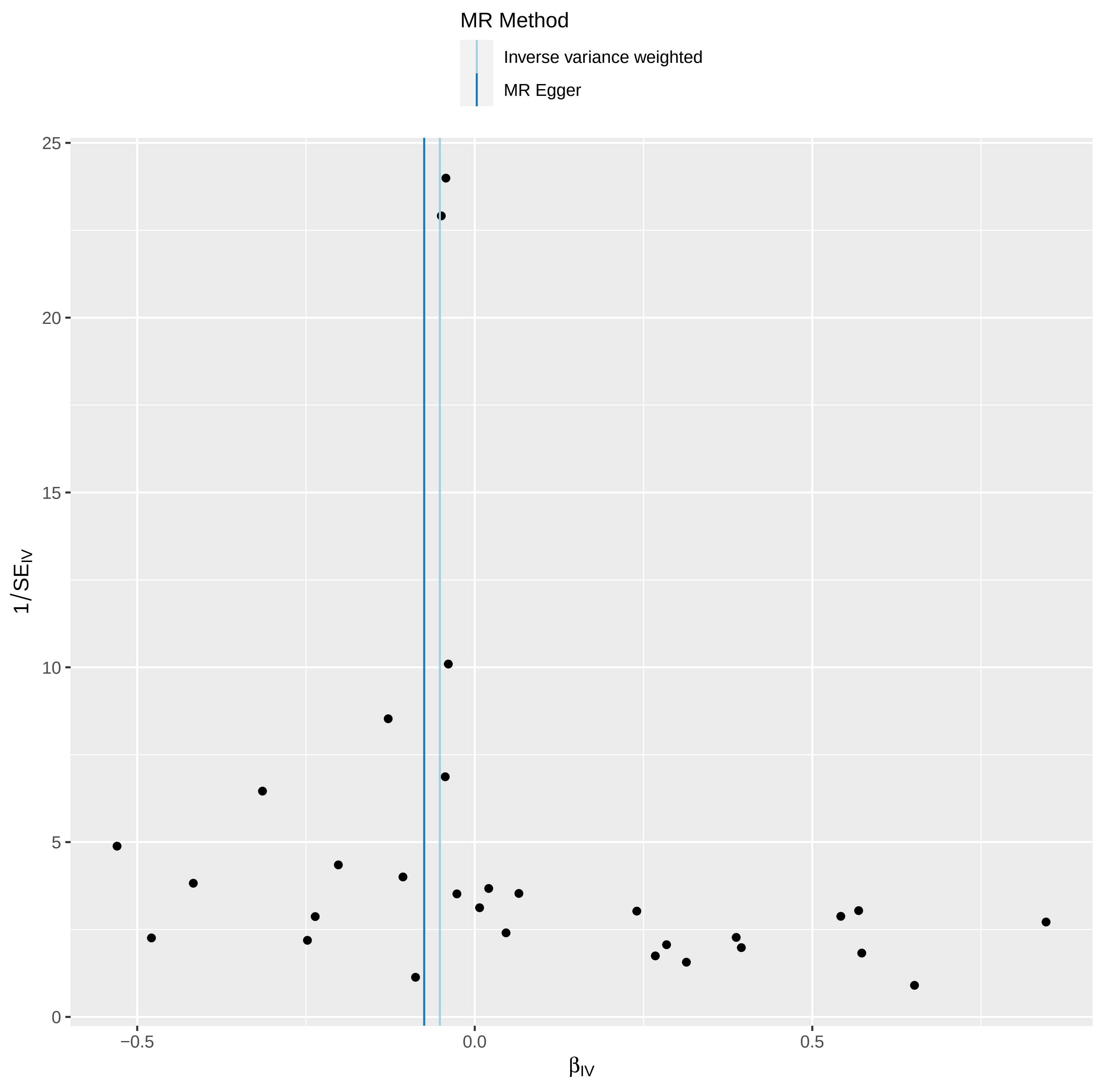
MR Method
Inverse variance weighted
MR Egger
25
20
15
10
5
0
1/SE_IV
−0.5
0.0
0.5
β_IV

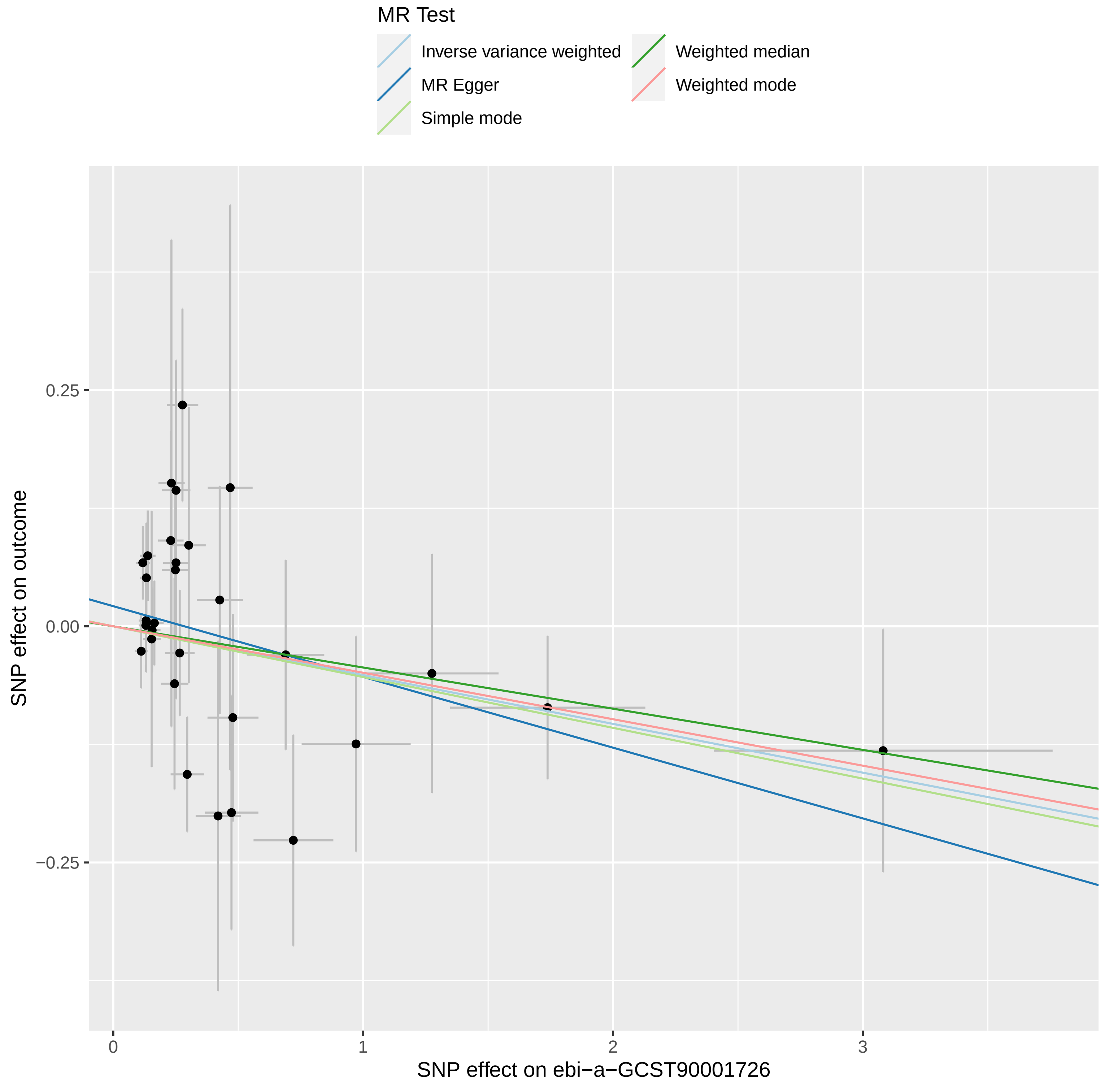

MR Test
Inverse variance weighted
MR Egger
Simple mode
Weighted median
Weighted mode
0.25
0.00
−0.25
0
1
2
3
SNP effect on outcome
SNP effect on ebi−a−GCST90001726

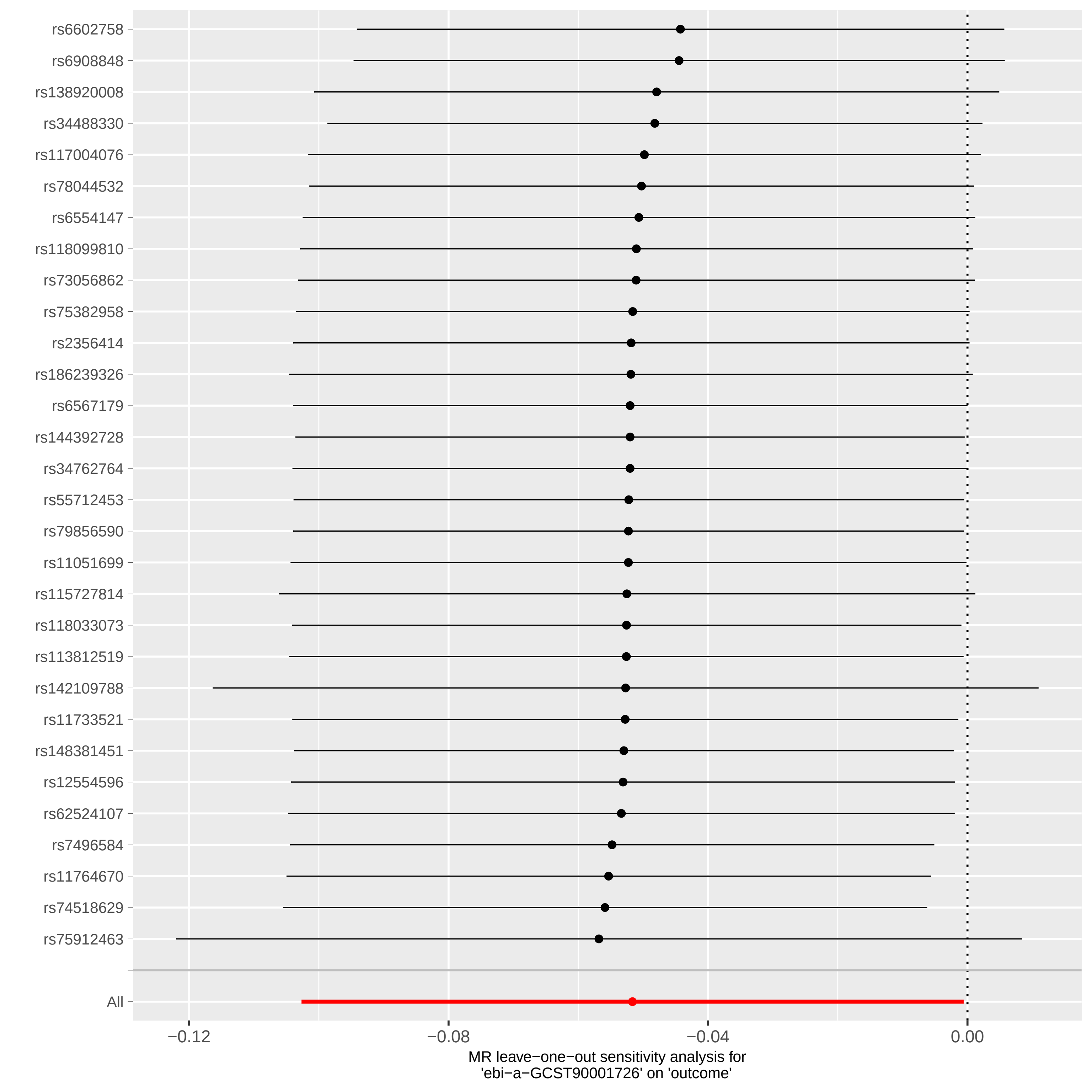
rs6602758
rs6908848
rs138920008
rs34488330
rs117004076
rs78044532
rs6554147
rs118099810
rs73056862
rs75382958
rs2356414
rs186239326
rs6567179
rs144392728
rs34762764
rs55712453
rs79856590
rs11051699
rs115727814
rs118033073
rs113812519
rs142109788
rs11733521
rs148381451
rs12554596
rs62524107
rs7496584
rs11764670
rs74518629
rs75912463
All
−0.12
−0.08
−0.04
0.00
MR leave−one−out sensitivity analysis for
'ebi−a−GCST90001726' on 'outcome'

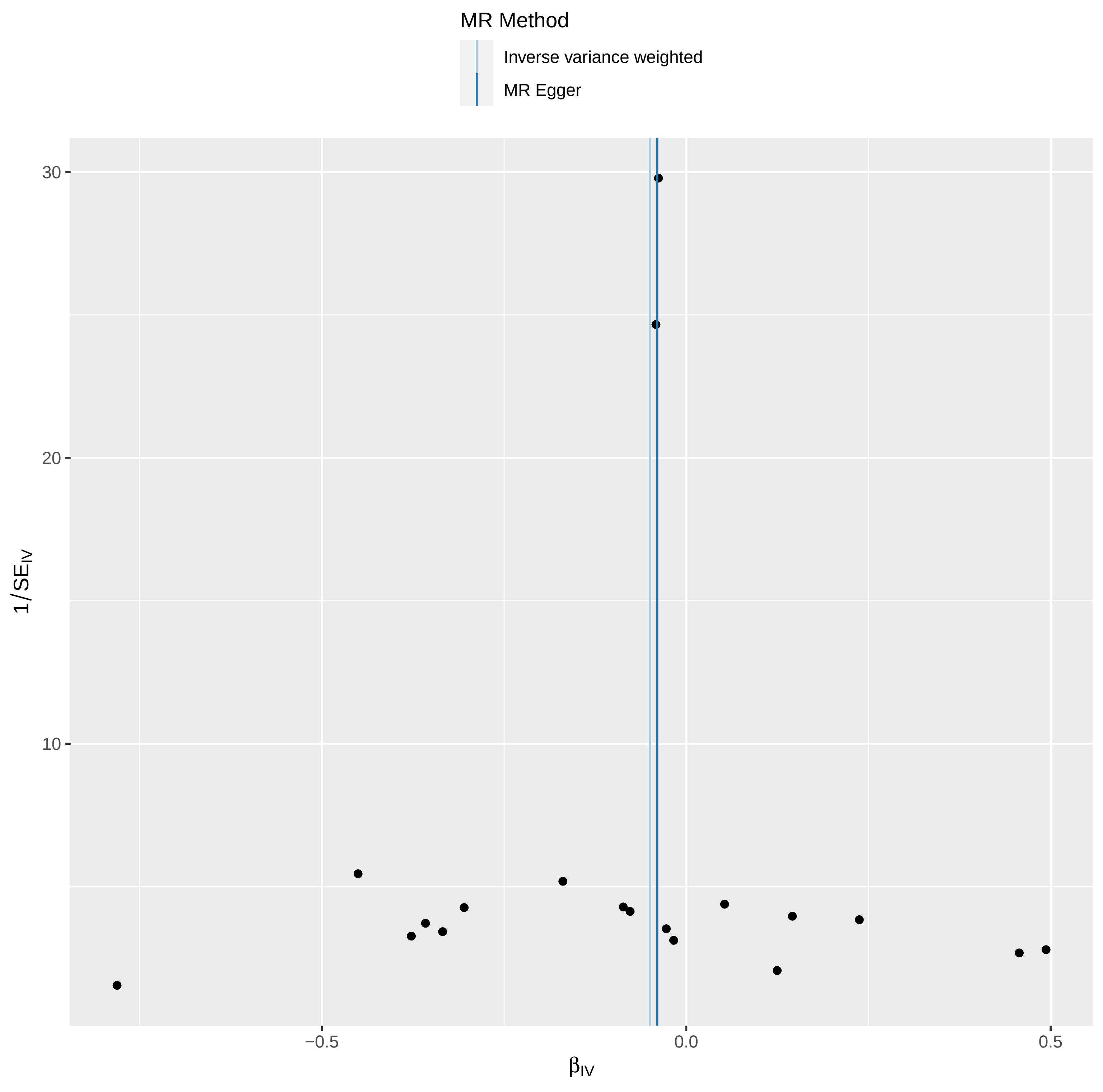
MR Method
Inverse variance weighted
MR Egger
30
20
10
1/SE_IV
−0.5
0.0
0.5
β_IV

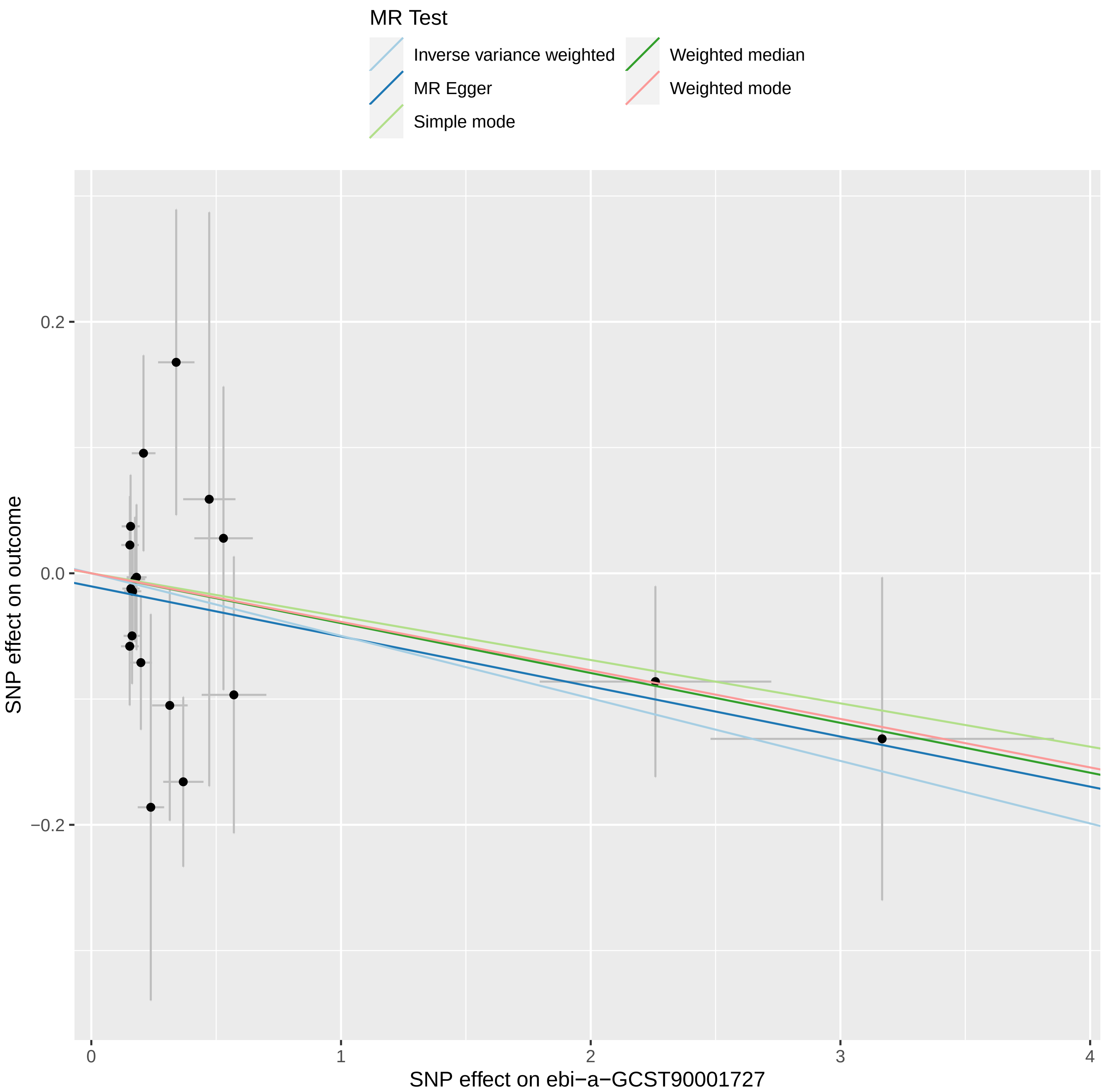
MR Test
Inverse variance weighted
MR Egger
Simple mode
Weighted median
Weighted mode
0.2
0.0
−0.2
SNP effect on outcome
0
1
2
3
4
SNP effect on ebi−a−GCST90001727

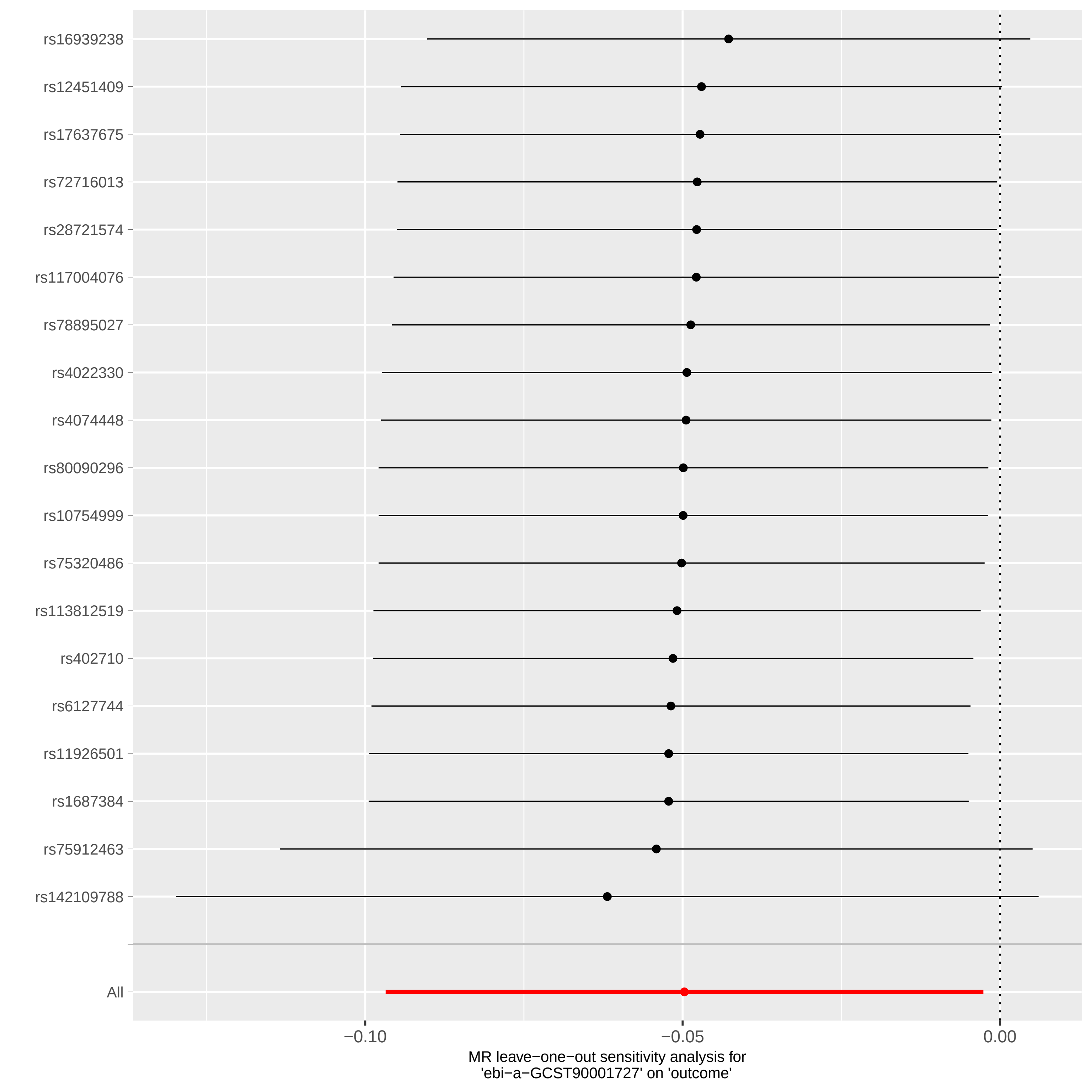
rs16939238
rs12451409
rs17637675
rs72716013
rs28721574
rs117004076
rs78895027
rs4022330
rs4074448
rs80090296
rs10754999
rs75320486
rs113812519
rs402710
rs6127744
rs11926501
rs1687384
rs75912463
rs142109788
All
−0.10
−0.05
0.00
MR leave−one−out sensitivity analysis for
'ebi−a−GCST90001727' on 'outcome'

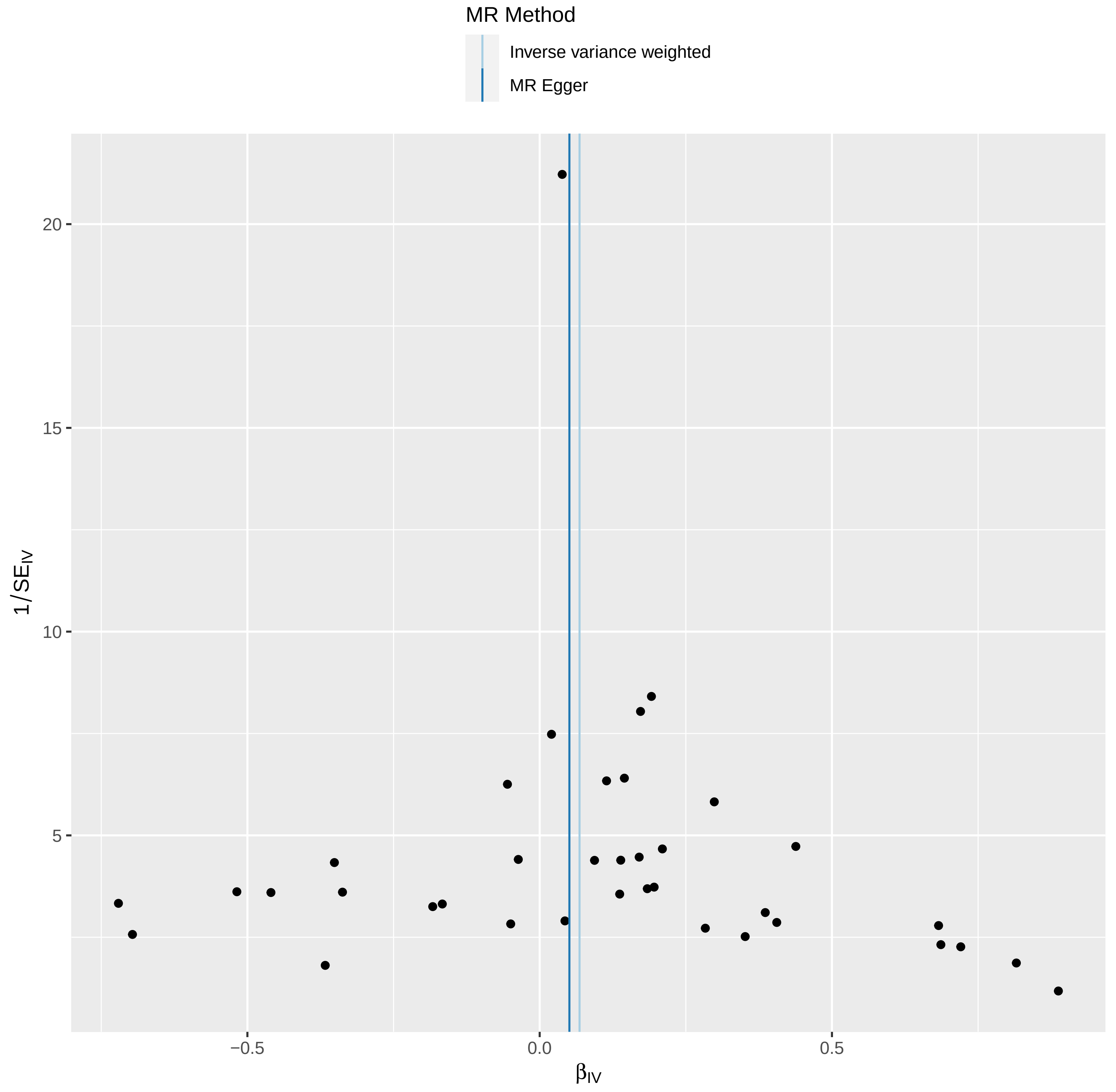

MR Method
Inverse variance weighted
MR Egger
20
15
10
5
1/SE_IV
−0.5
0.0
0.5
β_IV

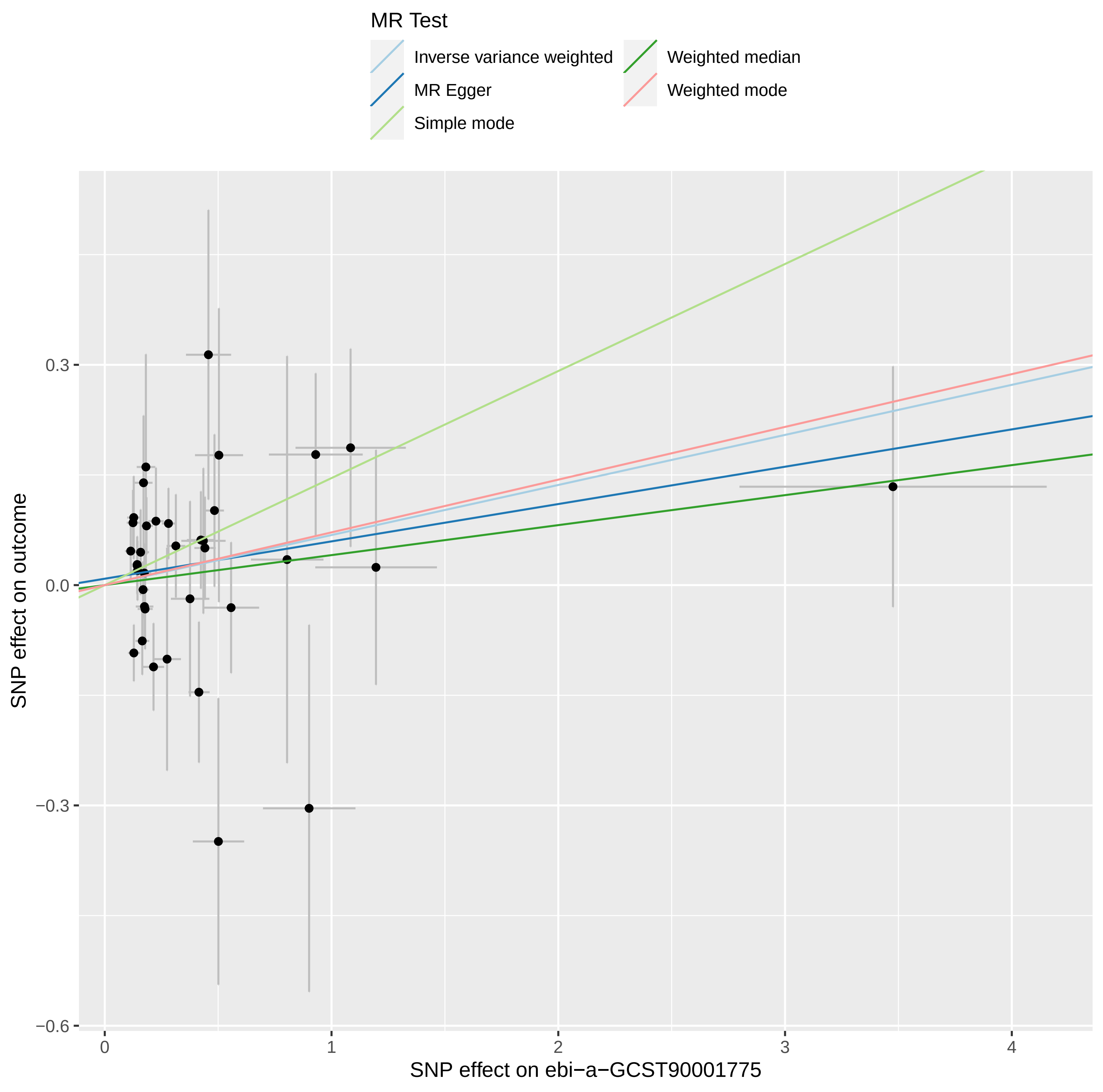

MR Test
Inverse variance weighted
MR Egger
Simple mode
Weighted median
Weighted mode
SNP effect on outcome
SNP effect on ebi−a−GCST90001775
0.3
0.0
−0.3
−0.6
0
1
2
3
4

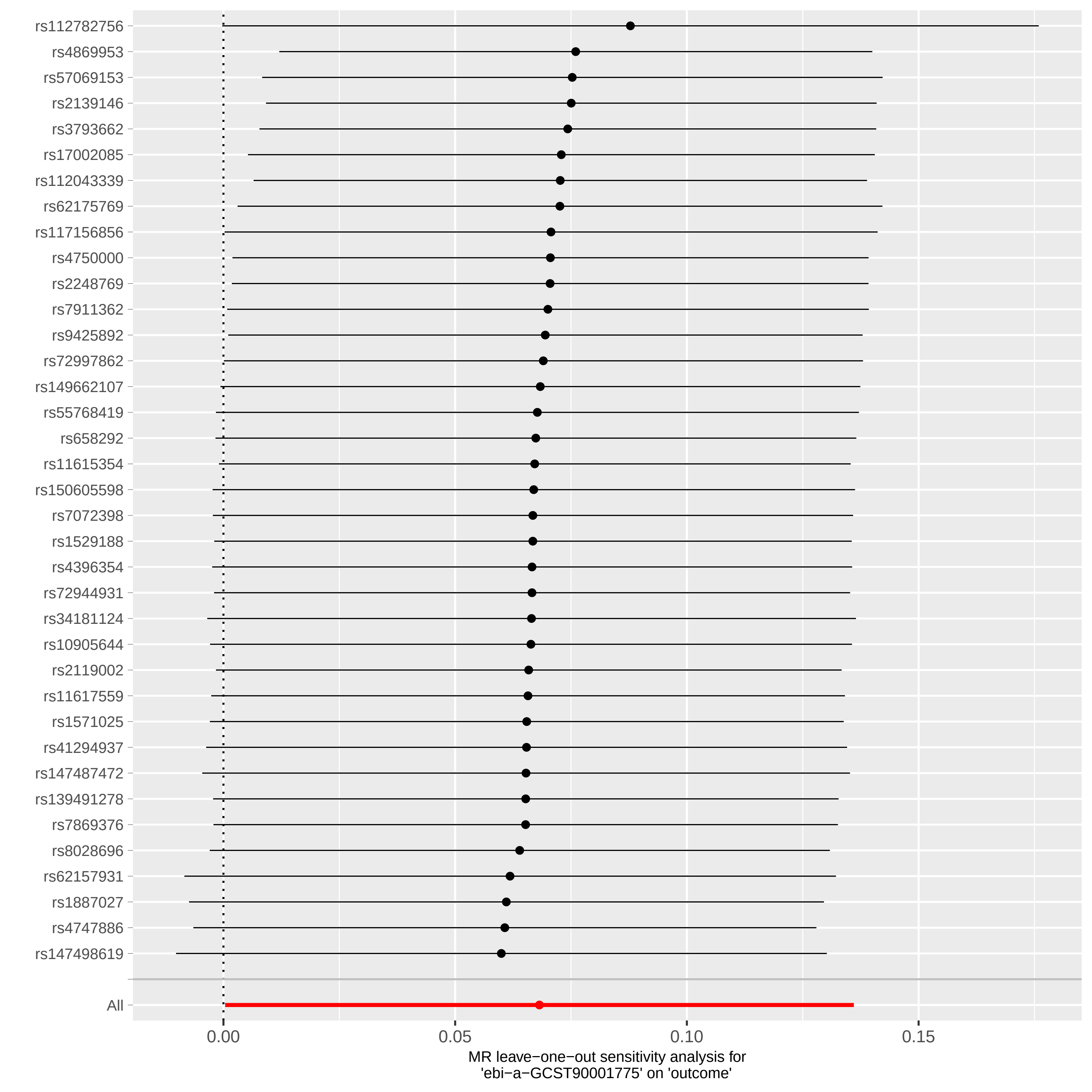
rs112782756
rs4869953
rs57069153
rs2139146
rs3793662
rs17002085
rs112043339
rs62175769
rs117156856
rs4750000
rs2248769
rs7911362
rs9425892
rs72997862
rs149662107
rs55768419
rs658292
rs11615354
rs150605598
rs7072398
rs1529188
rs4396354
rs72944931
rs34181124
rs10905644
rs2119002
rs11617559
rs1571025
rs41294937
rs147487472
rs139491278
rs7869376
rs8028696
rs62157931
rs1887027
rs4747886
rs147498619
All
0.00
0.05
0.10
0.15
MR leave−one−out sensitivity analysis for
'ebi−a−GCST90001775' on 'outcome'

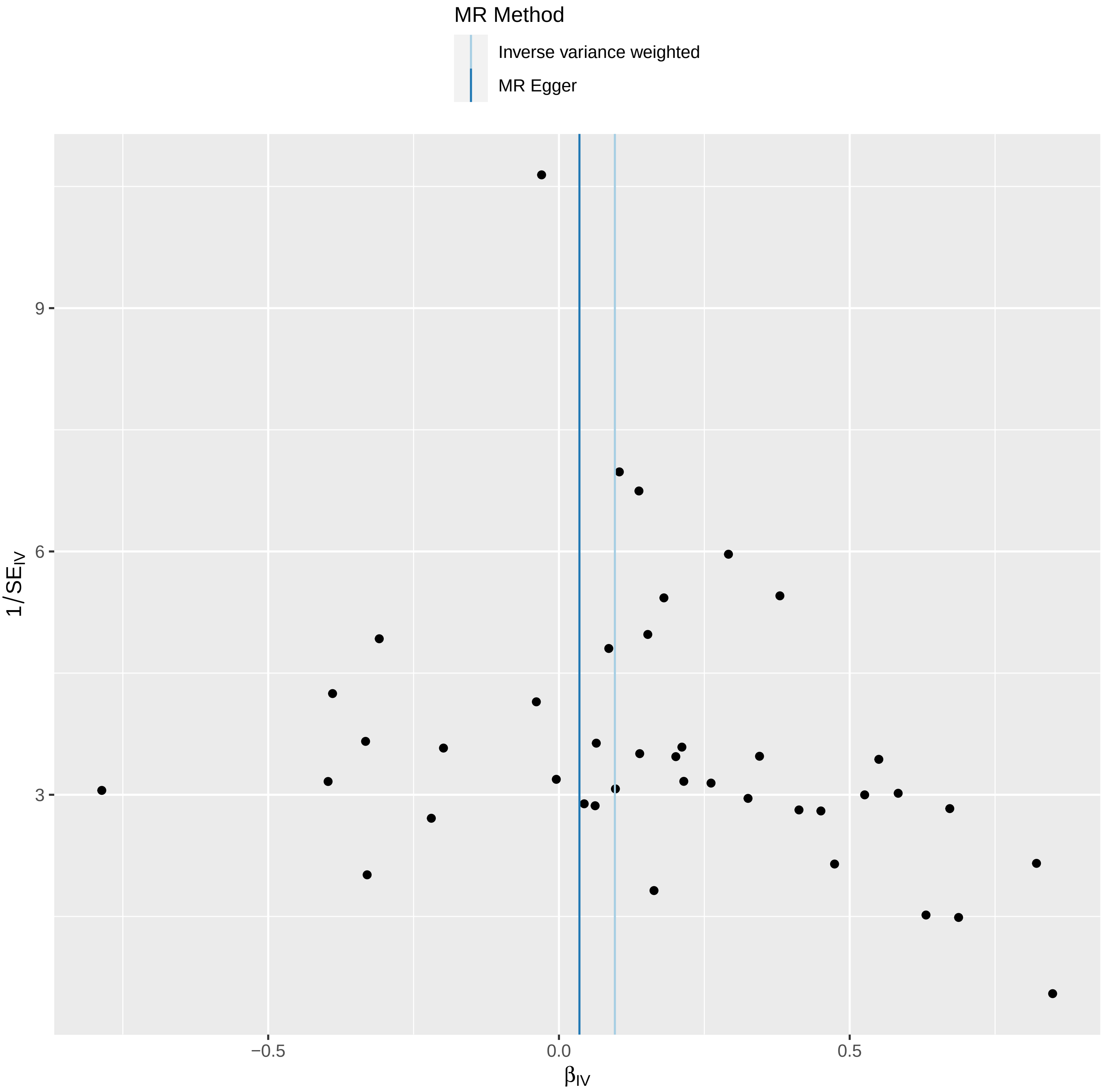

MR Method
Inverse variance weighted
MR Egger
9
6
3
−0.5
0.0
0.5
β_{IV}
$1/SE_{IV}$

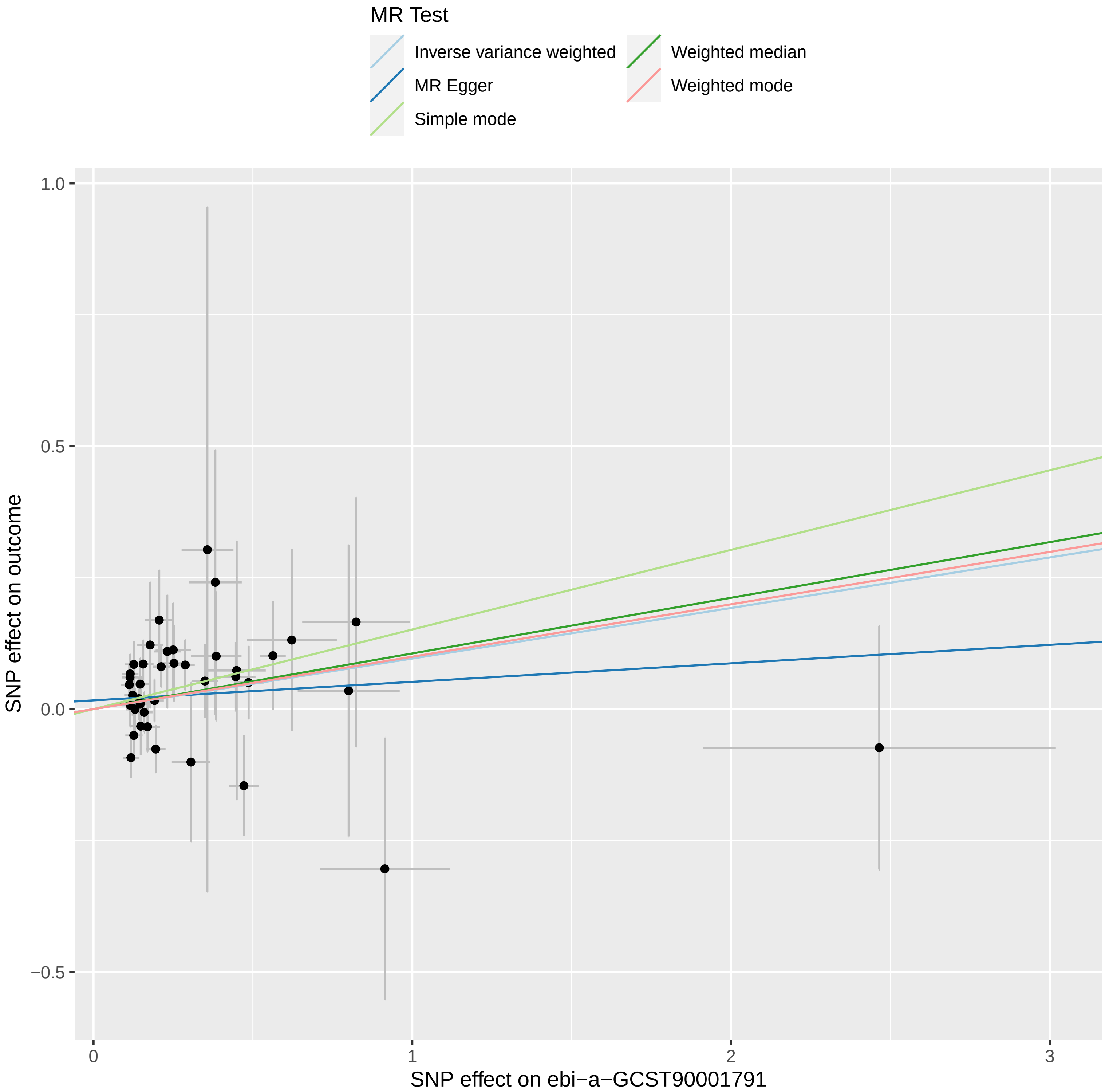

MR Test
Inverse variance weighted
MR Egger
Simple mode
Weighted median
Weighted mode
1.0
0.5
0.0
−0.5
SNP effect on outcome
0
1
2
3
SNP effect on ebi−a−GCST90001791

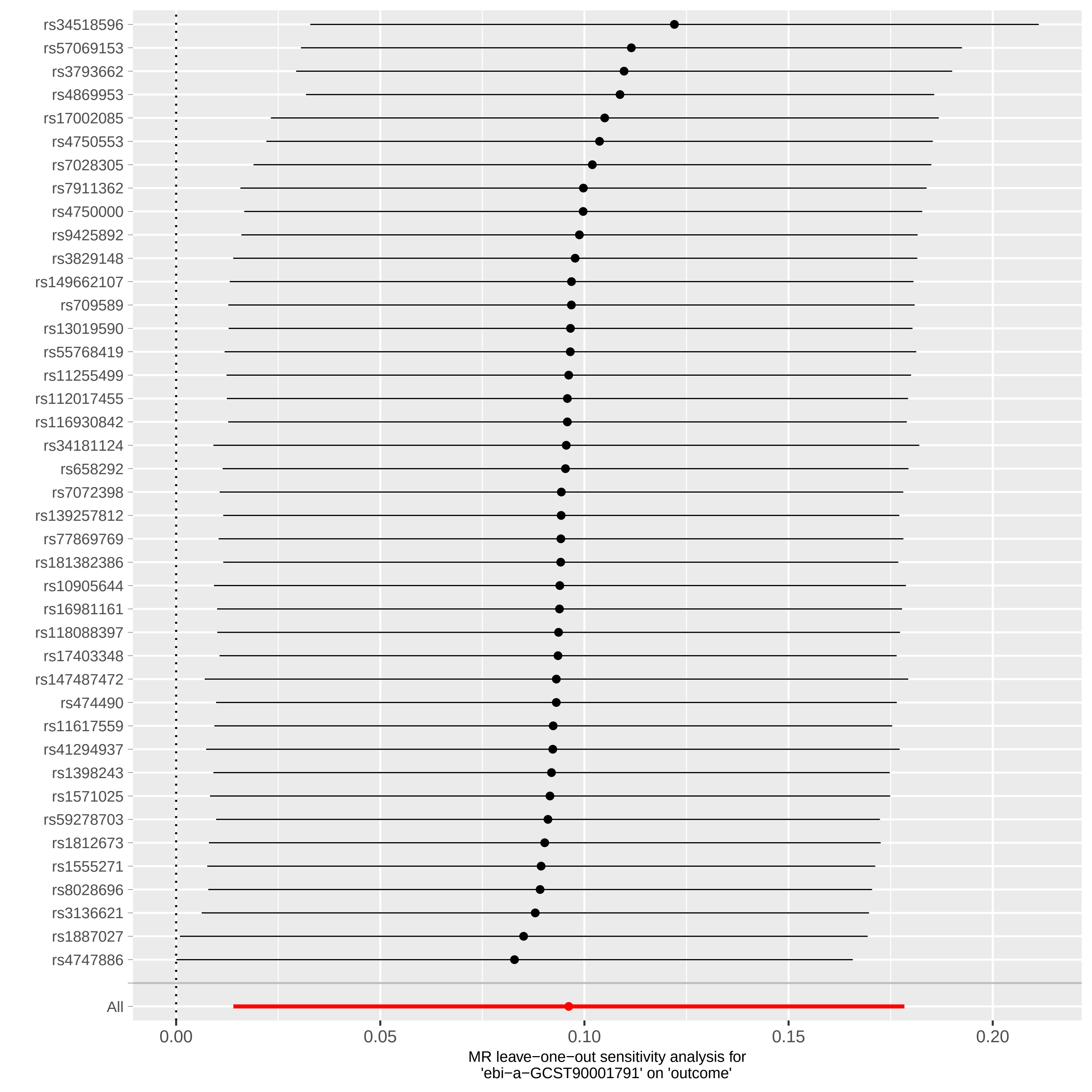
rs34518596
rs57069153
rs3793662
rs4869953
rs17002085
rs4750553
rs7028305
rs7911362
rs4750000
rs9425892
rs3829148
rs149662107
rs709589
rs13019590
rs55768419
rs11255499
rs112017455
rs116930842
rs34181124
rs658292
rs7072398
rs139257812
rs77869769
rs181382386
rs10905644
rs16981161
rs118088397
rs17403348
rs147487472
rs474490
rs11617559
rs41294937
rs1398243
rs1571025
rs59278703
rs1812673
rs1555271
rs8028696
rs3136621
rs1887027
rs4747886
All
0.00
0.05
0.10
0.15
0.20
MR leave−one−out sensitivity analysis for
'ebi−a−GCST90001791' on 'outcome'

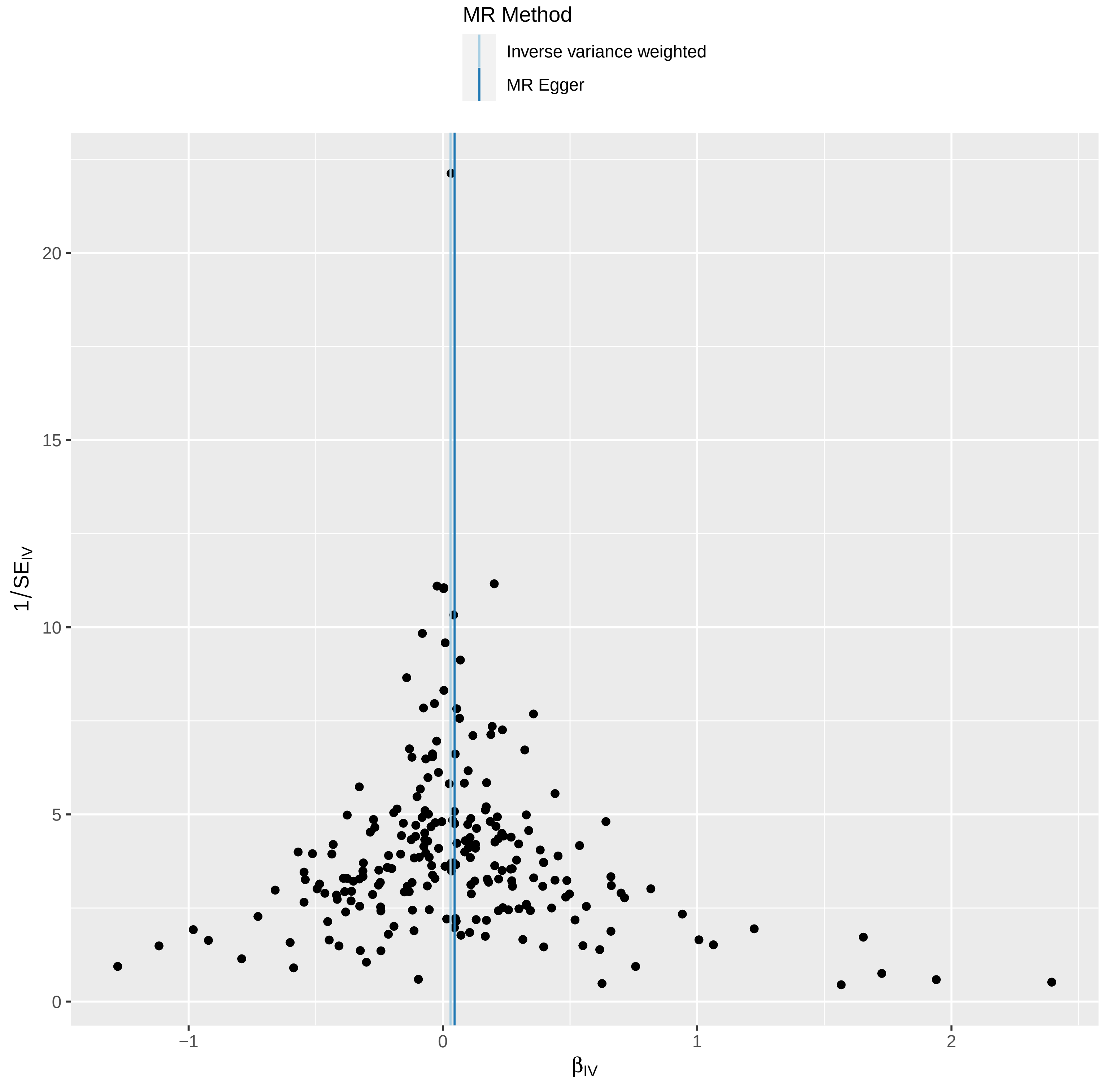
MR Method
Inverse variance weighted
MR Egger
1/SE$_{IV}$
β$_{IV}$
0
5
10
15
20
−1
0
1
2

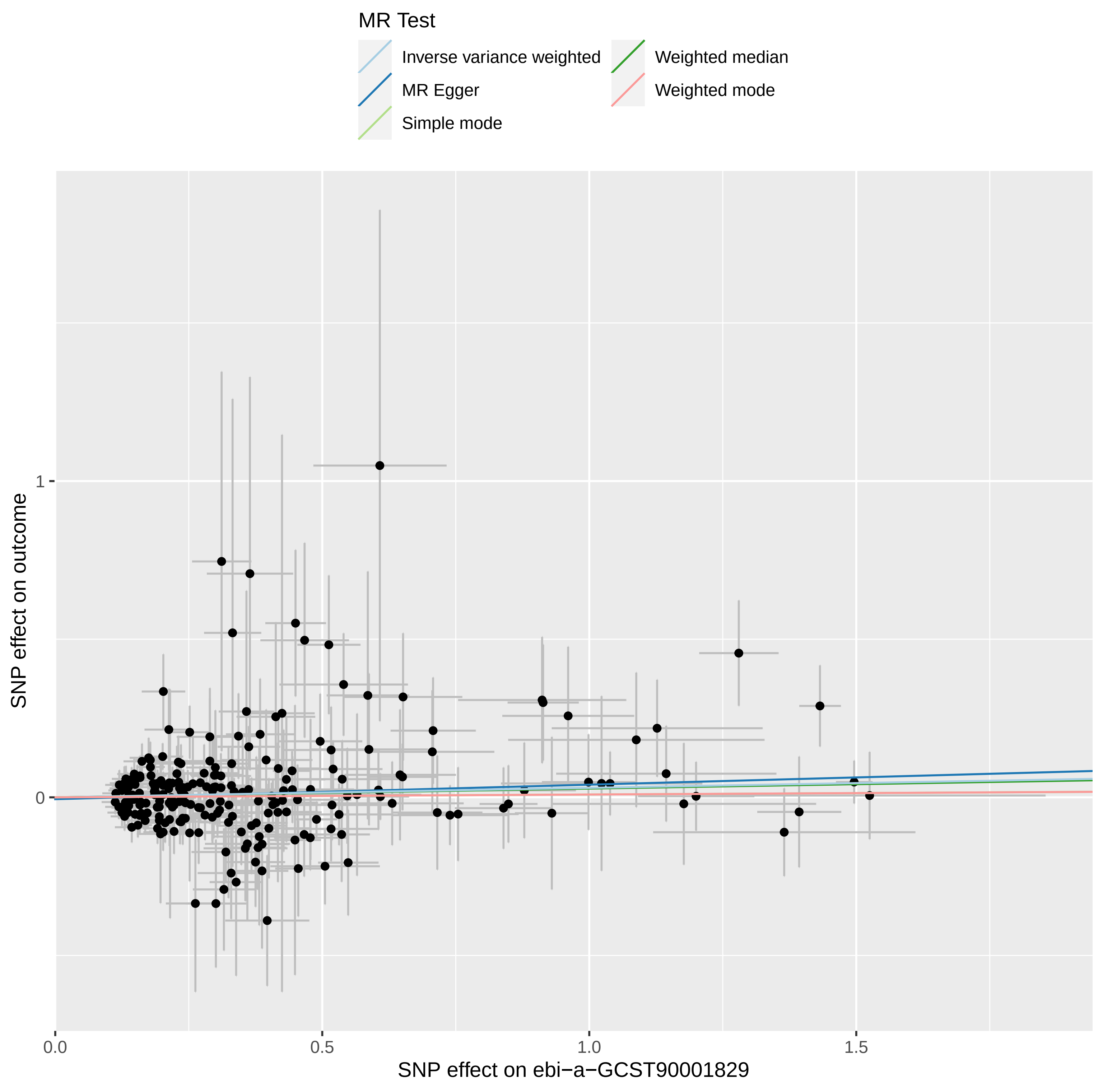
MR Test
Inverse variance weighted
MR Egger
Simple mode
Weighted median
Weighted mode
SNP effect on outcome
1
0
0.0
0.5
1.0
1.5
SNP effect on ebi−a−GCST90001829

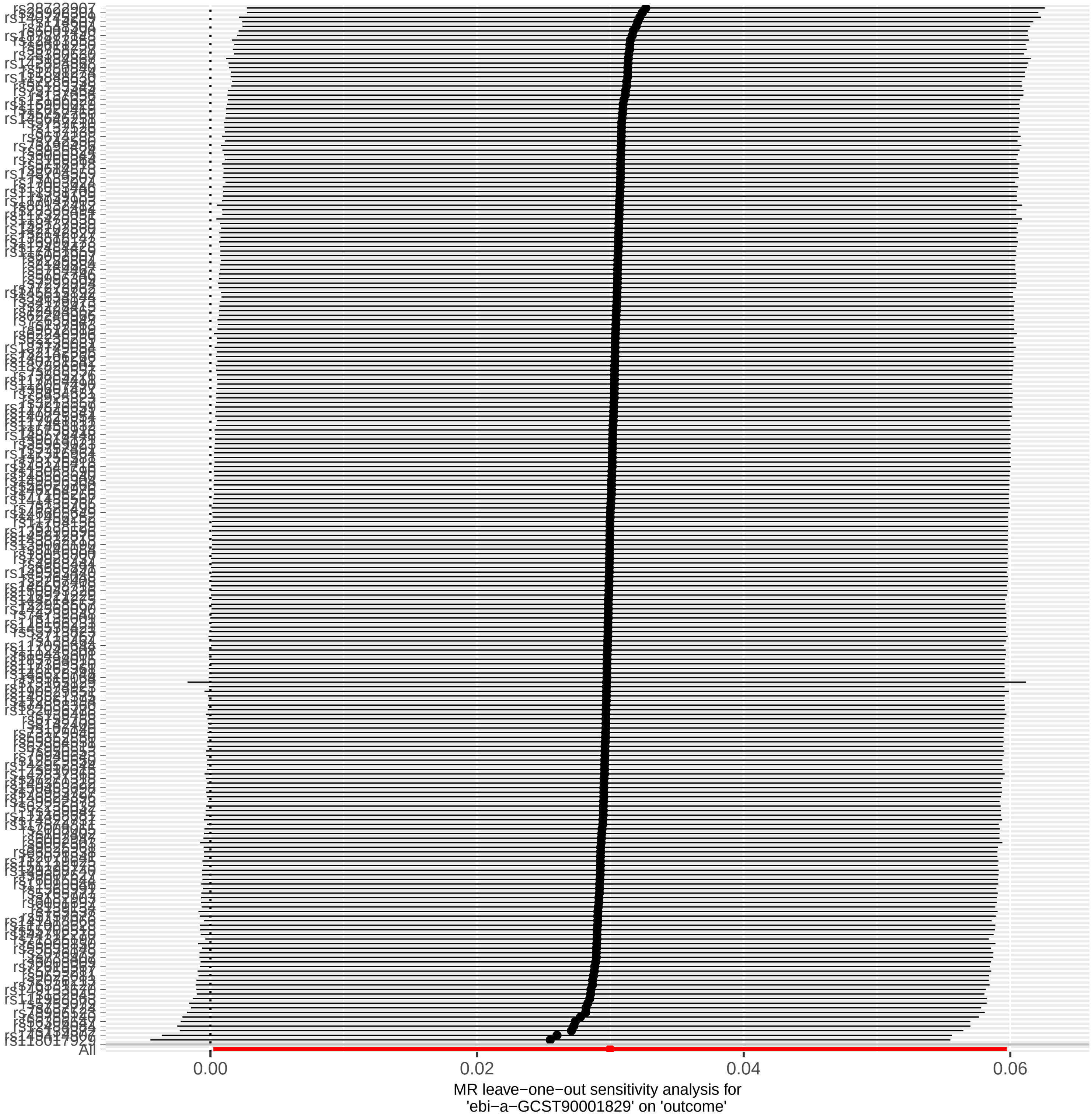
All
0.00
0.02
0.04
0.06
MR leave−one−out sensitivity analysis for
'ebi−a−GCST90001829' on 'outcome'

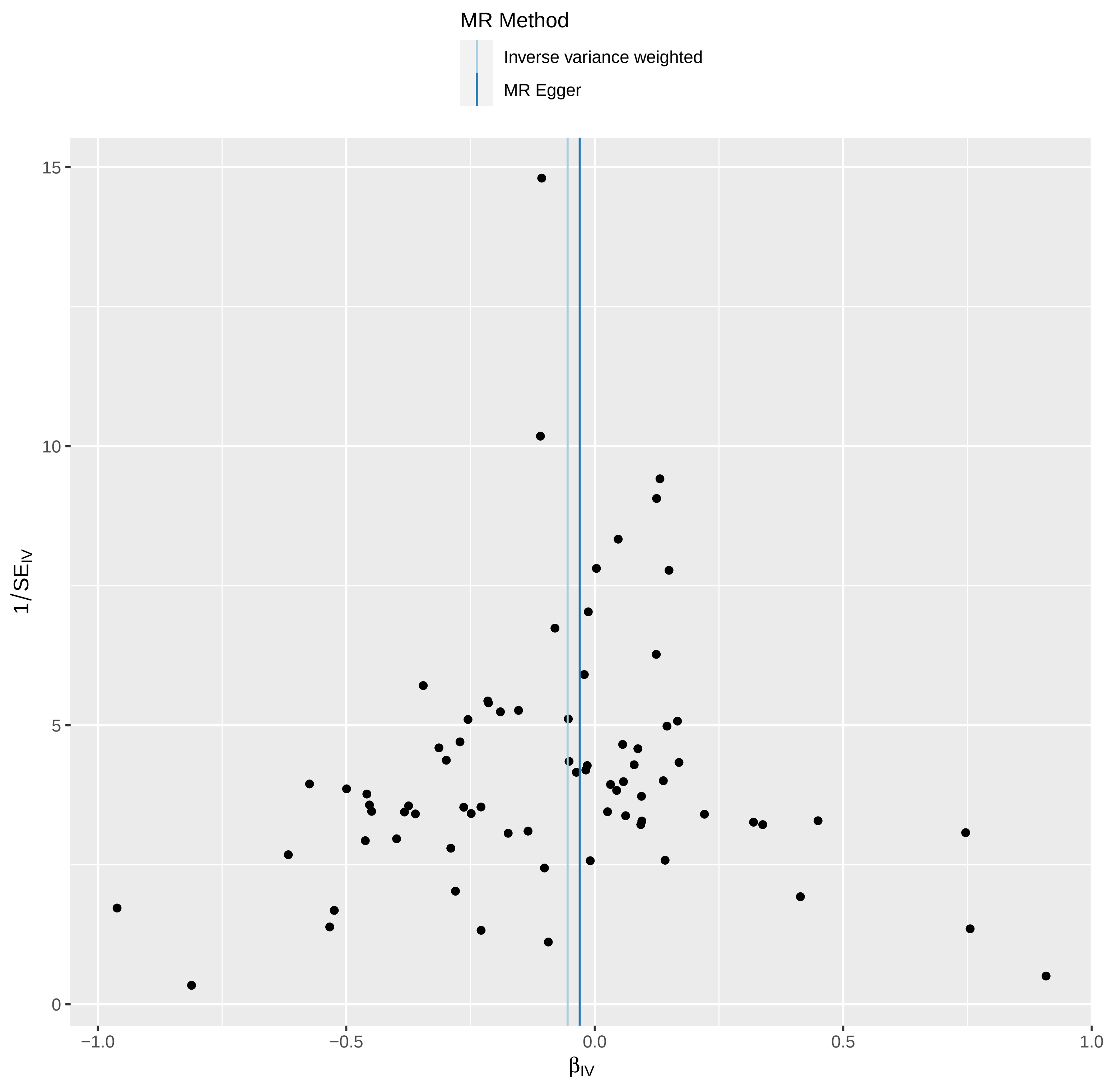
MR Method
Inverse variance weighted
MR Egger
15
10
5
0
1/SE_IV
−1.0
−0.5
0.0
0.5
1.0
β_IV

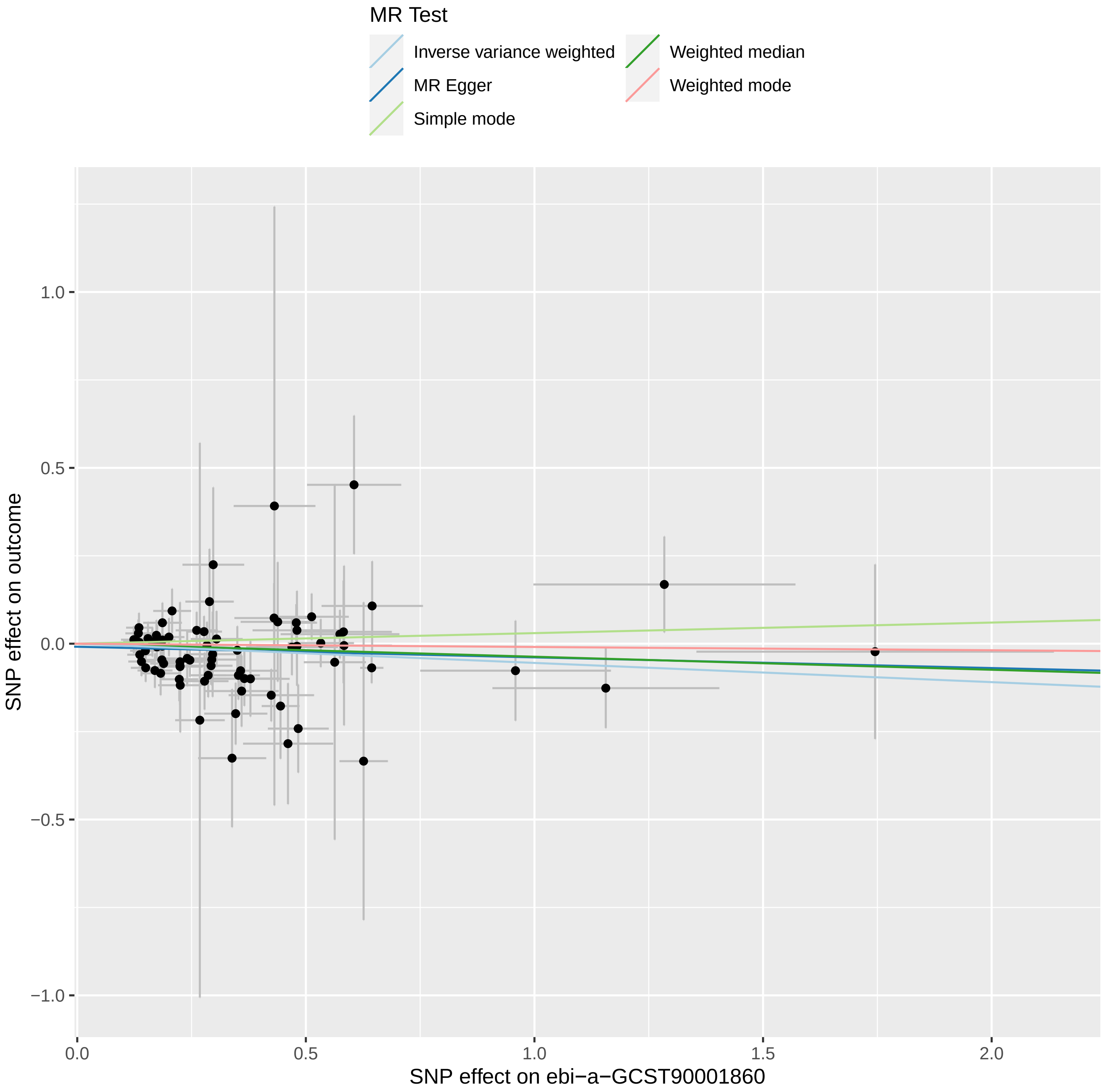

MR Test
Inverse variance weighted
MR Egger
Simple mode
Weighted median
Weighted mode
SNP effect on outcome
1.0
0.5
0.0
−0.5
−1.0
0.0
0.5
1.0
1.5
2.0
SNP effect on ebi−a−GCST90001860

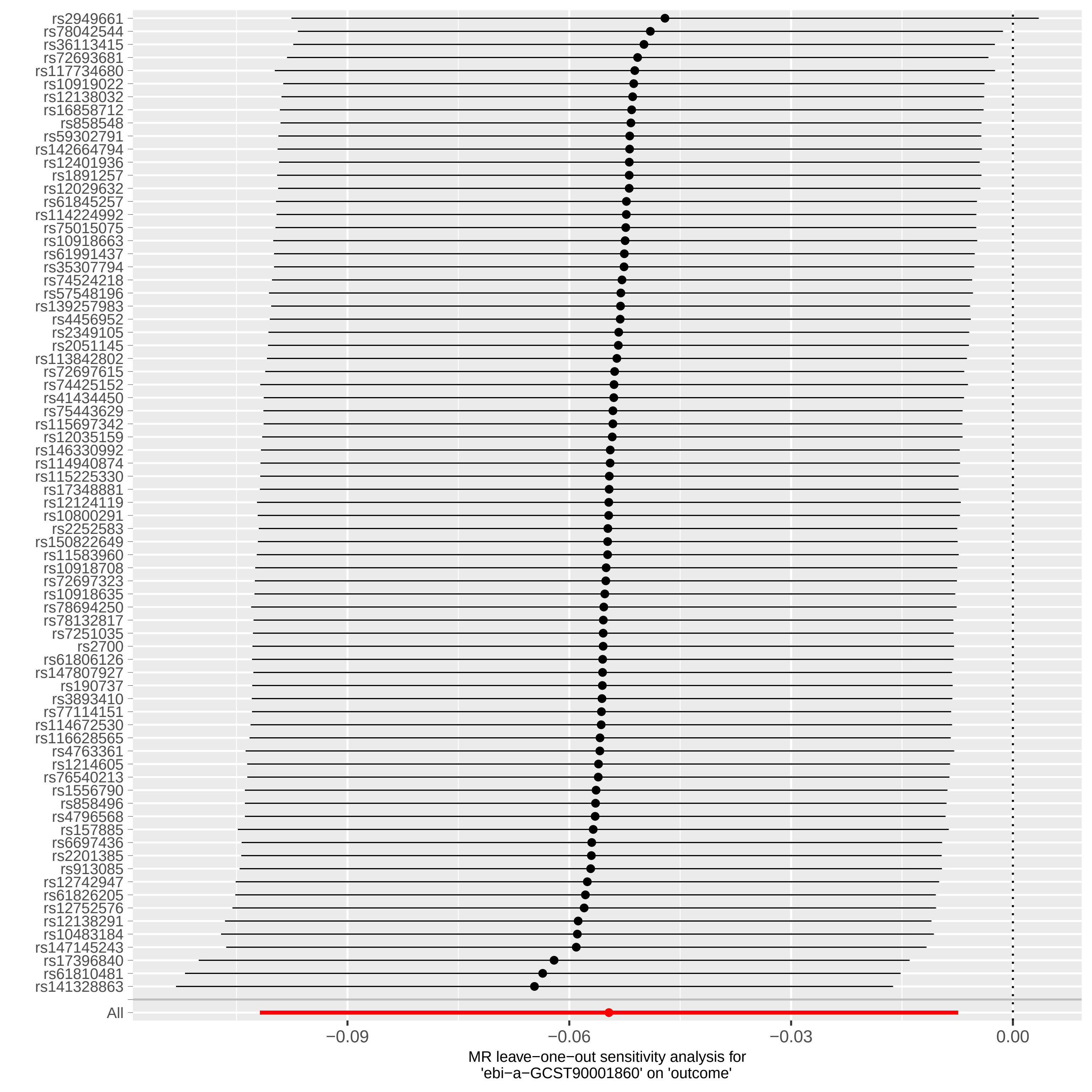

rs2949661
rs78042544
rs36113415
rs72693681
rs117734680
rs10919022
rs12138032
rs16858712
rs858548
rs59302791
rs142664794
rs12401936
rs1891257
rs12029632
rs61845257
rs114224992
rs75015075
rs10918663
rs61991437
rs35307794
rs74524218
rs57548196
rs139257983
rs4456952
rs2349105
rs2051145
rs113842802
rs72697615
rs74425152
rs41434450
rs75443629
rs115697342
rs12035159
rs146330992
rs114940874
rs115225330
rs17348881
rs12124119
rs10800291
rs2252583
rs150822649
rs11583960
rs10918708
rs72697323
rs10918635
rs78694250
rs78132817
rs7251035
rs2700
rs61806126
rs147807927
rs190737
rs3893410
rs77114151
rs114672530
rs116628565
rs4763361
rs1214605
rs76540213
rs1556790
rs858496
rs4796568
rs157885
rs6697436
rs2201385
rs913085
rs12742947
rs61826205
rs12752576
rs12138291
rs10483184
rs147145243
rs17396840
rs61810481
rs141328863
All
−0.09
−0.06
−0.03
0.00
MR leave−one−out sensitivity analysis for
'ebi−a−GCST90001860' on 'outcome'

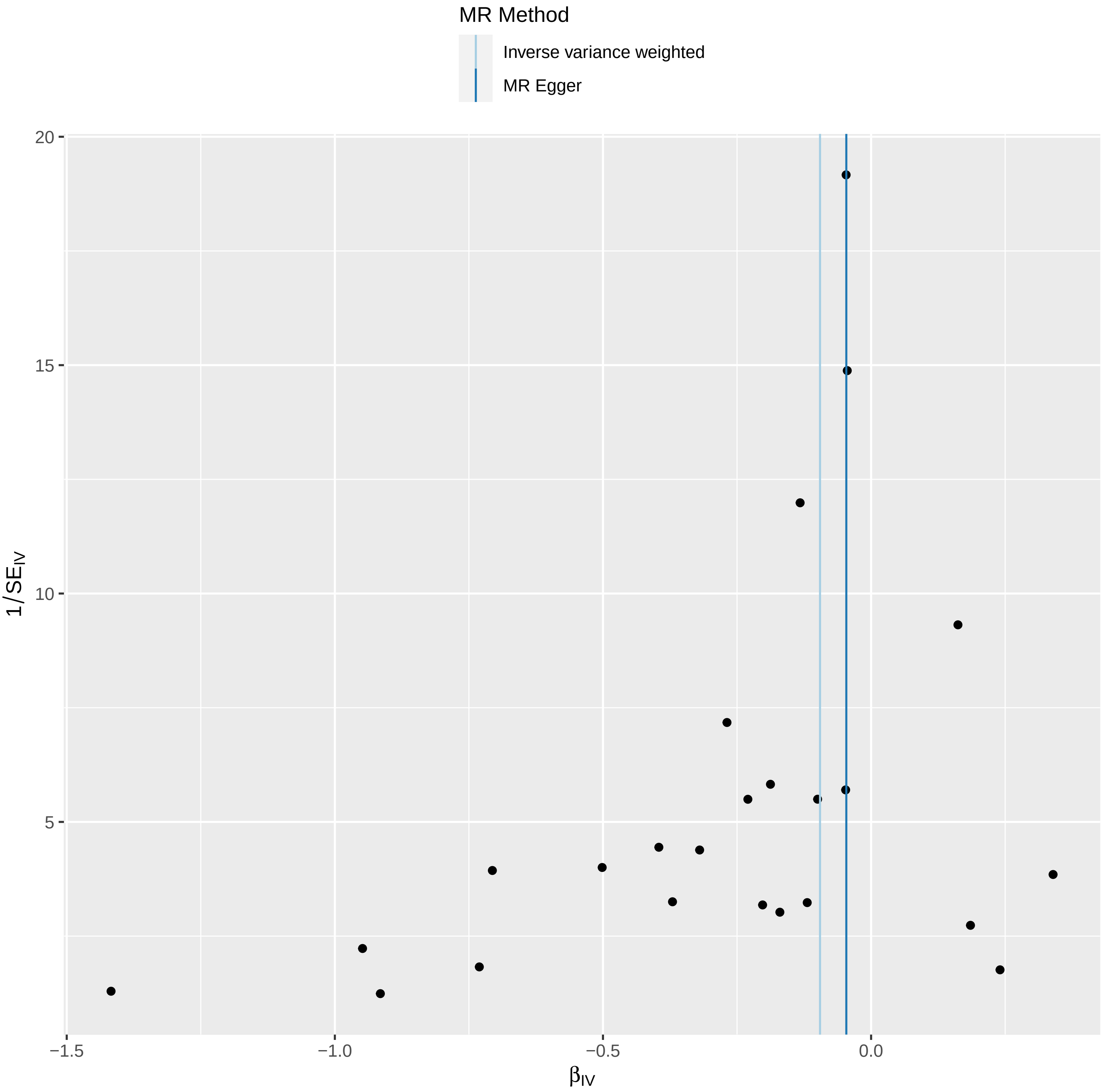
MR Method
Inverse variance weighted
MR Egger
20
15
10
5
1/SE_IV
−1.5
−1.0
−0.5
0.0
β_IV

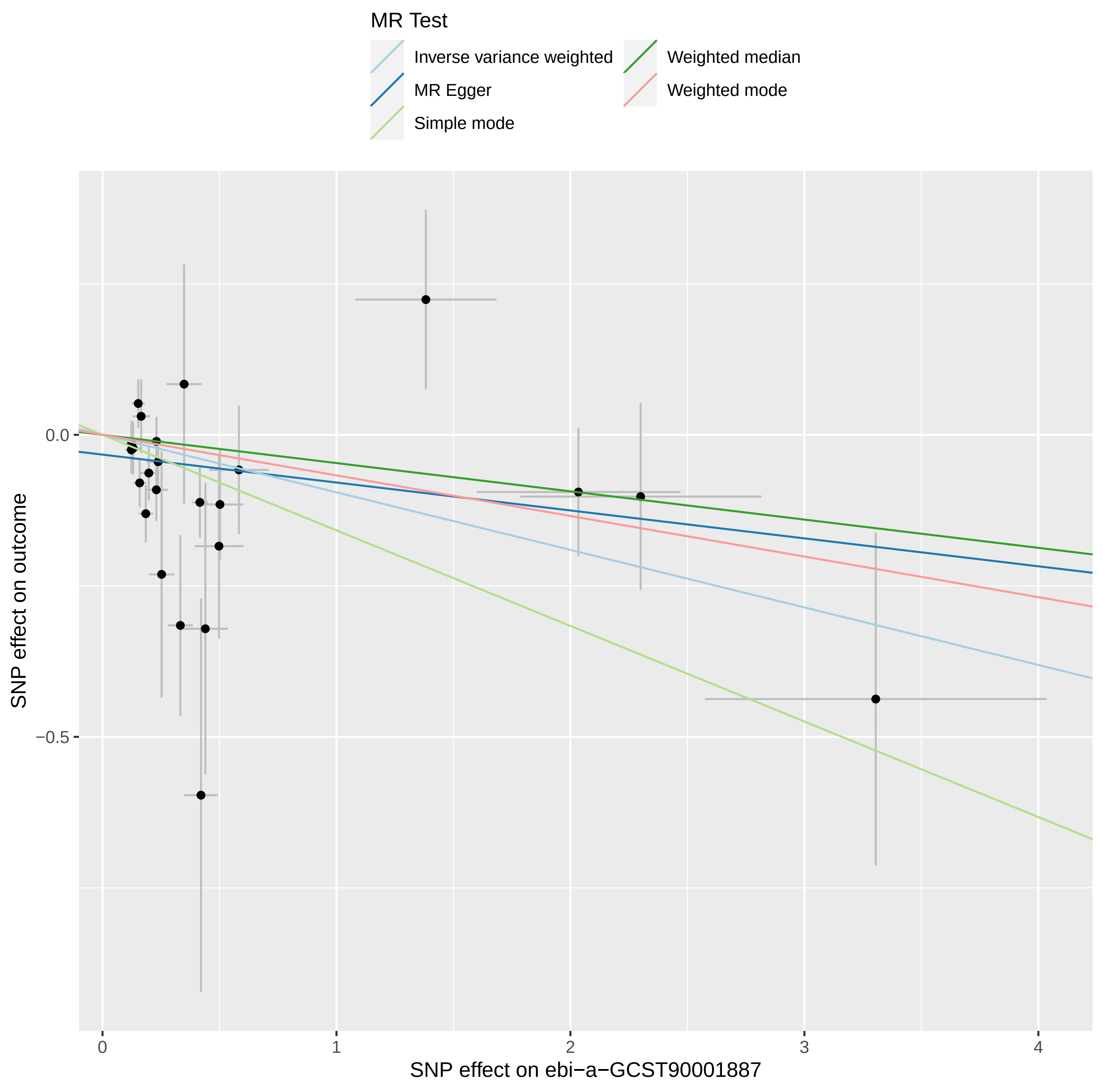
MR Test
Inverse variance weighted
MR Egger
Simple mode
Weighted median
Weighted mode
0.0
−0.5
0
1
2
3
4
SNP effect on outcome
SNP effect on ebi−a−GCST90001887

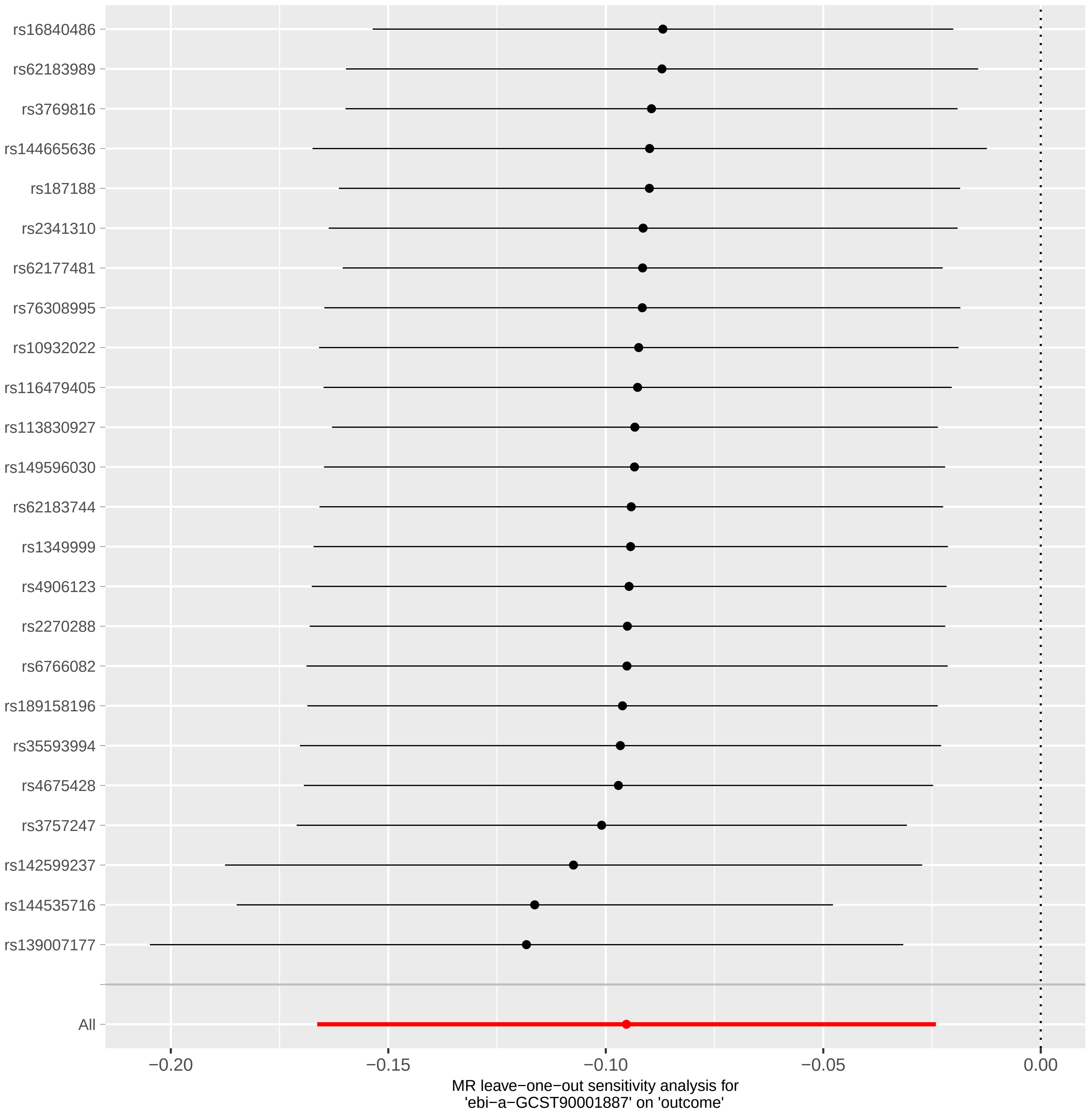
rs16840486
rs62183989
rs3769816
rs144665636
rs187188
rs2341310
rs62177481
rs76308995
rs10932022
rs116479405
rs113830927
rs149596030
rs62183744
rs1349999
rs4906123
rs2270288
rs6766082
rs189158196
rs35593994
rs4675428
rs3757247
rs142599237
rs144535716
rs139007177
All
−0.20
−0.15
−0.10
−0.05
0.00
MR leave−one−out sensitivity analysis for
'ebi−a−GCST90001887' on 'outcome'

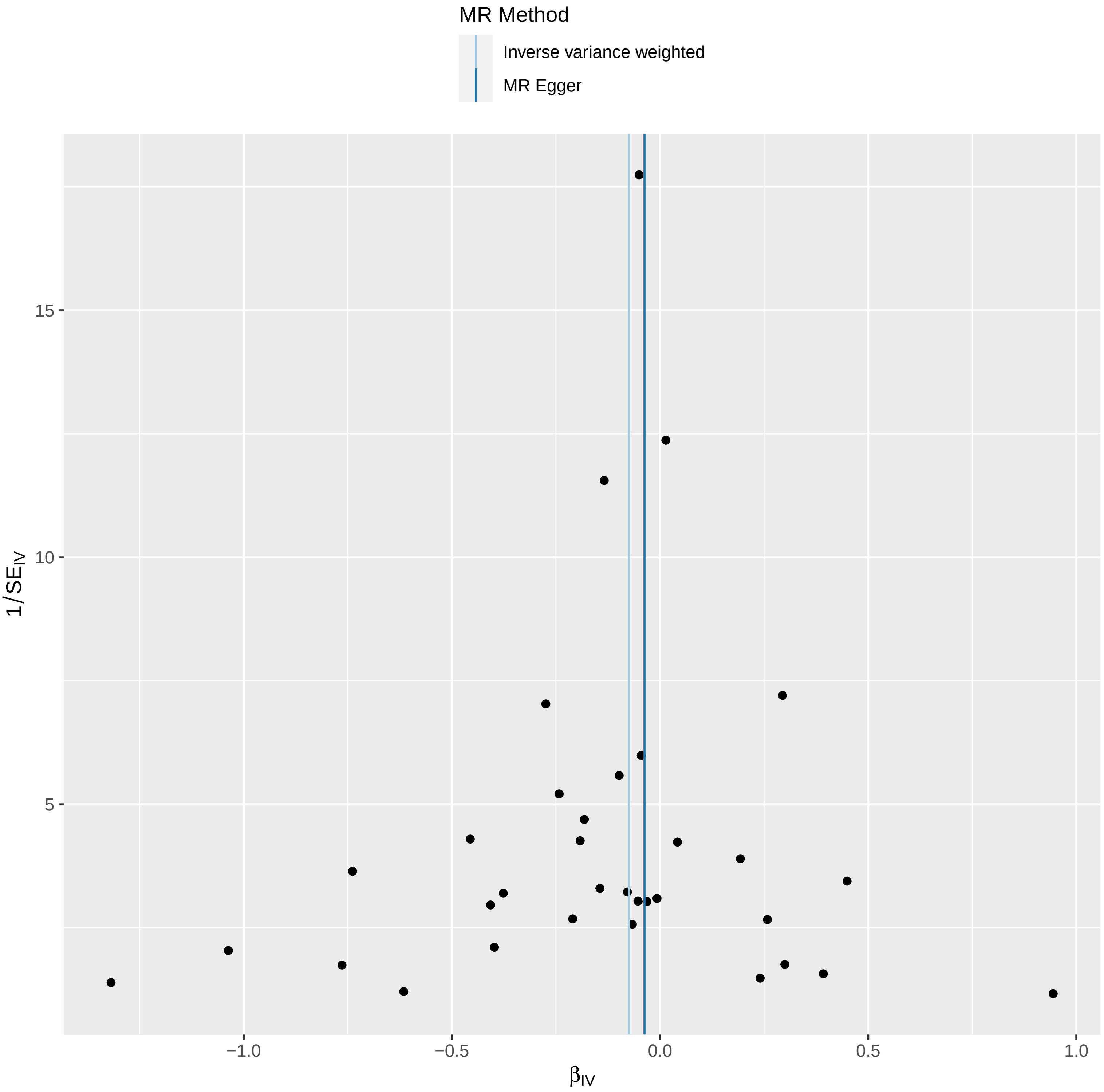

MR Method
Inverse variance weighted
MR Egger
15
10
5
$1/SE_{IV}$
−1.0
−0.5
0.0
0.5
1.0
β_{IV}

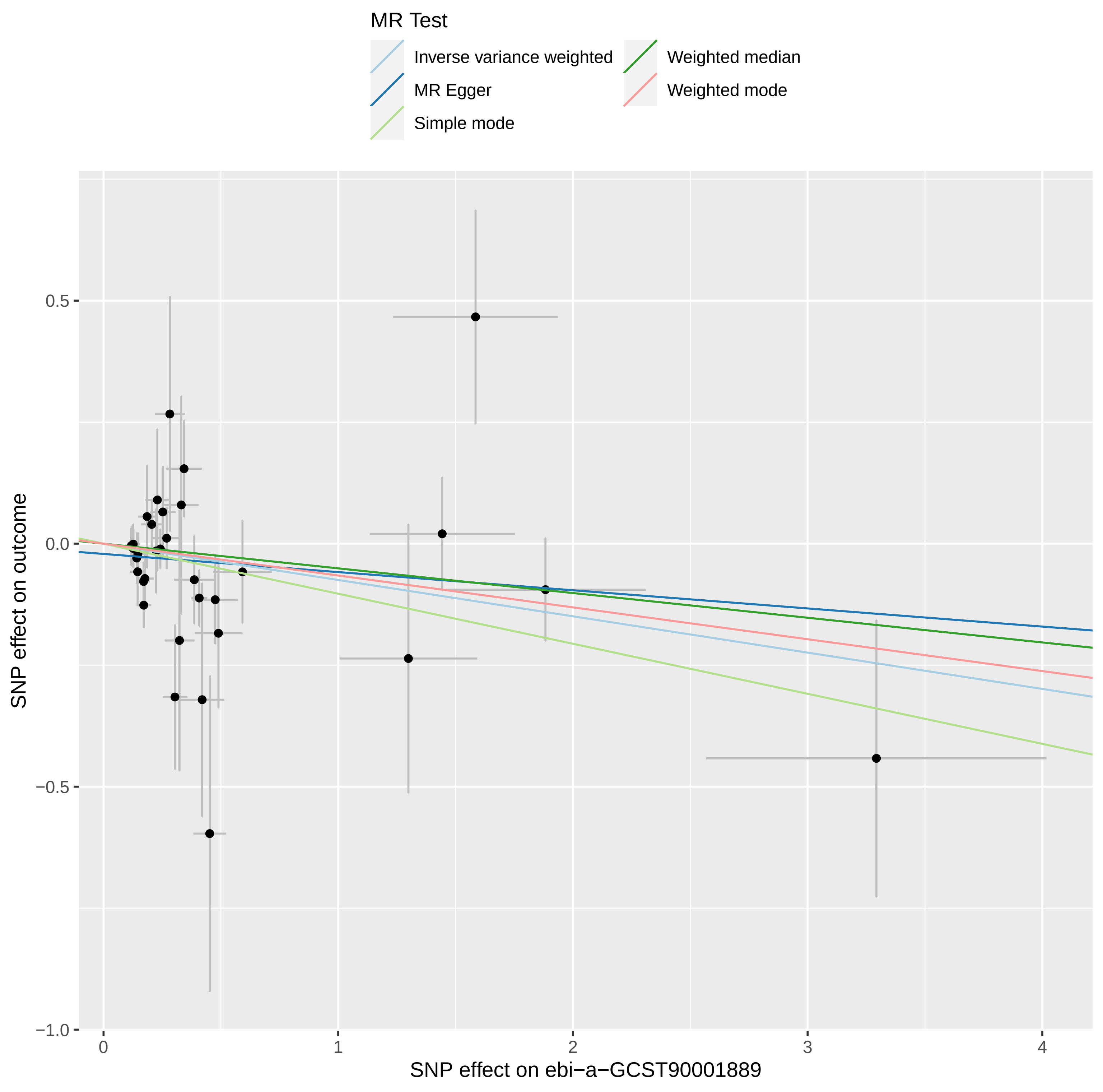
MR Test
Inverse variance weighted
MR Egger
Simple mode
Weighted median
Weighted mode
0.5
0.0
−0.5
−1.0
0
1
2
3
4
SNP effect on outcome
SNP effect on ebi−a−GCST90001889

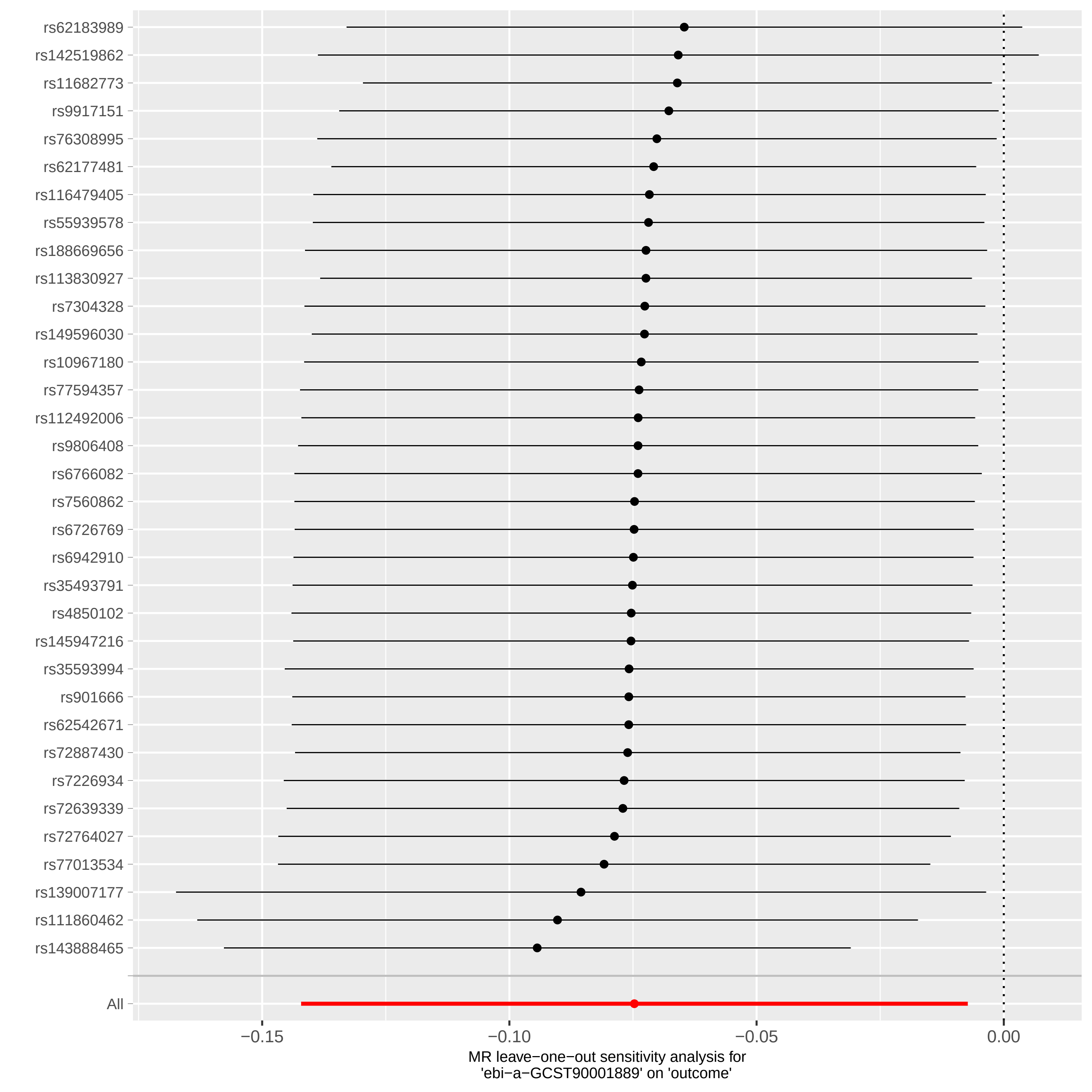

rs62183989
rs142519862
rs11682773
rs9917151
rs76308995
rs62177481
rs116479405
rs55939578
rs188669656
rs113830927
rs7304328
rs149596030
rs10967180
rs77594357
rs112492006
rs9806408
rs6766082
rs7560862
rs6726769
rs6942910
rs35493791
rs4850102
rs145947216
rs35593994
rs901666
rs62542671
rs72887430
rs7226934
rs72639339
rs72764027
rs77013534
rs139007177
rs111860462
rs143888465
All
−0.15
−0.10
−0.05
0.00
MR leave−one−out sensitivity analysis for
'ebi−a−GCST90001889' on 'outcome'

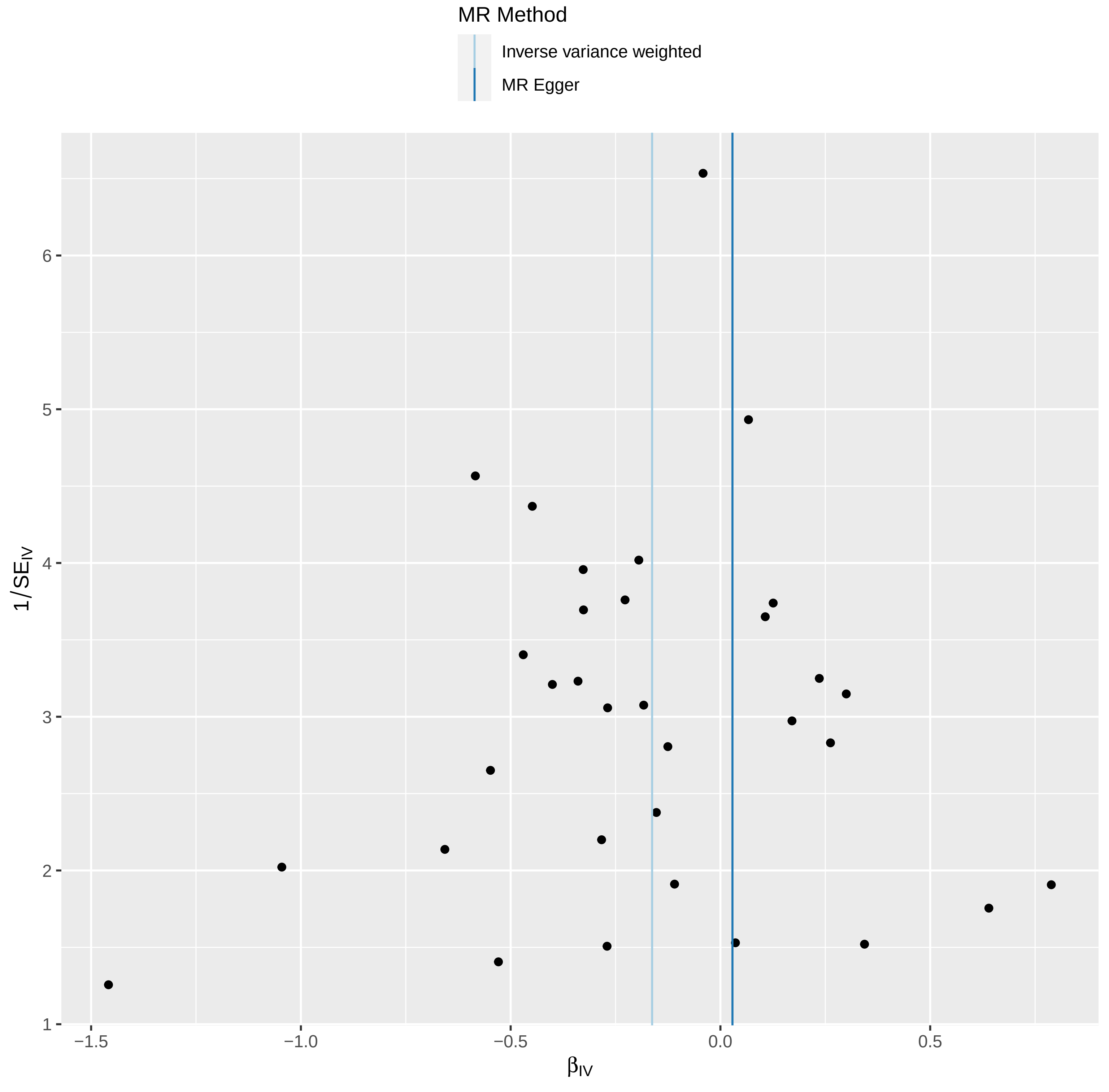
MR Method
Inverse variance weighted
MR Egger
1/SE$_{IV}$
6
5
4
3
2
1
−1.5
−1.0
−0.5
0.0
0.5
β$_{IV}$

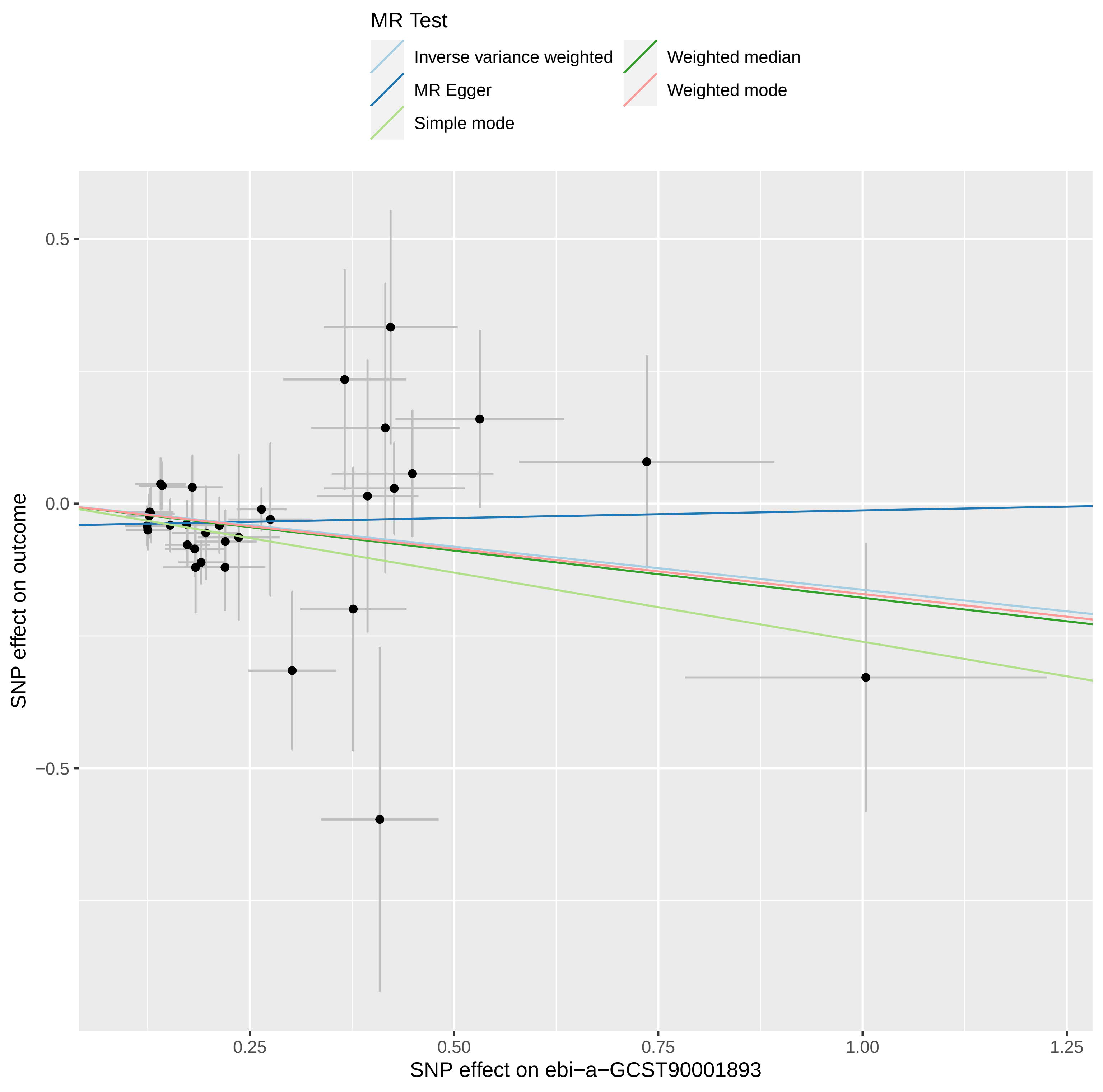

MR Test
Inverse variance weighted
MR Egger
Simple mode
Weighted median
Weighted mode
0.5
0.0
−0.5
SNP effect on outcome
0.25
0.50
0.75
1.00
1.25
SNP effect on ebi−a−GCST90001893

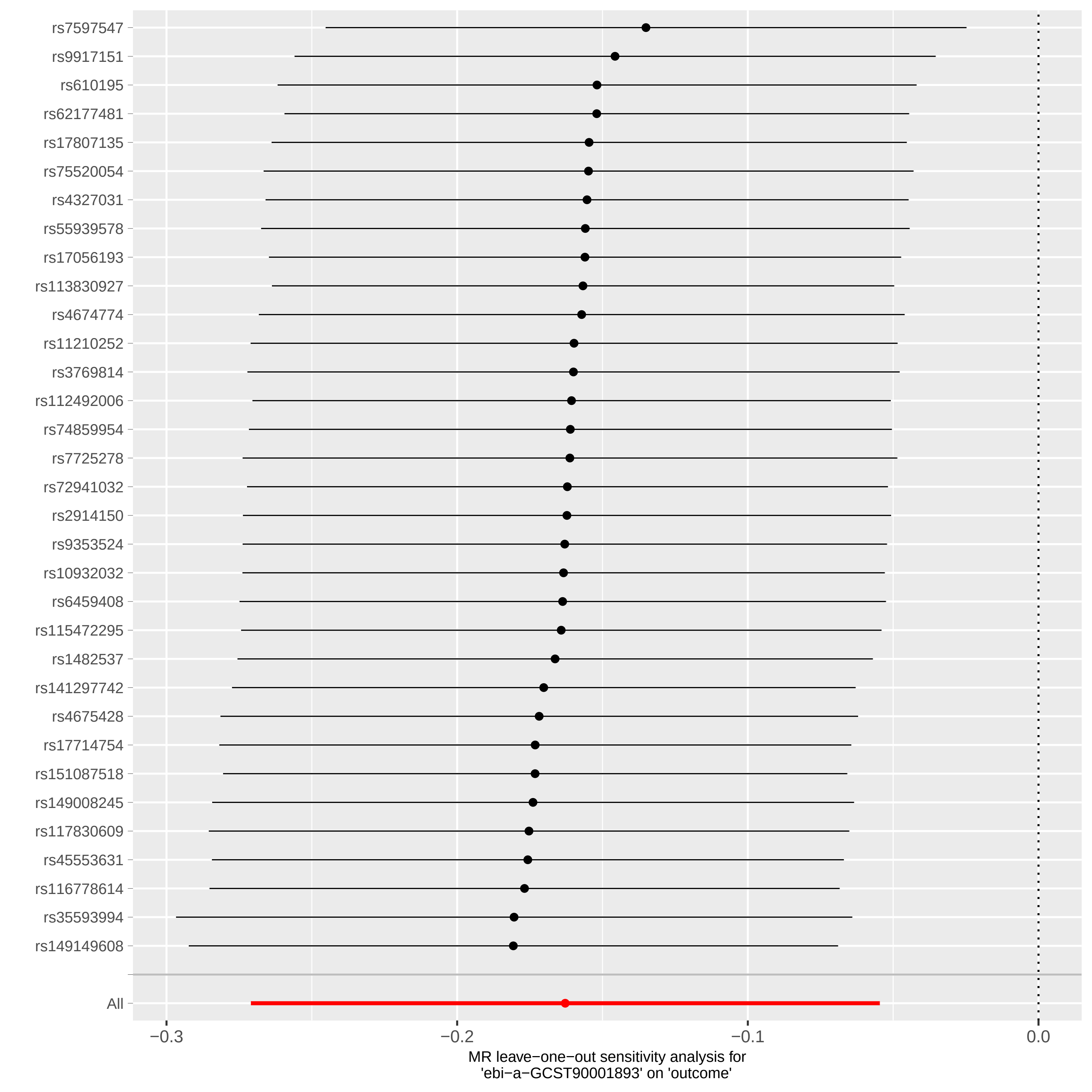
rs7597547
rs9917151
rs610195
rs62177481
rs17807135
rs75520054
rs4327031
rs55939578
rs17056193
rs113830927
rs4674774
rs11210252
rs3769814
rs112492006
rs74859954
rs7725278
rs72941032
rs2914150
rs9353524
rs10932032
rs6459408
rs115472295
rs1482537
rs141297742
rs4675428
rs17714754
rs151087518
rs149008245
rs117830609
rs45553631
rs116778614
rs35593994
rs149149608
All
−0.3
−0.2
−0.1
0.0
MR leave−one−out sensitivity analysis for
'ebi−a−GCST90001893' on 'outcome'

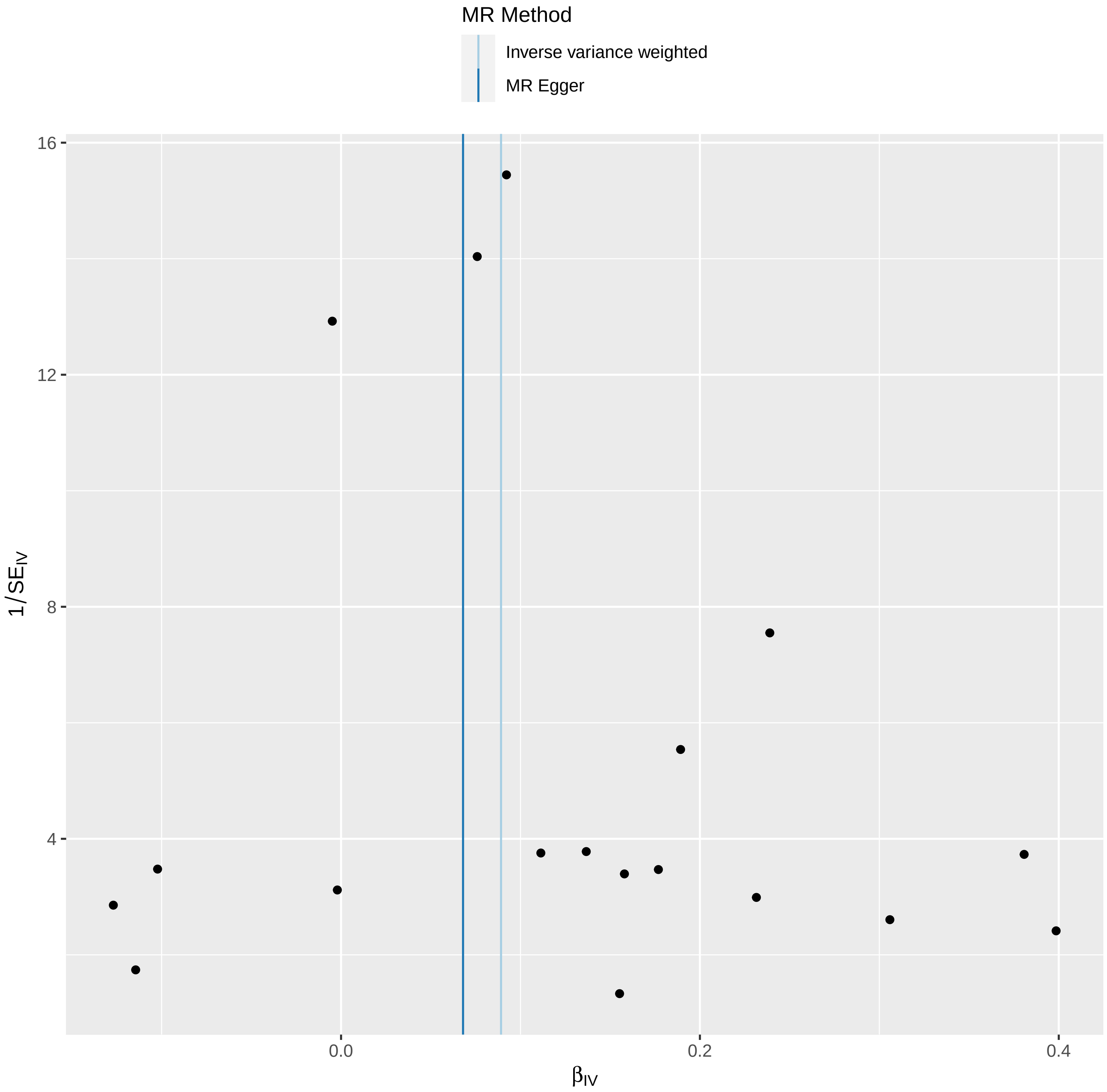
MR Method
Inverse variance weighted
MR Egger
16
12
8
4
0.0
0.2
0.4
$1/SE_{IV}$
β_{IV}

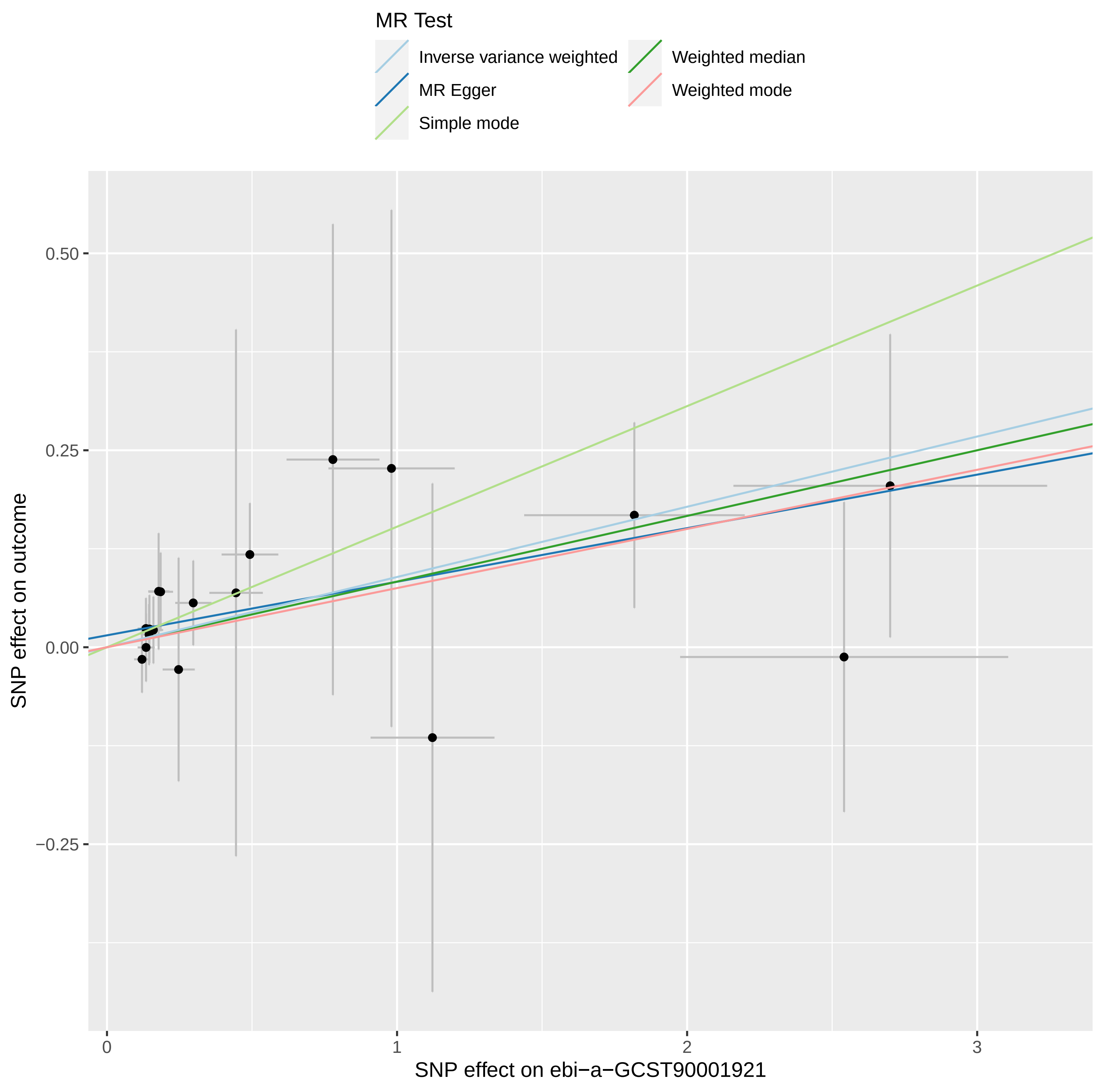
MR Test
Inverse variance weighted
MR Egger
Simple mode
Weighted median
Weighted mode
0.50
0.25
0.00
−0.25
0
1
2
3
SNP effect on outcome
SNP effect on ebi−a−GCST90001921

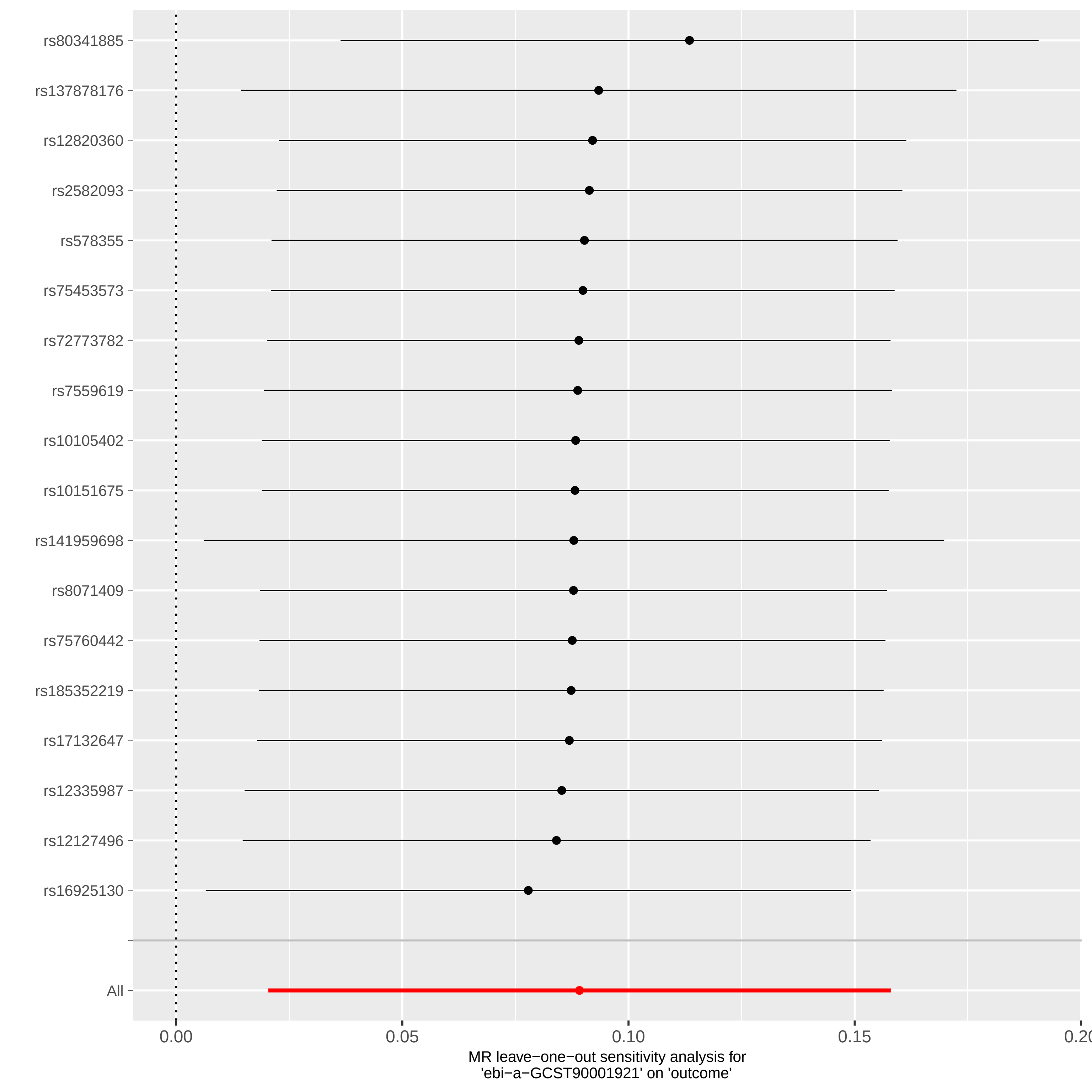

rs80341885
rs137878176
rs12820360
rs2582093
rs578355
rs75453573
rs72773782
rs7559619
rs10105402
rs10151675
rs141959698
rs8071409
rs75760442
rs185352219
rs17132647
rs12335987
rs12127496
rs16925130
All
0.00
0.05
0.10
0.15
0.20
MR leave−one−out sensitivity analysis for
'ebi−a−GCST90001921' on 'outcome'

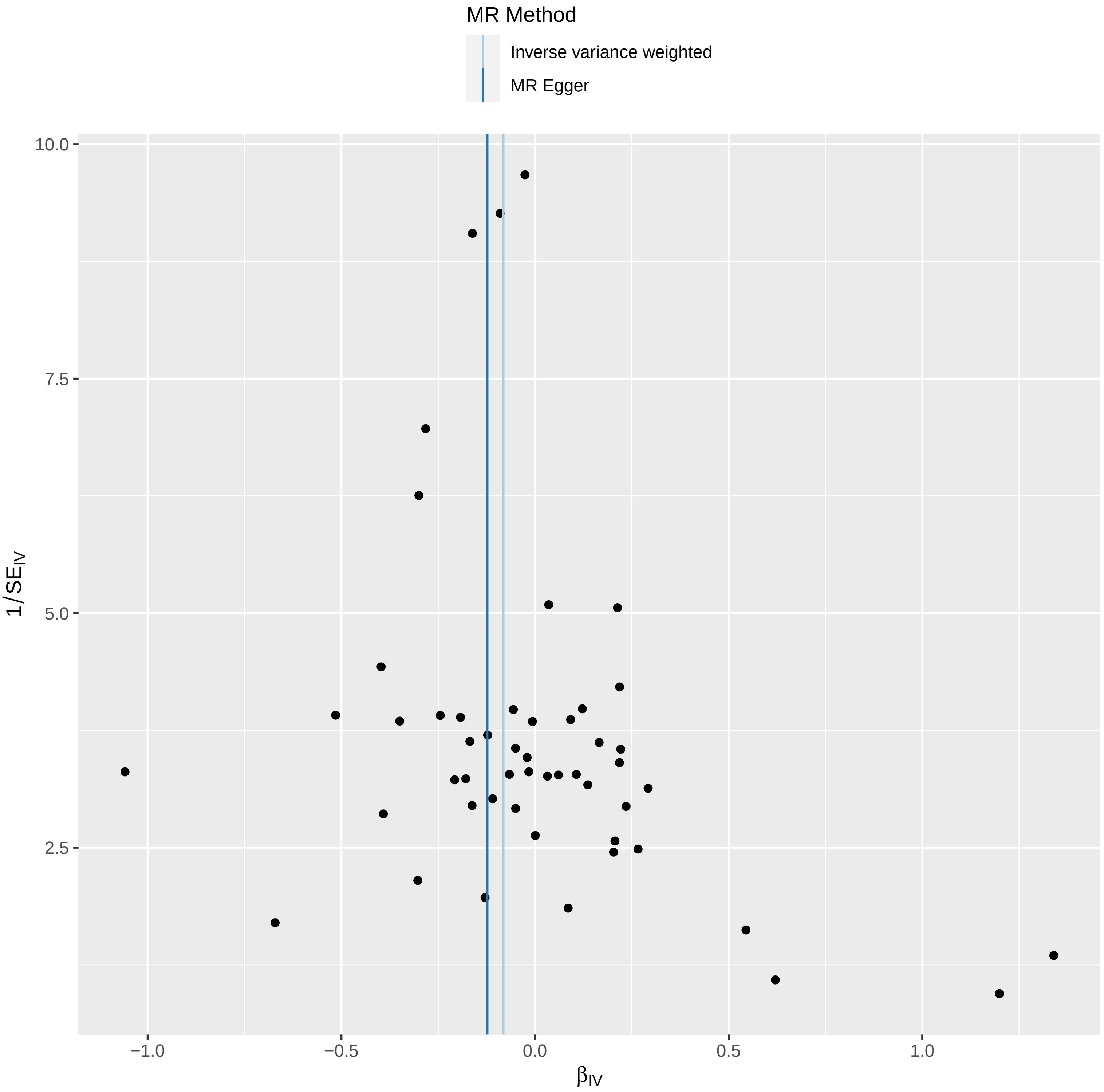

MR Method
Inverse variance weighted
MR Egger
10.0
7.5
5.0
2.5
1/SE_IV
−1.0
−0.5
0.0
0.5
1.0
β_IV

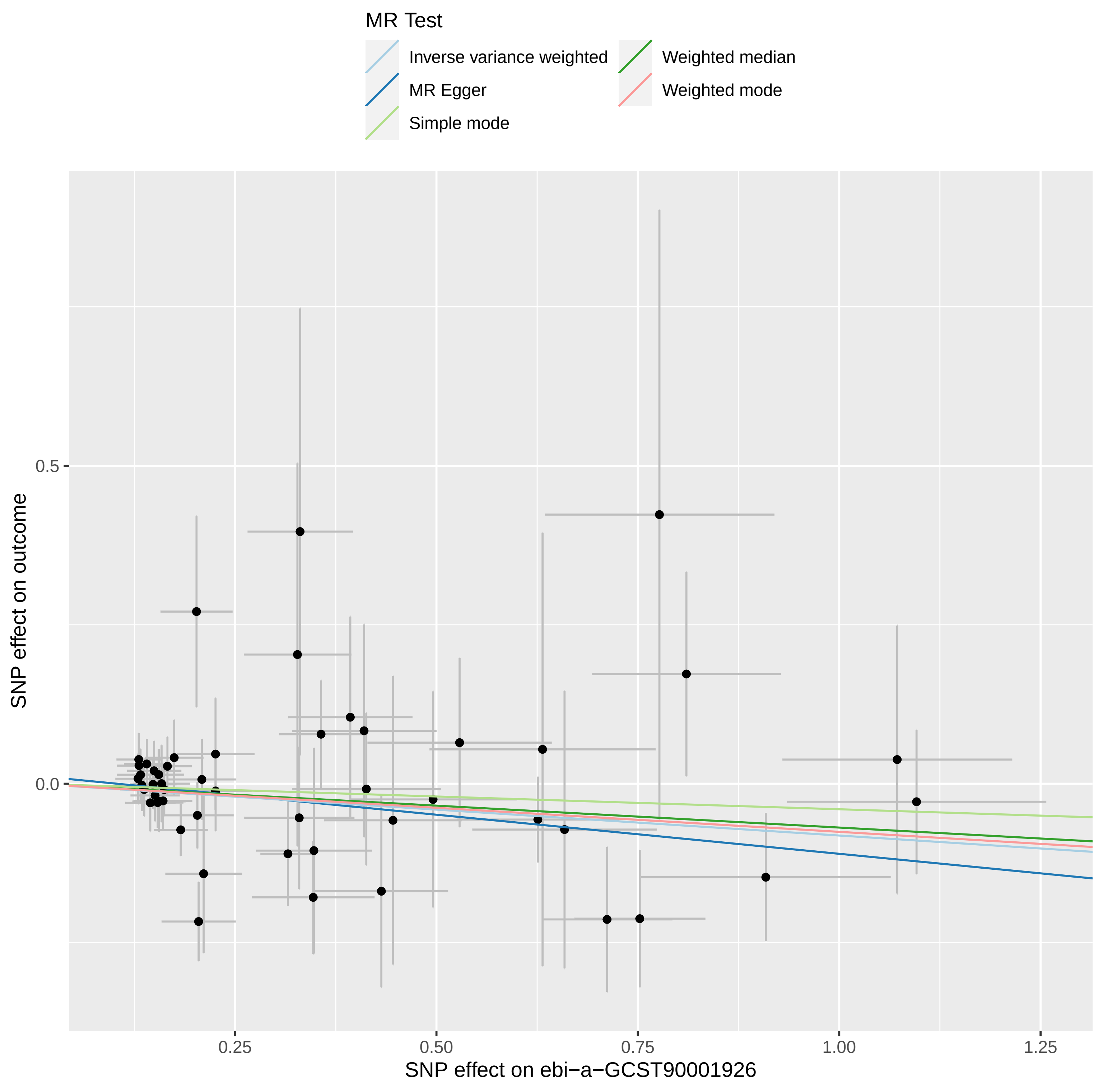

MR Test
Inverse variance weighted
MR Egger
Simple mode
Weighted median
Weighted mode
0.5
0.0
0.25
0.50
0.75
1.00
1.25
SNP effect on outcome
SNP effect on ebi−a−GCST90001926

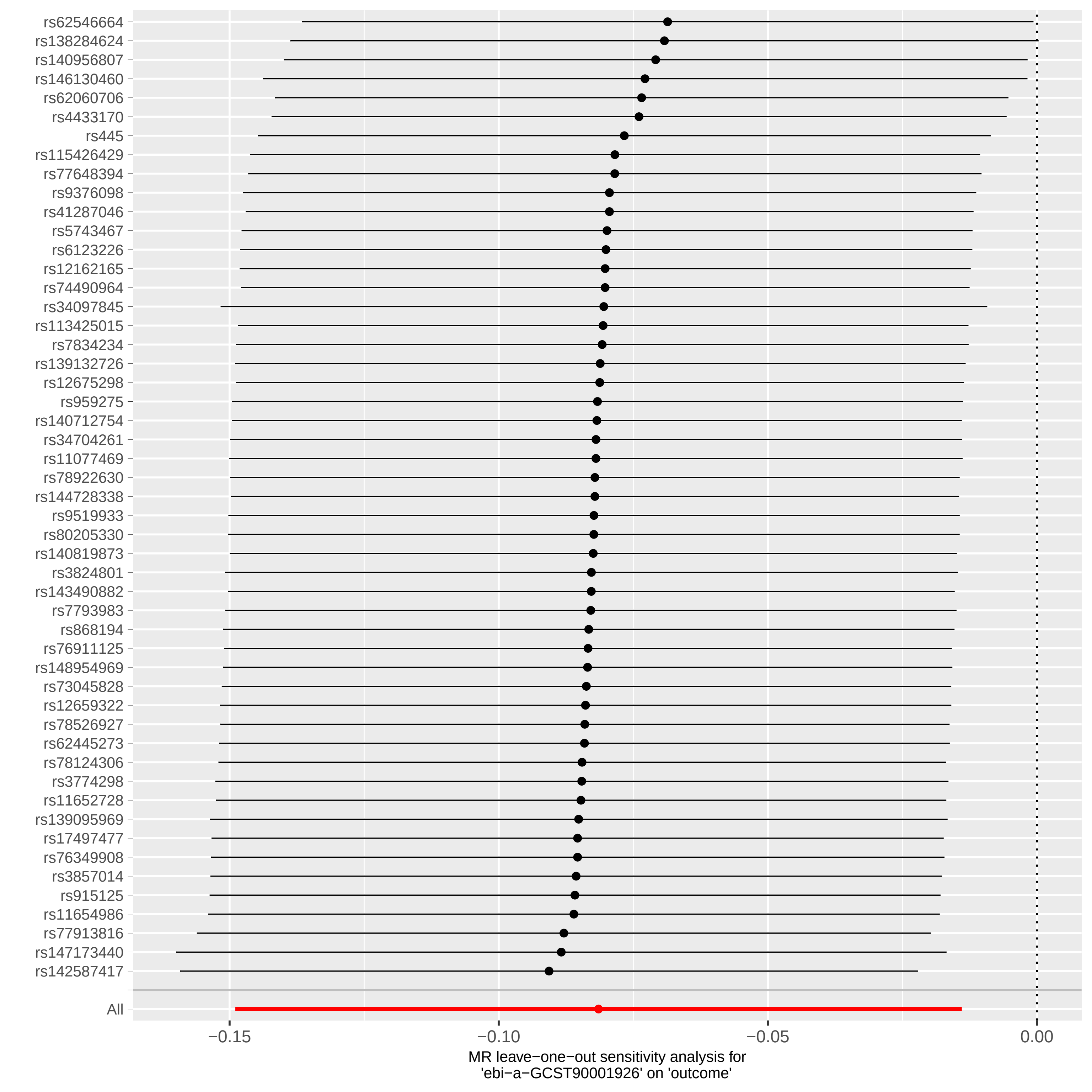

rs62546664
rs138284624
rs140956807
rs146130460
rs62060706
rs4433170
rs445
rs115426429
rs77648394
rs9376098
rs41287046
rs5743467
rs6123226
rs12162165
rs74490964
rs34097845
rs113425015
rs7834234
rs139132726
rs12675298
rs959275
rs140712754
rs34704261
rs11077469
rs78922630
rs144728338
rs9519933
rs80205330
rs140819873
rs3824801
rs143490882
rs7793983
rs868194
rs76911125
rs148954969
rs73045828
rs12659322
rs78526927
rs62445273
rs78124306
rs3774298
rs11652728
rs139095969
rs17497477
rs76349908
rs3857014
rs915125
rs11654986
rs77913816
rs147173440
rs142587417
All
−0.15
−0.10
−0.05
0.00
MR leave−one−out sensitivity analysis for
'ebi−a−GCST90001926' on 'outcome'

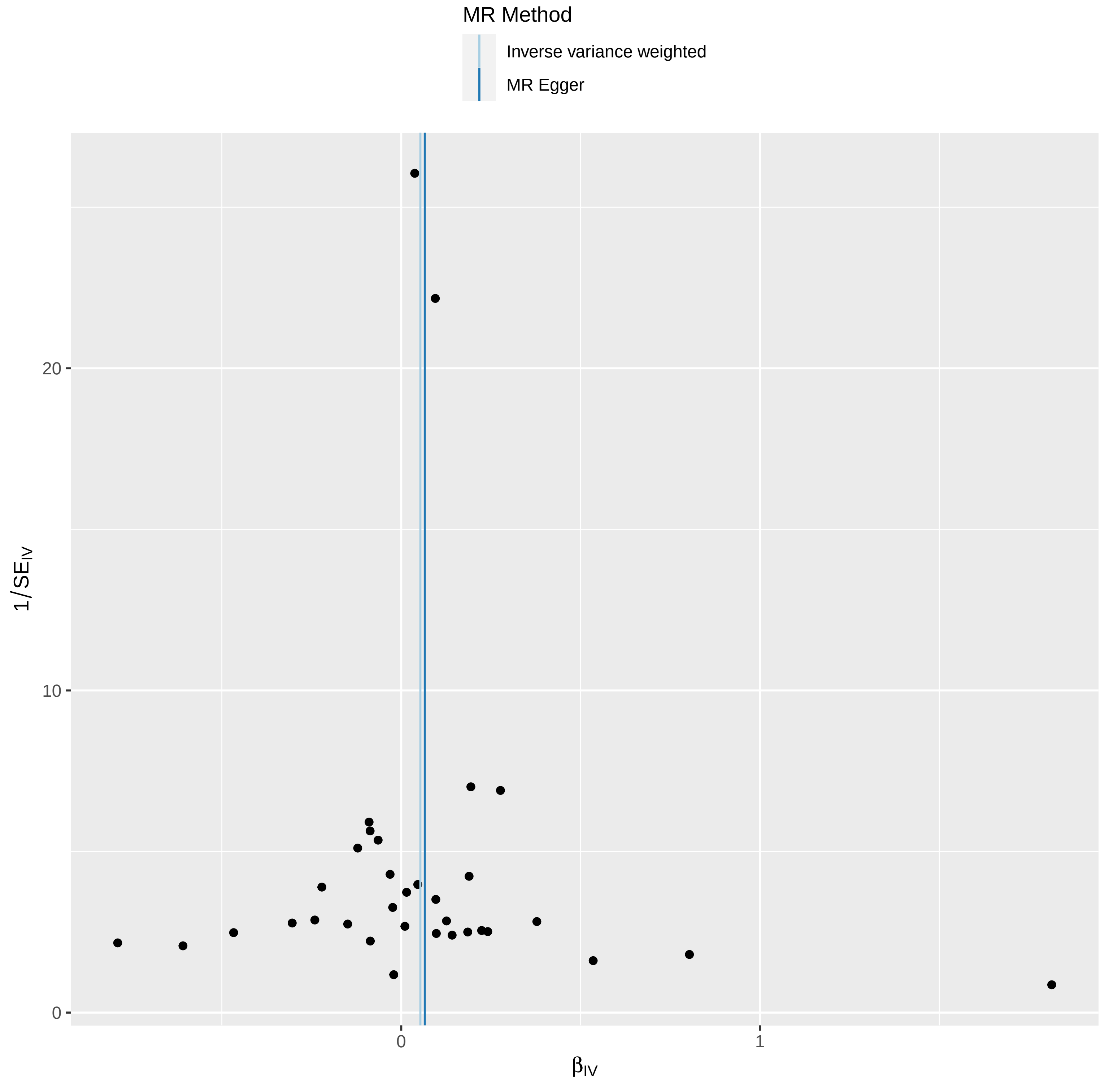

MR Method
Inverse variance weighted
MR Egger
20
10
0
1/SE_IV
0
1
β_IV

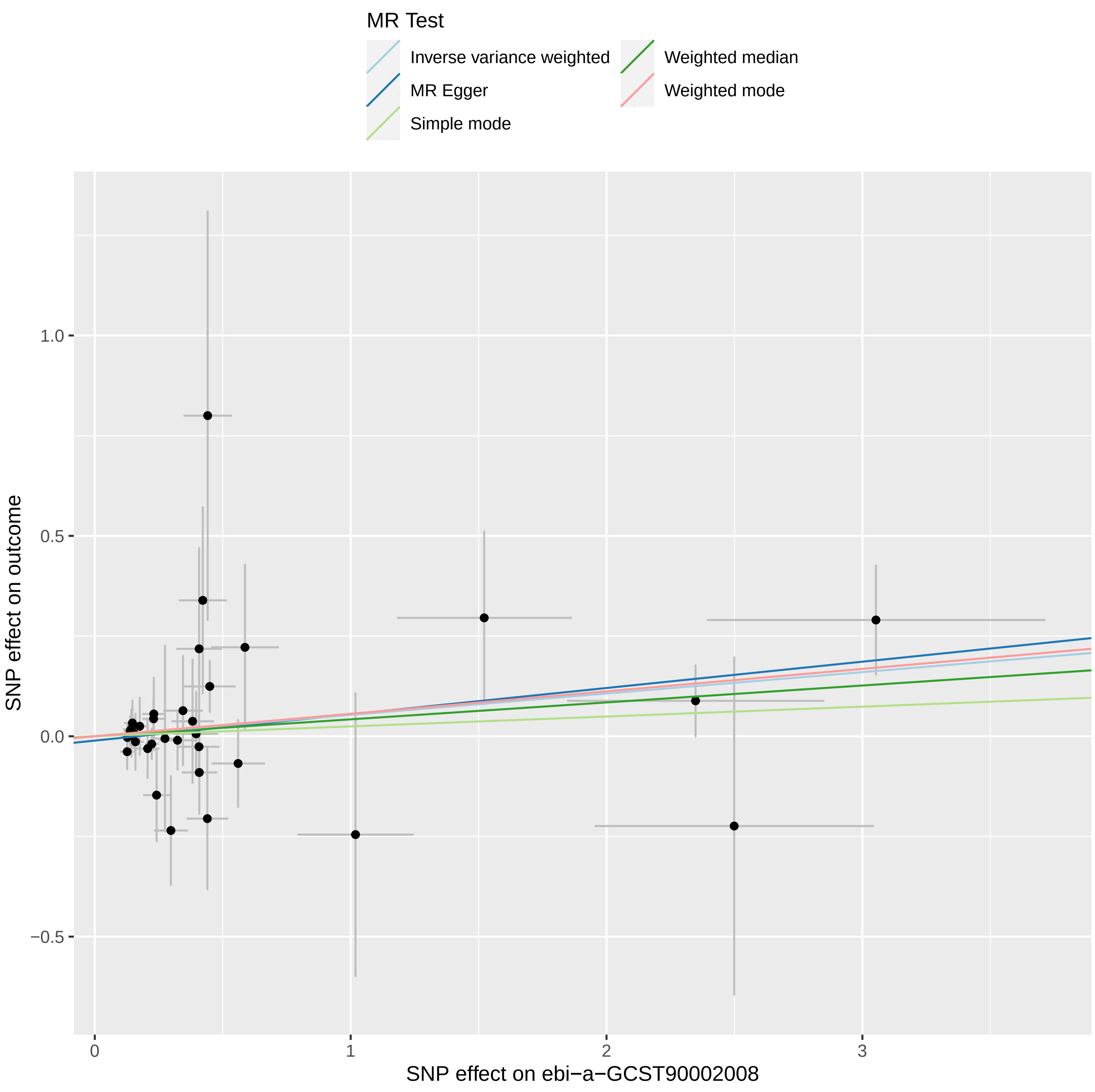
MR Test
Inverse variance weighted
MR Egger
Simple mode
Weighted median
Weighted mode
1.0
0.5
0.0
−0.5
0
1
2
3
SNP effect on outcome
SNP effect on ebi−a−GCST90002008

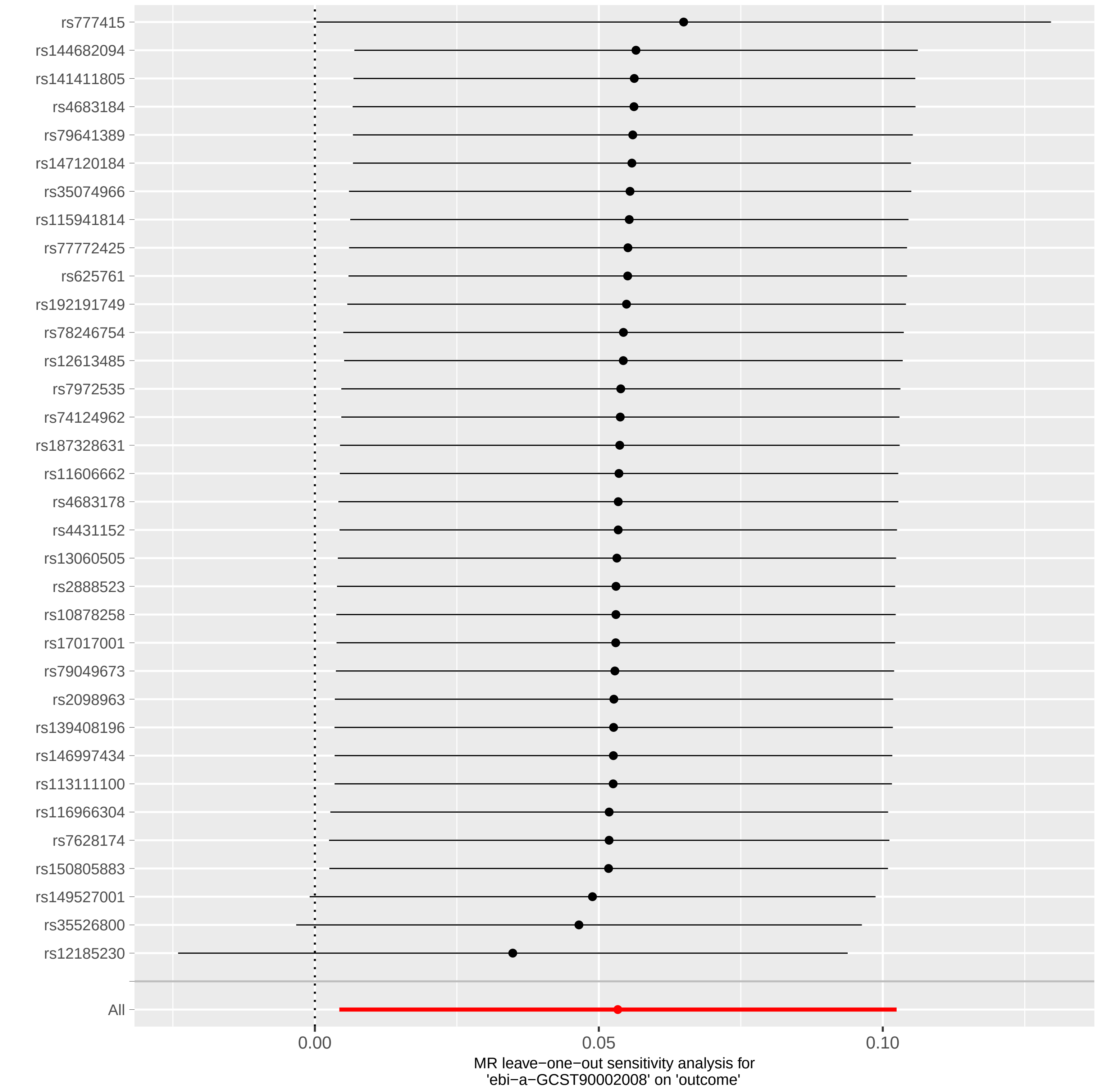
rs777415
rs144682094
rs141411805
rs4683184
rs79641389
rs147120184
rs35074966
rs115941814
rs77772425
rs625761
rs192191749
rs78246754
rs12613485
rs7972535
rs74124962
rs187328631
rs11606662
rs4683178
rs4431152
rs13060505
rs2888523
rs10878258
rs17017001
rs79049673
rs2098963
rs139408196
rs146997434
rs113111100
rs116966304
rs7628174
rs150805883
rs149527001
rs35526800
rs12185230
All
0.00
0.05
0.10
MR leave−one−out sensitivity analysis for
'ebi−a−GCST90002008' on 'outcome'

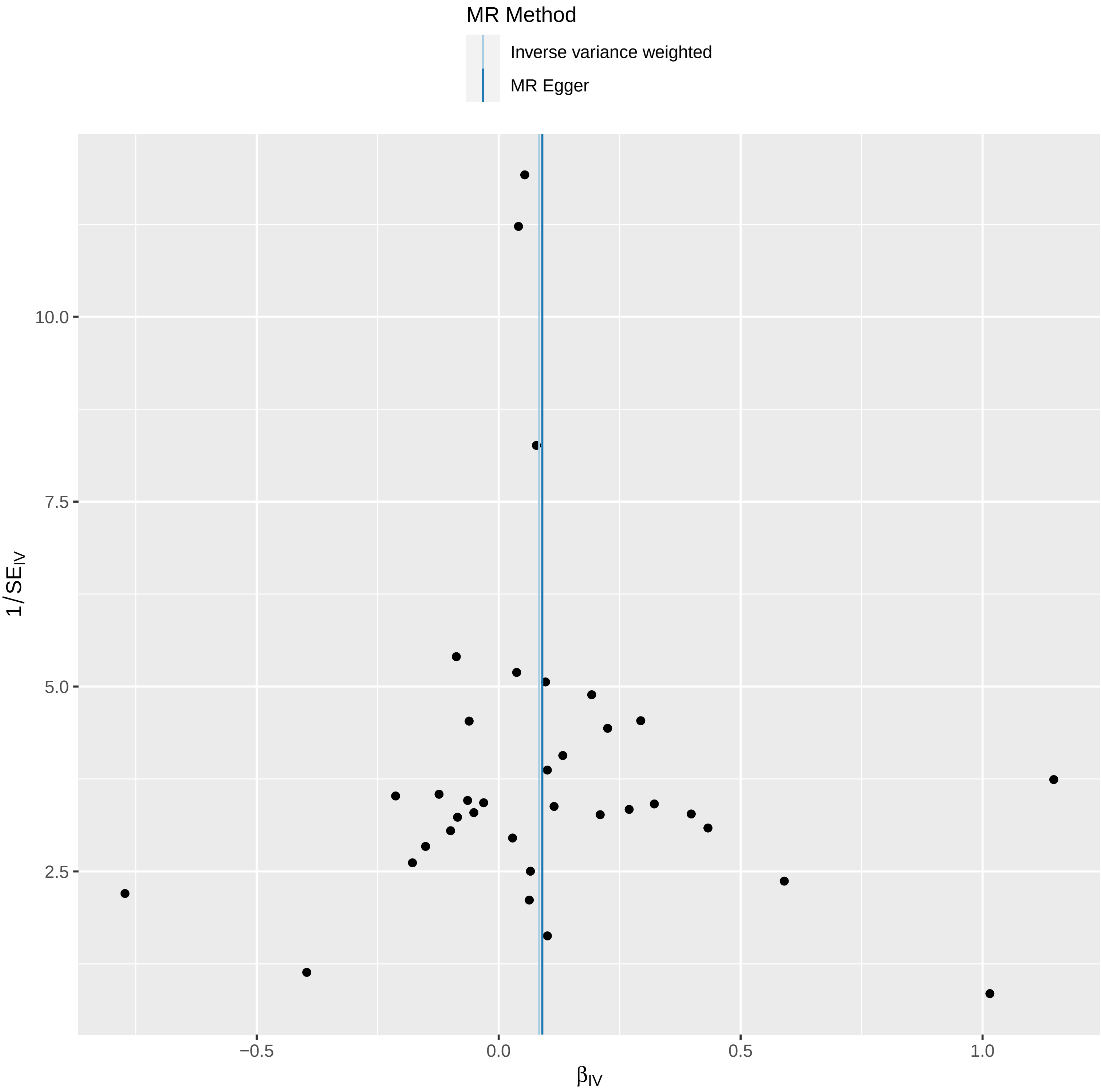
MR Method
Inverse variance weighted
MR Egger
1/SE$_{IV}$
10.0
7.5
5.0
2.5
−0.5
0.0
0.5
1.0
β$_{IV}$

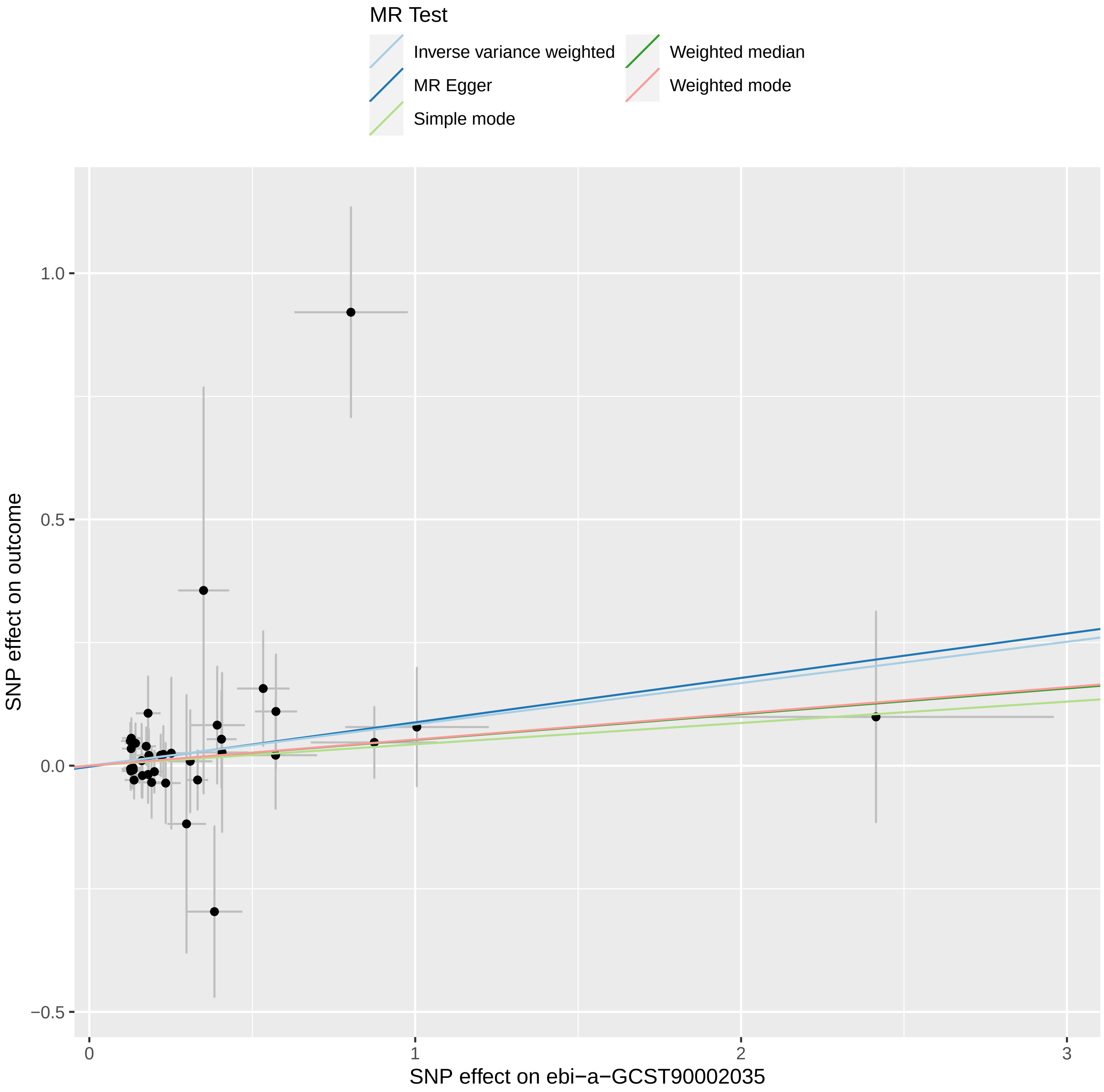
MR Test
Inverse variance weighted
MR Egger
Simple mode
Weighted median
Weighted mode
1.0
0.5
0.0
−0.5
0
1
2
3
SNP effect on outcome
SNP effect on ebi−a−GCST90002035

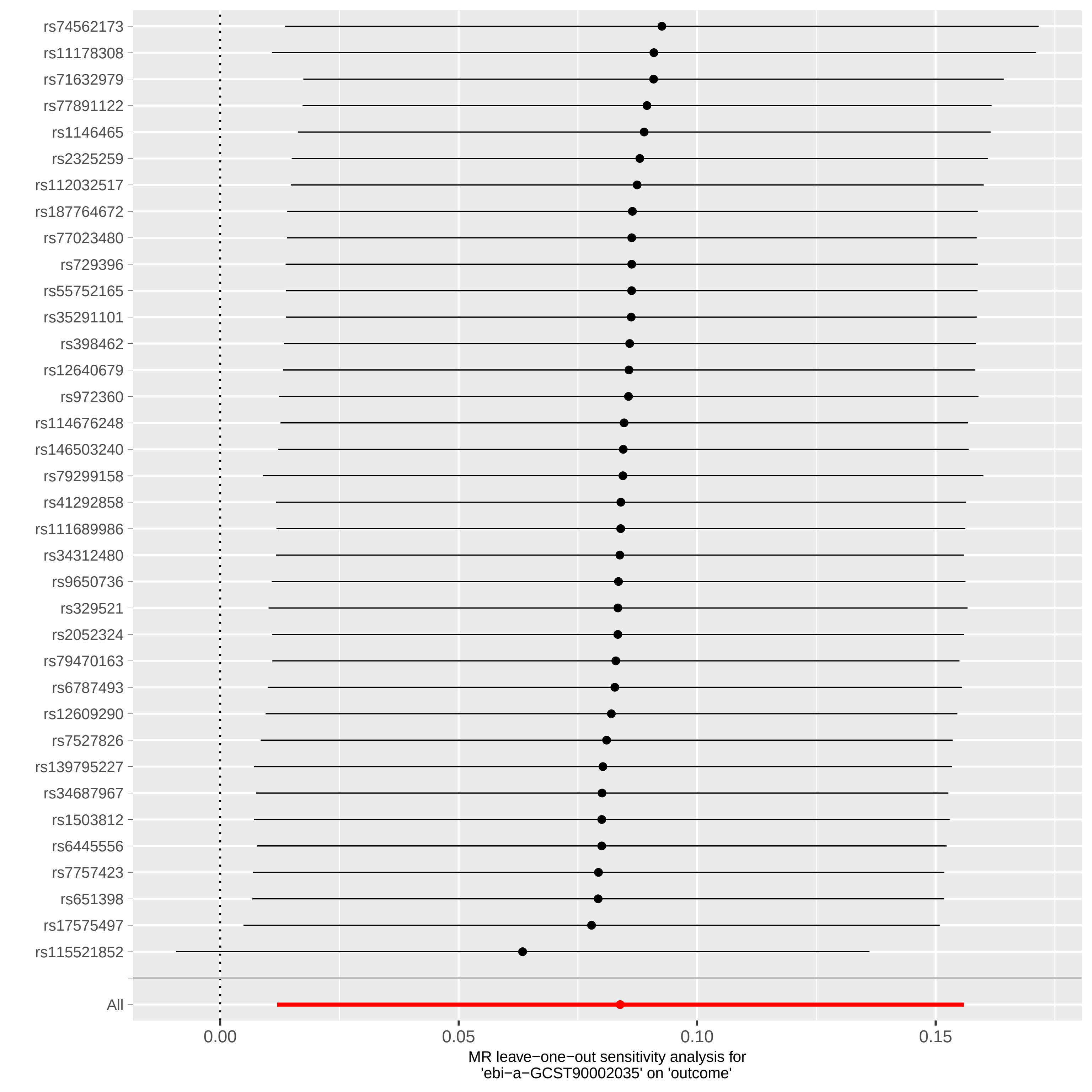
rs74562173
rs11178308
rs71632979
rs77891122
rs1146465
rs2325259
rs112032517
rs187764672
rs77023480
rs729396
rs55752165
rs35291101
rs398462
rs12640679
rs972360
rs114676248
rs146503240
rs79299158
rs41292858
rs111689986
rs34312480
rs9650736
rs329521
rs2052324
rs79470163
rs6787493
rs12609290
rs7527826
rs139795227
rs34687967
rs1503812
rs6445556
rs7757423
rs651398
rs17575497
rs115521852
All
0.00
0.05
0.10
0.15
MR leave−one−out sensitivity analysis for
'ebi−a−GCST90002035' on 'outcome'

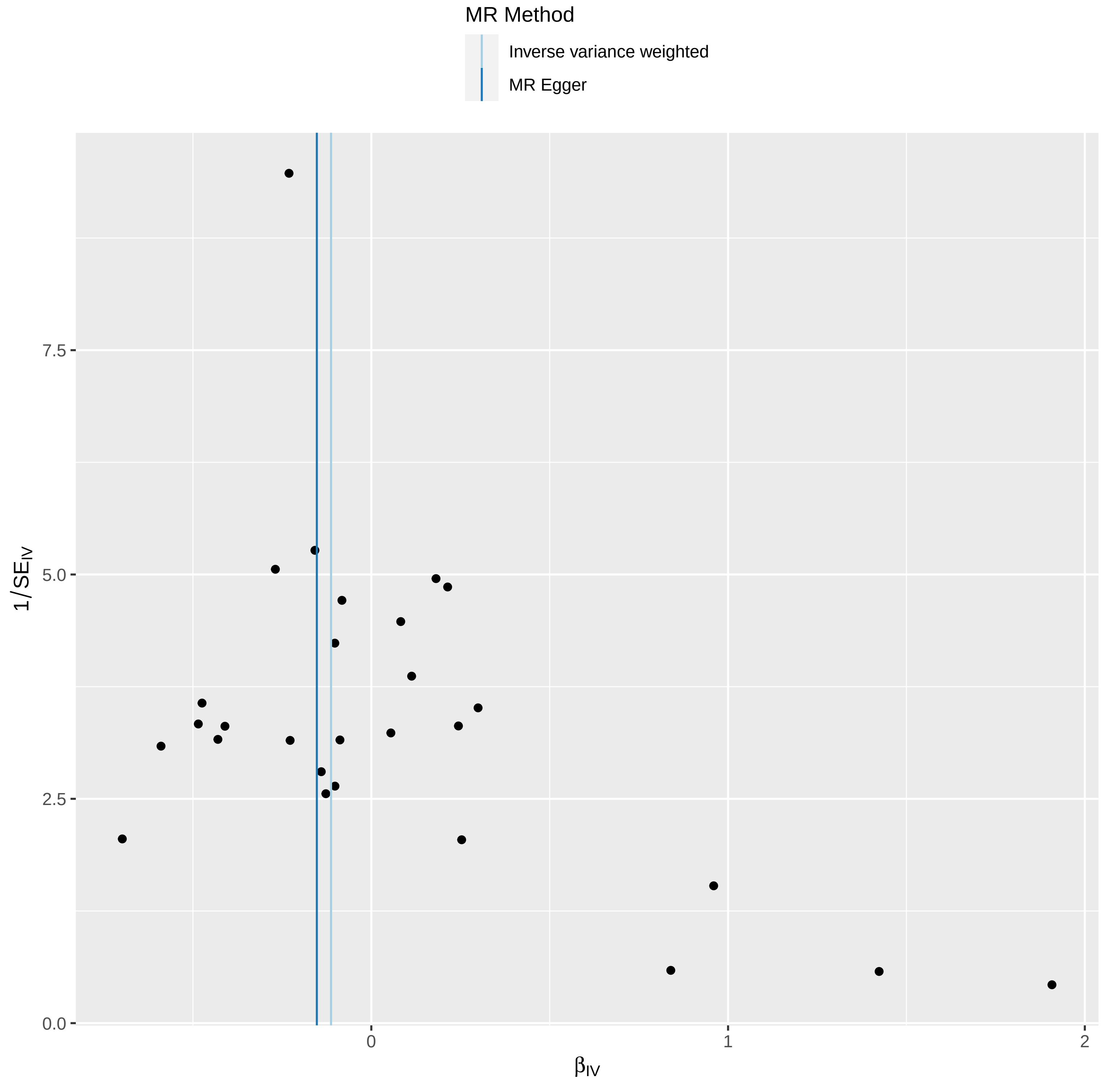
MR Method
Inverse variance weighted
MR Egger
7.5
5.0
2.5
0.0
1/SE$_{IV}$
0
1
2
β$_{IV}$

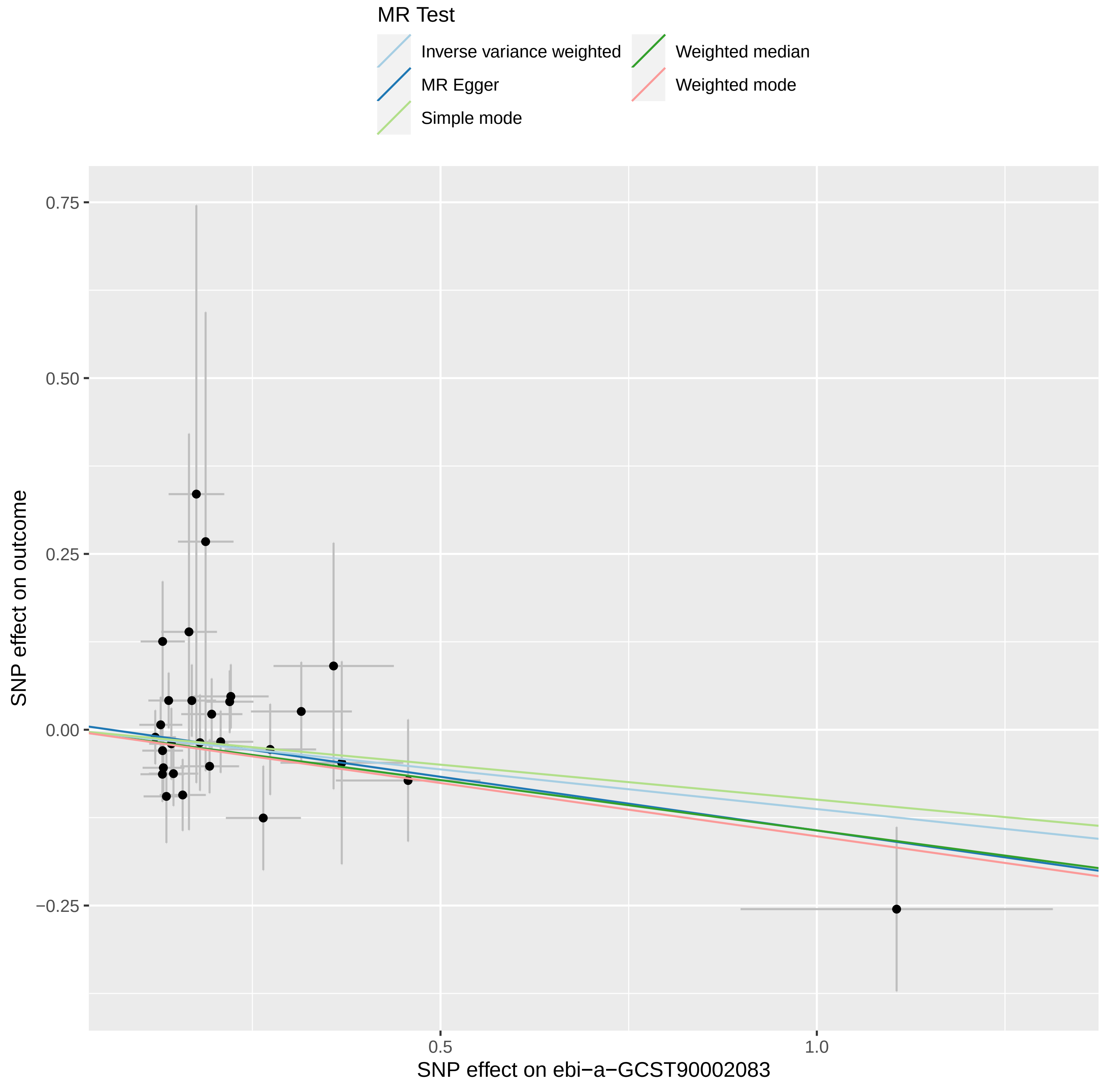
MR Test
Inverse variance weighted
MR Egger
Simple mode
Weighted median
Weighted mode
0.75
0.50
0.25
0.00
−0.25
SNP effect on outcome
0.5
1.0
SNP effect on ebi−a−GCST90002083

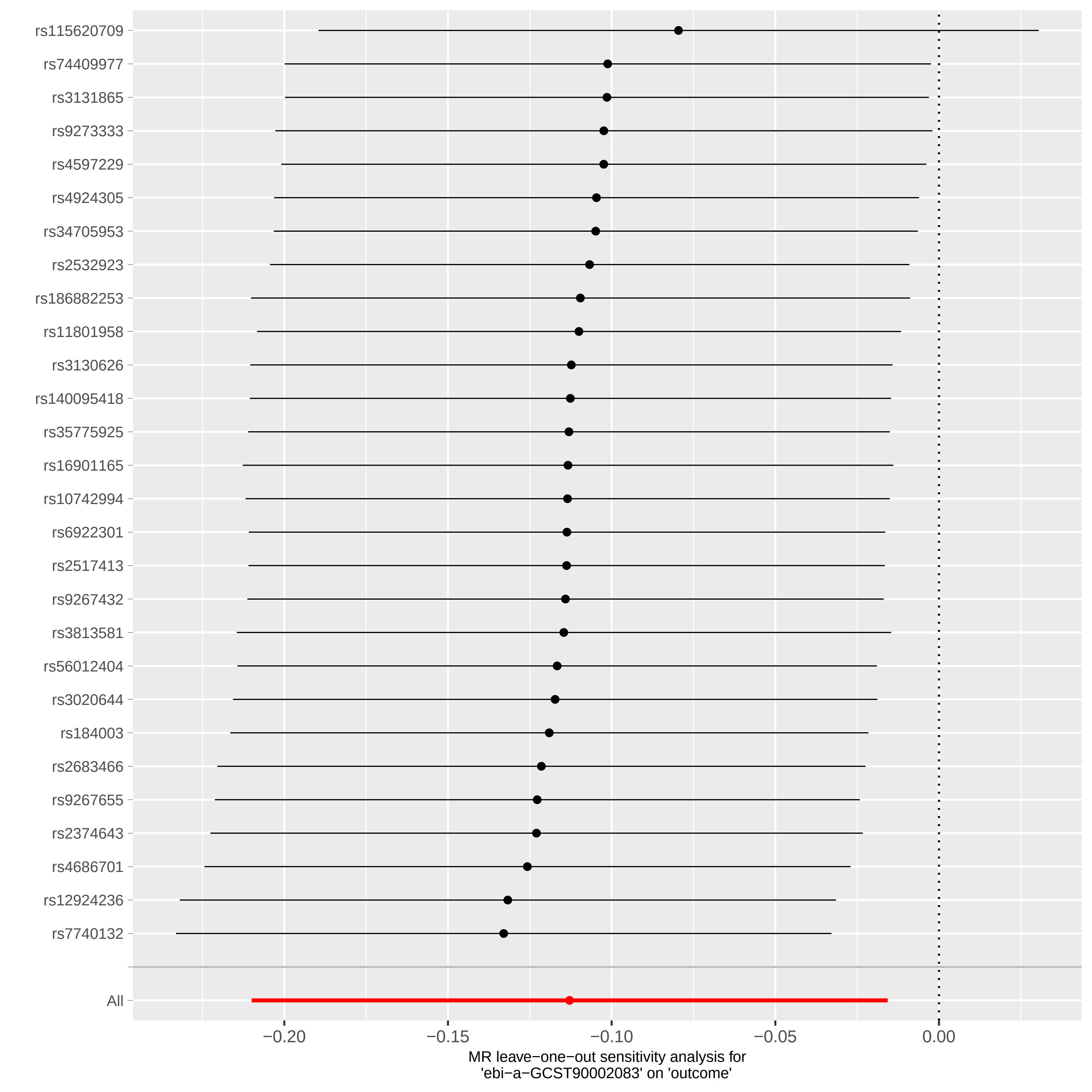
rs115620709
rs74409977
rs3131865
rs9273333
rs4597229
rs4924305
rs34705953
rs2532923
rs186882253
rs11801958
rs3130626
rs140095418
rs35775925
rs16901165
rs10742994
rs6922301
rs2517413
rs9267432
rs3813581
rs56012404
rs3020644
rs184003
rs2683466
rs9267655
rs2374643
rs4686701
rs12924236
rs7740132
All
−0.20
−0.15
−0.10
−0.05
0.00
MR leave−one−out sensitivity analysis for
'ebi−a−GCST90002083' on 'outcome'

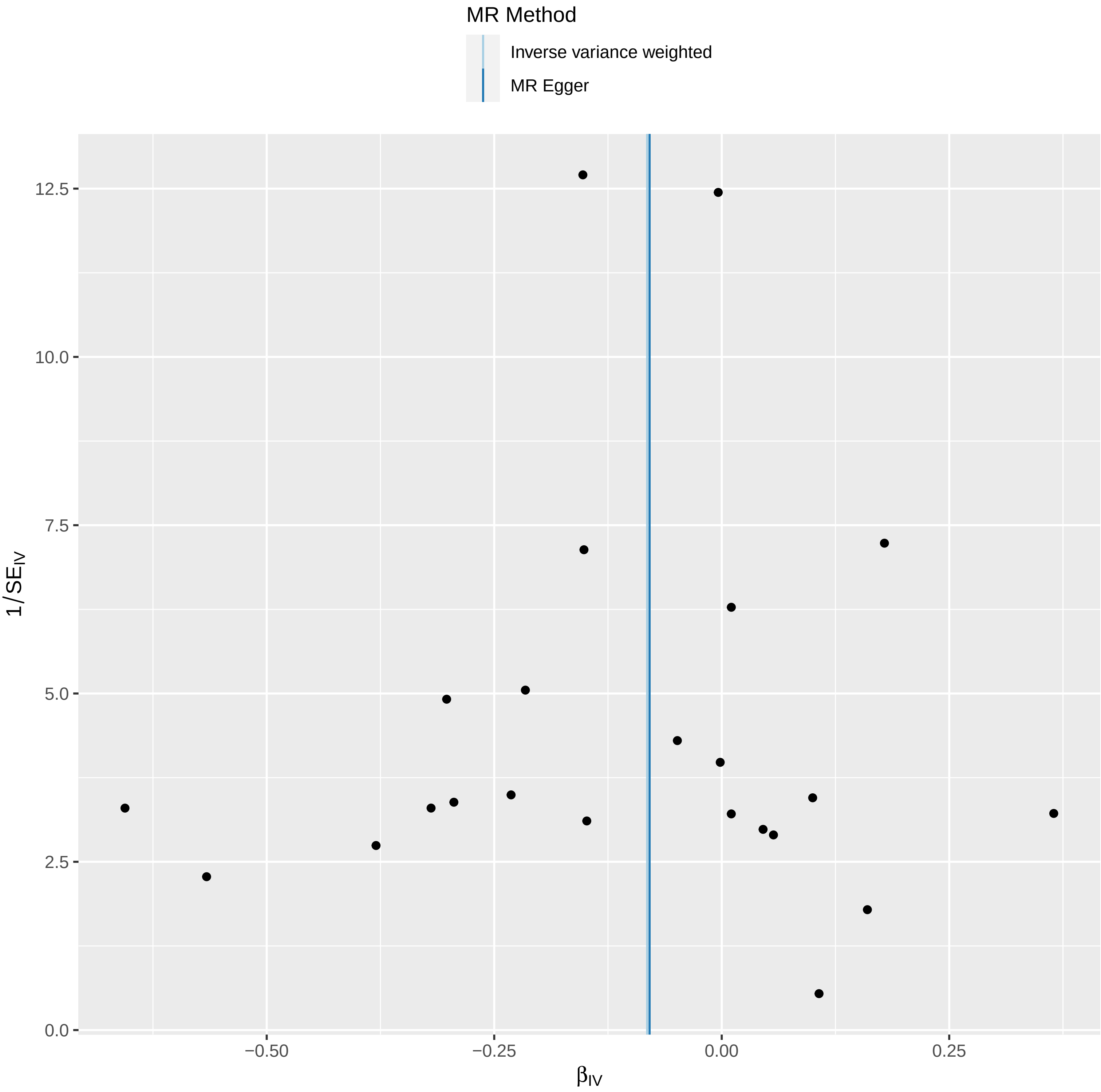
MR Method
Inverse variance weighted
MR Egger
12.5
10.0
7.5
5.0
2.5
0.0
$1/SE_{IV}$
−0.50
−0.25
0.00
0.25
β_{IV}

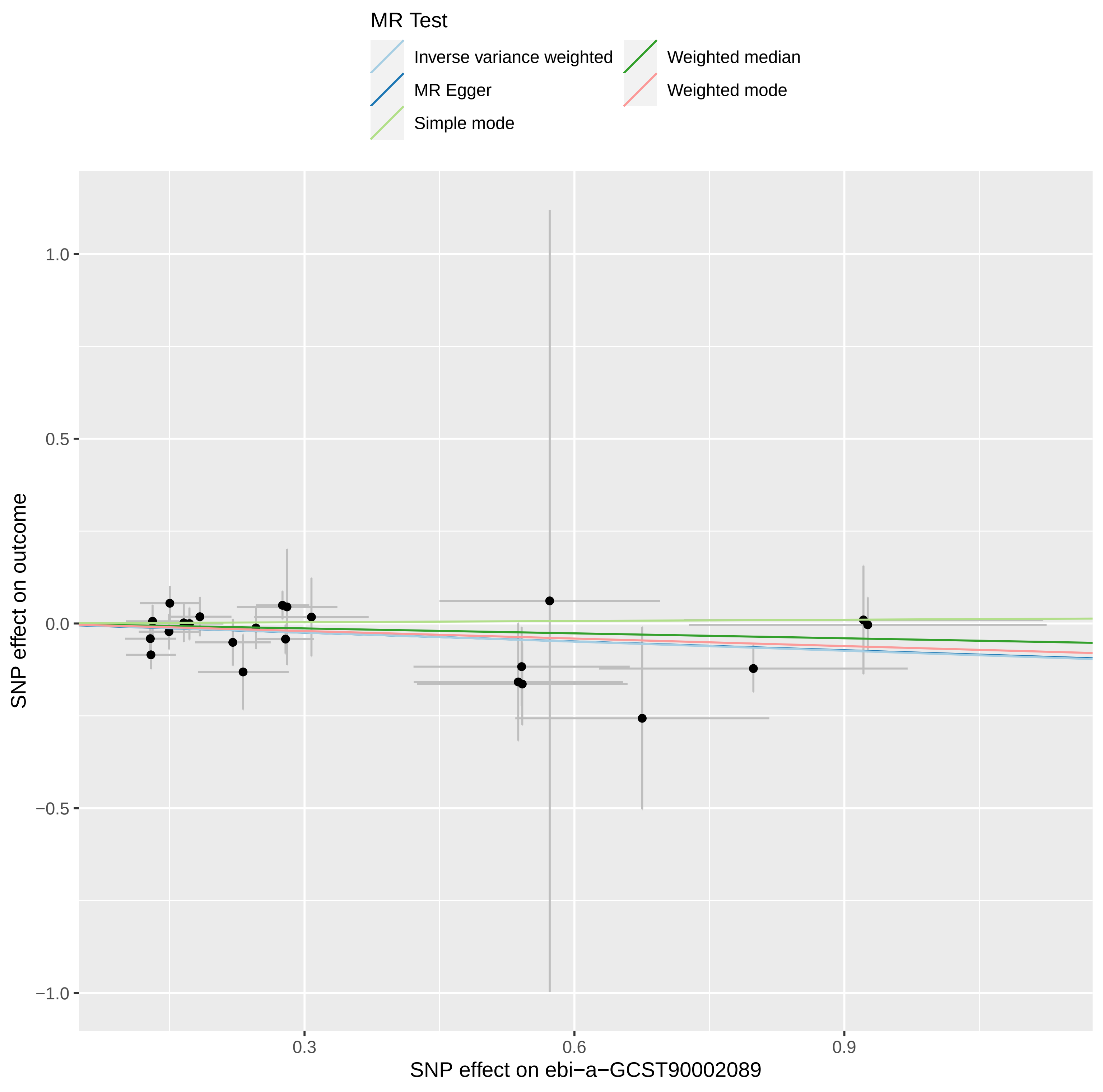
MR Test
Inverse variance weighted
MR Egger
Simple mode
Weighted median
Weighted mode
1.0
0.5
0.0
−0.5
−1.0
SNP effect on outcome
0.3
0.6
0.9
SNP effect on ebi−a−GCST90002089

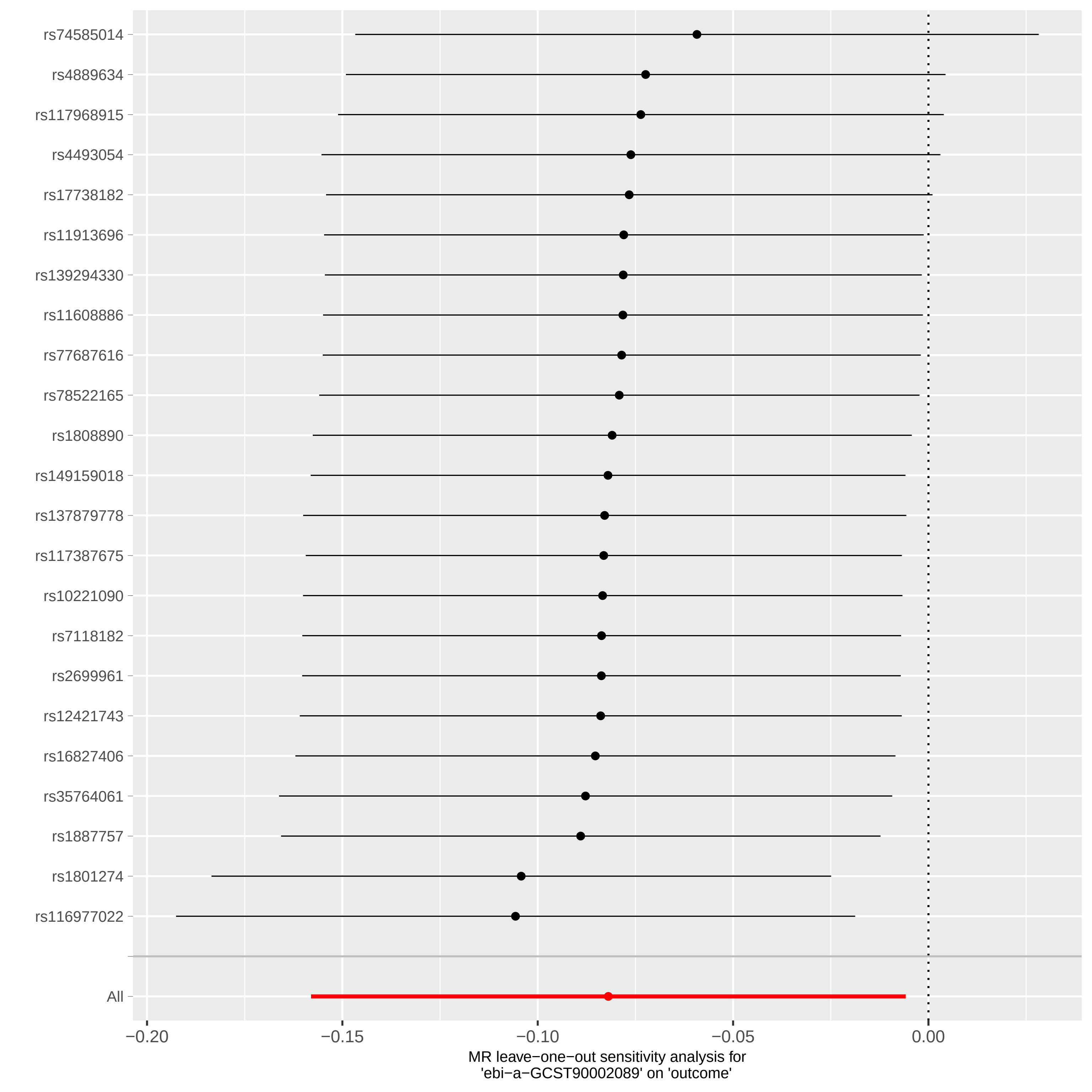
rs74585014
rs4889634
rs117968915
rs4493054
rs17738182
rs11913696
rs139294330
rs11608886
rs77687616
rs78522165
rs1808890
rs149159018
rs137879778
rs117387675
rs10221090
rs7118182
rs2699961
rs12421743
rs16827406
rs35764061
rs1887757
rs1801274
rs116977022
All
−0.20
−0.15
−0.10
−0.05
0.00
MR leave−one−out sensitivity analysis for
'ebi−a−GCST90002089' on 'outcome'

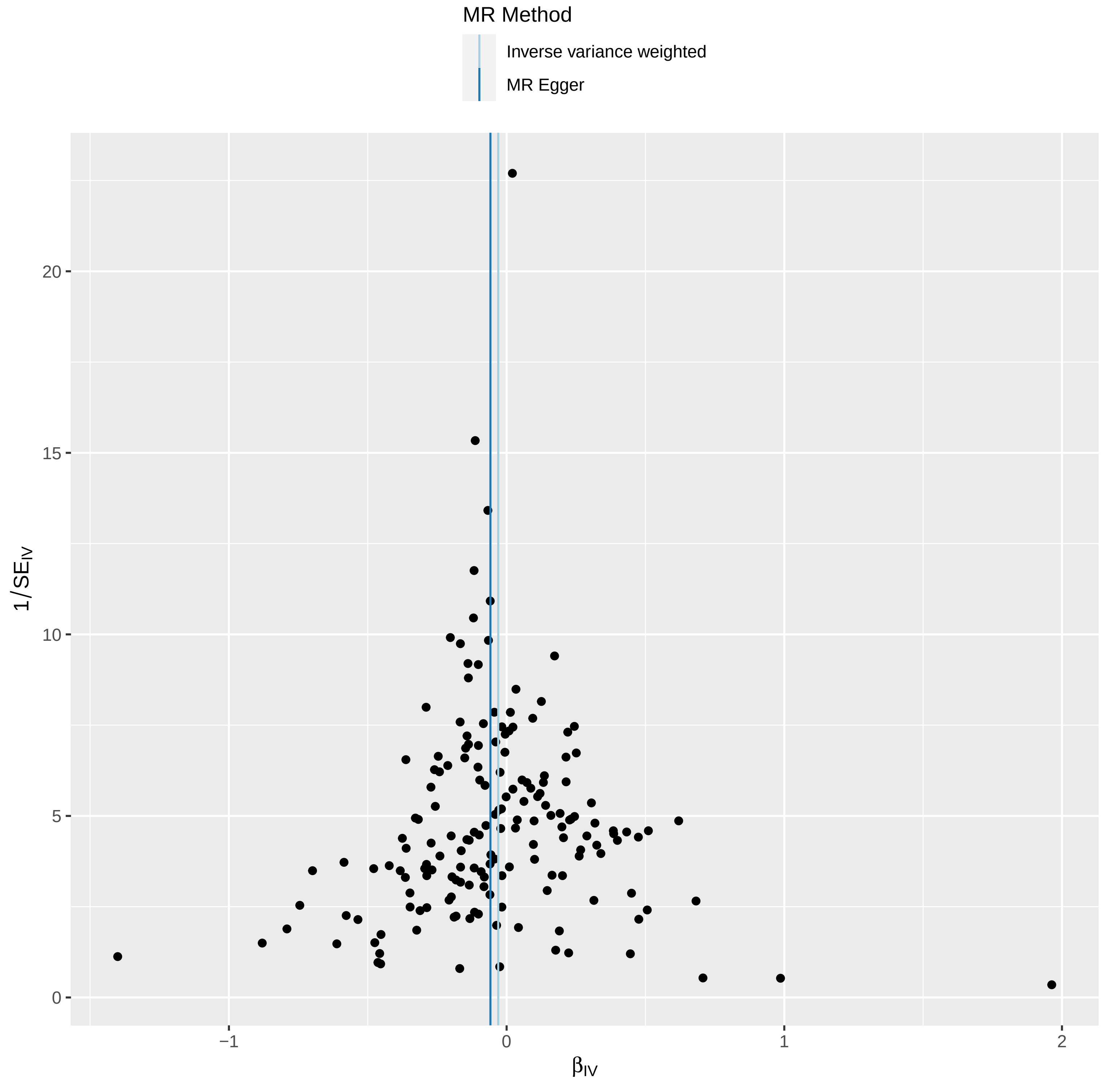
MR Method
Inverse variance weighted
MR Egger
1/SE_IV
20
15
10
5
0
−1
0
1
2
β_IV

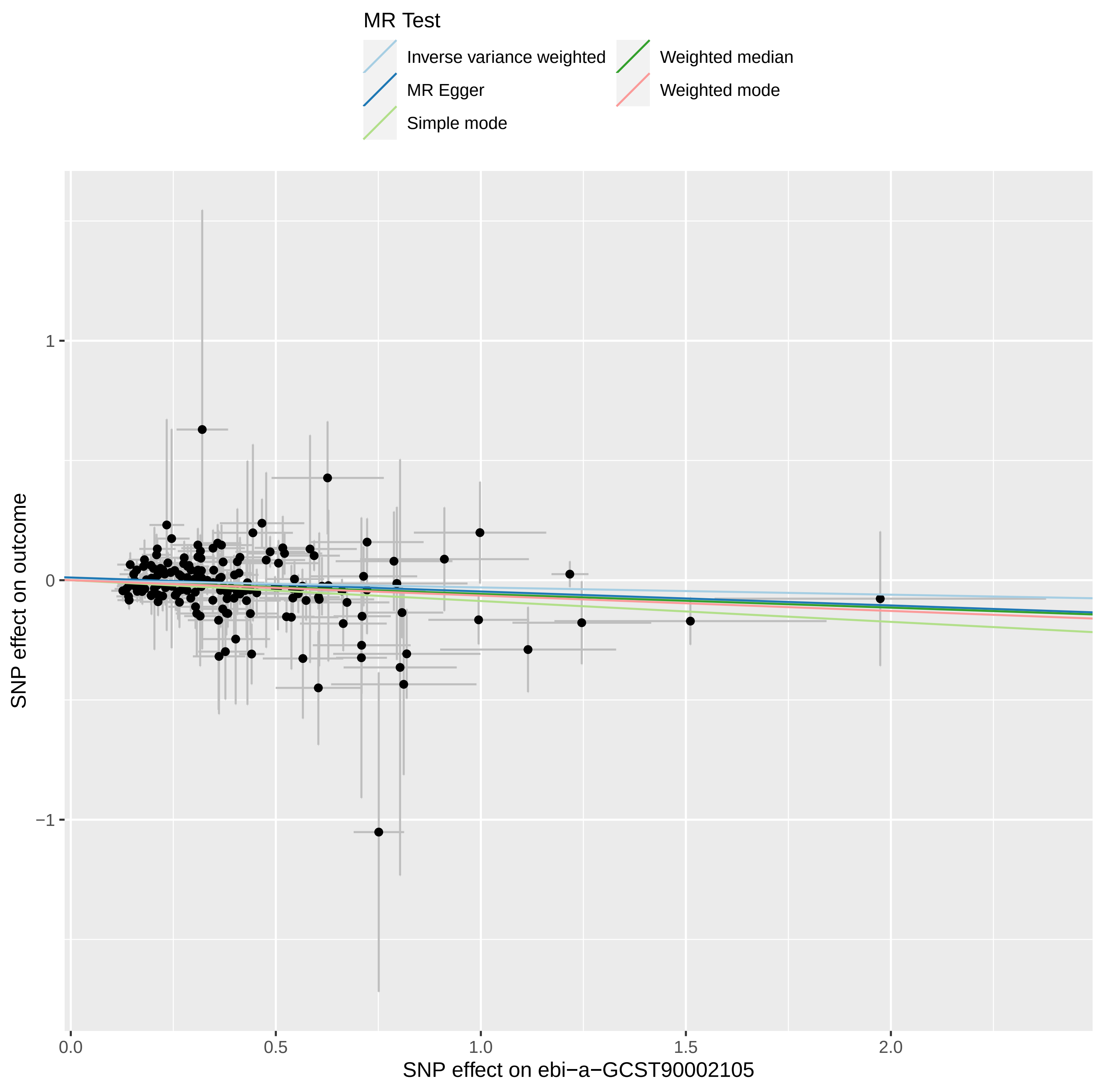

MR Test
Inverse variance weighted
MR Egger
Simple mode
Weighted median
Weighted mode
SNP effect on outcome
SNP effect on ebi−a−GCST90002105
1
0
−1
0.0
0.5
1.0
1.5
2.0

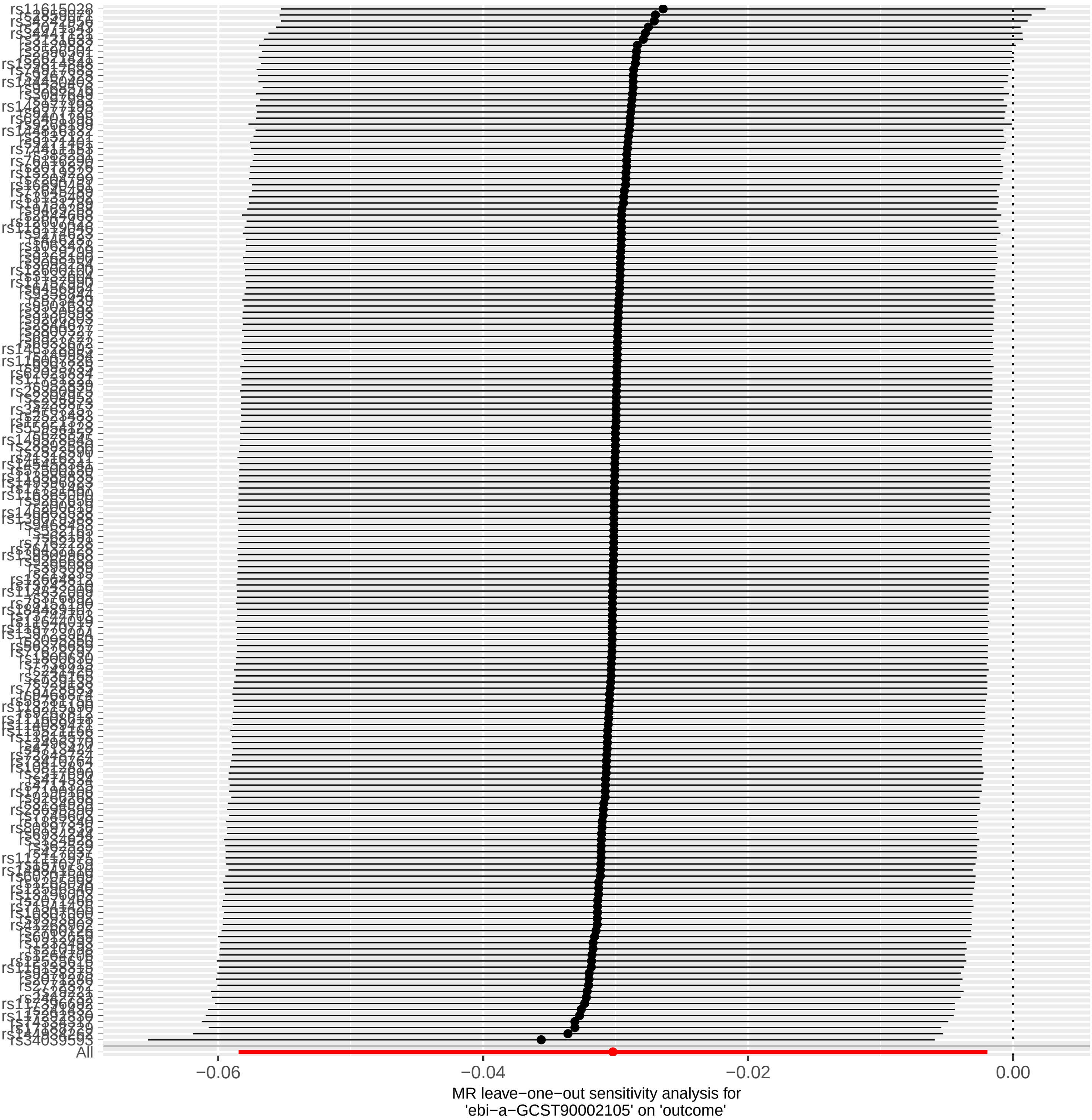
rs11615028
rs34039593
All
−0.06
−0.04
−0.02
0.00
MR leave−one−out sensitivity analysis for
'ebi−a−GCST90002105' on 'outcome'

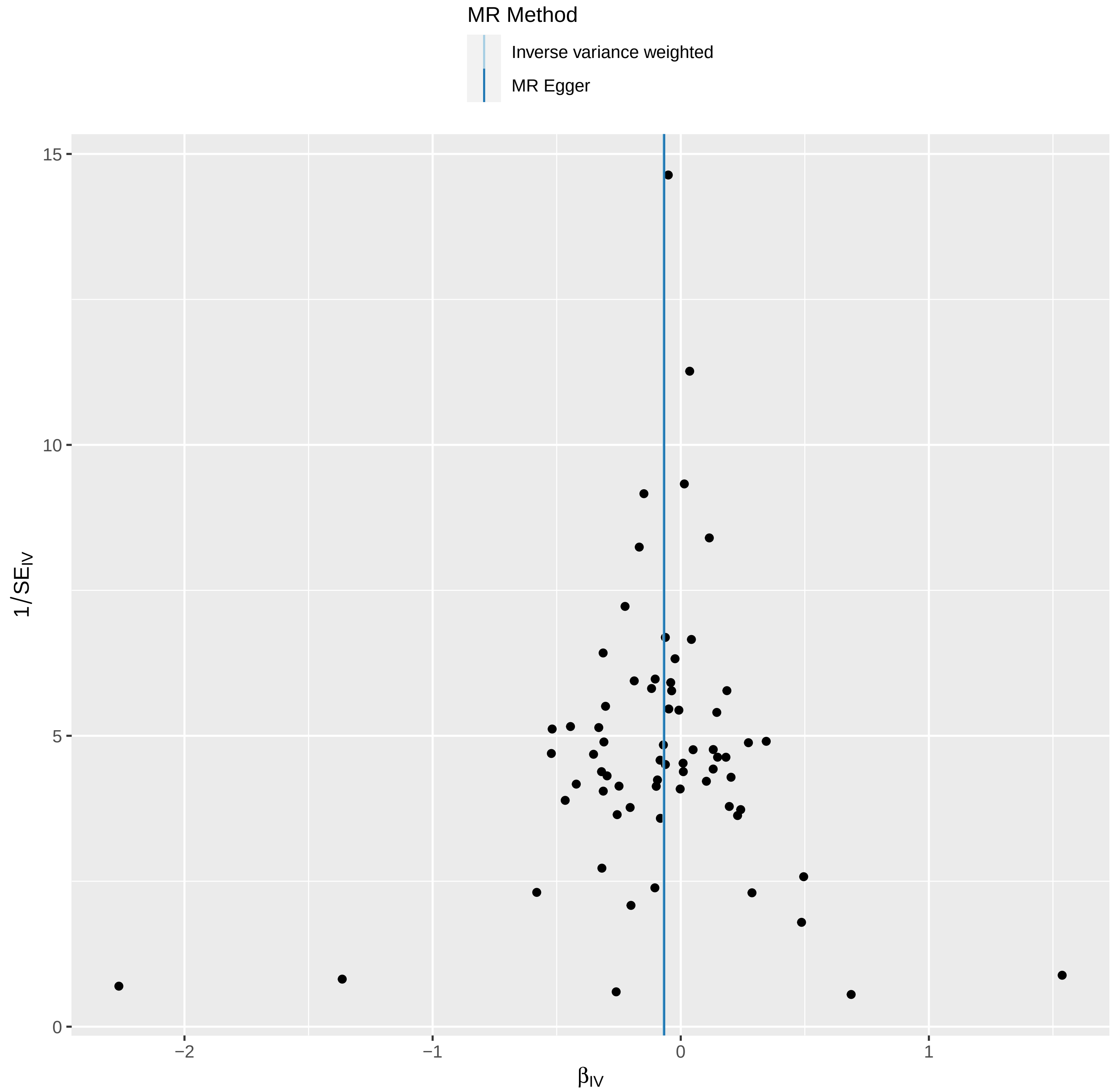

MR Method
Inverse variance weighted
MR Egger
15
10
5
0
−2
−1
0
1
1/SE_IV
β_IV

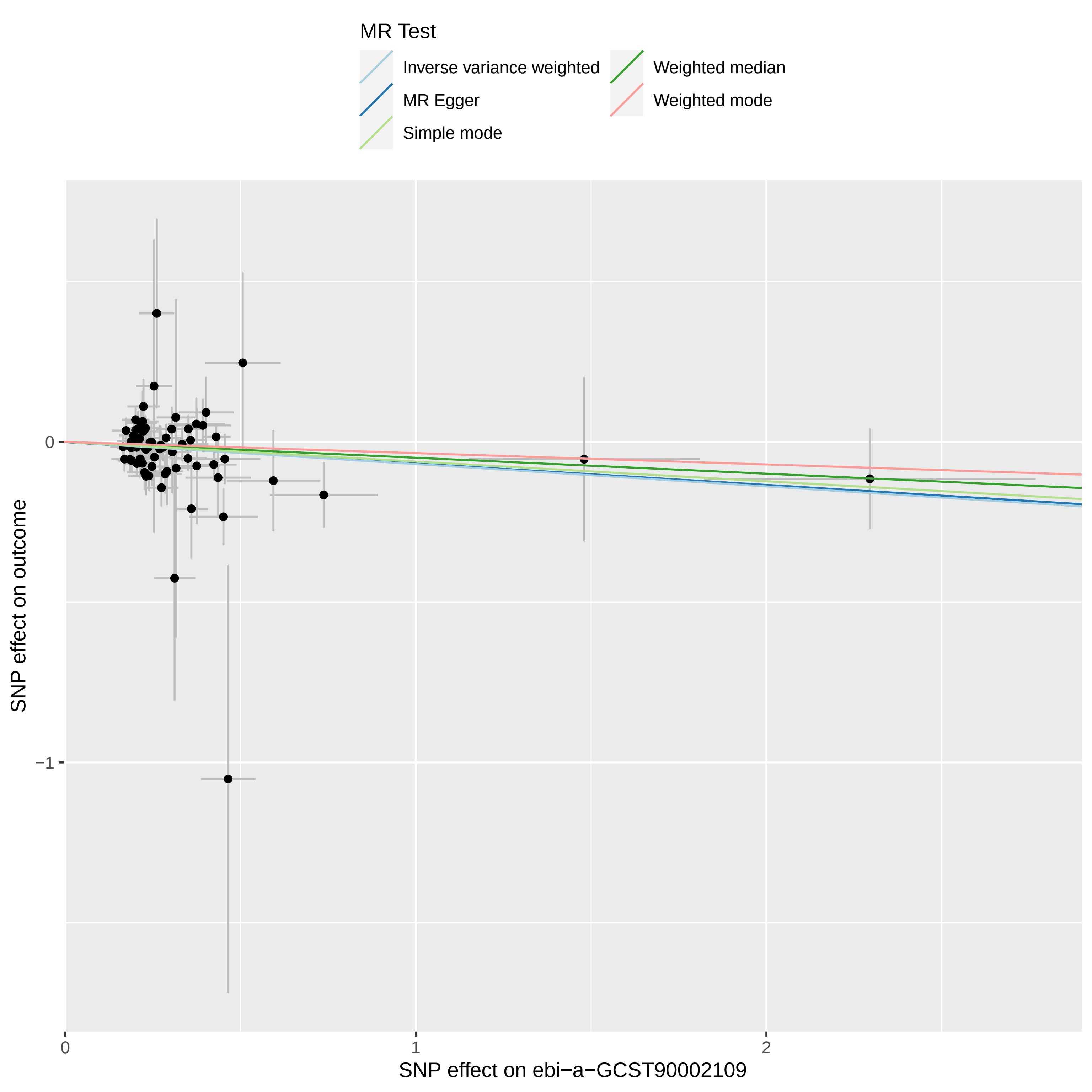

MR Test
Inverse variance weighted
MR Egger
Simple mode
Weighted median
Weighted mode
SNP effect on outcome
0
−1
0
1
2
SNP effect on ebi−a−GCST90002109

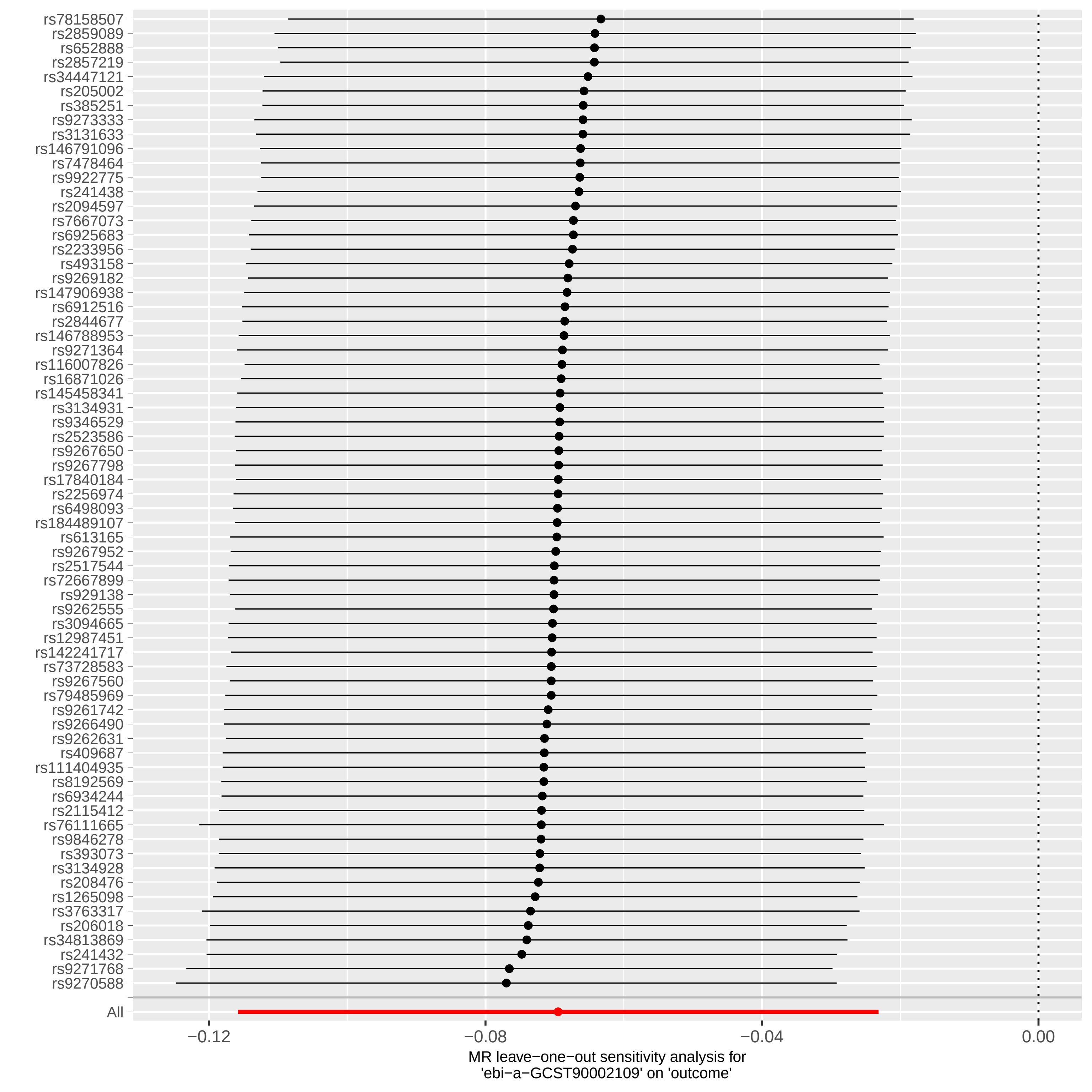

rs78158507
rs2859089
rs652888
rs2857219
rs34447121
rs205002
rs385251
rs9273333
rs3131633
rs146791096
rs7478464
rs9922775
rs241438
rs2094597
rs7667073
rs6925683
rs2233956
rs493158
rs9269182
rs147906938
rs6912516
rs2844677
rs146788953
rs9271364
rs116007826
rs16871026
rs145458341
rs3134931
rs9346529
rs2523586
rs9267650
rs9267798
rs17840184
rs2256974
rs6498093
rs184489107
rs613165
rs9267952
rs2517544
rs72667899
rs929138
rs9262555
rs3094665
rs12987451
rs142241717
rs73728583
rs9267560
rs79485969
rs9261742
rs9266490
rs9262631
rs409687
rs111404935
rs8192569
rs6934244
rs2115412
rs76111665
rs9846278
rs393073
rs3134928
rs208476
rs1265098
rs3763317
rs206018
rs34813869
rs241432
rs9271768
rs9270588
All
−0.12
−0.08
−0.04
0.00
MR leave−one−out sensitivity analysis for
'ebi−a−GCST90002109' on 'outcome'

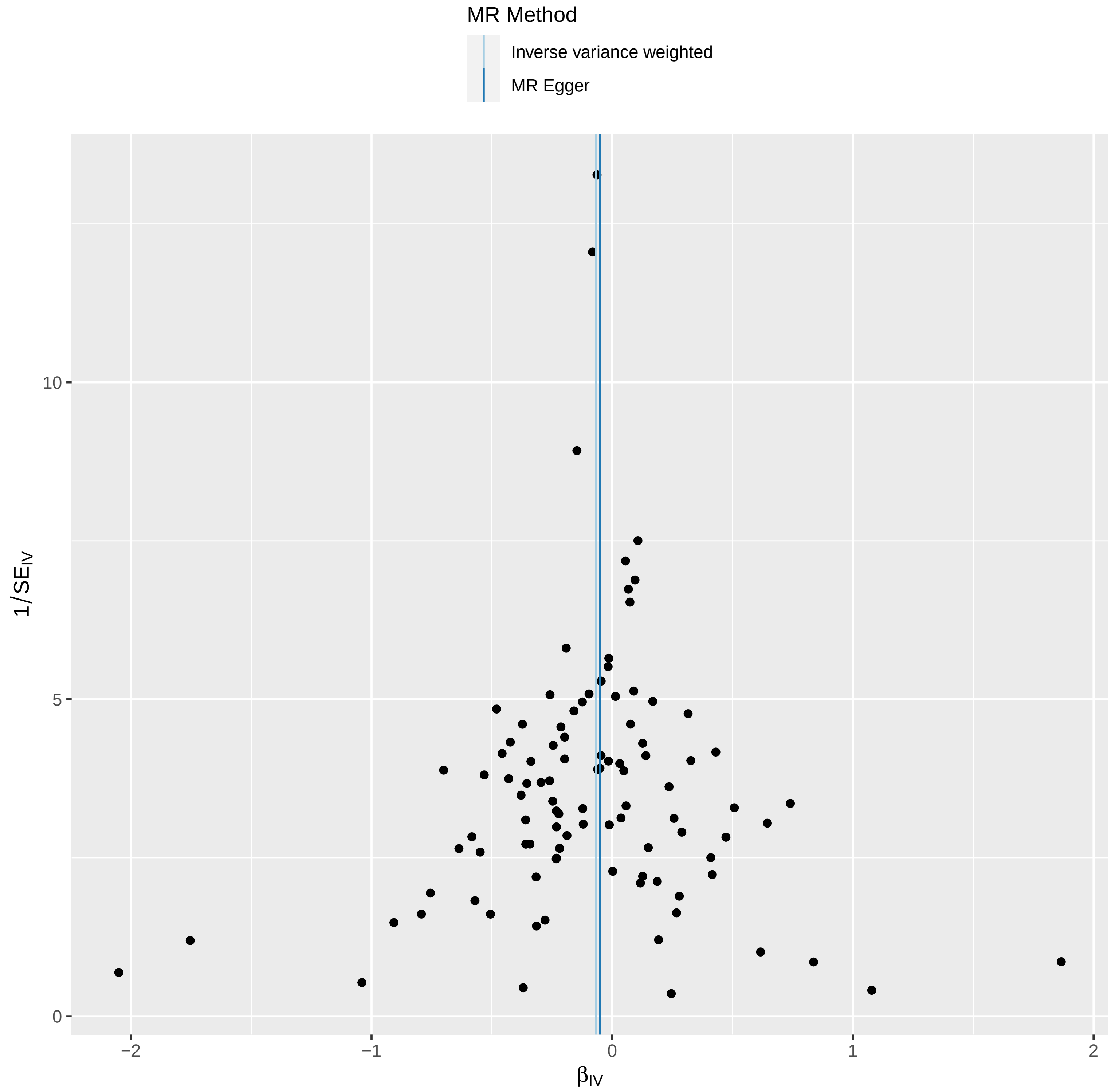

MR Method
Inverse variance weighted
MR Egger
1/SE_IV
10
5
0
β_IV
−2
−1
0
1
2

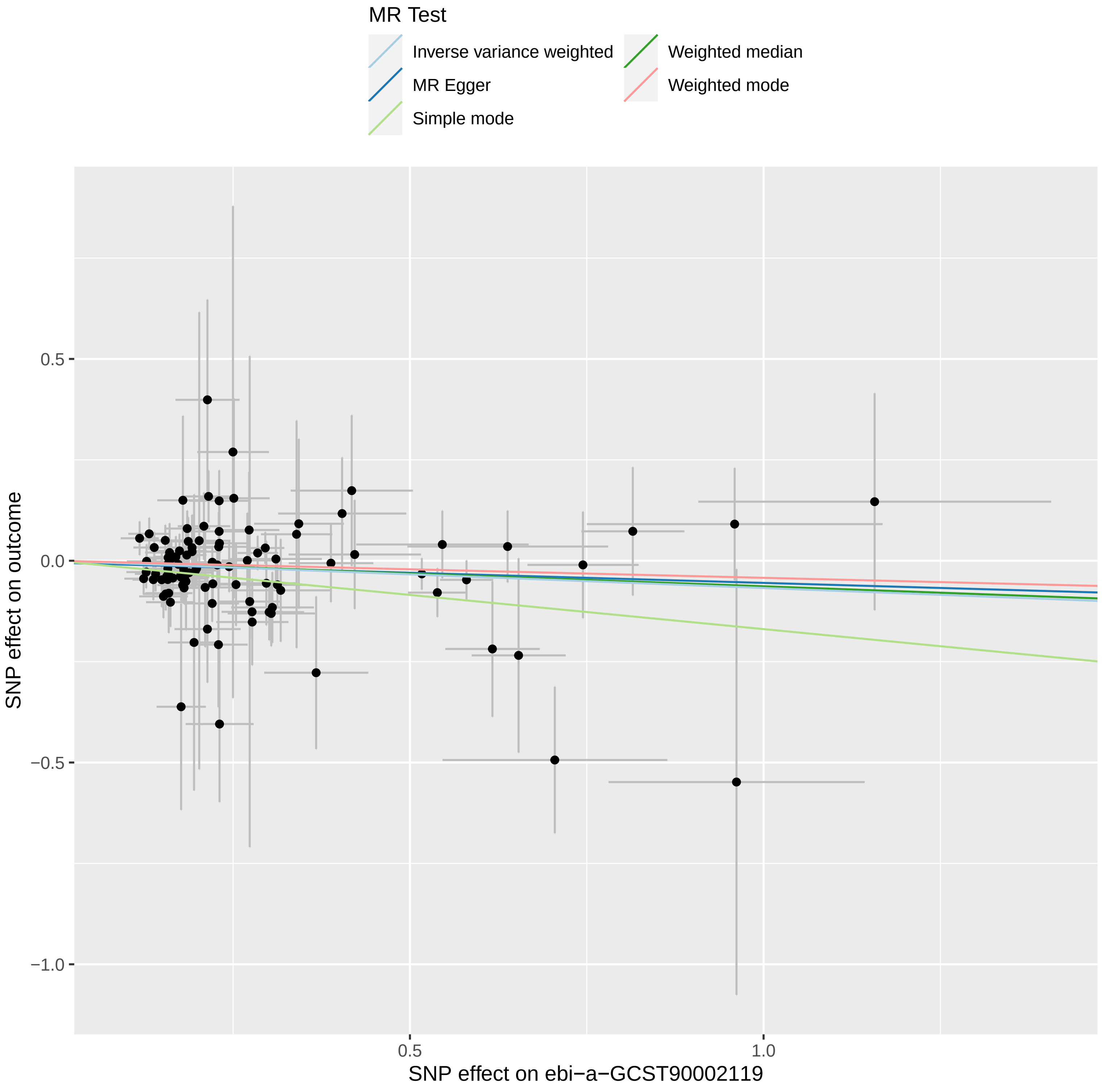
MR Test
Inverse variance weighted
MR Egger
Simple mode
Weighted median
Weighted mode
0.5
0.0
−0.5
−1.0
SNP effect on outcome
0.5
1.0
SNP effect on ebi−a−GCST90002119

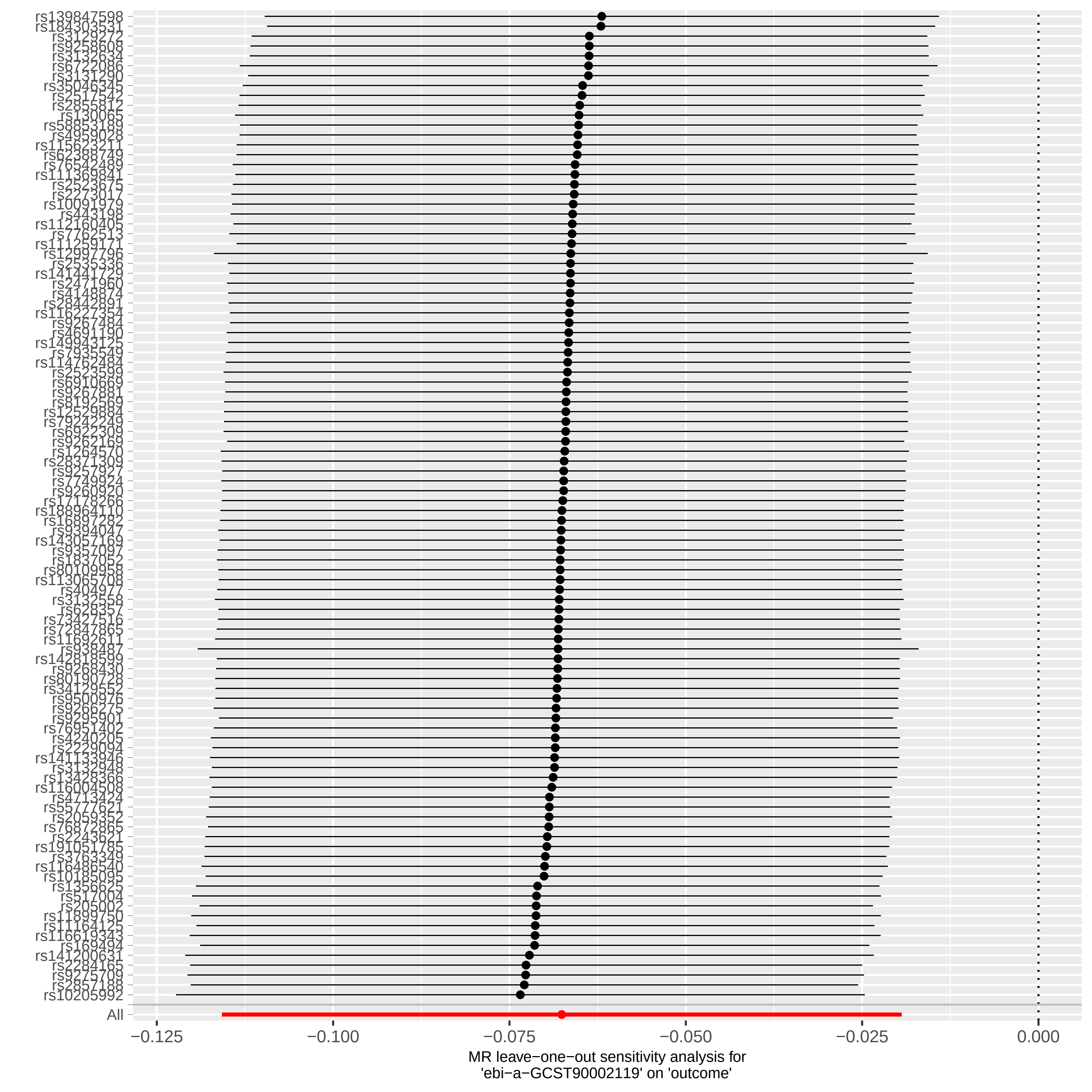
rs139847598
rs184303531
rs3129272
rs9258608
rs3132634
rs6722086
rs3131290
rs35046345
rs2517542
rs2855812
rs130065
rs58853189
rs4959028
rs115623211
rs62388749
rs76542489
rs111369841
rs2523675
rs2273017
rs10091979
rs443198
rs112160405
rs7762513
rs111259171
rs12997796
rs2535336
rs141441729
rs2471960
rs4148874
rs28442891
rs116227354
rs9267484
rs4691190
rs149943125
rs7935549
rs114762484
rs2523599
rs6910669
rs9267881
rs8192569
rs12529884
rs79242249
rs6922309
rs9262169
rs1264570
rs28371309
rs9257927
rs7749924
rs9260920
rs17178266
rs188964110
rs16897282
rs9394047
rs143057169
rs9357097
rs1837052
rs80109958
rs113065708
rs404977
rs3132558
rs629357
rs73427516
rs72847865
rs11692611
rs938487
rs142818599
rs9268430
rs80190728
rs34129552
rs9500976
rs9266275
rs9295901
rs76951402
rs4240205
rs2229094
rs141133946
rs3132948
rs13428366
rs116004508
rs4713424
rs55777621
rs2059352
rs76872865
rs2243621
rs191051785
rs3763349
rs116486540
rs10185095
rs1356625
rs517004
rs205002
rs11899750
rs11164125
rs116619343
rs169494
rs141200631
rs2284165
rs9275709
rs2857188
rs10205992
All
−0.125
−0.100
−0.075
−0.050
−0.025
0.000
MR leave−one−out sensitivity analysis for
'ebi−a−GCST90002119' on 'outcome'
