# Supplementary material for: Identifying the causal relationship between immune factors and osteonecrosis: a two-sample Mendelian randomization study
Source: Sci Rep. 2024 Apr 23;14:9371. doi: 10.1038/s41598-024-59810-0 (PMC11039661; doi:10.1038/s41598-024-59810-0)
Supplement: Supplementary file 12 — Supplementary Information 12. [file 41598_2024_59810_MOESM12_ESM.pdf]

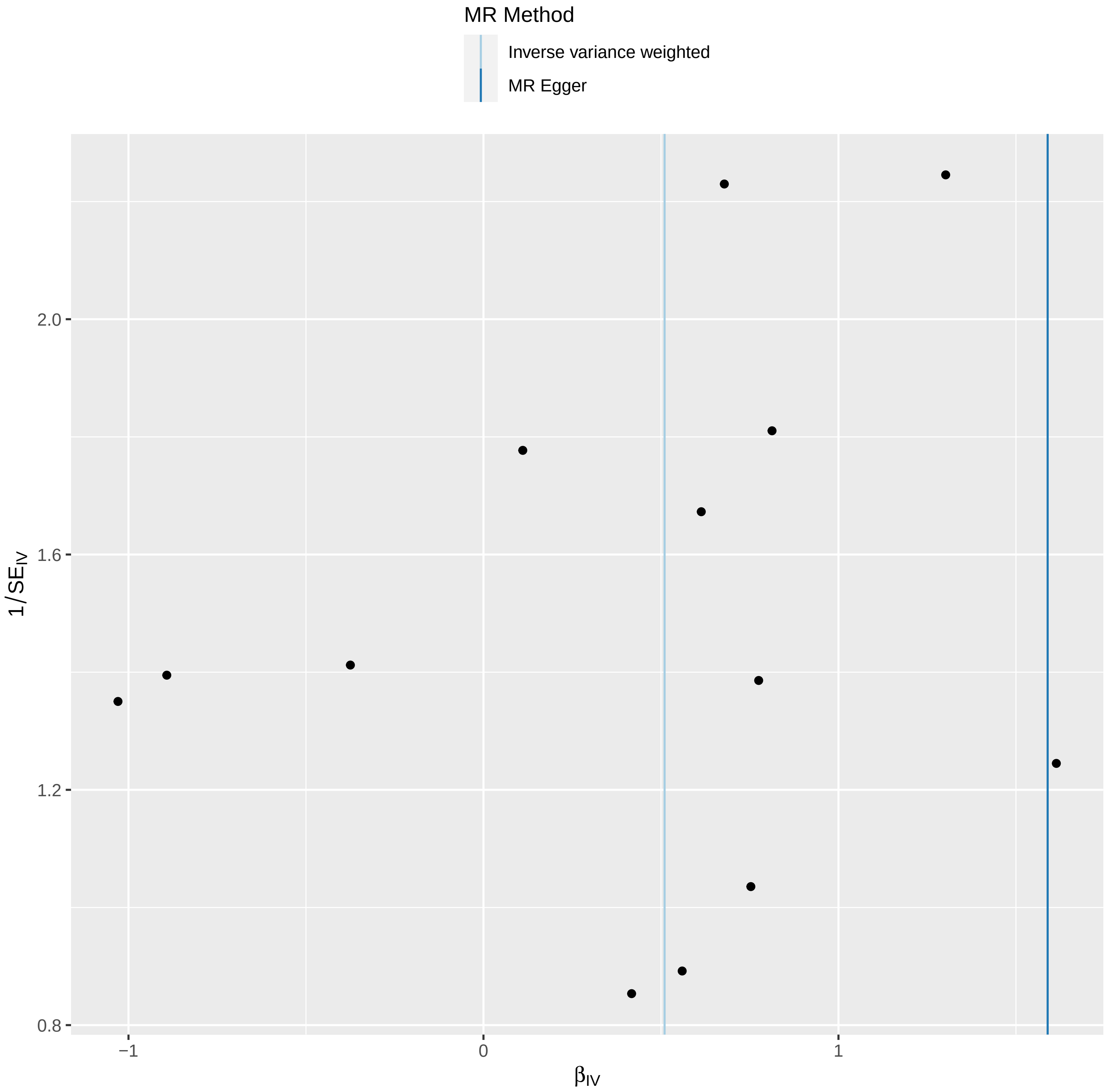

MR Method
Inverse variance weighted
MR Egger
2.0
1.6
1.2
0.8
1/SE_IV
−1
0
1
β_IV

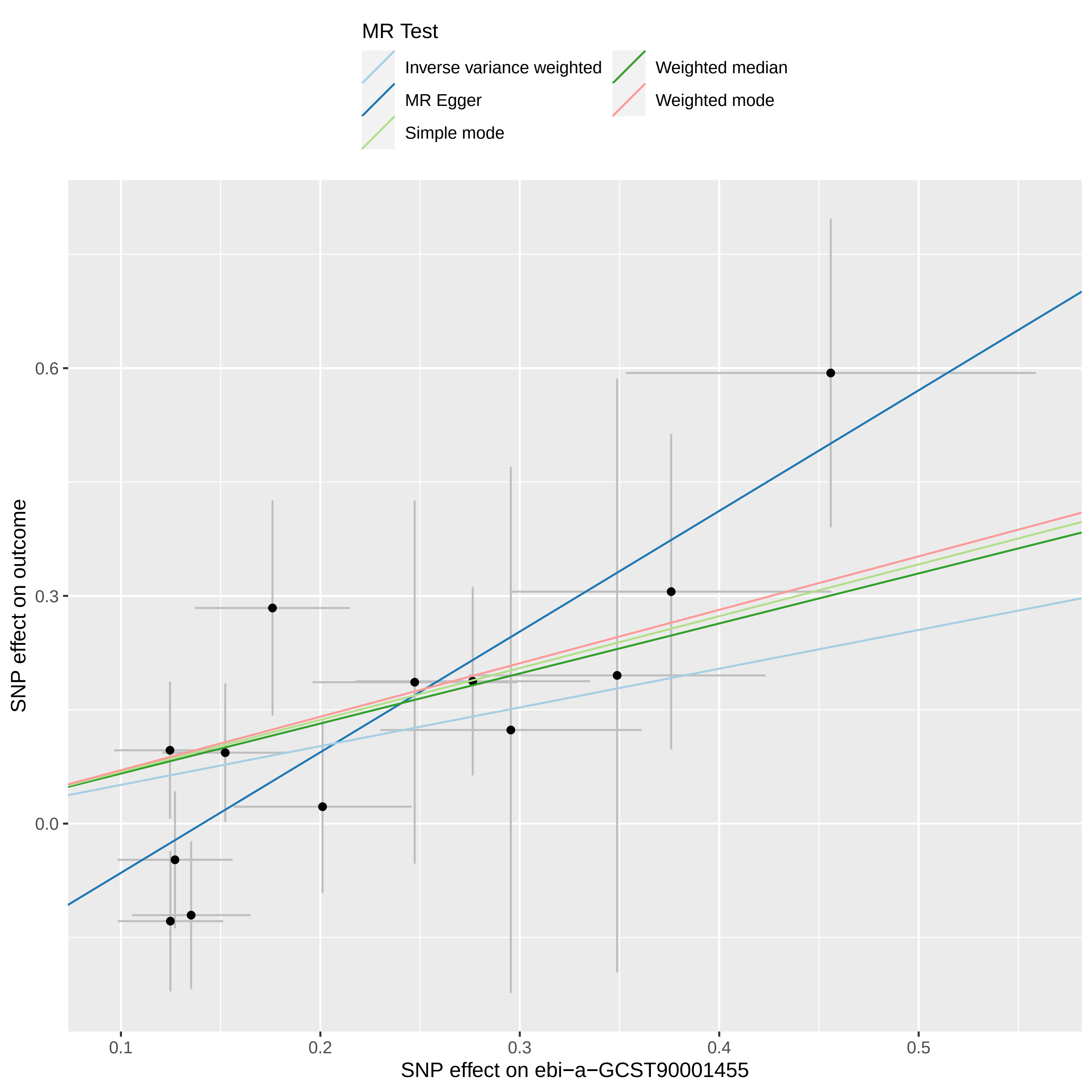
MR Test
Inverse variance weighted
MR Egger
Simple mode
Weighted median
Weighted mode
SNP effect on outcome
0.6
0.3
0.0
0.1
0.2
0.3
0.4
0.5
SNP effect on ebi−a−GCST90001455

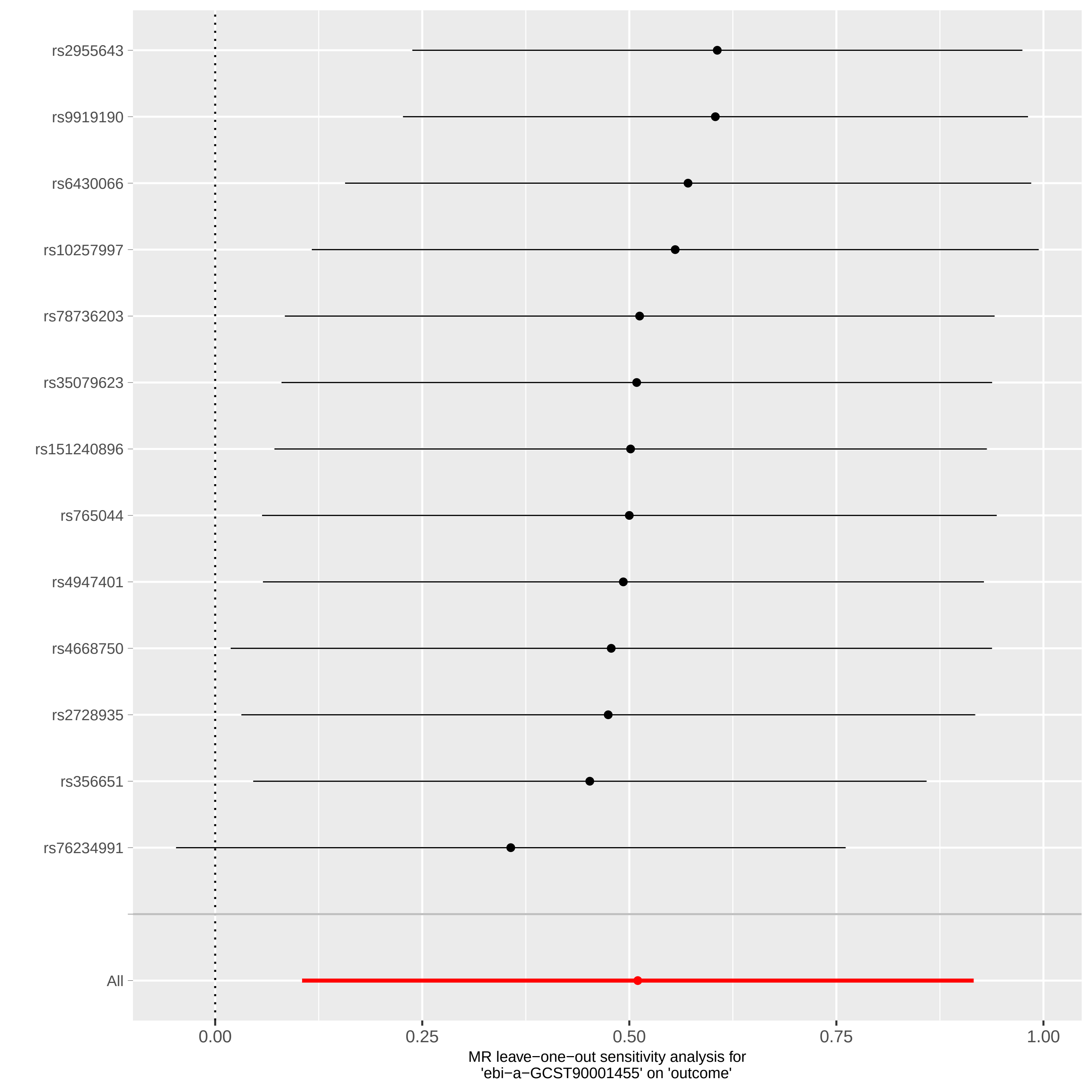
rs2955643
rs9919190
rs6430066
rs10257997
rs78736203
rs35079623
rs151240896
rs765044
rs4947401
rs4668750
rs2728935
rs356651
rs76234991
All
0.00
0.25
0.50
0.75
1.00
MR leave−one−out sensitivity analysis for
'ebi−a−GCST90001455' on 'outcome'

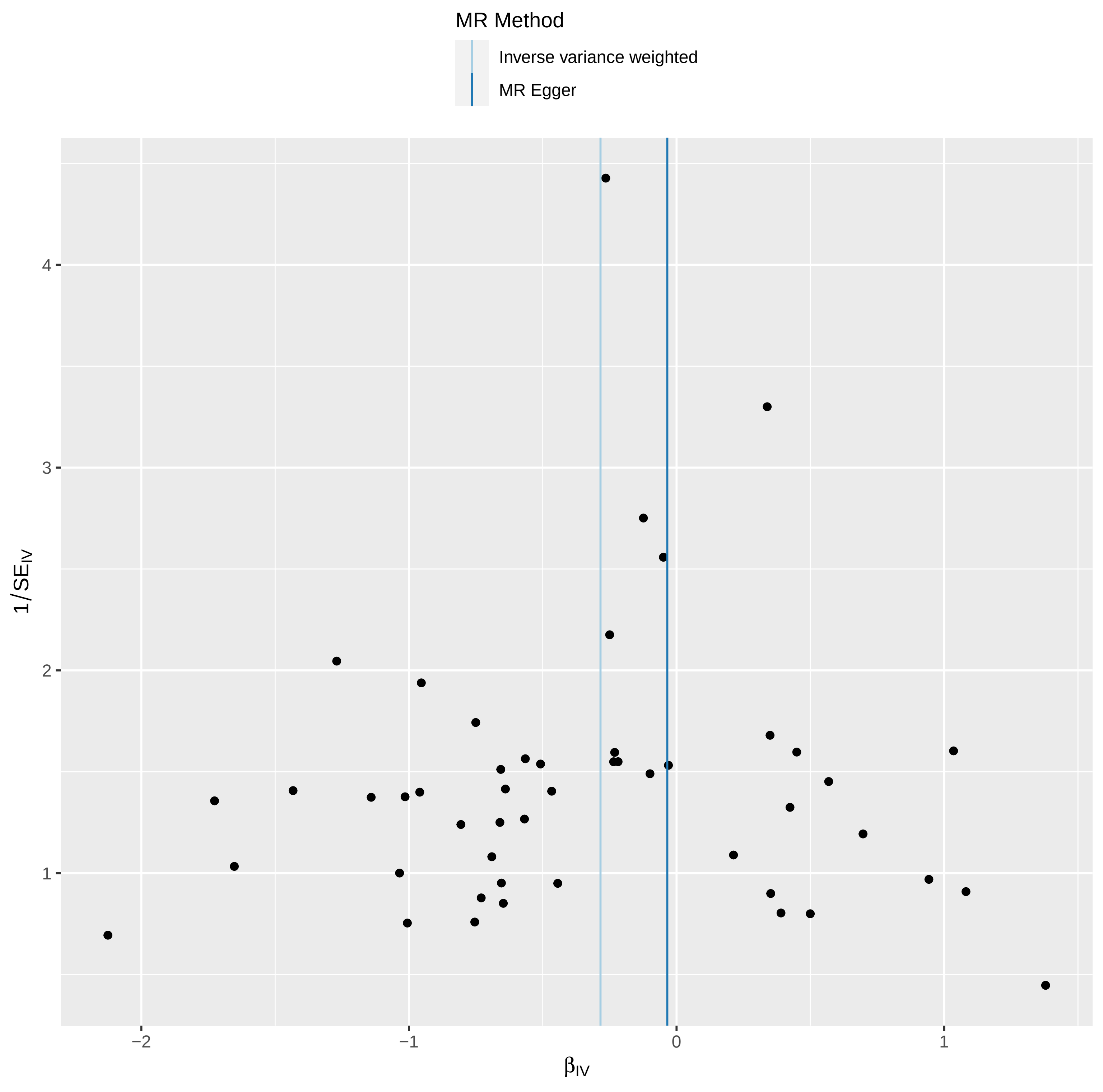

MR Method
Inverse variance weighted
MR Egger
4
3
2
1
1/SE$_{IV}$
−2
−1
0
1
β_{IV}

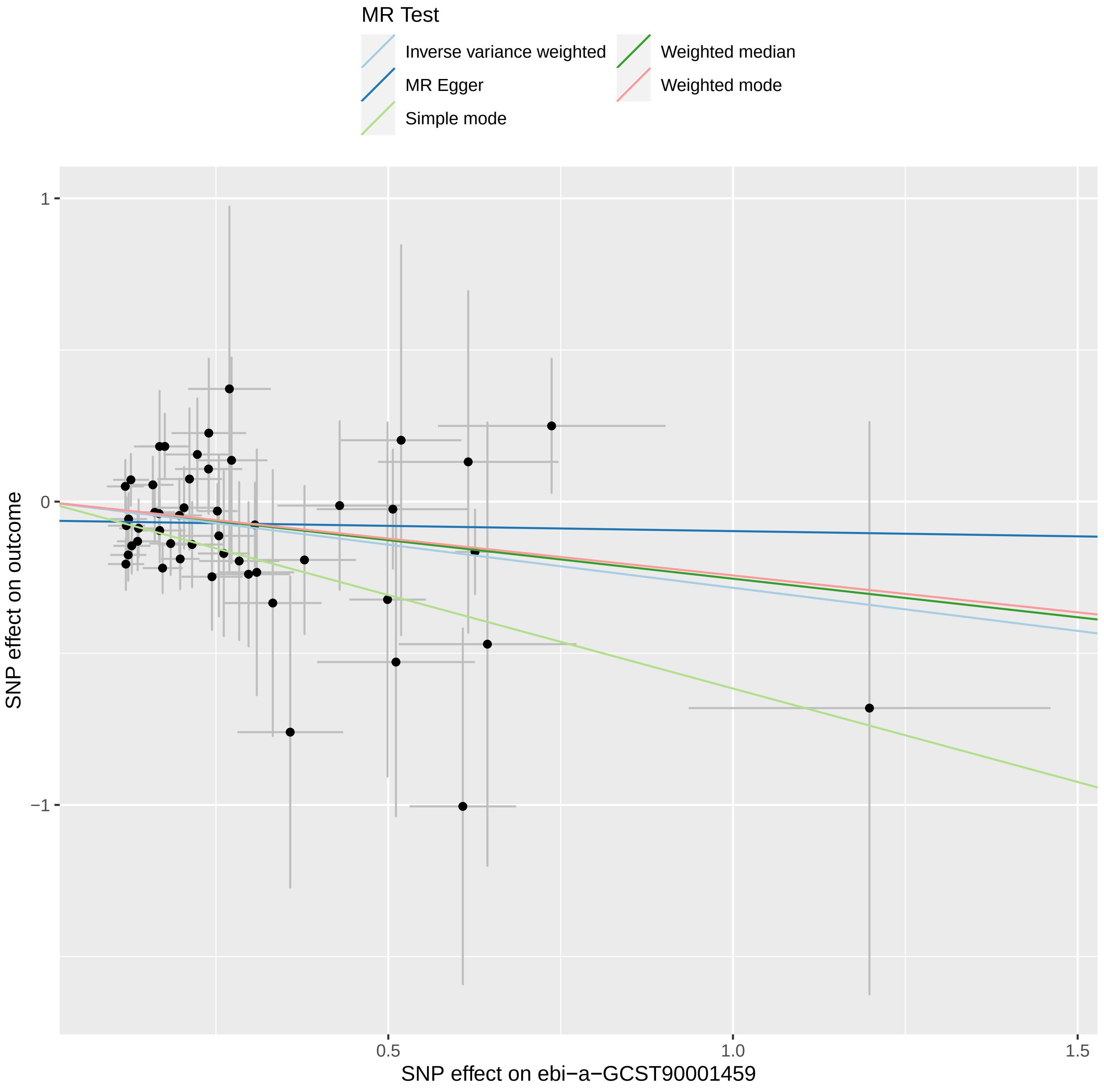
MR Test
Inverse variance weighted
MR Egger
Simple mode
Weighted median
Weighted mode
SNP effect on outcome
1
0
−1
0.5
1.0
1.5
SNP effect on ebi−a−GCST90001459

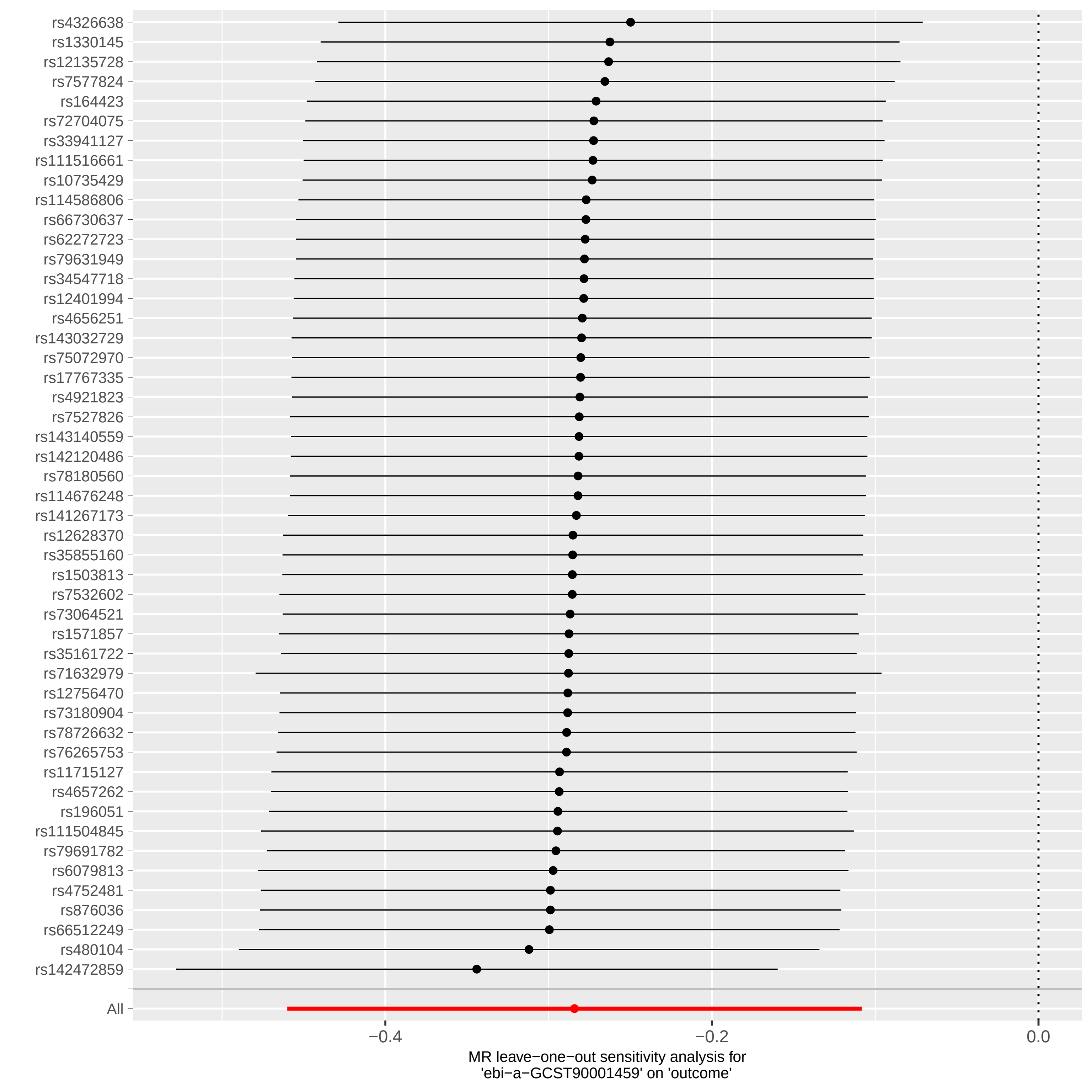

rs4326638
rs1330145
rs12135728
rs7577824
rs164423
rs72704075
rs33941127
rs111516661
rs10735429
rs114586806
rs66730637
rs62272723
rs79631949
rs34547718
rs12401994
rs4656251
rs143032729
rs75072970
rs17767335
rs4921823
rs7527826
rs143140559
rs142120486
rs78180560
rs114676248
rs141267173
rs12628370
rs35855160
rs1503813
rs7532602
rs73064521
rs1571857
rs35161722
rs71632979
rs12756470
rs73180904
rs78726632
rs76265753
rs11715127
rs4657262
rs196051
rs111504845
rs79691782
rs6079813
rs4752481
rs876036
rs66512249
rs480104
rs142472859
All
−0.4
−0.2
0.0
MR leave−one−out sensitivity analysis for
'ebi−a−GCST90001459' on 'outcome'

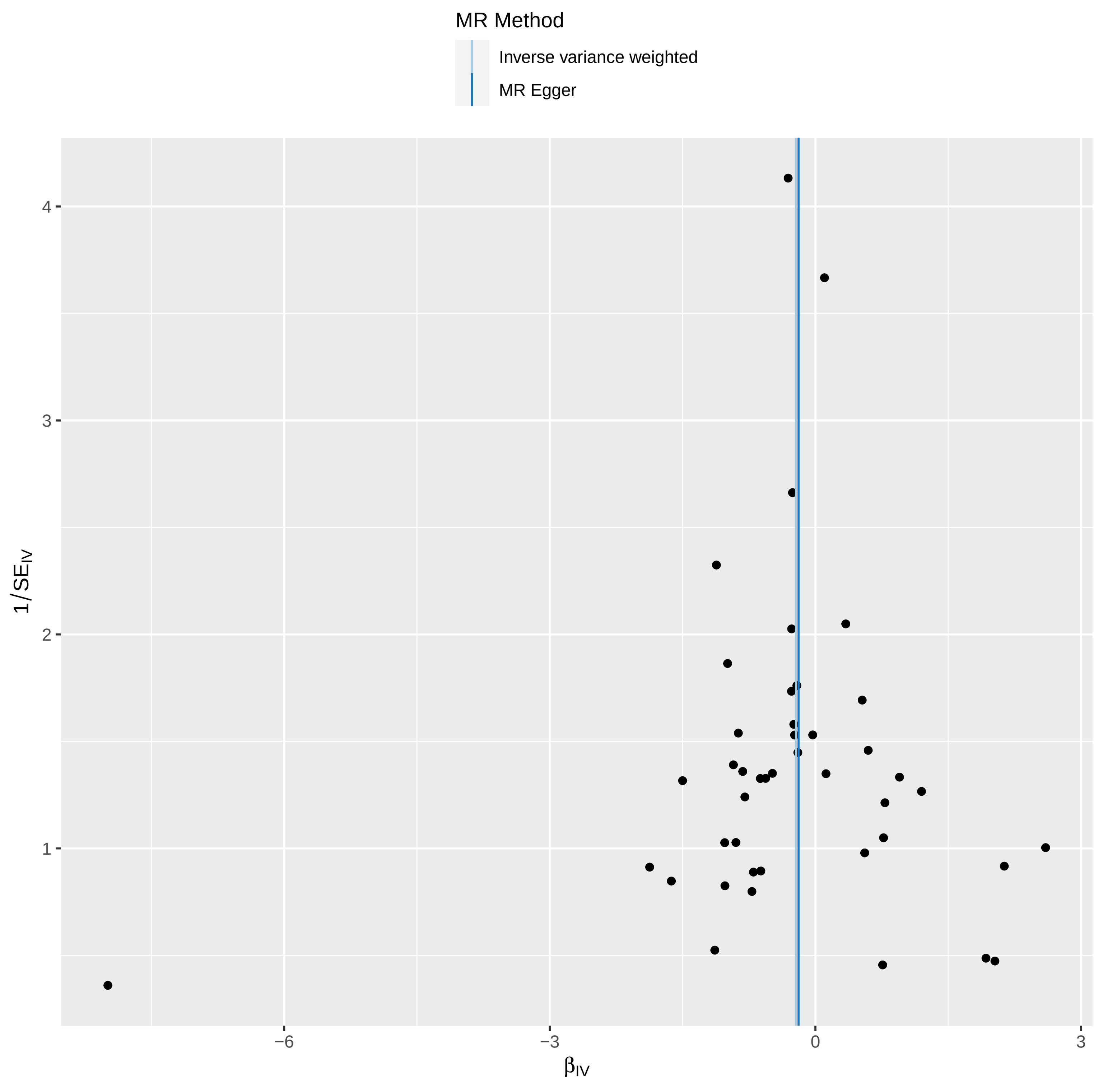

MR Method
Inverse variance weighted
MR Egger
4
3
2
1
$1/SE_{IV}$
−6
−3
0
3
β_{IV}

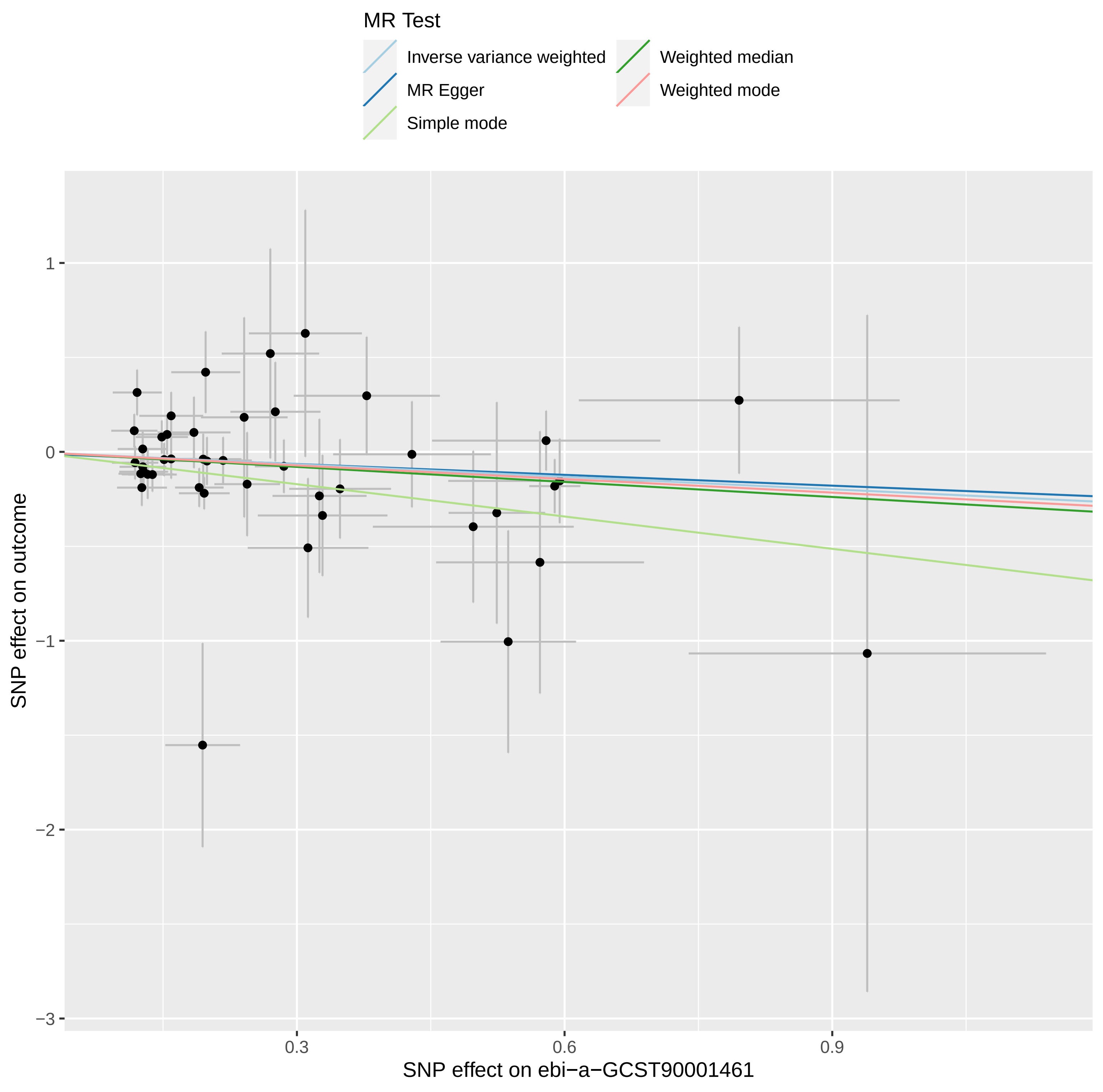

MR Test
Inverse variance weighted
MR Egger
Simple mode
Weighted median
Weighted mode
1
0
−1
−2
−3
0.3
0.6
0.9
SNP effect on outcome
SNP effect on ebi−a−GCST90001461

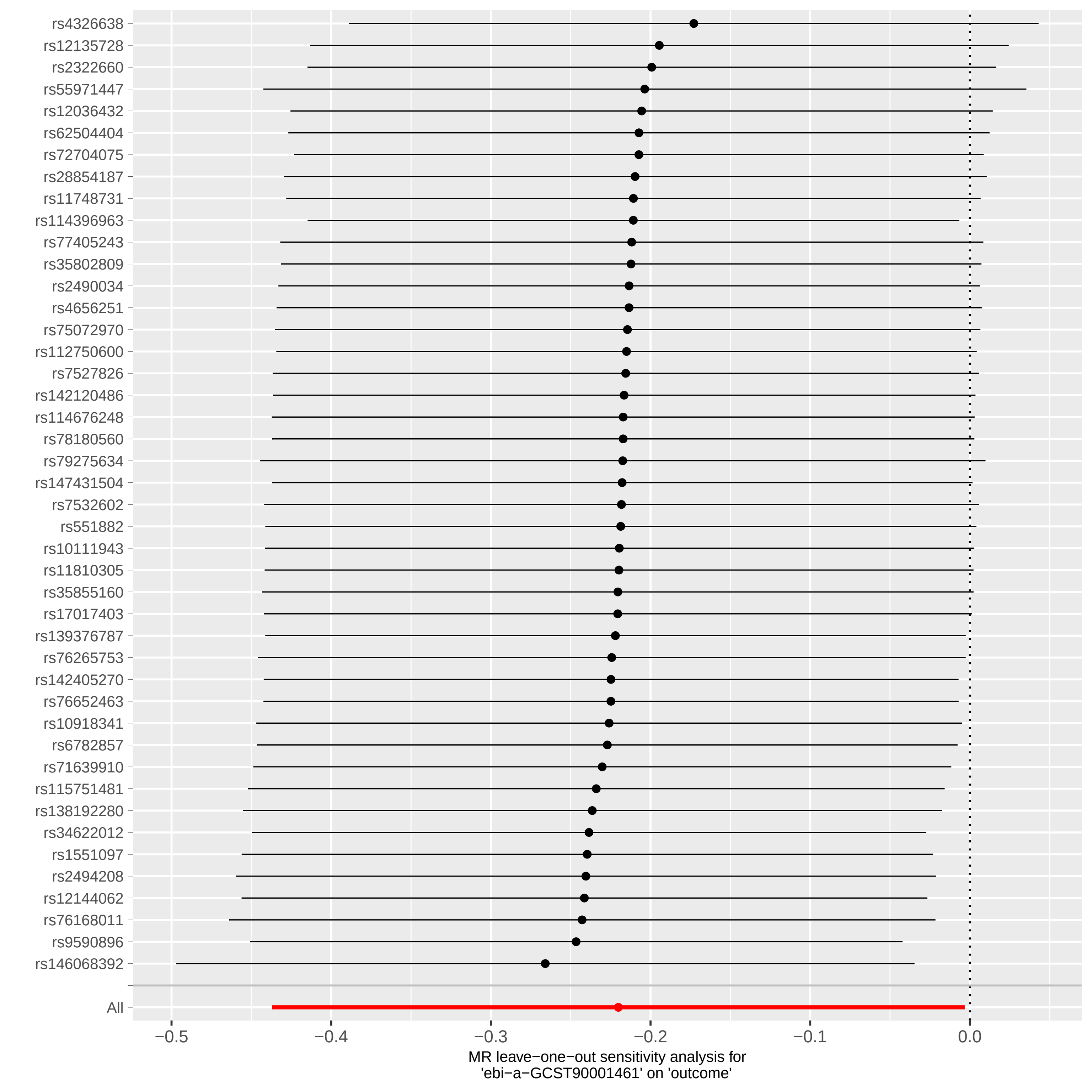

rs4326638
rs12135728
rs2322660
rs55971447
rs12036432
rs62504404
rs72704075
rs28854187
rs11748731
rs114396963
rs77405243
rs35802809
rs2490034
rs4656251
rs75072970
rs112750600
rs7527826
rs142120486
rs114676248
rs78180560
rs79275634
rs147431504
rs7532602
rs551882
rs10111943
rs11810305
rs35855160
rs17017403
rs139376787
rs76265753
rs142405270
rs76652463
rs10918341
rs6782857
rs71639910
rs115751481
rs138192280
rs34622012
rs1551097
rs2494208
rs12144062
rs76168011
rs9590896
rs146068392
All
−0.5
−0.4
−0.3
−0.2
−0.1
0.0
MR leave−one−out sensitivity analysis for
'ebi−a−GCST90001461' on 'outcome'

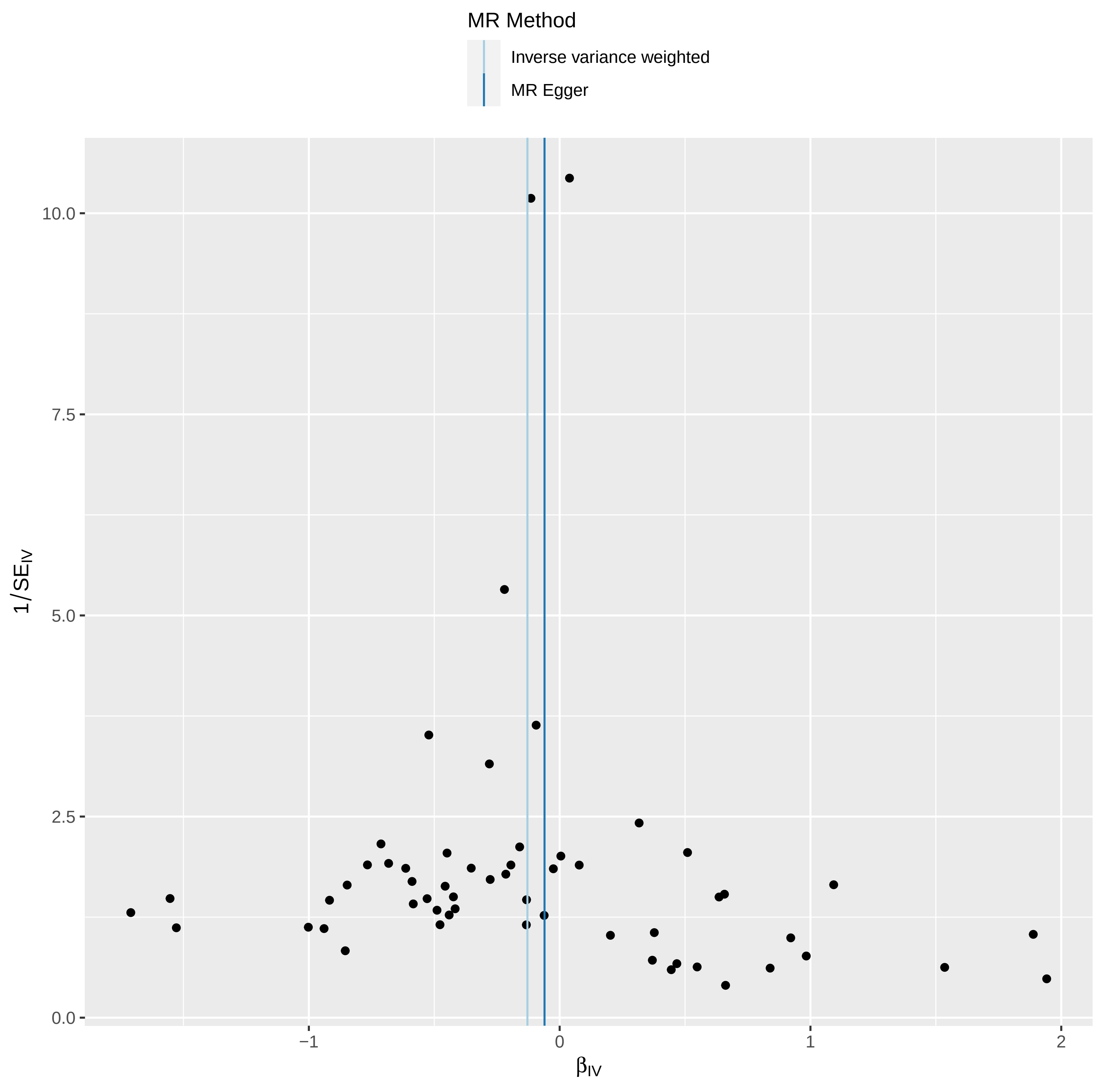
MR Method
Inverse variance weighted
MR Egger
1/SE_IV
β_IV
10.0
7.5
5.0
2.5
0.0
−1
0
1
2

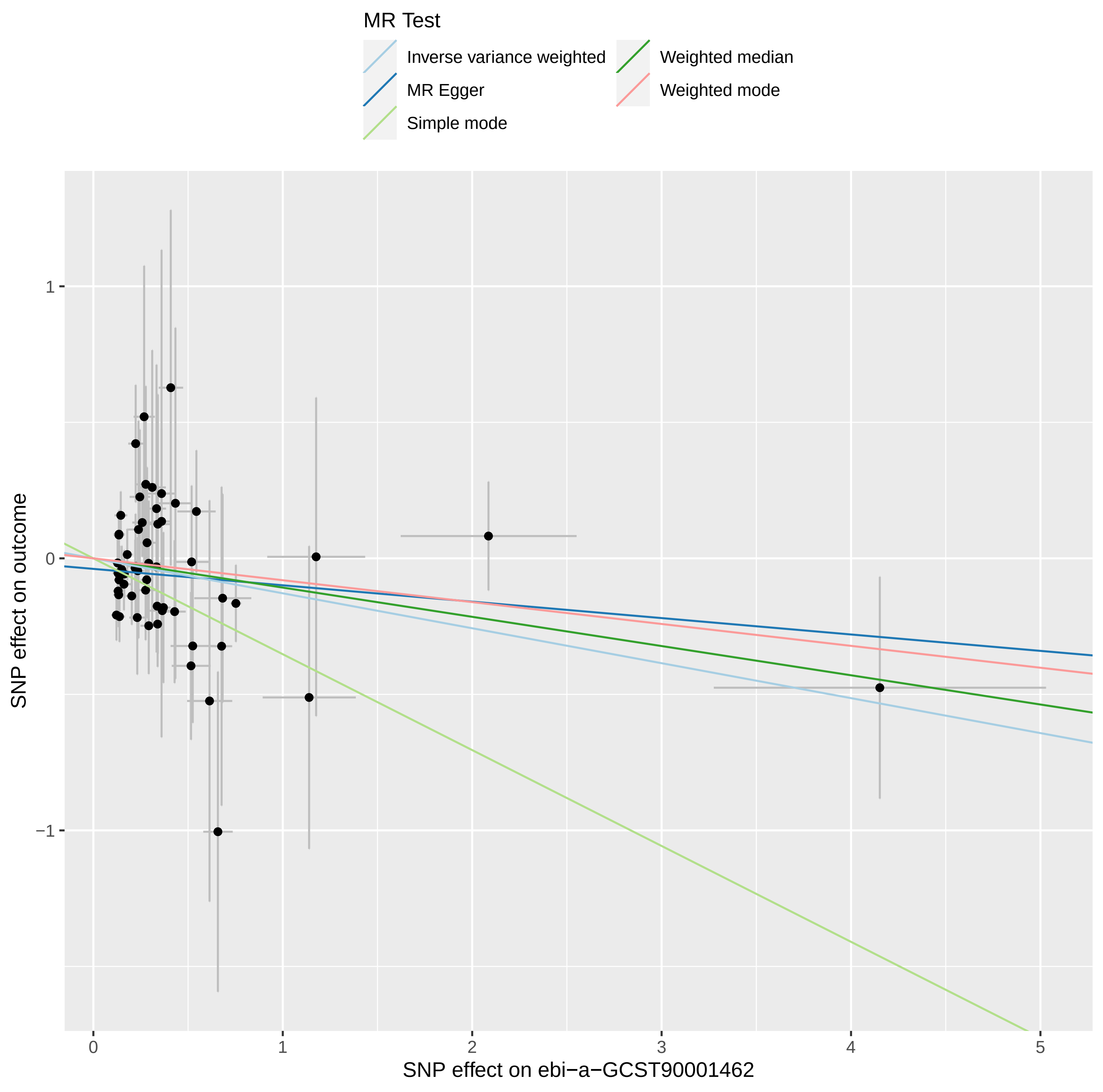

MR Test
Inverse variance weighted
MR Egger
Simple mode
Weighted median
Weighted mode
SNP effect on outcome
1
0
−1
0
1
2
3
4
5
SNP effect on ebi−a−GCST90001462

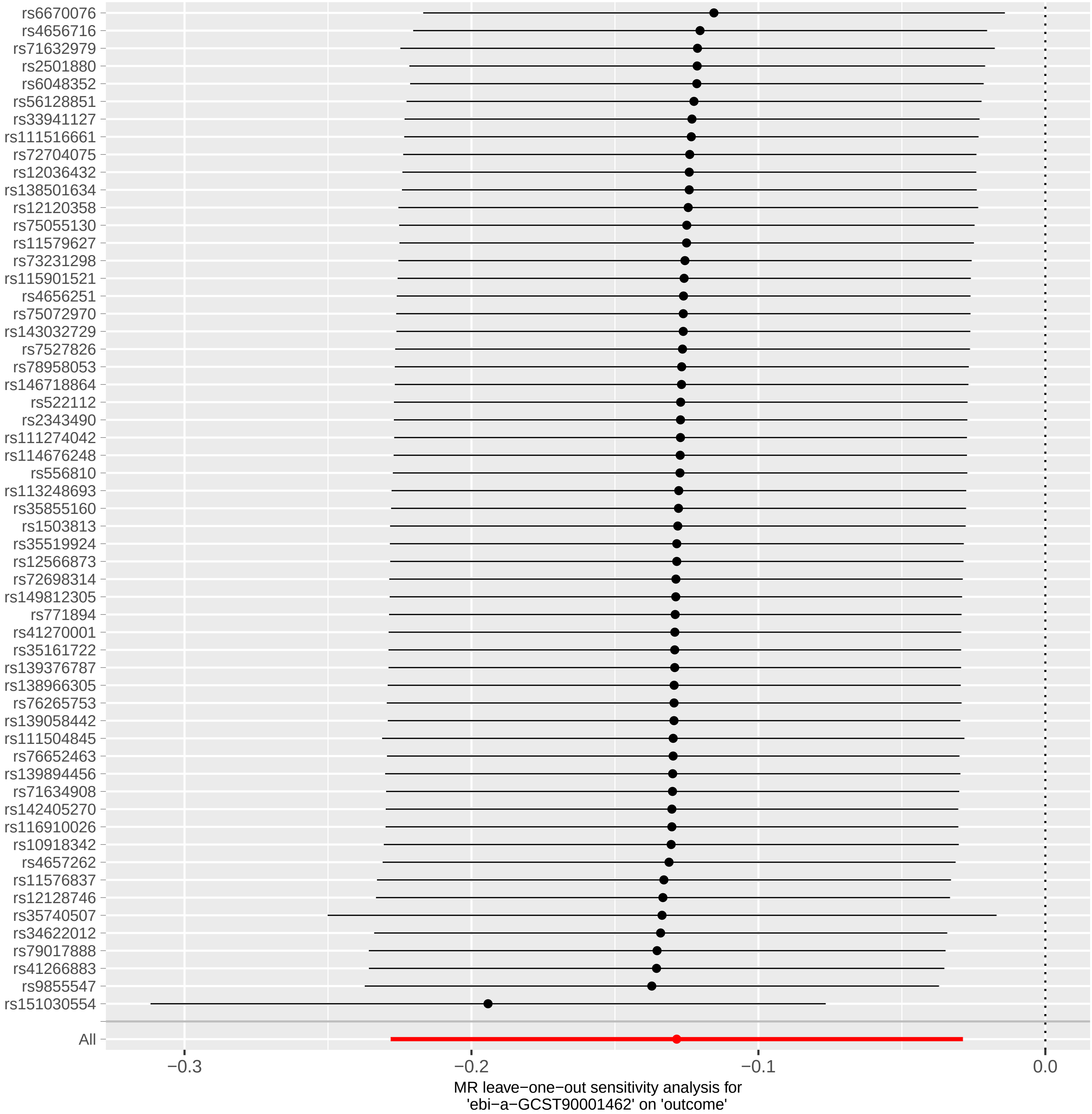
rs6670076
rs4656716
rs71632979
rs2501880
rs6048352
rs56128851
rs33941127
rs111516661
rs72704075
rs12036432
rs138501634
rs12120358
rs75055130
rs11579627
rs73231298
rs115901521
rs4656251
rs75072970
rs143032729
rs7527826
rs78958053
rs146718864
rs522112
rs2343490
rs111274042
rs114676248
rs556810
rs113248693
rs35855160
rs1503813
rs35519924
rs12566873
rs72698314
rs149812305
rs771894
rs41270001
rs35161722
rs139376787
rs138966305
rs76265753
rs139058442
rs111504845
rs76652463
rs139894456
rs71634908
rs142405270
rs116910026
rs10918342
rs4657262
rs11576837
rs12128746
rs35740507
rs34622012
rs79017888
rs41266883
rs9855547
rs151030554
All
−0.3
−0.2
−0.1
0.0
MR leave−one−out sensitivity analysis for
'ebi−a−GCST90001462' on 'outcome'

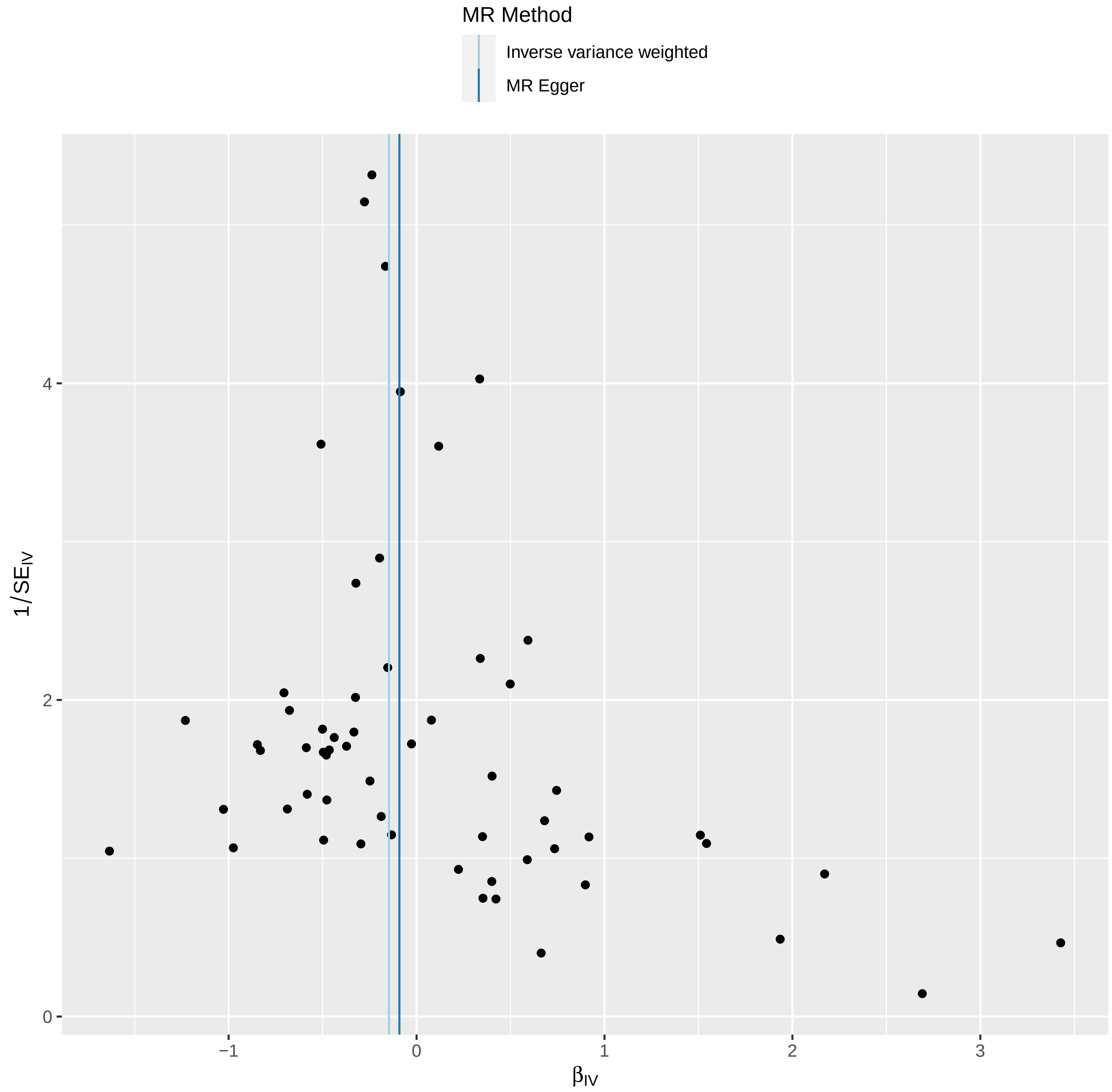

MR Method
Inverse variance weighted
MR Egger
1/SE_IV
4
2
0
−1
0
1
2
3
β_IV

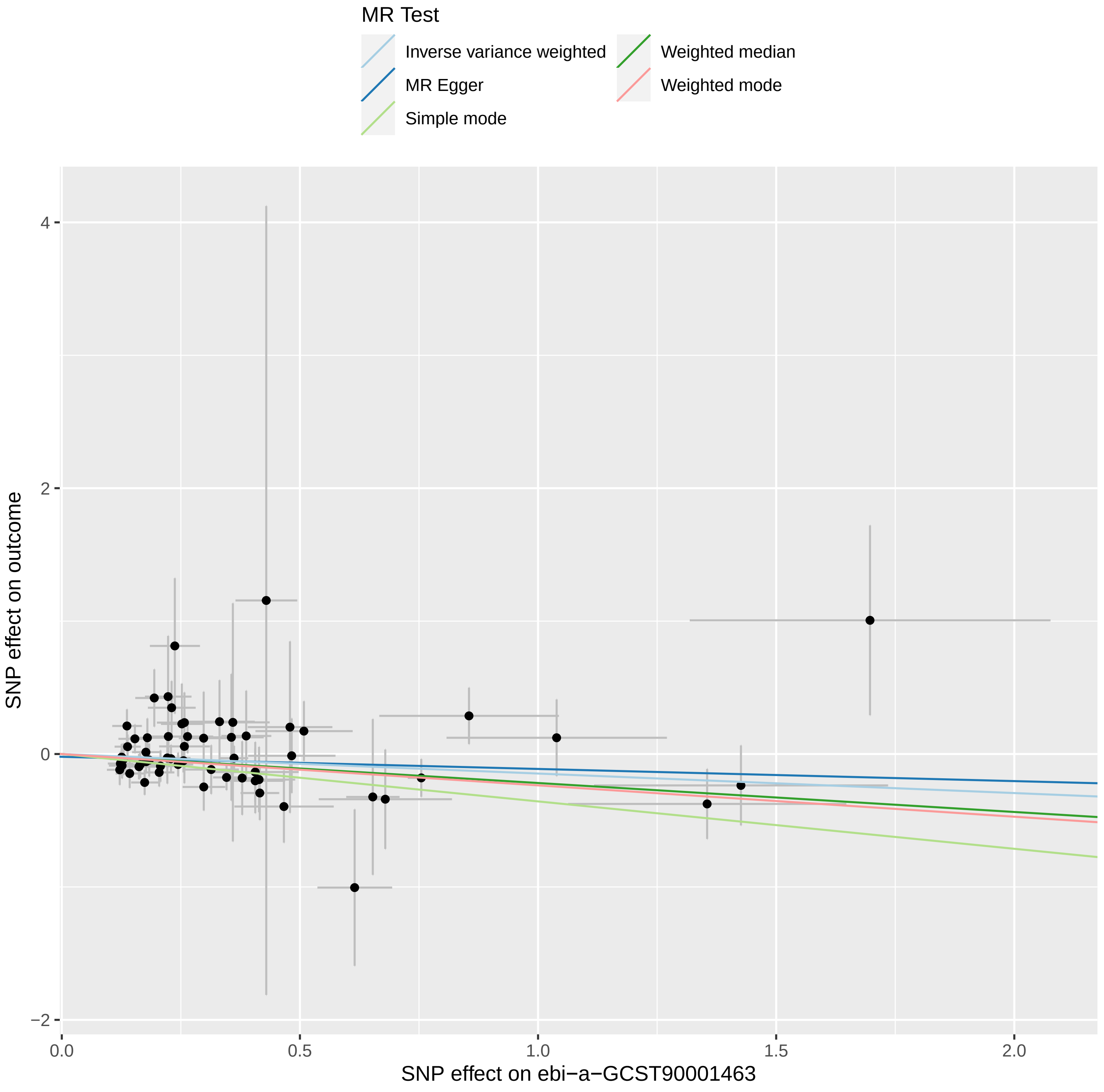

MR Test
Inverse variance weighted
MR Egger
Simple mode
Weighted median
Weighted mode
4
2
0
−2
SNP effect on outcome
0.0
0.5
1.0
1.5
2.0
SNP effect on ebi−a−GCST90001463

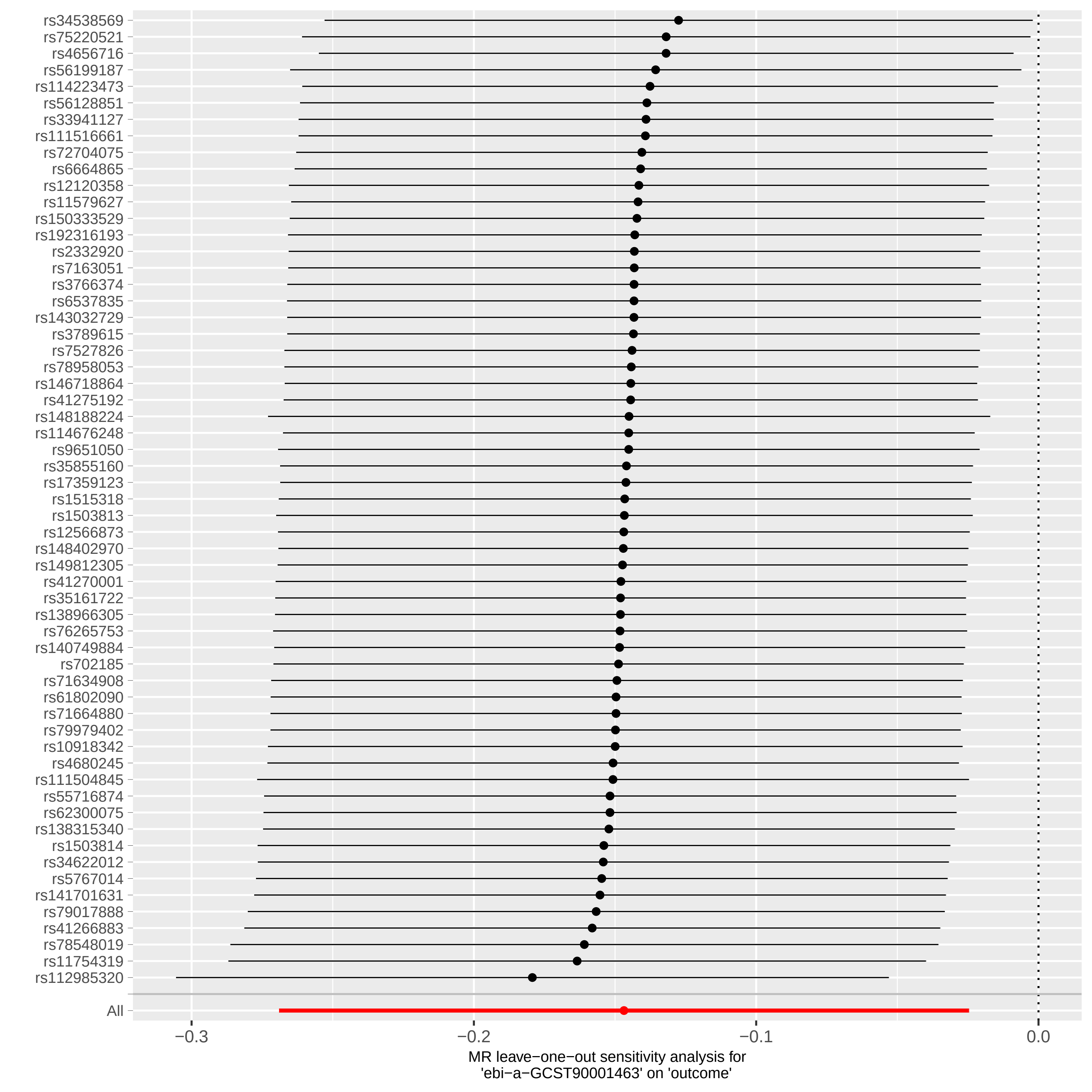
rs34538569
rs75220521
rs4656716
rs56199187
rs114223473
rs56128851
rs33941127
rs111516661
rs72704075
rs6664865
rs12120358
rs11579627
rs150333529
rs192316193
rs2332920
rs7163051
rs3766374
rs6537835
rs143032729
rs3789615
rs7527826
rs78958053
rs146718864
rs41275192
rs148188224
rs114676248
rs9651050
rs35855160
rs17359123
rs1515318
rs1503813
rs12566873
rs148402970
rs149812305
rs41270001
rs35161722
rs138966305
rs76265753
rs140749884
rs702185
rs71634908
rs61802090
rs71664880
rs79979402
rs10918342
rs4680245
rs111504845
rs55716874
rs62300075
rs138315340
rs1503814
rs34622012
rs5767014
rs141701631
rs79017888
rs41266883
rs78548019
rs11754319
rs112985320
All
−0.3
−0.2
−0.1
0.0
MR leave−one−out sensitivity analysis for
'ebi−a−GCST90001463' on 'outcome'

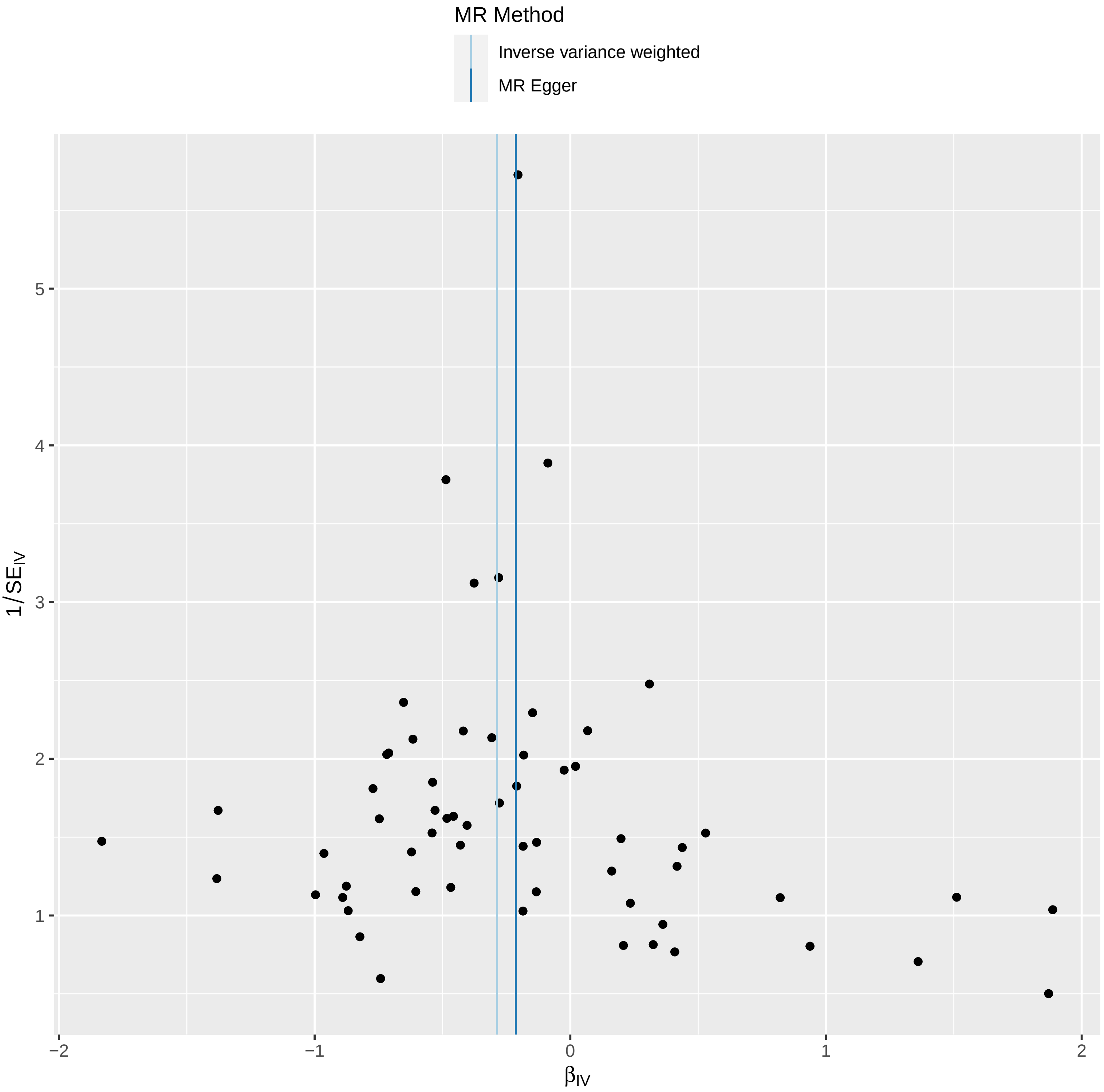
MR Method
Inverse variance weighted
MR Egger
1/SE_IV
β_IV
5
4
3
2
1
−2
−1
0
1
2

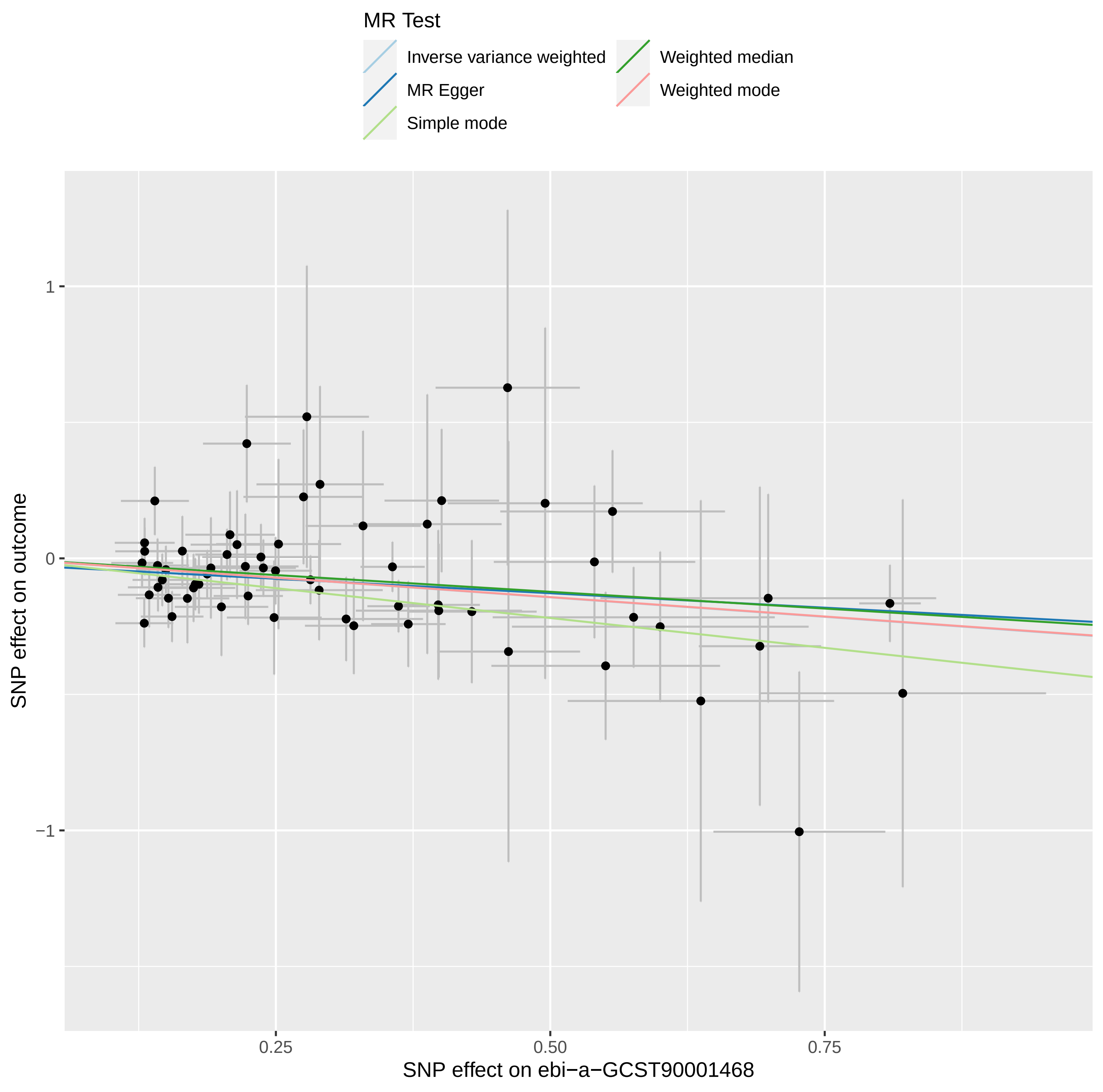

MR Test
Inverse variance weighted
MR Egger
Simple mode
Weighted median
Weighted mode
1
0
−1
0.25
0.50
0.75
SNP effect on outcome
SNP effect on ebi−a−GCST90001468

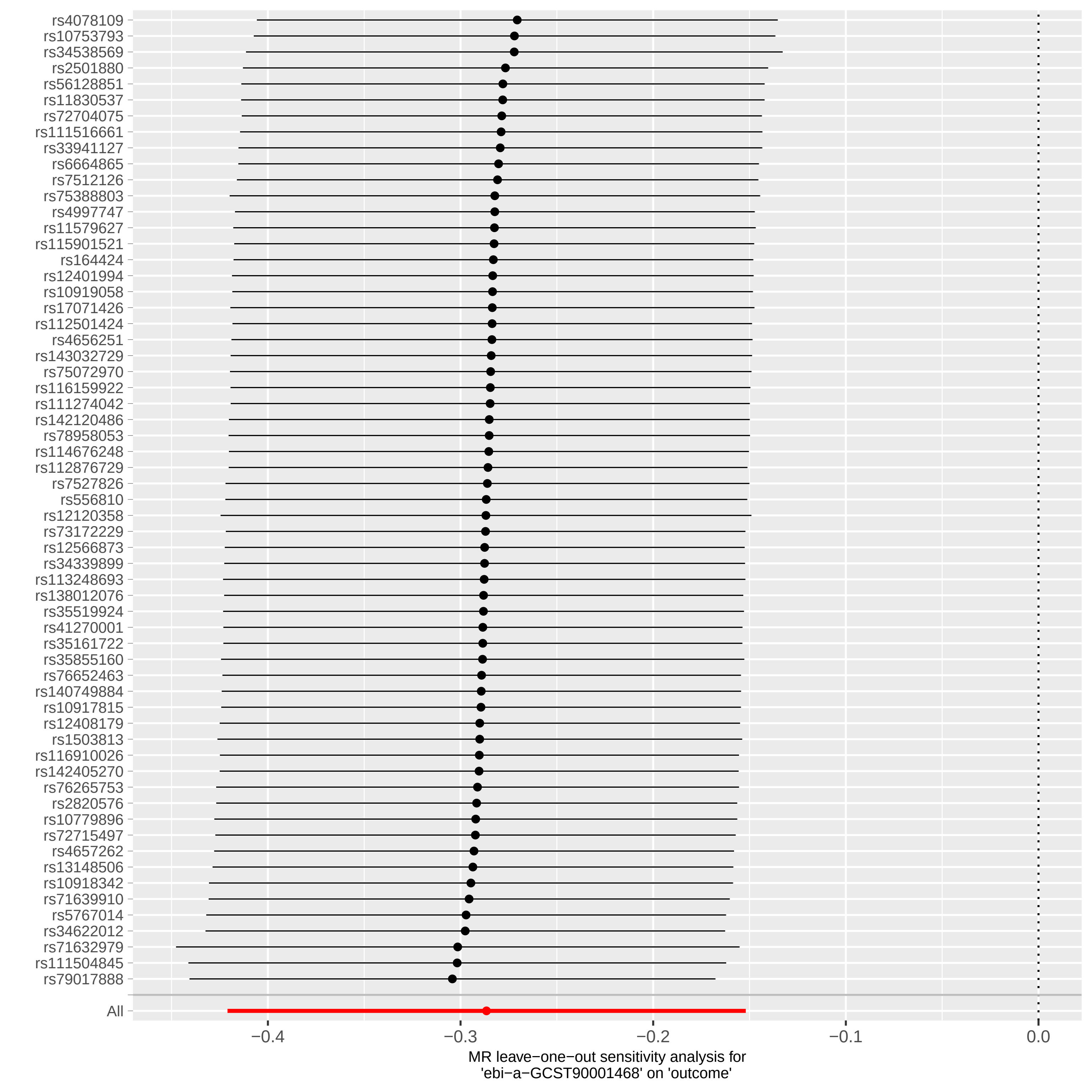
rs4078109
rs10753793
rs34538569
rs2501880
rs56128851
rs11830537
rs72704075
rs111516661
rs33941127
rs6664865
rs7512126
rs75388803
rs4997747
rs11579627
rs115901521
rs164424
rs12401994
rs10919058
rs17071426
rs112501424
rs4656251
rs143032729
rs75072970
rs116159922
rs111274042
rs142120486
rs78958053
rs114676248
rs112876729
rs7527826
rs556810
rs12120358
rs73172229
rs12566873
rs34339899
rs113248693
rs138012076
rs35519924
rs41270001
rs35161722
rs35855160
rs76652463
rs140749884
rs10917815
rs12408179
rs1503813
rs116910026
rs142405270
rs76265753
rs2820576
rs10779896
rs72715497
rs4657262
rs13148506
rs10918342
rs71639910
rs5767014
rs34622012
rs71632979
rs111504845
rs79017888
All
−0.4
−0.3
−0.2
−0.1
0.0
MR leave−one−out sensitivity analysis for
'ebi−a−GCST90001468' on 'outcome'

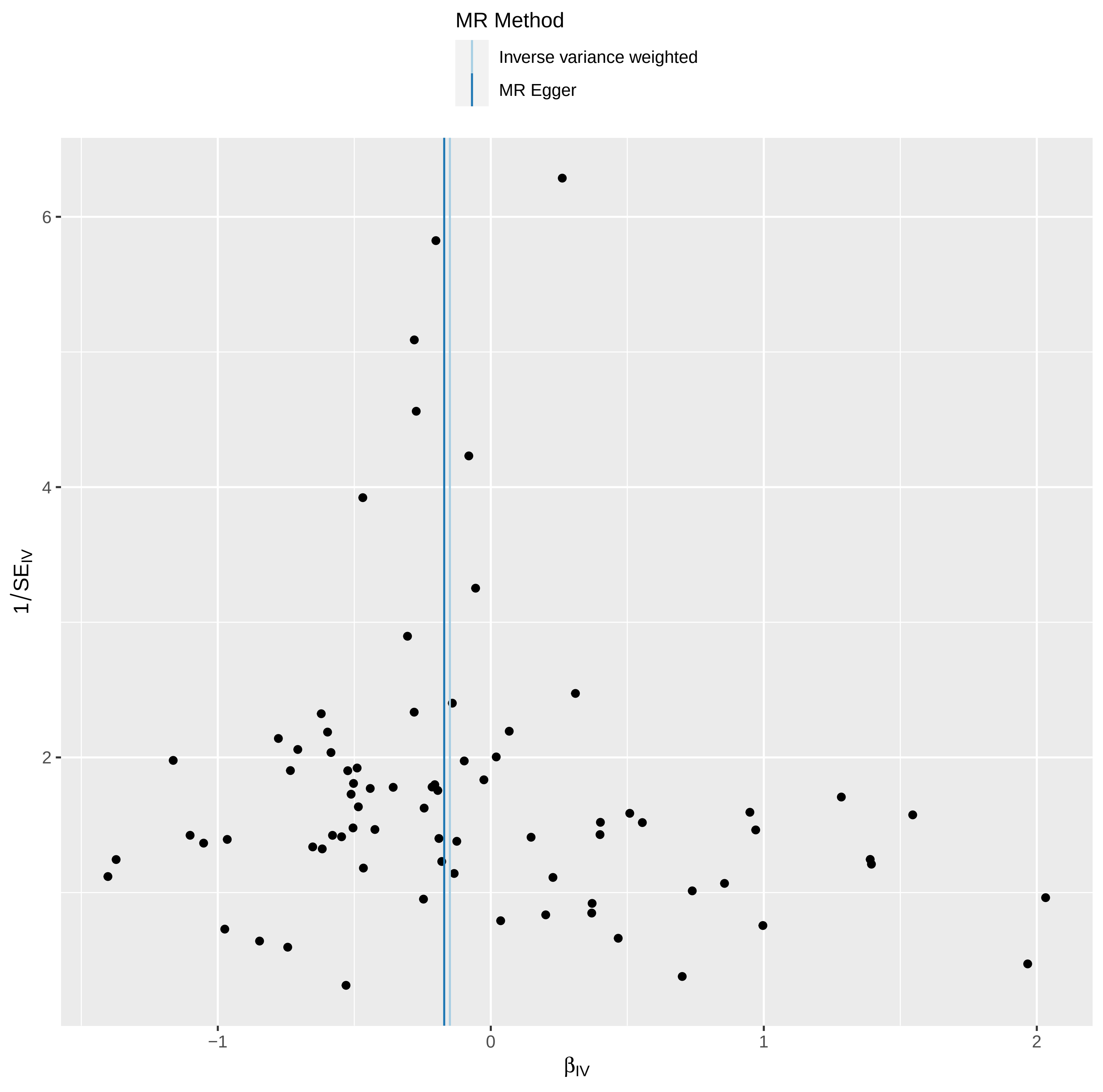

MR Method
Inverse variance weighted
MR Egger
1/SE_IV
β_IV
6
4
2
−1
0
1
2

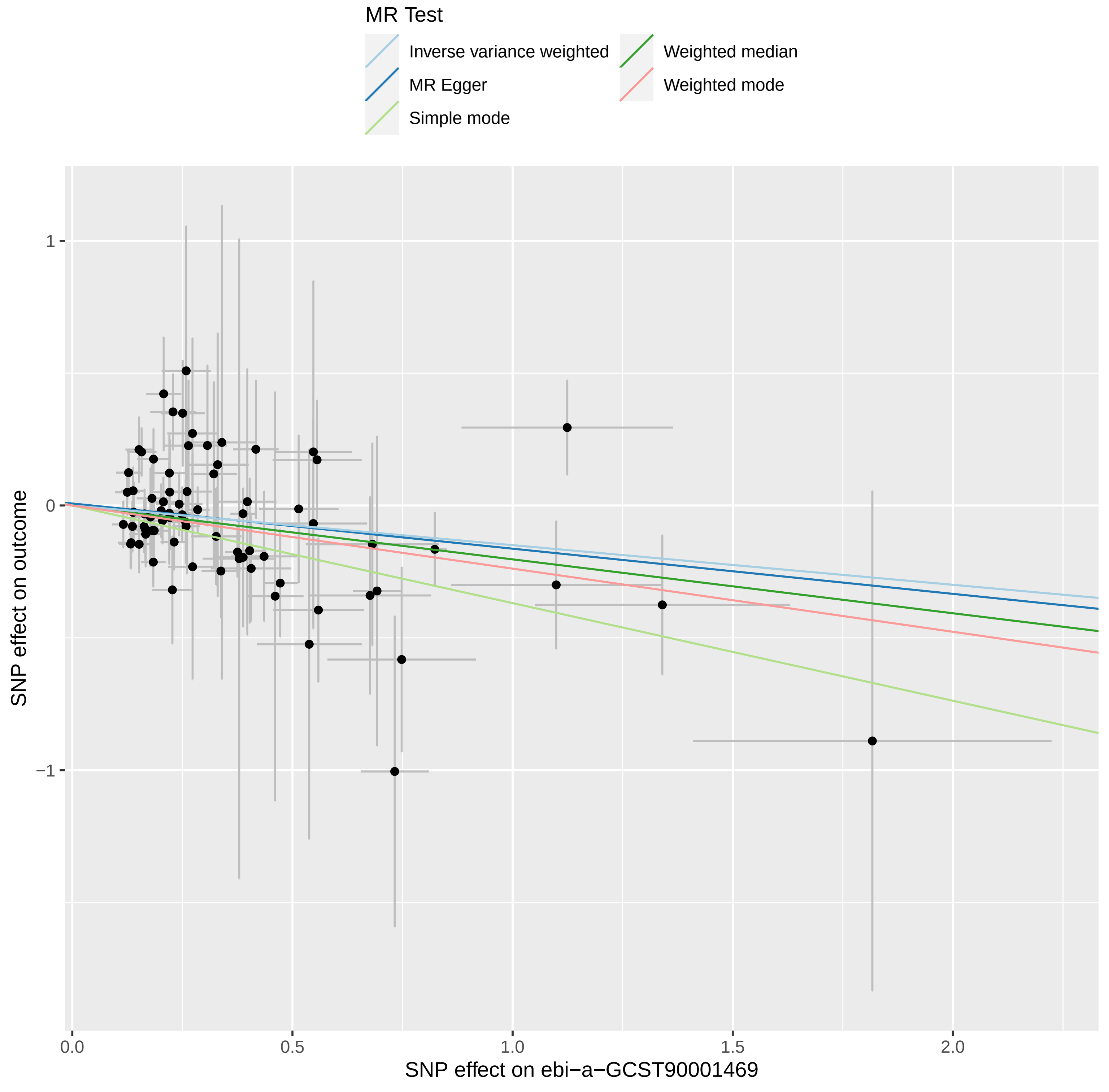

MR Test
Inverse variance weighted
MR Egger
Simple mode
Weighted median
Weighted mode
1
0
−1
0.0
0.5
1.0
1.5
2.0
SNP effect on outcome
SNP effect on ebi−a−GCST90001469

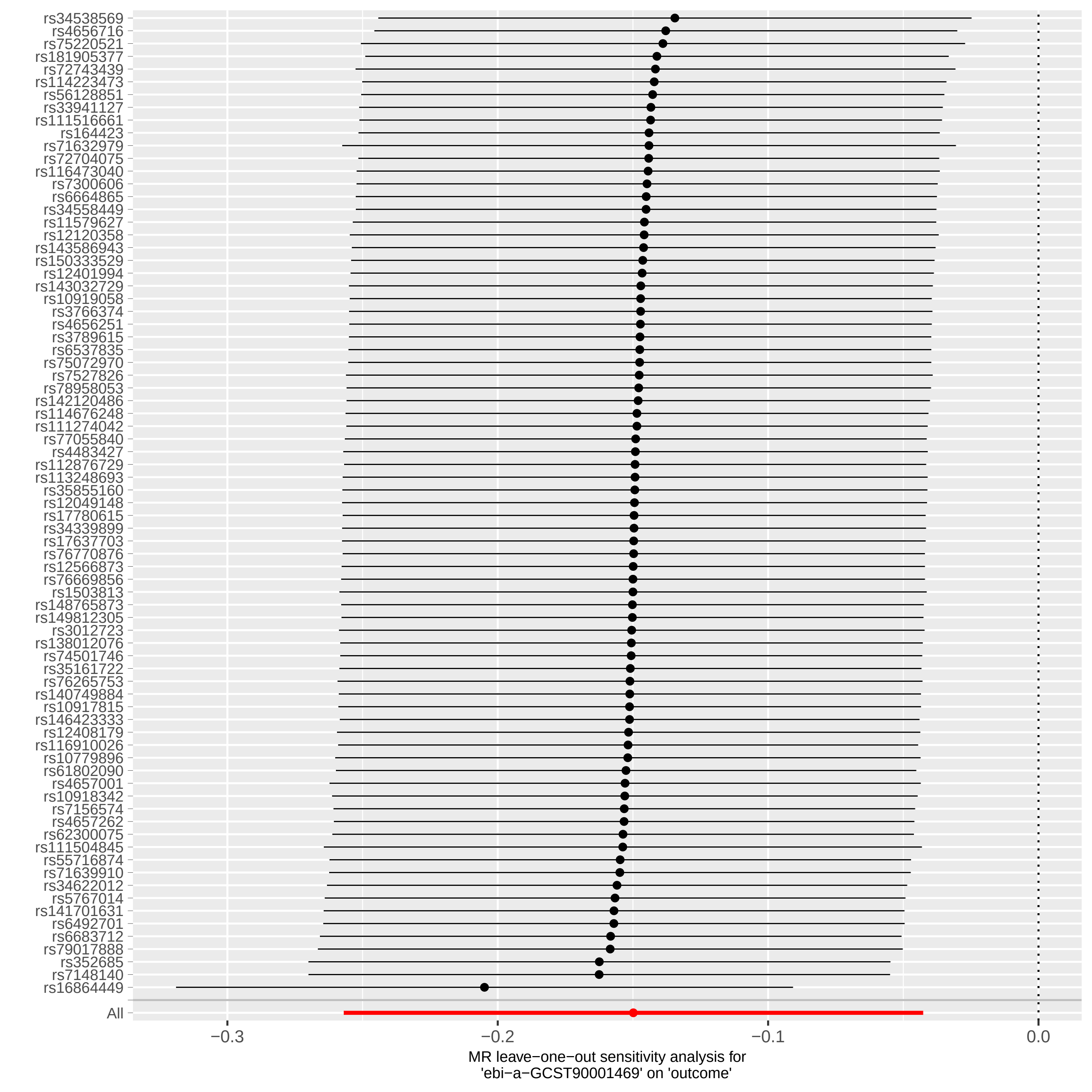

rs34538569
rs4656716
rs75220521
rs181905377
rs72743439
rs114223473
rs56128851
rs33941127
rs111516661
rs164423
rs71632979
rs72704075
rs116473040
rs7300606
rs6664865
rs34558449
rs11579627
rs12120358
rs143586943
rs150333529
rs12401994
rs143032729
rs10919058
rs3766374
rs4656251
rs3789615
rs6537835
rs75072970
rs7527826
rs78958053
rs142120486
rs114676248
rs111274042
rs77055840
rs4483427
rs112876729
rs113248693
rs35855160
rs12049148
rs17780615
rs34339899
rs17637703
rs76770876
rs12566873
rs76669856
rs1503813
rs148765873
rs149812305
rs3012723
rs138012076
rs74501746
rs35161722
rs76265753
rs140749884
rs10917815
rs146423333
rs12408179
rs116910026
rs10779896
rs61802090
rs4657001
rs10918342
rs7156574
rs4657262
rs62300075
rs111504845
rs55716874
rs71639910
rs34622012
rs5767014
rs141701631
rs6492701
rs6683712
rs79017888
rs352685
rs7148140
rs16864449
All
−0.3
−0.2
−0.1
0.0
MR leave−one−out sensitivity analysis for
'ebi−a−GCST90001469' on 'outcome'

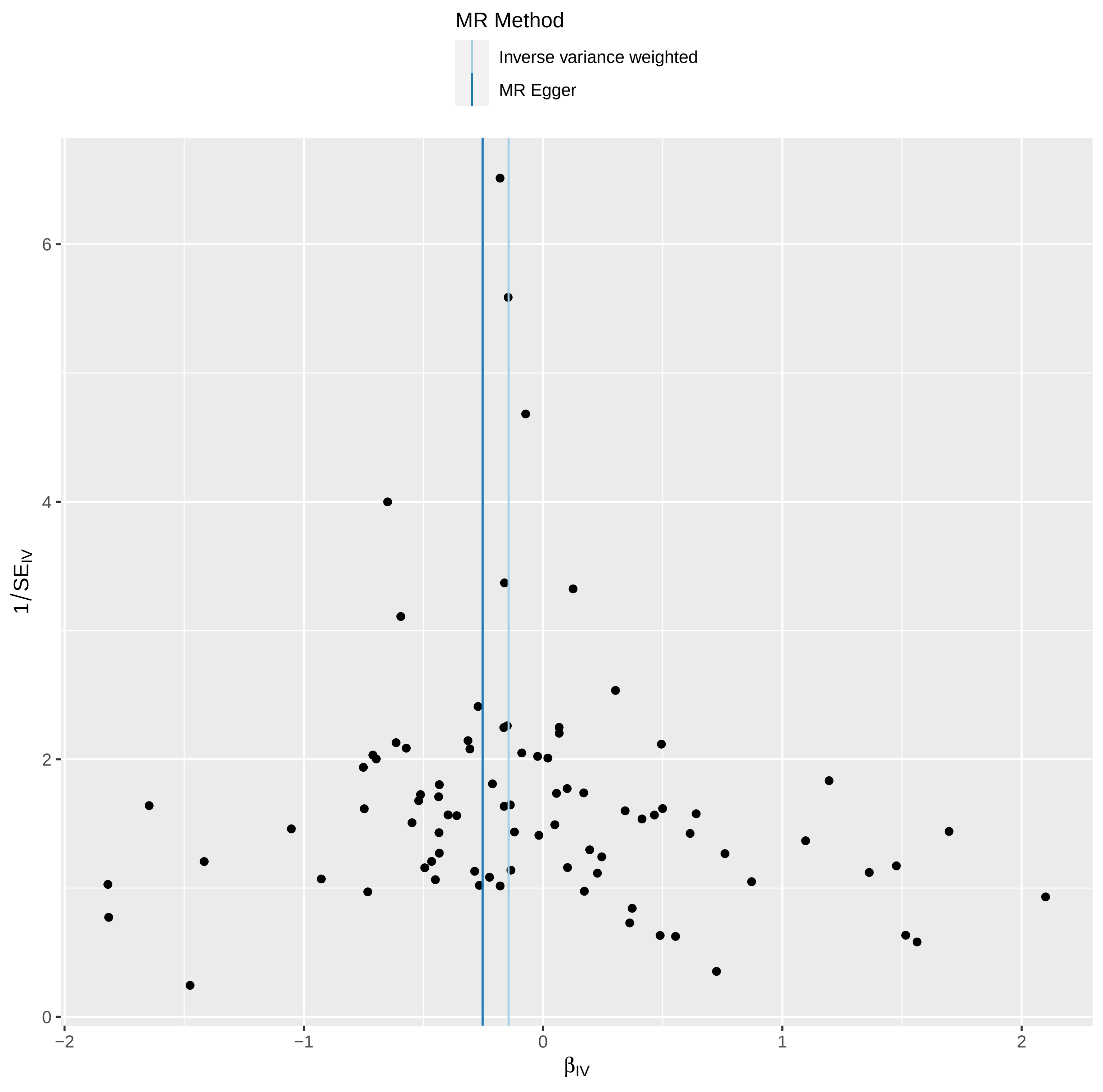

MR Method
Inverse variance weighted
MR Egger
6
4
2
0
−2
−1
0
1
2
1/SE_IV
β_IV

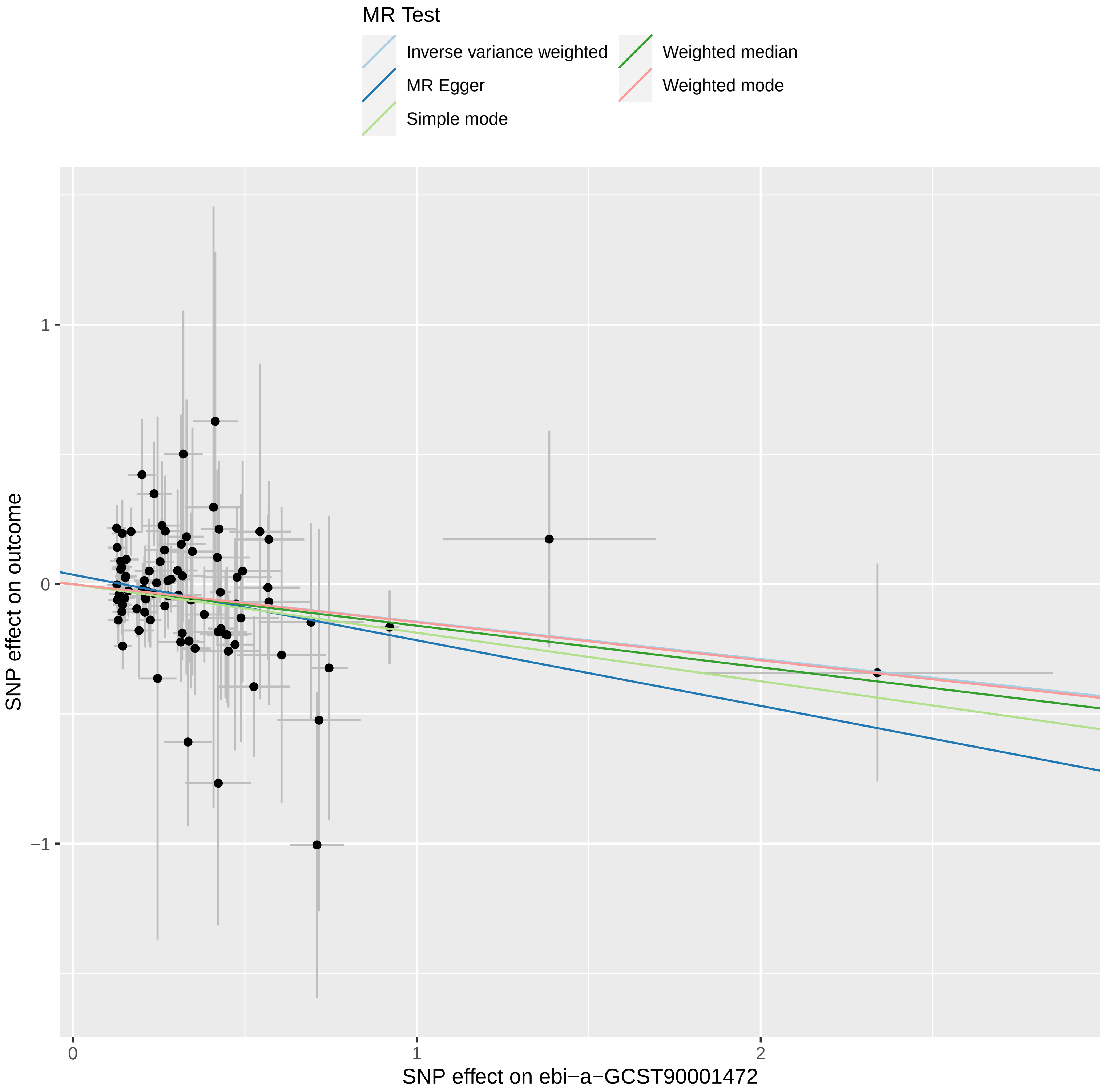

MR Test
Inverse variance weighted
MR Egger
Simple mode
Weighted median
Weighted mode
SNP effect on outcome
1
0
−1
0
1
2
SNP effect on ebi−a−GCST90001472

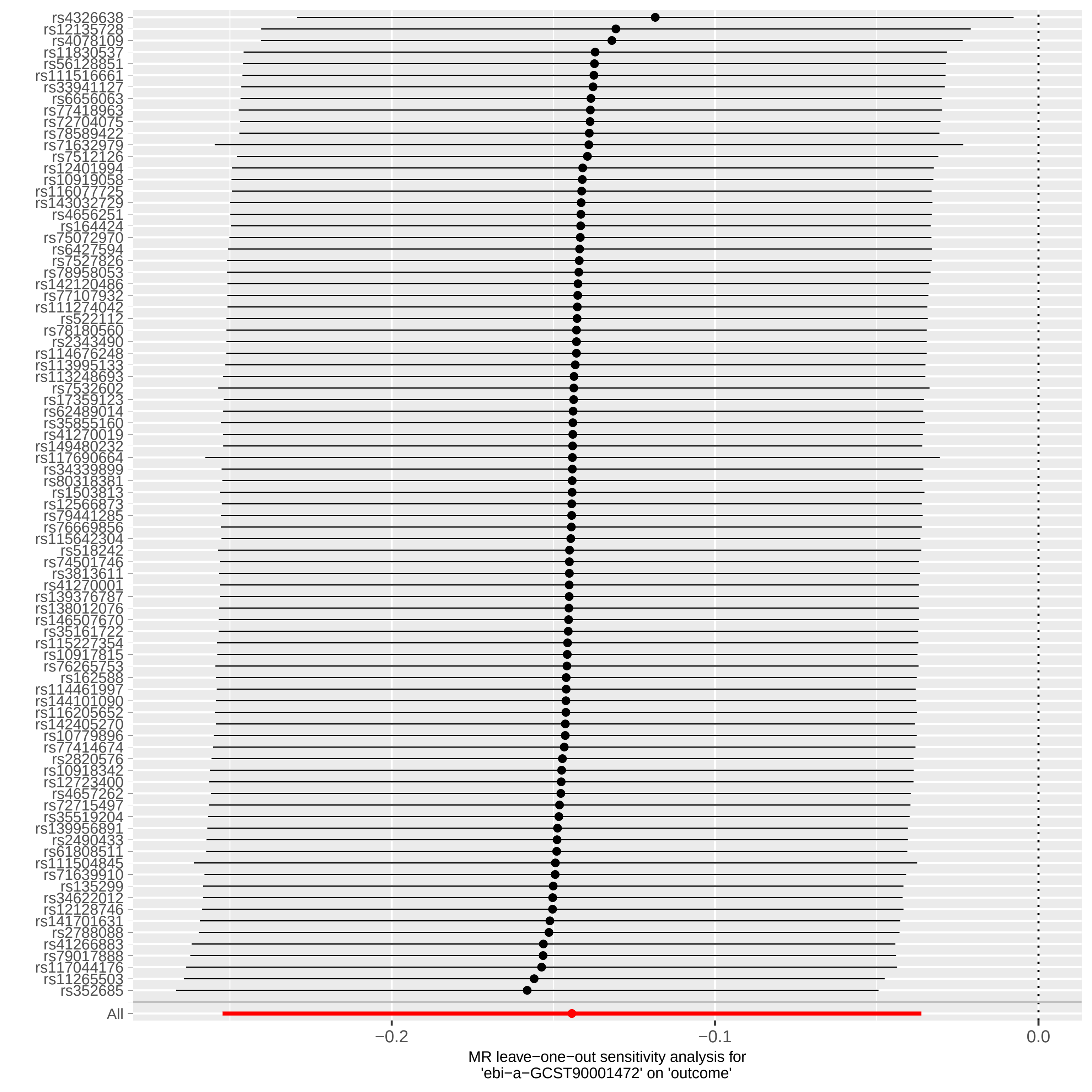

rs4326638
rs12135728
rs4078109
rs11830537
rs56128851
rs111516661
rs33941127
rs6656063
rs77418963
rs72704075
rs78589422
rs71632979
rs7512126
rs12401994
rs10919058
rs116077725
rs143032729
rs4656251
rs164424
rs75072970
rs6427594
rs7527826
rs78958053
rs142120486
rs77107932
rs111274042
rs522112
rs78180560
rs2343490
rs114676248
rs113995133
rs113248693
rs7532602
rs17359123
rs62489014
rs35855160
rs41270019
rs149480232
rs117690664
rs34339899
rs80318381
rs1503813
rs12566873
rs79441285
rs76669856
rs115642304
rs518242
rs74501746
rs3813611
rs41270001
rs139376787
rs138012076
rs146507670
rs35161722
rs115227354
rs10917815
rs76265753
rs162588
rs114461997
rs144101090
rs116205652
rs142405270
rs10779896
rs77414674
rs2820576
rs10918342
rs12723400
rs4657262
rs72715497
rs35519204
rs139956891
rs2490433
rs61808511
rs111504845
rs71639910
rs135299
rs34622012
rs12128746
rs141701631
rs2788088
rs41266883
rs79017888
rs117044176
rs11265503
rs352685
All
−0.2
−0.1
0.0
MR leave−one−out sensitivity analysis for
'ebi−a−GCST90001472' on 'outcome'

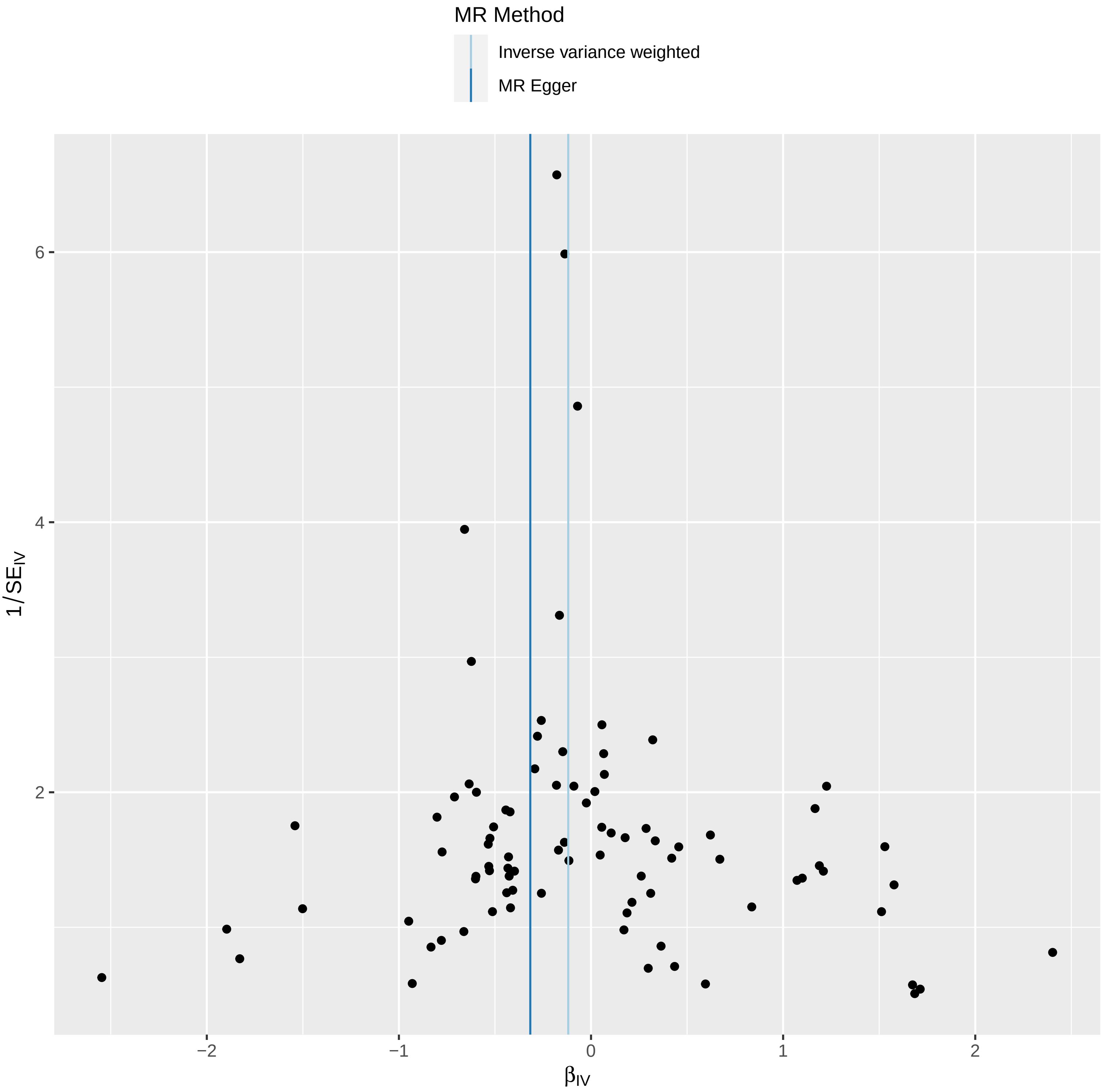
MR Method
Inverse variance weighted
MR Egger
1/SE_IV
6
4
2
−2
−1
0
1
2
β_IV

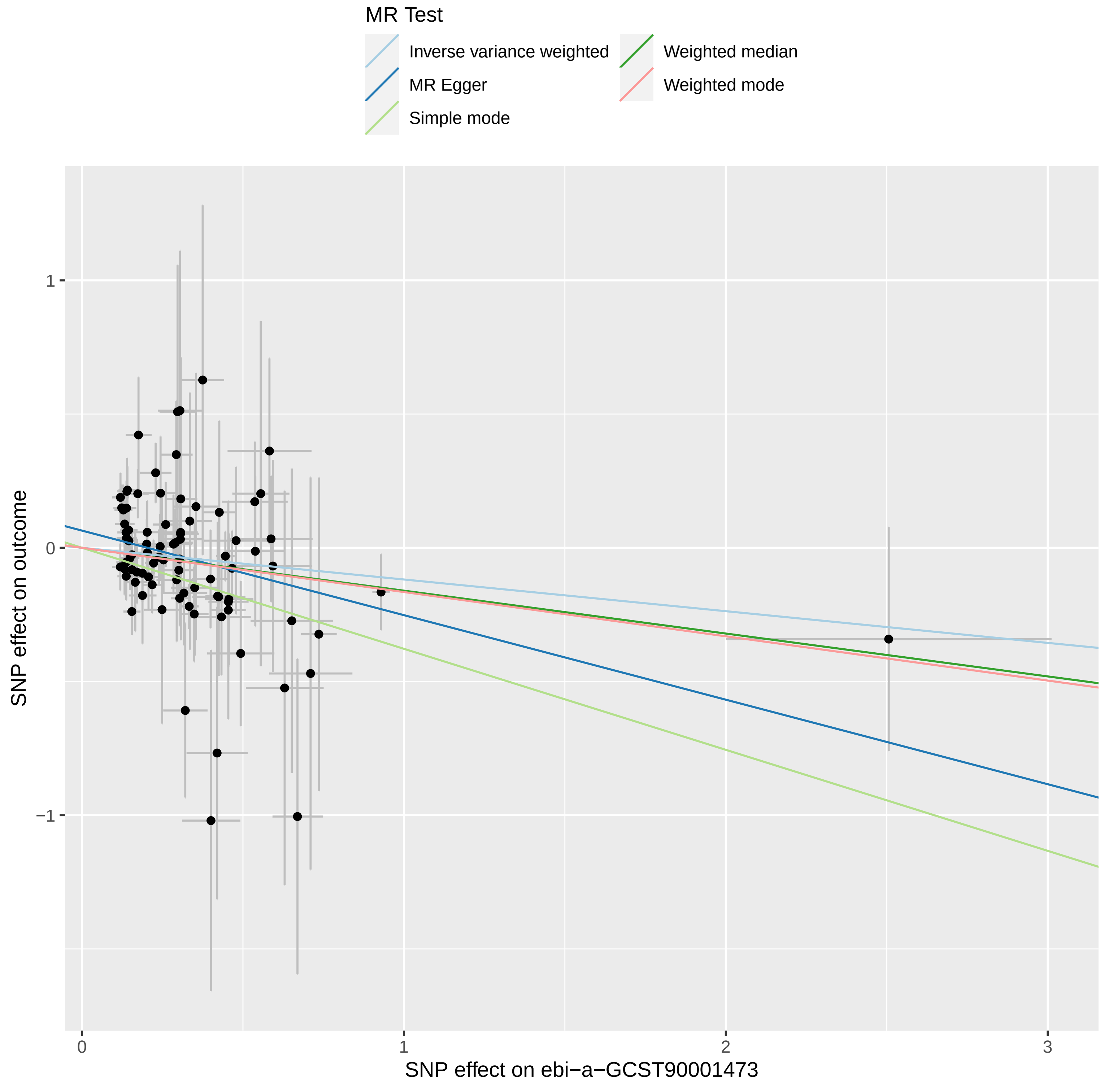
MR Test
Inverse variance weighted
MR Egger
Simple mode
Weighted median
Weighted mode
SNP effect on outcome
1
0
−1
0
1
2
3
SNP effect on ebi−a−GCST90001473

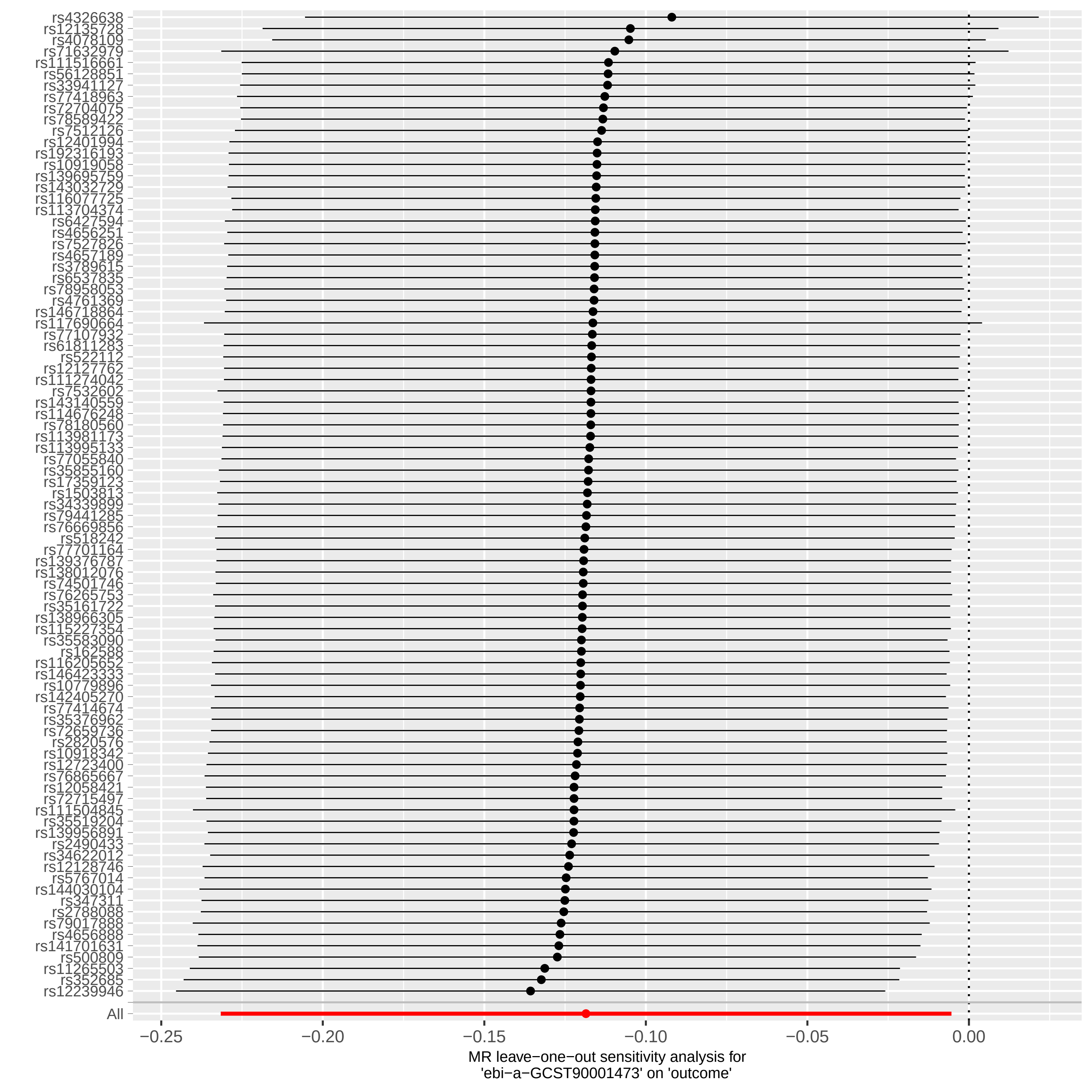
rs4326638
rs12135728
rs4078109
rs71632979
rs111516661
rs56128851
rs33941127
rs77418963
rs72704075
rs78589422
rs7512126
rs12401994
rs192316193
rs10919058
rs139695759
rs143032729
rs116077725
rs113704374
rs6427594
rs4656251
rs7527826
rs4657189
rs3789615
rs6537835
rs78958053
rs4761369
rs146718864
rs117690664
rs77107932
rs61811283
rs522112
rs12127762
rs111274042
rs7532602
rs143140559
rs114676248
rs78180560
rs113981173
rs113995133
rs77055840
rs35855160
rs17359123
rs1503813
rs34339899
rs79441285
rs76669856
rs518242
rs77701164
rs139376787
rs138012076
rs74501746
rs76265753
rs35161722
rs138966305
rs115227354
rs35583090
rs162588
rs116205652
rs146423333
rs10779896
rs142405270
rs77414674
rs35376962
rs72659736
rs2820576
rs10918342
rs12723400
rs76865667
rs12058421
rs72715497
rs111504845
rs35519204
rs139956891
rs2490433
rs34622012
rs12128746
rs5767014
rs144030104
rs347311
rs2788088
rs79017888
rs4656888
rs141701631
rs500809
rs11265503
rs352685
rs12239946
All
−0.25
−0.20
−0.15
−0.10
−0.05
0.00
MR leave−one−out sensitivity analysis for
'ebi−a−GCST90001473' on 'outcome'

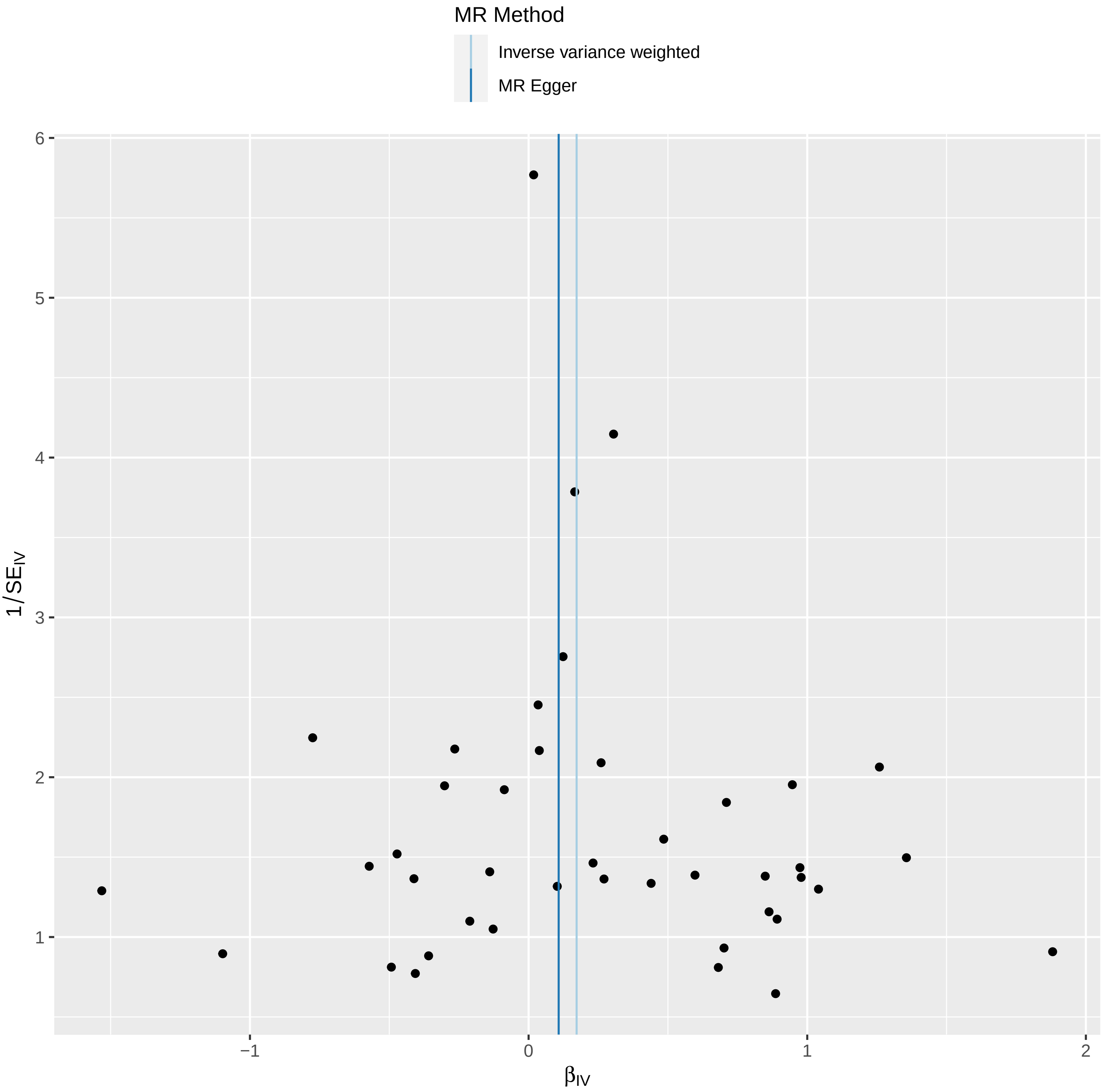

MR Method
Inverse variance weighted
MR Egger
1/SE_{IV}
β_{IV}
6
5
4
3
2
1
−1
0
1
2

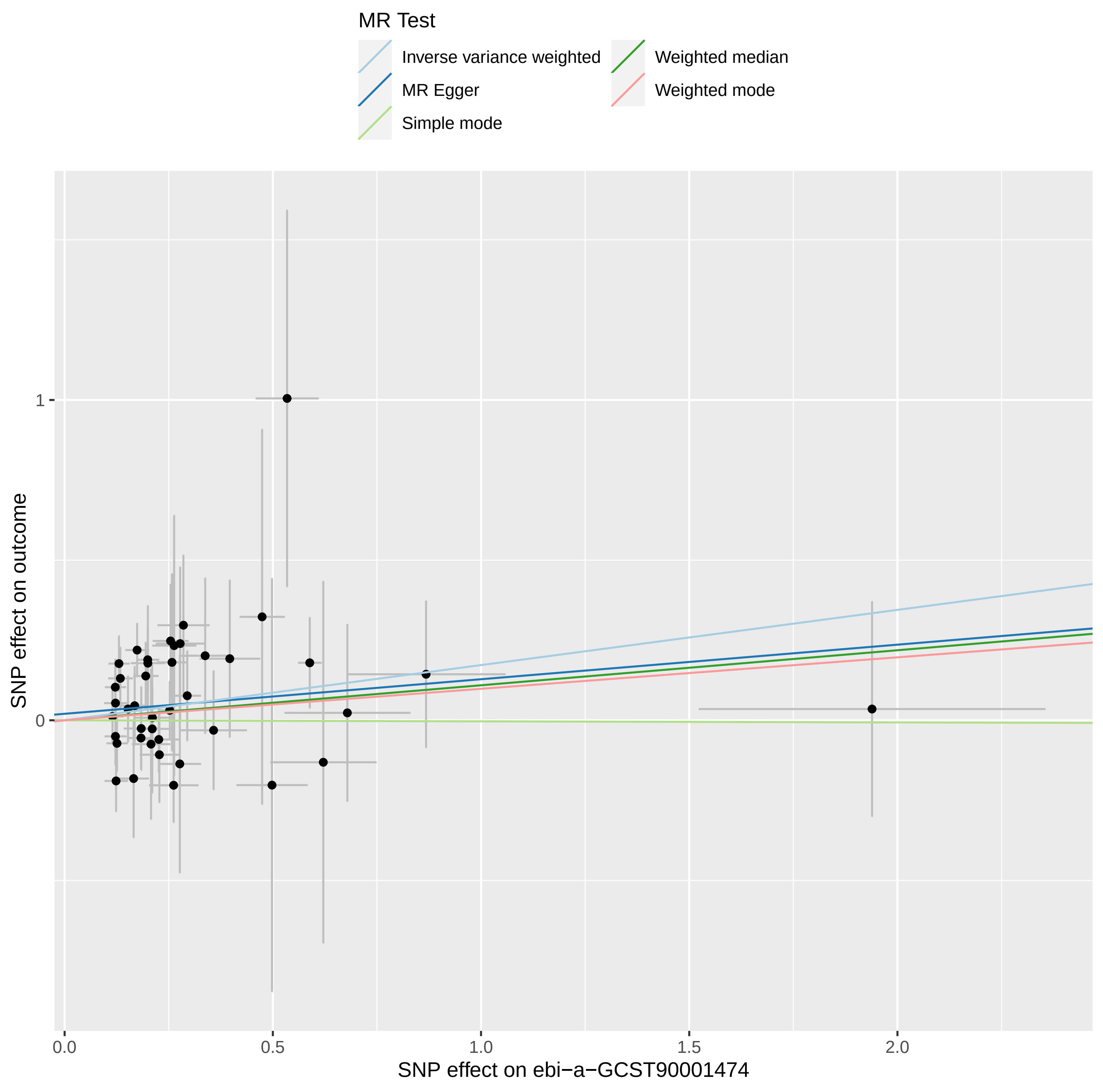
MR Test
Inverse variance weighted
MR Egger
Simple mode
Weighted median
Weighted mode
SNP effect on outcome
1
0
0.0
0.5
1.0
1.5
2.0
SNP effect on ebi−a−GCST90001474

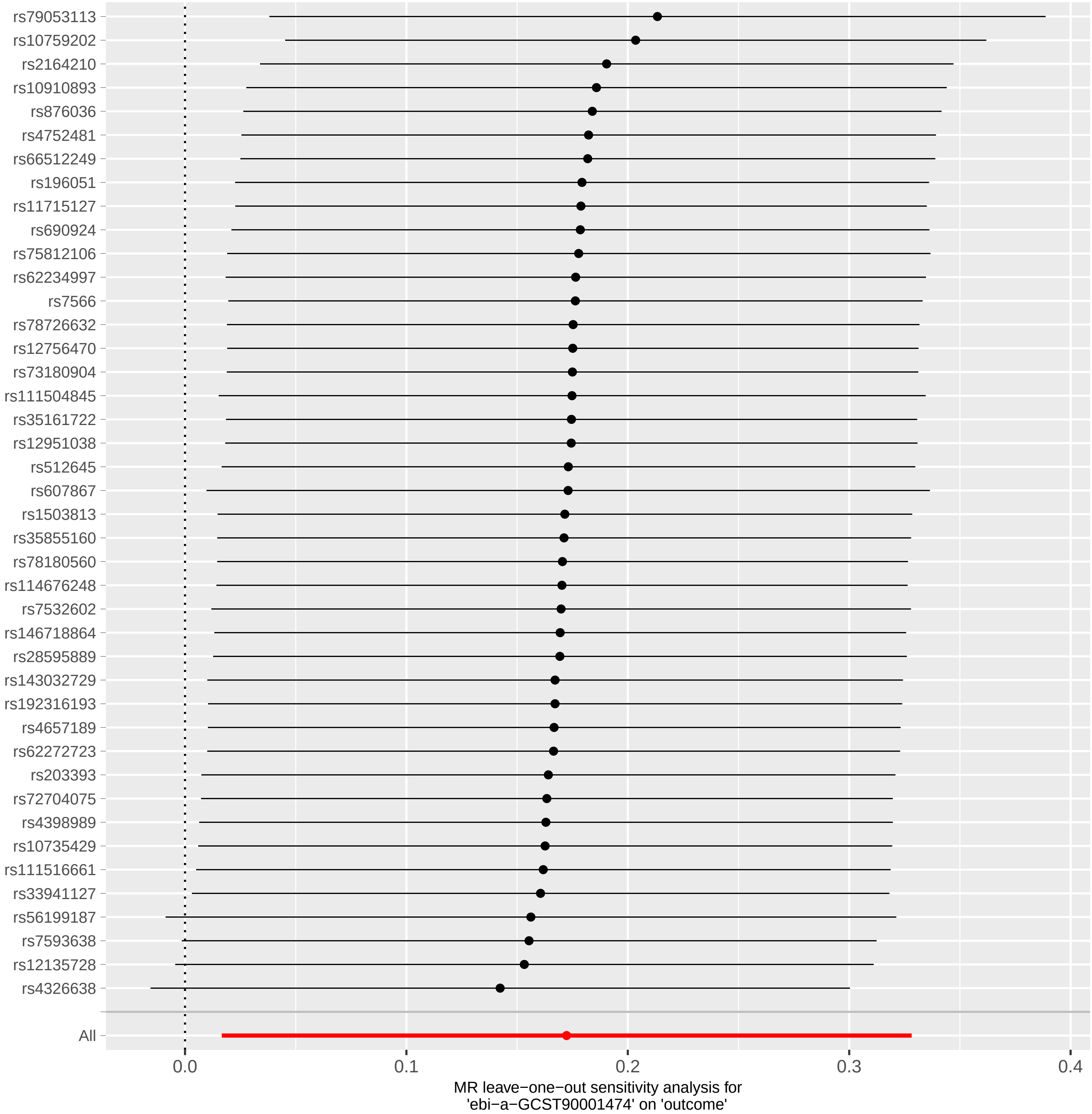

rs79053113
rs10759202
rs2164210
rs10910893
rs876036
rs4752481
rs66512249
rs196051
rs11715127
rs690924
rs75812106
rs62234997
rs7566
rs78726632
rs12756470
rs73180904
rs111504845
rs35161722
rs12951038
rs512645
rs607867
rs1503813
rs35855160
rs78180560
rs114676248
rs7532602
rs146718864
rs28595889
rs143032729
rs192316193
rs4657189
rs62272723
rs203393
rs72704075
rs4398989
rs10735429
rs111516661
rs33941127
rs56199187
rs7593638
rs12135728
rs4326638
All
0.0
0.1
0.2
0.3
0.4
MR leave−one−out sensitivity analysis for
'ebi−a−GCST90001474' on 'outcome'

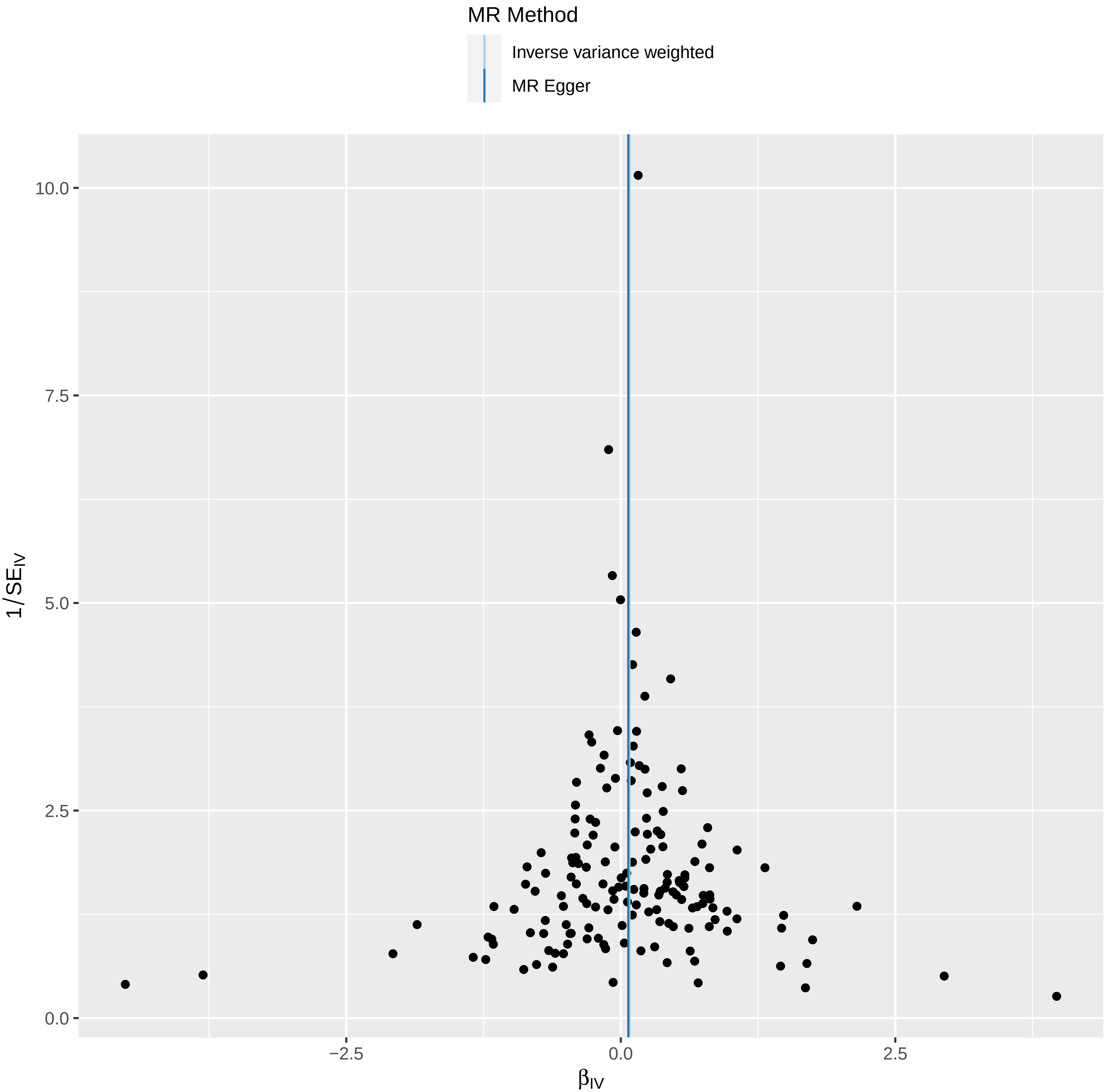

MR Method
Inverse variance weighted
MR Egger
10.0
7.5
5.0
2.5
0.0
1/SE_IV
−2.5
0.0
2.5
β_IV

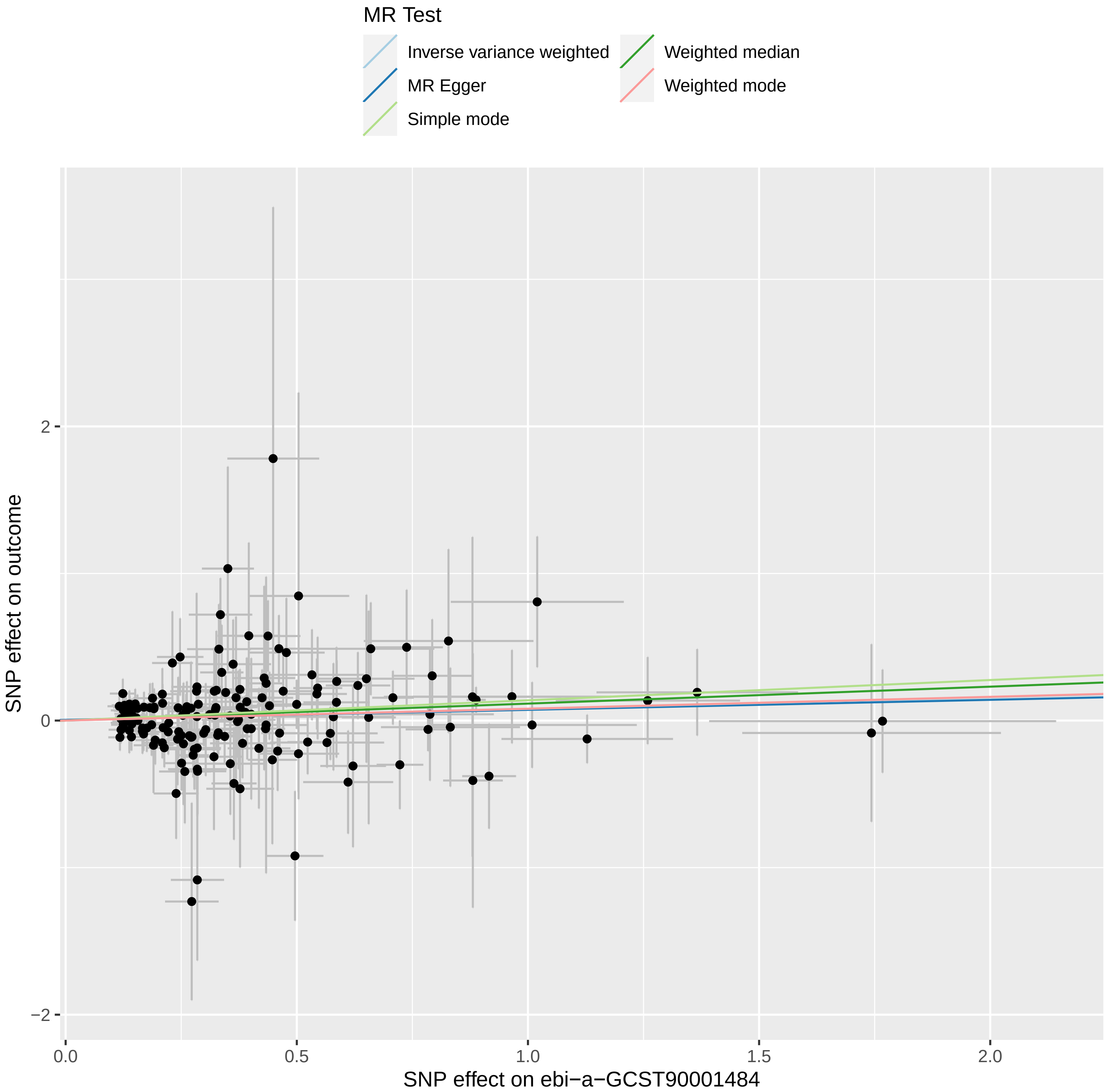
MR Test
Inverse variance weighted
MR Egger
Simple mode
Weighted median
Weighted mode
SNP effect on outcome
SNP effect on ebi−a−GCST90001484
2
0
−2
0.0
0.5
1.0
1.5
2.0

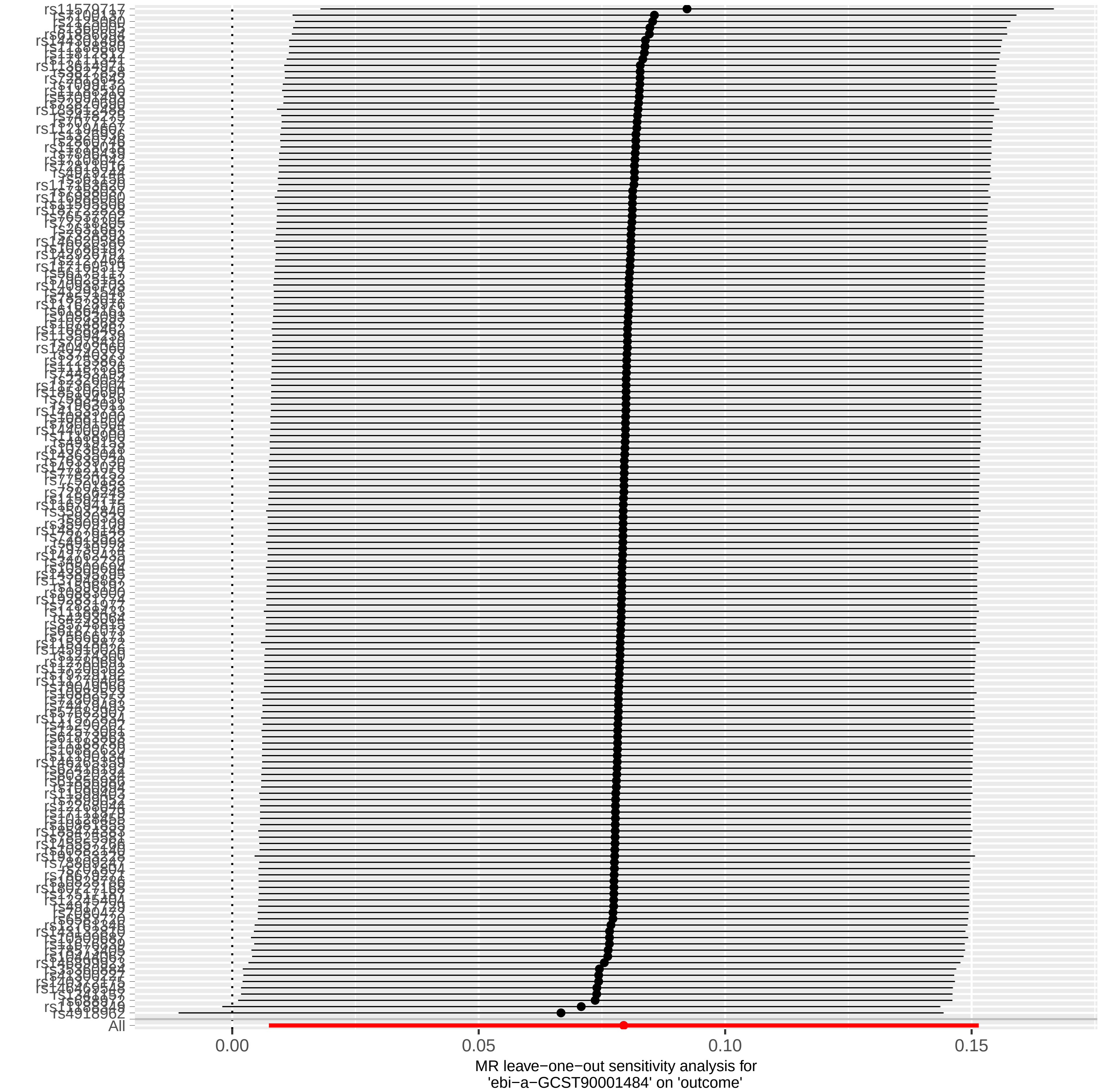
rs11579717
rs4918902
All
0.00
0.05
0.10
0.15
MR leave−one−out sensitivity analysis for
'ebi−a−GCST90001484' on 'outcome'

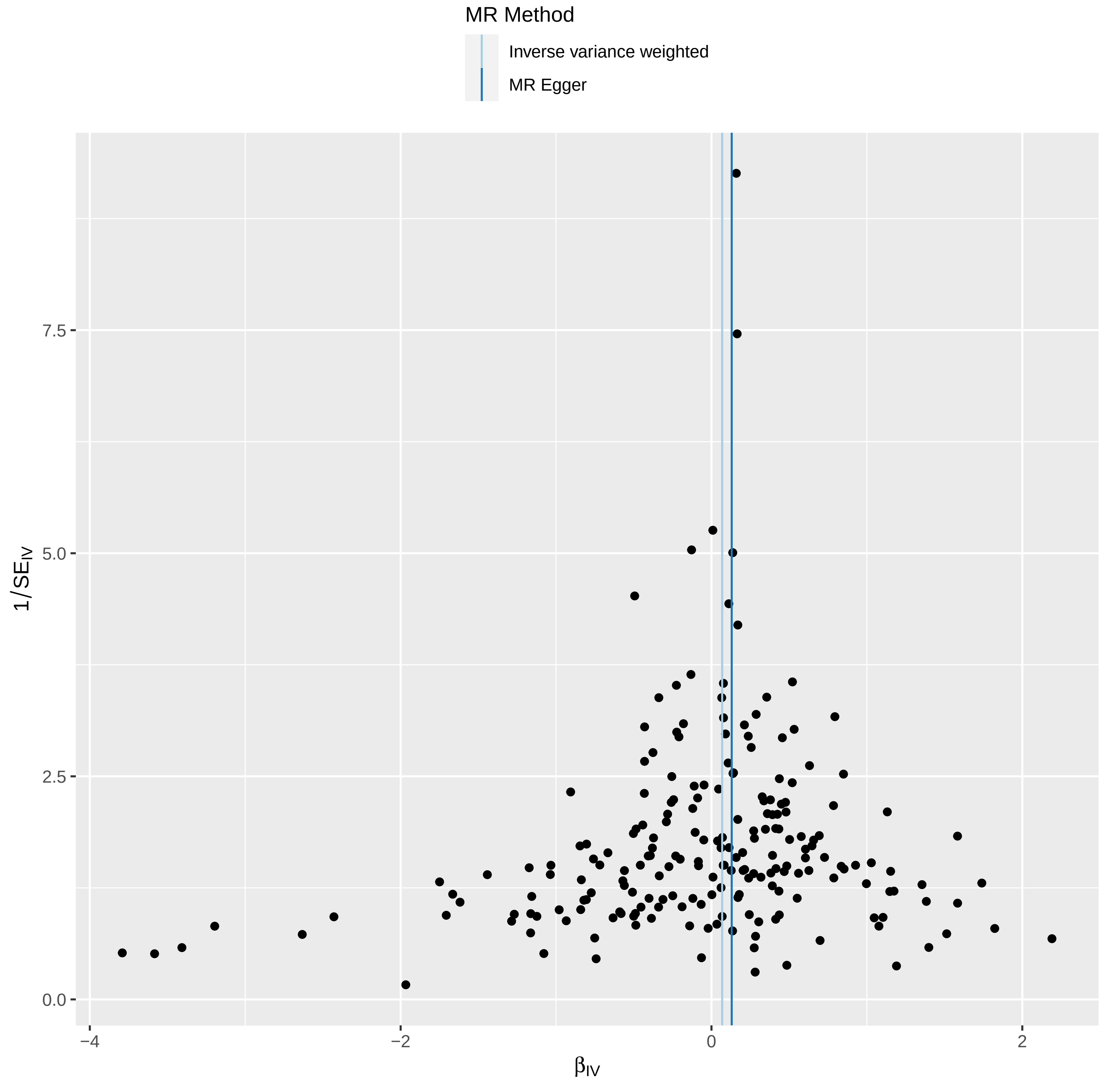

MR Method
Inverse variance weighted
MR Egger
1/SE_IV
7.5
5.0
2.5
0.0
−4
−2
0
2
β_IV

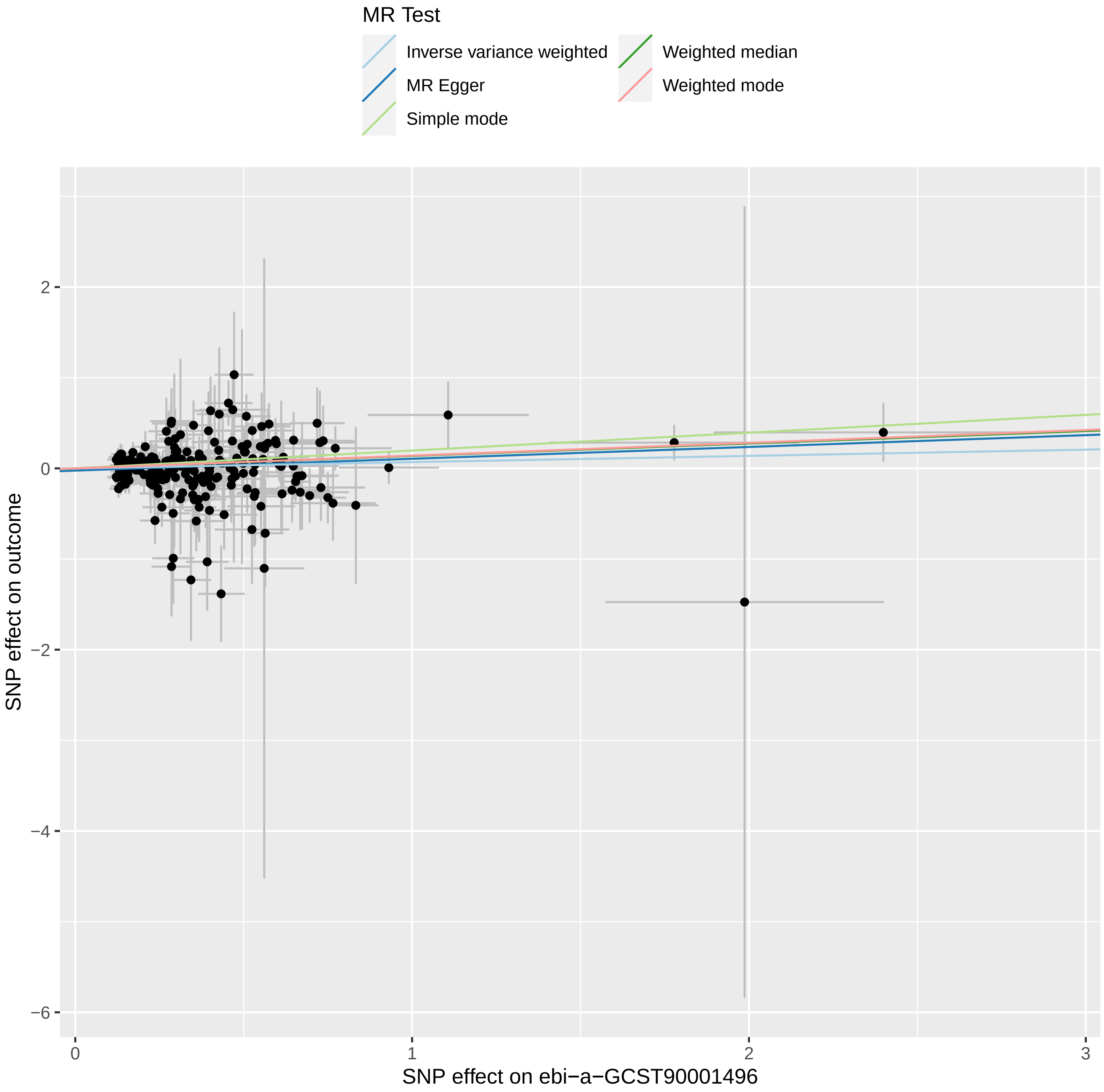

MR Test
Inverse variance weighted
MR Egger
Simple mode
Weighted median
Weighted mode
SNP effect on outcome
SNP effect on ebi−a−GCST90001496
2
0
−2
−4
−6
0
1
2
3

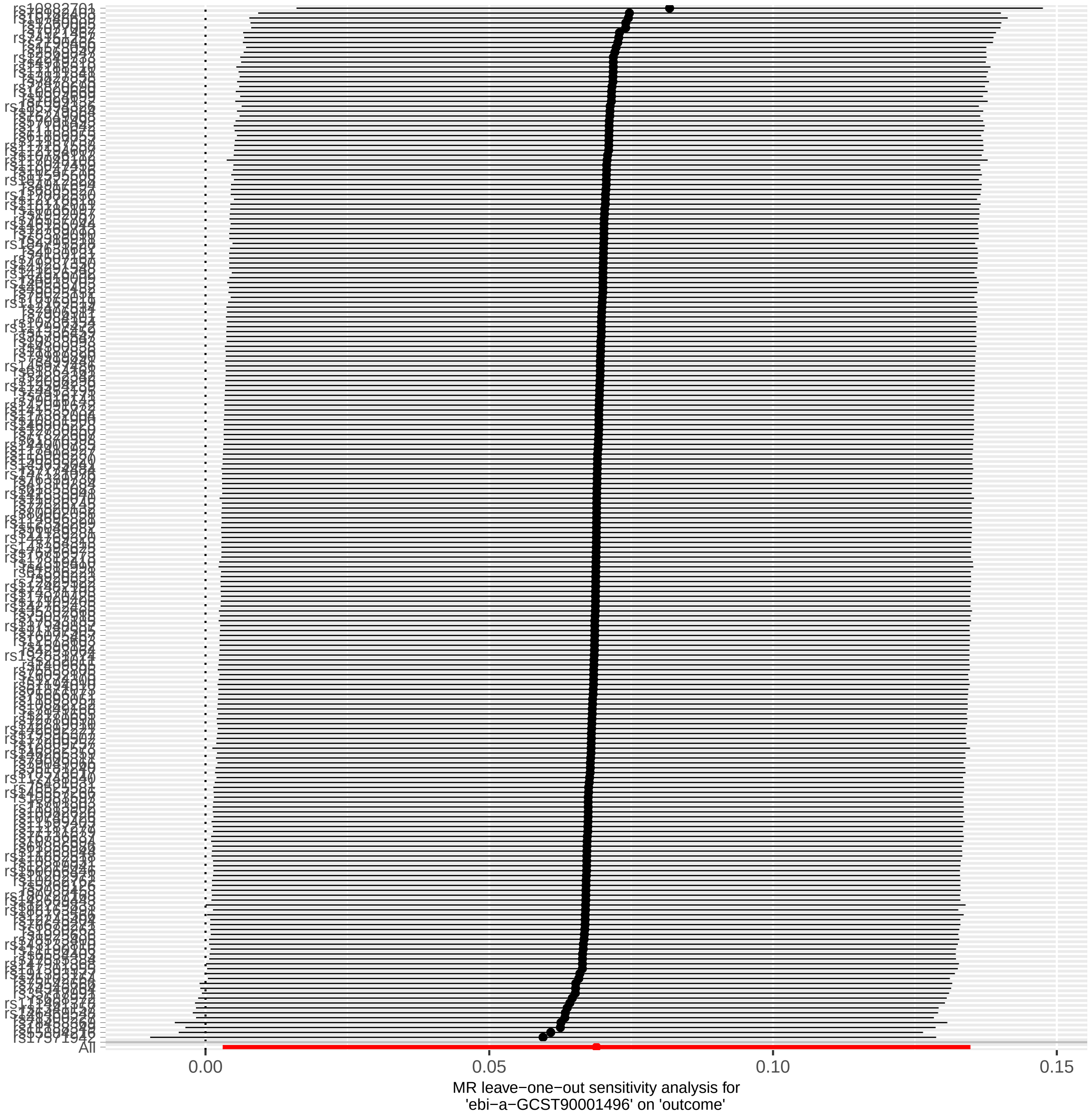

All
0.00
0.05
0.10
0.15
MR leave−one−out sensitivity analysis for
'ebi−a−GCST90001496' on 'outcome'

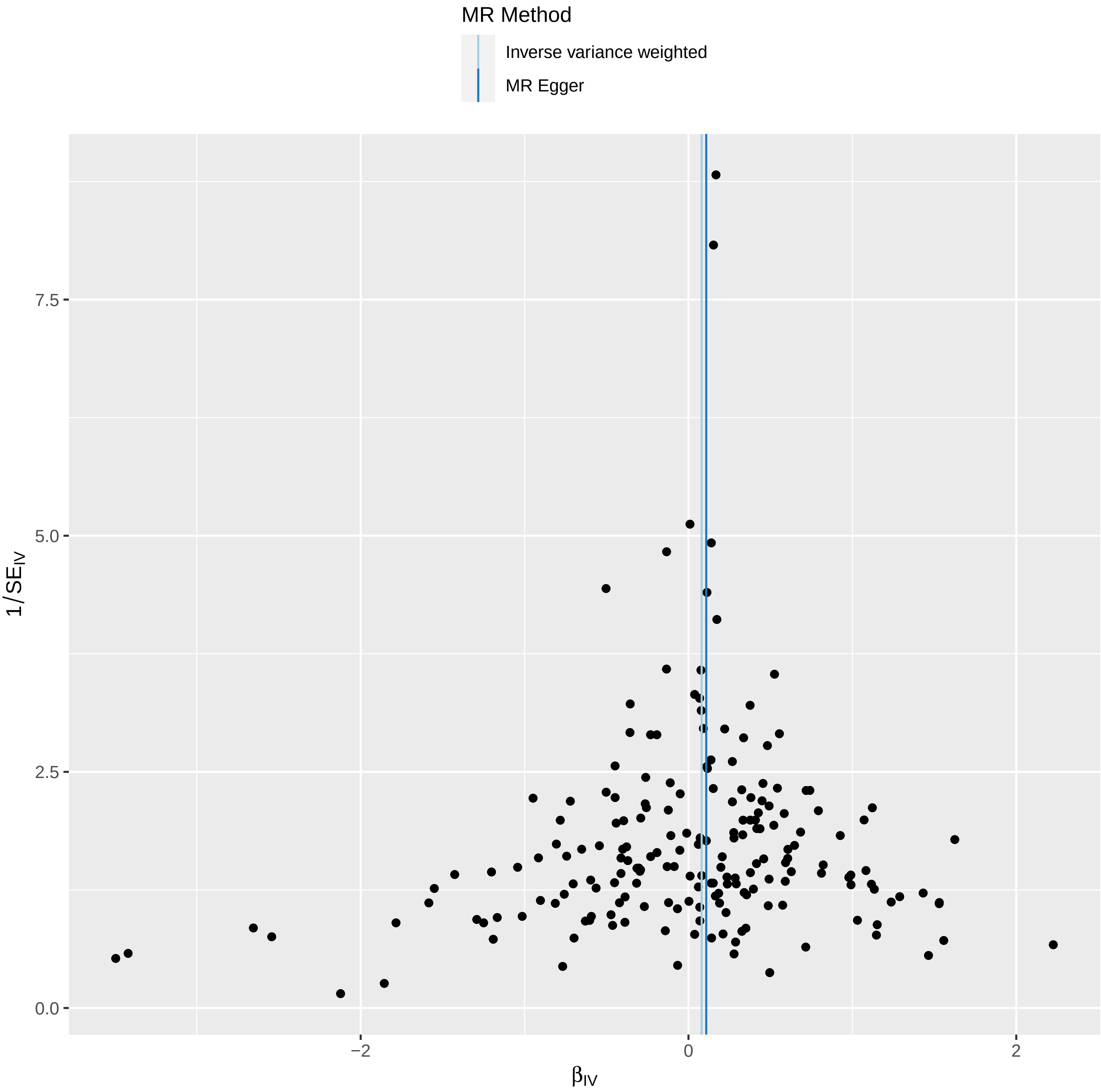
MR Method
Inverse variance weighted
MR Egger
1/SE_IV
7.5
5.0
2.5
0.0
−2
0
2
β_IV

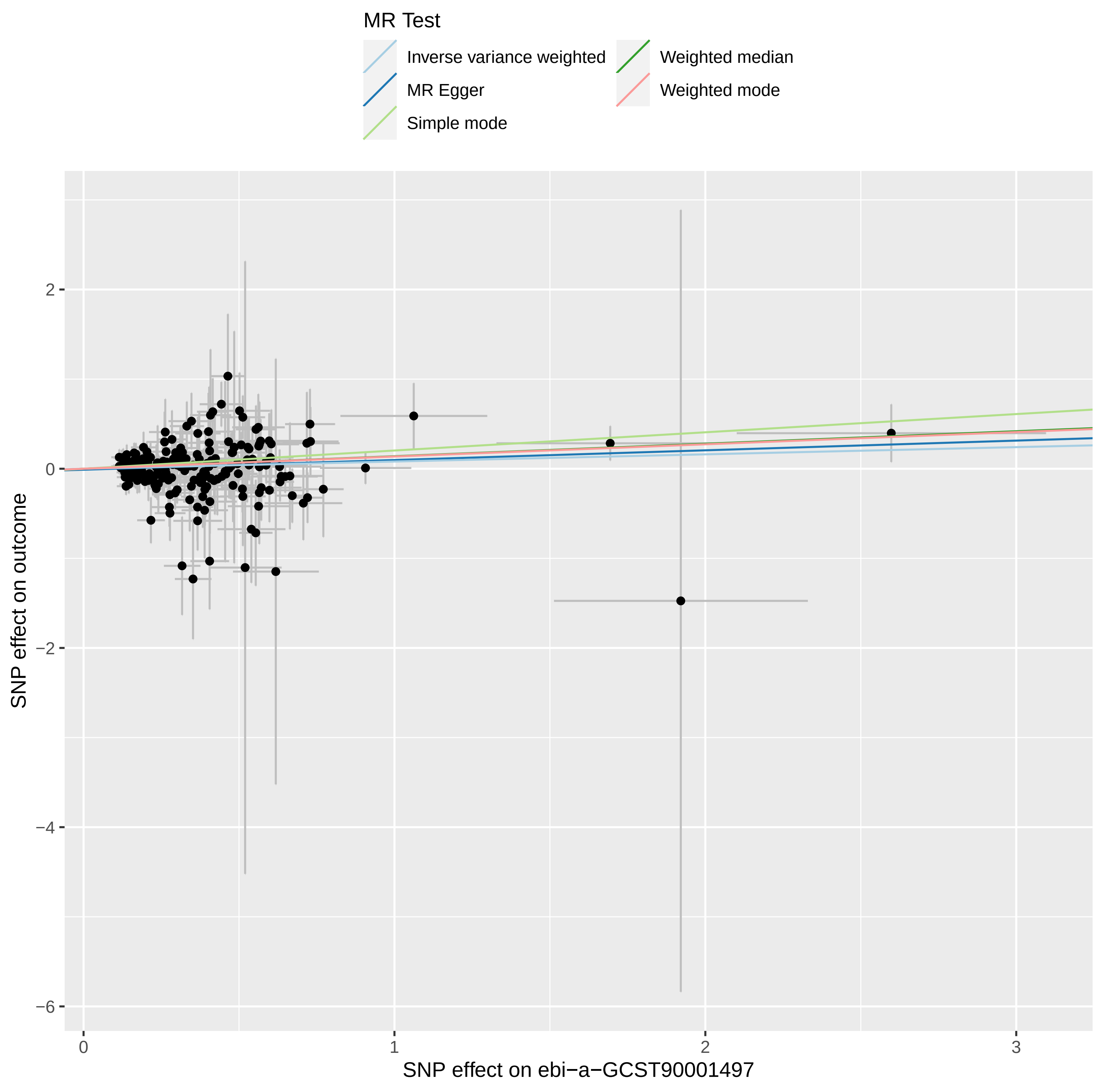
MR Test
Inverse variance weighted
MR Egger
Simple mode
Weighted median
Weighted mode
SNP effect on outcome
2
0
−2
−4
−6
0
1
2
3
SNP effect on ebi−a−GCST90001497

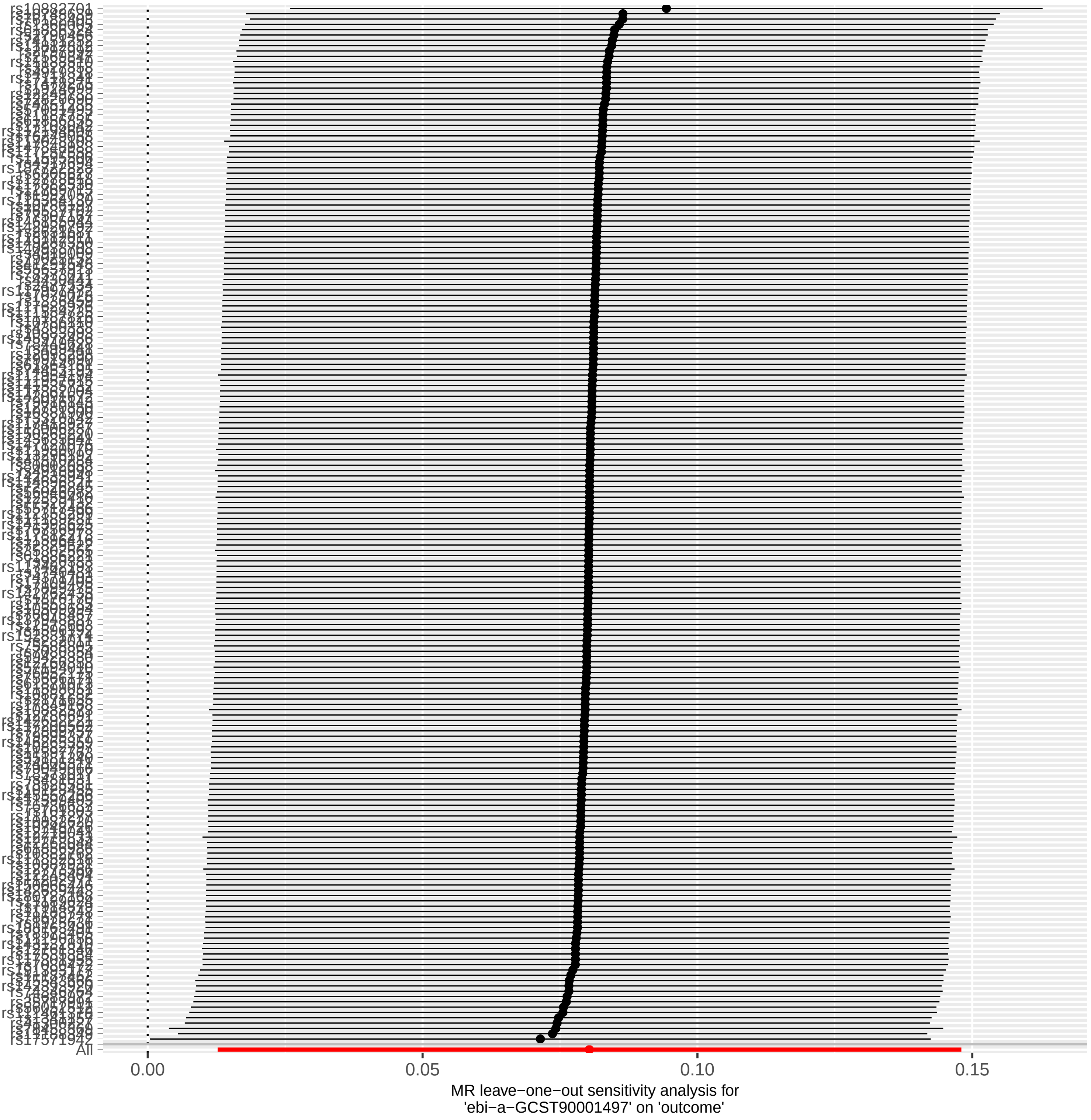

rs10882701
rs17571942
All
0.00
0.05
0.10
0.15
MR leave−one−out sensitivity analysis for
'ebi−a−GCST90001497' on 'outcome'

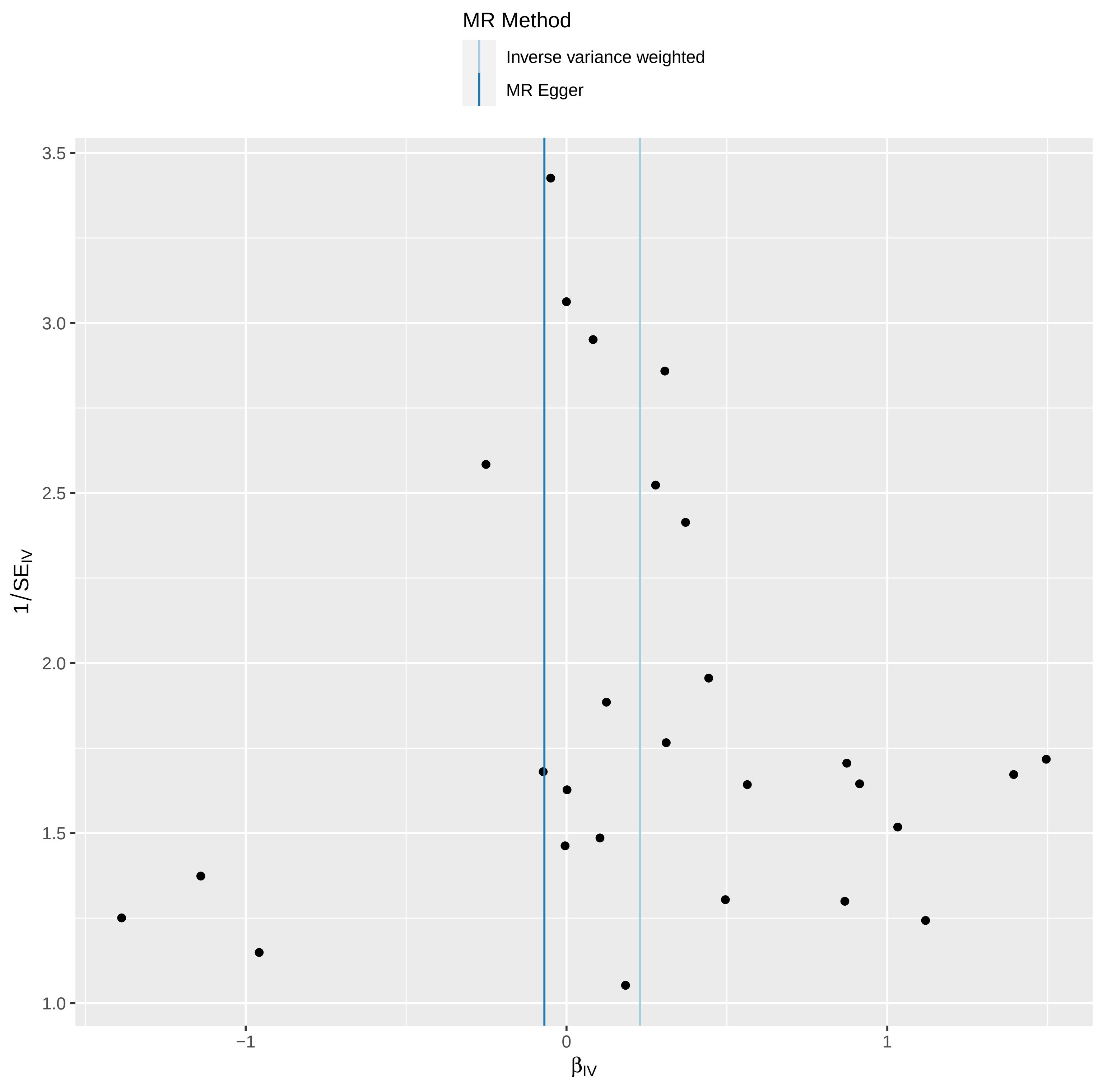

MR Method
Inverse variance weighted
MR Egger
3.5
3.0
2.5
2.0
1.5
1.0
1/SE_IV
−1
0
1
β_IV

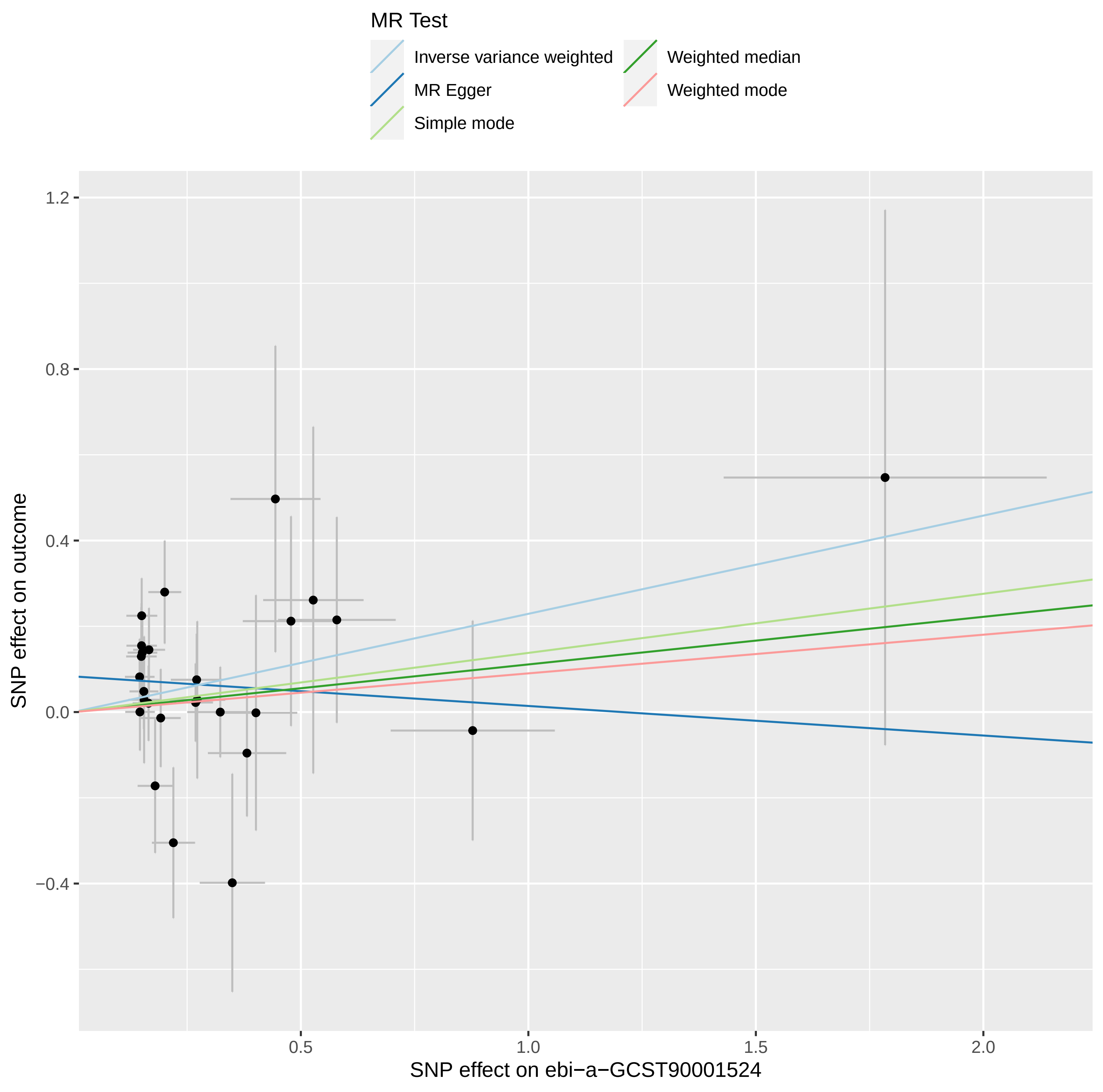
MR Test
Inverse variance weighted
MR Egger
Simple mode
Weighted median
Weighted mode
1.2
0.8
0.4
0.0
−0.4
0.5
1.0
1.5
2.0
SNP effect on outcome
SNP effect on ebi−a−GCST90001524

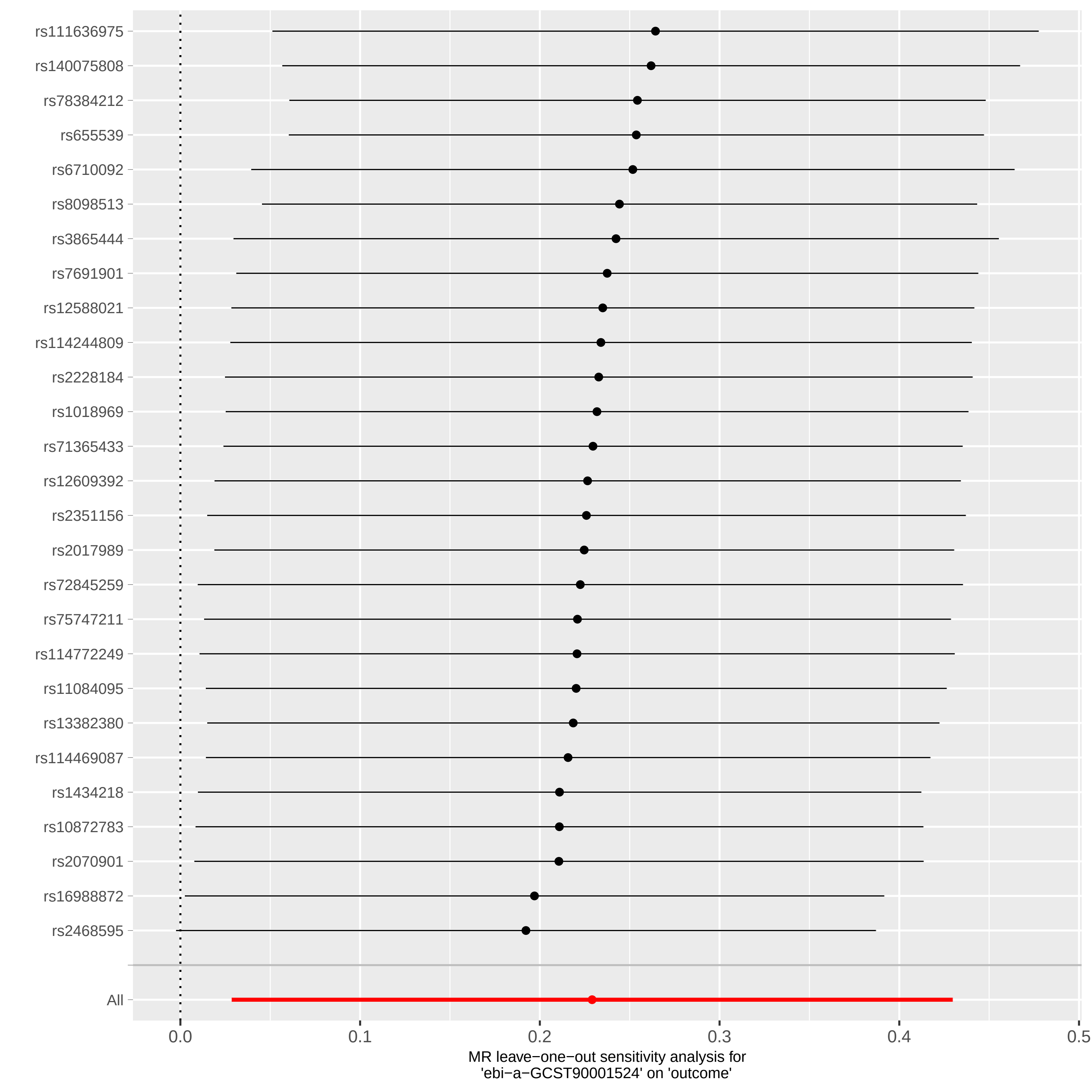
rs111636975
rs140075808
rs78384212
rs655539
rs6710092
rs8098513
rs3865444
rs7691901
rs12588021
rs114244809
rs2228184
rs1018969
rs71365433
rs12609392
rs2351156
rs2017989
rs72845259
rs75747211
rs114772249
rs11084095
rs13382380
rs114469087
rs1434218
rs10872783
rs2070901
rs16988872
rs2468595
All
0.0
0.1
0.2
0.3
0.4
0.5
MR leave−one−out sensitivity analysis for
'ebi−a−GCST90001524' on 'outcome'

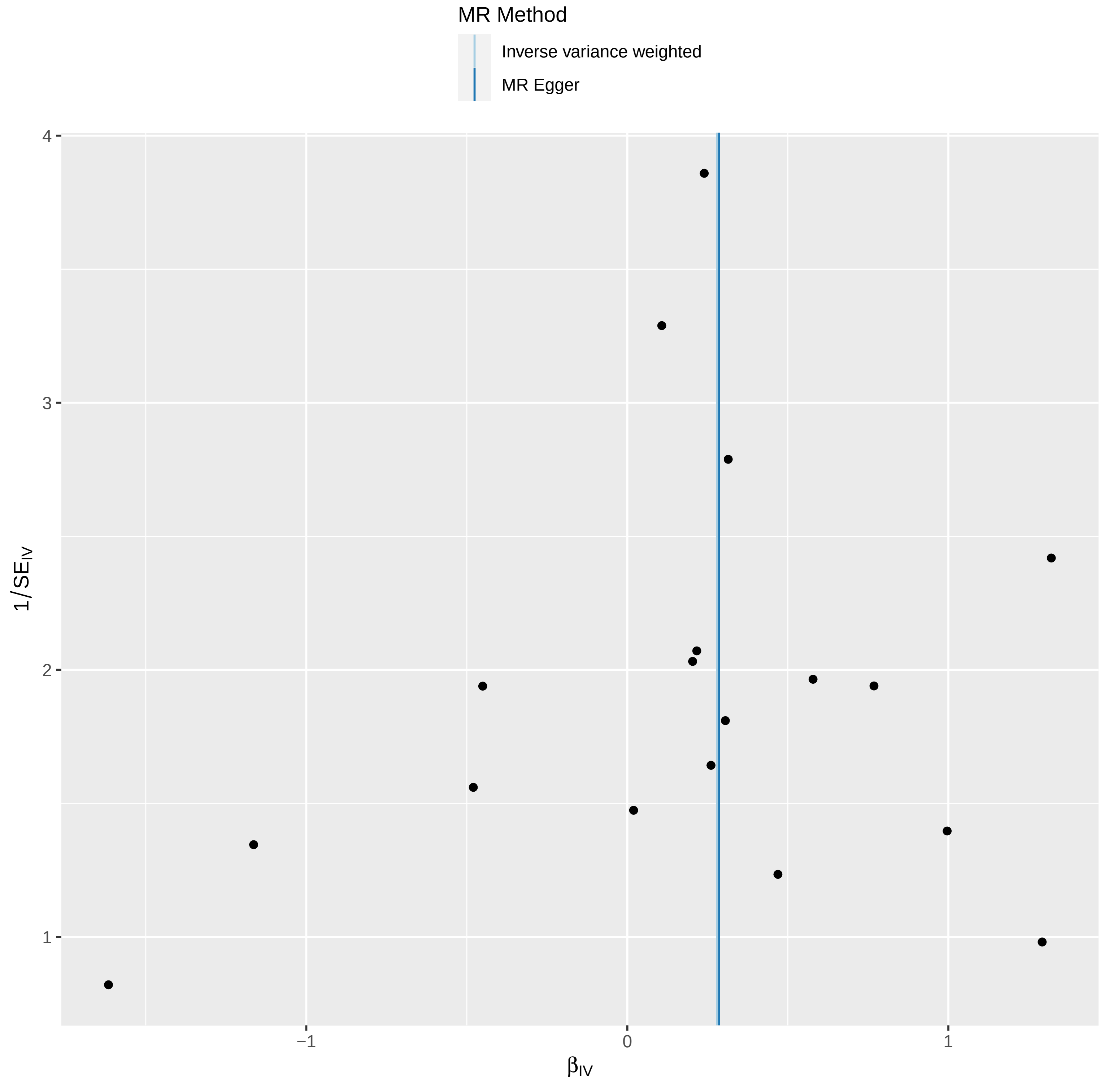
MR Method
Inverse variance weighted
MR Egger
4
3
2
1
1/SE$_{IV}$
−1
0
1
β$_{IV}$

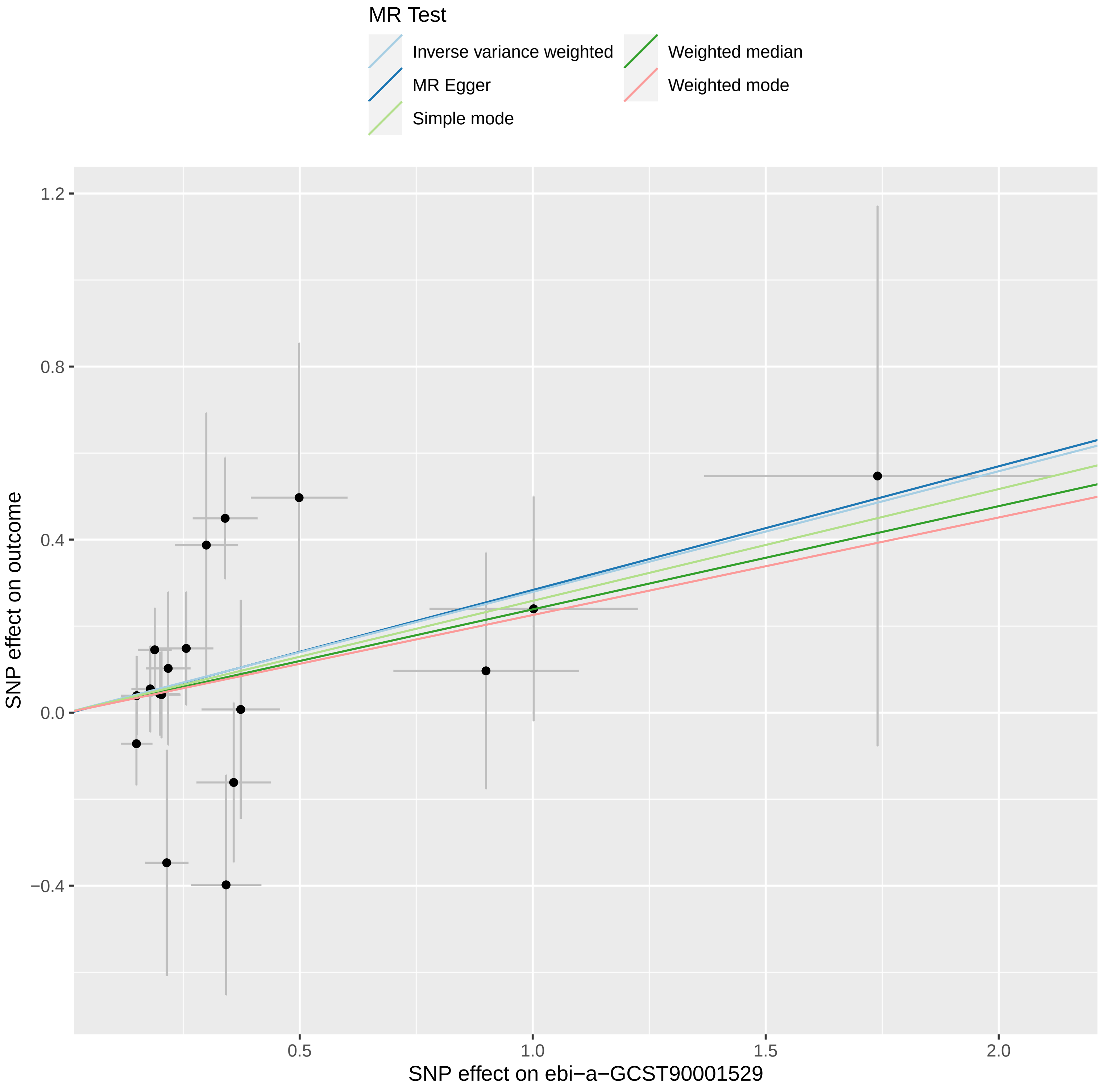

MR Test
Inverse variance weighted
MR Egger
Simple mode
Weighted median
Weighted mode
1.2
0.8
0.4
0.0
−0.4
0.5
1.0
1.5
2.0
SNP effect on outcome
SNP effect on ebi−a−GCST90001529

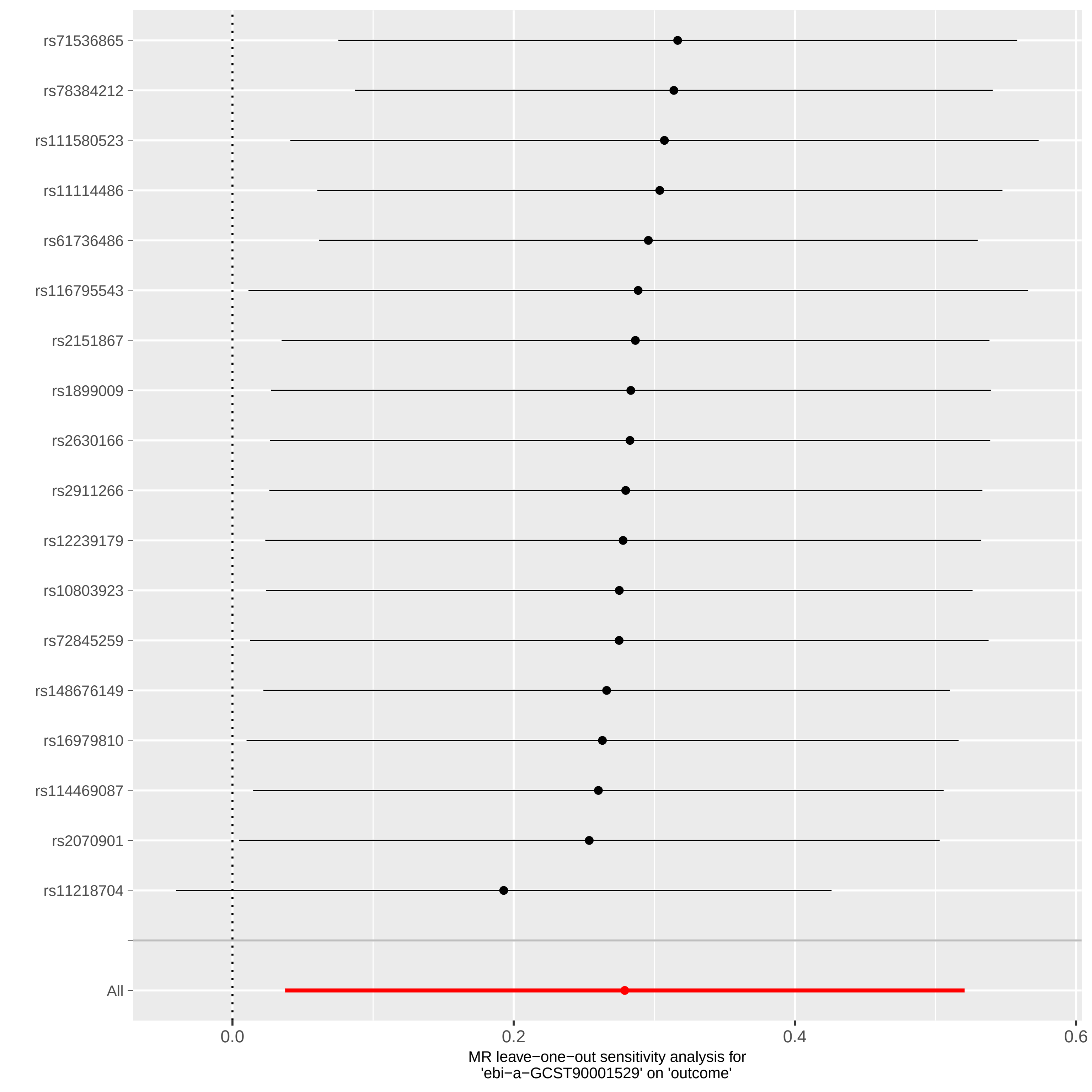
rs71536865
rs78384212
rs111580523
rs11114486
rs61736486
rs116795543
rs2151867
rs1899009
rs2630166
rs2911266
rs12239179
rs10803923
rs72845259
rs148676149
rs16979810
rs114469087
rs2070901
rs11218704
All
0.0
0.2
0.4
0.6
MR leave−one−out sensitivity analysis for
'ebi−a−GCST90001529' on 'outcome'

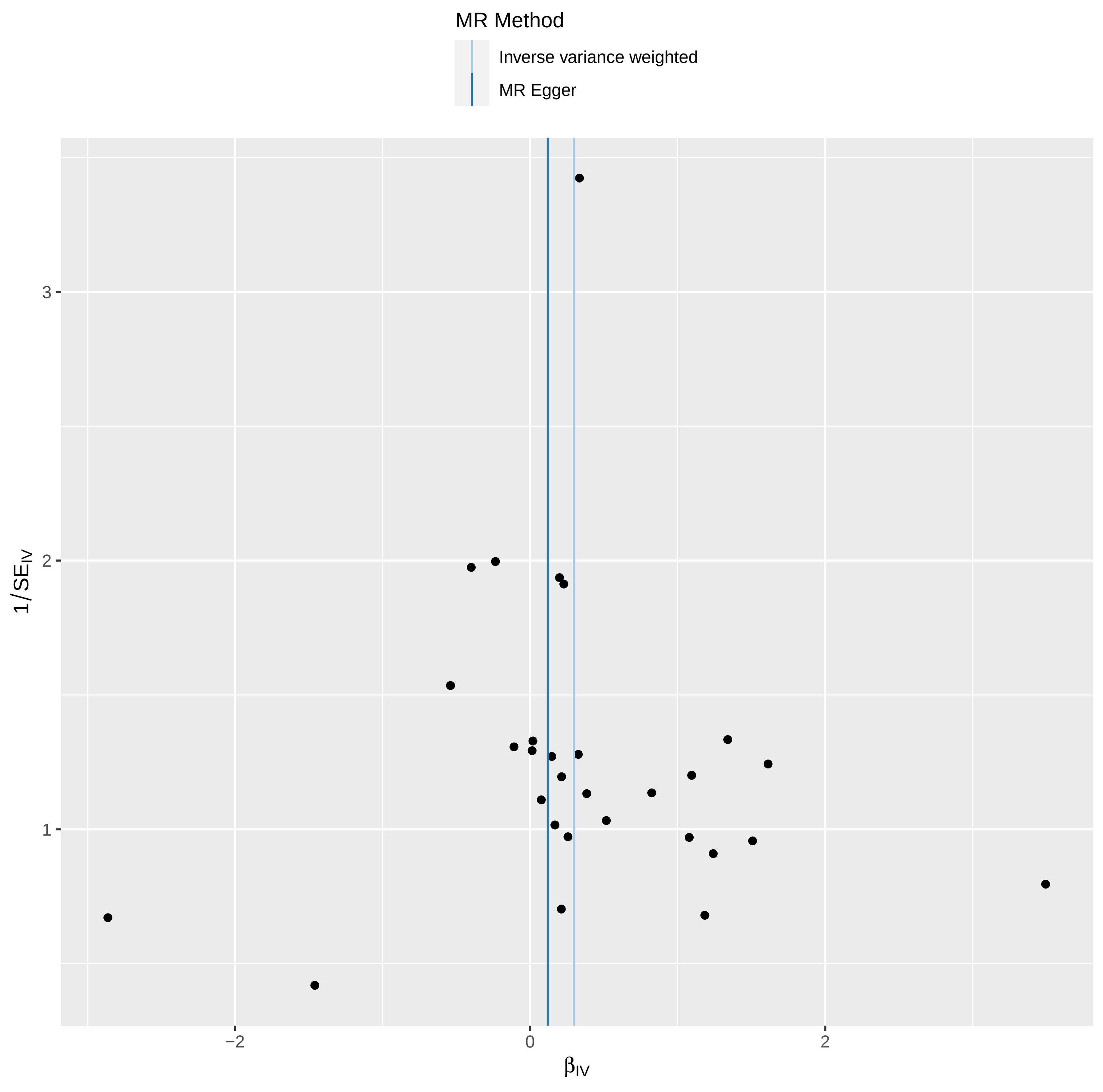

MR Method
Inverse variance weighted
MR Egger
3
2
1
1/SE_IV
−2
0
2
β_IV

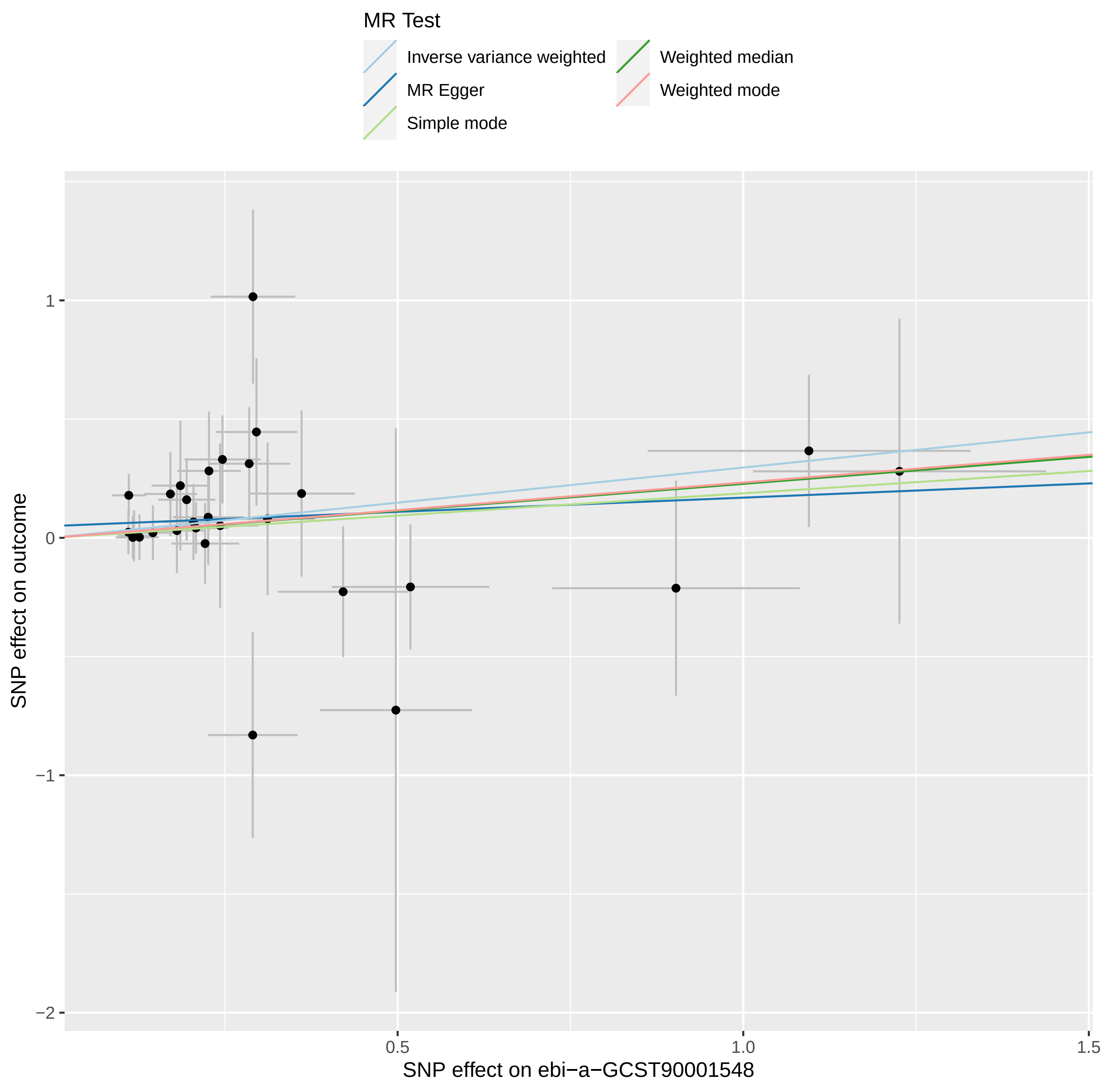

MR Test
Inverse variance weighted
MR Egger
Simple mode
Weighted median
Weighted mode
SNP effect on outcome
1
0
−1
−2
0.5
1.0
1.5
SNP effect on ebi−a−GCST90001548

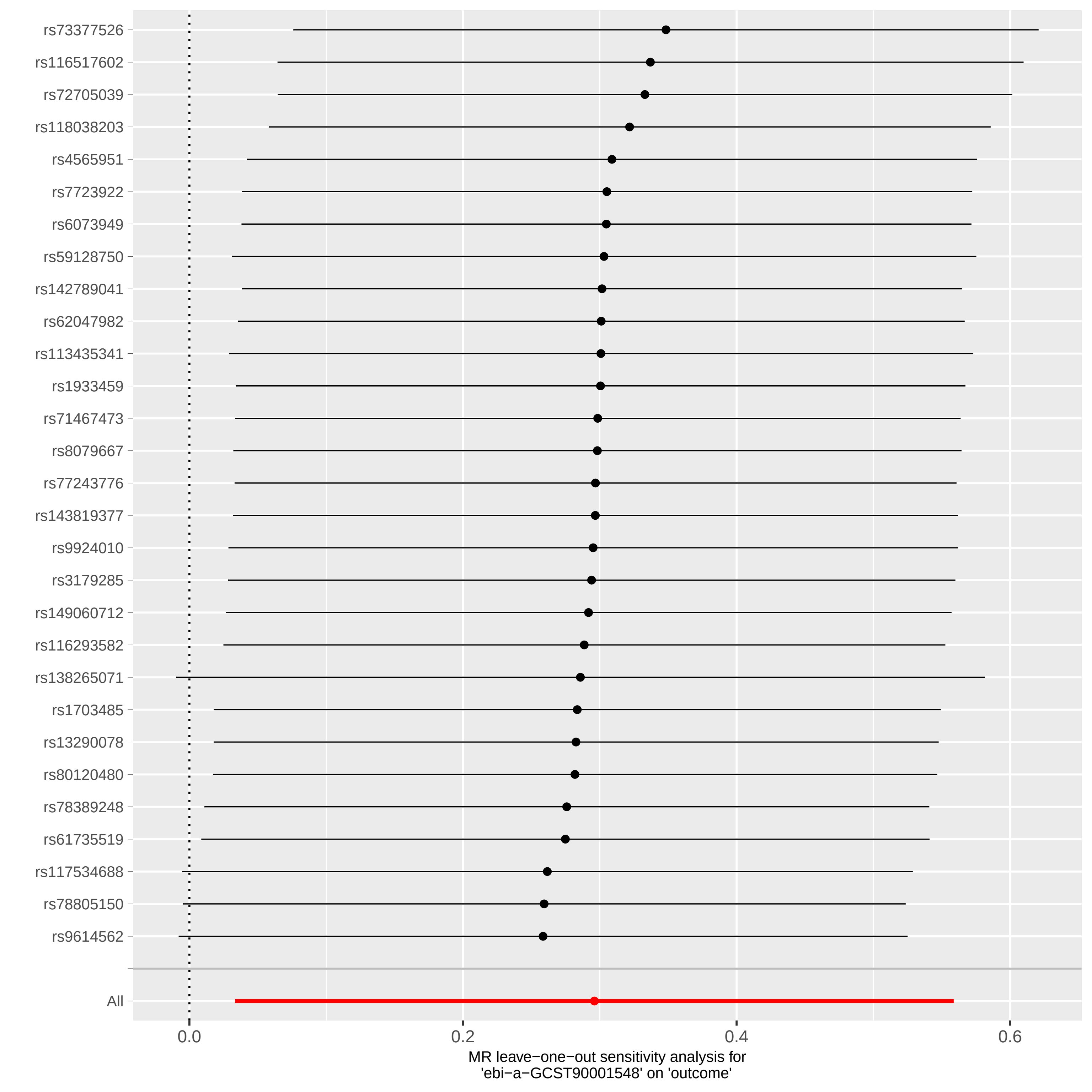
rs73377526
rs116517602
rs72705039
rs118038203
rs4565951
rs7723922
rs6073949
rs59128750
rs142789041
rs62047982
rs113435341
rs1933459
rs71467473
rs8079667
rs77243776
rs143819377
rs9924010
rs3179285
rs149060712
rs116293582
rs138265071
rs1703485
rs13290078
rs80120480
rs78389248
rs61735519
rs117534688
rs78805150
rs9614562
All
0.0
0.2
0.4
0.6
MR leave−one−out sensitivity analysis for
'ebi−a−GCST90001548' on 'outcome'

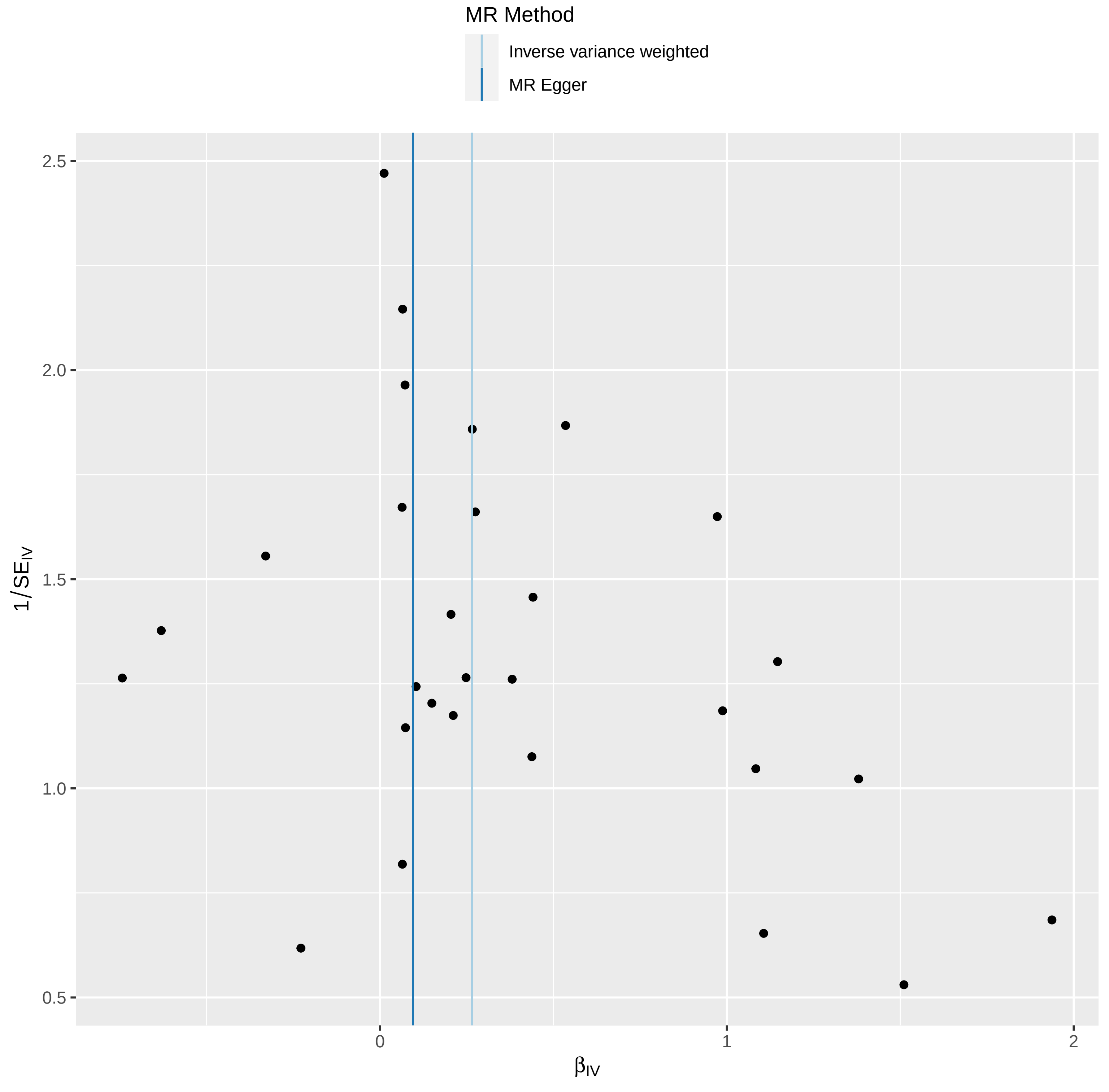
MR Method
Inverse variance weighted
MR Egger
2.5
2.0
1.5
1.0
0.5
1/SE_IV
0
1
2
β_IV

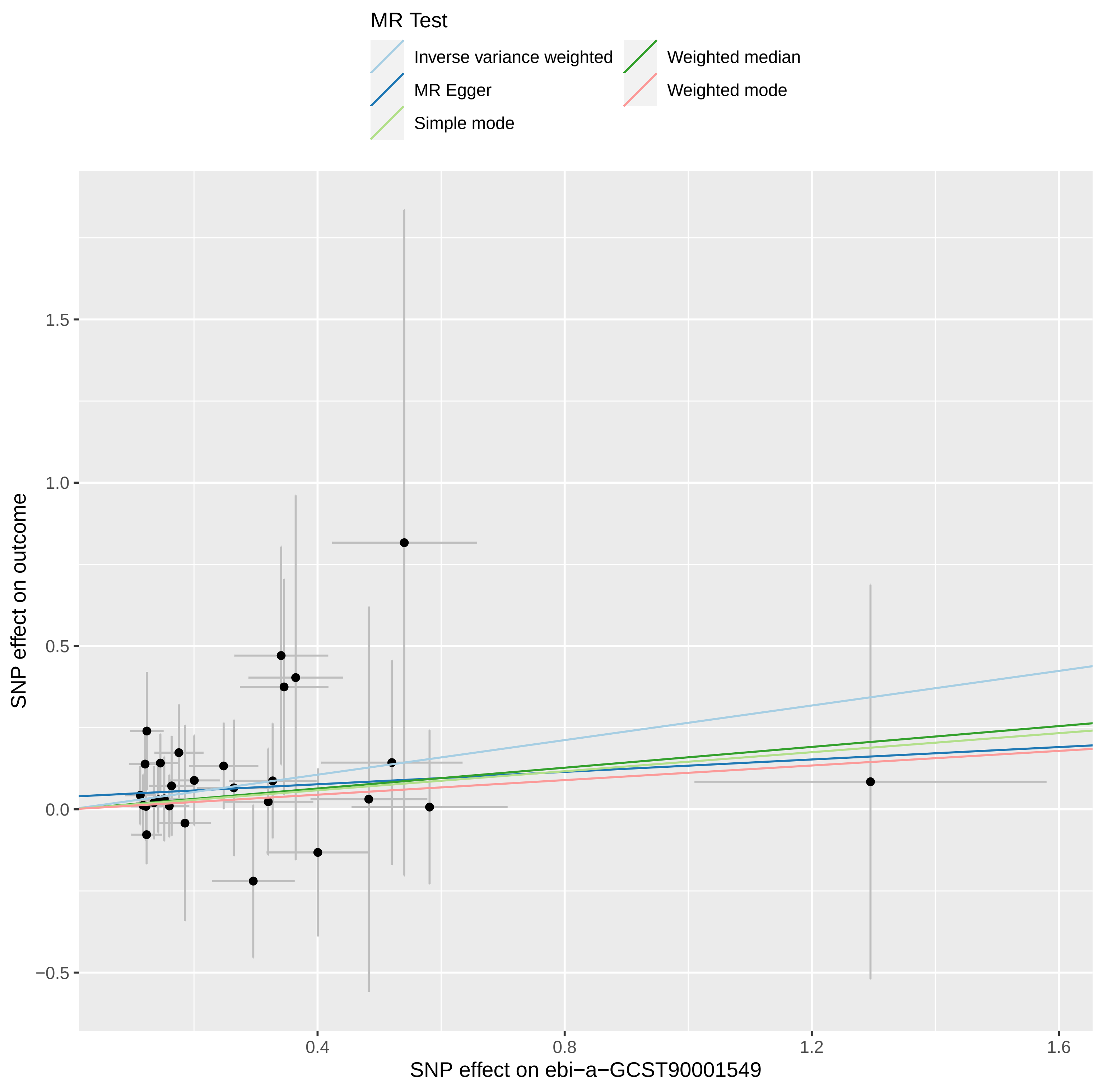
MR Test
Inverse variance weighted
MR Egger
Simple mode
Weighted median
Weighted mode
1.5
1.0
0.5
0.0
−0.5
SNP effect on outcome
0.4
0.8
1.2
1.6
SNP effect on ebi−a−GCST90001549

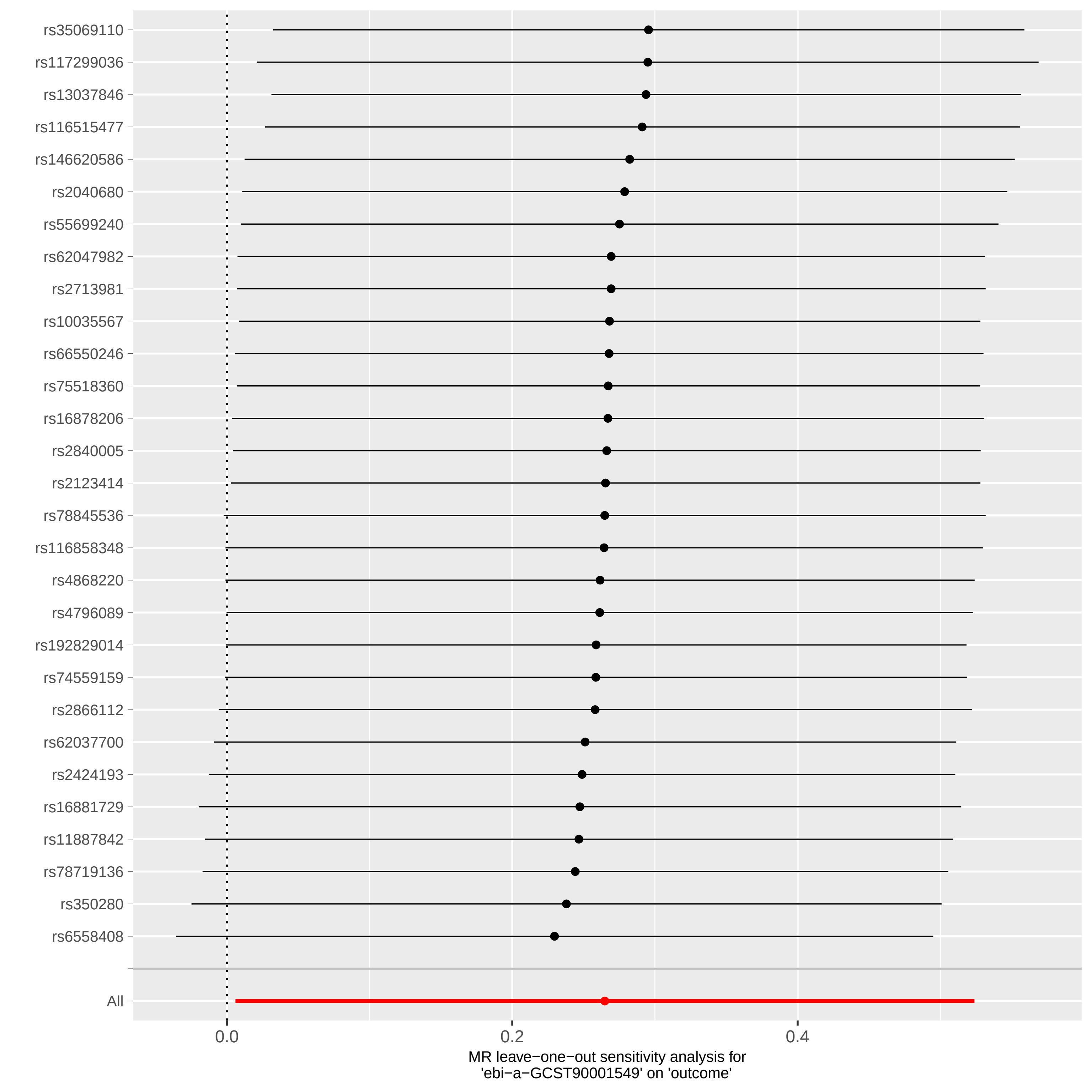
rs35069110
rs117299036
rs13037846
rs116515477
rs146620586
rs2040680
rs55699240
rs62047982
rs2713981
rs10035567
rs66550246
rs75518360
rs16878206
rs2840005
rs2123414
rs78845536
rs116858348
rs4868220
rs4796089
rs192829014
rs74559159
rs2866112
rs62037700
rs2424193
rs16881729
rs11887842
rs78719136
rs350280
rs6558408
All
0.0
0.2
0.4
MR leave−one−out sensitivity analysis for
'ebi−a−GCST90001549' on 'outcome'

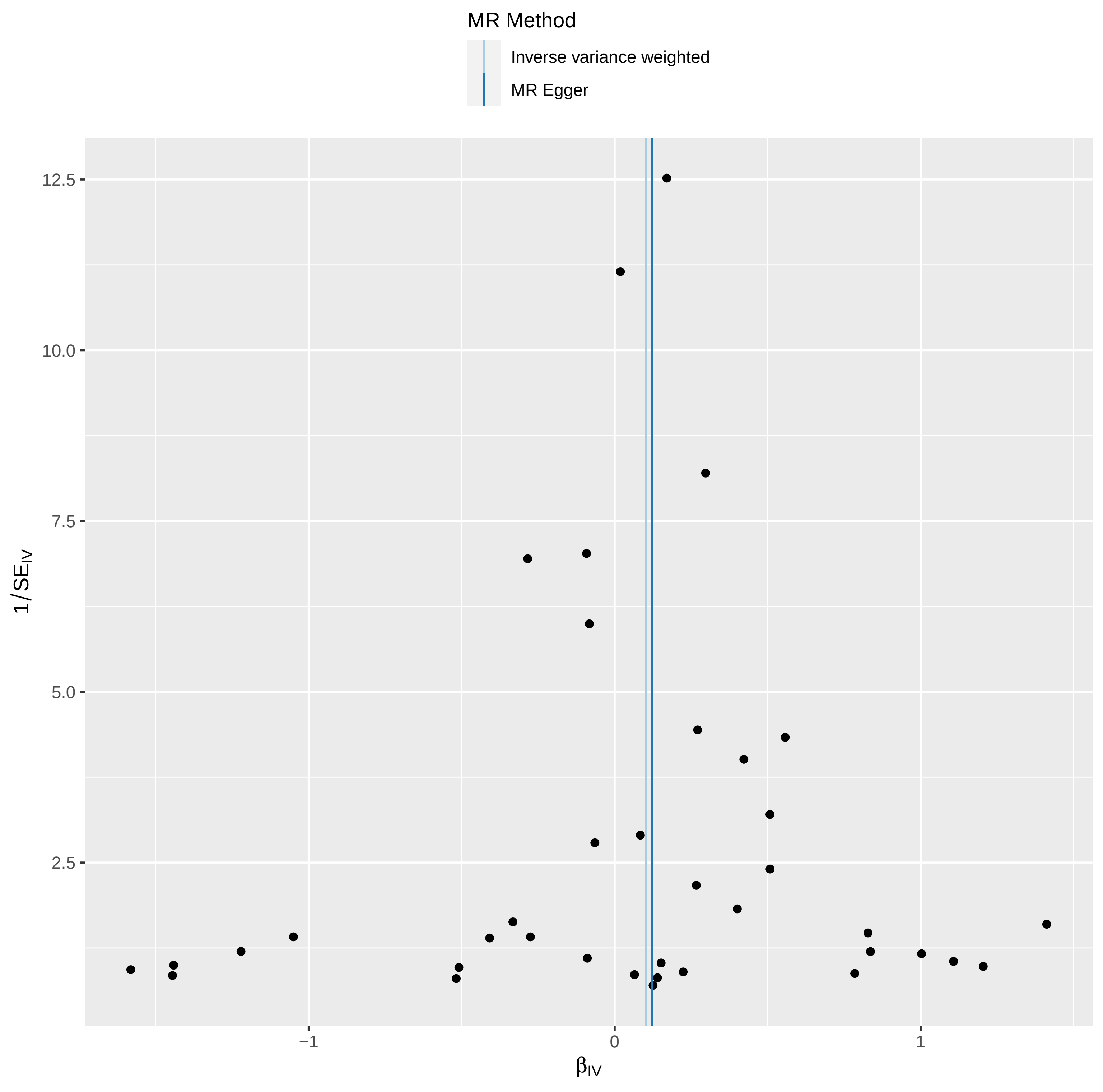
MR Method
Inverse variance weighted
MR Egger
12.5
10.0
7.5
5.0
2.5
−1
0
1
$1/SE_{IV}$
β_{IV}

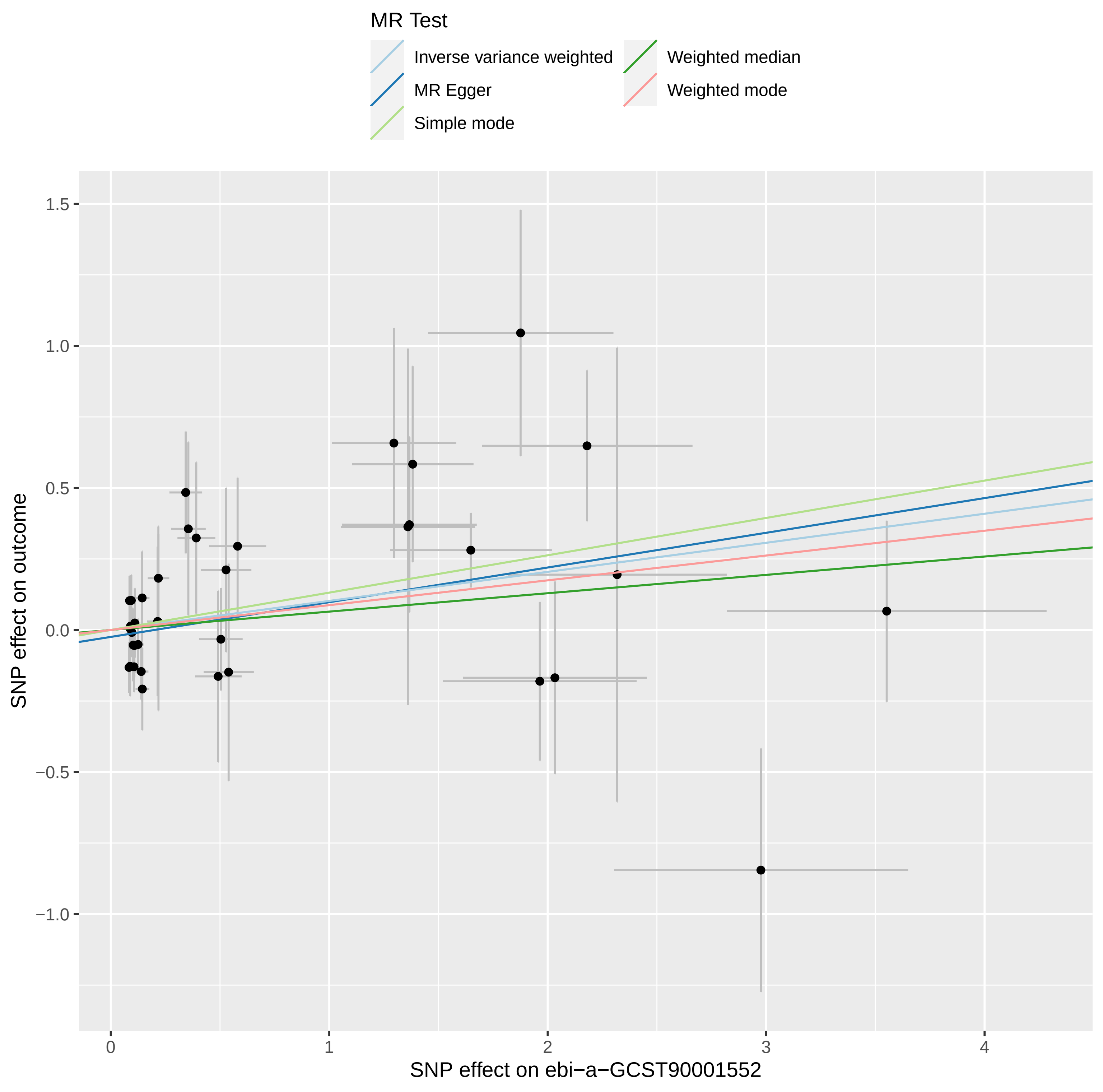

MR Test
Inverse variance weighted
MR Egger
Simple mode
Weighted median
Weighted mode
SNP effect on outcome
1.5
1.0
0.5
0.0
−0.5
−1.0
0
1
2
3
4
SNP effect on ebi−a−GCST90001552

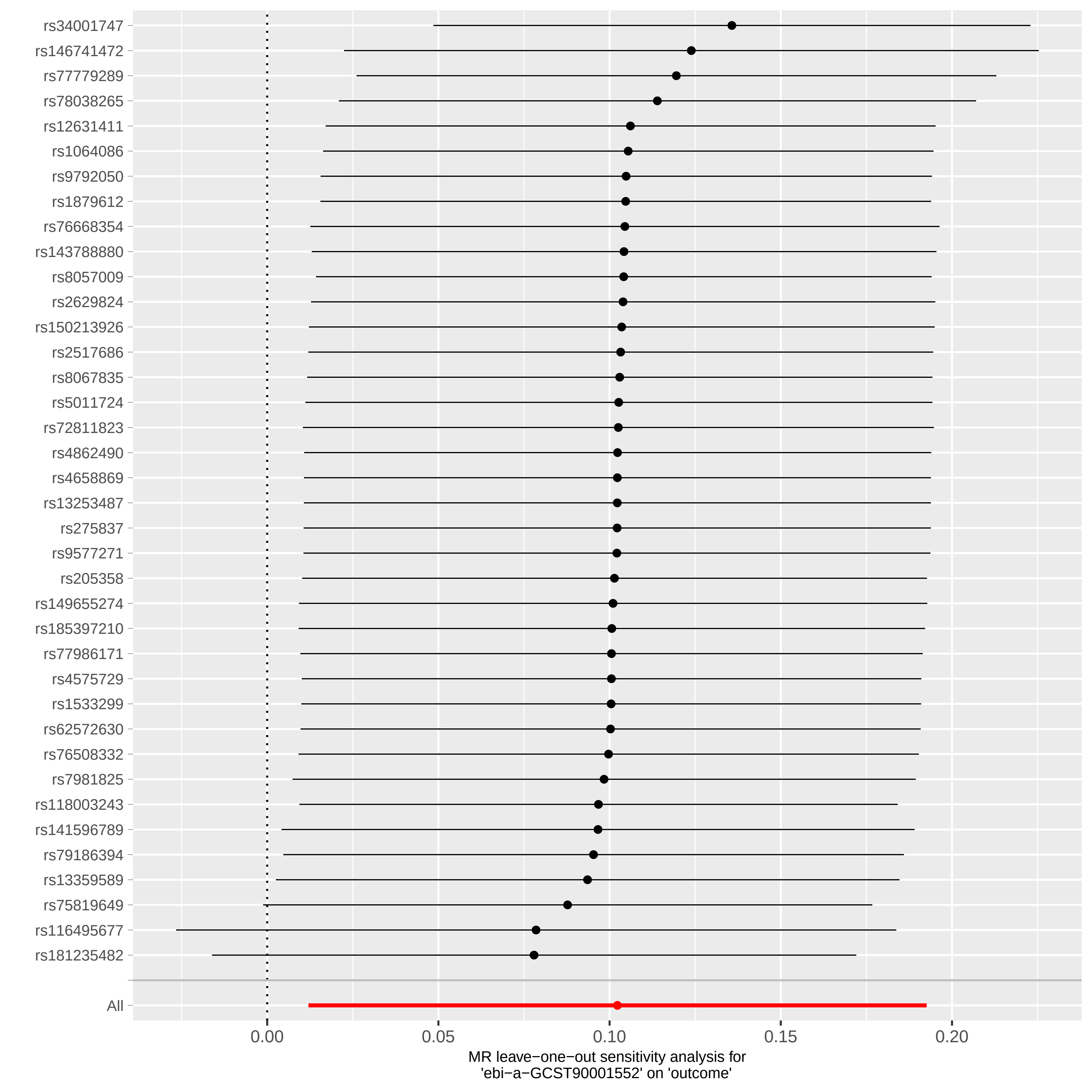

rs34001747
rs146741472
rs77779289
rs78038265
rs12631411
rs1064086
rs9792050
rs1879612
rs76668354
rs143788880
rs8057009
rs2629824
rs150213926
rs2517686
rs8067835
rs5011724
rs72811823
rs4862490
rs4658869
rs13253487
rs275837
rs9577271
rs205358
rs149655274
rs185397210
rs77986171
rs4575729
rs1533299
rs62572630
rs76508332
rs7981825
rs118003243
rs141596789
rs79186394
rs13359589
rs75819649
rs116495677
rs181235482
All
0.00
0.05
0.10
0.15
0.20
MR leave−one−out sensitivity analysis for
'ebi−a−GCST90001552' on 'outcome'

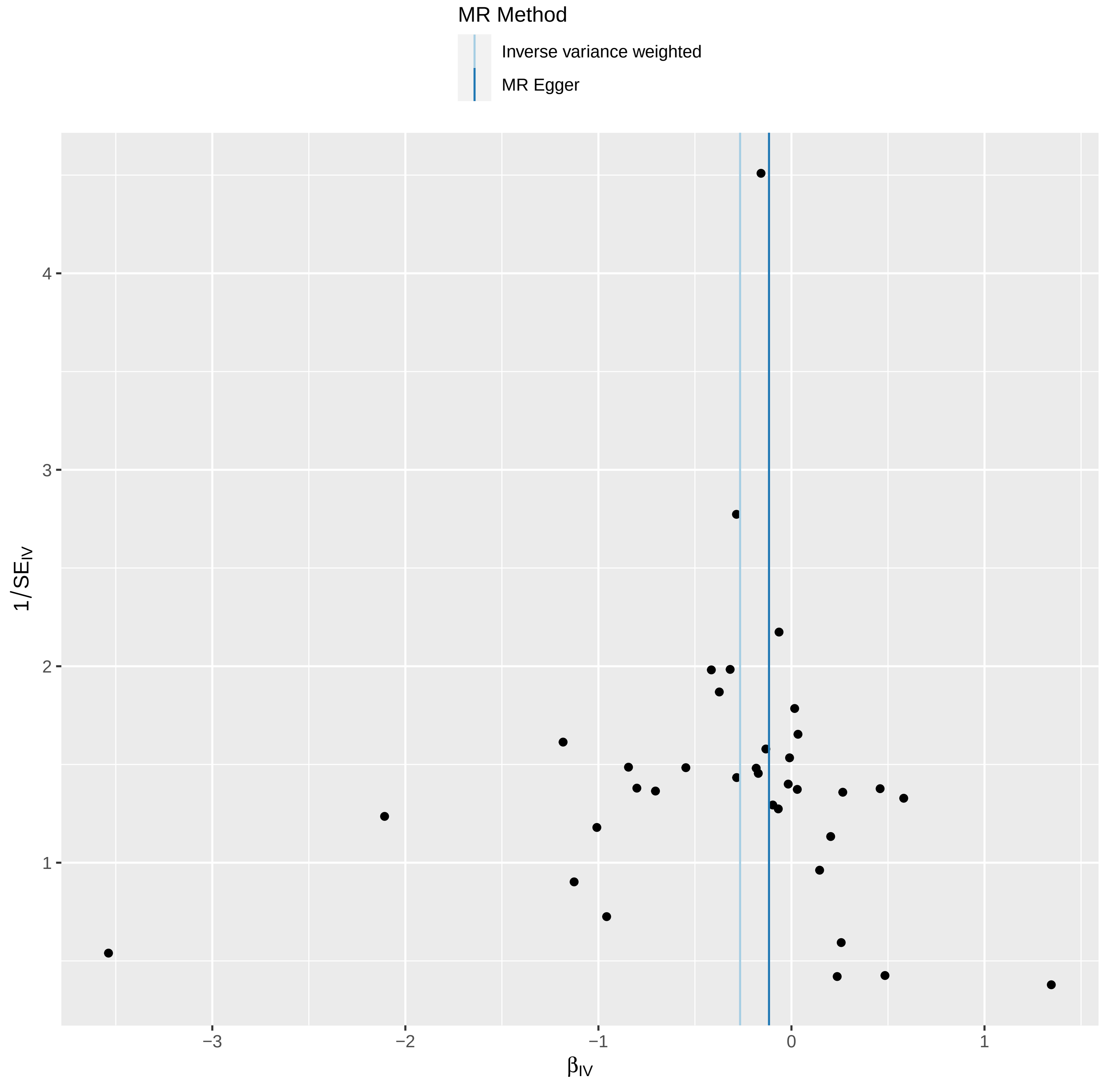

MR Method
Inverse variance weighted
MR Egger
1/SE_IV
β_IV
4
3
2
1
−3
−2
−1
0
1

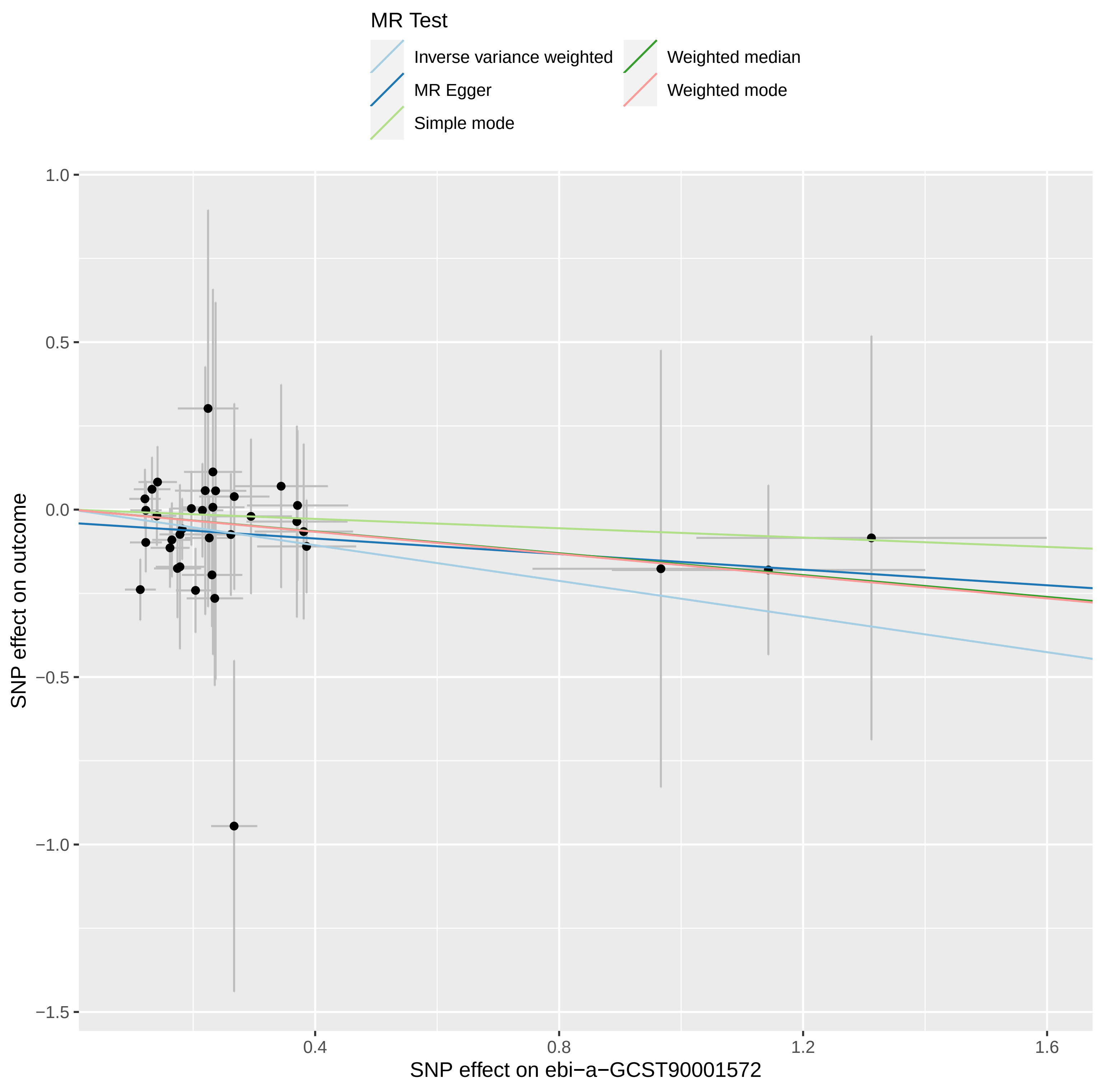
MR Test
Inverse variance weighted
MR Egger
Simple mode
Weighted median
Weighted mode
1.0
0.5
0.0
−0.5
−1.0
−1.5
SNP effect on outcome
0.4
0.8
1.2
1.6
SNP effect on ebi−a−GCST90001572

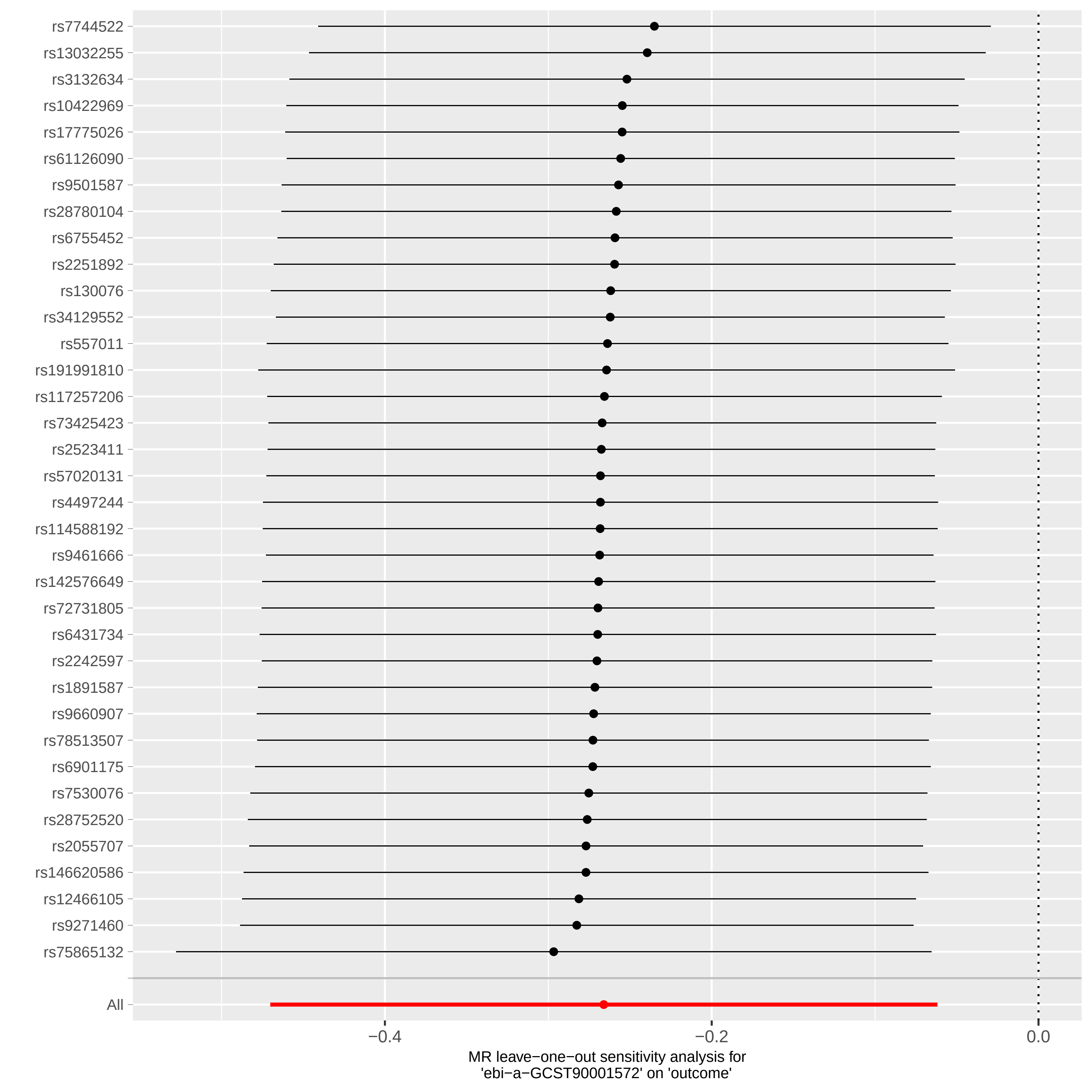
rs7744522
rs13032255
rs3132634
rs10422969
rs17775026
rs61126090
rs9501587
rs28780104
rs6755452
rs2251892
rs130076
rs34129552
rs557011
rs191991810
rs117257206
rs73425423
rs2523411
rs57020131
rs4497244
rs114588192
rs9461666
rs142576649
rs72731805
rs6431734
rs2242597
rs1891587
rs9660907
rs78513507
rs6901175
rs7530076
rs28752520
rs2055707
rs146620586
rs12466105
rs9271460
rs75865132
All
−0.4
−0.2
0.0
MR leave−one−out sensitivity analysis for
'ebi−a−GCST90001572' on 'outcome'

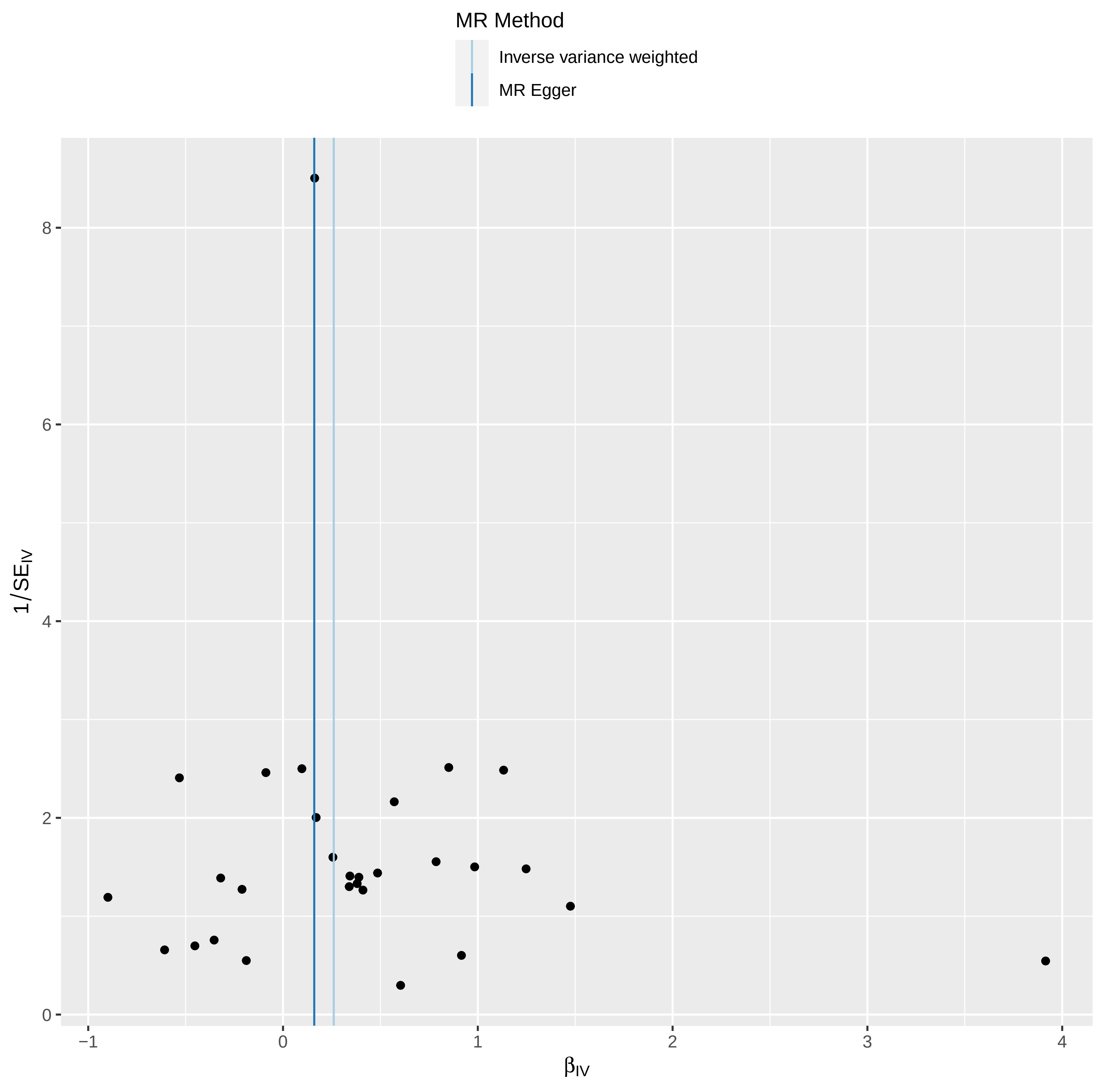

MR Method
Inverse variance weighted
MR Egger
8
6
4
2
0
1/SE$_{IV}$
−1
0
1
2
3
4
β$_{IV}$

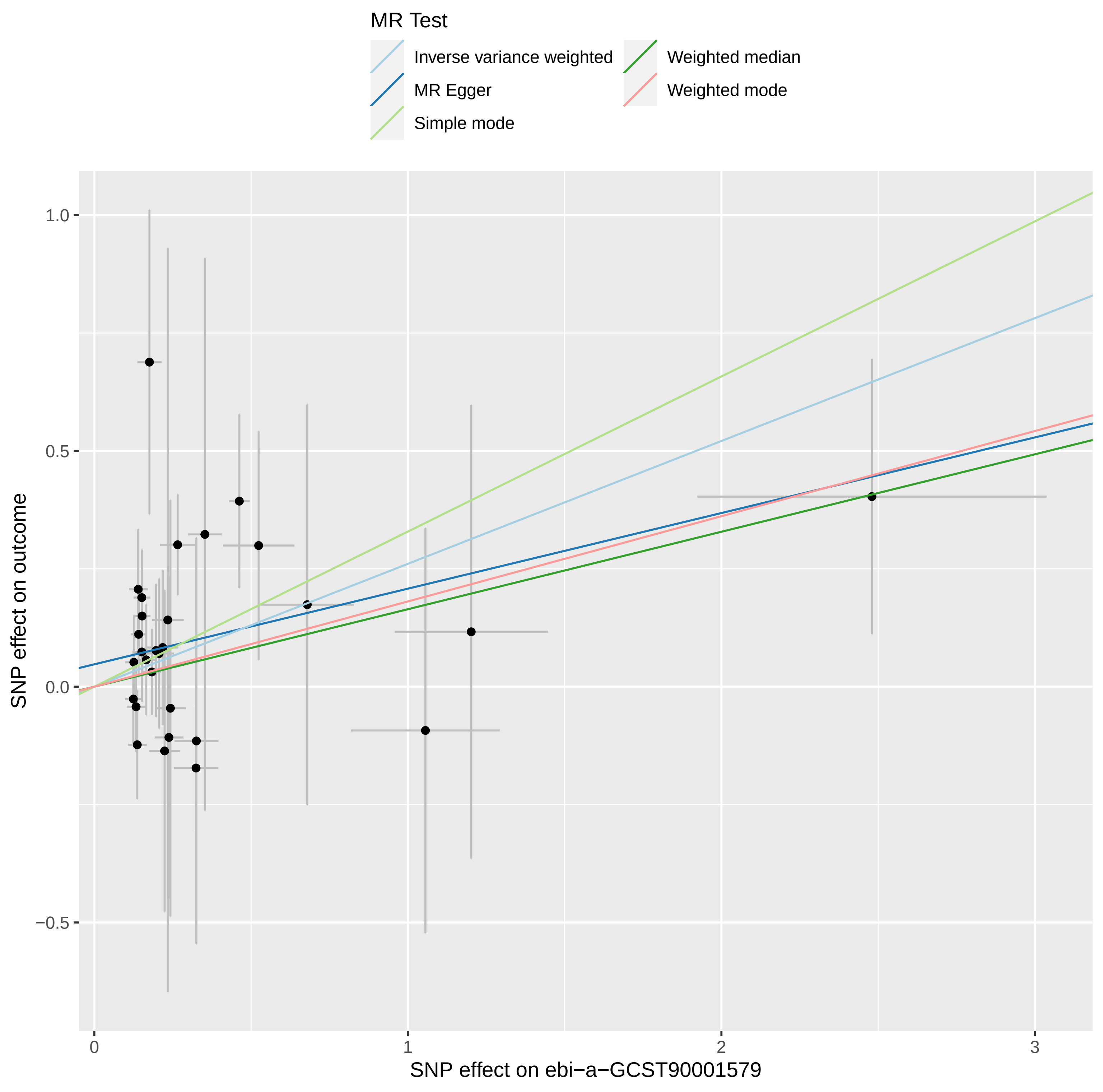
MR Test
Inverse variance weighted
MR Egger
Simple mode
Weighted median
Weighted mode
1.0
0.5
0.0
−0.5
SNP effect on outcome
0
1
2
3
SNP effect on ebi−a−GCST90001579

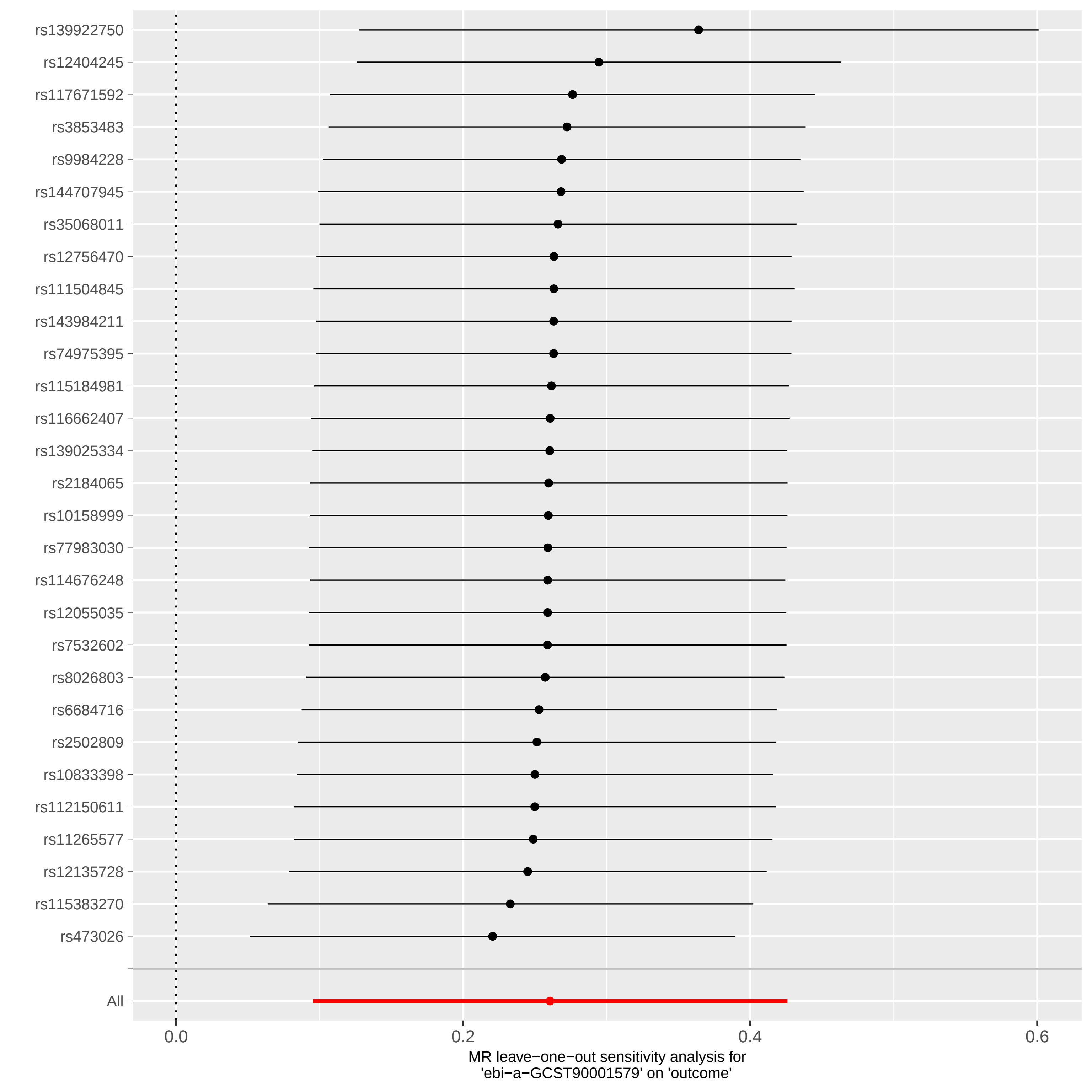

rs139922750
rs12404245
rs117671592
rs3853483
rs9984228
rs144707945
rs35068011
rs12756470
rs111504845
rs143984211
rs74975395
rs115184981
rs116662407
rs139025334
rs2184065
rs10158999
rs77983030
rs114676248
rs12055035
rs7532602
rs8026803
rs6684716
rs2502809
rs10833398
rs112150611
rs11265577
rs12135728
rs115383270
rs473026
All
0.0
0.2
0.4
0.6
MR leave−one−out sensitivity analysis for
'ebi−a−GCST90001579' on 'outcome'

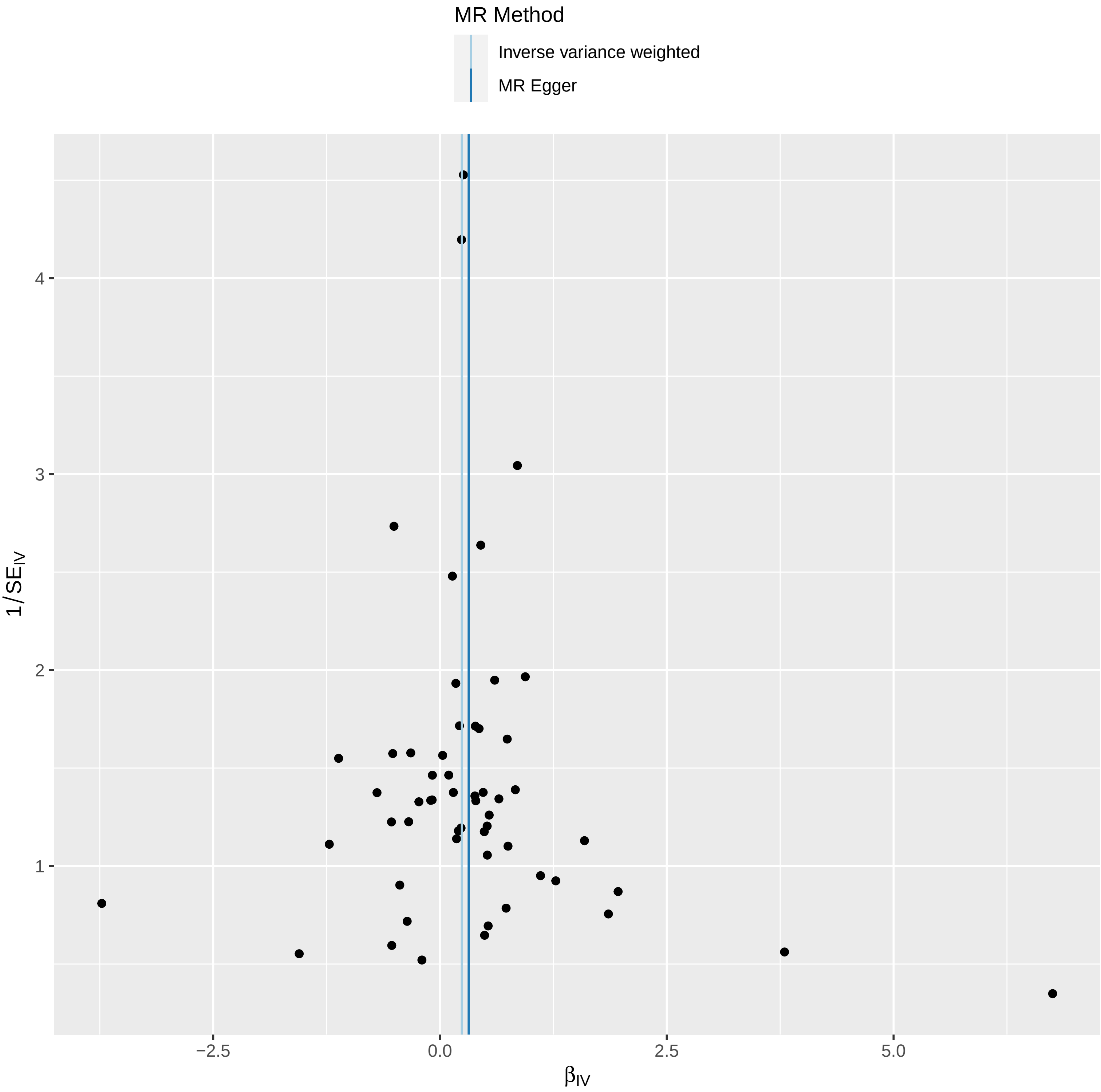
MR Method
Inverse variance weighted
MR Egger
1/SE_IV
β_IV
4
3
2
1
−2.5
0.0
2.5
5.0

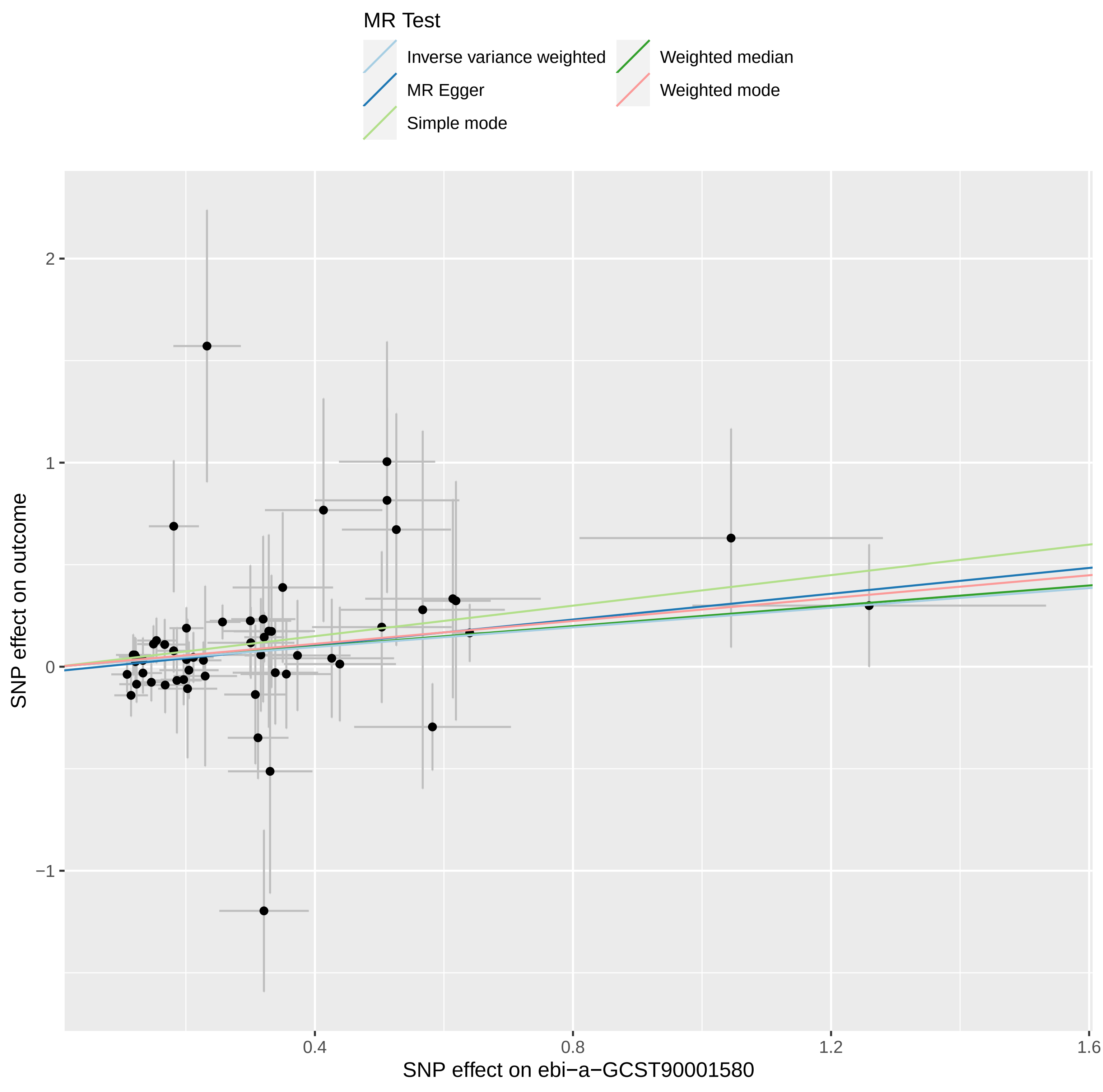

MR Test
Inverse variance weighted
MR Egger
Simple mode
Weighted median
Weighted mode
2
1
0
−1
SNP effect on outcome
0.4
0.8
1.2
1.6
SNP effect on ebi−a−GCST90001580

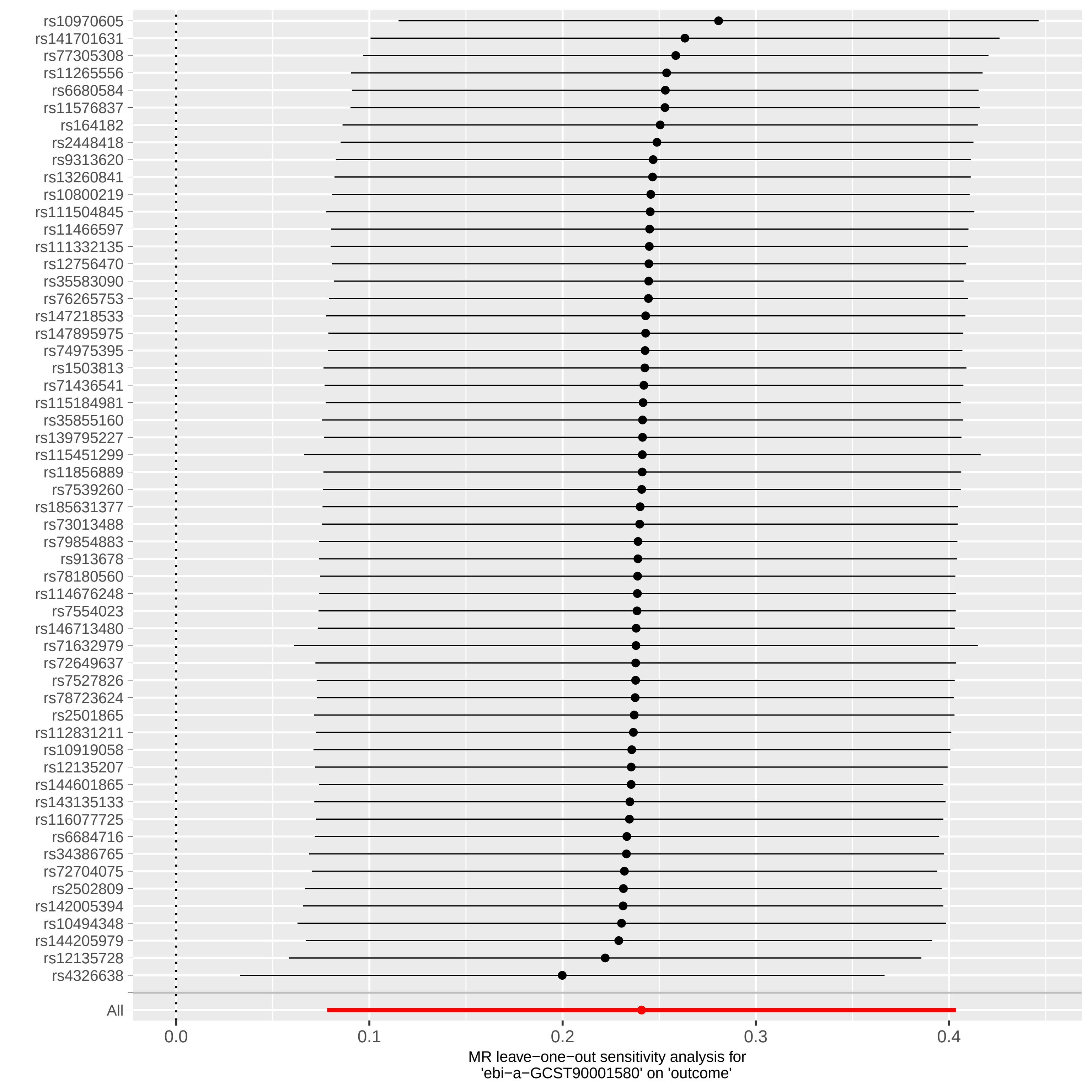

rs10970605
rs141701631
rs77305308
rs11265556
rs6680584
rs11576837
rs164182
rs2448418
rs9313620
rs13260841
rs10800219
rs111504845
rs11466597
rs111332135
rs12756470
rs35583090
rs76265753
rs147218533
rs147895975
rs74975395
rs1503813
rs71436541
rs115184981
rs35855160
rs139795227
rs115451299
rs11856889
rs7539260
rs185631377
rs73013488
rs79854883
rs913678
rs78180560
rs114676248
rs7554023
rs146713480
rs71632979
rs72649637
rs7527826
rs78723624
rs2501865
rs112831211
rs10919058
rs12135207
rs144601865
rs143135133
rs116077725
rs6684716
rs34386765
rs72704075
rs2502809
rs142005394
rs10494348
rs144205979
rs12135728
rs4326638
All
0.0
0.1
0.2
0.3
0.4
MR leave−one−out sensitivity analysis for
'ebi−a−GCST90001580' on 'outcome'

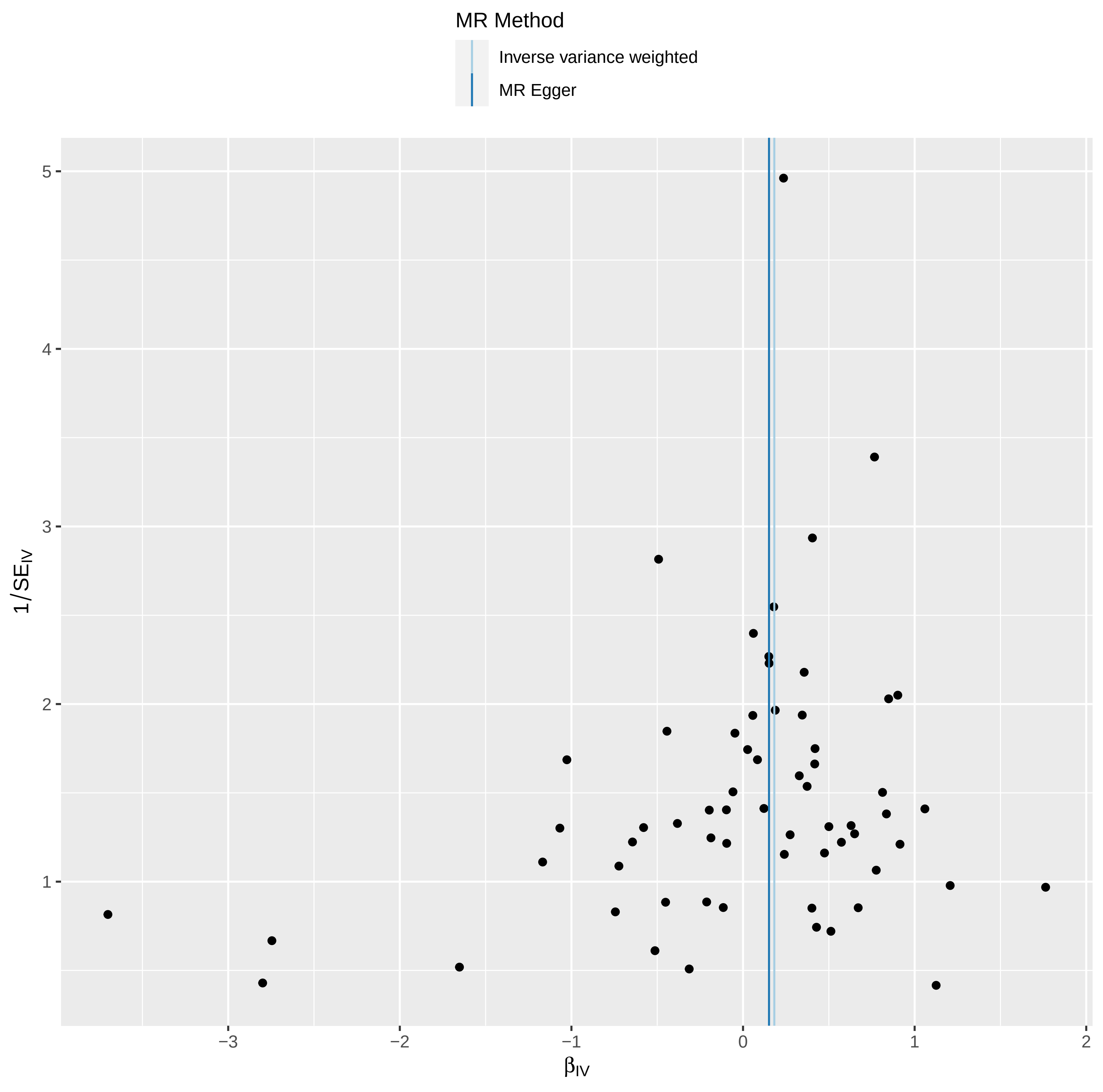

MR Method
Inverse variance weighted
MR Egger
5
4
3
2
1
1/SE_{IV}
−3
−2
−1
0
1
2
β_{IV}

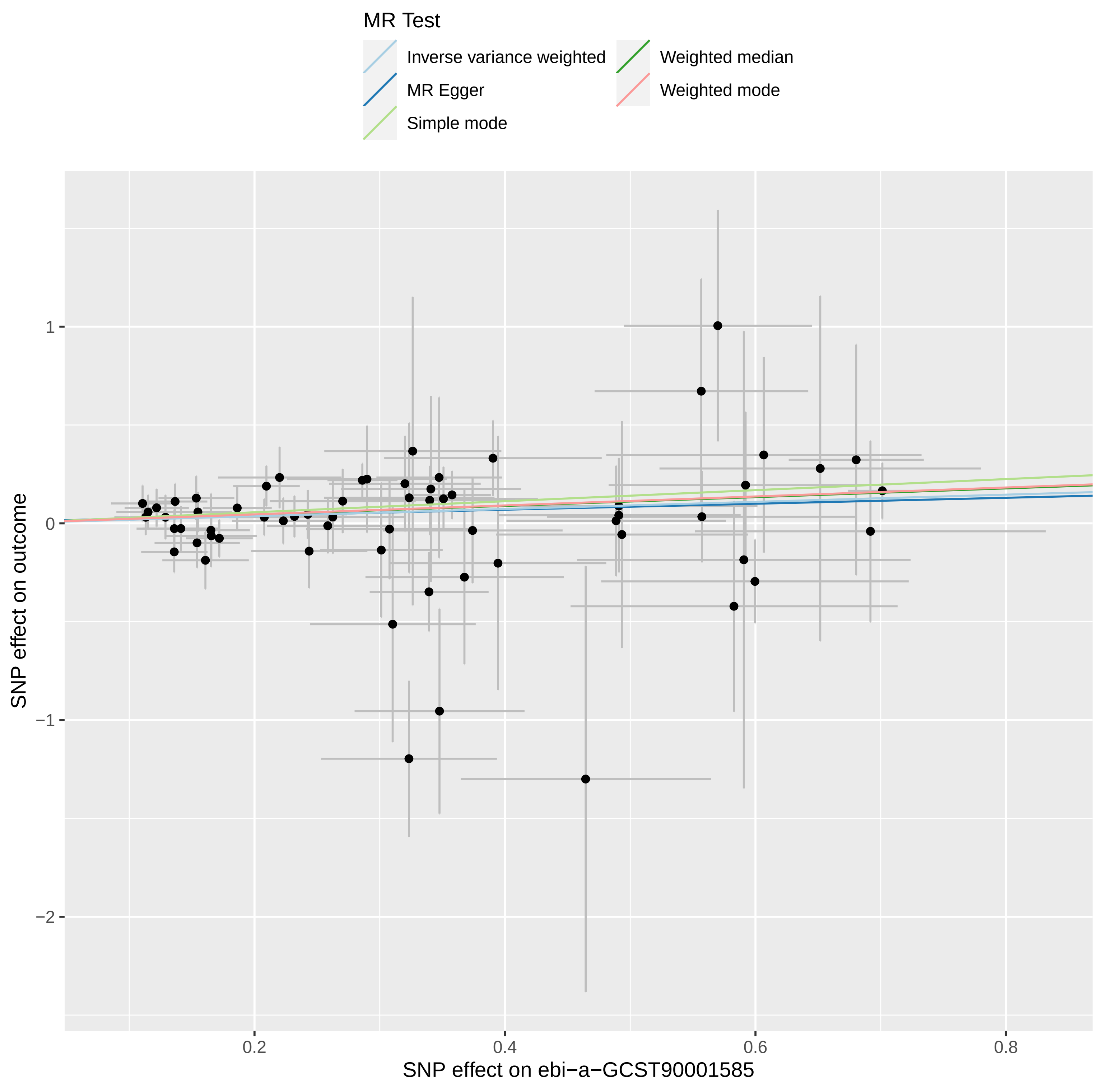
MR Test
Inverse variance weighted
MR Egger
Simple mode
Weighted median
Weighted mode
SNP effect on outcome
SNP effect on ebi−a−GCST90001585
1
0
−1
−2
0.2
0.4
0.6
0.8

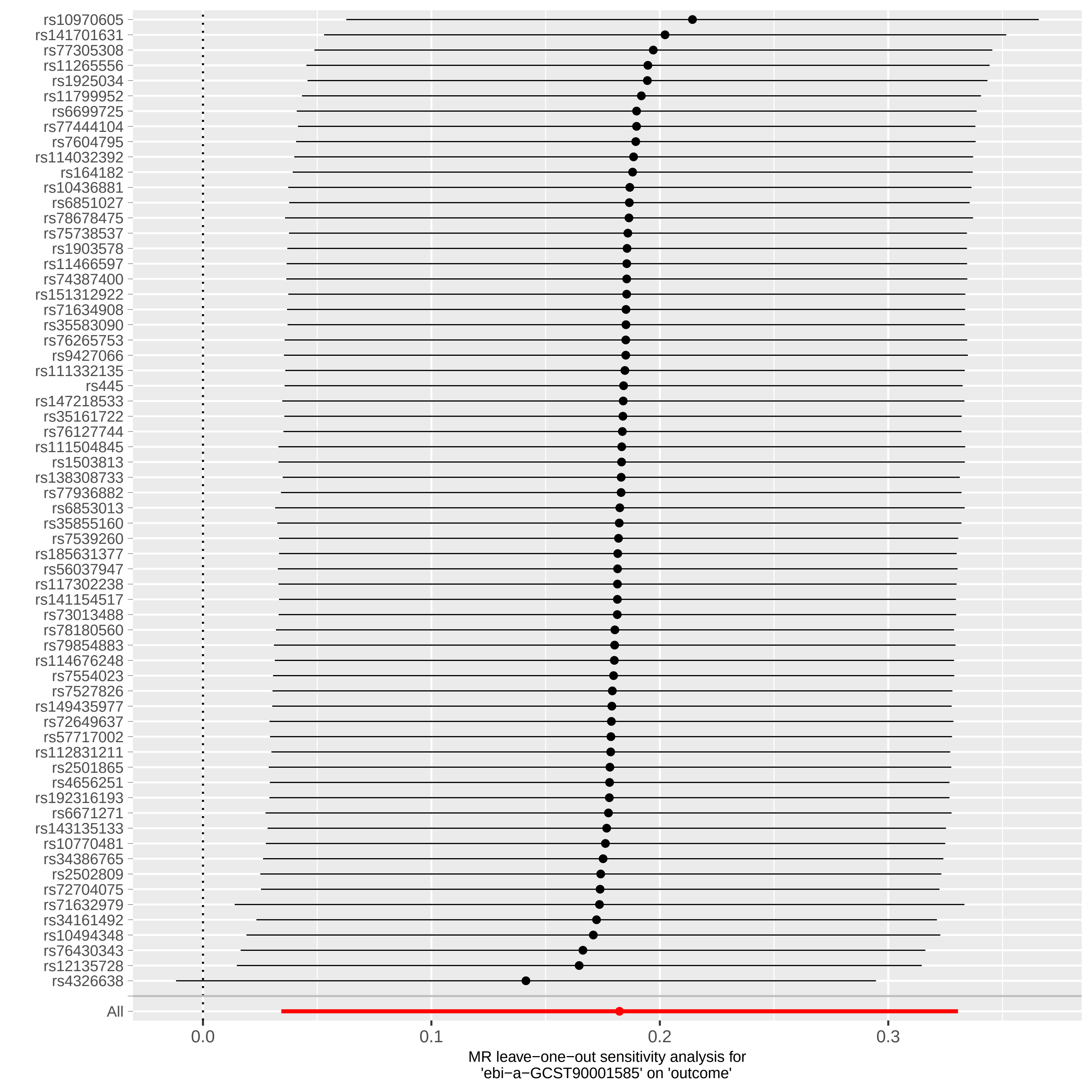

rs10970605
rs141701631
rs77305308
rs11265556
rs1925034
rs11799952
rs6699725
rs77444104
rs7604795
rs114032392
rs164182
rs10436881
rs6851027
rs78678475
rs75738537
rs1903578
rs11466597
rs74387400
rs151312922
rs71634908
rs35583090
rs76265753
rs9427066
rs111332135
rs445
rs147218533
rs35161722
rs76127744
rs111504845
rs1503813
rs138308733
rs77936882
rs6853013
rs35855160
rs7539260
rs185631377
rs56037947
rs117302238
rs141154517
rs73013488
rs78180560
rs79854883
rs114676248
rs7554023
rs7527826
rs149435977
rs72649637
rs57717002
rs112831211
rs2501865
rs4656251
rs192316193
rs6671271
rs143135133
rs10770481
rs34386765
rs2502809
rs72704075
rs71632979
rs34161492
rs10494348
rs76430343
rs12135728
rs4326638
All
0.0
0.1
0.2
0.3
MR leave−one−out sensitivity analysis for
'ebi−a−GCST90001585' on 'outcome'

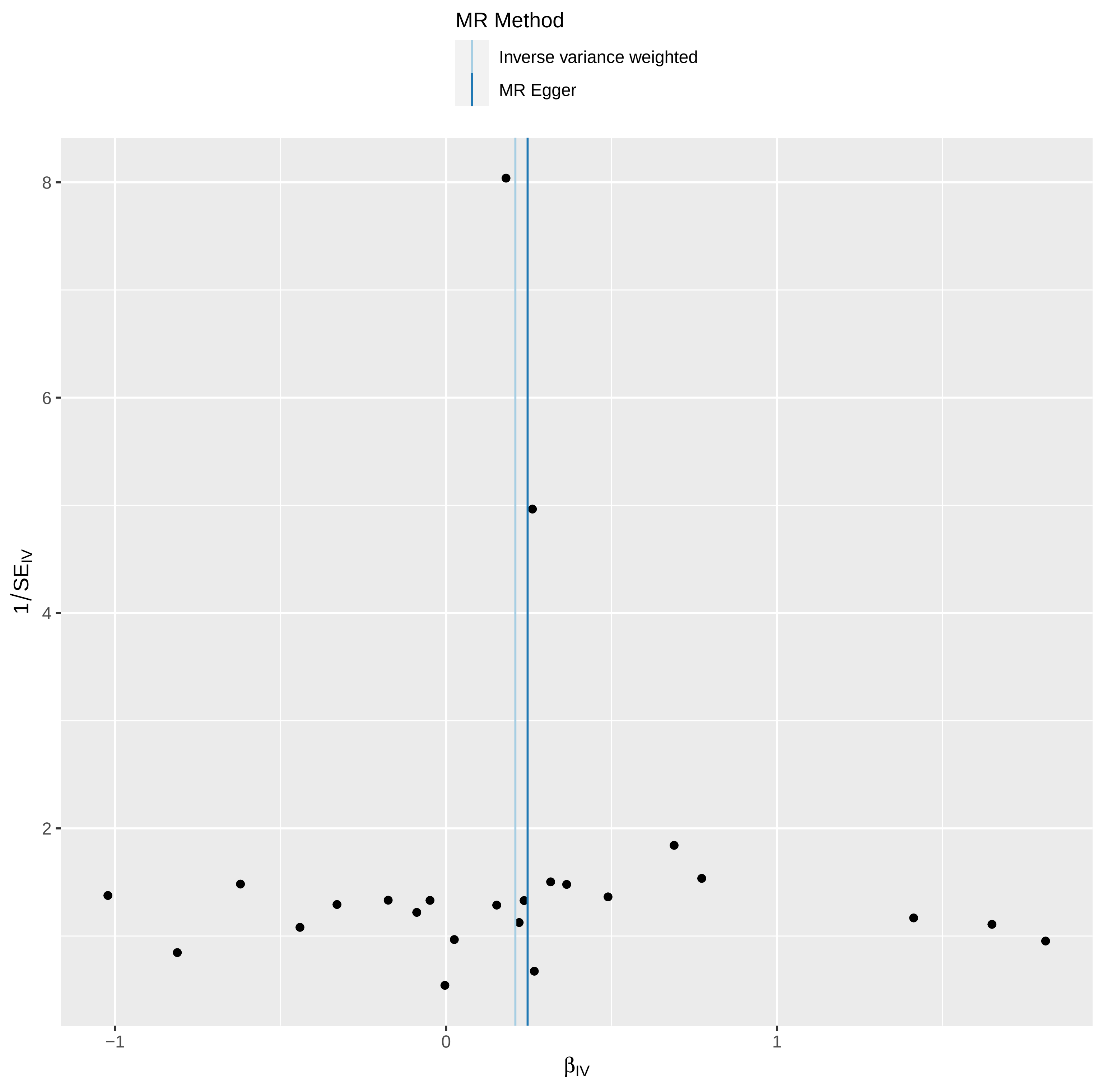

MR Method
Inverse variance weighted
MR Egger
8
6
4
2
1/SE$_{IV}$
−1
0
1
β$_{IV}$

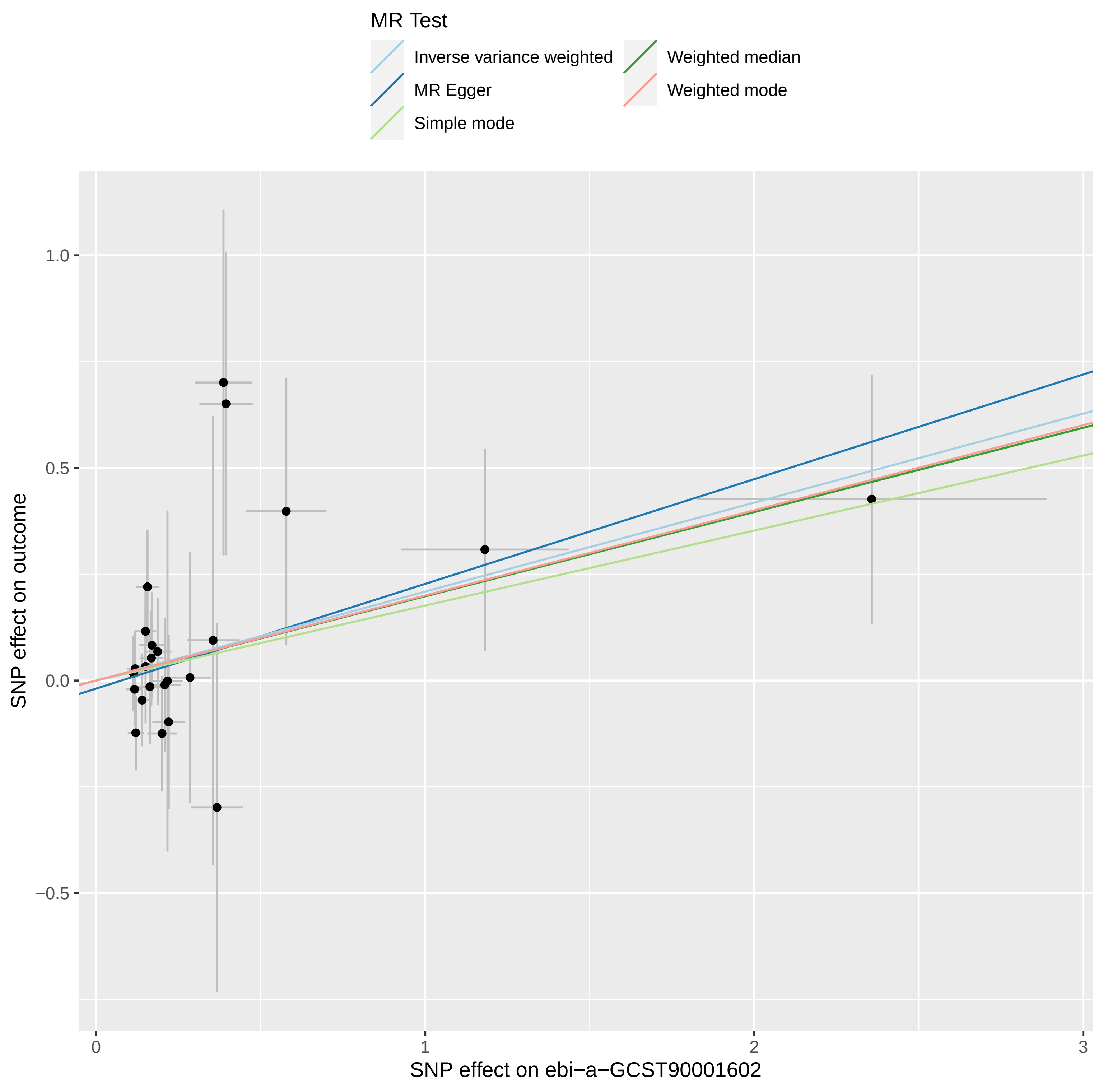
MR Test
Inverse variance weighted
MR Egger
Simple mode
Weighted median
Weighted mode
SNP effect on outcome
1.0
0.5
0.0
−0.5
0
1
2
3
SNP effect on ebi−a−GCST90001602

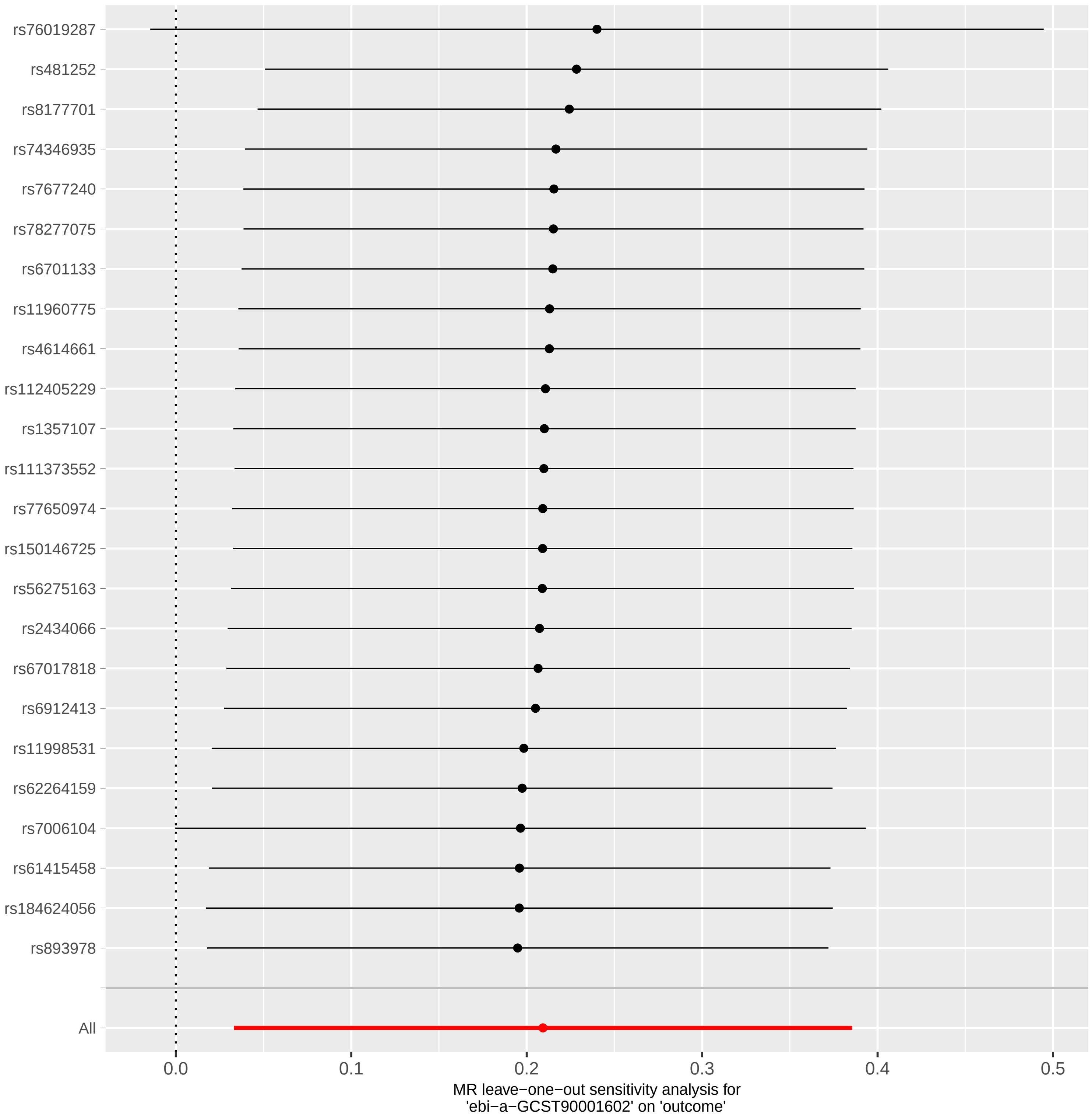
rs76019287
rs481252
rs8177701
rs74346935
rs7677240
rs78277075
rs6701133
rs11960775
rs4614661
rs112405229
rs1357107
rs111373552
rs77650974
rs150146725
rs56275163
rs2434066
rs67017818
rs6912413
rs11998531
rs62264159
rs7006104
rs61415458
rs184624056
rs893978
All
0.0
0.1
0.2
0.3
0.4
0.5
MR leave−one−out sensitivity analysis for
'ebi−a−GCST90001602' on 'outcome'

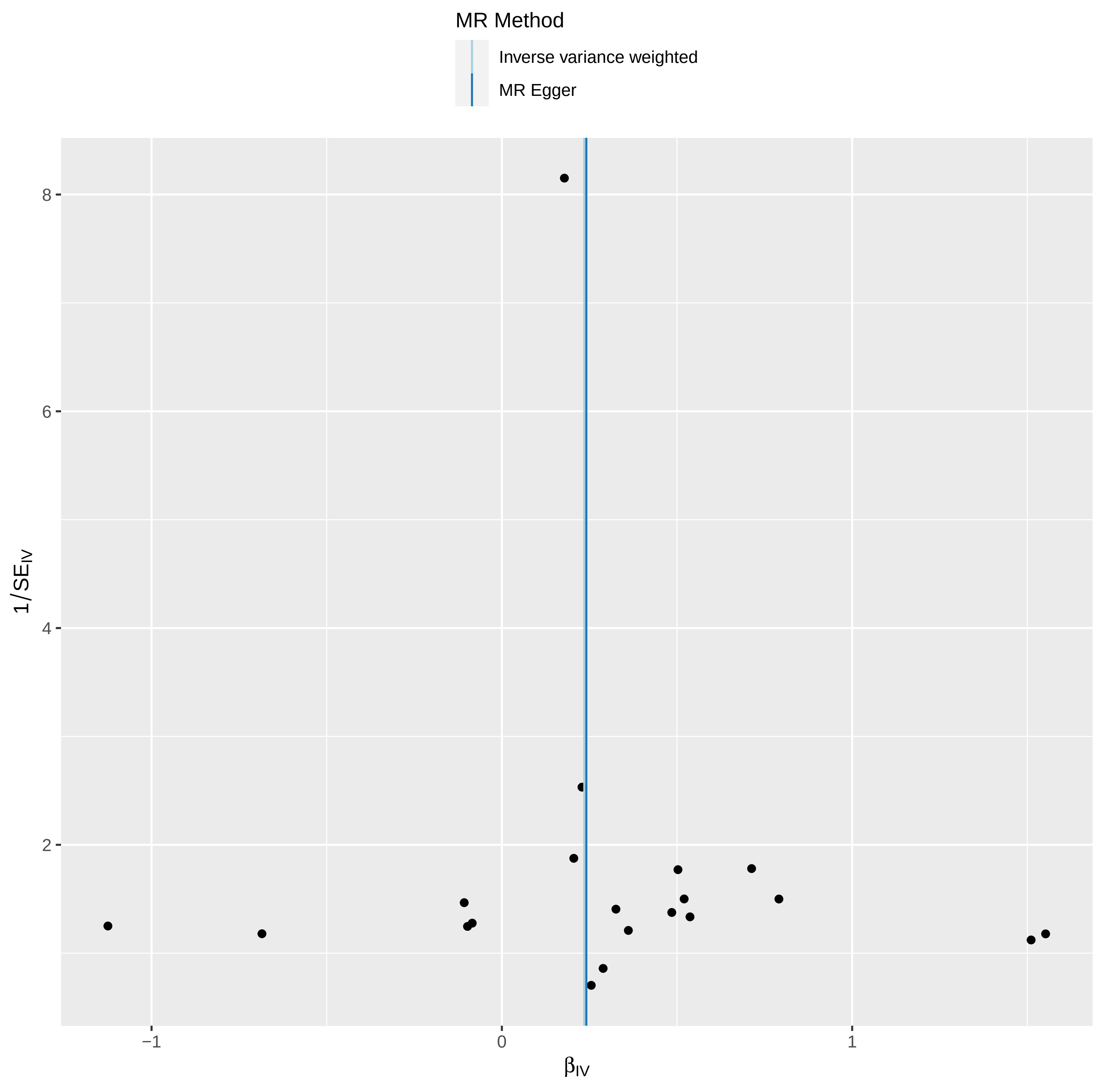

MR Method
Inverse variance weighted
MR Egger
8
6
4
2
1/SE_IV
−1
0
1
β_IV

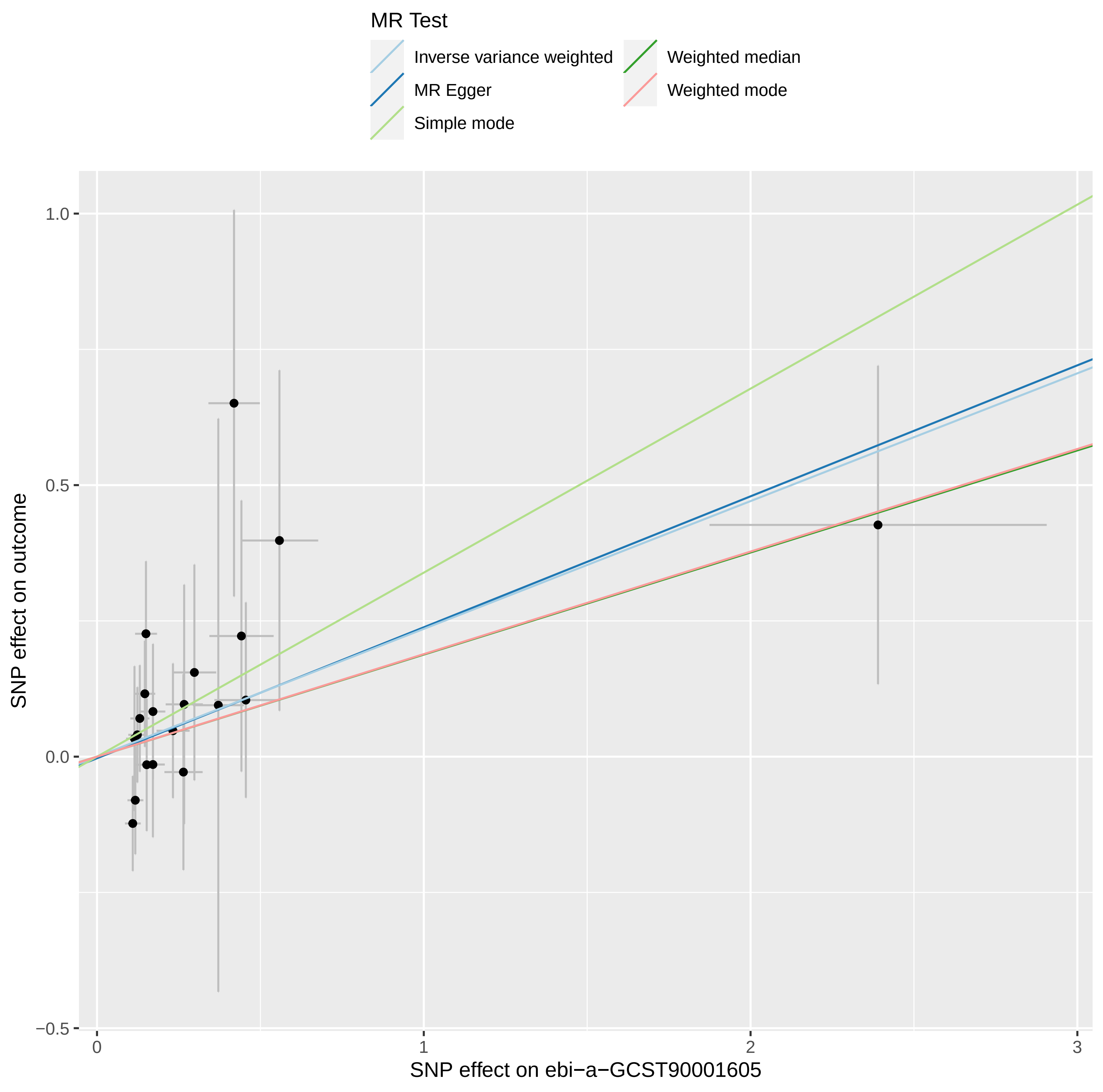
MR Test
Inverse variance weighted
MR Egger
Simple mode
Weighted median
Weighted mode
1.0
0.5
0.0
−0.5
SNP effect on outcome
0
1
2
3
SNP effect on ebi−a−GCST90001605

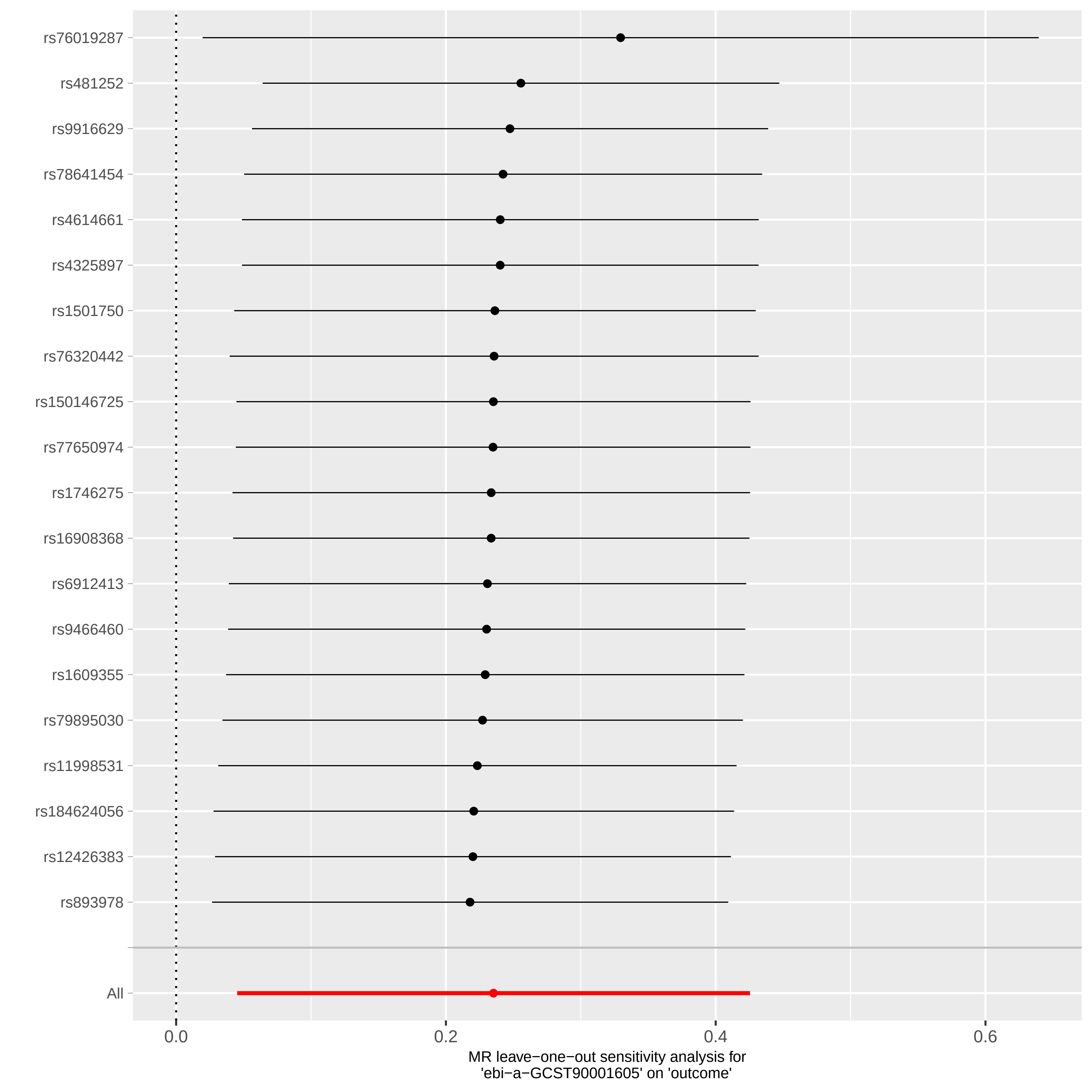

rs76019287
rs481252
rs9916629
rs78641454
rs4614661
rs4325897
rs1501750
rs76320442
rs150146725
rs77650974
rs1746275
rs16908368
rs6912413
rs9466460
rs1609355
rs79895030
rs11998531
rs184624056
rs12426383
rs893978
All
0.0
0.2
0.4
0.6
MR leave−one−out sensitivity analysis for
'ebi−a−GCST90001605' on 'outcome'

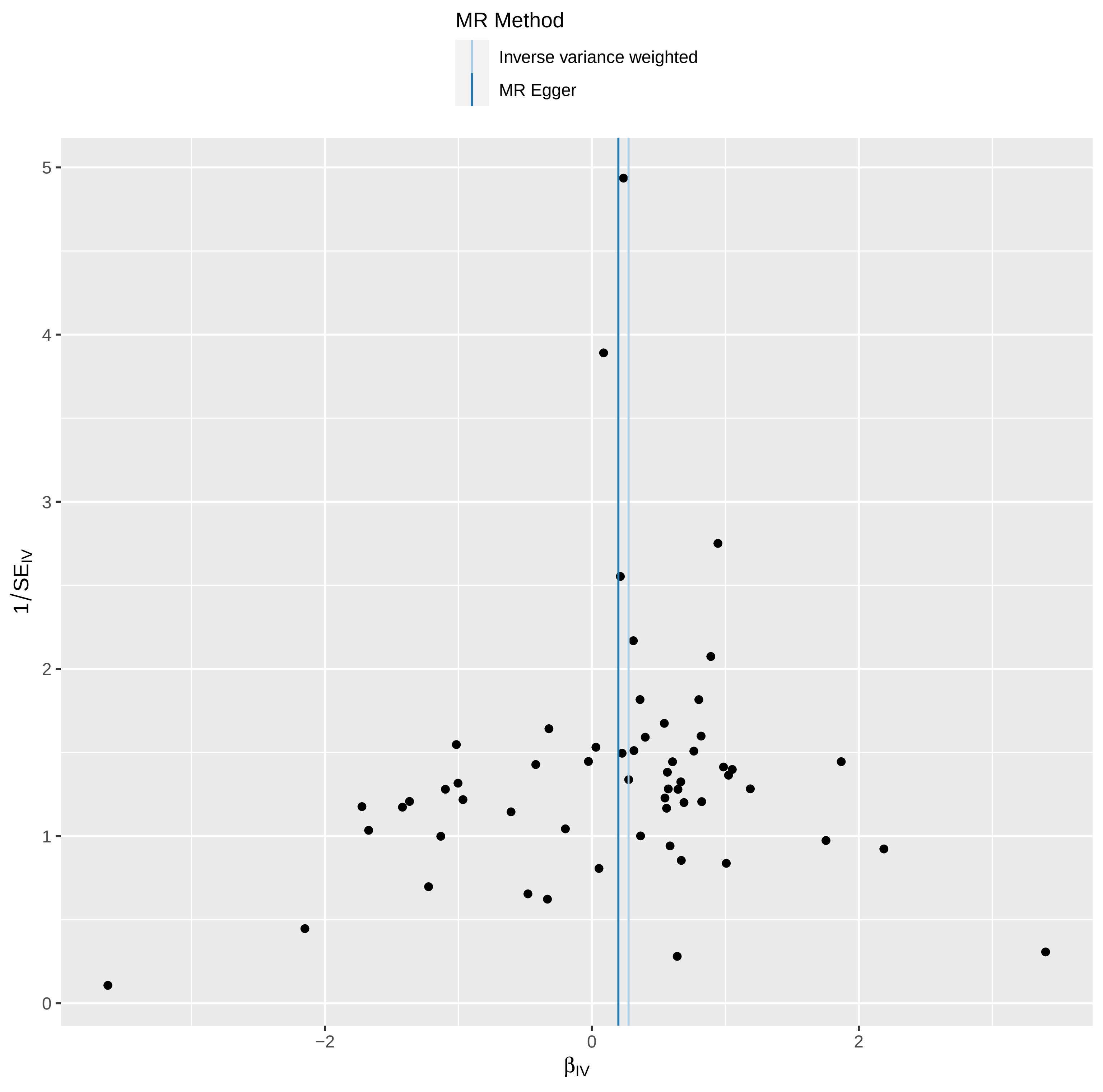

MR Method
Inverse variance weighted
MR Egger
5
4
3
2
1
0
−2
0
2
1/SE_IV
β_IV

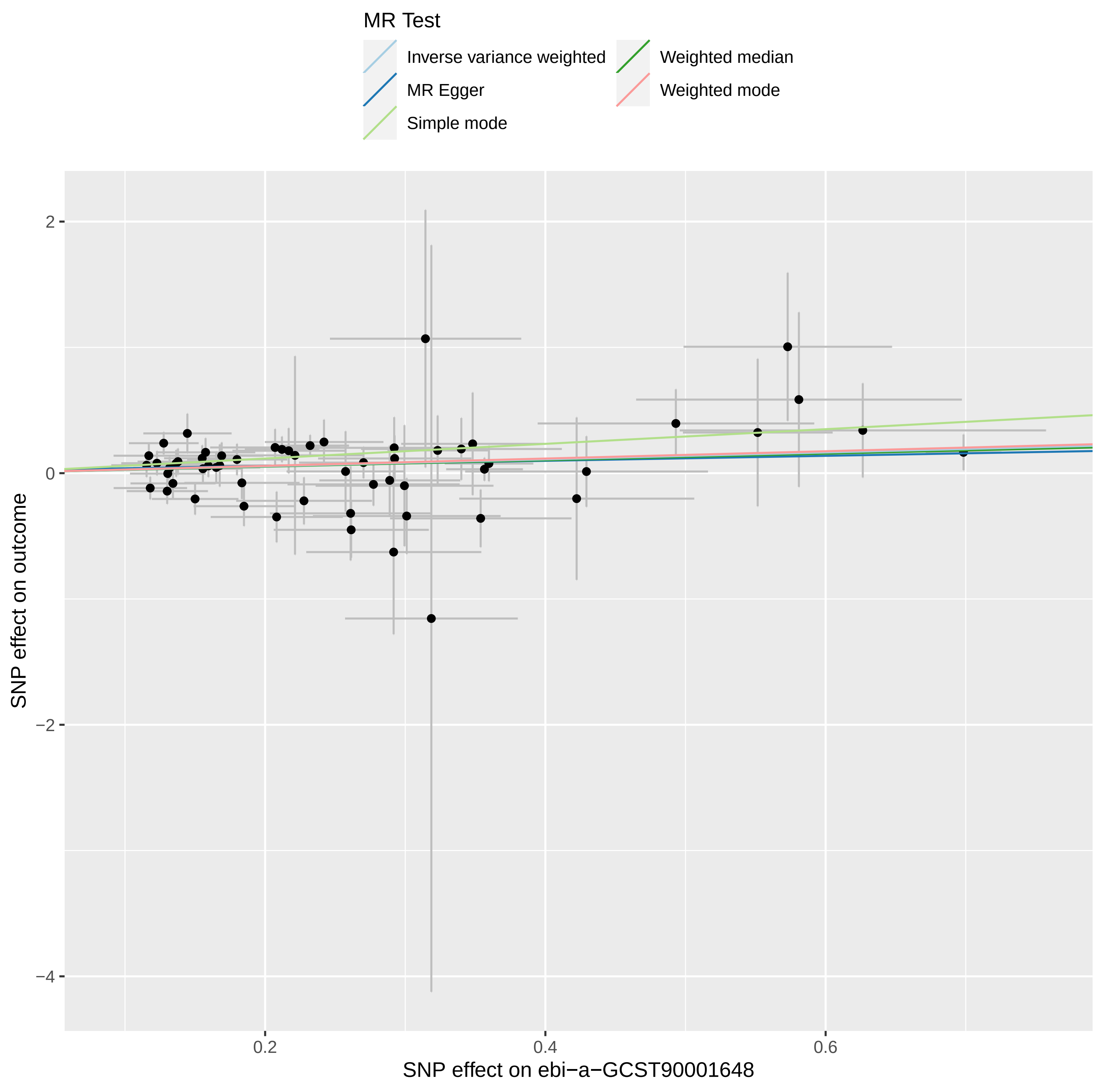

MR Test
Inverse variance weighted
MR Egger
Simple mode
Weighted median
Weighted mode
2
0
−2
−4
0.2
0.4
0.6
SNP effect on outcome
SNP effect on ebi−a−GCST90001648

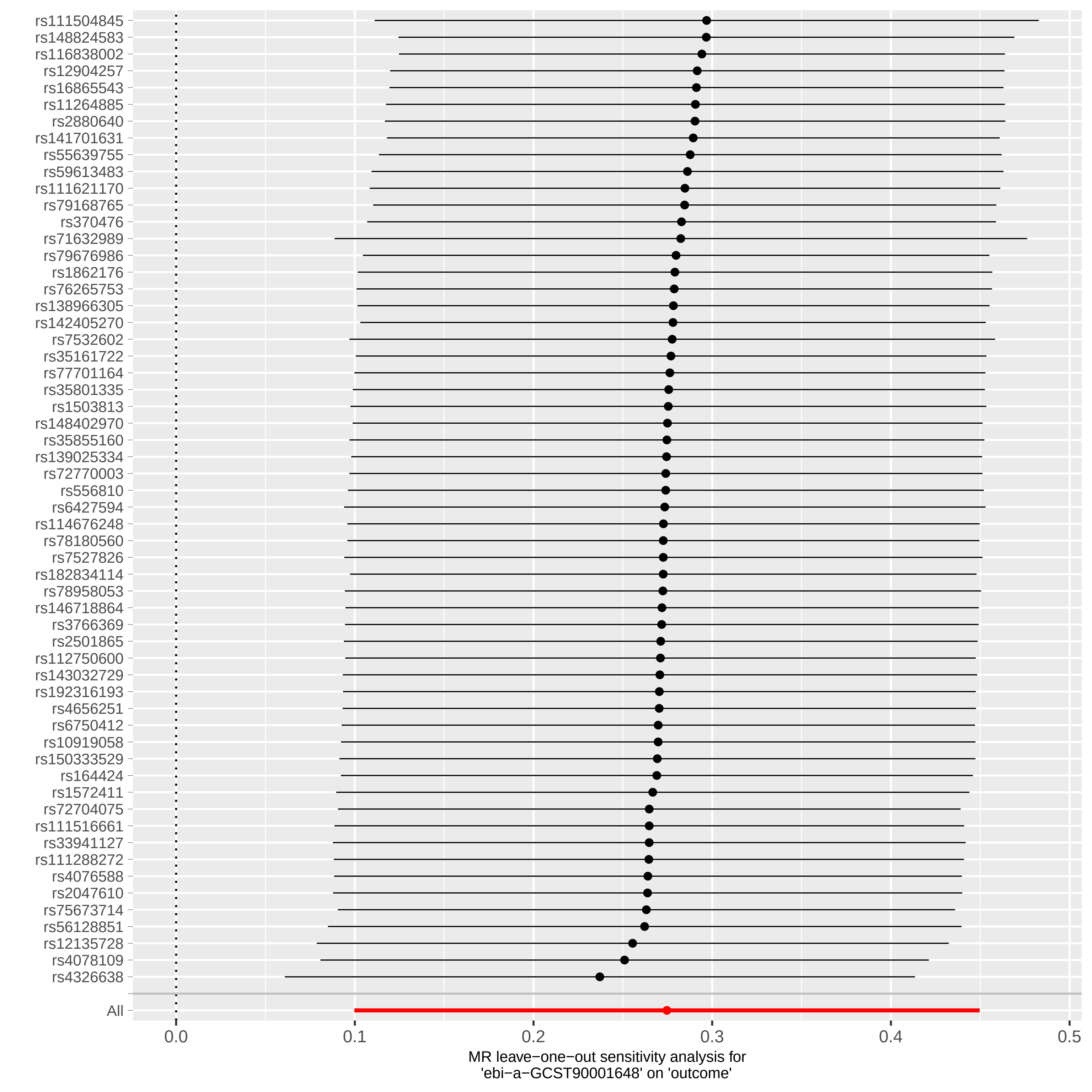

rs111504845
rs148824583
rs116838002
rs12904257
rs16865543
rs11264885
rs2880640
rs141701631
rs55639755
rs59613483
rs111621170
rs79168765
rs370476
rs71632989
rs79676986
rs1862176
rs76265753
rs138966305
rs142405270
rs7532602
rs35161722
rs77701164
rs35801335
rs1503813
rs148402970
rs35855160
rs139025334
rs72770003
rs556810
rs6427594
rs114676248
rs78180560
rs7527826
rs182834114
rs78958053
rs146718864
rs3766369
rs2501865
rs112750600
rs143032729
rs192316193
rs4656251
rs6750412
rs10919058
rs150333529
rs164424
rs1572411
rs72704075
rs111516661
rs33941127
rs111288272
rs4076588
rs2047610
rs75673714
rs56128851
rs12135728
rs4078109
rs4326638
All
0.0
0.1
0.2
0.3
0.4
0.5
MR leave−one−out sensitivity analysis for
'ebi−a−GCST90001648' on 'outcome'

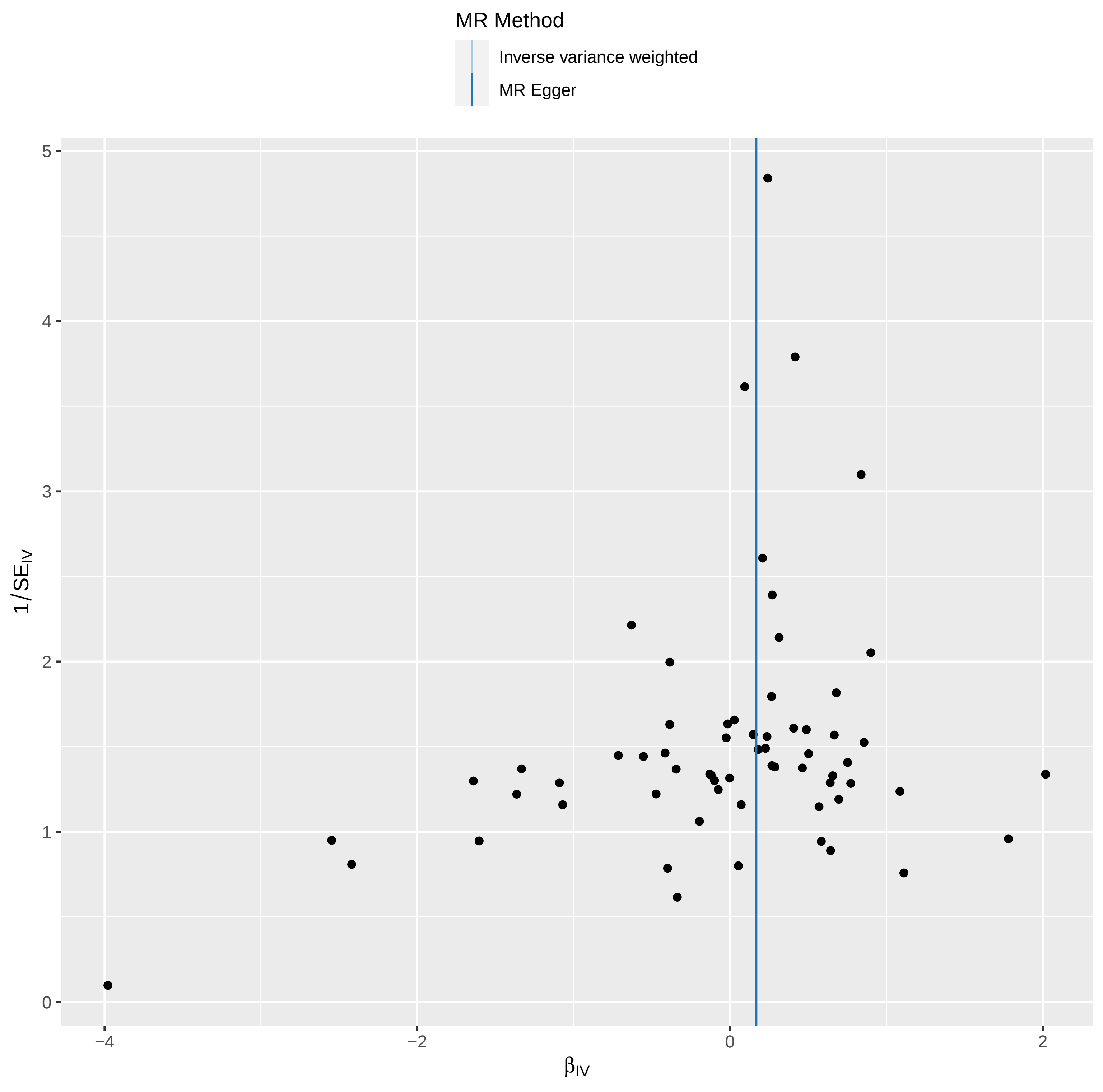

MR Method
Inverse variance weighted
MR Egger
5
4
3
2
1
0
1/SE_IV
−4
−2
0
2
β_IV

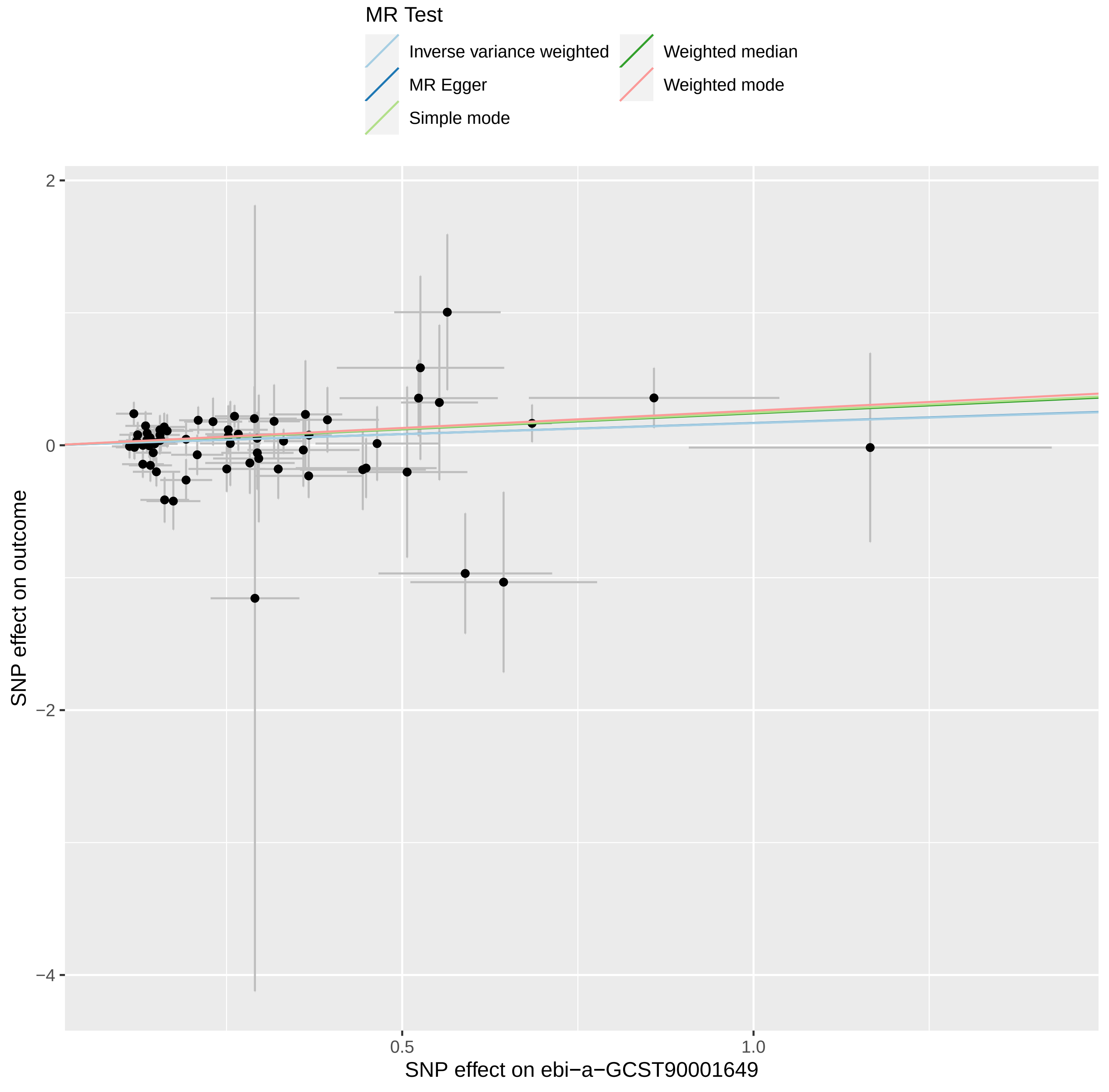

MR Test
Inverse variance weighted
MR Egger
Simple mode
Weighted median
Weighted mode
2
0
−2
−4
0.5
1.0
SNP effect on outcome
SNP effect on ebi−a−GCST90001649

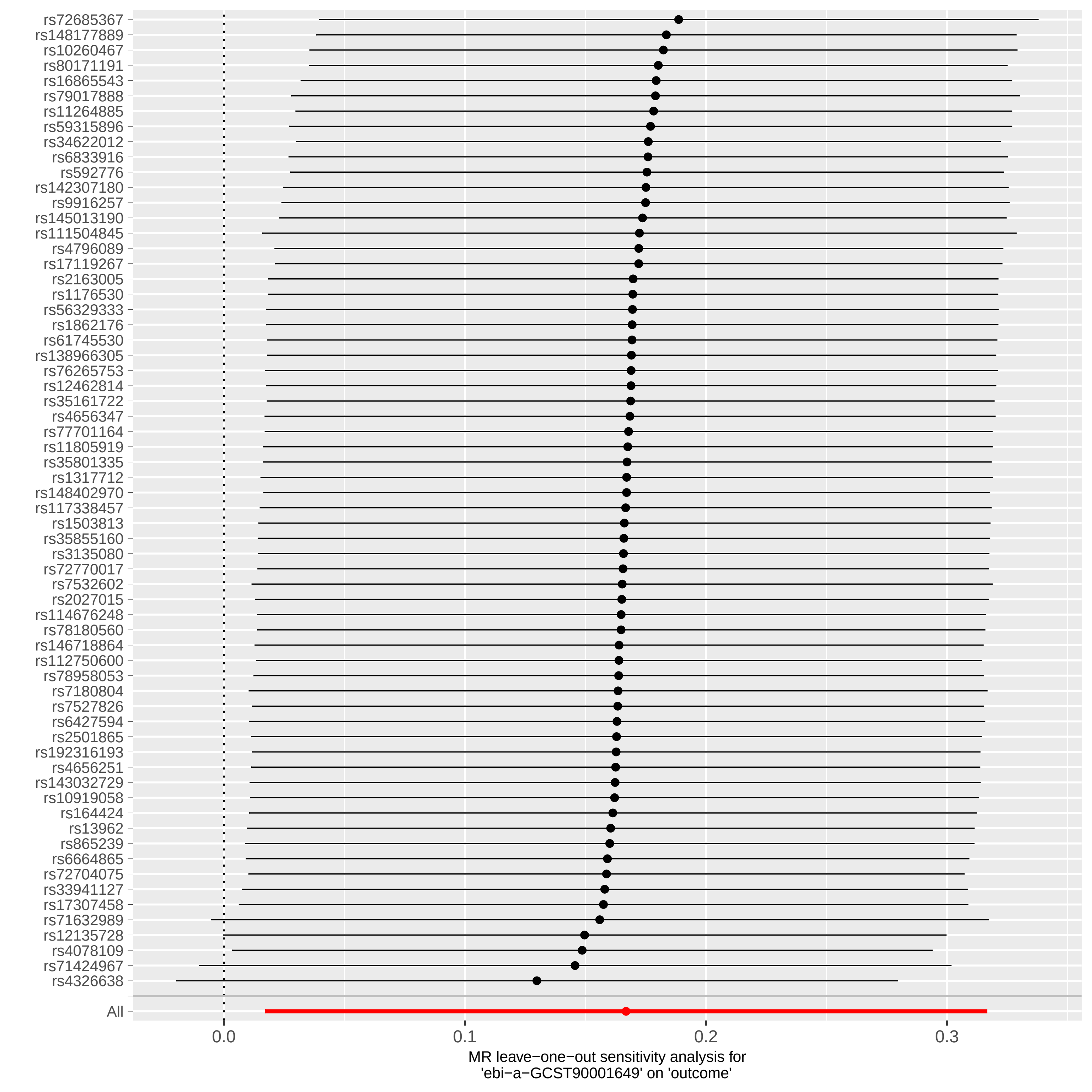
rs72685367
rs148177889
rs10260467
rs80171191
rs16865543
rs79017888
rs11264885
rs59315896
rs34622012
rs6833916
rs592776
rs142307180
rs9916257
rs145013190
rs111504845
rs4796089
rs17119267
rs2163005
rs1176530
rs56329333
rs1862176
rs61745530
rs138966305
rs76265753
rs12462814
rs35161722
rs4656347
rs77701164
rs11805919
rs35801335
rs1317712
rs148402970
rs117338457
rs1503813
rs35855160
rs3135080
rs72770017
rs7532602
rs2027015
rs114676248
rs78180560
rs146718864
rs112750600
rs78958053
rs7180804
rs7527826
rs6427594
rs2501865
rs192316193
rs4656251
rs143032729
rs10919058
rs164424
rs13962
rs865239
rs6664865
rs72704075
rs33941127
rs17307458
rs71632989
rs12135728
rs4078109
rs71424967
rs4326638
All
0.0
0.1
0.2
0.3
MR leave−one−out sensitivity analysis for
'ebi−a−GCST90001649' on 'outcome'

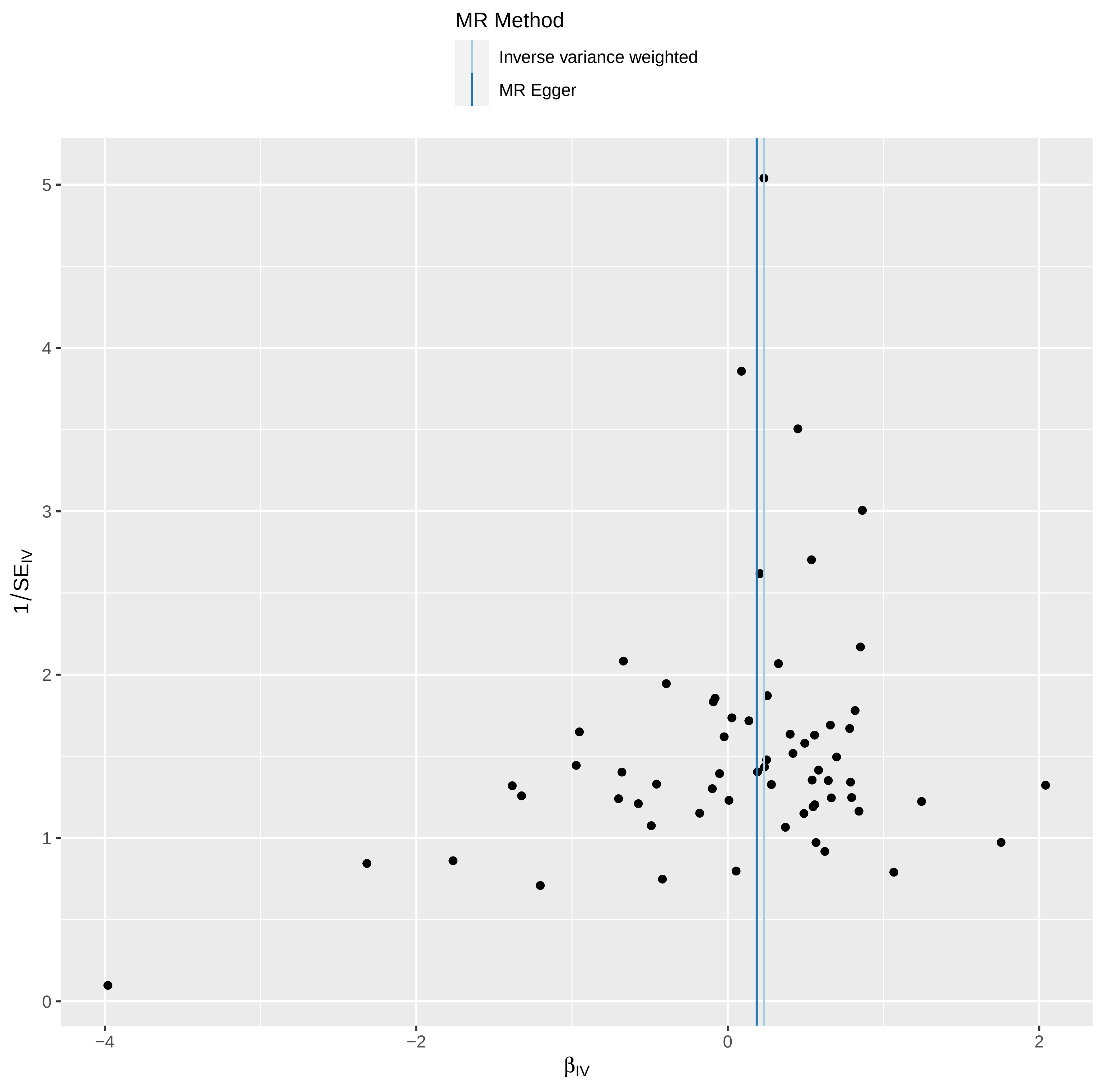

MR Method
Inverse variance weighted
MR Egger
5
4
3
2
1
0
1/SE_IV
−4
−2
0
2
β_IV

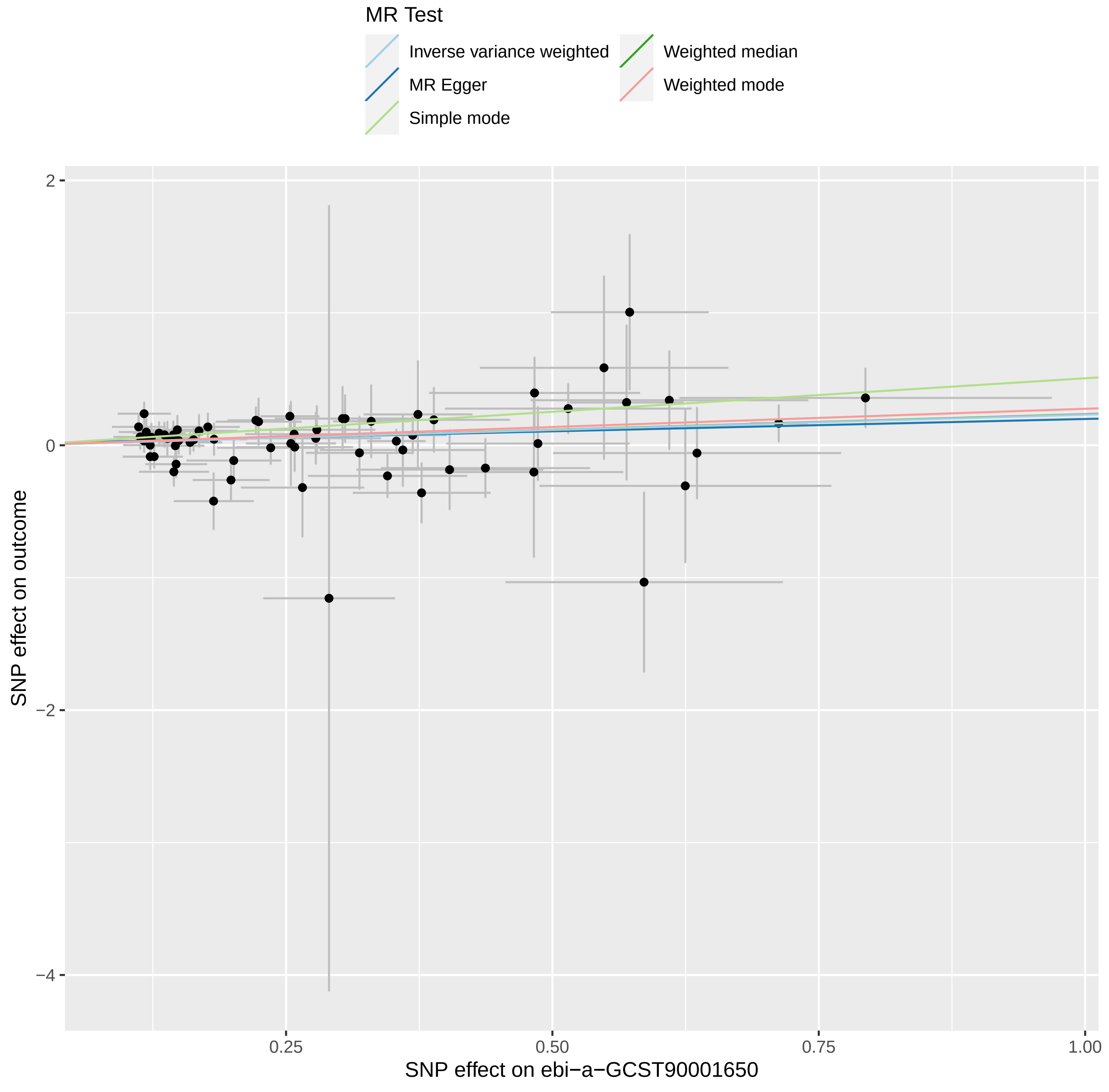

MR Test
Inverse variance weighted
MR Egger
Simple mode
Weighted median
Weighted mode
2
0
−2
−4
0.25
0.50
0.75
1.00
SNP effect on outcome
SNP effect on ebi−a−GCST90001650

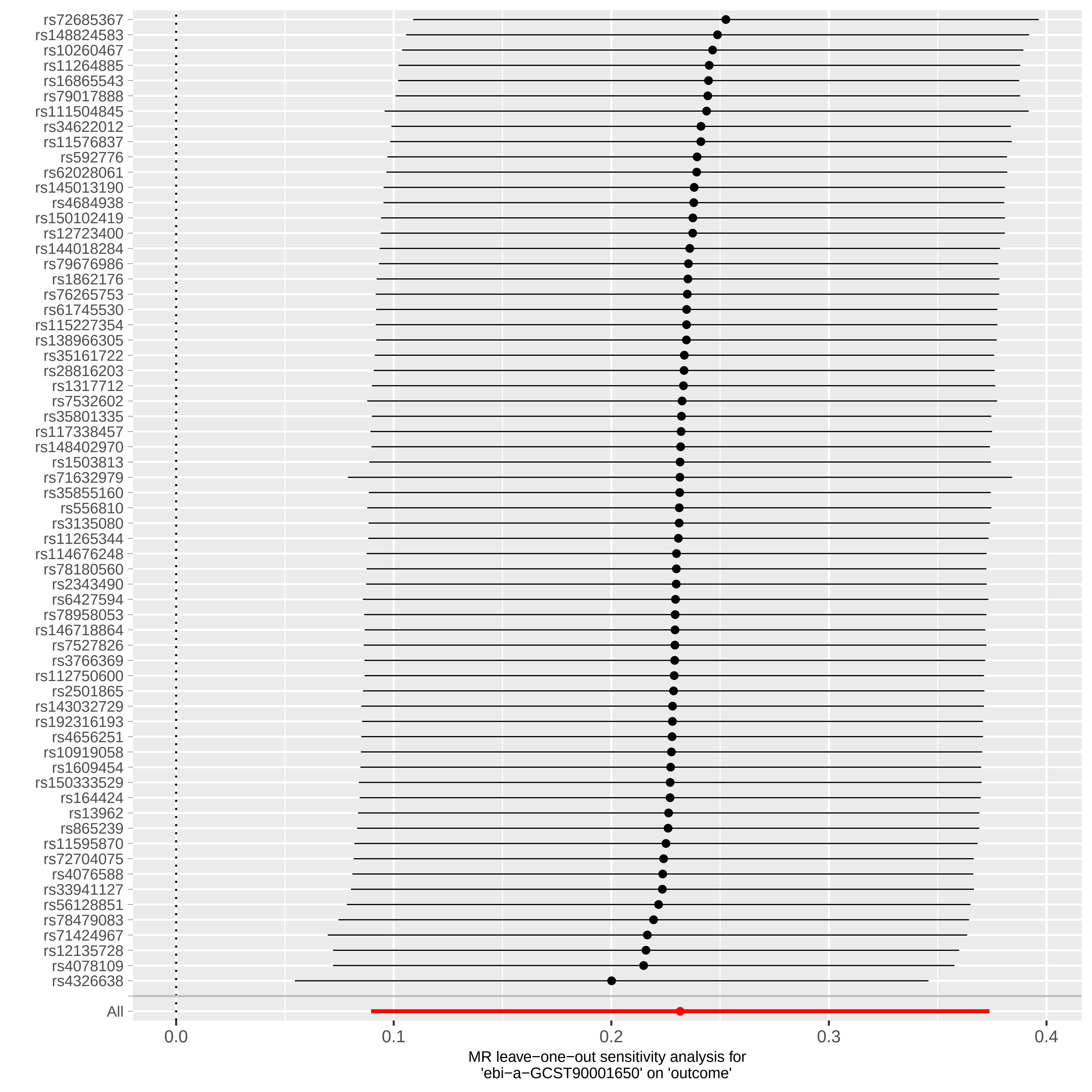

rs72685367
rs148824583
rs10260467
rs11264885
rs16865543
rs79017888
rs111504845
rs34622012
rs11576837
rs592776
rs62028061
rs145013190
rs4684938
rs150102419
rs12723400
rs144018284
rs79676986
rs1862176
rs76265753
rs61745530
rs115227354
rs138966305
rs35161722
rs28816203
rs1317712
rs7532602
rs35801335
rs117338457
rs148402970
rs1503813
rs71632979
rs35855160
rs556810
rs3135080
rs11265344
rs114676248
rs78180560
rs2343490
rs6427594
rs78958053
rs146718864
rs7527826
rs3766369
rs112750600
rs2501865
rs143032729
rs192316193
rs4656251
rs10919058
rs1609454
rs150333529
rs164424
rs13962
rs865239
rs11595870
rs72704075
rs4076588
rs33941127
rs56128851
rs78479083
rs71424967
rs12135728
rs4078109
rs4326638
All
0.0
0.1
0.2
0.3
0.4
MR leave−one−out sensitivity analysis for
'ebi−a−GCST90001650' on 'outcome'

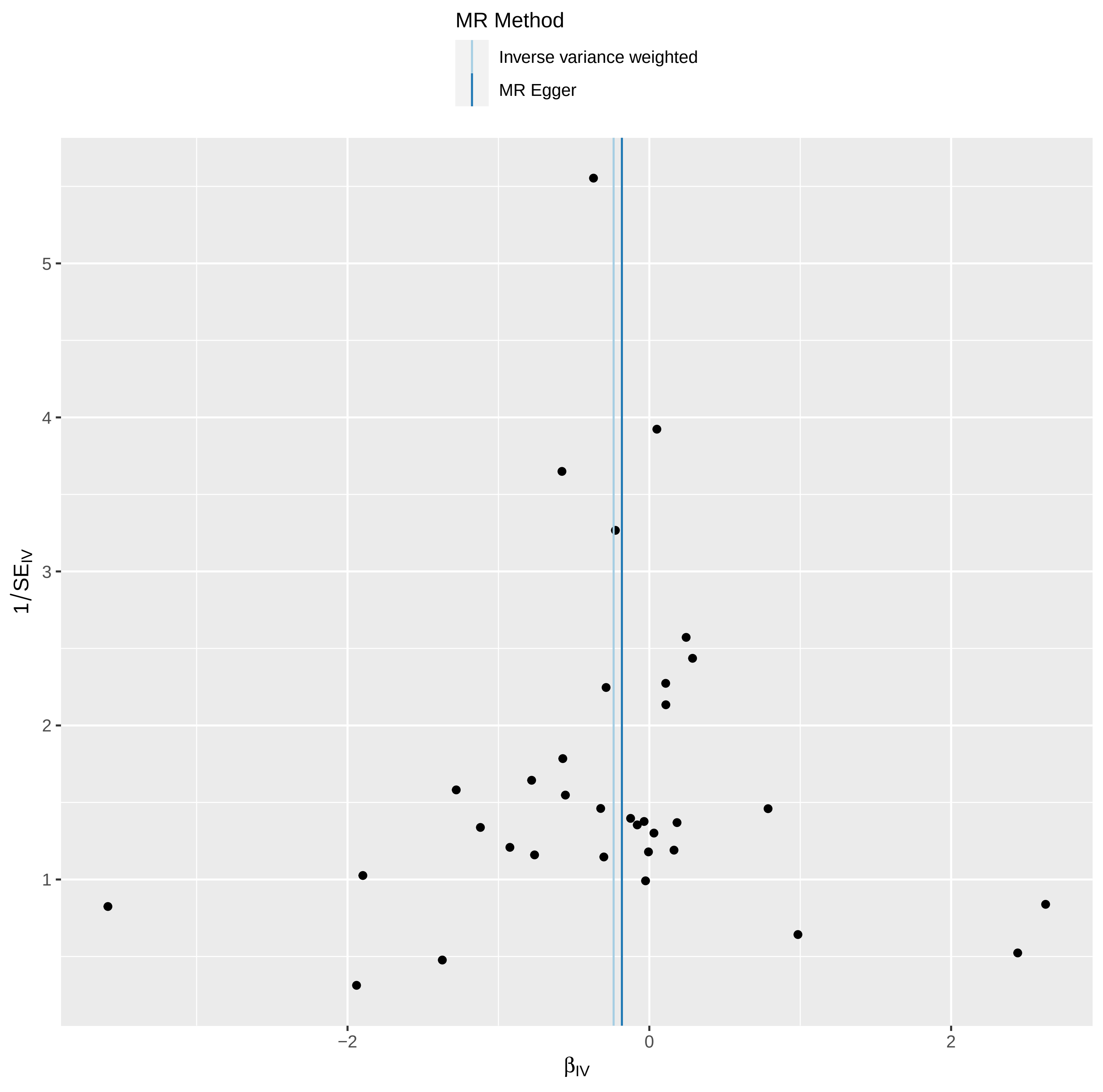

MR Method
Inverse variance weighted
MR Egger
5
4
3
2
1
1/SE_IV
−2
0
2
β_IV

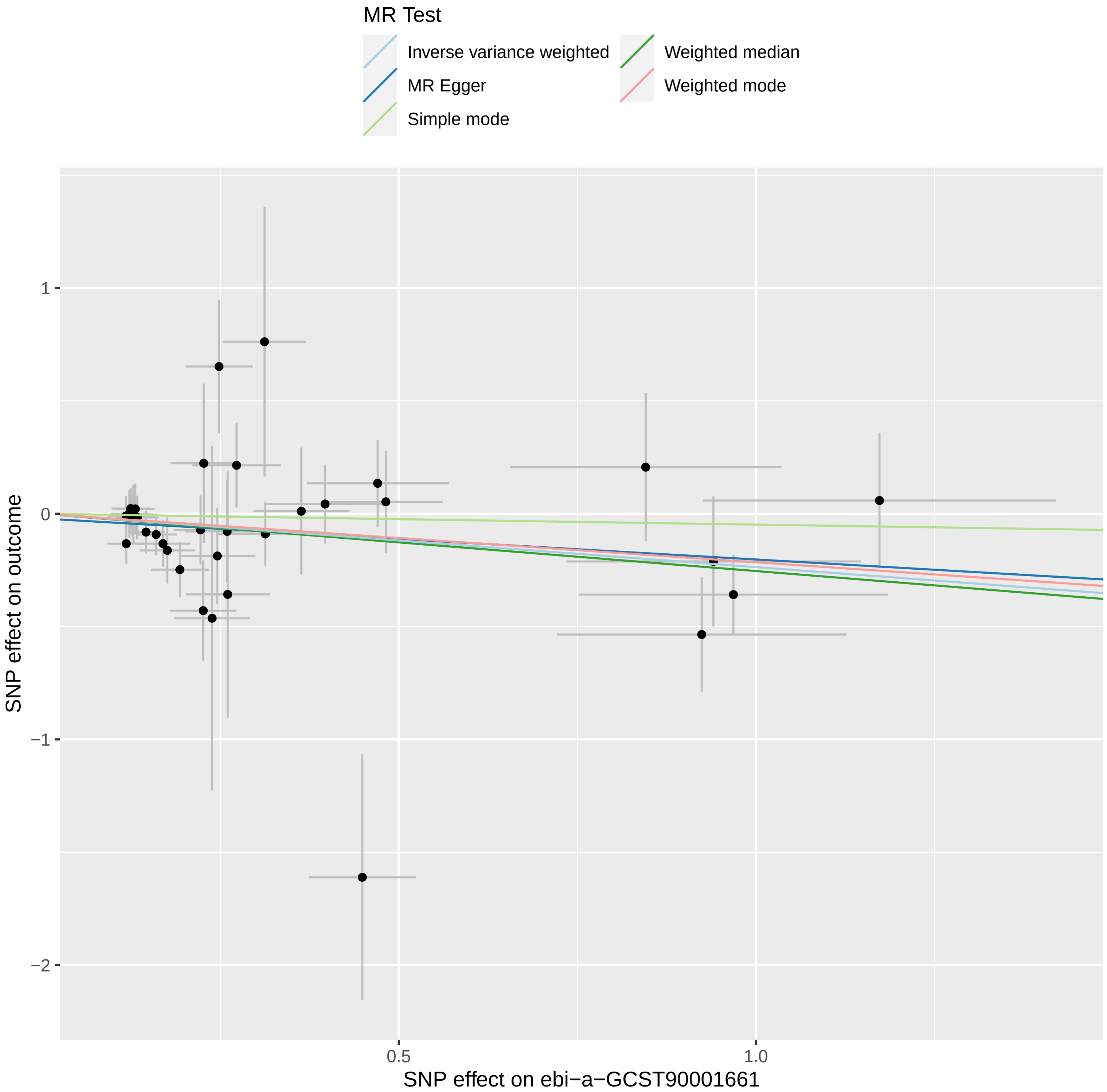
MR Test
Inverse variance weighted
MR Egger
Simple mode
Weighted median
Weighted mode
1
0
−1
−2
0.5
1.0
SNP effect on outcome
SNP effect on ebi−a−GCST90001661

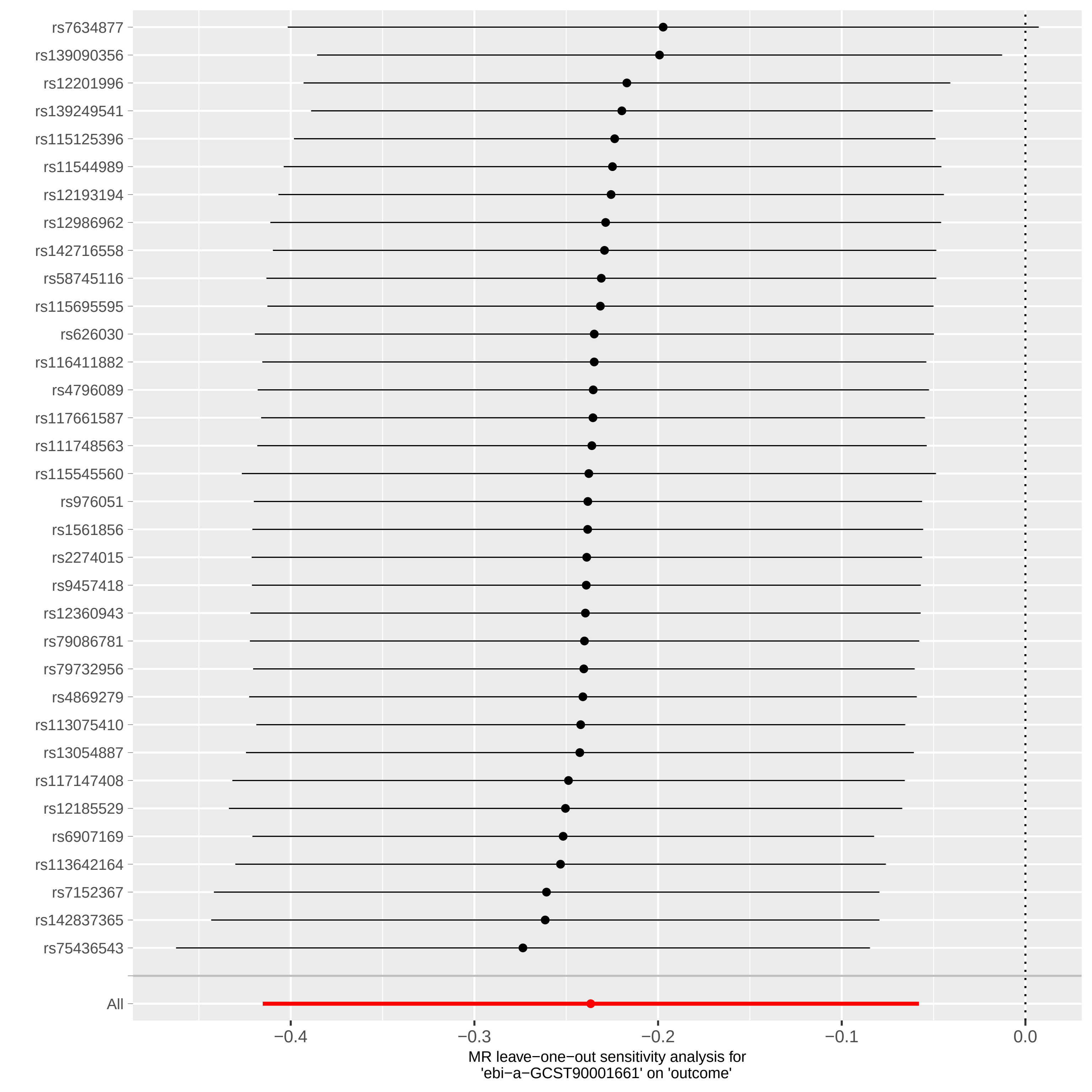
rs7634877
rs139090356
rs12201996
rs139249541
rs115125396
rs11544989
rs12193194
rs12986962
rs142716558
rs58745116
rs115695595
rs626030
rs116411882
rs4796089
rs117661587
rs111748563
rs115545560
rs976051
rs1561856
rs2274015
rs9457418
rs12360943
rs79086781
rs79732956
rs4869279
rs113075410
rs13054887
rs117147408
rs12185529
rs6907169
rs113642164
rs7152367
rs142837365
rs75436543
All
−0.4
−0.3
−0.2
−0.1
0.0
MR leave−one−out sensitivity analysis for
'ebi−a−GCST90001661' on 'outcome'

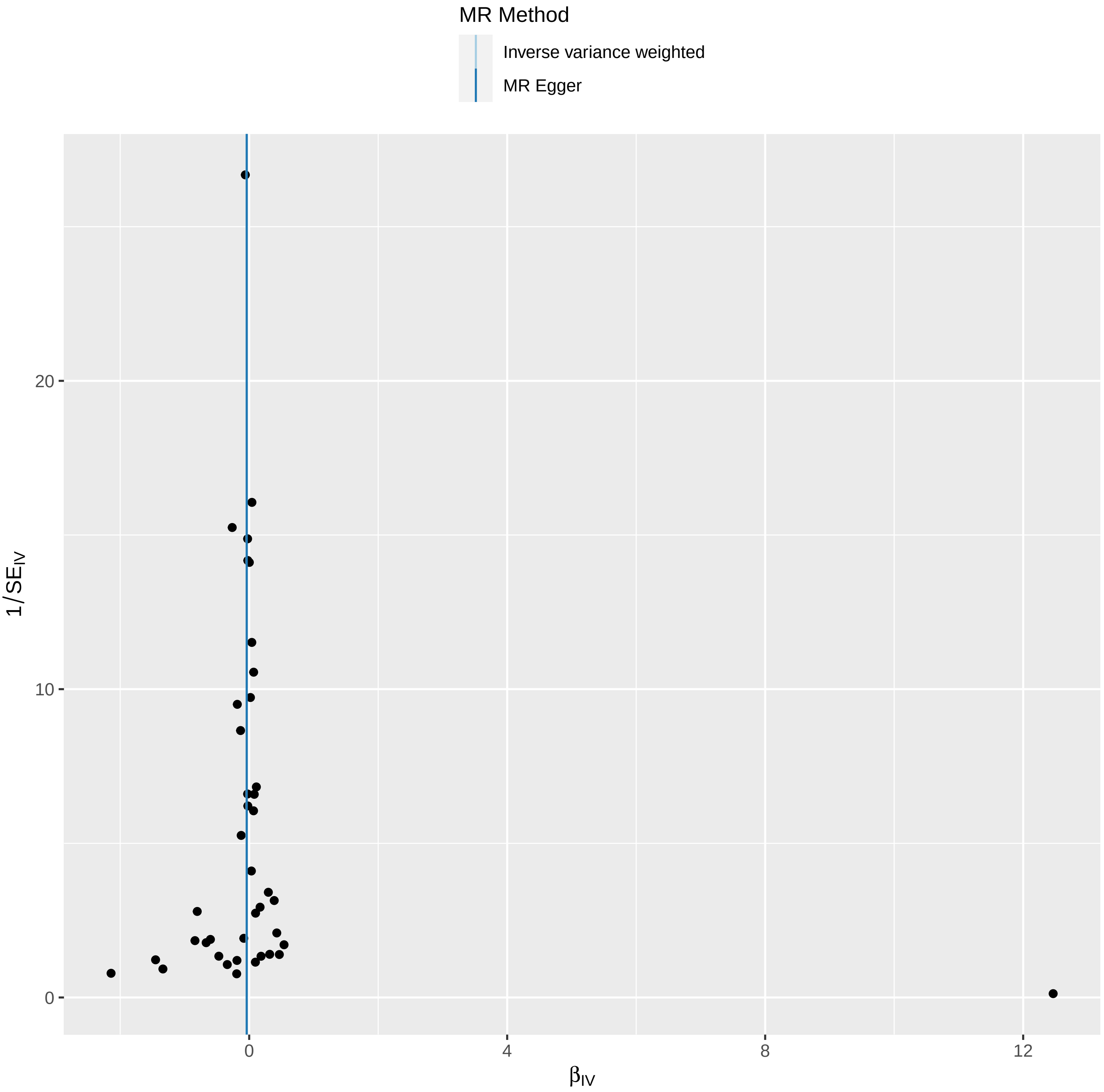

MR Method
Inverse variance weighted
MR Egger
1/SE_IV
β_IV
20
10
0
0
4
8
12

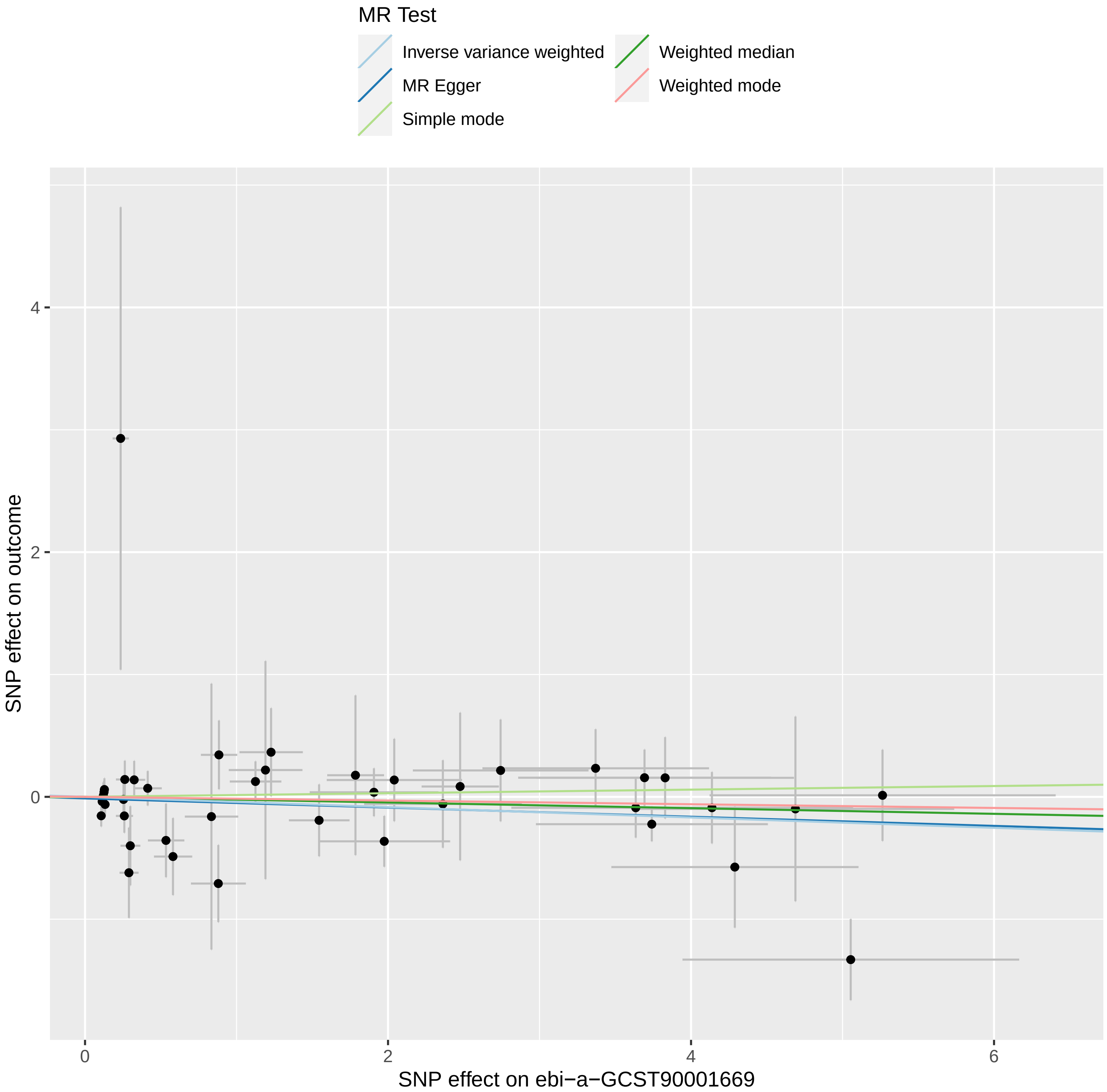

MR Test
Inverse variance weighted
MR Egger
Simple mode
Weighted median
Weighted mode
SNP effect on outcome
4
2
0
0
2
4
6
SNP effect on ebi−a−GCST90001669

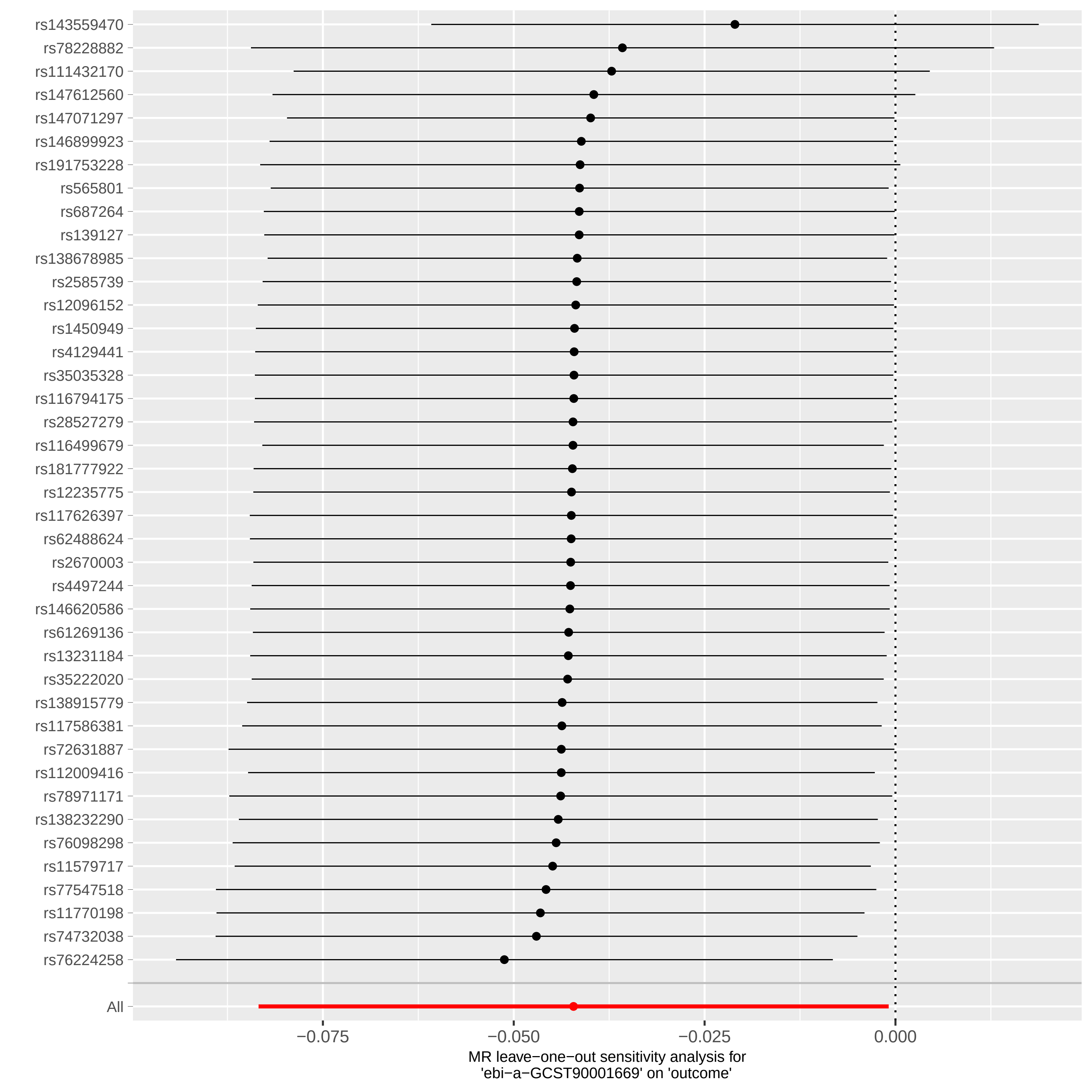
rs143559470
rs78228882
rs111432170
rs147612560
rs147071297
rs146899923
rs191753228
rs565801
rs687264
rs139127
rs138678985
rs2585739
rs12096152
rs1450949
rs4129441
rs35035328
rs116794175
rs28527279
rs116499679
rs181777922
rs12235775
rs117626397
rs62488624
rs2670003
rs4497244
rs146620586
rs61269136
rs13231184
rs35222020
rs138915779
rs117586381
rs72631887
rs112009416
rs78971171
rs138232290
rs76098298
rs11579717
rs77547518
rs11770198
rs74732038
rs76224258
All
−0.075
−0.050
−0.025
0.000
MR leave−one−out sensitivity analysis for
'ebi−a−GCST90001669' on 'outcome'

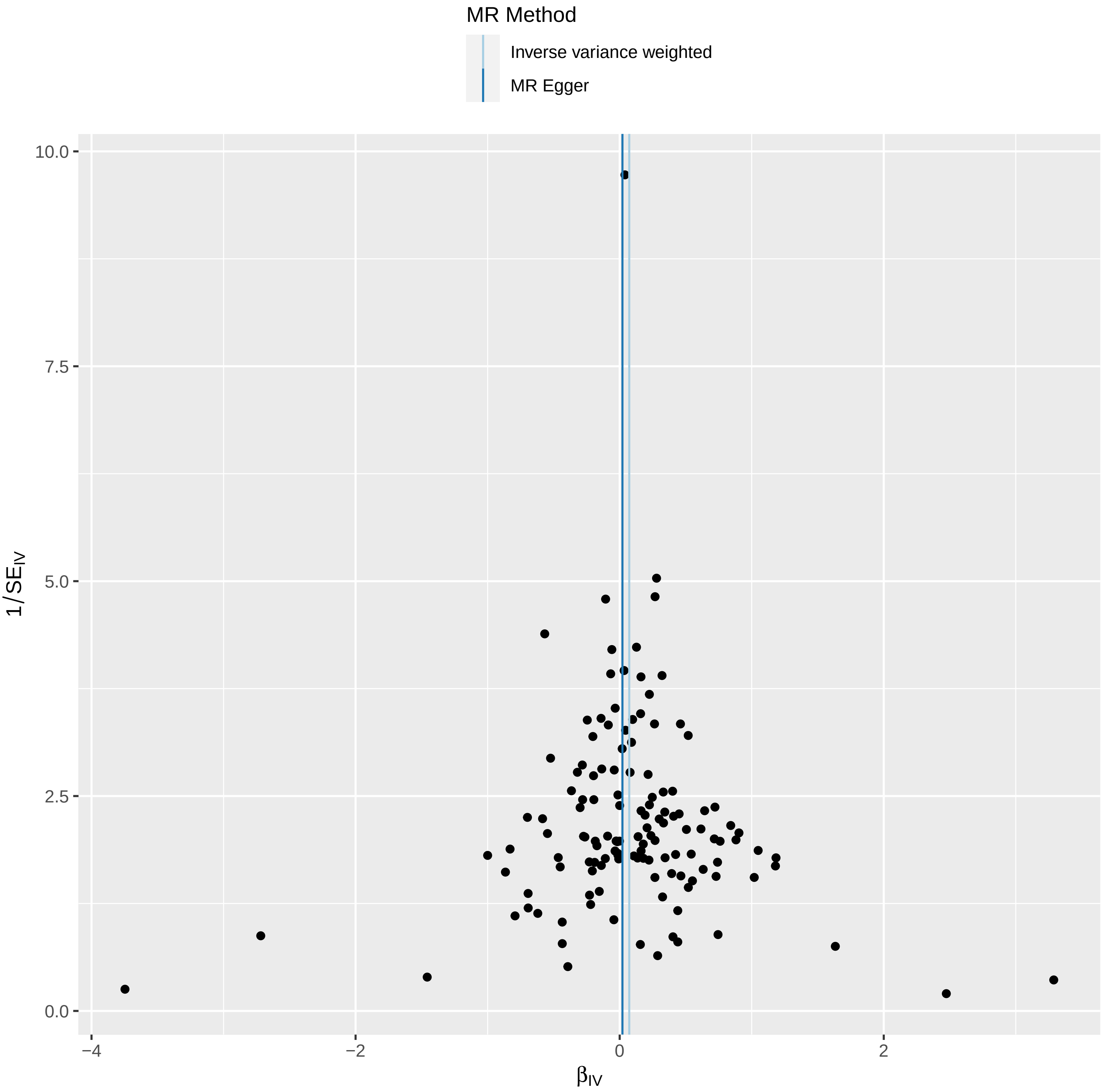
MR Method
Inverse variance weighted
MR Egger
10.0
7.5
5.0
2.5
0.0
1/SE$_{IV}$
−4
−2
0
2
β$_{IV}$

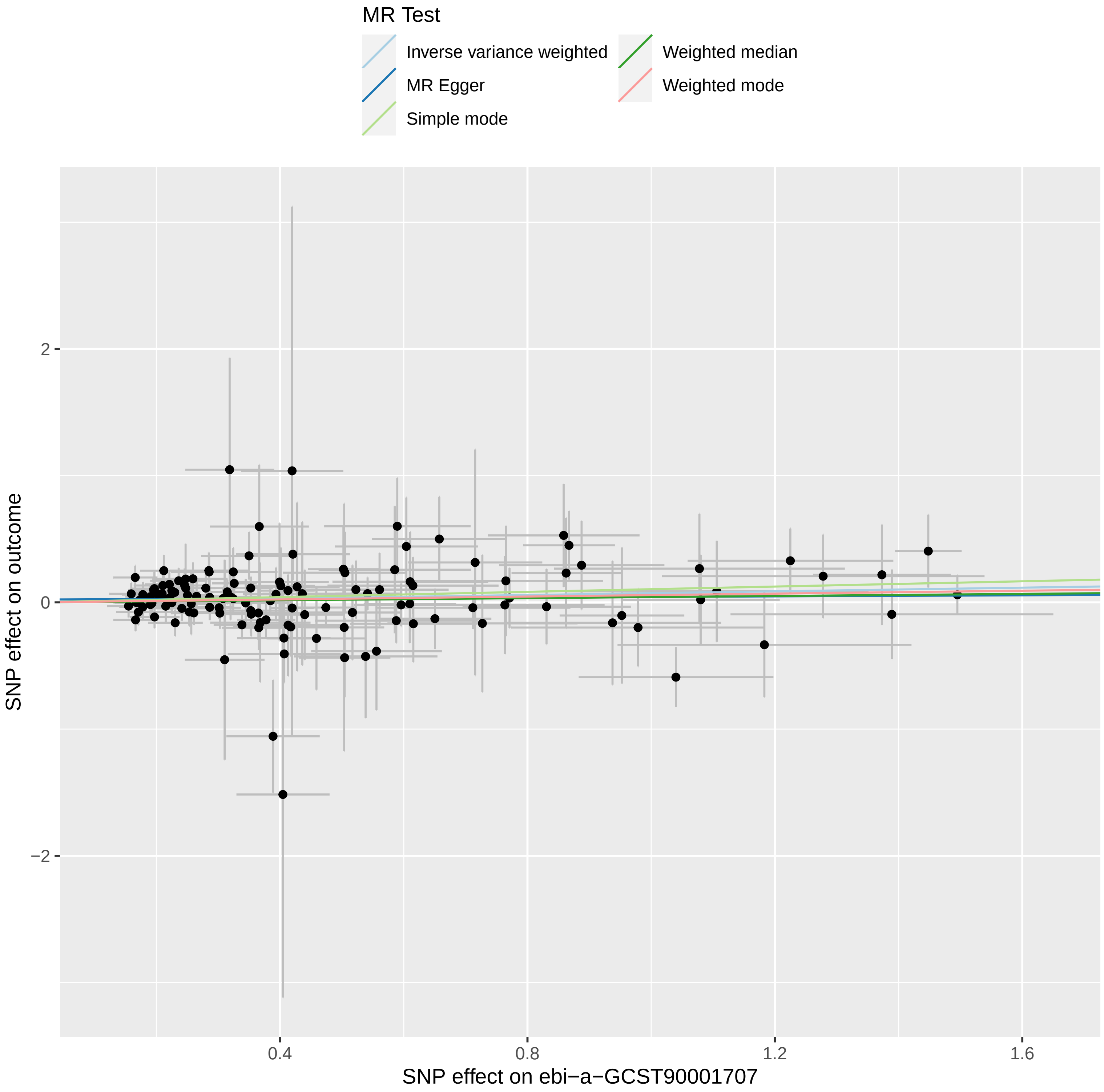
MR Test
Inverse variance weighted
MR Egger
Simple mode
Weighted median
Weighted mode
SNP effect on outcome
2
0
−2
0.4
0.8
1.2
1.6
SNP effect on ebi−a−GCST90001707

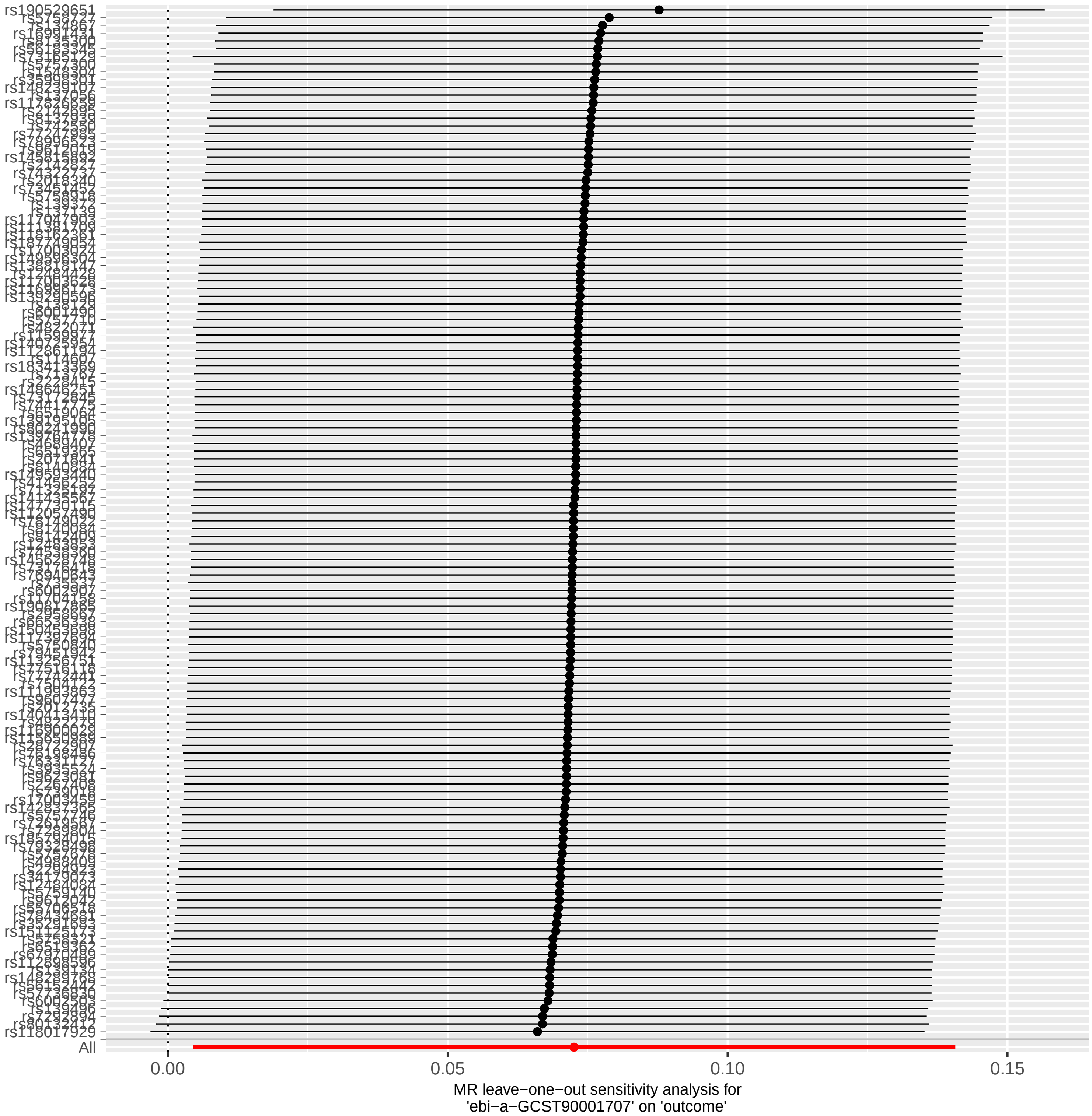
rs190529651
rs118017929
All
0.00
0.05
0.10
0.15
MR leave−one−out sensitivity analysis for
'ebi−a−GCST90001707' on 'outcome'

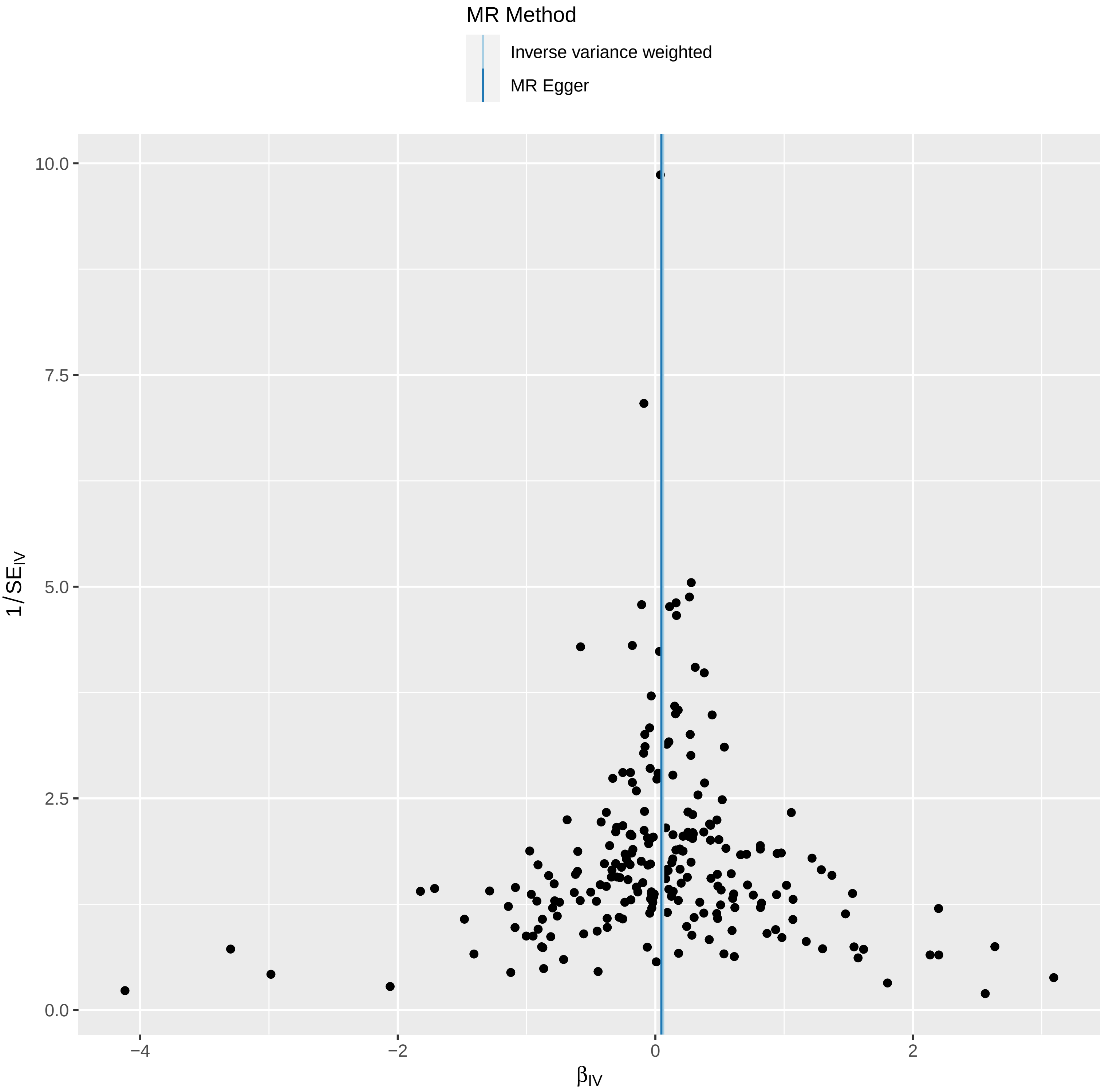
MR Method
Inverse variance weighted
MR Egger
10.0
7.5
5.0
2.5
0.0
$1/SE_{IV}$
−4
−2
0
2
β_{IV}

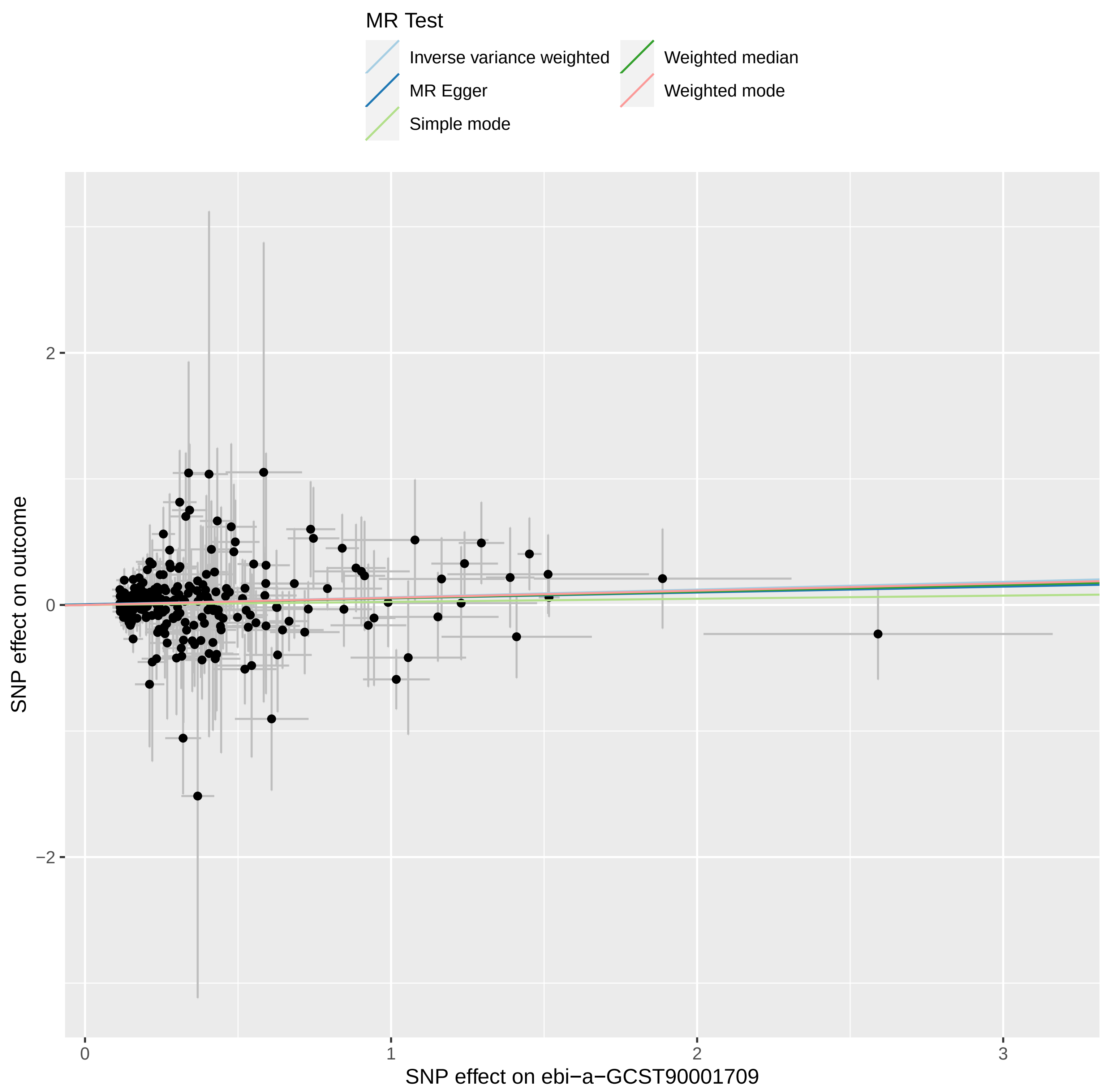
MR Test
Inverse variance weighted
MR Egger
Simple mode
Weighted median
Weighted mode
SNP effect on outcome
2
0
−2
0
1
2
3
SNP effect on ebi−a−GCST90001709

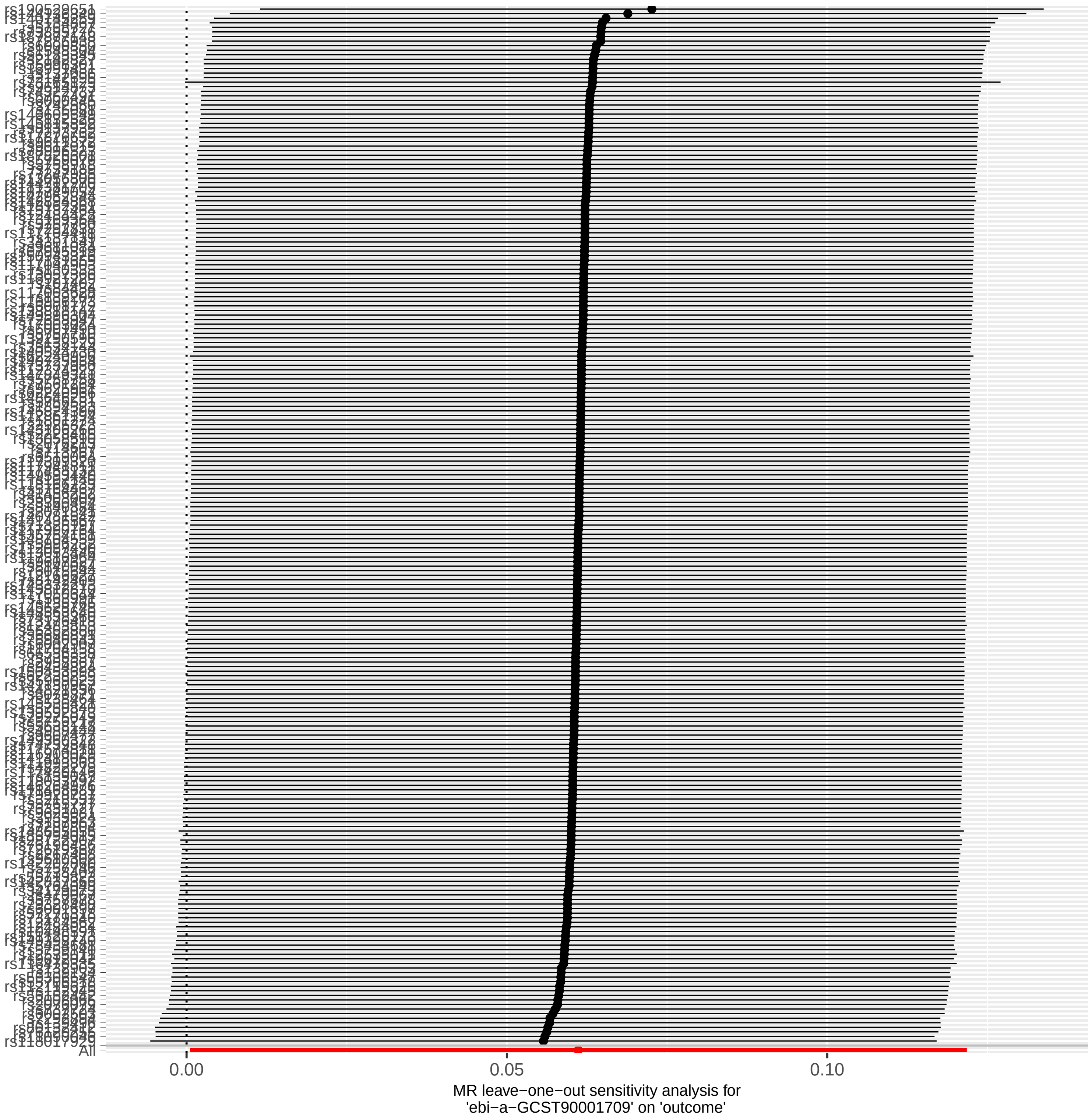

All
0.00
0.05
0.10
MR leave−one−out sensitivity analysis for
'ebi−a−GCST90001709' on 'outcome'

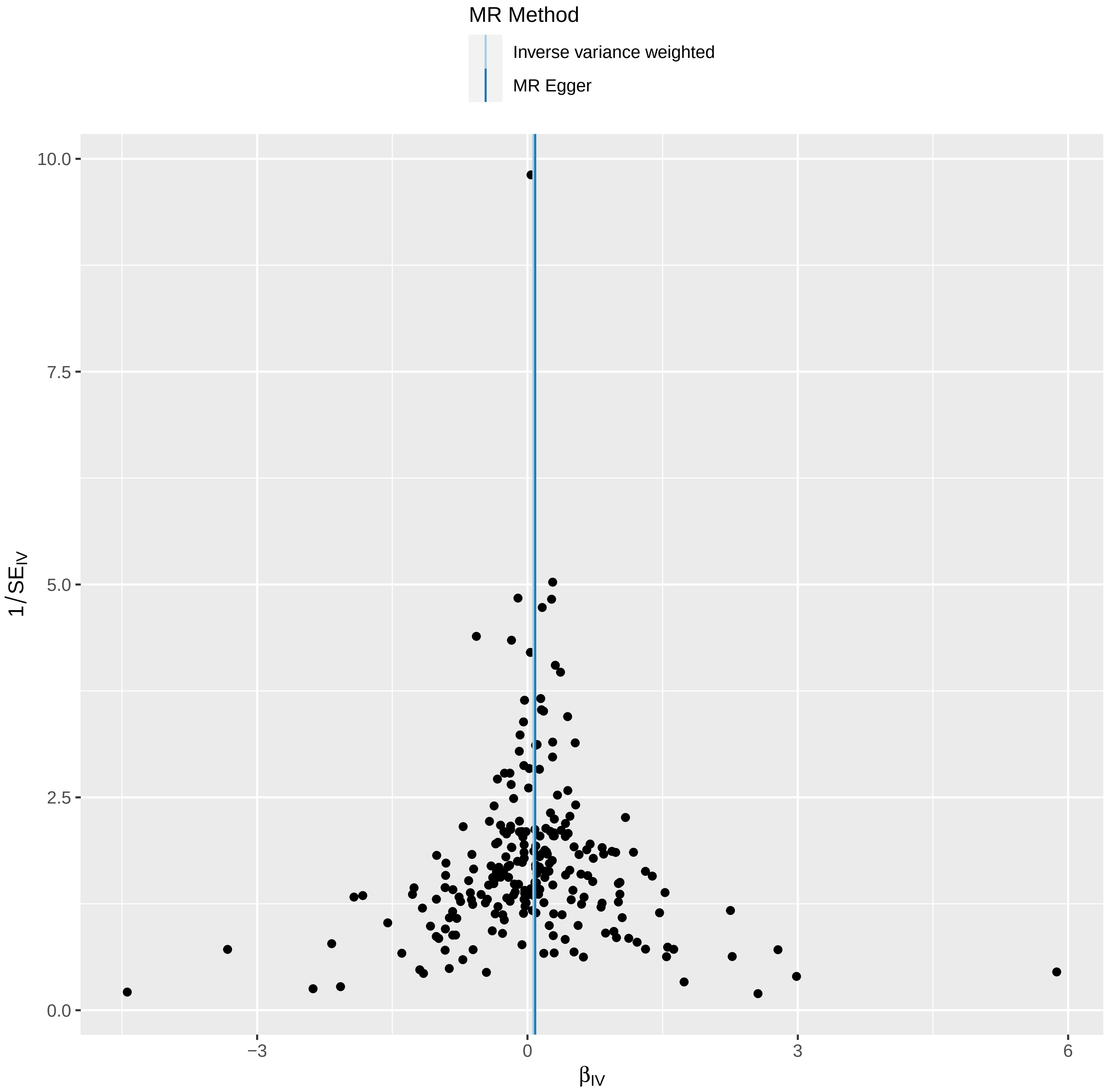

MR Method
Inverse variance weighted
MR Egger
10.0
7.5
5.0
2.5
0.0
$1/SE_{IV}$
−3
0
3
6
β_{IV}

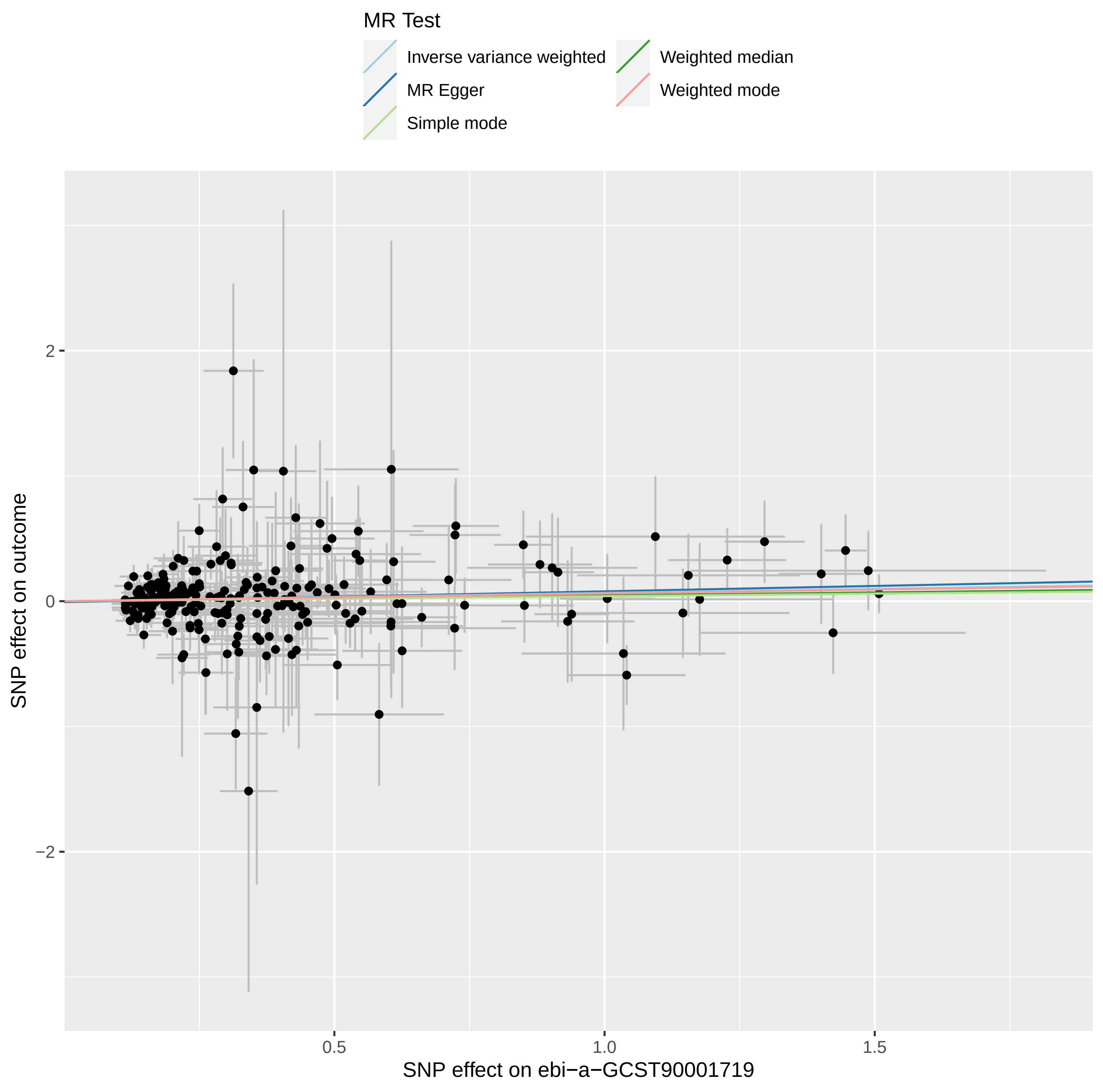

MR Test
Inverse variance weighted
MR Egger
Simple mode
Weighted median
Weighted mode
2
0
−2
SNP effect on outcome
0.5
1.0
1.5
SNP effect on ebi−a−GCST90001719

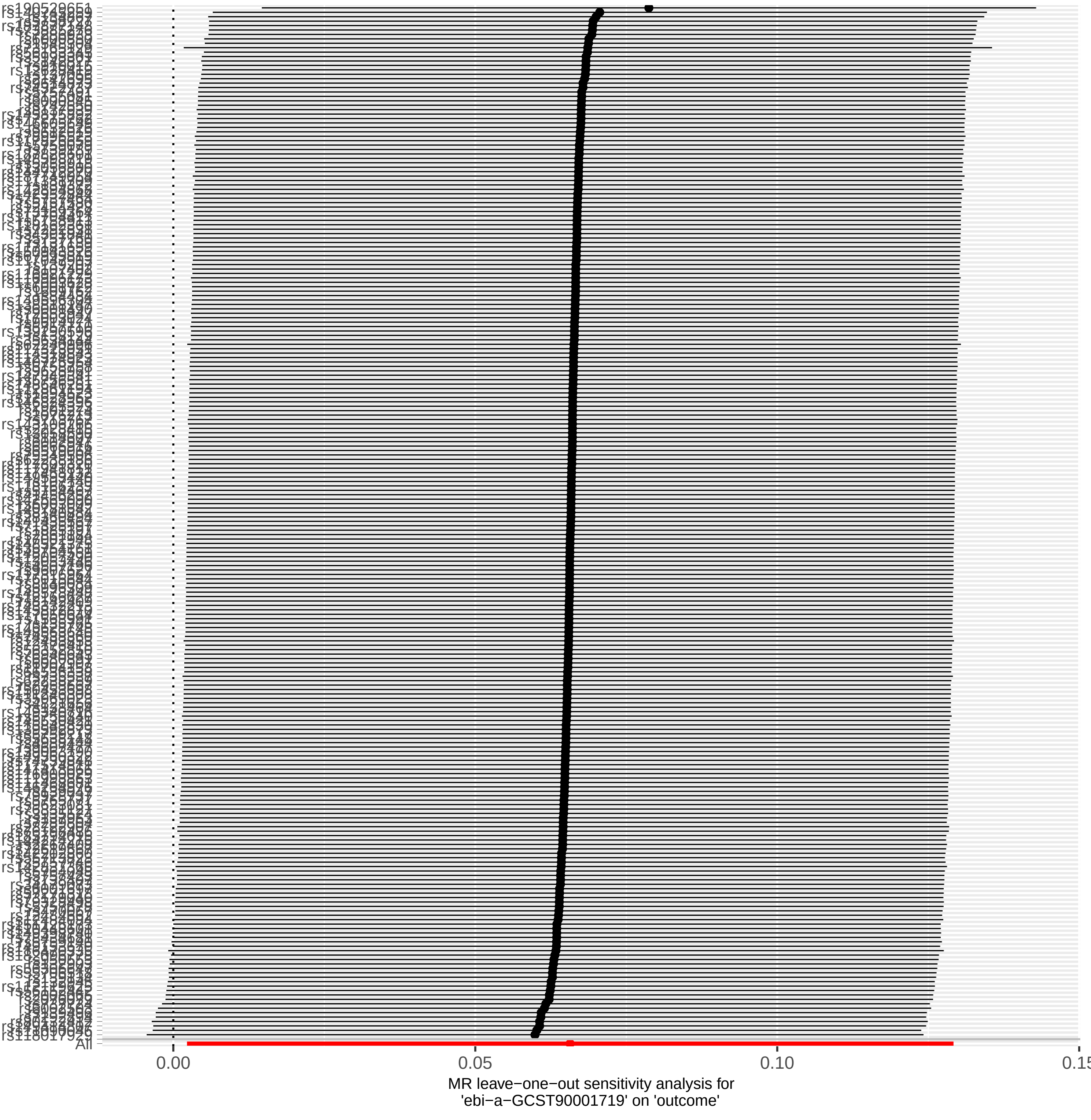
All
0.00
0.05
0.10
0.15
MR leave−one−out sensitivity analysis for
'ebi−a−GCST90001719' on 'outcome'

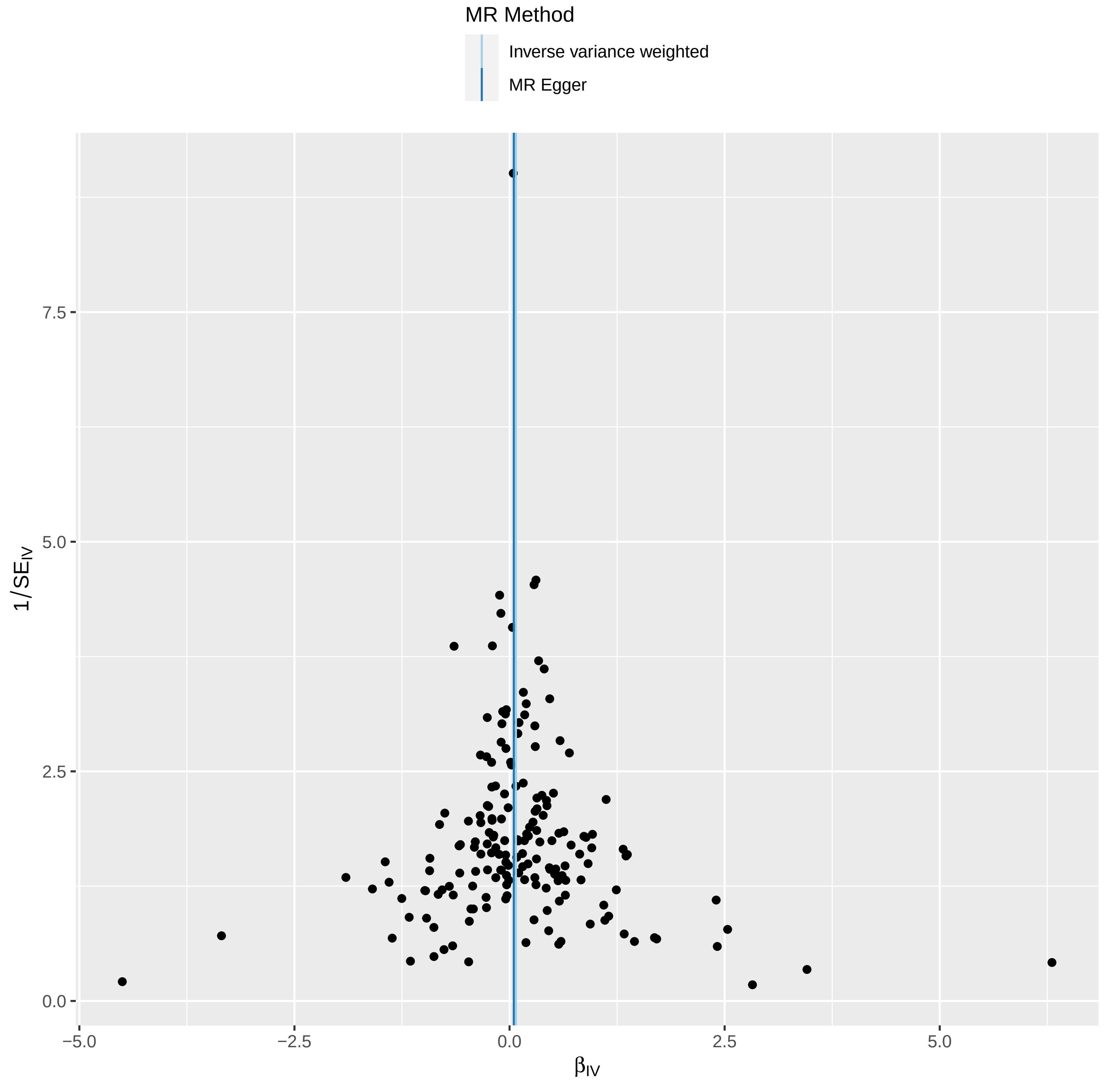

MR Method
Inverse variance weighted
MR Egger
1/SE_IV
β_IV
7.5
5.0
2.5
0.0
−5.0
−2.5
0.0
2.5
5.0

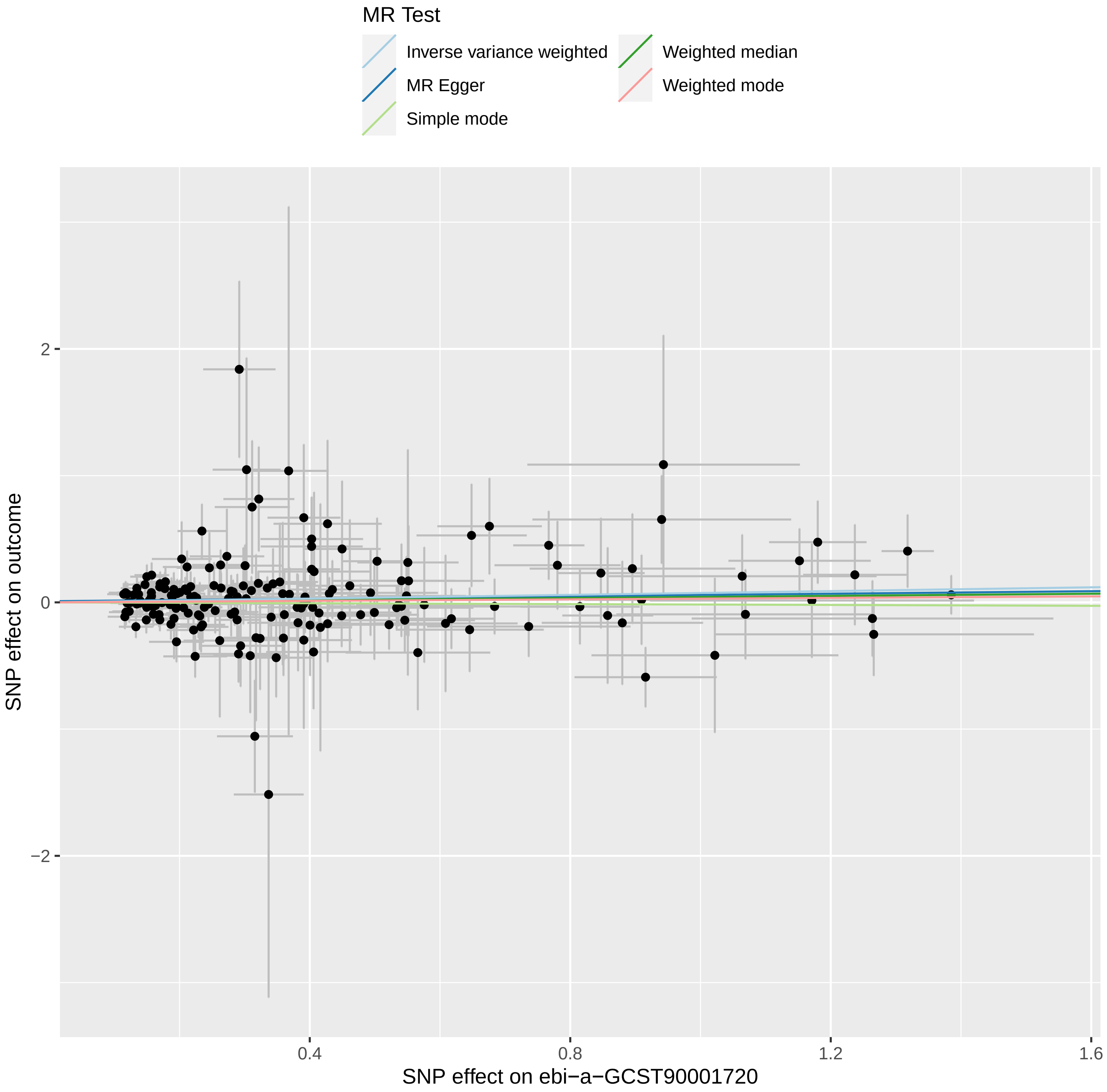

MR Test
Inverse variance weighted
MR Egger
Simple mode
Weighted median
Weighted mode
SNP effect on outcome
2
0
−2
0.4
0.8
1.2
1.6
SNP effect on ebi−a−GCST90001720

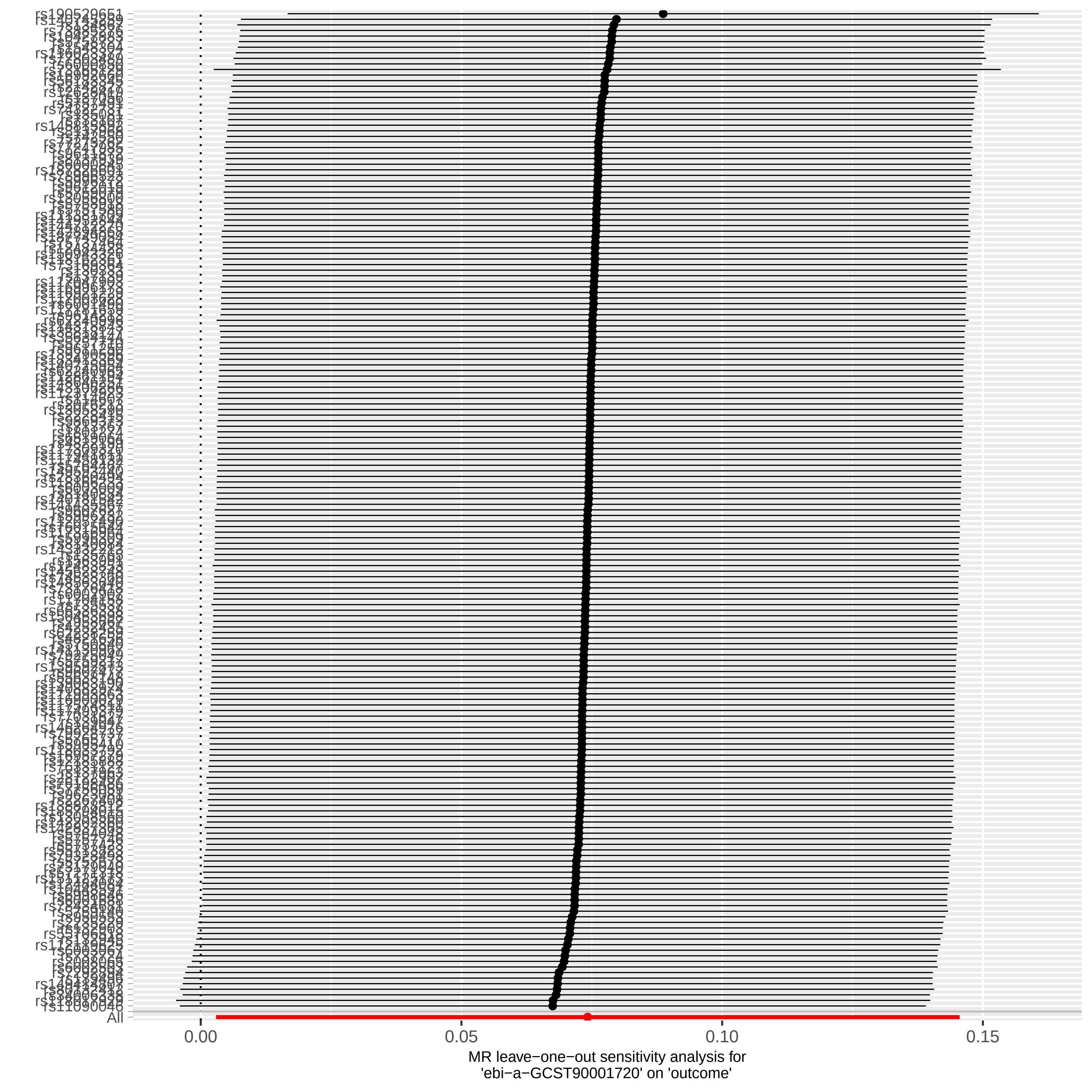
rs190529651
rs11090046
All
0.00
0.05
0.10
0.15
MR leave−one−out sensitivity analysis for
'ebi−a−GCST90001720' on 'outcome'

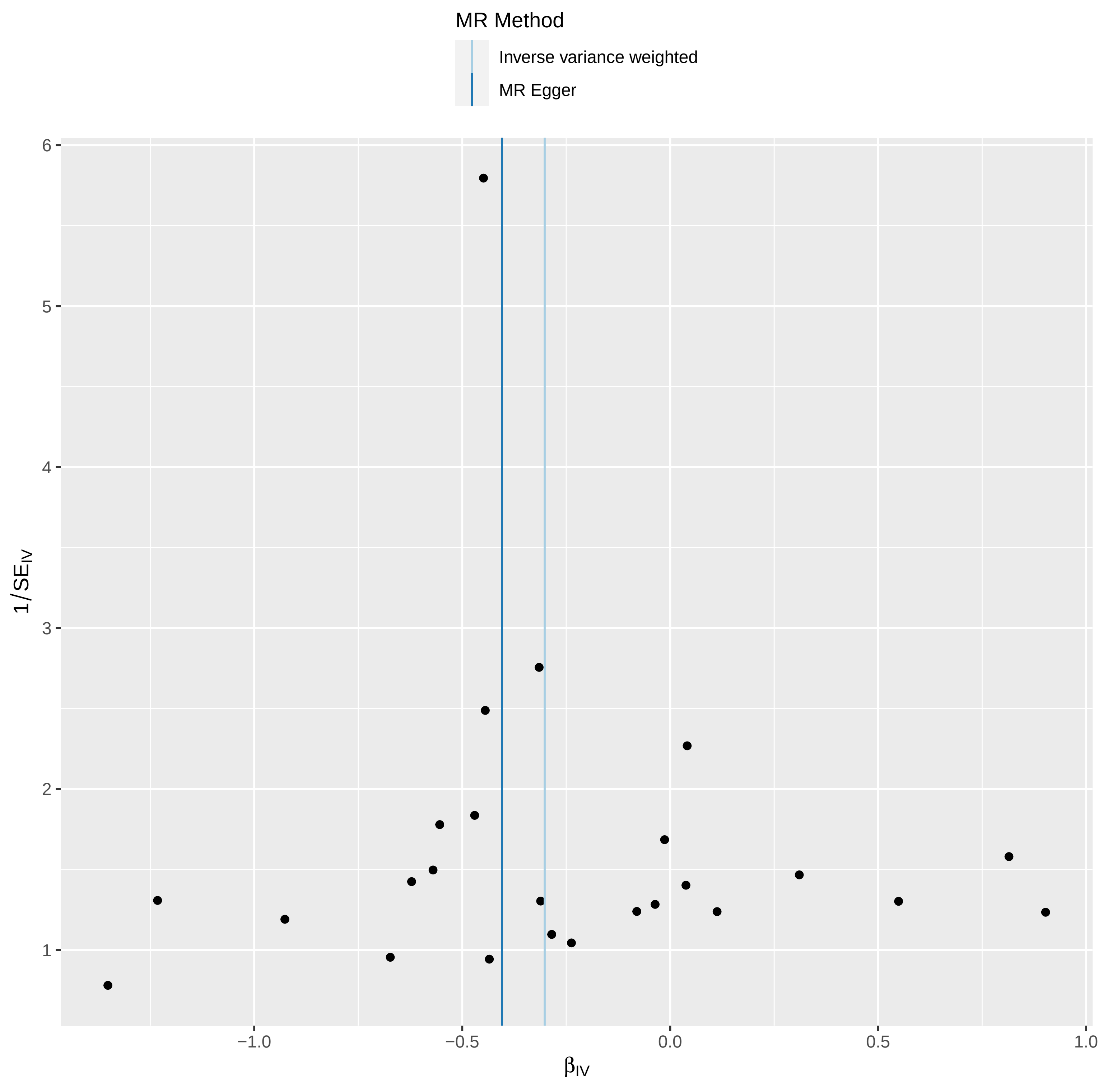
MR Method
Inverse variance weighted
MR Egger
1/SE$_{IV}$
β_{IV}
−1.0
−0.5
0.0
0.5
1.0
1
2
3
4
5
6

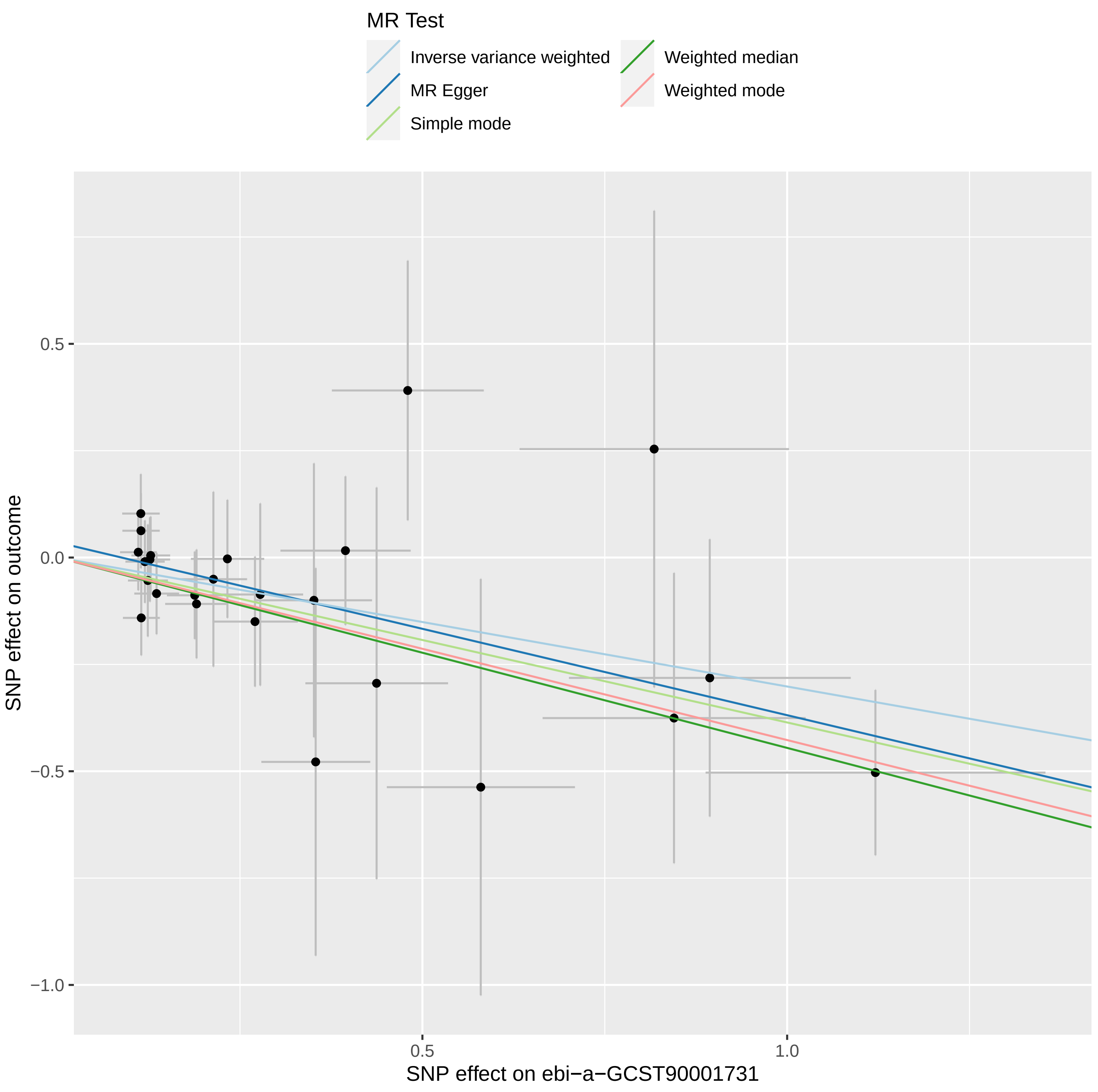
MR Test
Inverse variance weighted
MR Egger
Simple mode
Weighted median
Weighted mode
0.5
0.0
−0.5
−1.0
0.5
1.0
SNP effect on outcome
SNP effect on ebi−a−GCST90001731

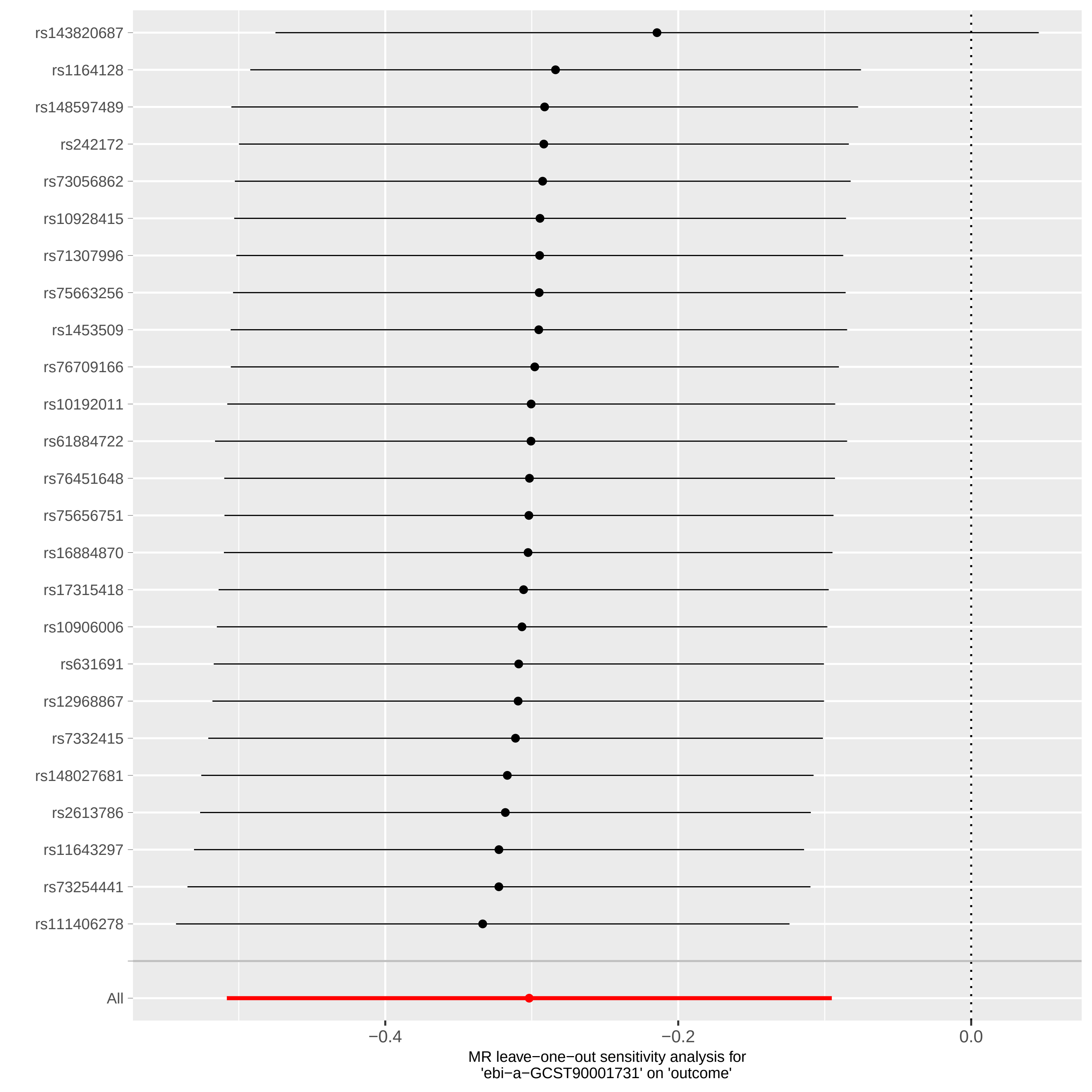
rs143820687
rs1164128
rs148597489
rs242172
rs73056862
rs10928415
rs71307996
rs75663256
rs1453509
rs76709166
rs10192011
rs61884722
rs76451648
rs75656751
rs16884870
rs17315418
rs10906006
rs631691
rs12968867
rs7332415
rs148027681
rs2613786
rs11643297
rs73254441
rs111406278
All
−0.4
−0.2
0.0
MR leave−one−out sensitivity analysis for
'ebi−a−GCST90001731' on 'outcome'

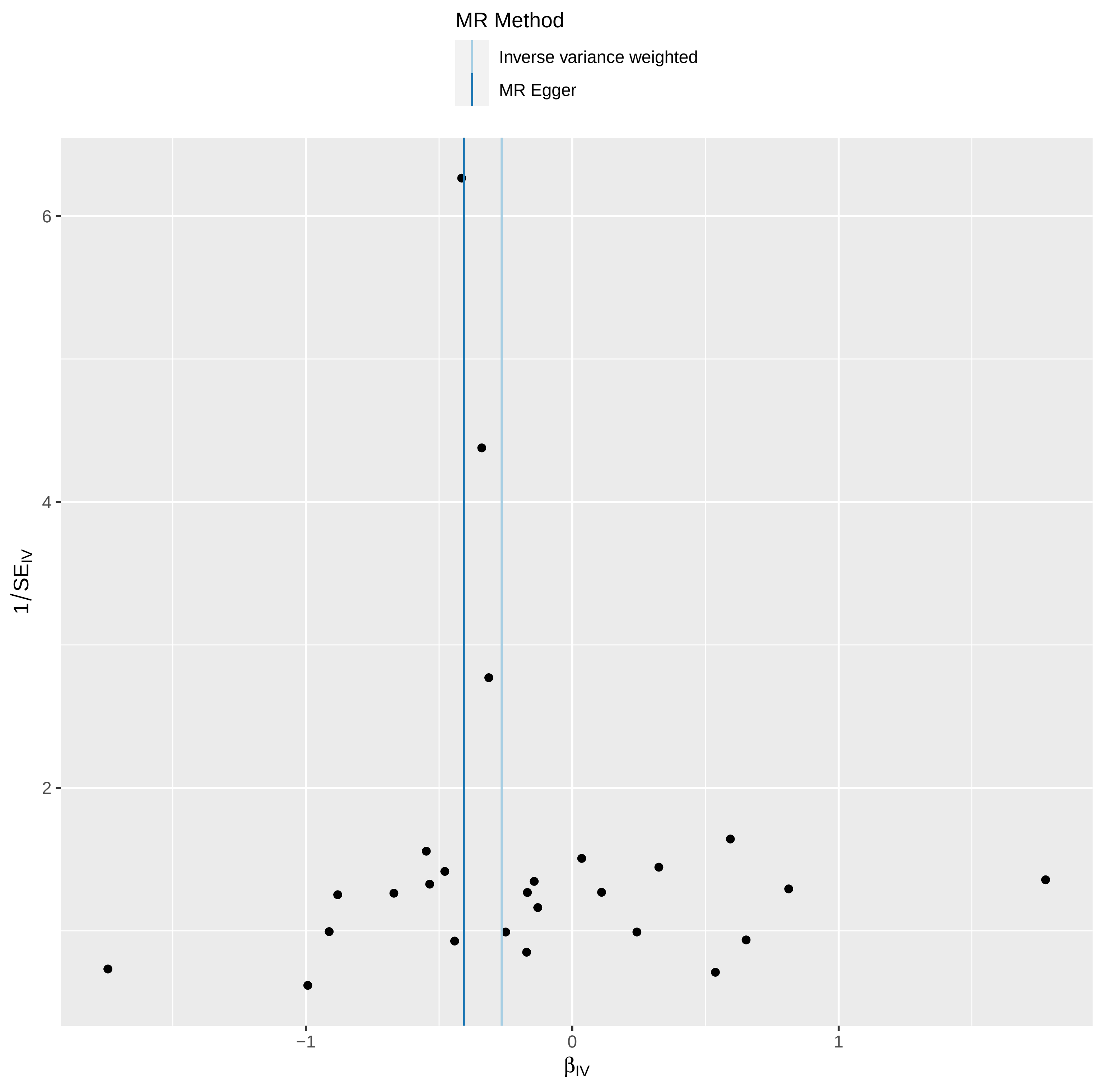

MR Method
Inverse variance weighted
MR Egger
6
4
2
1/SE$_{IV}$
−1
0
1
β$_{IV}$

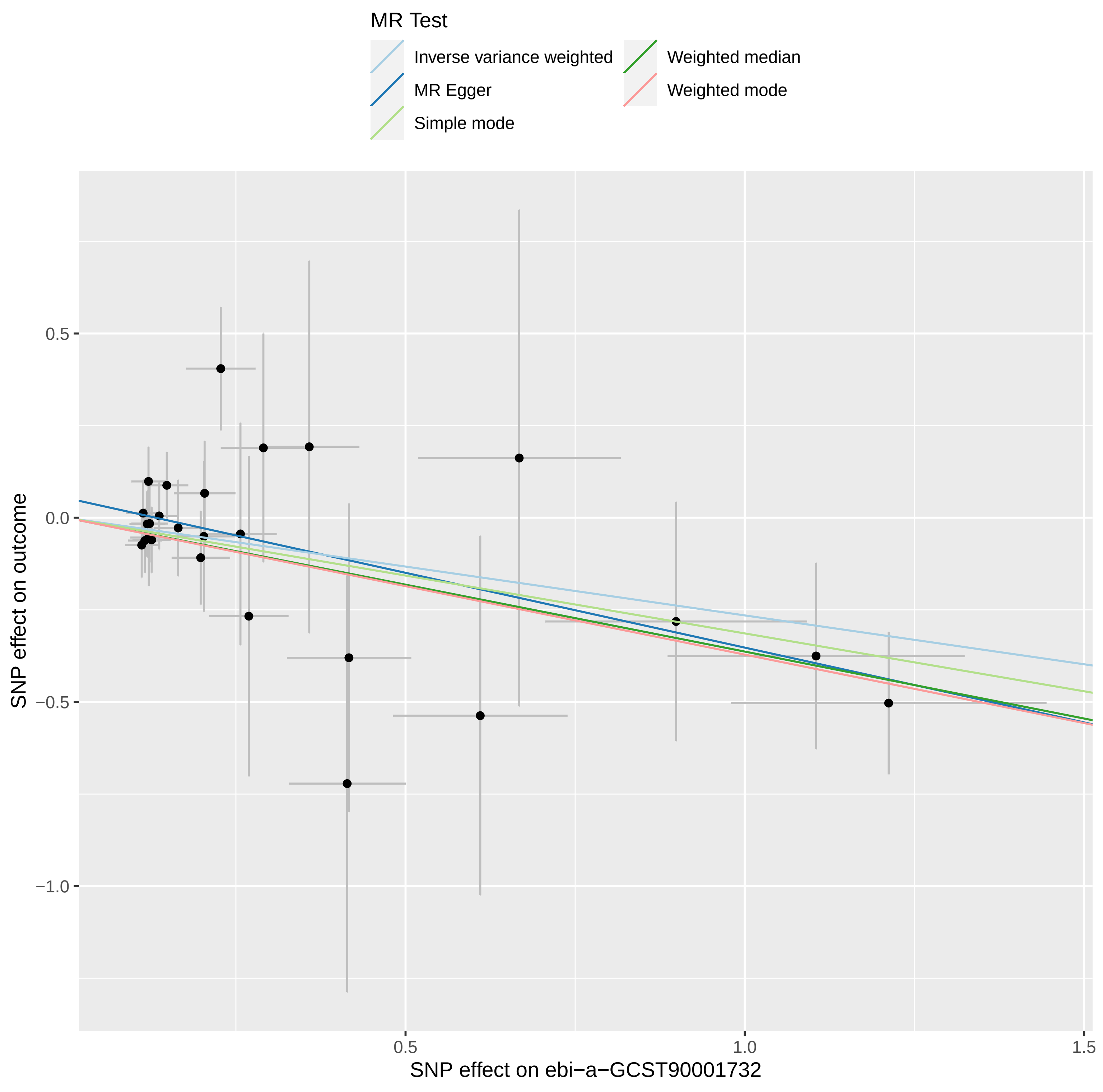
MR Test
Inverse variance weighted
MR Egger
Simple mode
Weighted median
Weighted mode
0.5
0.0
−0.5
−1.0
SNP effect on outcome
0.5
1.0
1.5
SNP effect on ebi−a−GCST90001732

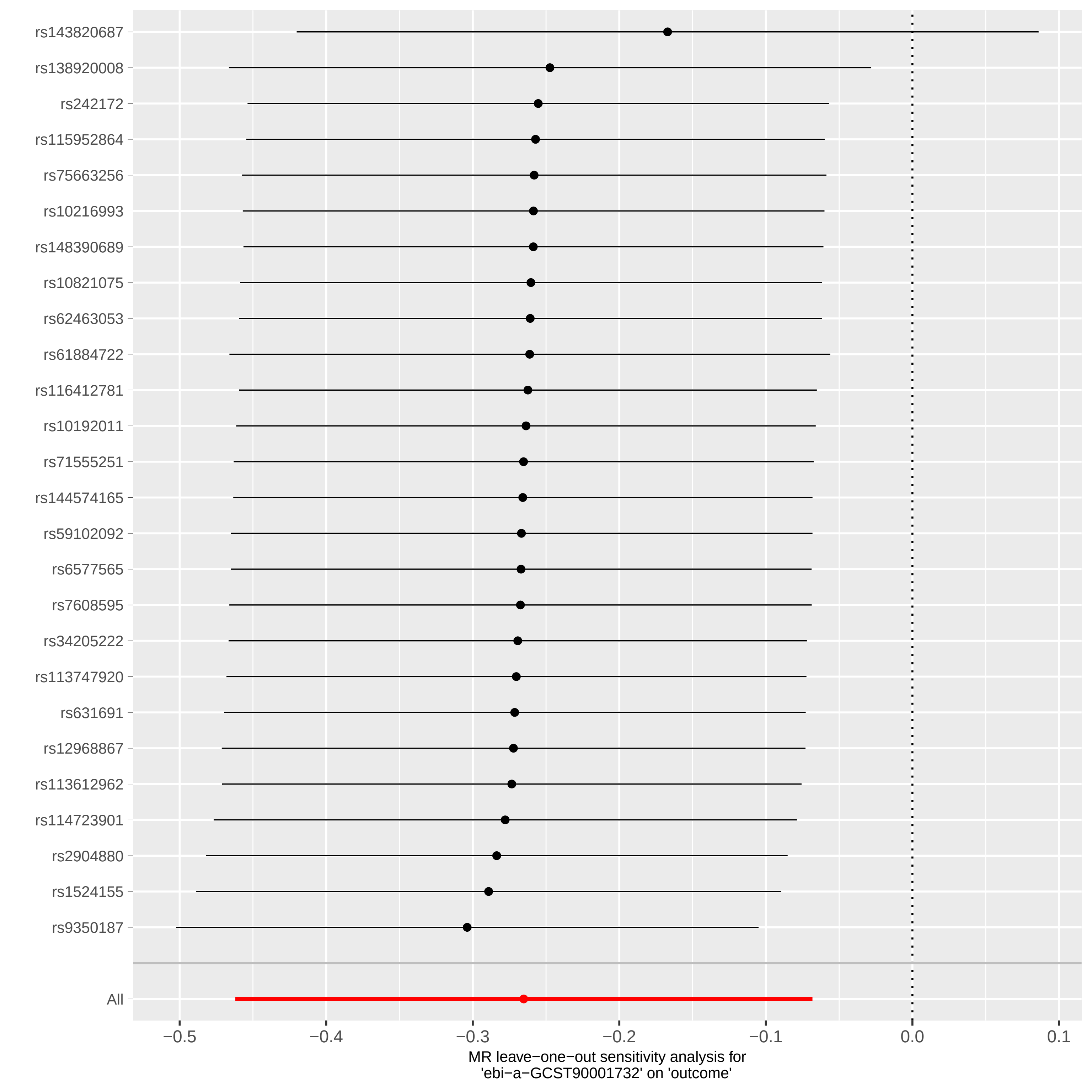

rs143820687
rs138920008
rs242172
rs115952864
rs75663256
rs10216993
rs148390689
rs10821075
rs62463053
rs61884722
rs116412781
rs10192011
rs71555251
rs144574165
rs59102092
rs6577565
rs7608595
rs34205222
rs113747920
rs631691
rs12968867
rs113612962
rs114723901
rs2904880
rs1524155
rs9350187
All
−0.5
−0.4
−0.3
−0.2
−0.1
0.0
0.1
MR leave−one−out sensitivity analysis for
'ebi−a−GCST90001732' on 'outcome'

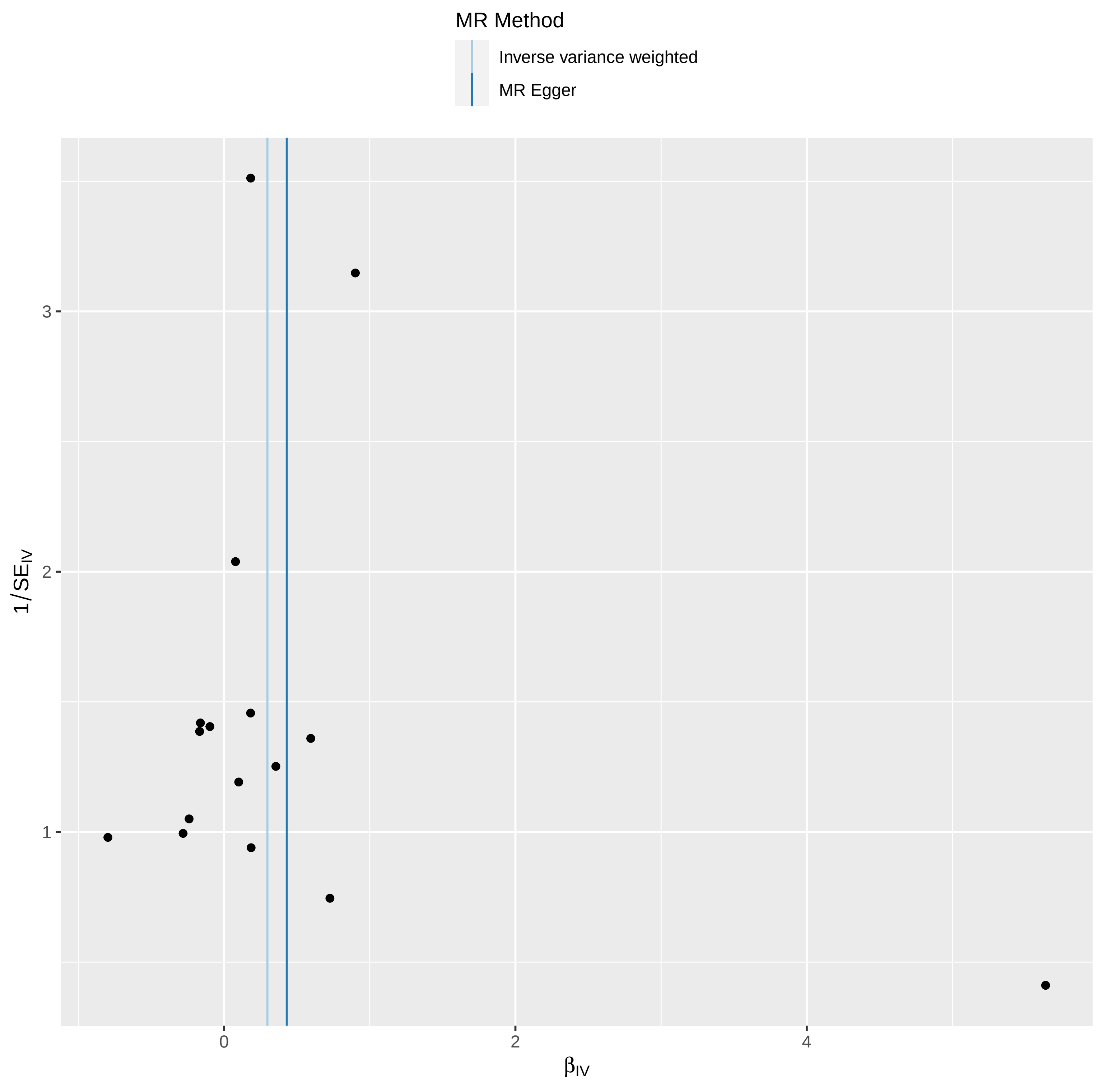
MR Method
Inverse variance weighted
MR Egger
1/SE_IV
3
2
1
0
2
4
β_IV

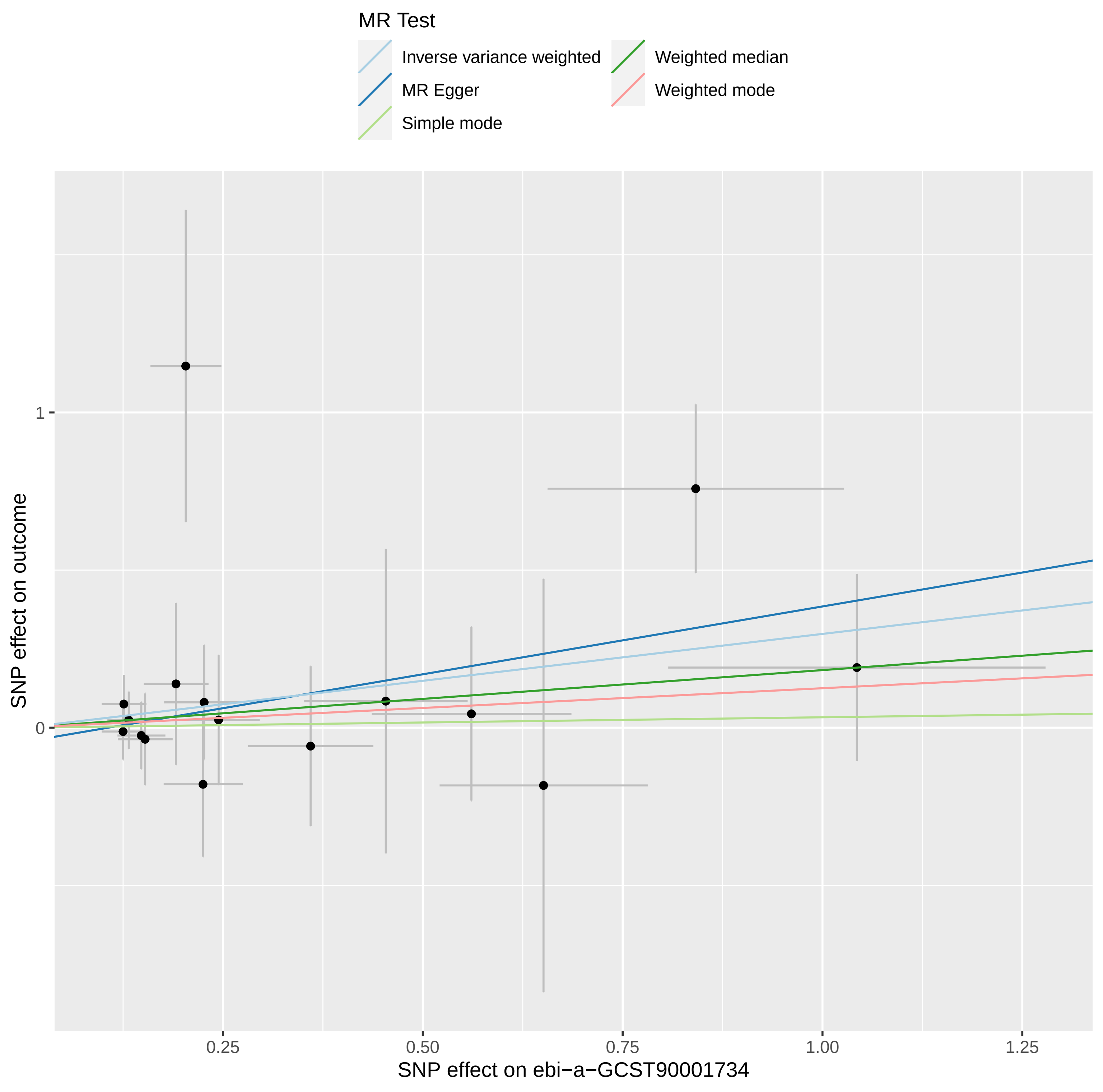

MR Test
Inverse variance weighted
MR Egger
Simple mode
Weighted median
Weighted mode
1
0
0.25
0.50
0.75
1.00
1.25
SNP effect on outcome
SNP effect on ebi−a−GCST90001734

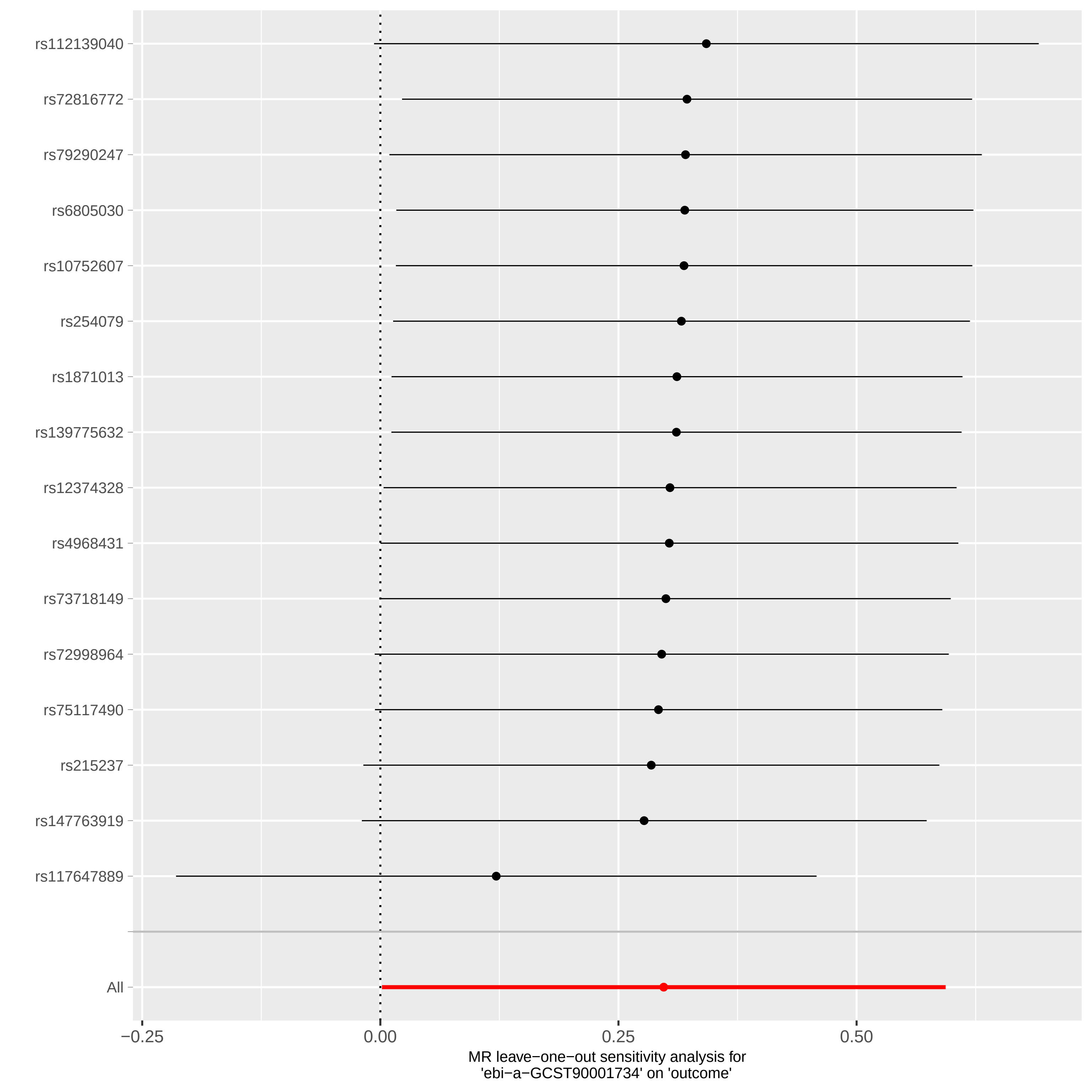
rs112139040
rs72816772
rs79290247
rs6805030
rs10752607
rs254079
rs1871013
rs139775632
rs12374328
rs4968431
rs73718149
rs72998964
rs75117490
rs215237
rs147763919
rs117647889
All
−0.25
0.00
0.25
0.50
MR leave−one−out sensitivity analysis for
'ebi−a−GCST90001734' on 'outcome'

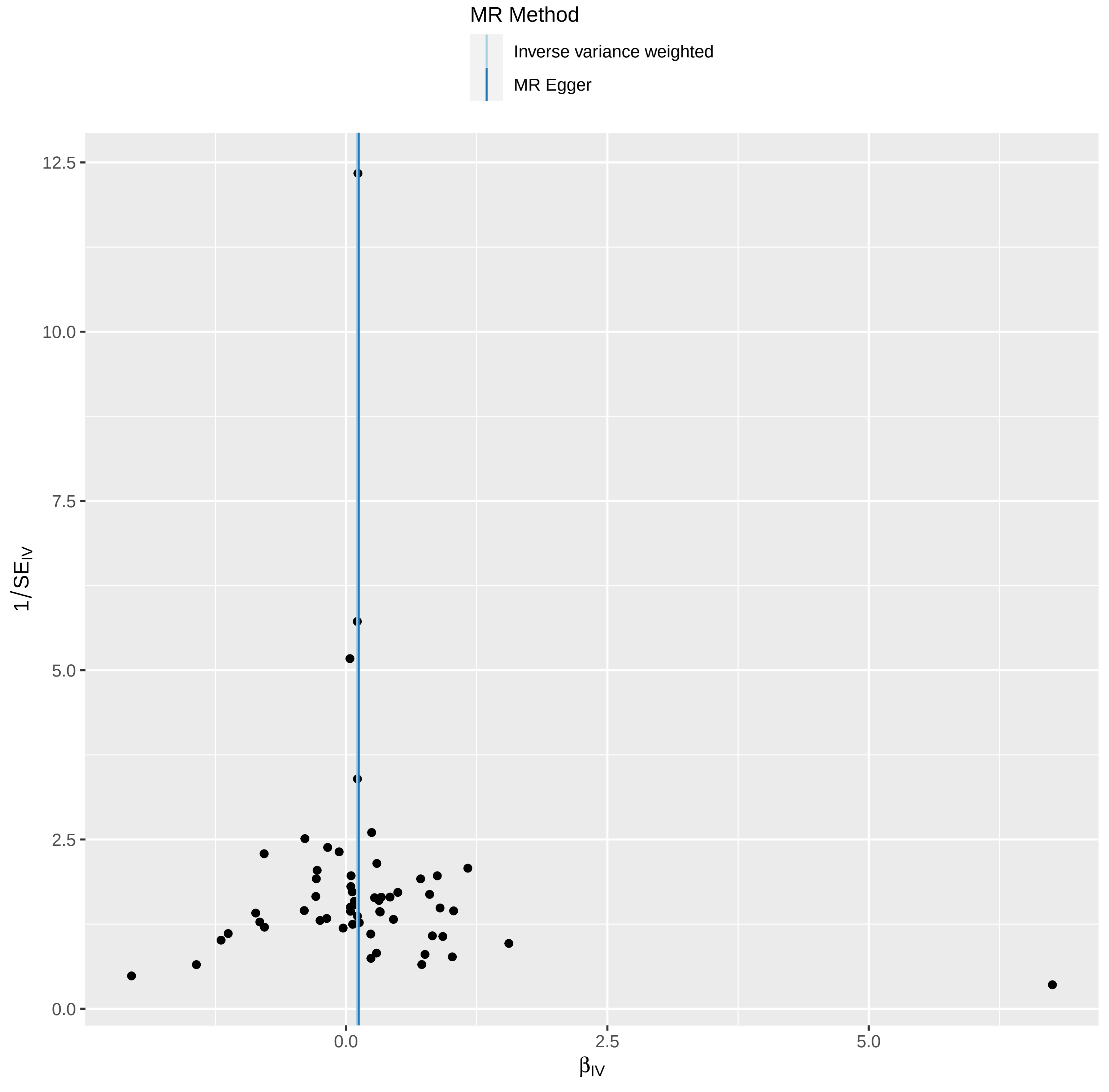

MR Method
Inverse variance weighted
MR Egger
12.5
10.0
7.5
5.0
2.5
0.0
1/SE_IV
0.0
2.5
5.0
β_IV

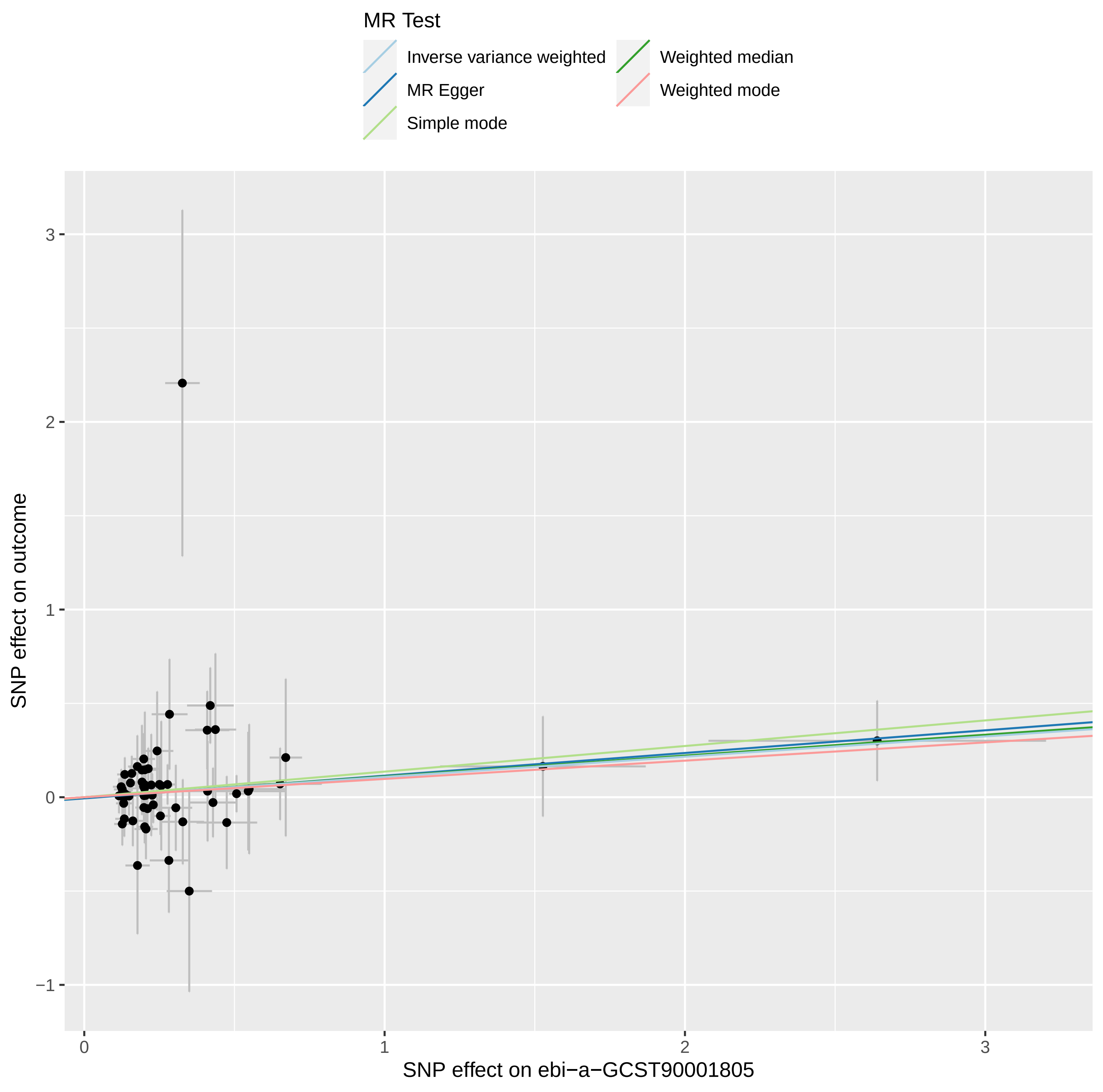
MR Test
Inverse variance weighted
MR Egger
Simple mode
Weighted median
Weighted mode
SNP effect on outcome
SNP effect on ebi−a−GCST90001805
3
2
1
0
−1
0
1
2
3

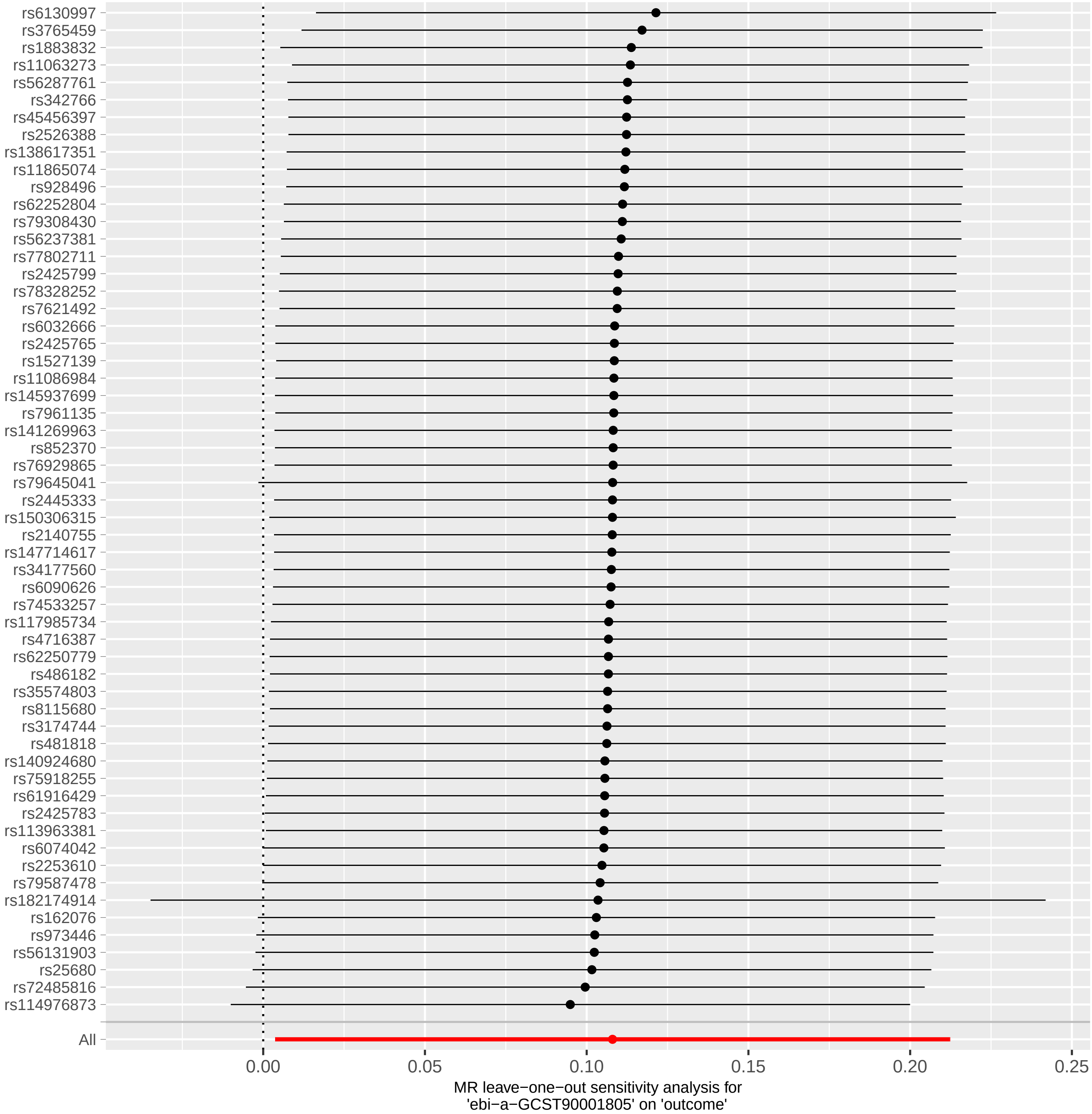
rs6130997
rs3765459
rs1883832
rs11063273
rs56287761
rs342766
rs45456397
rs2526388
rs138617351
rs11865074
rs928496
rs62252804
rs79308430
rs56237381
rs77802711
rs2425799
rs78328252
rs7621492
rs6032666
rs2425765
rs1527139
rs11086984
rs145937699
rs7961135
rs141269963
rs852370
rs76929865
rs79645041
rs2445333
rs150306315
rs2140755
rs147714617
rs34177560
rs6090626
rs74533257
rs117985734
rs4716387
rs62250779
rs486182
rs35574803
rs8115680
rs3174744
rs481818
rs140924680
rs75918255
rs61916429
rs2425783
rs113963381
rs6074042
rs2253610
rs79587478
rs182174914
rs162076
rs973446
rs56131903
rs25680
rs72485816
rs114976873
All
0.00
0.05
0.10
0.15
0.20
0.25
MR leave−one−out sensitivity analysis for
'ebi−a−GCST90001805' on 'outcome'

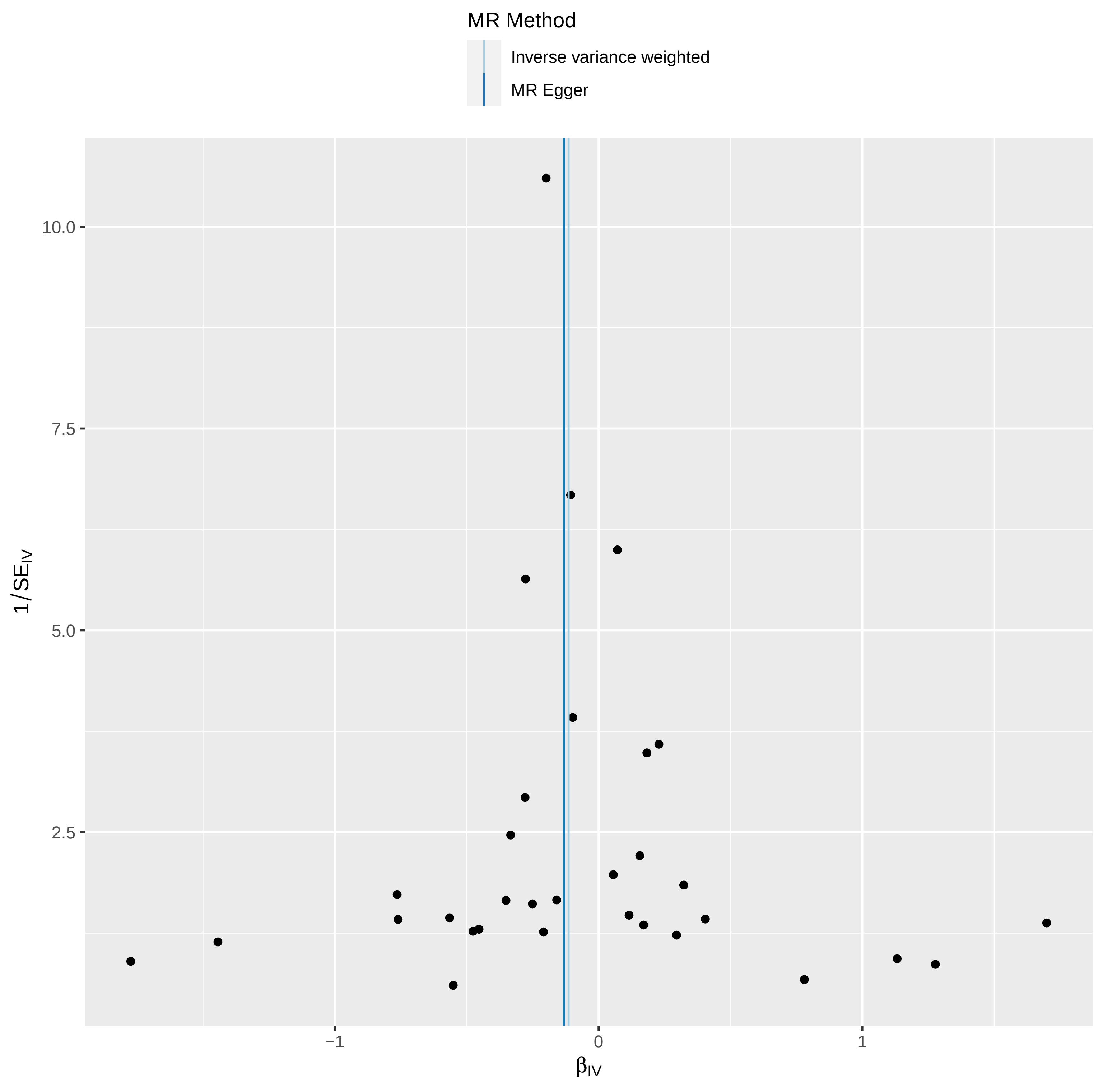
MR Method
Inverse variance weighted
MR Egger
10.0
7.5
5.0
2.5
1/SE$_{IV}$
−1
0
1
β$_{IV}$

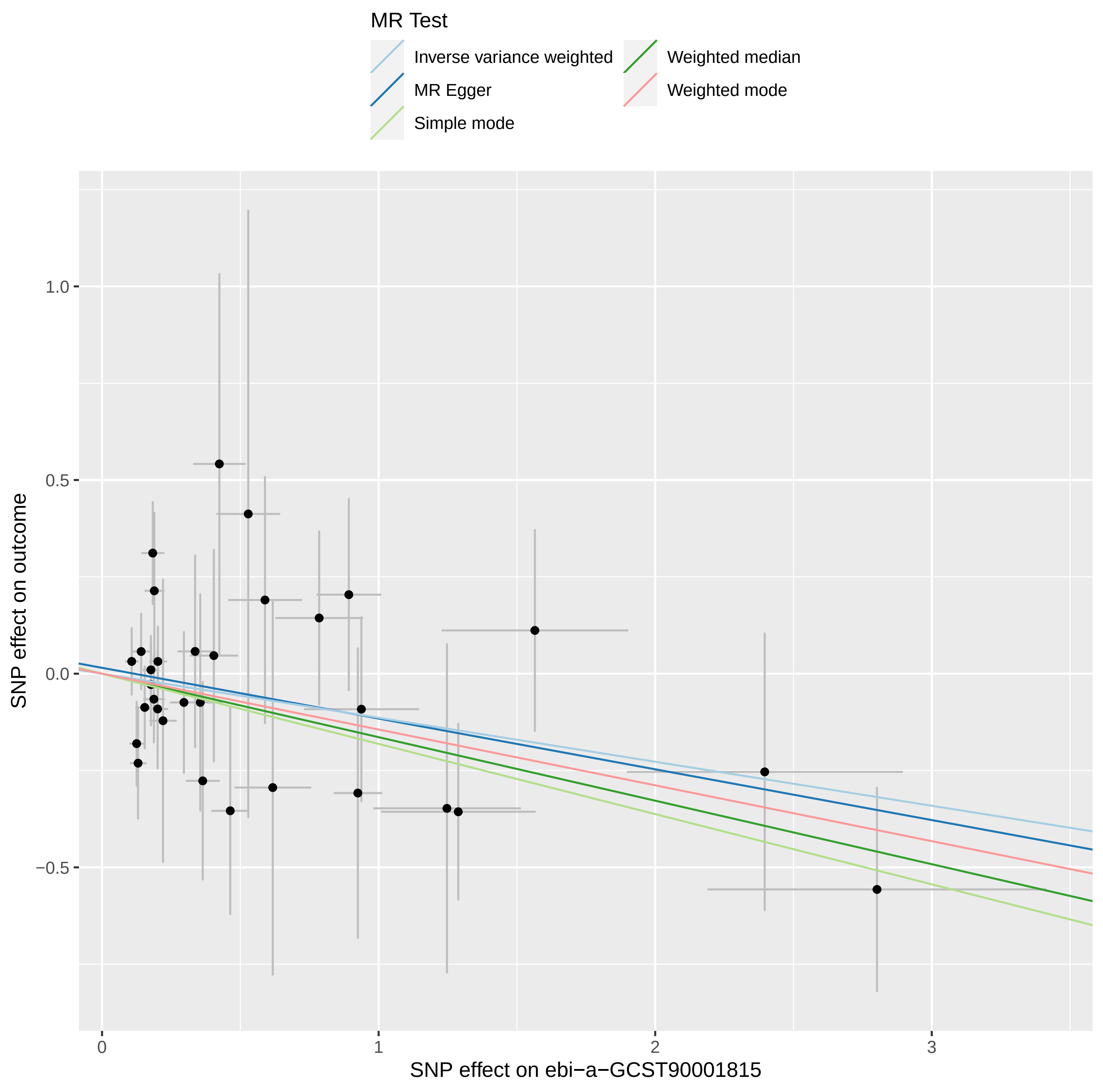

MR Test
Inverse variance weighted
MR Egger
Simple mode
Weighted median
Weighted mode
1.0
0.5
0.0
−0.5
0
1
2
3
SNP effect on outcome
SNP effect on ebi−a−GCST90001815

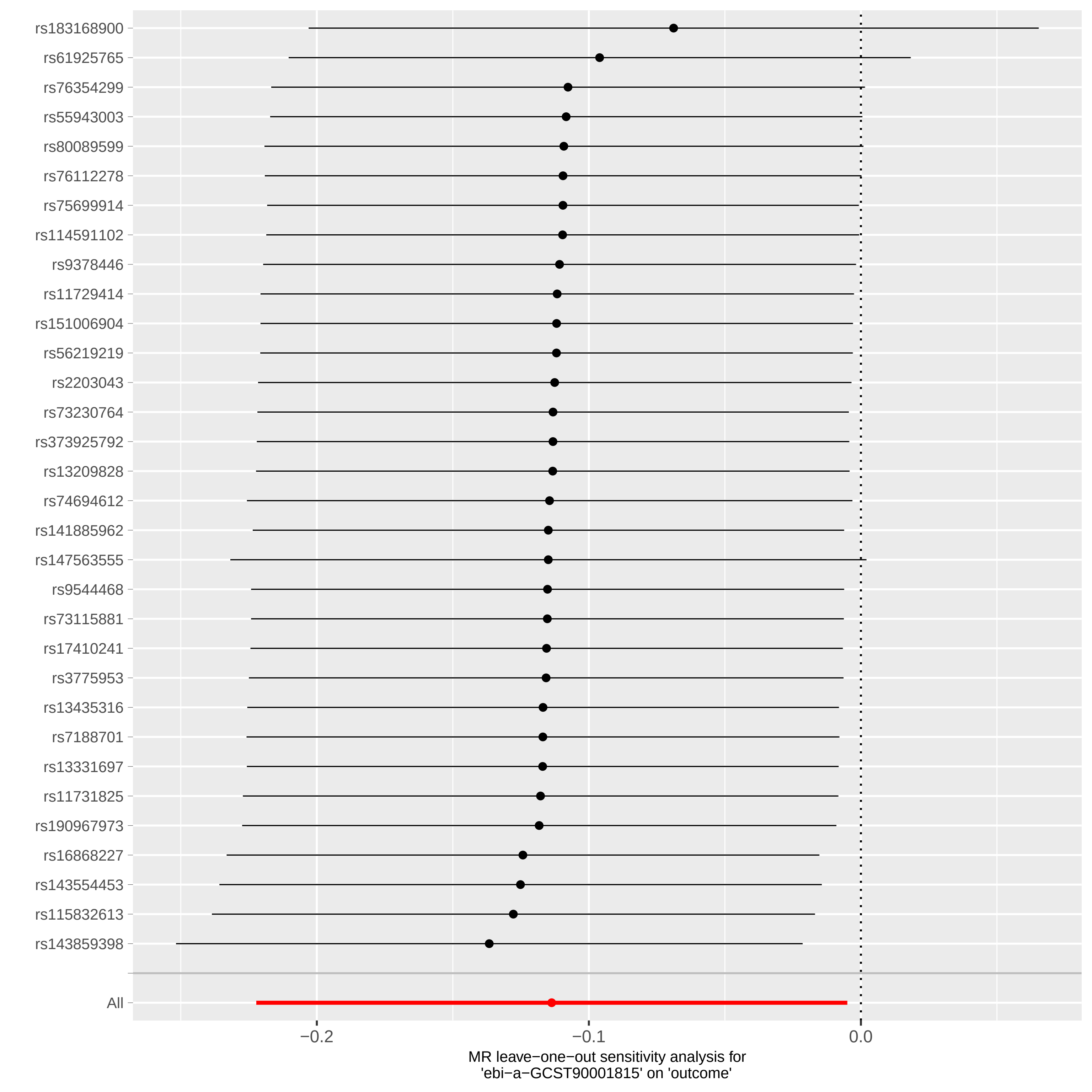
rs183168900
rs61925765
rs76354299
rs55943003
rs80089599
rs76112278
rs75699914
rs114591102
rs9378446
rs11729414
rs151006904
rs56219219
rs2203043
rs73230764
rs373925792
rs13209828
rs74694612
rs141885962
rs147563555
rs9544468
rs73115881
rs17410241
rs3775953
rs13435316
rs7188701
rs13331697
rs11731825
rs190967973
rs16868227
rs143554453
rs115832613
rs143859398
All
−0.2
−0.1
0.0
MR leave−one−out sensitivity analysis for
'ebi−a−GCST90001815' on 'outcome'

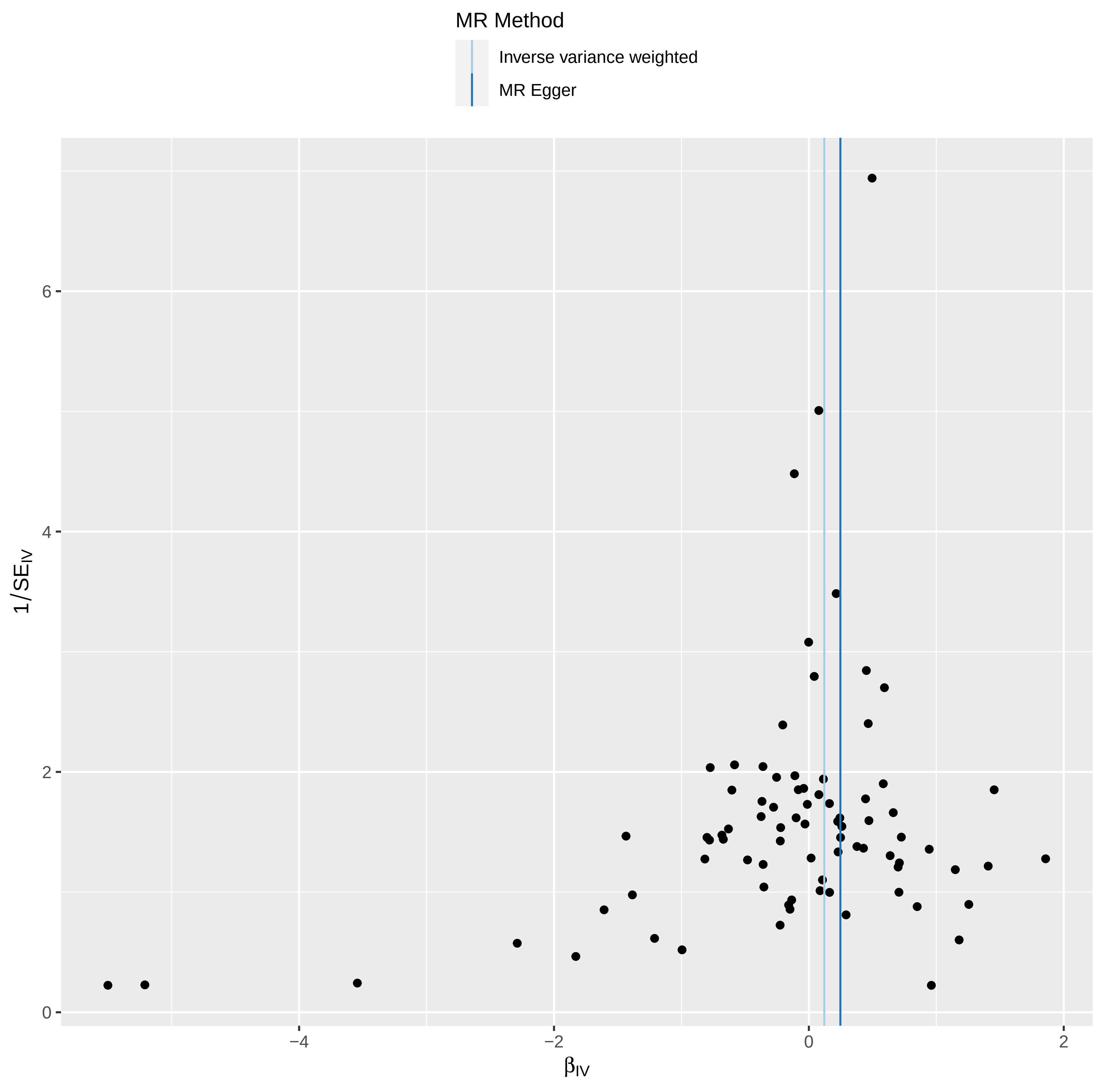

MR Method
Inverse variance weighted
MR Egger
6
4
2
0
1/SE$_{IV}$
−4
−2
0
2
β_{IV}

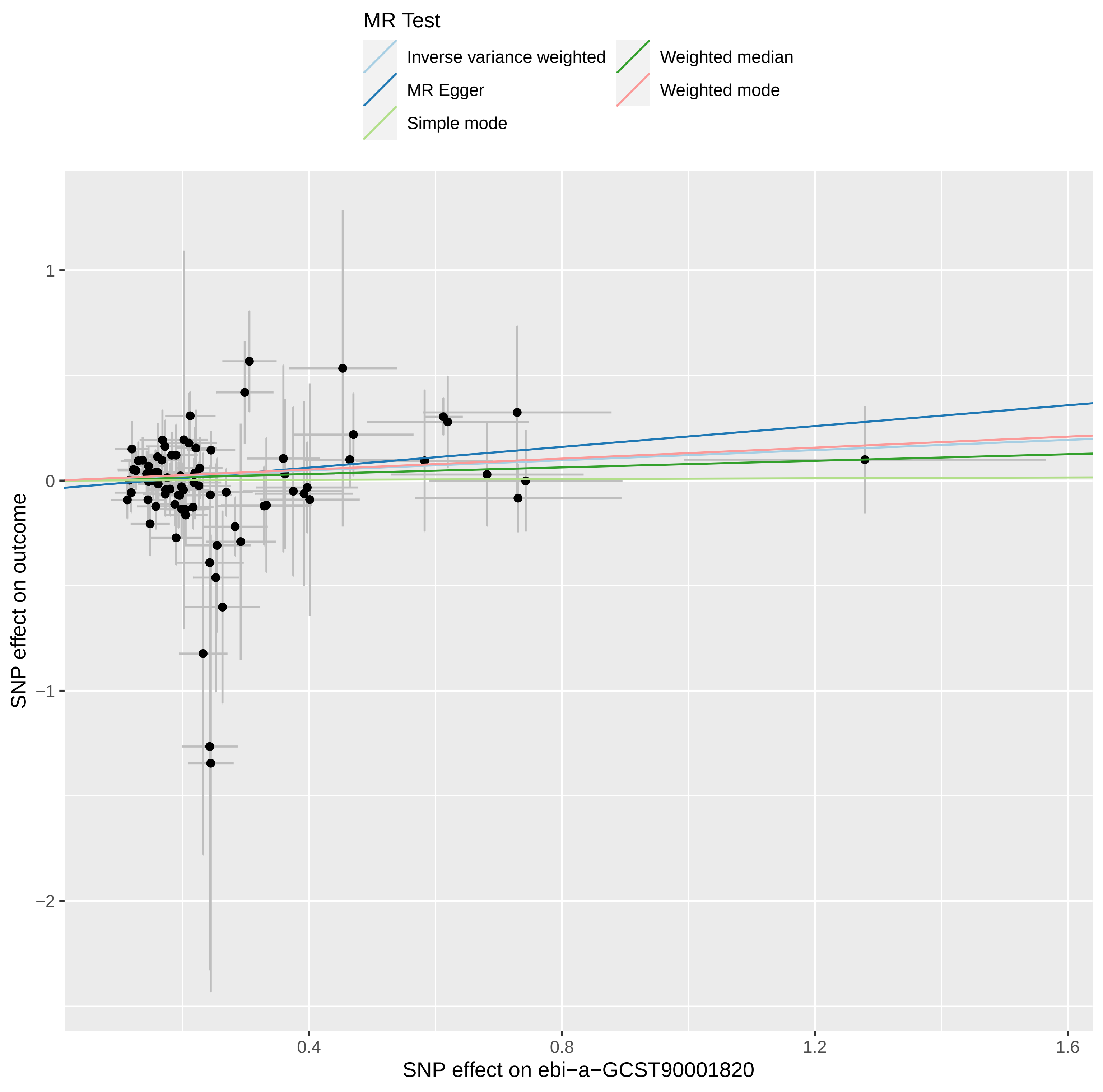

MR Test
Inverse variance weighted
MR Egger
Simple mode
Weighted median
Weighted mode
SNP effect on outcome
1
0
−1
−2
0.4
0.8
1.2
1.6
SNP effect on ebi−a−GCST90001820

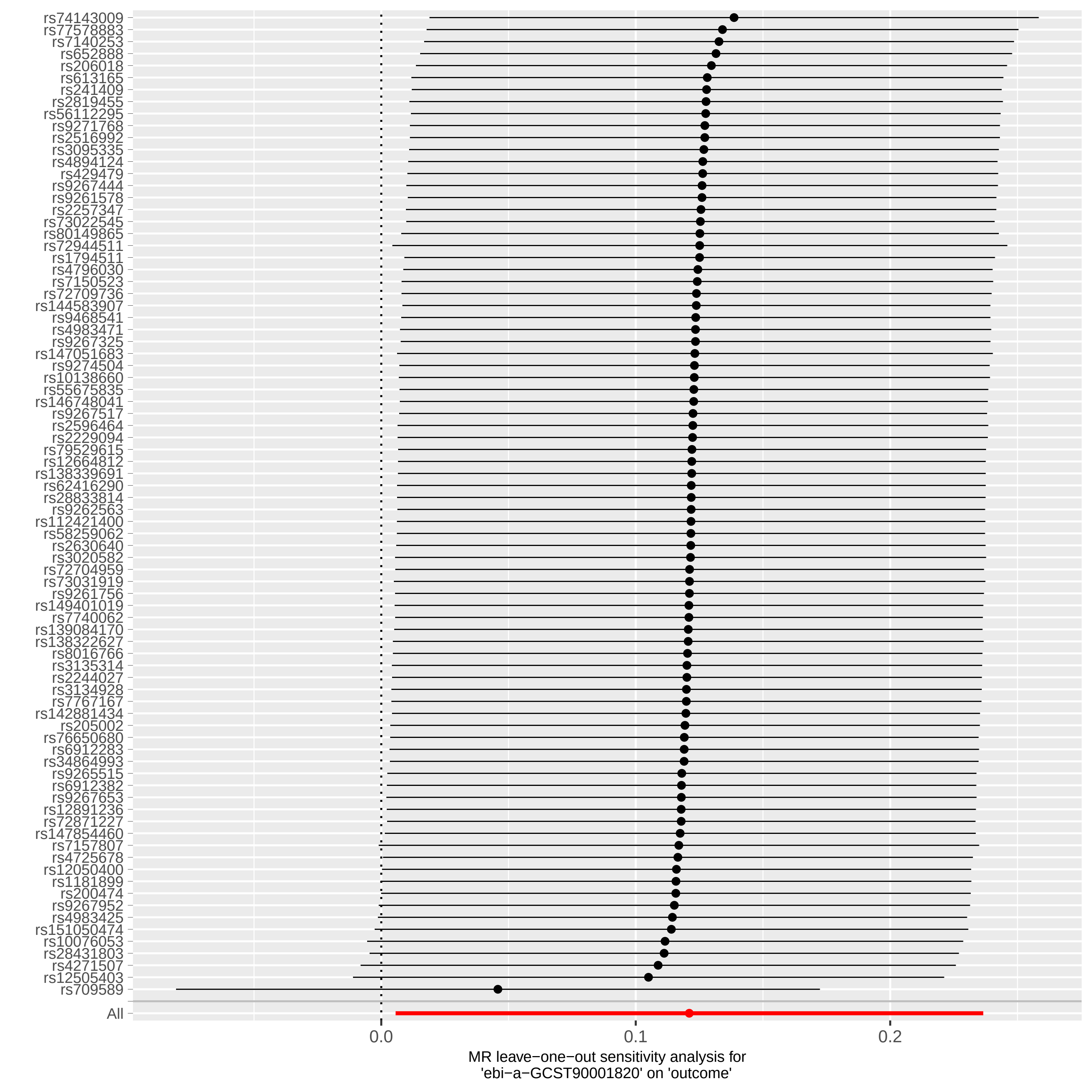
rs74143009
rs77578883
rs7140253
rs652888
rs206018
rs613165
rs241409
rs2819455
rs56112295
rs9271768
rs2516992
rs3095335
rs4894124
rs429479
rs9267444
rs9261578
rs2257347
rs73022545
rs80149865
rs72944511
rs1794511
rs4796030
rs7150523
rs72709736
rs144583907
rs9468541
rs4983471
rs9267325
rs147051683
rs9274504
rs10138660
rs55675835
rs146748041
rs9267517
rs2596464
rs2229094
rs79529615
rs12664812
rs138339691
rs62416290
rs28833814
rs9262563
rs112421400
rs58259062
rs2630640
rs3020582
rs72704959
rs73031919
rs9261756
rs149401019
rs7740062
rs139084170
rs138322627
rs8016766
rs3135314
rs2244027
rs3134928
rs7767167
rs142881434
rs205002
rs76650680
rs6912283
rs34864993
rs9265515
rs6912382
rs9267653
rs12891236
rs72871227
rs147854460
rs7157807
rs4725678
rs12050400
rs1181899
rs200474
rs9267952
rs4983425
rs151050474
rs10076053
rs28431803
rs4271507
rs12505403
rs709589
All
0.0
0.1
0.2
MR leave−one−out sensitivity analysis for
'ebi−a−GCST90001820' on 'outcome'

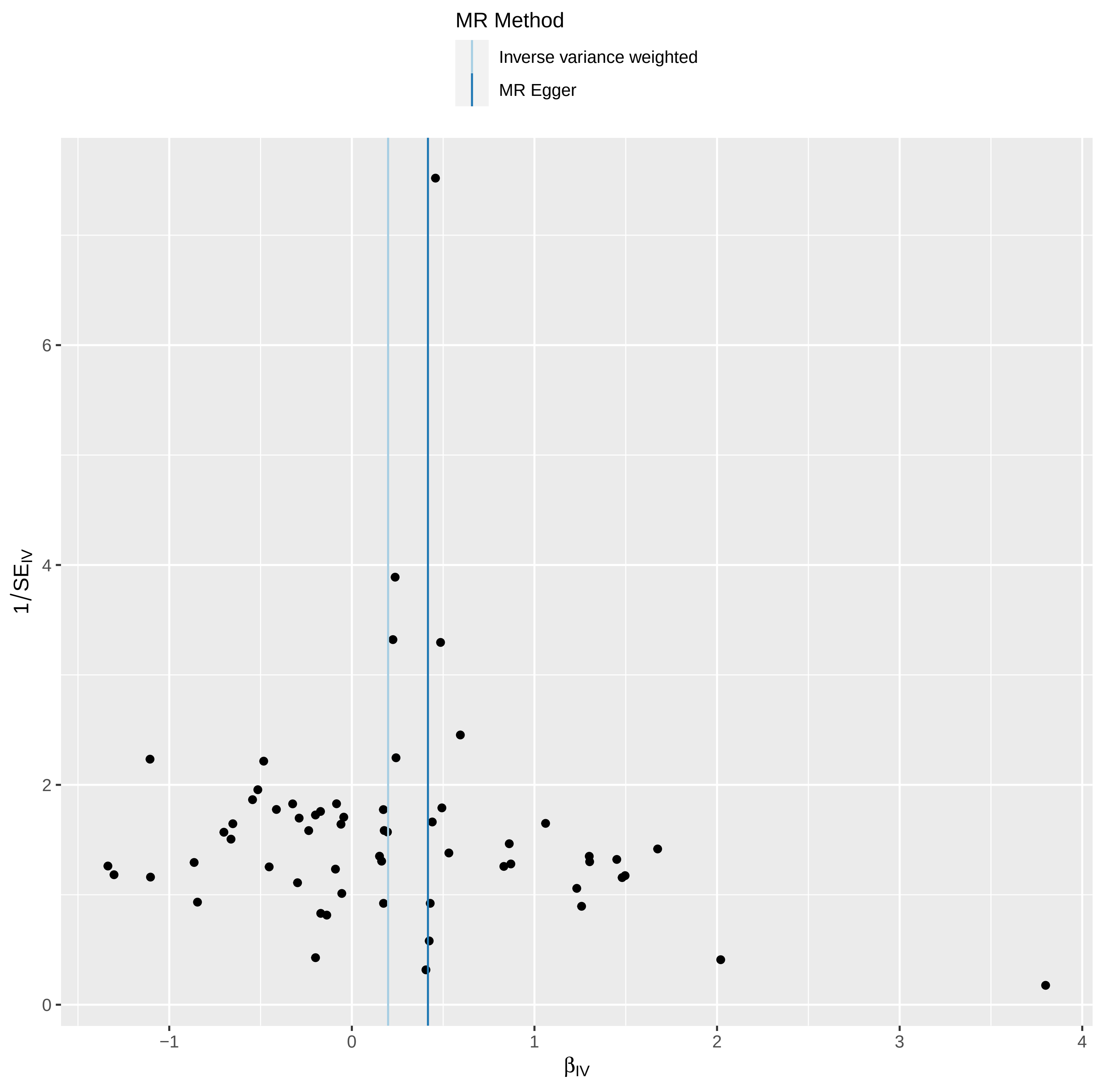
MR Method
Inverse variance weighted
MR Egger
1/SE_IV
β_IV
0
2
4
6
−1
0
1
2
3
4

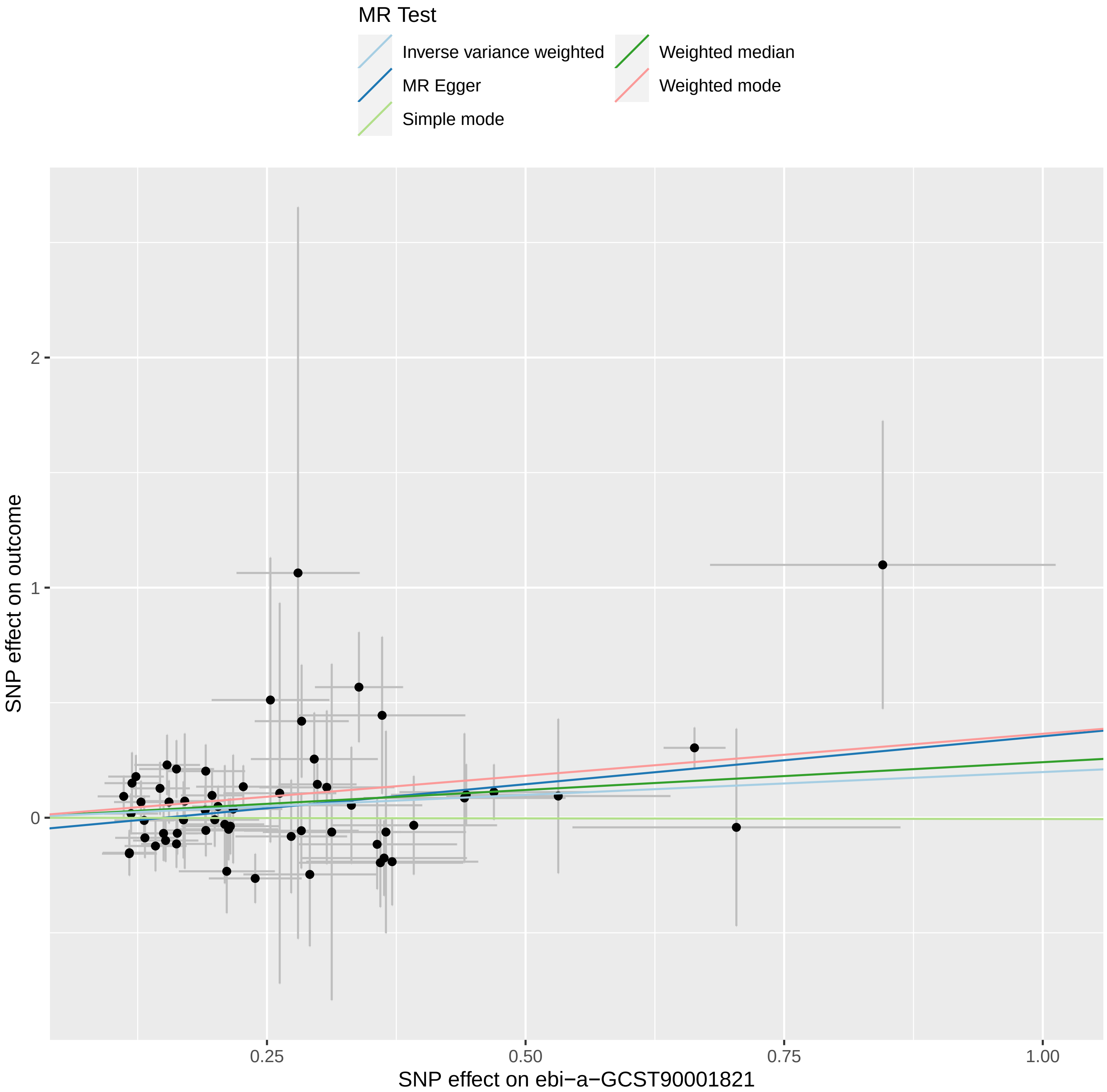
MR Test
Inverse variance weighted
MR Egger
Simple mode
Weighted median
Weighted mode
SNP effect on outcome
2
1
0
0.25
0.50
0.75
1.00
SNP effect on ebi−a−GCST90001821

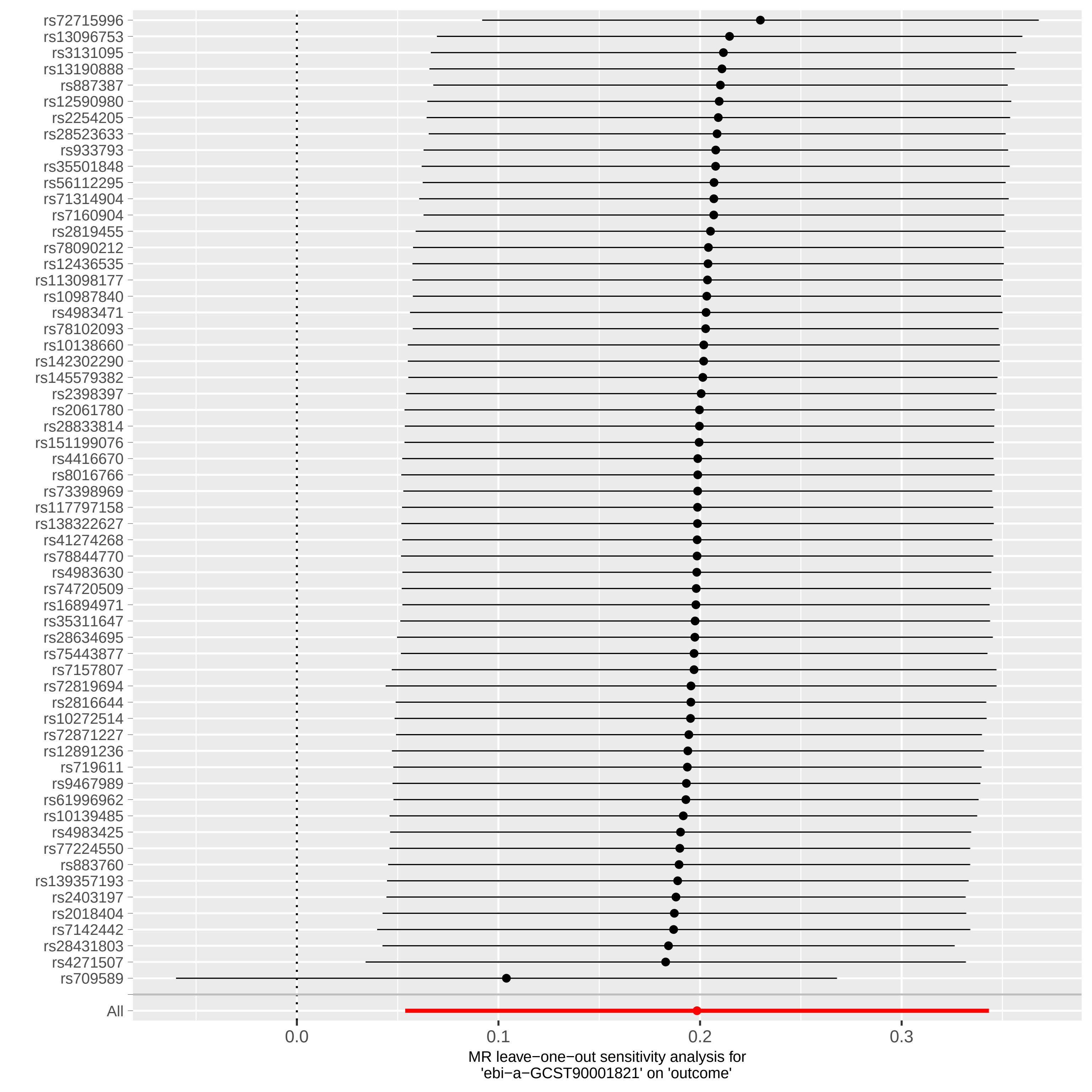
rs72715996
rs13096753
rs3131095
rs13190888
rs887387
rs12590980
rs2254205
rs28523633
rs933793
rs35501848
rs56112295
rs71314904
rs7160904
rs2819455
rs78090212
rs12436535
rs113098177
rs10987840
rs4983471
rs78102093
rs10138660
rs142302290
rs145579382
rs2398397
rs2061780
rs28833814
rs151199076
rs4416670
rs8016766
rs73398969
rs117797158
rs138322627
rs41274268
rs78844770
rs4983630
rs74720509
rs16894971
rs35311647
rs28634695
rs75443877
rs7157807
rs72819694
rs2816644
rs10272514
rs72871227
rs12891236
rs719611
rs9467989
rs61996962
rs10139485
rs4983425
rs77224550
rs883760
rs139357193
rs2403197
rs2018404
rs7142442
rs28431803
rs4271507
rs709589
All
0.0
0.1
0.2
0.3
MR leave−one−out sensitivity analysis for
'ebi−a−GCST90001821' on 'outcome'

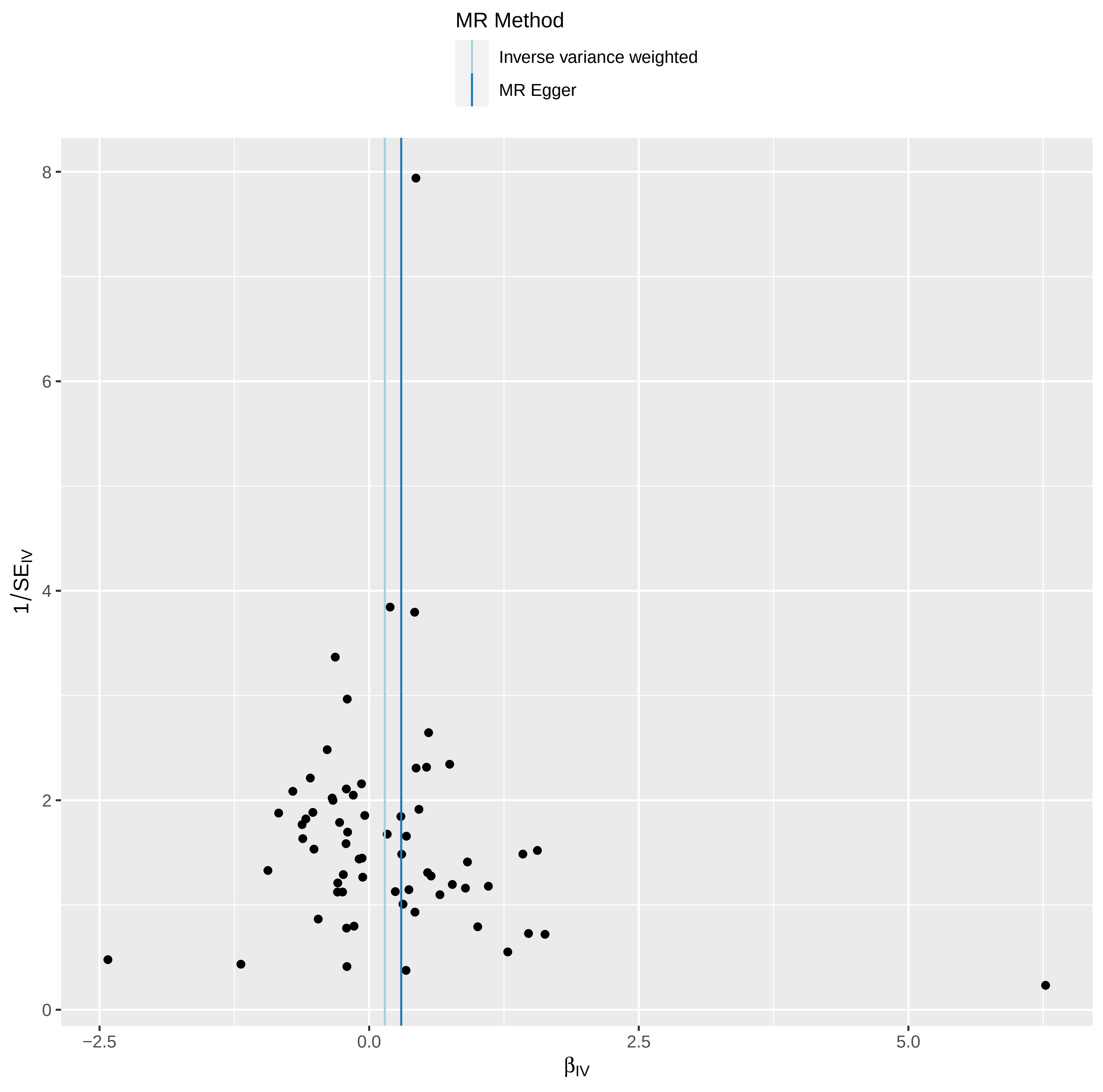

MR Method
Inverse variance weighted
MR Egger
8
6
4
2
0
1/SE_IV
−2.5
0.0
2.5
5.0
β_IV

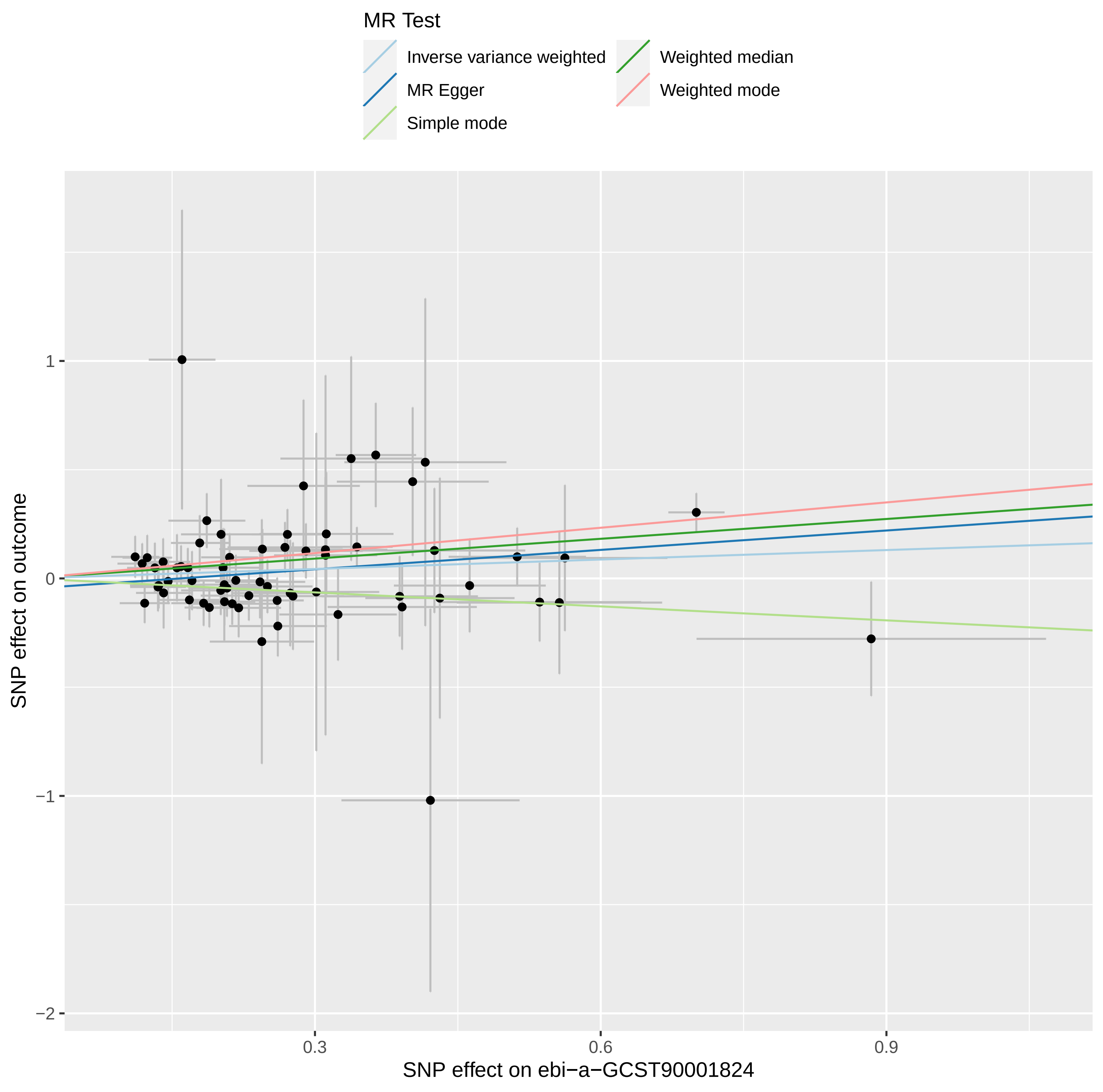

MR Test
Inverse variance weighted
MR Egger
Simple mode
Weighted median
Weighted mode
SNP effect on outcome
1
0
−1
−2
0.3
0.6
0.9
SNP effect on ebi−a−GCST90001824

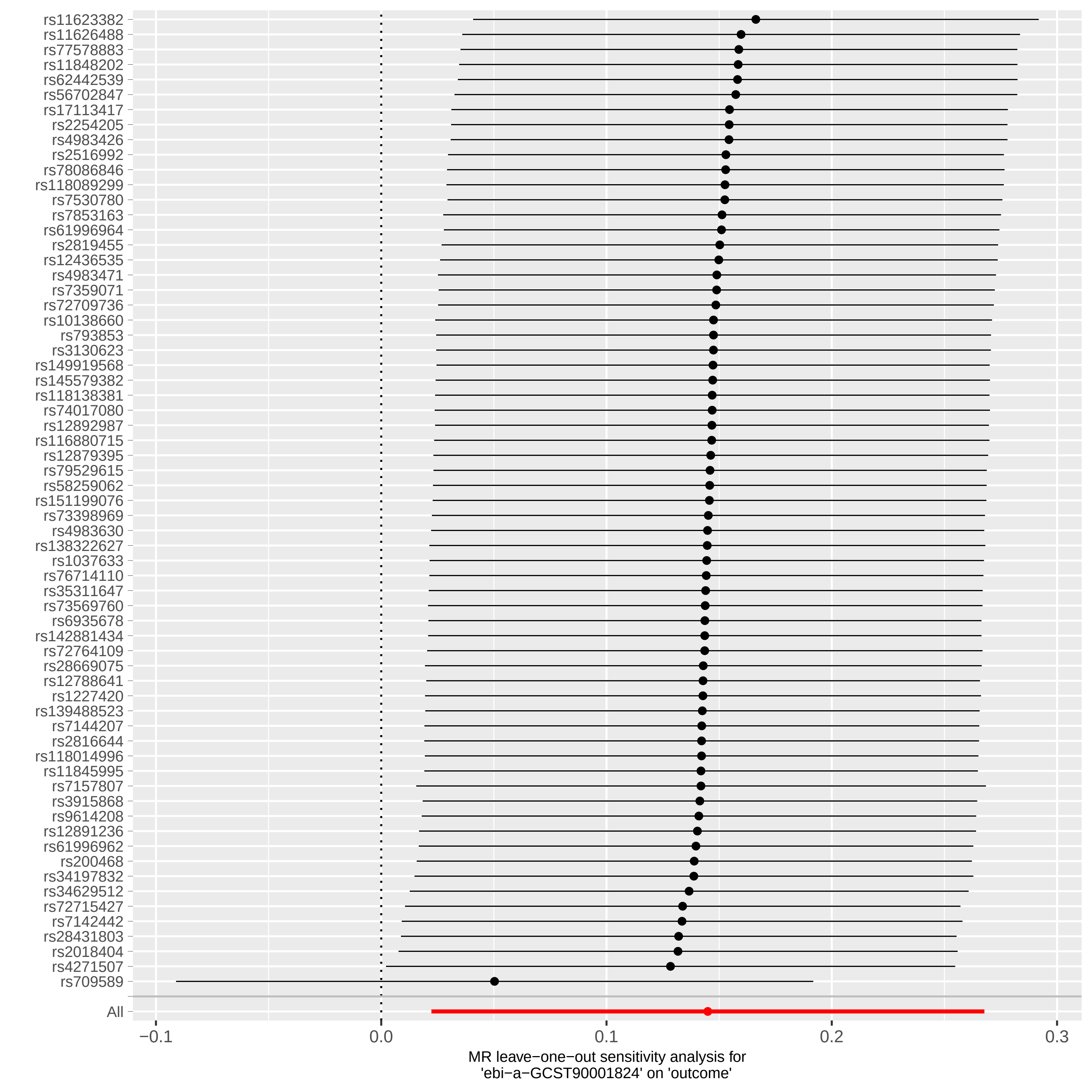
rs11623382
rs11626488
rs77578883
rs11848202
rs62442539
rs56702847
rs17113417
rs2254205
rs4983426
rs2516992
rs78086846
rs118089299
rs7530780
rs7853163
rs61996964
rs2819455
rs12436535
rs4983471
rs7359071
rs72709736
rs10138660
rs793853
rs3130623
rs149919568
rs145579382
rs118138381
rs74017080
rs12892987
rs116880715
rs12879395
rs79529615
rs58259062
rs151199076
rs73398969
rs4983630
rs138322627
rs1037633
rs76714110
rs35311647
rs73569760
rs6935678
rs142881434
rs72764109
rs28669075
rs12788641
rs1227420
rs139488523
rs7144207
rs2816644
rs118014996
rs11845995
rs7157807
rs3915868
rs9614208
rs12891236
rs61996962
rs200468
rs34197832
rs34629512
rs72715427
rs7142442
rs28431803
rs2018404
rs4271507
rs709589
All
−0.1
0.0
0.1
0.2
0.3
MR leave−one−out sensitivity analysis for
'ebi−a−GCST90001824' on 'outcome'

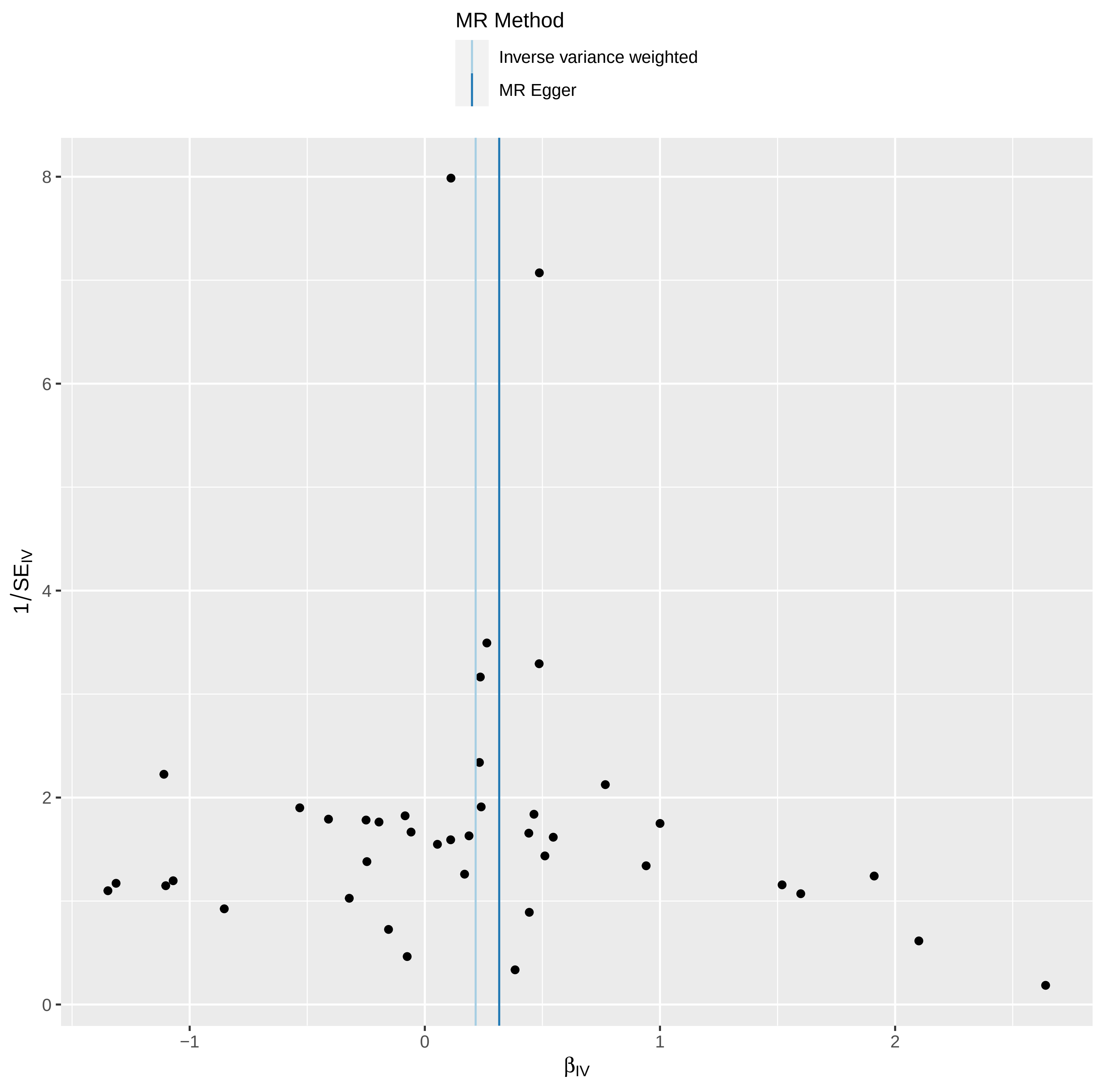
MR Method
Inverse variance weighted
MR Egger
8
6
4
2
0
−1
0
1
2
$1/SE_{IV}$
β_{IV}

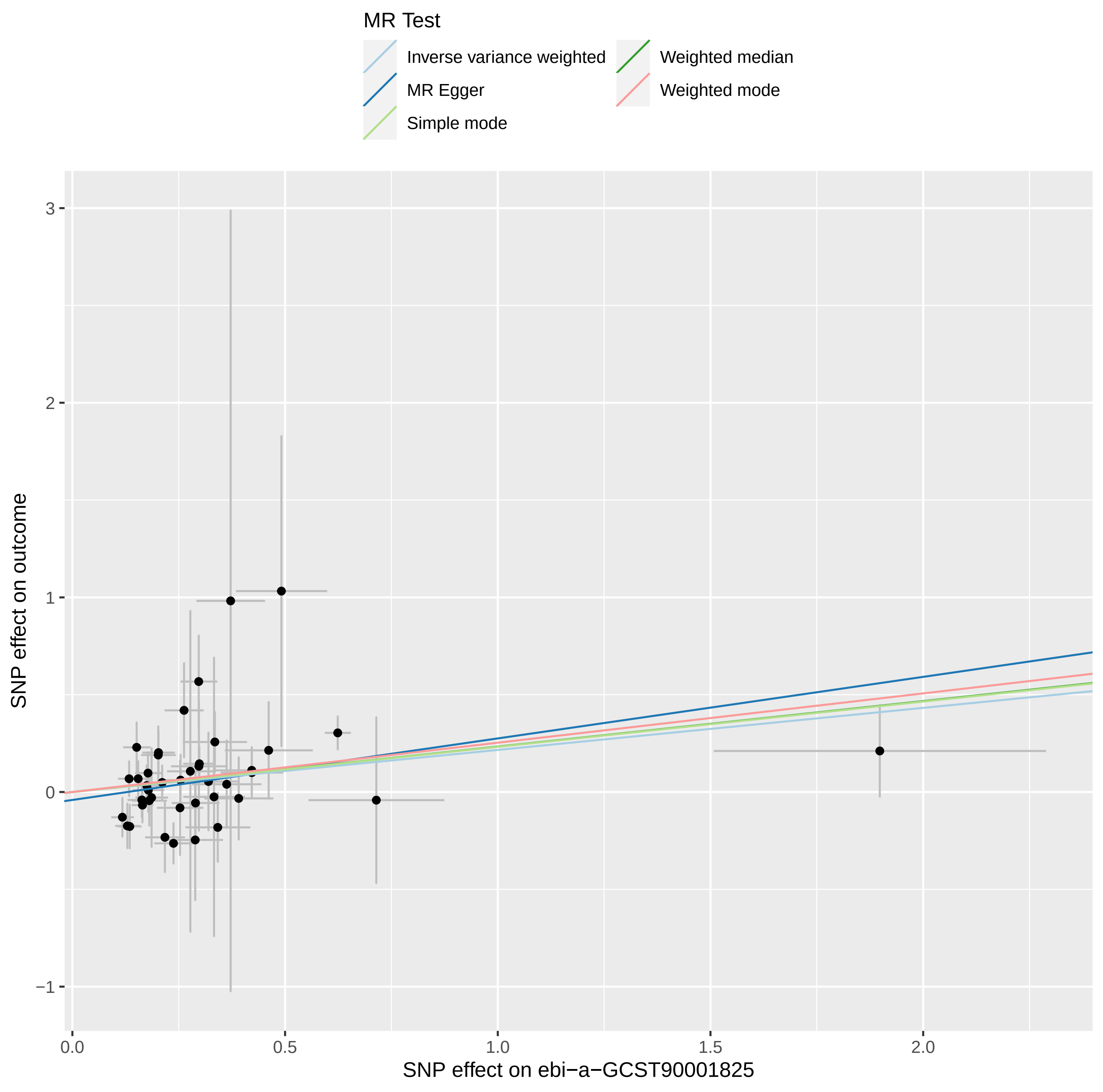

MR Test
Inverse variance weighted
MR Egger
Simple mode
Weighted median
Weighted mode
3
2
1
0
−1
0.0
0.5
1.0
1.5
2.0
SNP effect on outcome
SNP effect on ebi−a−GCST90001825

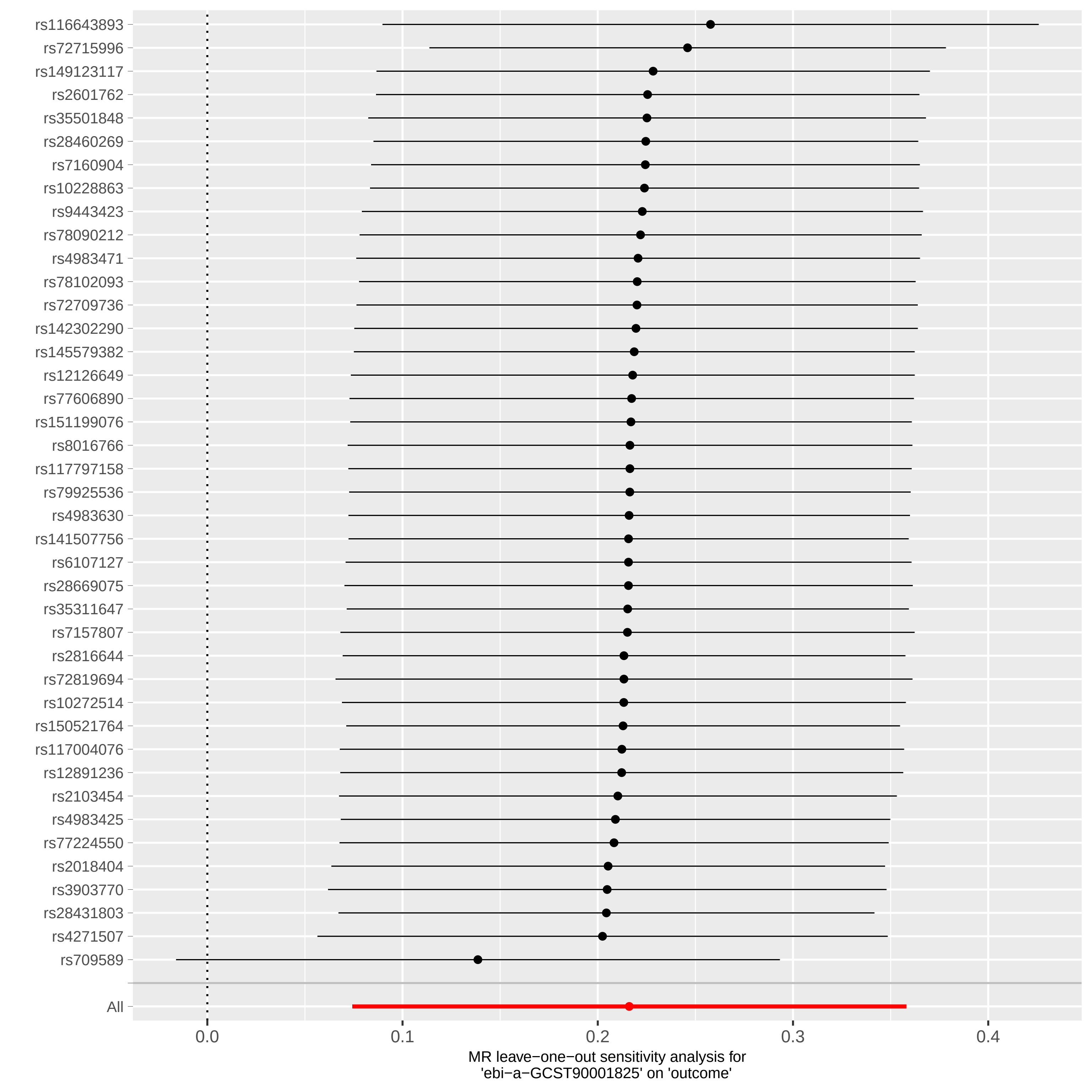
rs116643893
rs72715996
rs149123117
rs2601762
rs35501848
rs28460269
rs7160904
rs10228863
rs9443423
rs78090212
rs4983471
rs78102093
rs72709736
rs142302290
rs145579382
rs12126649
rs77606890
rs151199076
rs8016766
rs117797158
rs79925536
rs4983630
rs141507756
rs6107127
rs28669075
rs35311647
rs7157807
rs2816644
rs72819694
rs10272514
rs150521764
rs117004076
rs12891236
rs2103454
rs4983425
rs77224550
rs2018404
rs3903770
rs28431803
rs4271507
rs709589
All
0.0
0.1
0.2
0.3
0.4
MR leave−one−out sensitivity analysis for
'ebi−a−GCST90001825' on 'outcome'

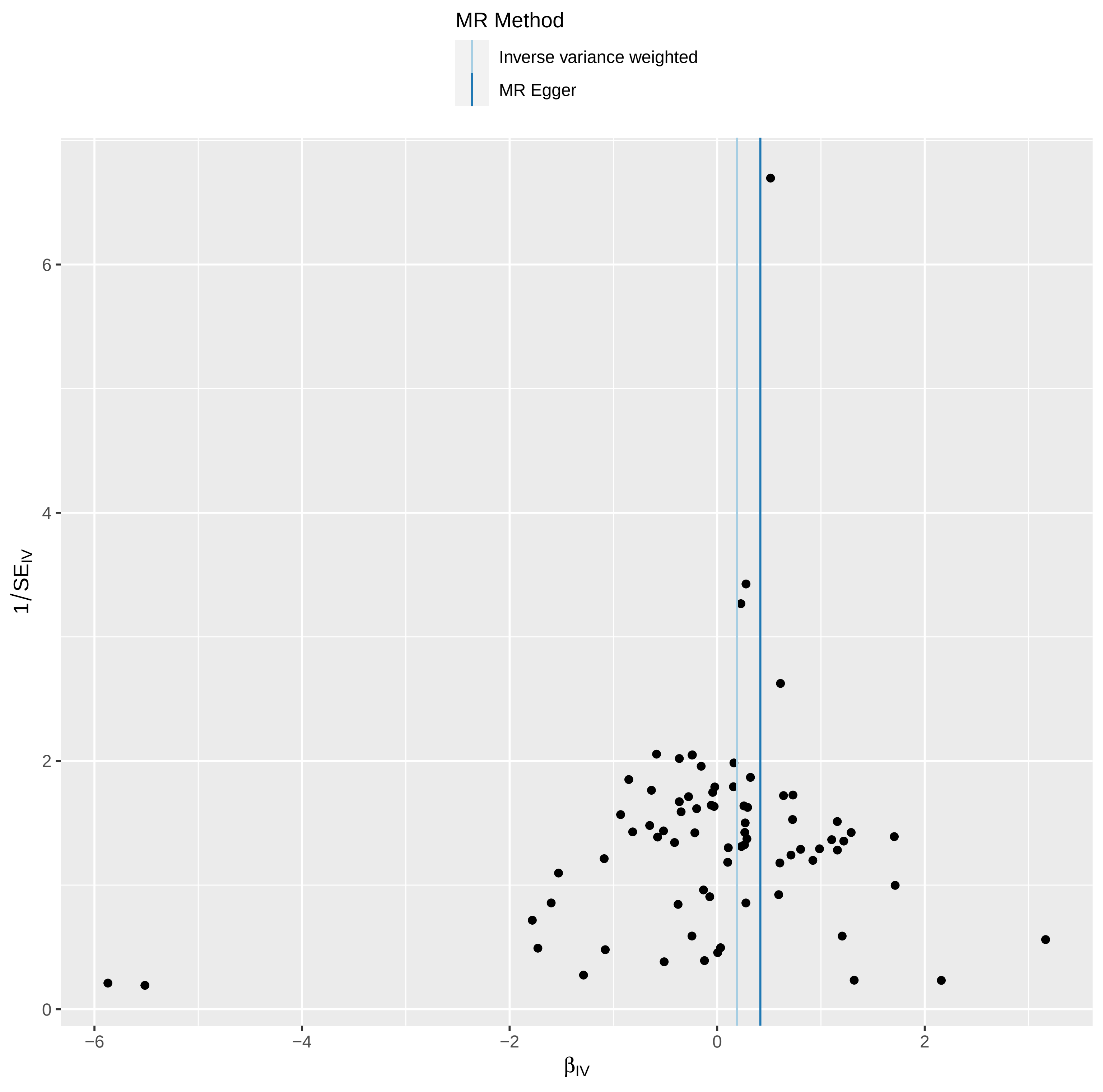
MR Method
Inverse variance weighted
MR Egger
6
4
2
0
1/SE_IV
−6
−4
−2
0
2
β_IV

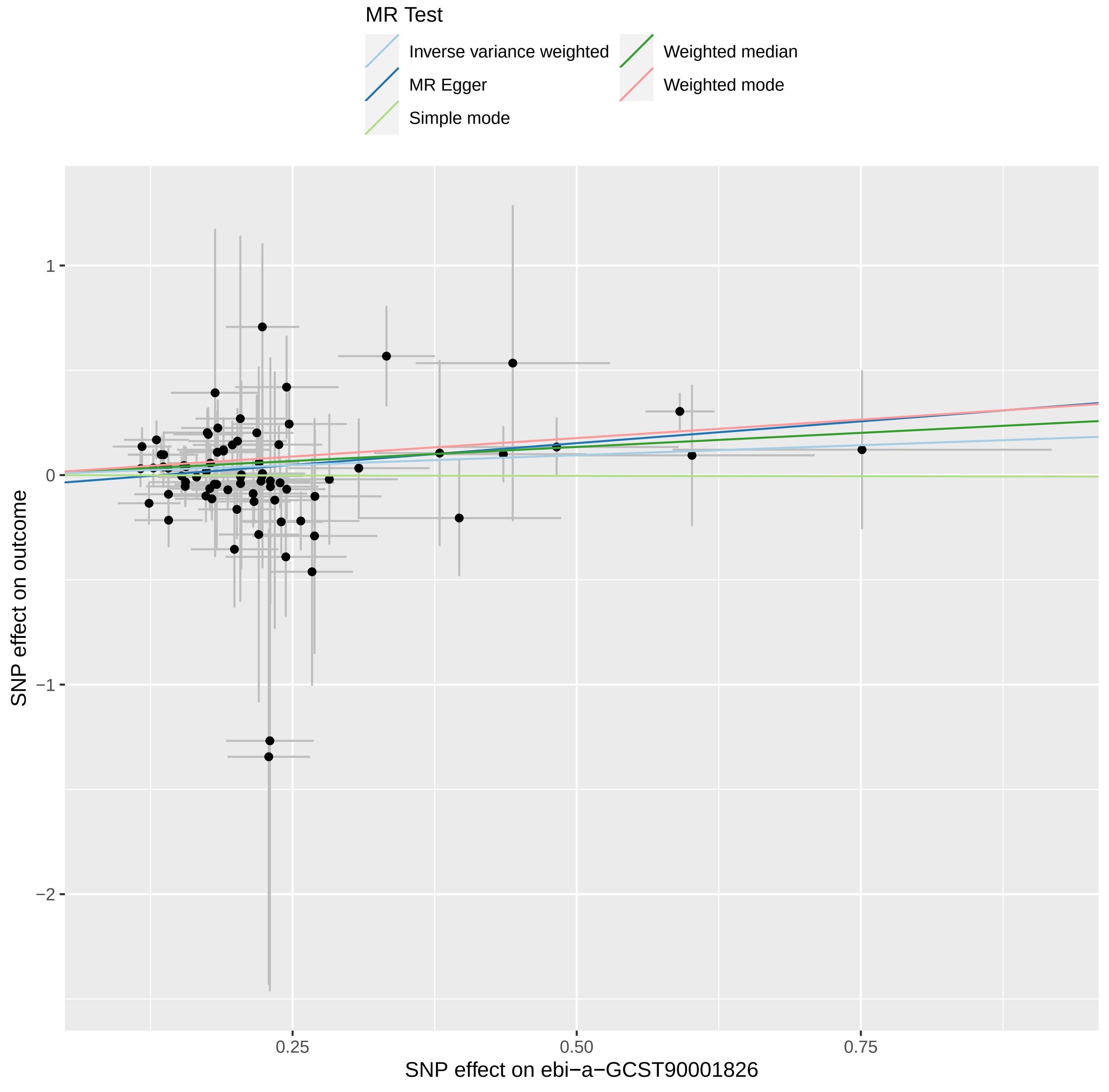
MR Test
Inverse variance weighted
MR Egger
Simple mode
Weighted median
Weighted mode
SNP effect on outcome
1
0
−1
−2
0.25
0.50
0.75
SNP effect on ebi−a−GCST90001826

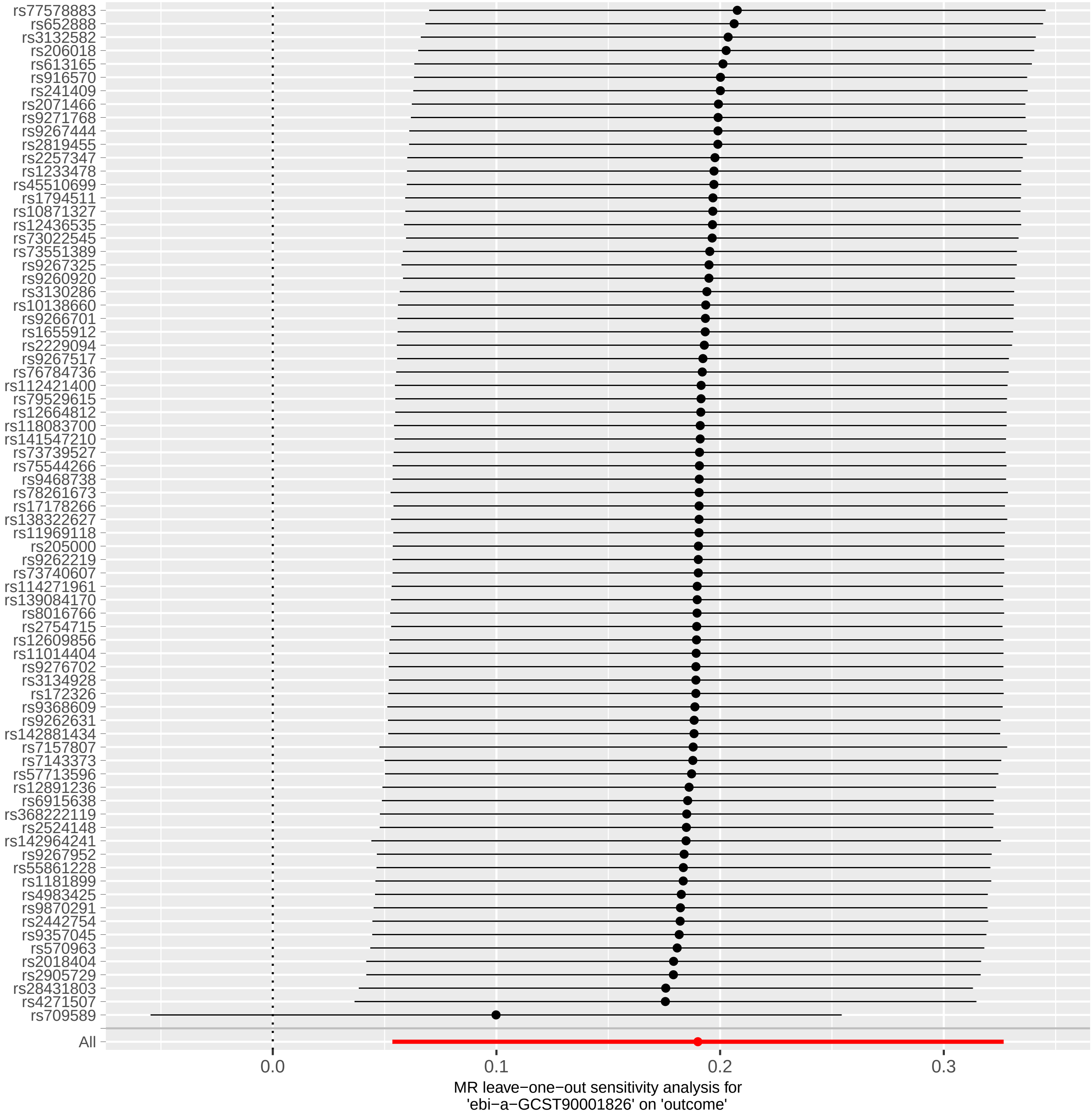

rs77578883
rs652888
rs3132582
rs206018
rs613165
rs916570
rs241409
rs2071466
rs9271768
rs9267444
rs2819455
rs2257347
rs1233478
rs45510699
rs1794511
rs10871327
rs12436535
rs73022545
rs73551389
rs9267325
rs9260920
rs3130286
rs10138660
rs9266701
rs1655912
rs2229094
rs9267517
rs76784736
rs112421400
rs79529615
rs12664812
rs118083700
rs141547210
rs73739527
rs75544266
rs9468738
rs78261673
rs17178266
rs138322627
rs11969118
rs205000
rs9262219
rs73740607
rs114271961
rs139084170
rs8016766
rs2754715
rs12609856
rs11014404
rs9276702
rs3134928
rs172326
rs9368609
rs9262631
rs142881434
rs7157807
rs7143373
rs57713596
rs12891236
rs6915638
rs368222119
rs2524148
rs142964241
rs9267952
rs55861228
rs1181899
rs4983425
rs9870291
rs2442754
rs9357045
rs570963
rs2018404
rs2905729
rs28431803
rs4271507
rs709589
All
0.0
0.1
0.2
0.3
MR leave−one−out sensitivity analysis for
'ebi−a−GCST90001826' on 'outcome'

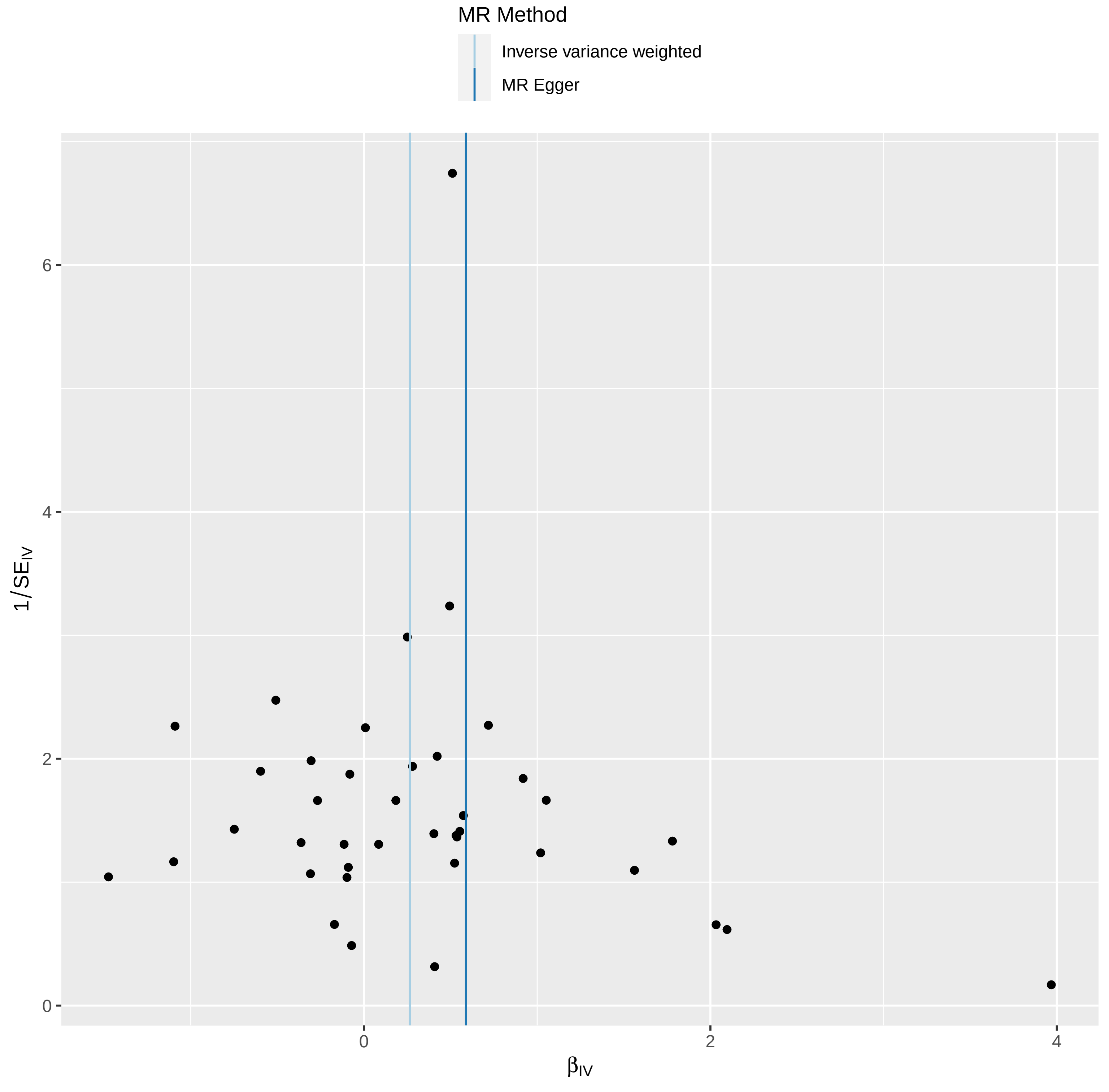
MR Method
Inverse variance weighted
MR Egger
1/SE_IV
β_IV
0
2
4
6
0
2
4

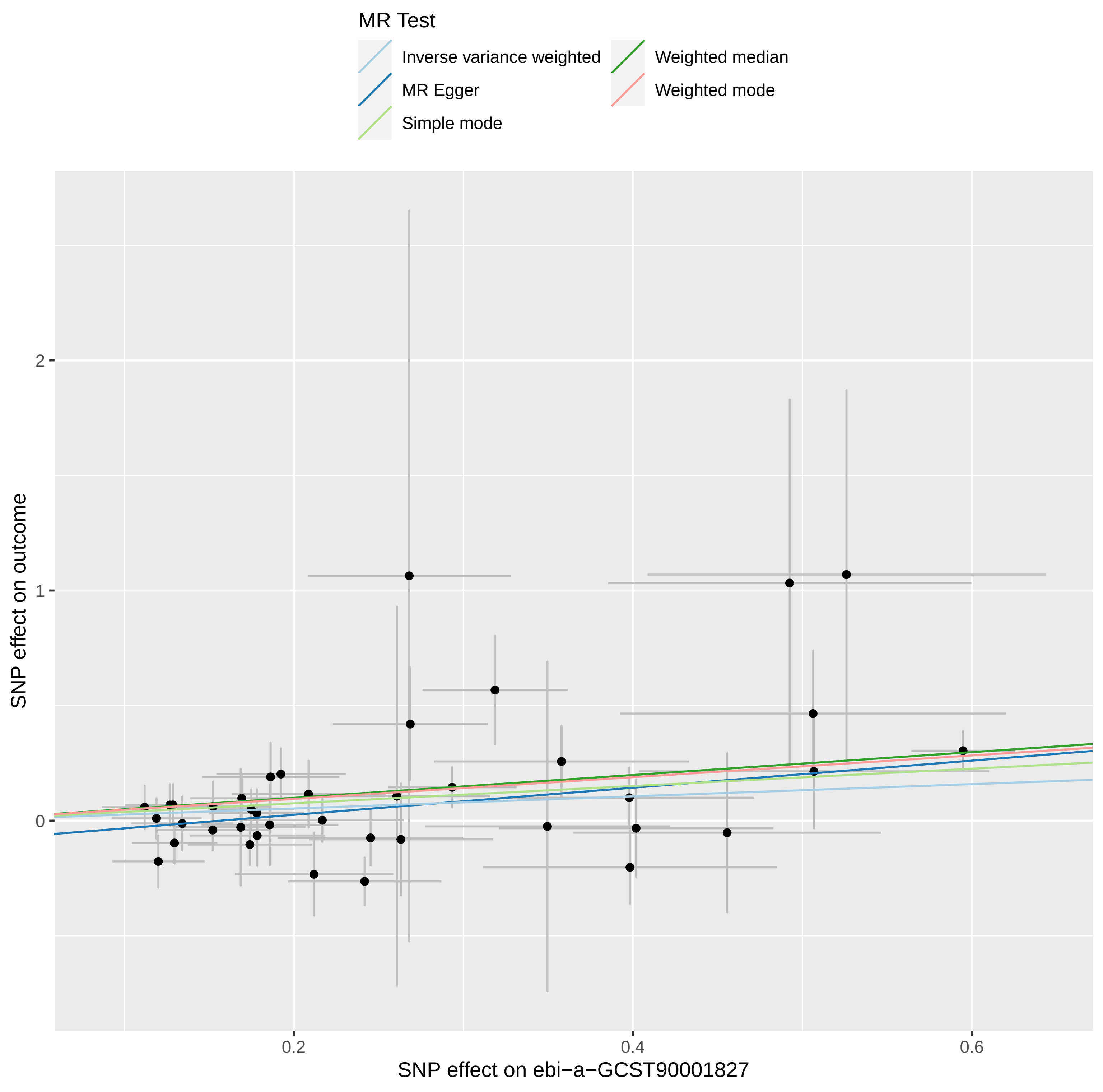

MR Test
Inverse variance weighted
MR Egger
Simple mode
Weighted median
Weighted mode
SNP effect on outcome
2
1
0
0.2
0.4
0.6
SNP effect on ebi−a−GCST90001827

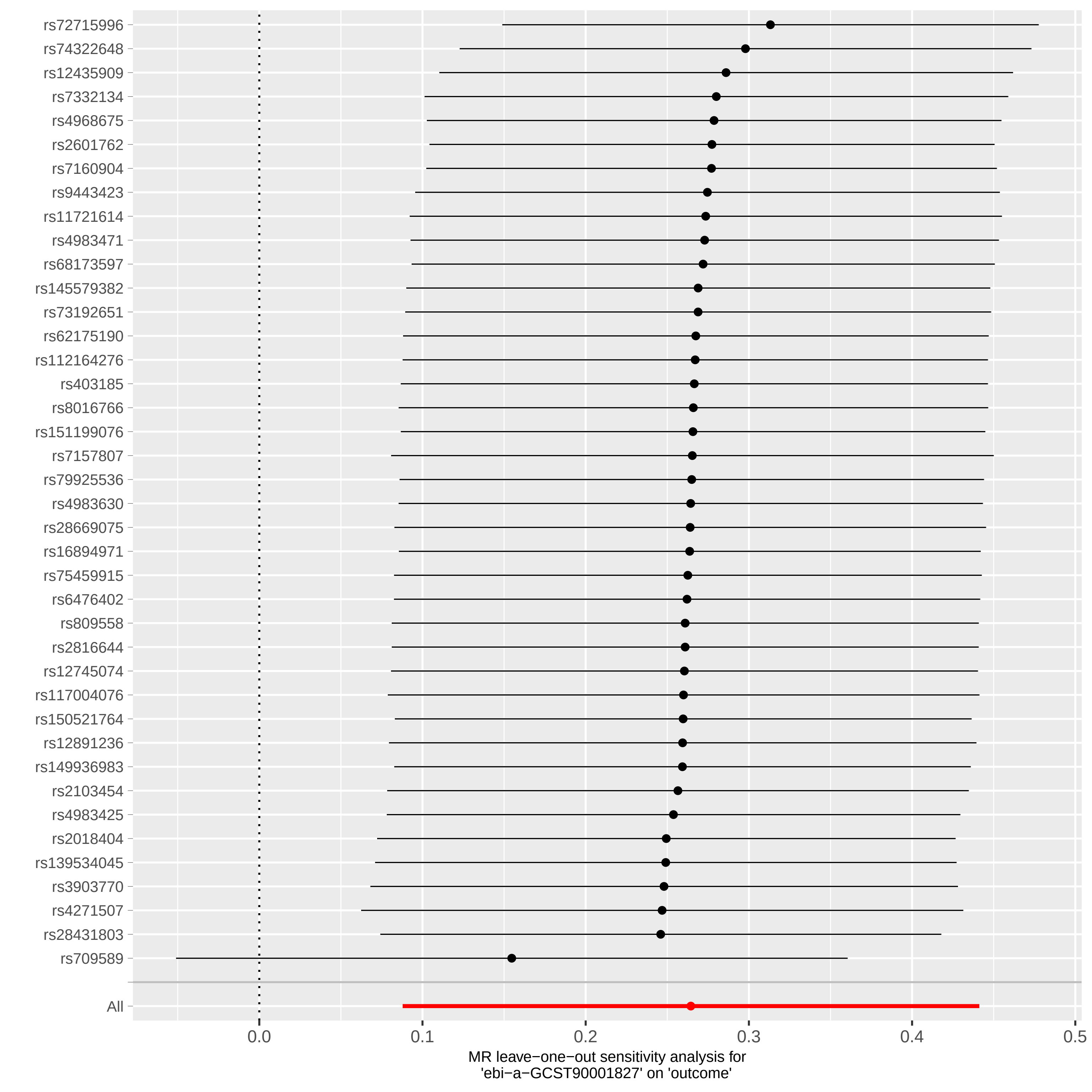
rs72715996
rs74322648
rs12435909
rs7332134
rs4968675
rs2601762
rs7160904
rs9443423
rs11721614
rs4983471
rs68173597
rs145579382
rs73192651
rs62175190
rs112164276
rs403185
rs8016766
rs151199076
rs7157807
rs79925536
rs4983630
rs28669075
rs16894971
rs75459915
rs6476402
rs809558
rs2816644
rs12745074
rs117004076
rs150521764
rs12891236
rs149936983
rs2103454
rs4983425
rs2018404
rs139534045
rs3903770
rs4271507
rs28431803
rs709589
All
0.0
0.1
0.2
0.3
0.4
0.5
MR leave−one−out sensitivity analysis for
'ebi−a−GCST90001827' on 'outcome'

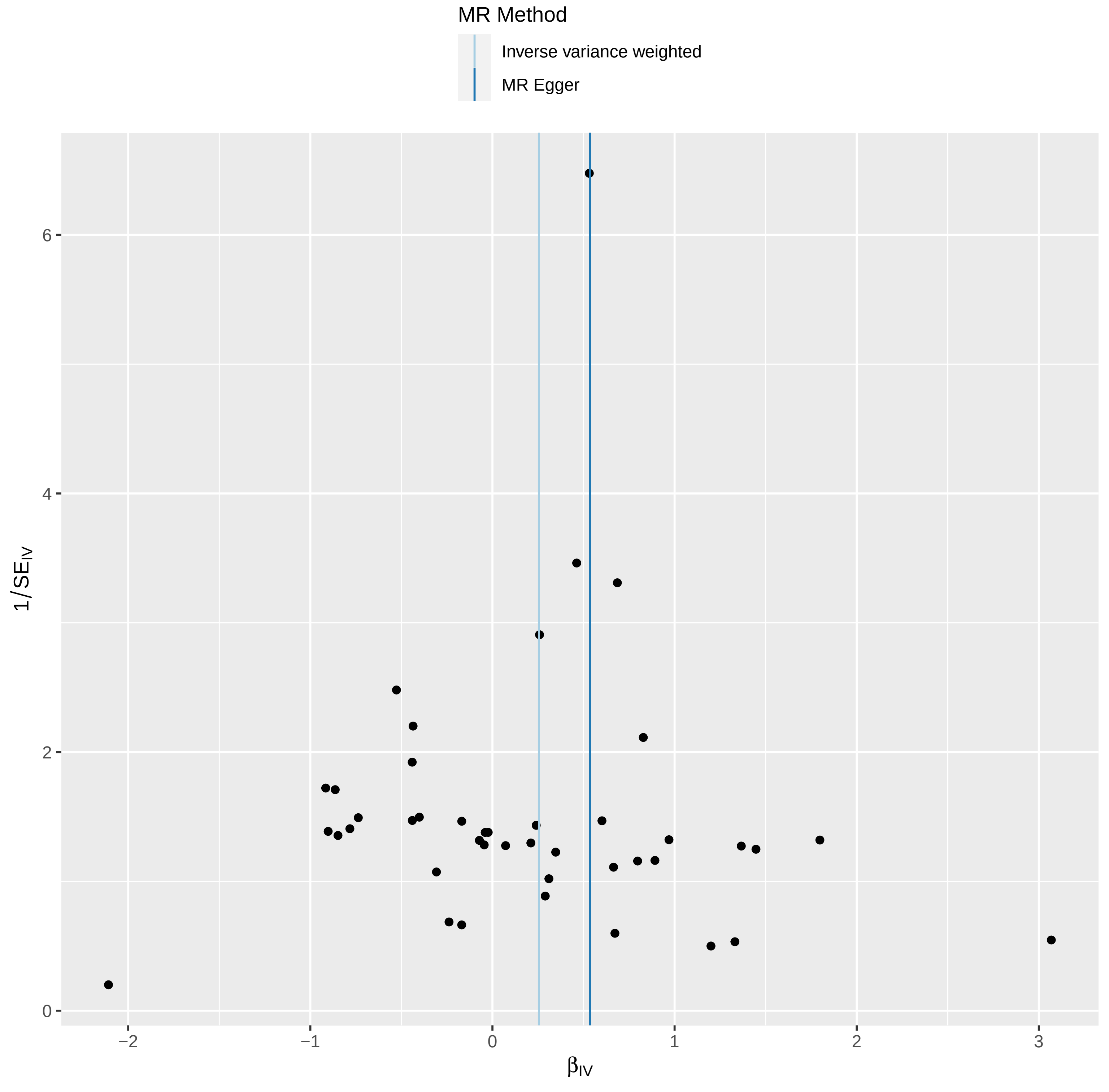
MR Method
Inverse variance weighted
MR Egger
1/SE_IV
β_IV
6
4
2
0
−2
−1
0
1
2
3

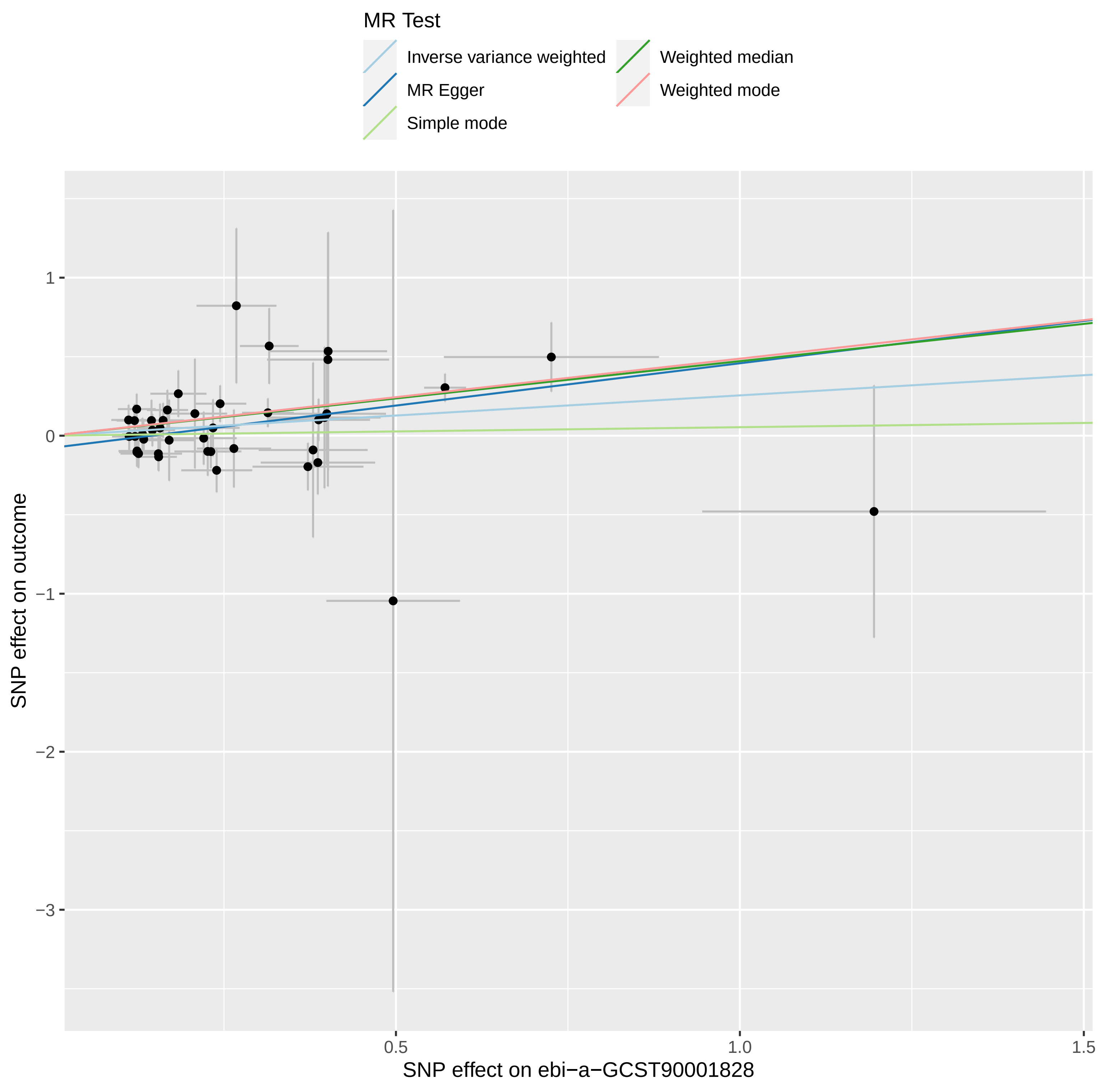
MR Test
Inverse variance weighted
MR Egger
Simple mode
Weighted median
Weighted mode
1
0
−1
−2
−3
0.5
1.0
1.5
SNP effect on outcome
SNP effect on ebi−a−GCST90001828

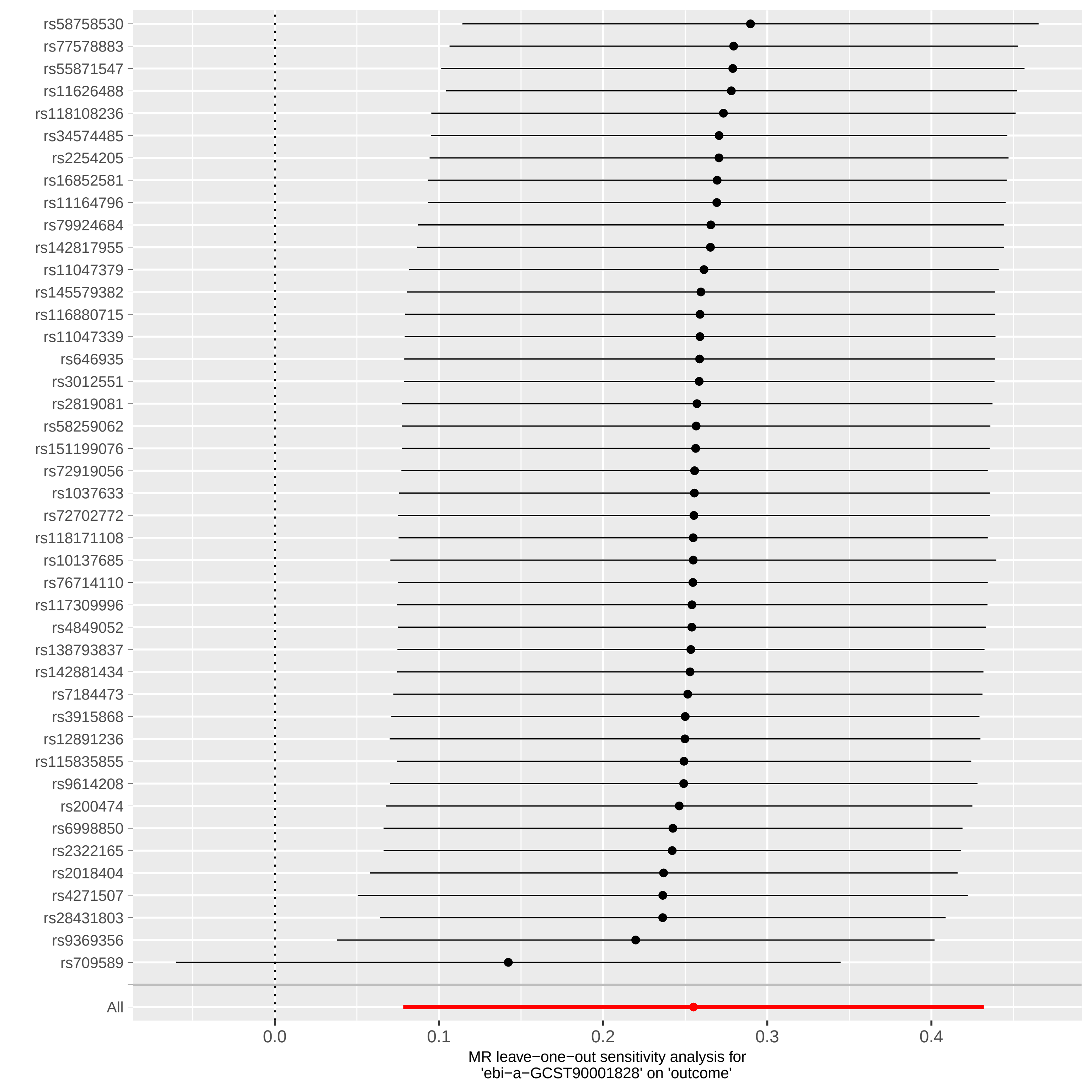
rs58758530
rs77578883
rs55871547
rs11626488
rs118108236
rs34574485
rs2254205
rs16852581
rs11164796
rs79924684
rs142817955
rs11047379
rs145579382
rs116880715
rs11047339
rs646935
rs3012551
rs2819081
rs58259062
rs151199076
rs72919056
rs1037633
rs72702772
rs118171108
rs10137685
rs76714110
rs117309996
rs4849052
rs138793837
rs142881434
rs7184473
rs3915868
rs12891236
rs115835855
rs9614208
rs200474
rs6998850
rs2322165
rs2018404
rs4271507
rs28431803
rs9369356
rs709589
All
0.0
0.1
0.2
0.3
0.4
MR leave−one−out sensitivity analysis for
'ebi−a−GCST90001828' on 'outcome'

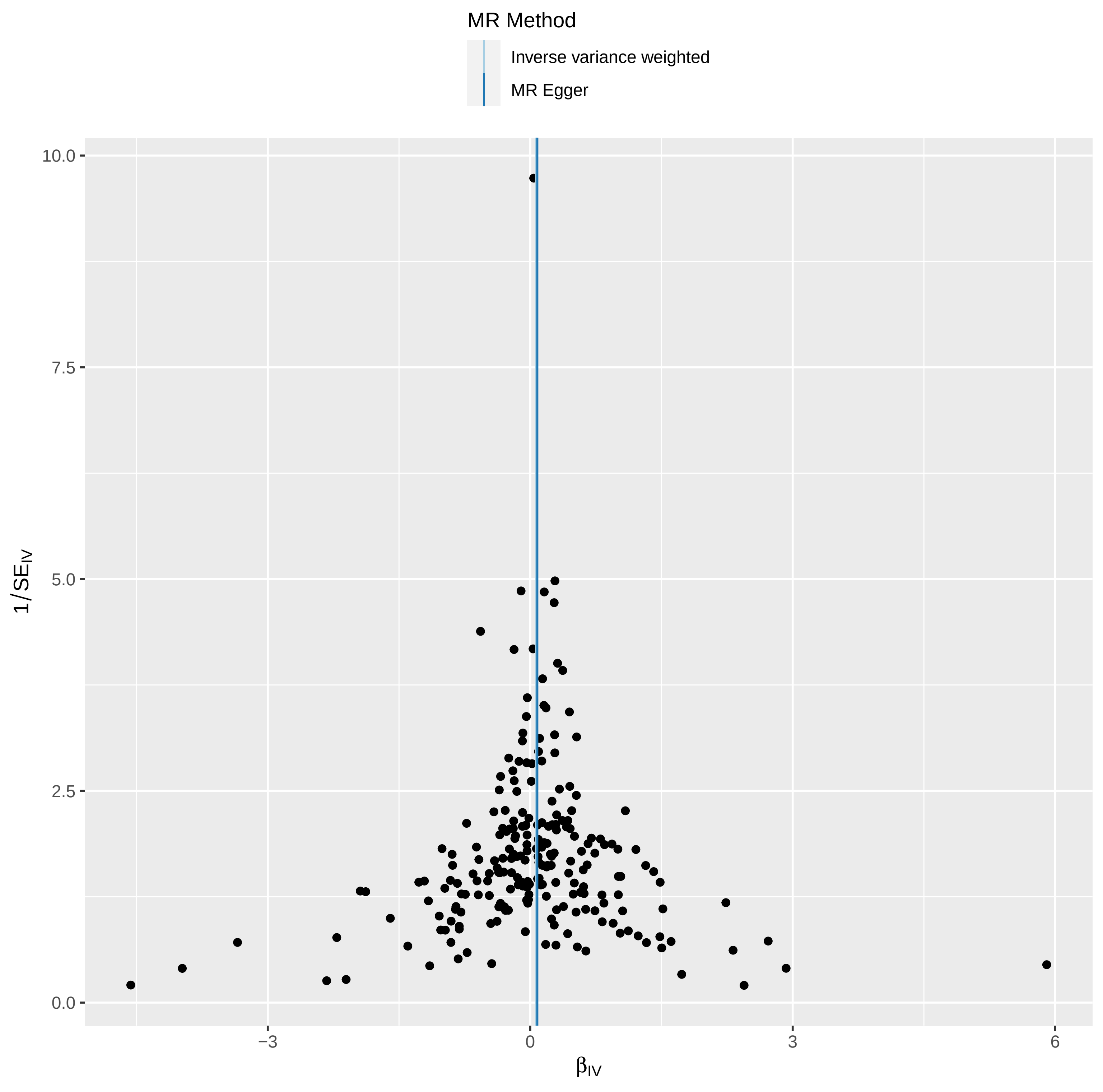

MR Method
Inverse variance weighted
MR Egger
10.0
7.5
5.0
2.5
0.0
$1/SE_{IV}$
−3
0
3
6
β_{IV}

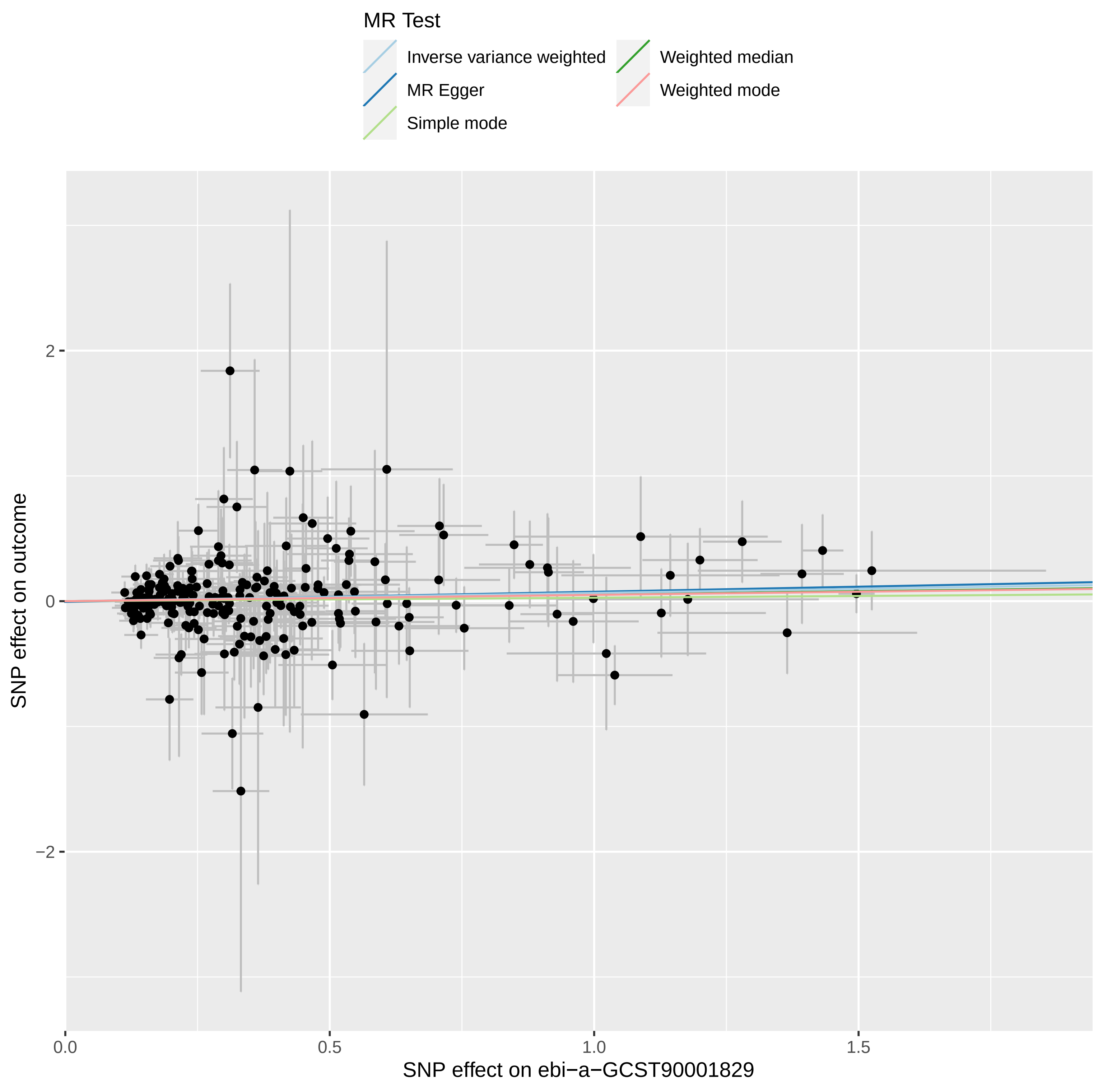
MR Test
Inverse variance weighted
MR Egger
Simple mode
Weighted median
Weighted mode
2
0
−2
0.0
0.5
1.0
1.5
SNP effect on outcome
SNP effect on ebi−a−GCST90001829

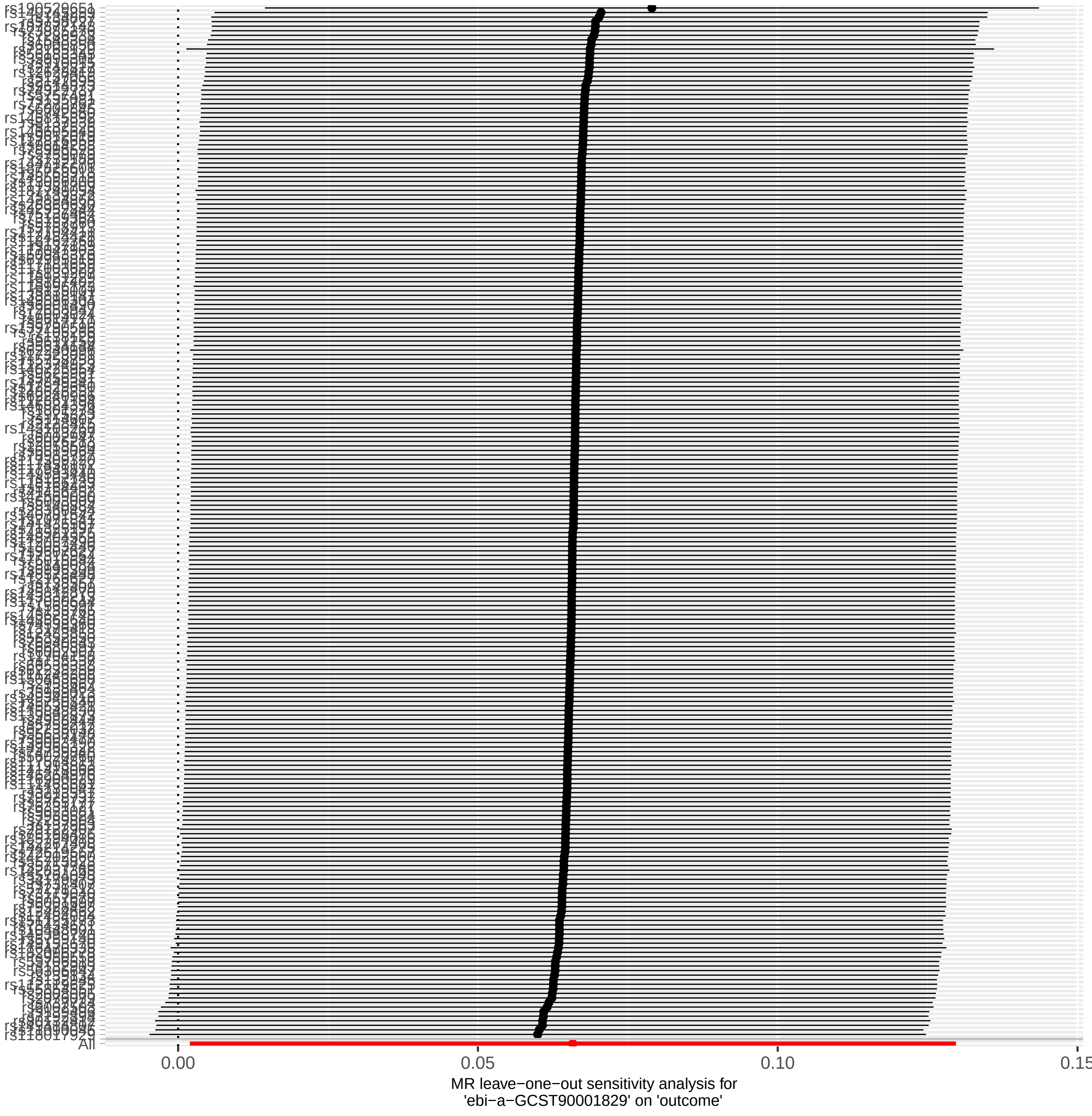
rs190529651
All
0.00
0.05
0.10
0.15
MR leave−one−out sensitivity analysis for
'ebi−a−GCST90001829' on 'outcome'

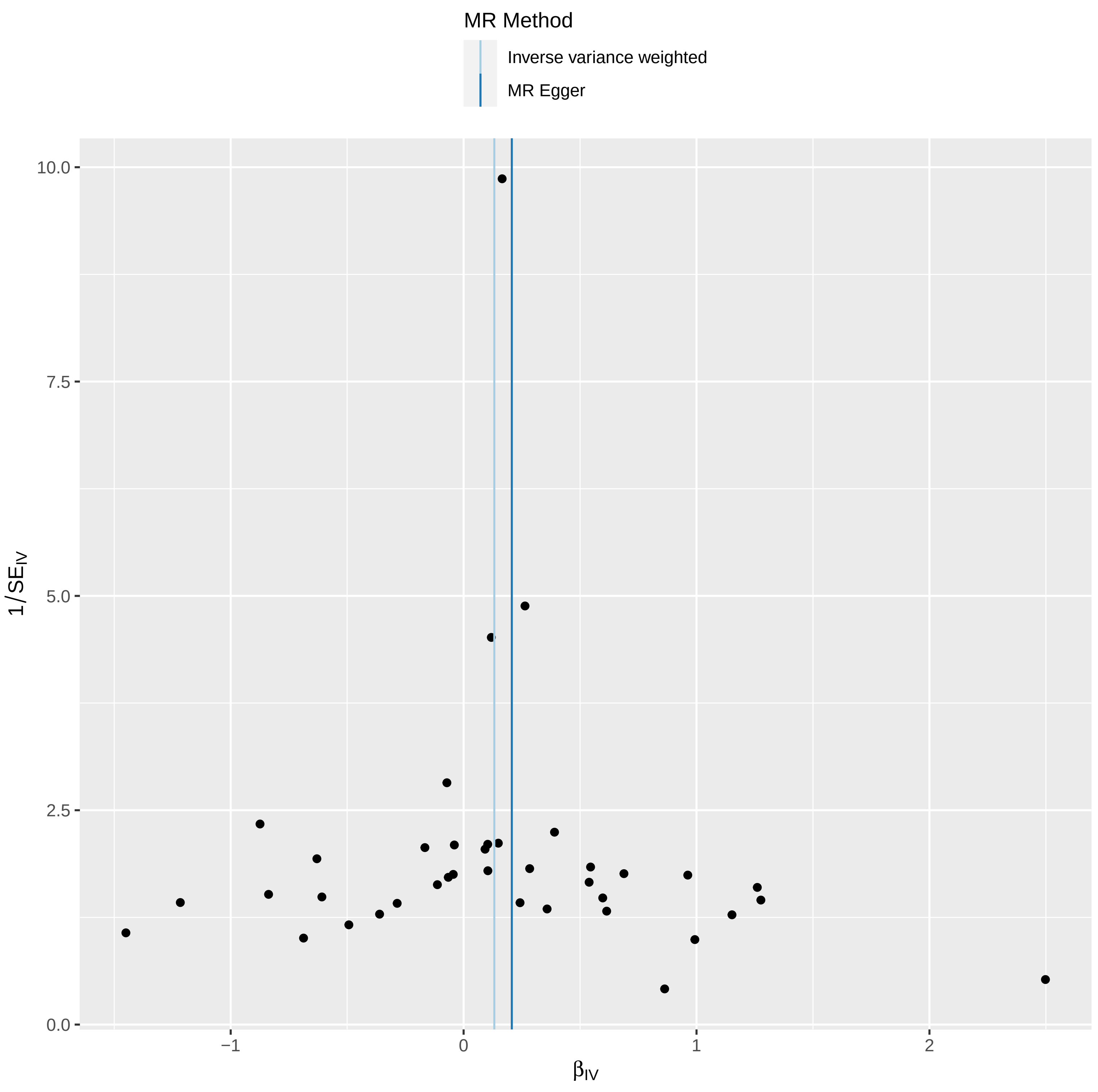
MR Method
Inverse variance weighted
MR Egger
10.0
7.5
5.0
2.5
0.0
−1
0
1
2
$1/SE_{IV}$
β_{IV}

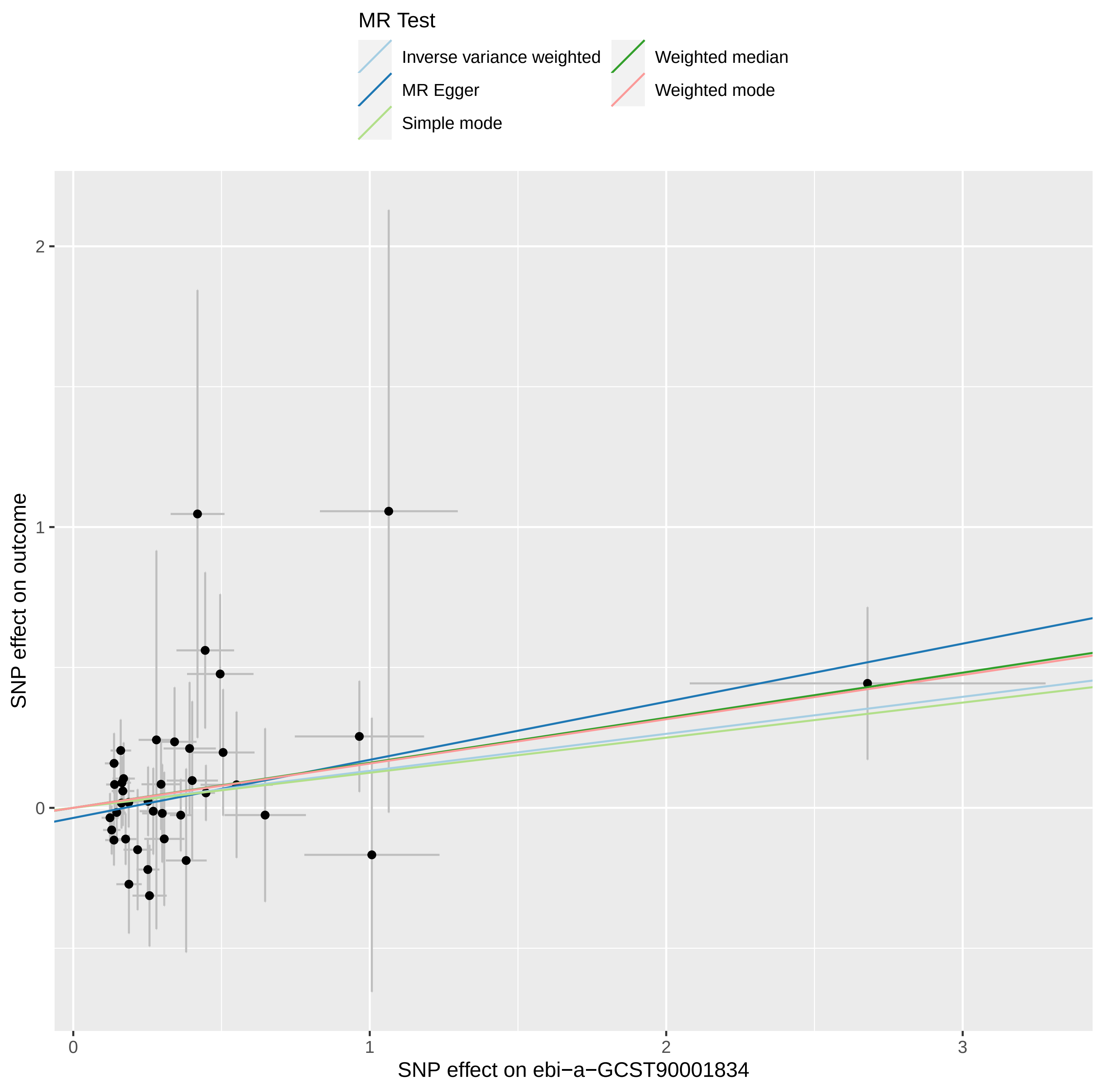
MR Test
Inverse variance weighted
MR Egger
Simple mode
Weighted median
Weighted mode
SNP effect on outcome
SNP effect on ebi−a−GCST90001834
0
1
2
3
0
1
2

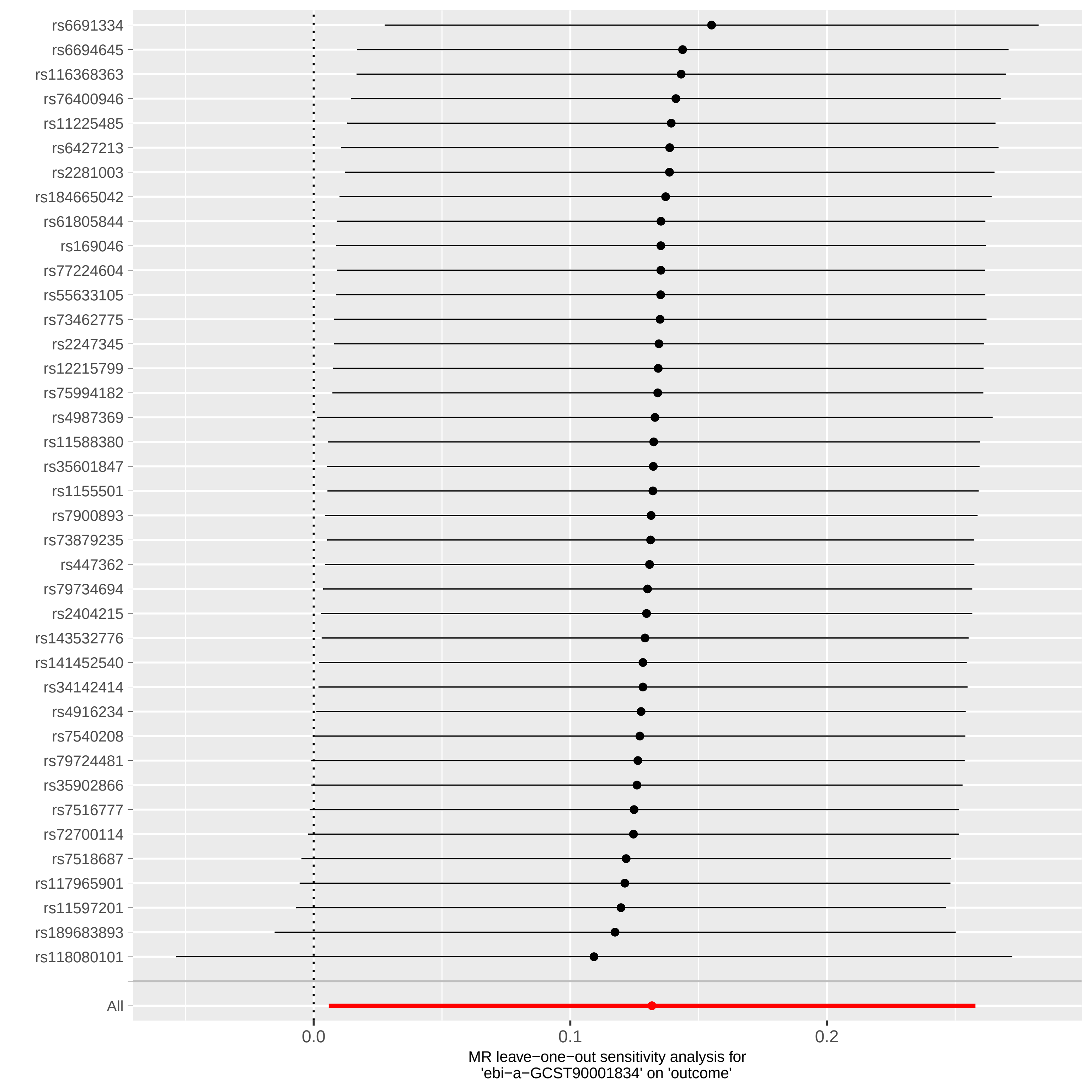

rs6691334
rs6694645
rs116368363
rs76400946
rs11225485
rs6427213
rs2281003
rs184665042
rs61805844
rs169046
rs77224604
rs55633105
rs73462775
rs2247345
rs12215799
rs75994182
rs4987369
rs11588380
rs35601847
rs1155501
rs7900893
rs73879235
rs447362
rs79734694
rs2404215
rs143532776
rs141452540
rs34142414
rs4916234
rs7540208
rs79724481
rs35902866
rs7516777
rs72700114
rs7518687
rs117965901
rs11597201
rs189683893
rs118080101
All
0.0
0.1
0.2
MR leave−one−out sensitivity analysis for
'ebi−a−GCST90001834' on 'outcome'

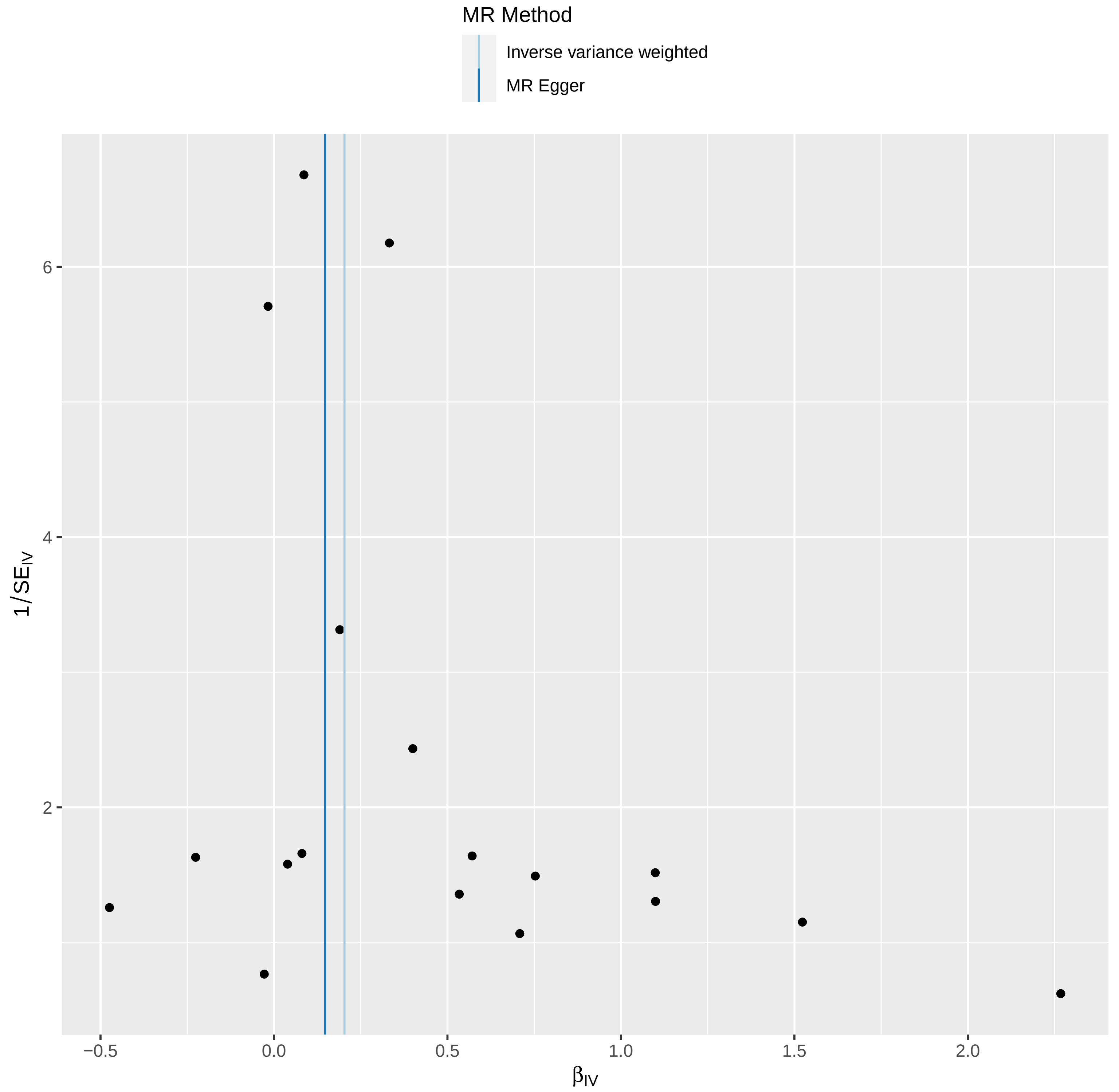
MR Method
Inverse variance weighted
MR Egger
1/SE_{IV}
6
4
2
−0.5
0.0
0.5
1.0
1.5
2.0
β_{IV}

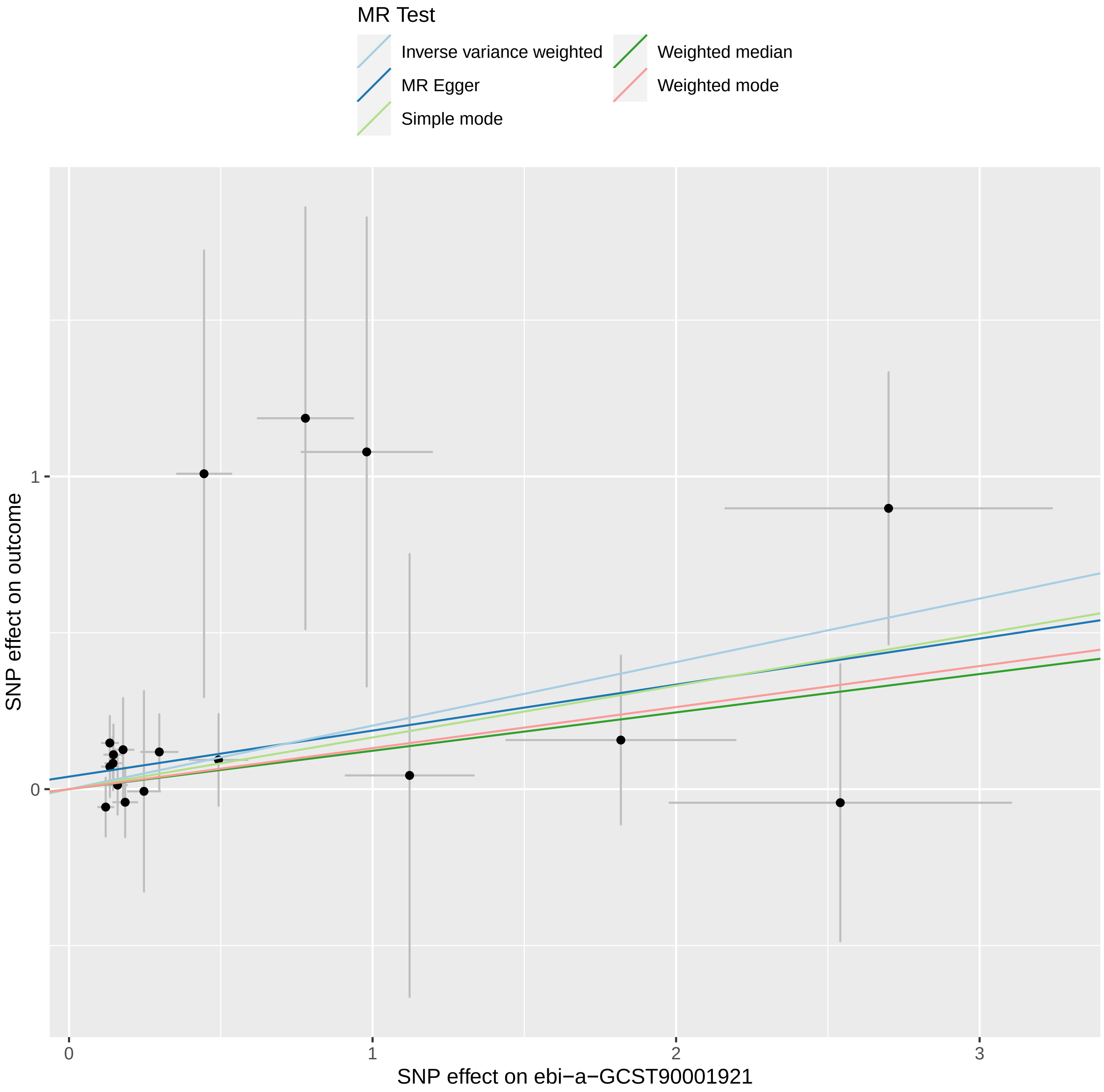
MR Test
Inverse variance weighted
MR Egger
Simple mode
Weighted median
Weighted mode
SNP effect on outcome
1
0
0
1
2
3
SNP effect on ebi−a−GCST90001921

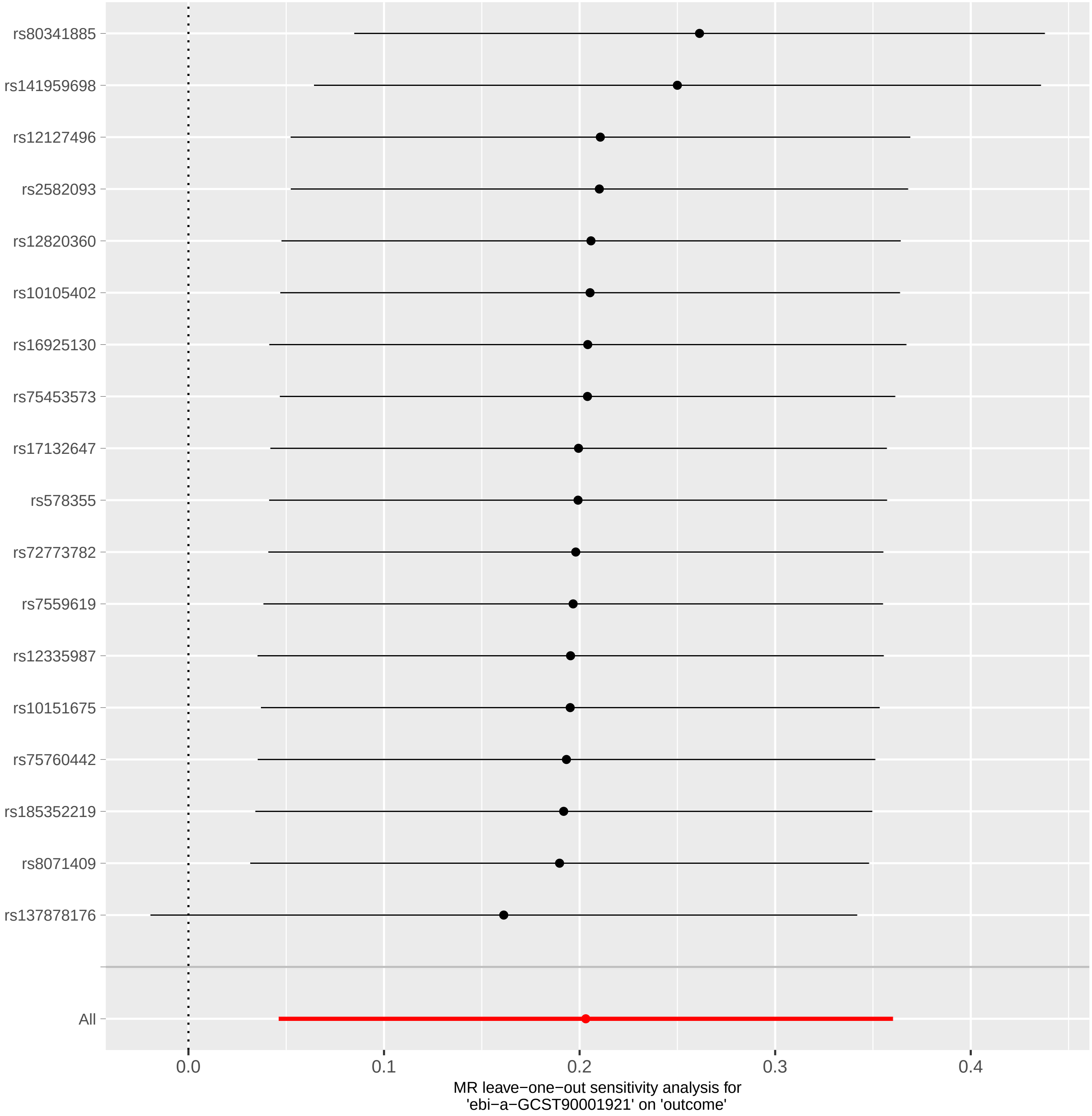

rs80341885
rs141959698
rs12127496
rs2582093
rs12820360
rs10105402
rs16925130
rs75453573
rs17132647
rs578355
rs72773782
rs7559619
rs12335987
rs10151675
rs75760442
rs185352219
rs8071409
rs137878176
All
0.0
0.1
0.2
0.3
0.4
MR leave−one−out sensitivity analysis for
'ebi−a−GCST90001921' on 'outcome'

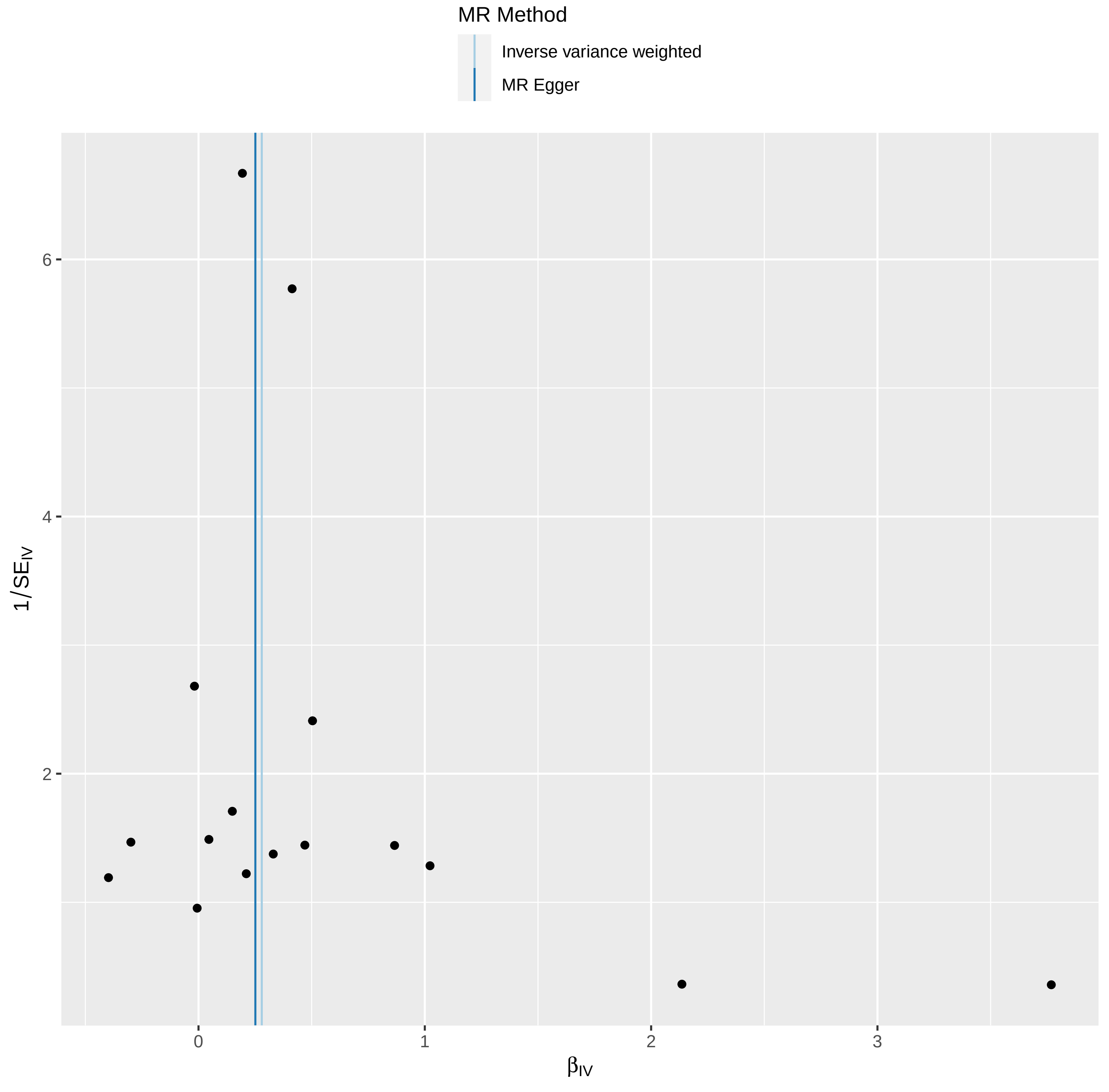
MR Method
Inverse variance weighted
MR Egger
6
4
2
1/SE_IV
0
1
2
3
β_IV

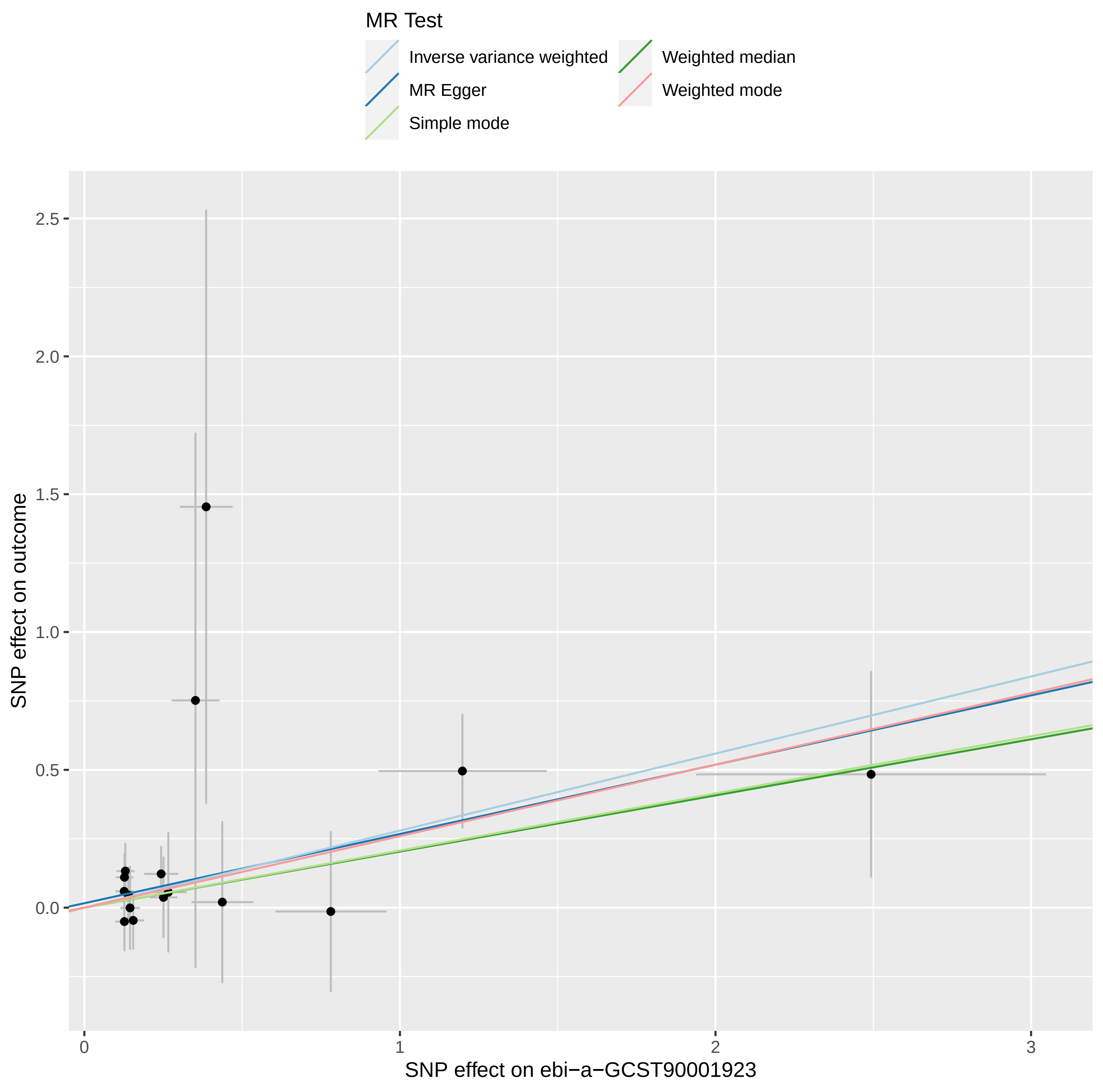
MR Test
Inverse variance weighted
MR Egger
Simple mode
Weighted median
Weighted mode
SNP effect on outcome
2.5
2.0
1.5
1.0
0.5
0.0
0
1
2
3
SNP effect on ebi−a−GCST90001923

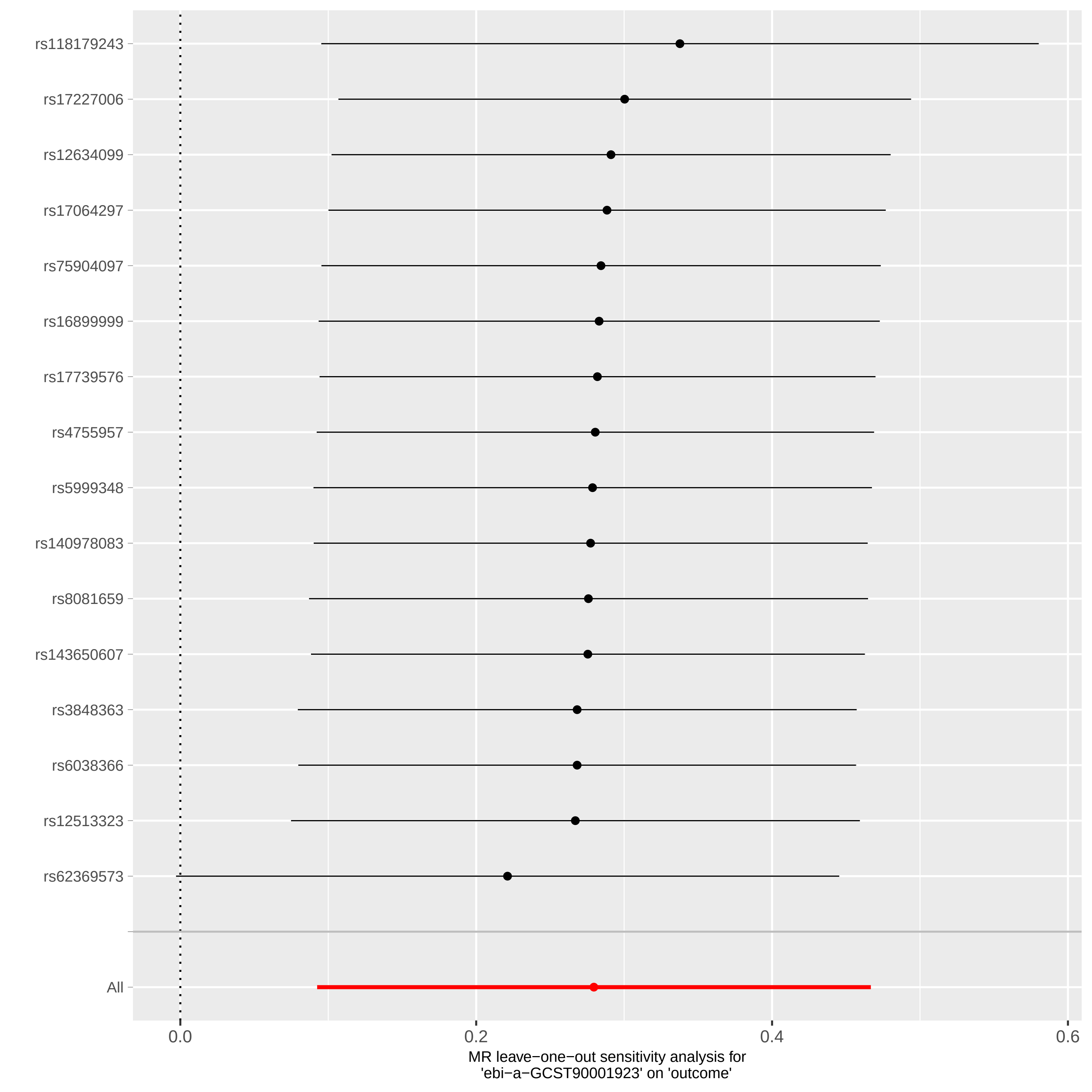

rs118179243
rs17227006
rs12634099
rs17064297
rs75904097
rs16899999
rs17739576
rs4755957
rs5999348
rs140978083
rs8081659
rs143650607
rs3848363
rs6038366
rs12513323
rs62369573
All
0.0
0.2
0.4
0.6
MR leave−one−out sensitivity analysis for
'ebi−a−GCST90001923' on 'outcome'

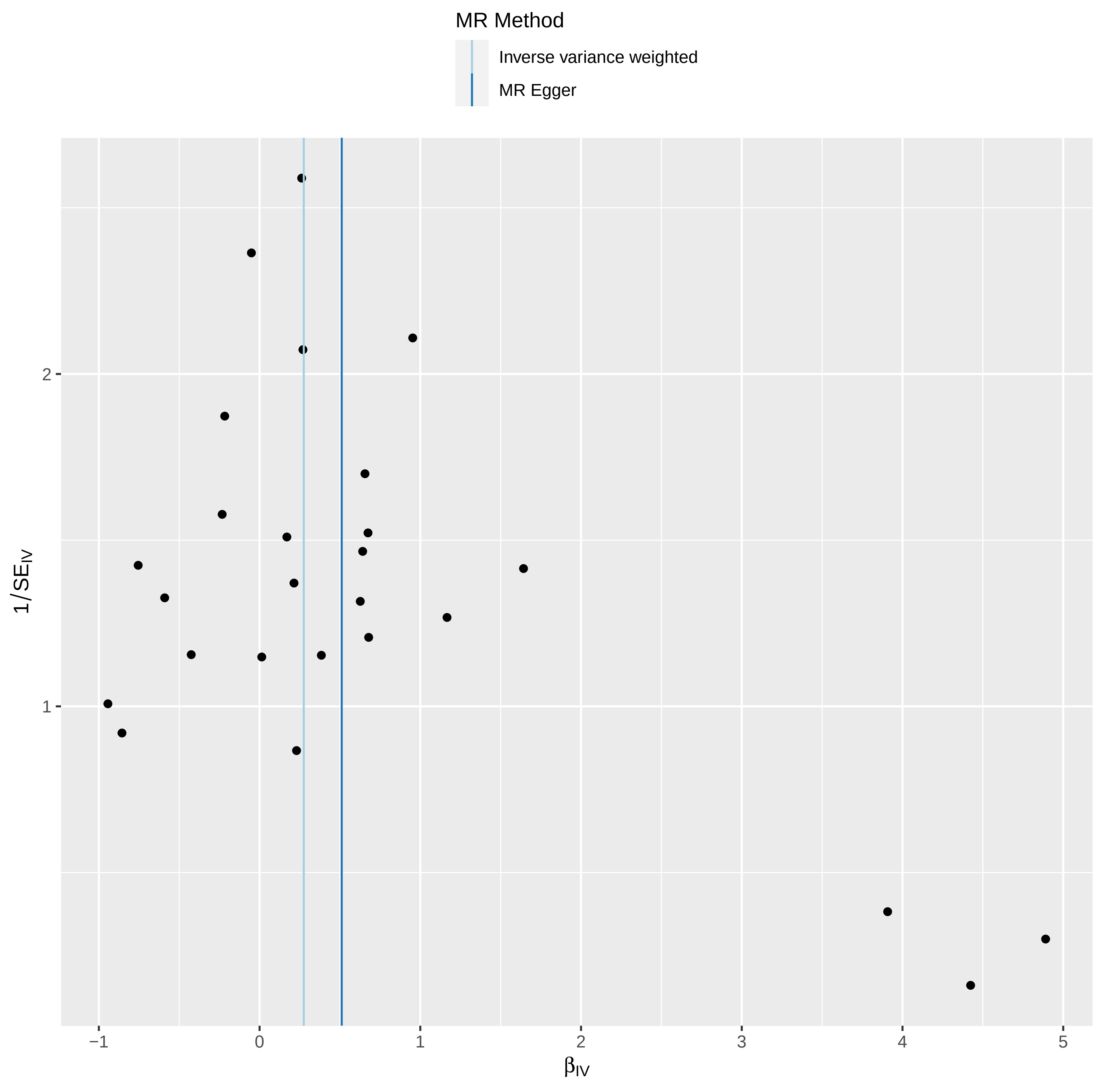
MR Method
Inverse variance weighted
MR Egger
1/SE_{IV}
2
1
−1
0
1
2
3
4
5
β_{IV}

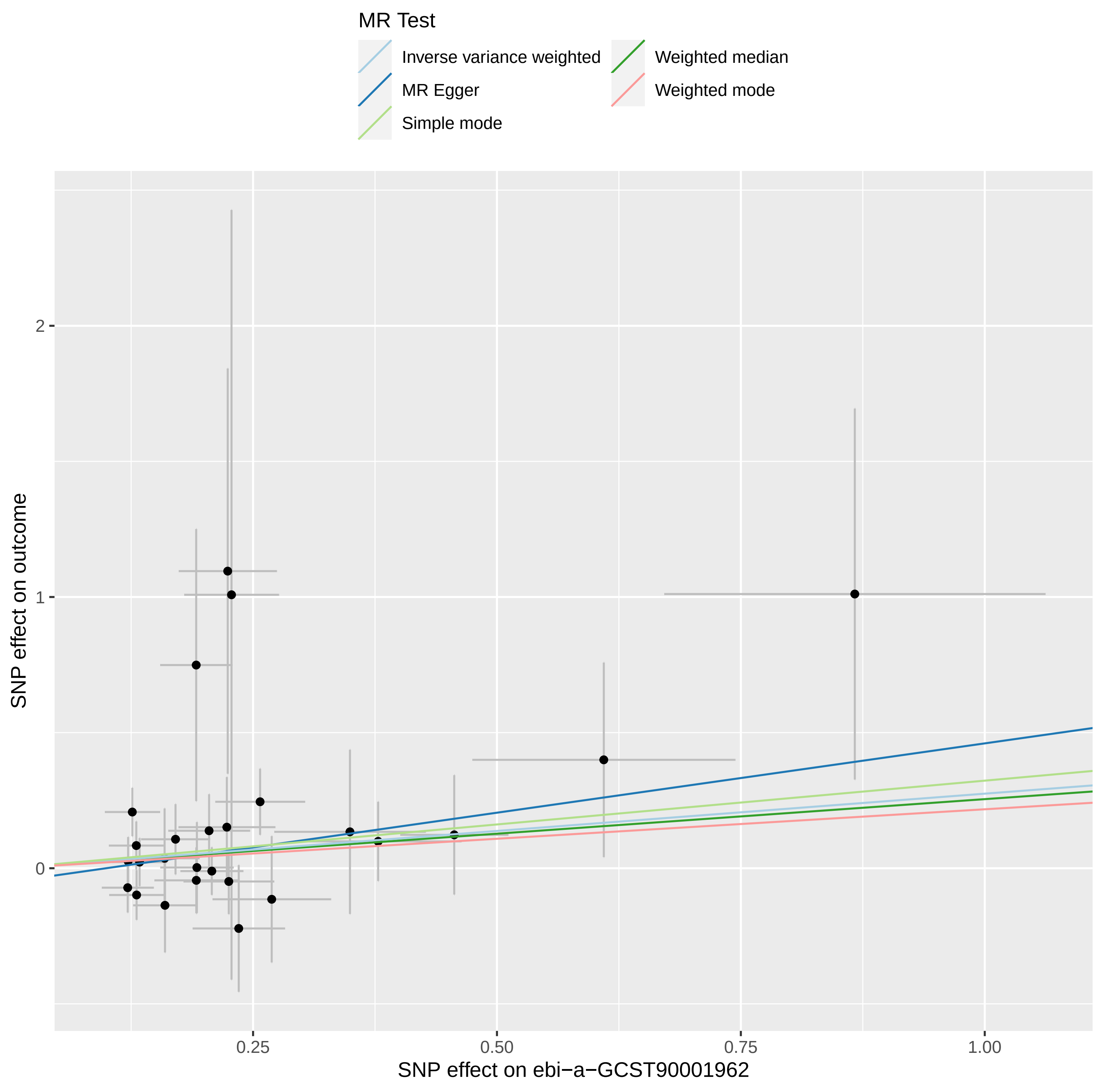
MR Test
Inverse variance weighted
MR Egger
Simple mode
Weighted median
Weighted mode
SNP effect on outcome
2
1
0
0.25
0.50
0.75
1.00
SNP effect on ebi−a−GCST90001962

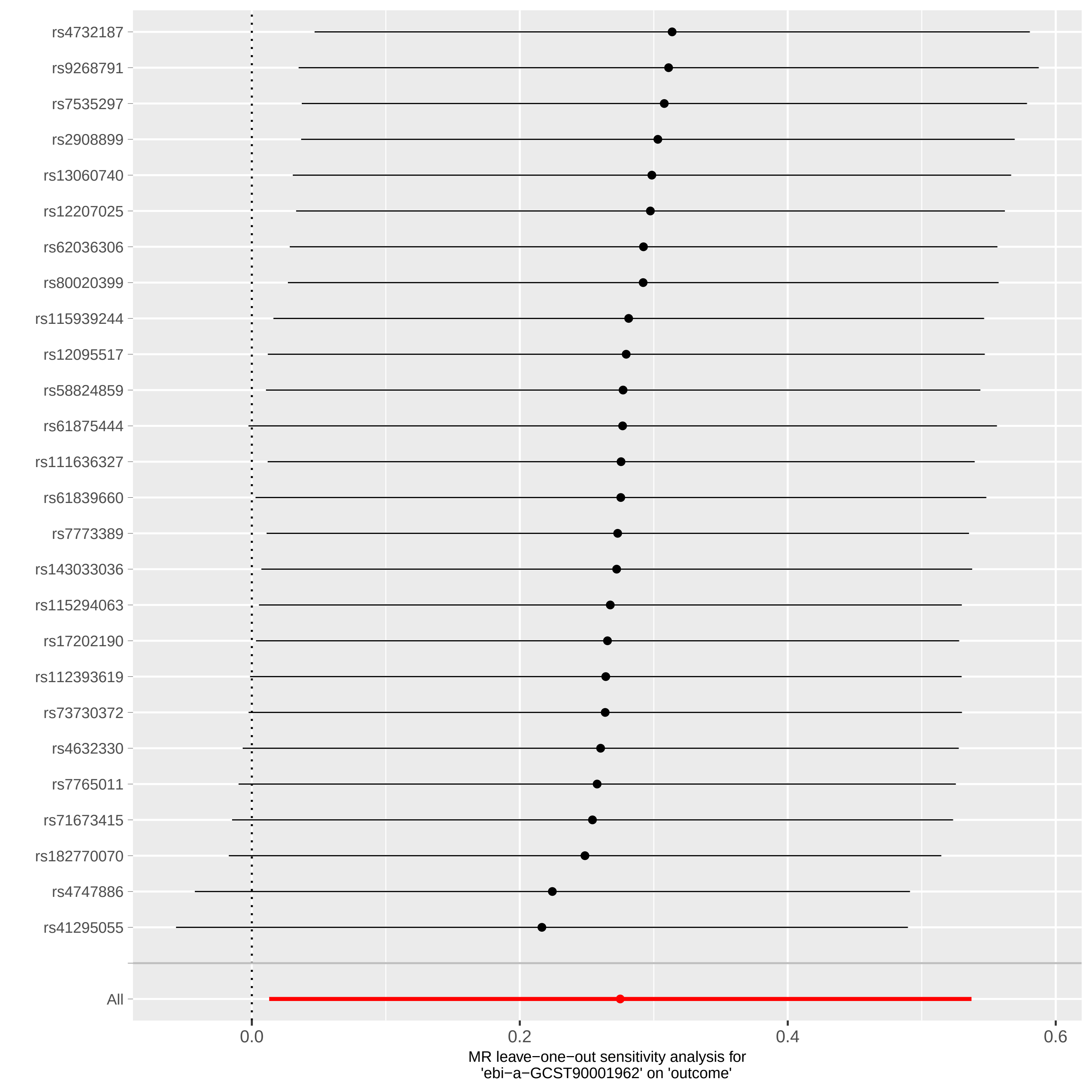
rs4732187
rs9268791
rs7535297
rs2908899
rs13060740
rs12207025
rs62036306
rs80020399
rs115939244
rs12095517
rs58824859
rs61875444
rs111636327
rs61839660
rs7773389
rs143033036
rs115294063
rs17202190
rs112393619
rs73730372
rs4632330
rs7765011
rs71673415
rs182770070
rs4747886
rs41295055
All
0.0
0.2
0.4
0.6
MR leave−one−out sensitivity analysis for
'ebi−a−GCST90001962' on 'outcome'

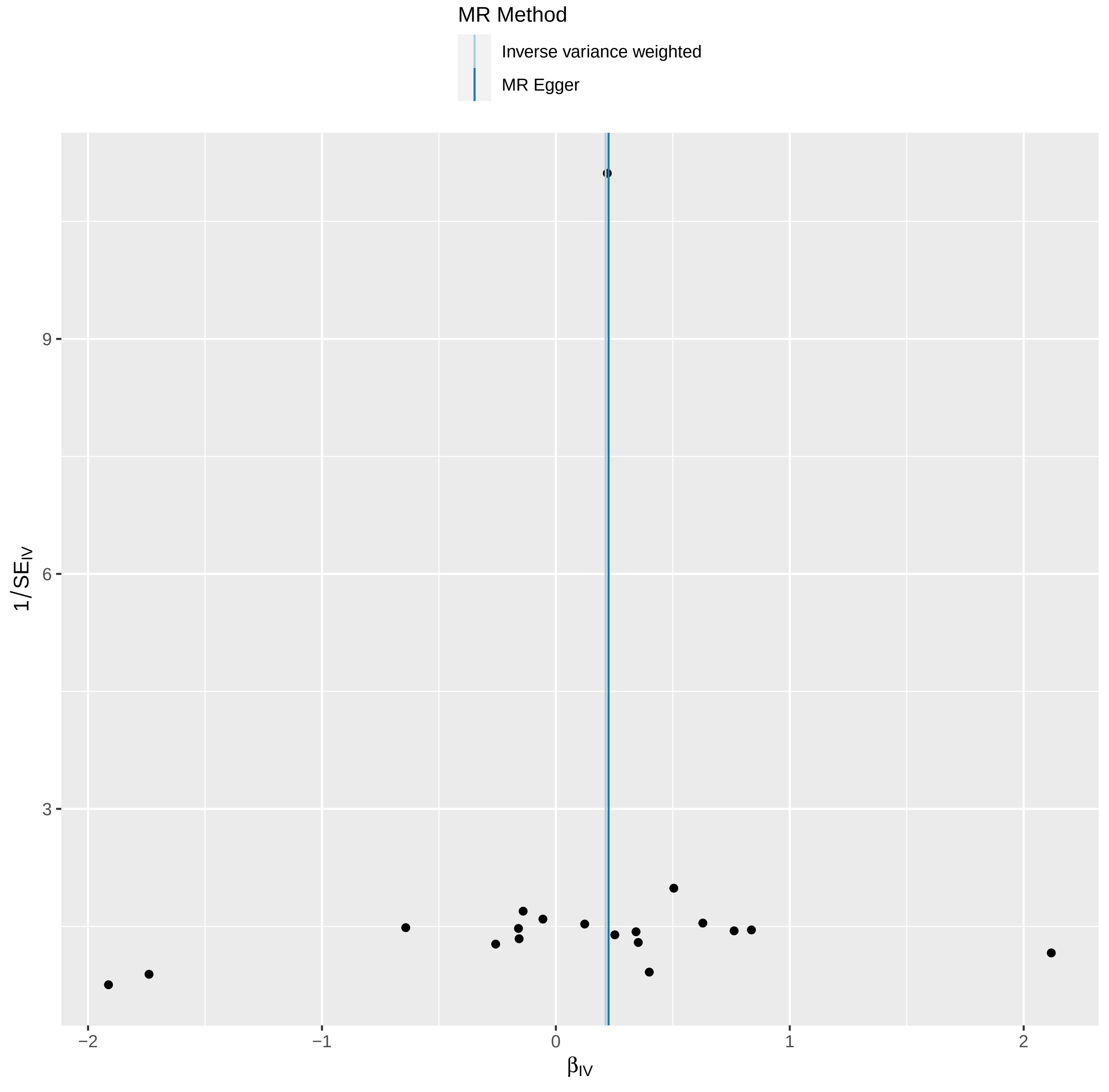

MR Method
Inverse variance weighted
MR Egger
9
6
3
1/SE_IV
−2
−1
0
1
2
β_IV

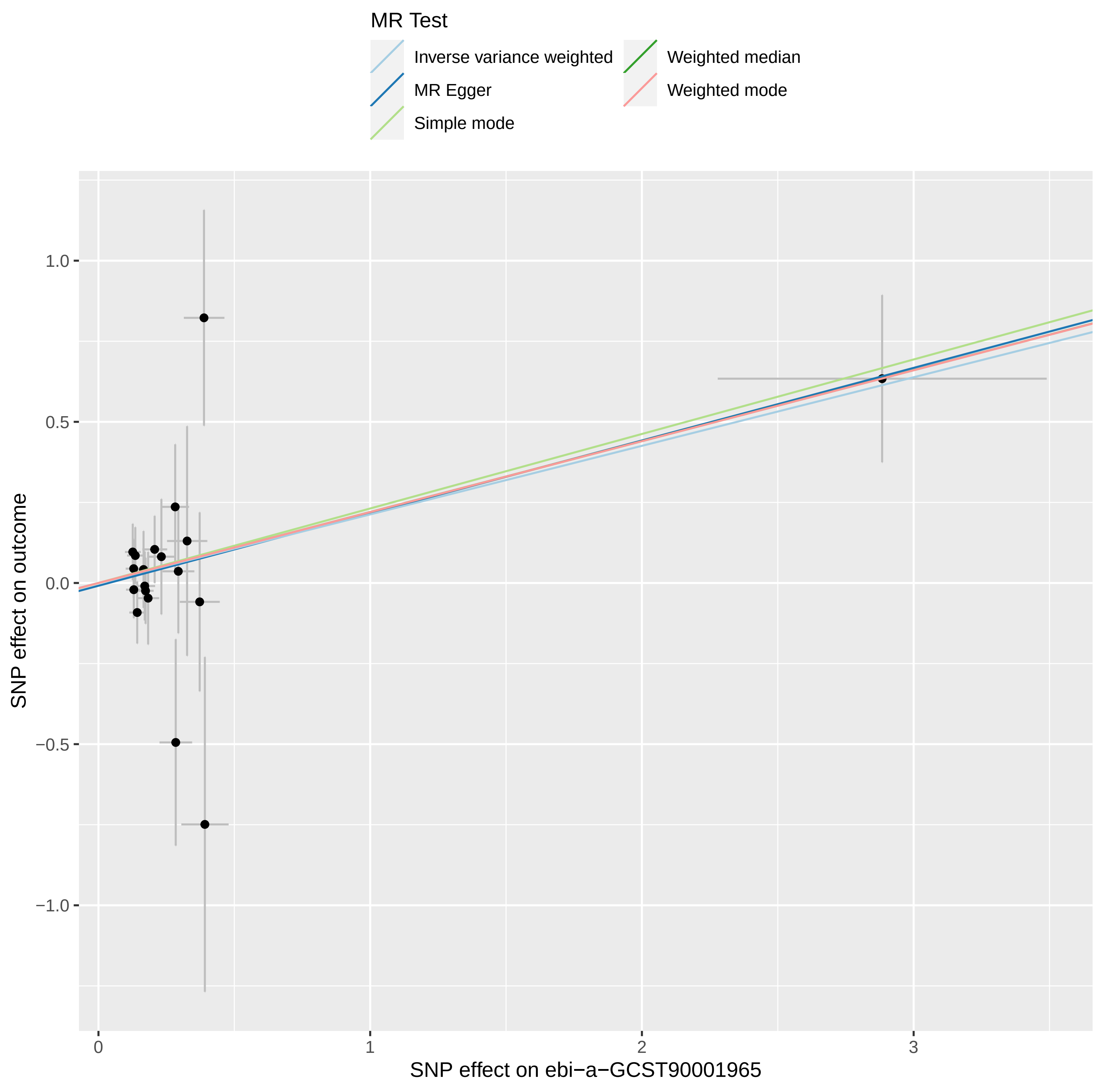

MR Test
Inverse variance weighted
MR Egger
Simple mode
Weighted median
Weighted mode
1.0
0.5
0.0
−0.5
−1.0
SNP effect on outcome
0
1
2
3
SNP effect on ebi−a−GCST90001965

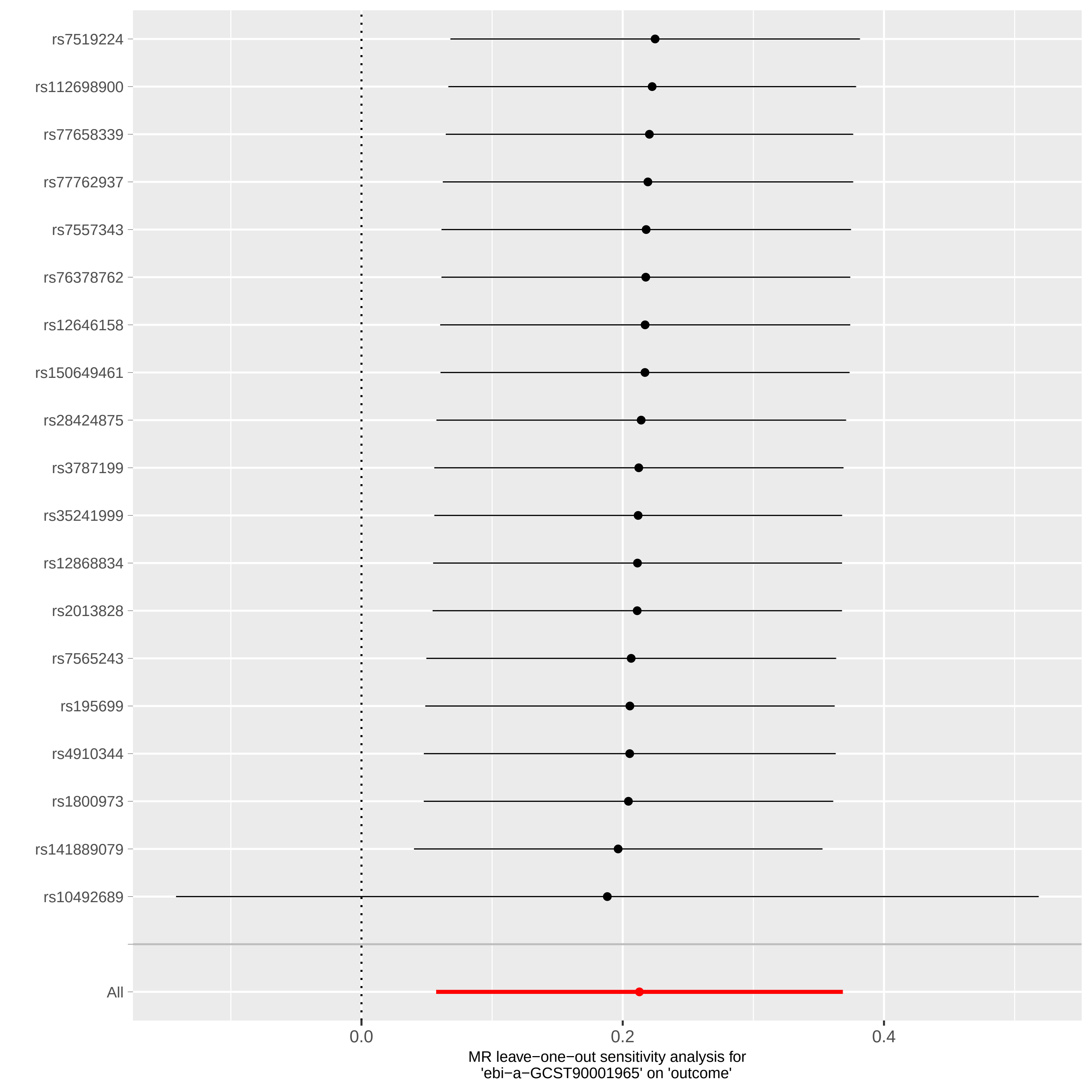
rs7519224
rs112698900
rs77658339
rs77762937
rs7557343
rs76378762
rs12646158
rs150649461
rs28424875
rs3787199
rs35241999
rs12868834
rs2013828
rs7565243
rs195699
rs4910344
rs1800973
rs141889079
rs10492689
All
0.0
0.2
0.4
MR leave−one−out sensitivity analysis for
'ebi−a−GCST90001965' on 'outcome'

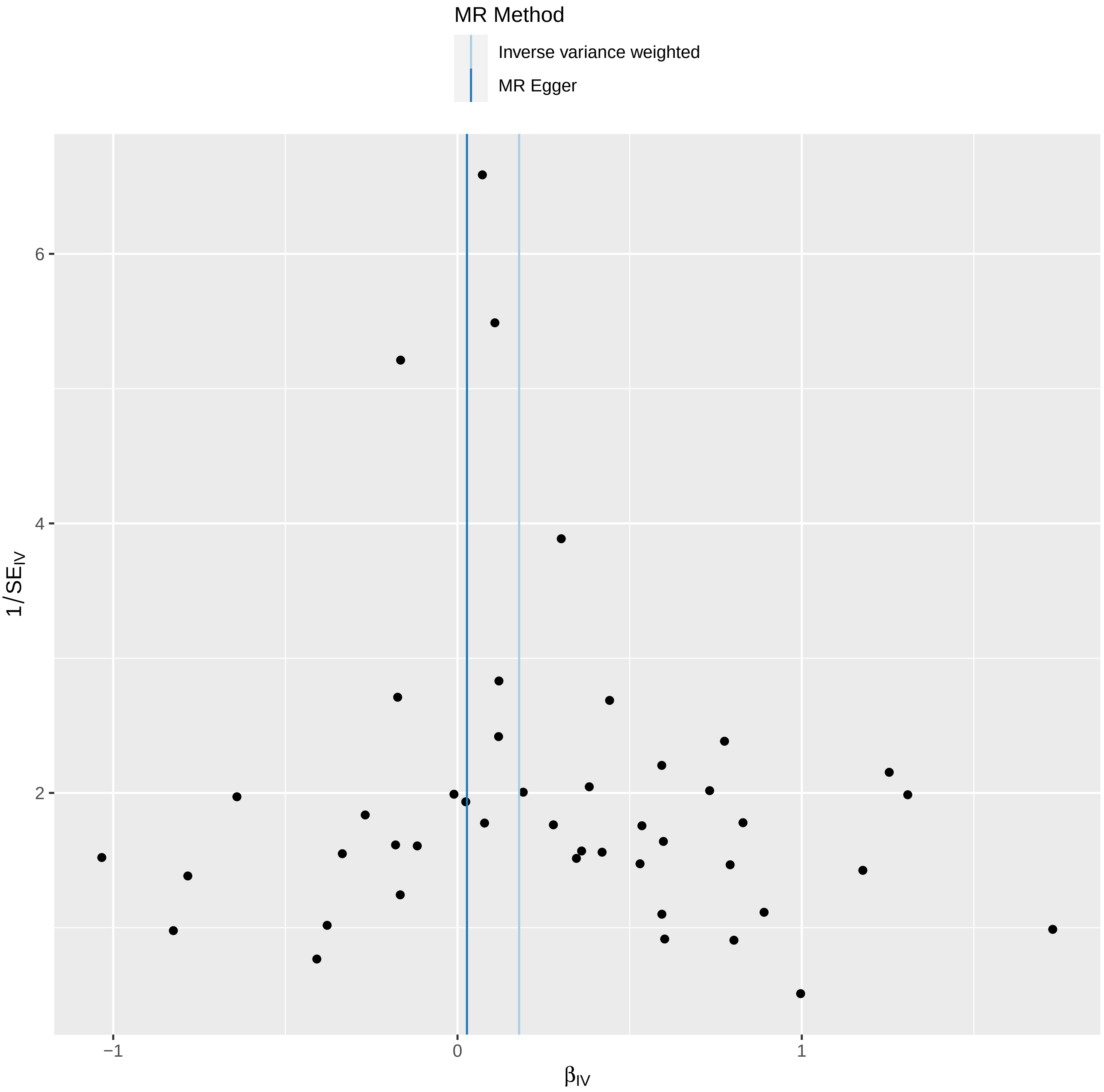

MR Method
Inverse variance weighted
MR Egger
6
4
2
1/SE_IV
−1
0
1
β_IV

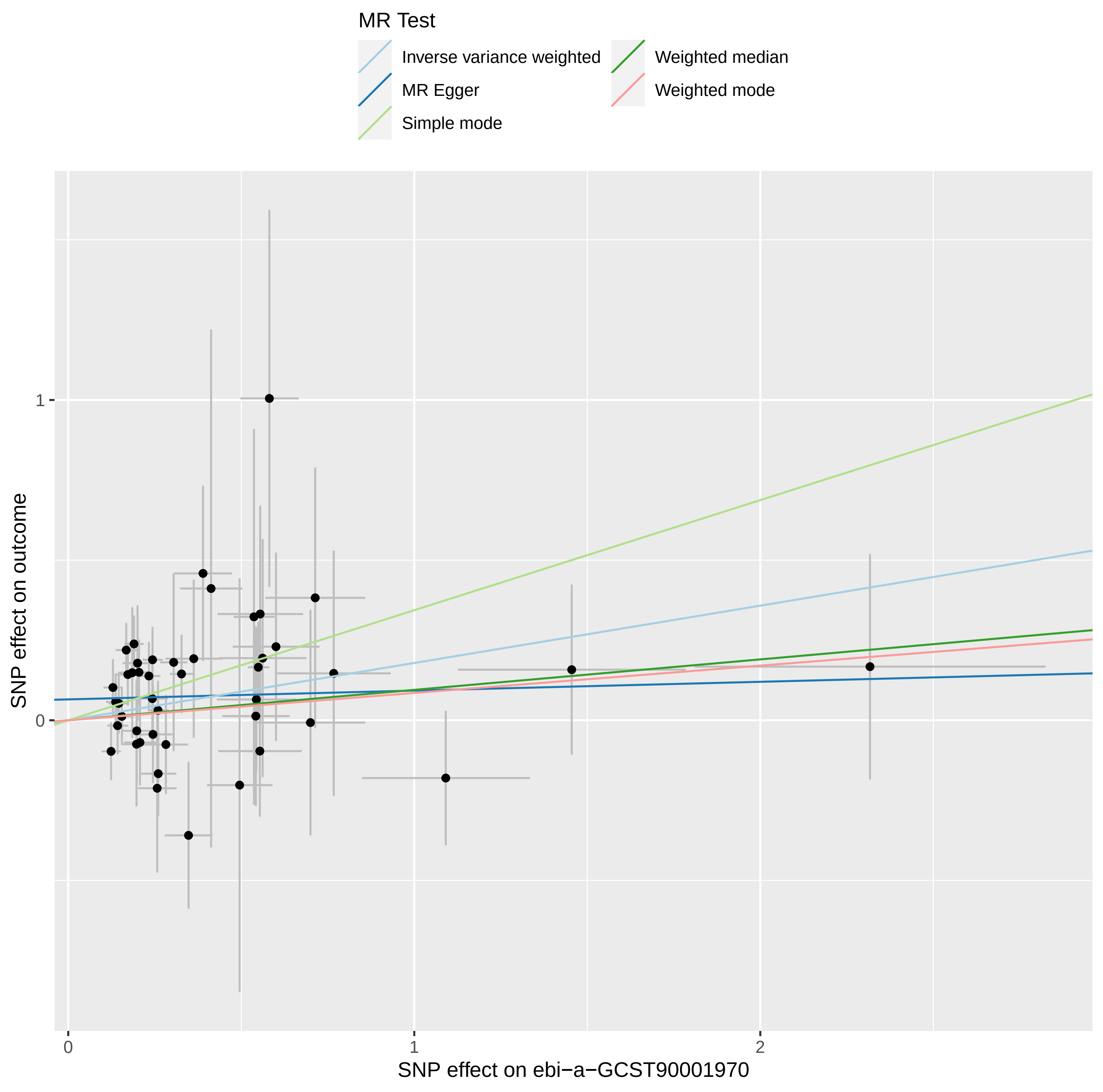
MR Test
Inverse variance weighted
MR Egger
Simple mode
Weighted median
Weighted mode
SNP effect on outcome
1
0
0
1
2
SNP effect on ebi−a−GCST90001970

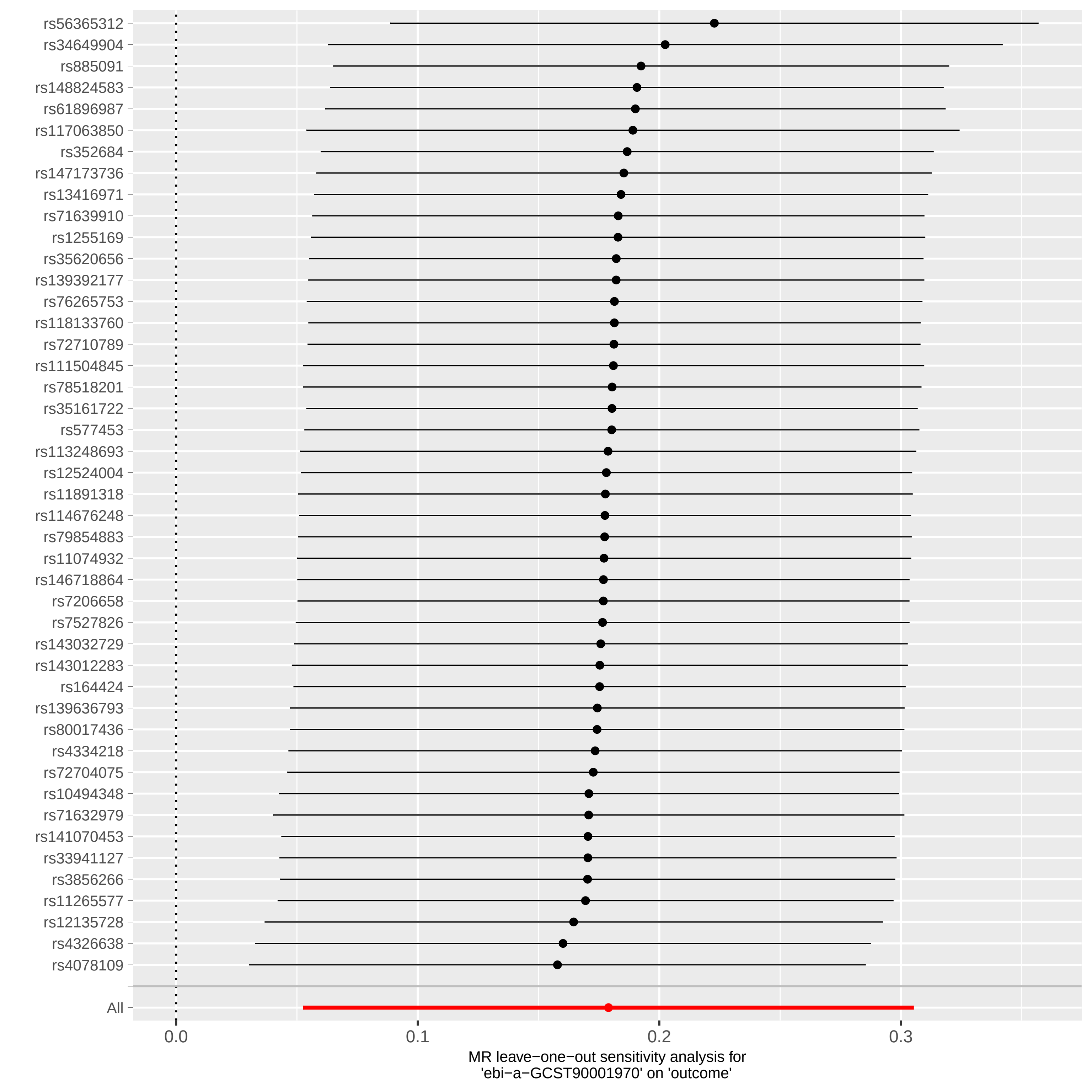

rs56365312
rs34649904
rs885091
rs148824583
rs61896987
rs117063850
rs352684
rs147173736
rs13416971
rs71639910
rs1255169
rs35620656
rs139392177
rs76265753
rs118133760
rs72710789
rs111504845
rs78518201
rs35161722
rs577453
rs113248693
rs12524004
rs11891318
rs114676248
rs79854883
rs11074932
rs146718864
rs7206658
rs7527826
rs143032729
rs143012283
rs164424
rs139636793
rs80017436
rs4334218
rs72704075
rs10494348
rs71632979
rs141070453
rs33941127
rs3856266
rs11265577
rs12135728
rs4326638
rs4078109
All
0.0
0.1
0.2
0.3
MR leave−one−out sensitivity analysis for
'ebi−a−GCST90001970' on 'outcome'

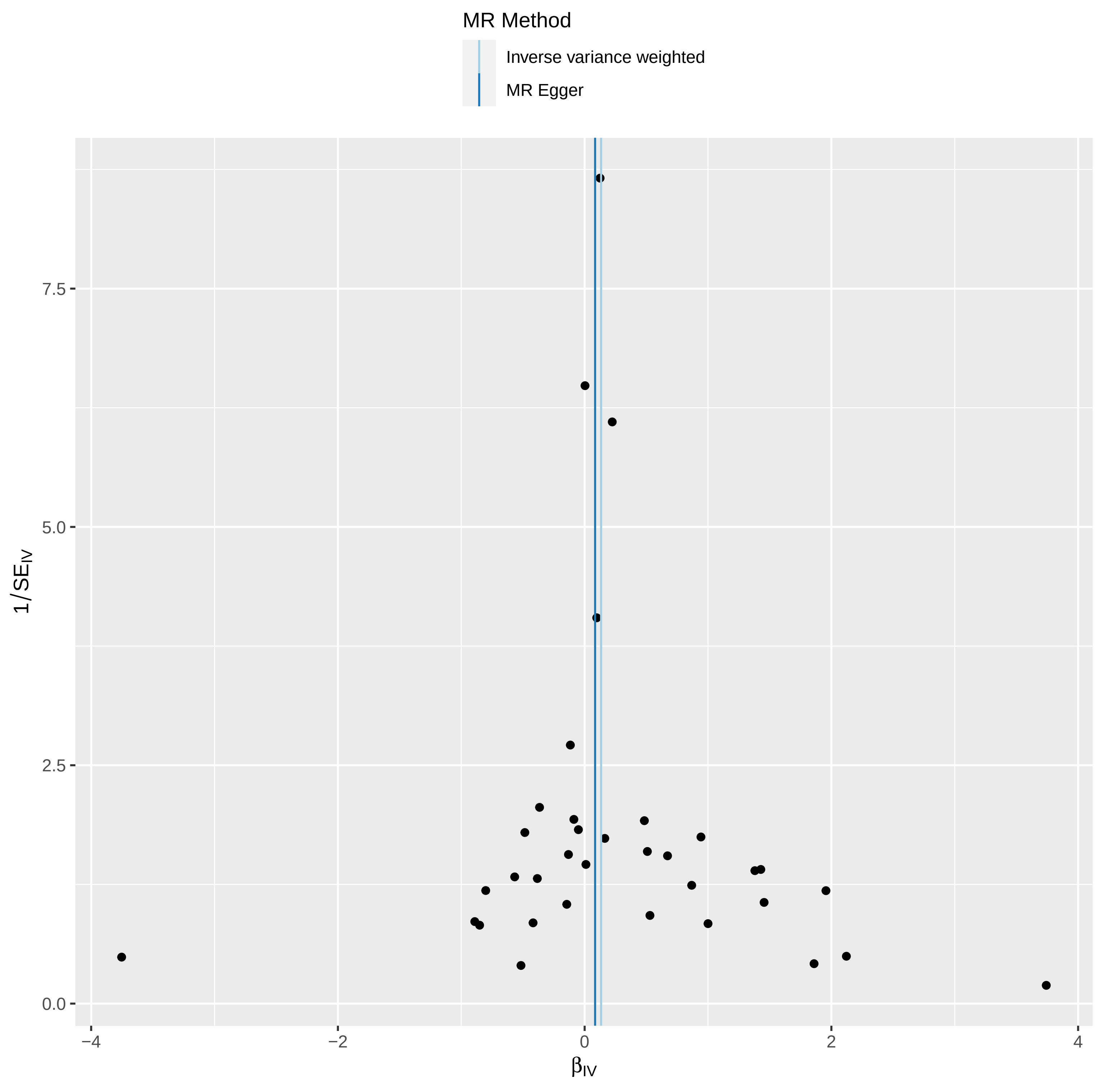

MR Method
Inverse variance weighted
MR Egger
7.5
5.0
2.5
0.0
1/SE_IV
−4
−2
0
2
4
β_IV

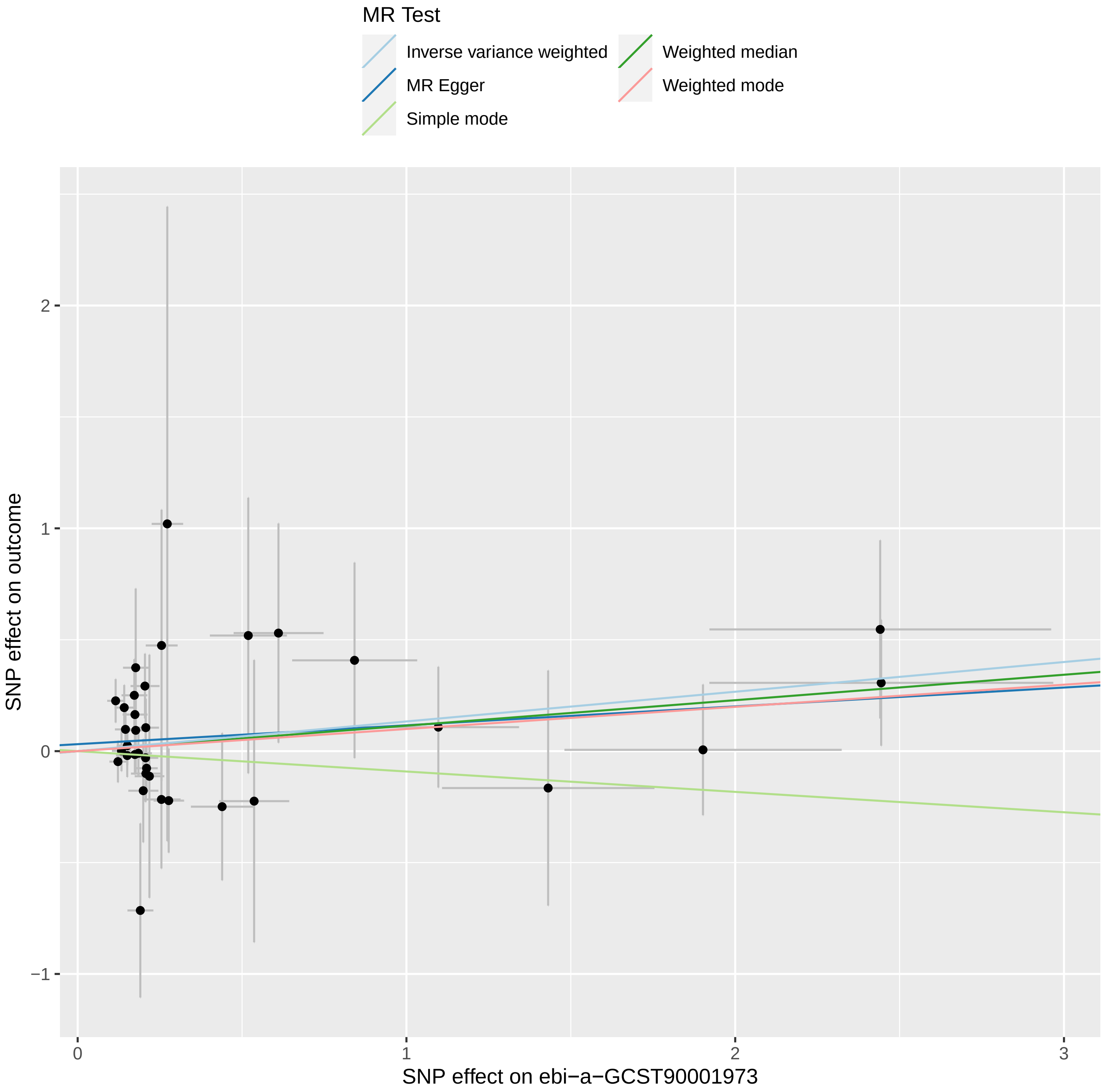

MR Test
Inverse variance weighted
MR Egger
Simple mode
Weighted median
Weighted mode
SNP effect on outcome
SNP effect on ebi−a−GCST90001973
2
1
0
−1
0
1
2
3

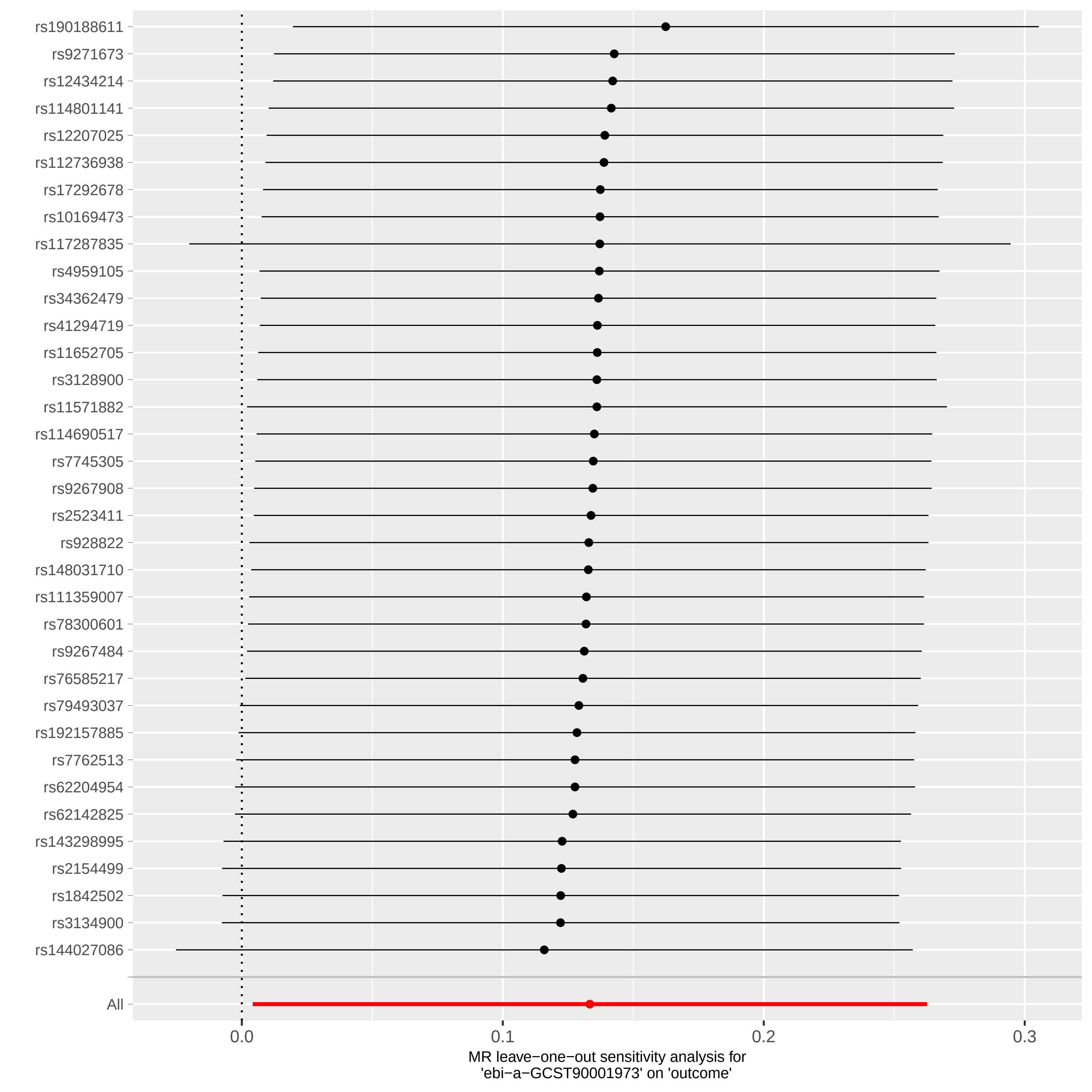

rs190188611
rs9271673
rs12434214
rs114801141
rs12207025
rs112736938
rs17292678
rs10169473
rs117287835
rs4959105
rs34362479
rs41294719
rs11652705
rs3128900
rs11571882
rs114690517
rs7745305
rs9267908
rs2523411
rs928822
rs148031710
rs111359007
rs78300601
rs9267484
rs76585217
rs79493037
rs192157885
rs7762513
rs62204954
rs62142825
rs143298995
rs2154499
rs1842502
rs3134900
rs144027086
All
0.0
0.1
0.2
0.3
MR leave−one−out sensitivity analysis for
'ebi−a−GCST90001973' on 'outcome'

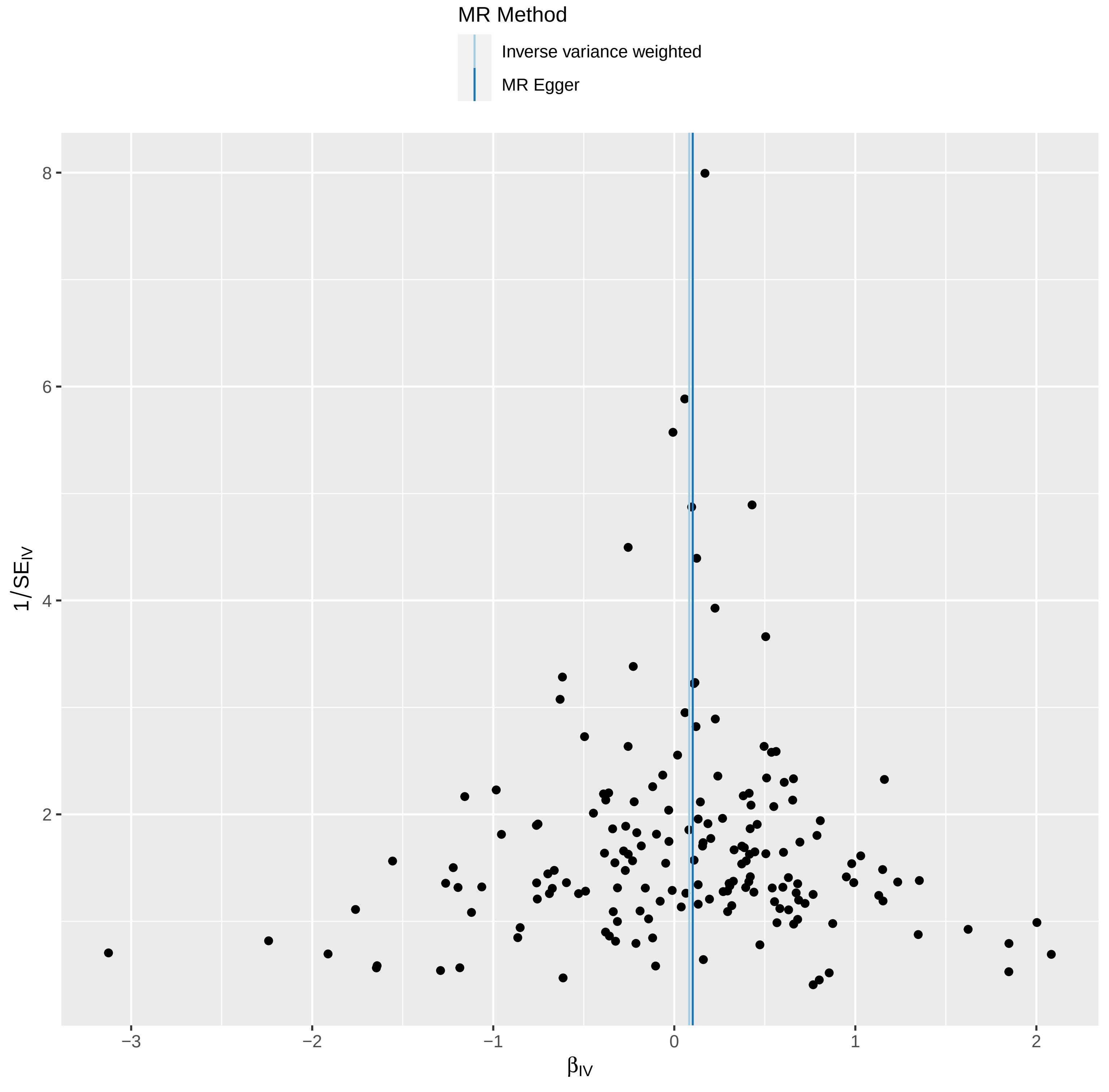

MR Method
Inverse variance weighted
MR Egger
1/SE_IV
8
6
4
2
−3
−2
−1
0
1
2
β_IV

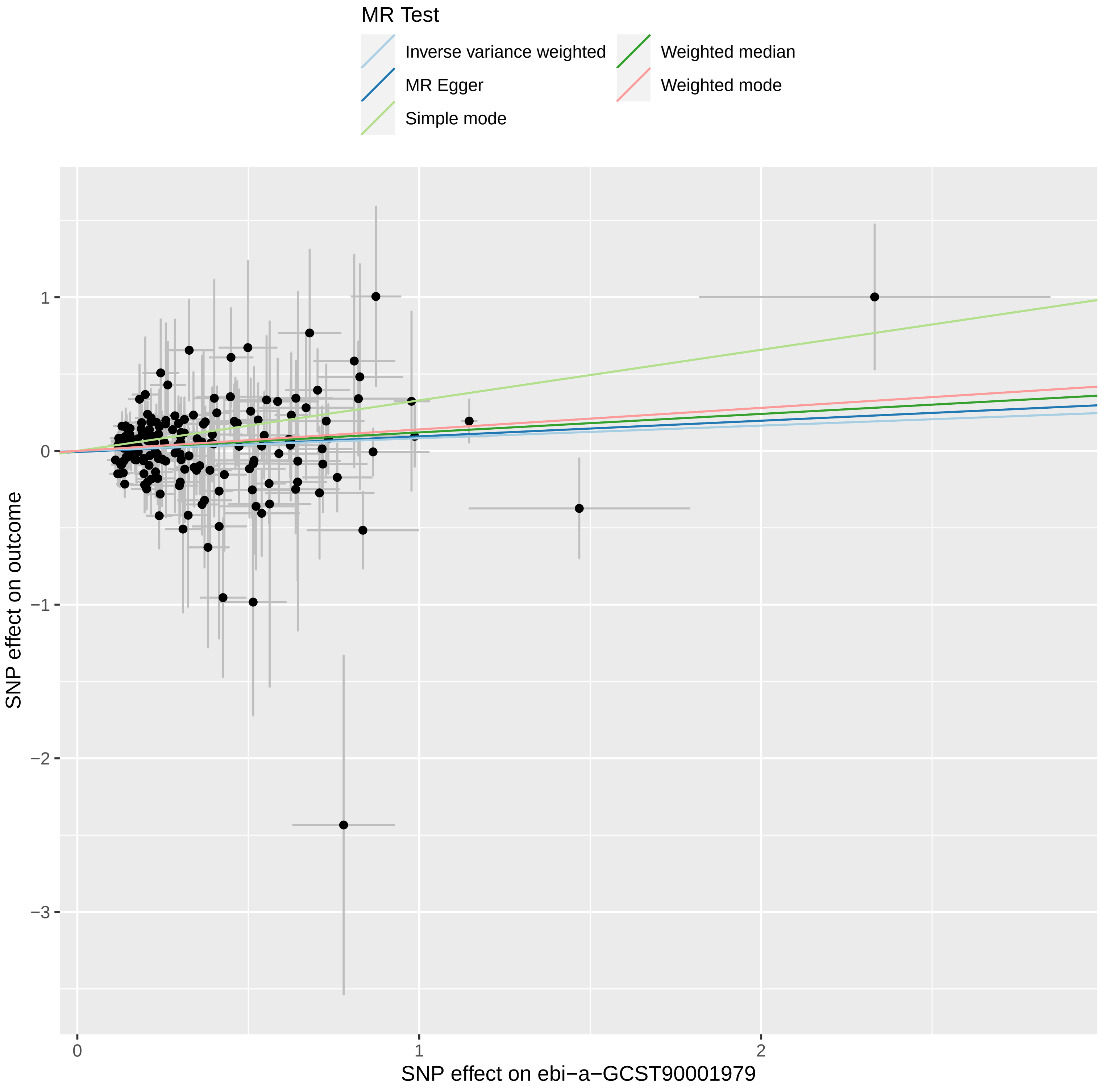
MR Test
Inverse variance weighted
MR Egger
Simple mode
Weighted median
Weighted mode
SNP effect on outcome
SNP effect on ebi−a−GCST90001979
1
0
−1
−2
−3
0
1
2

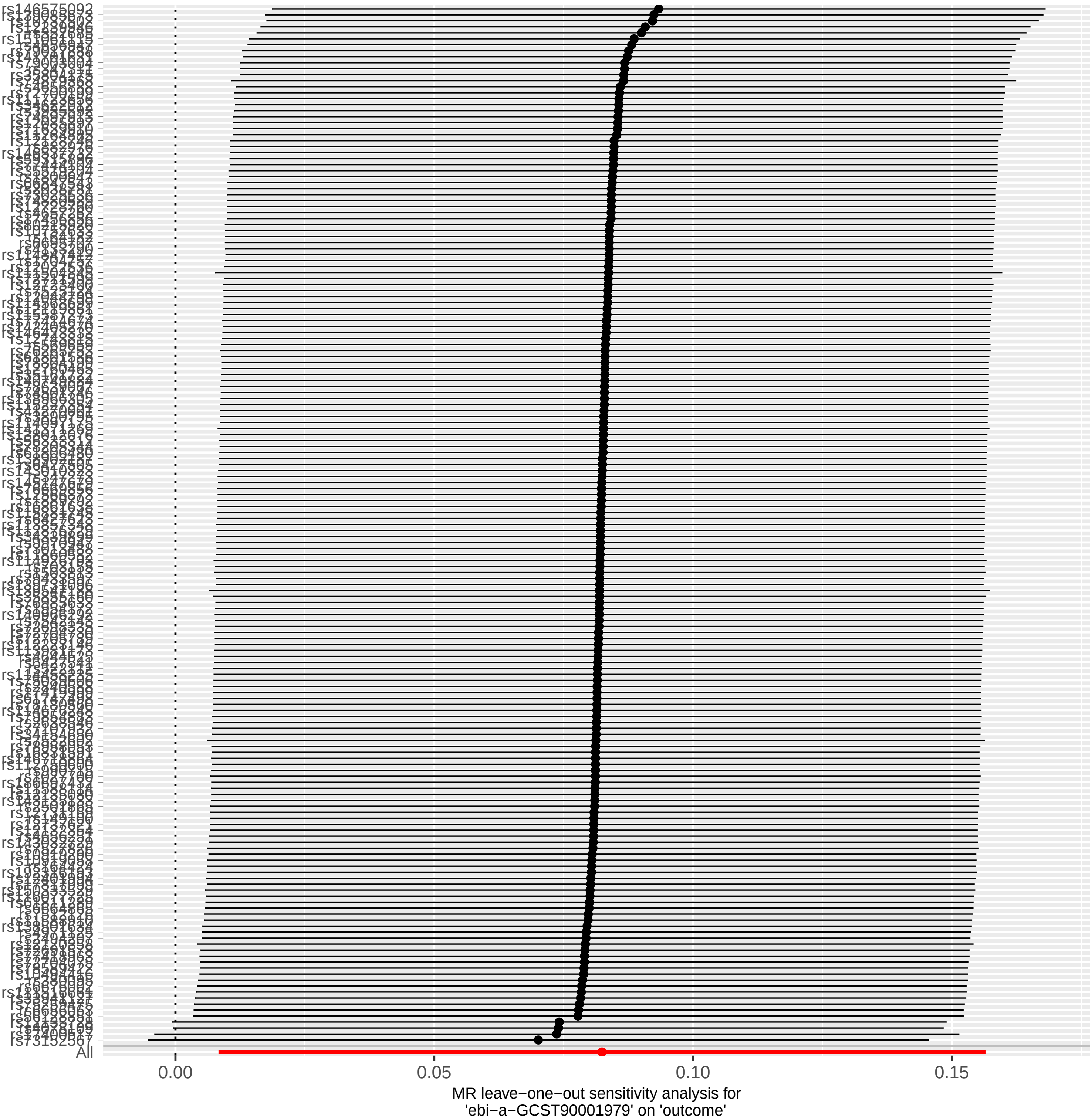
rs146575092
rs73152567
All
0.00
0.05
0.10
0.15
MR leave−one−out sensitivity analysis for
'ebi−a−GCST90001979' on 'outcome'

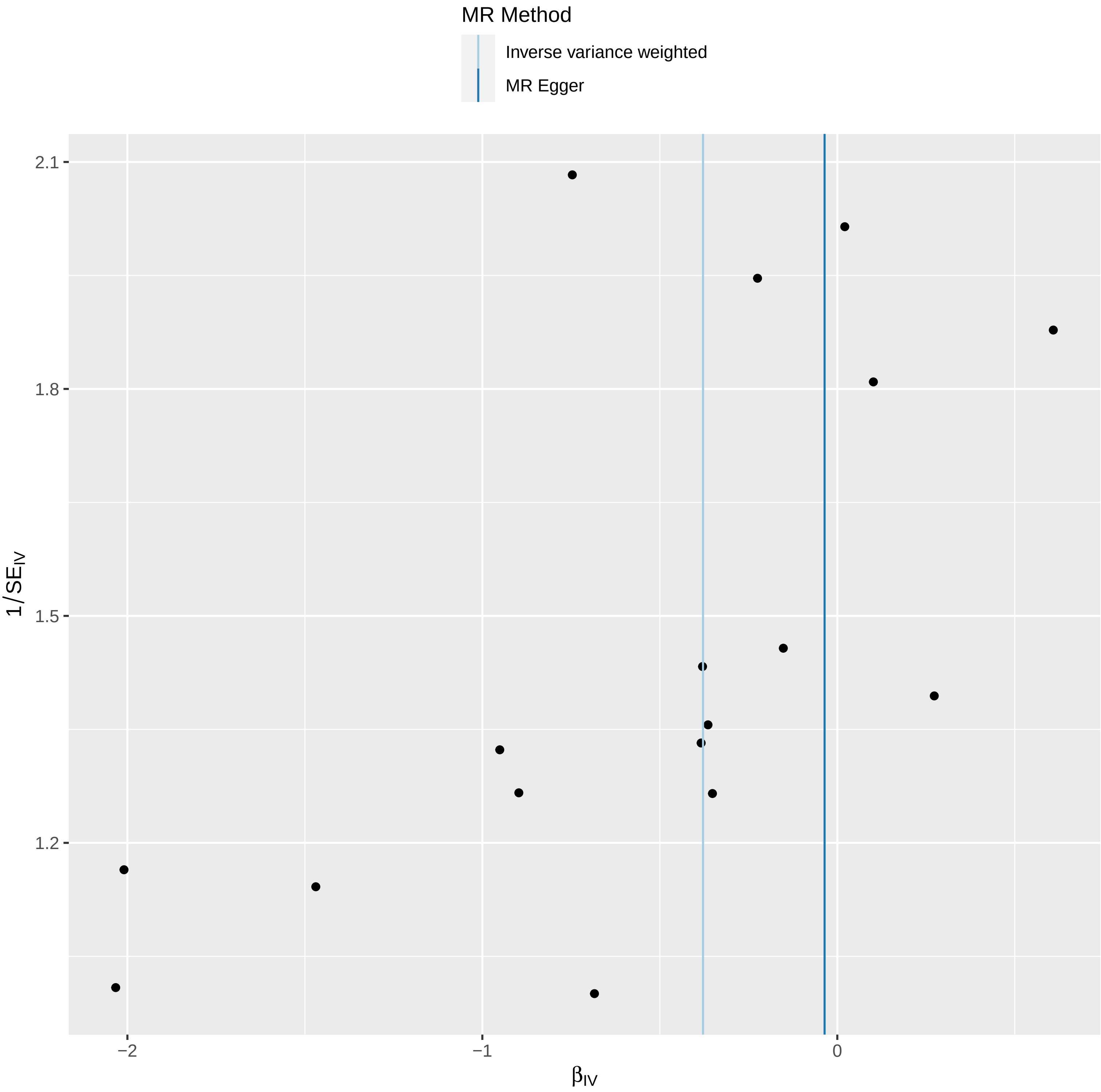
MR Method
Inverse variance weighted
MR Egger
2.1
1.8
1.5
1.2
1/SE_IV
−2
−1
0
β_IV

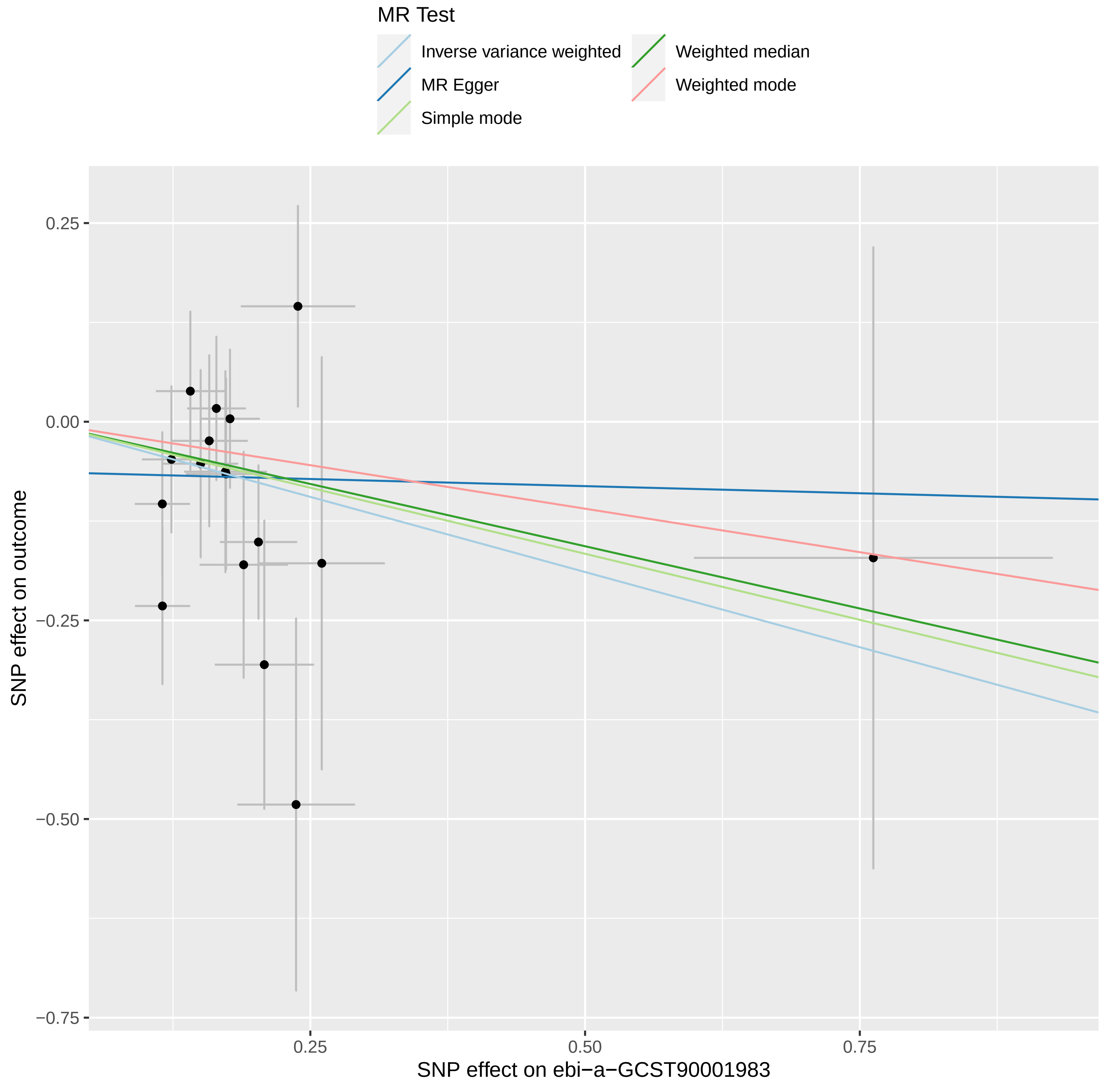
MR Test
Inverse variance weighted
MR Egger
Simple mode
Weighted median
Weighted mode
0.25
0.00
−0.25
−0.50
−0.75
SNP effect on outcome
0.25
0.50
0.75
SNP effect on ebi−a−GCST90001983

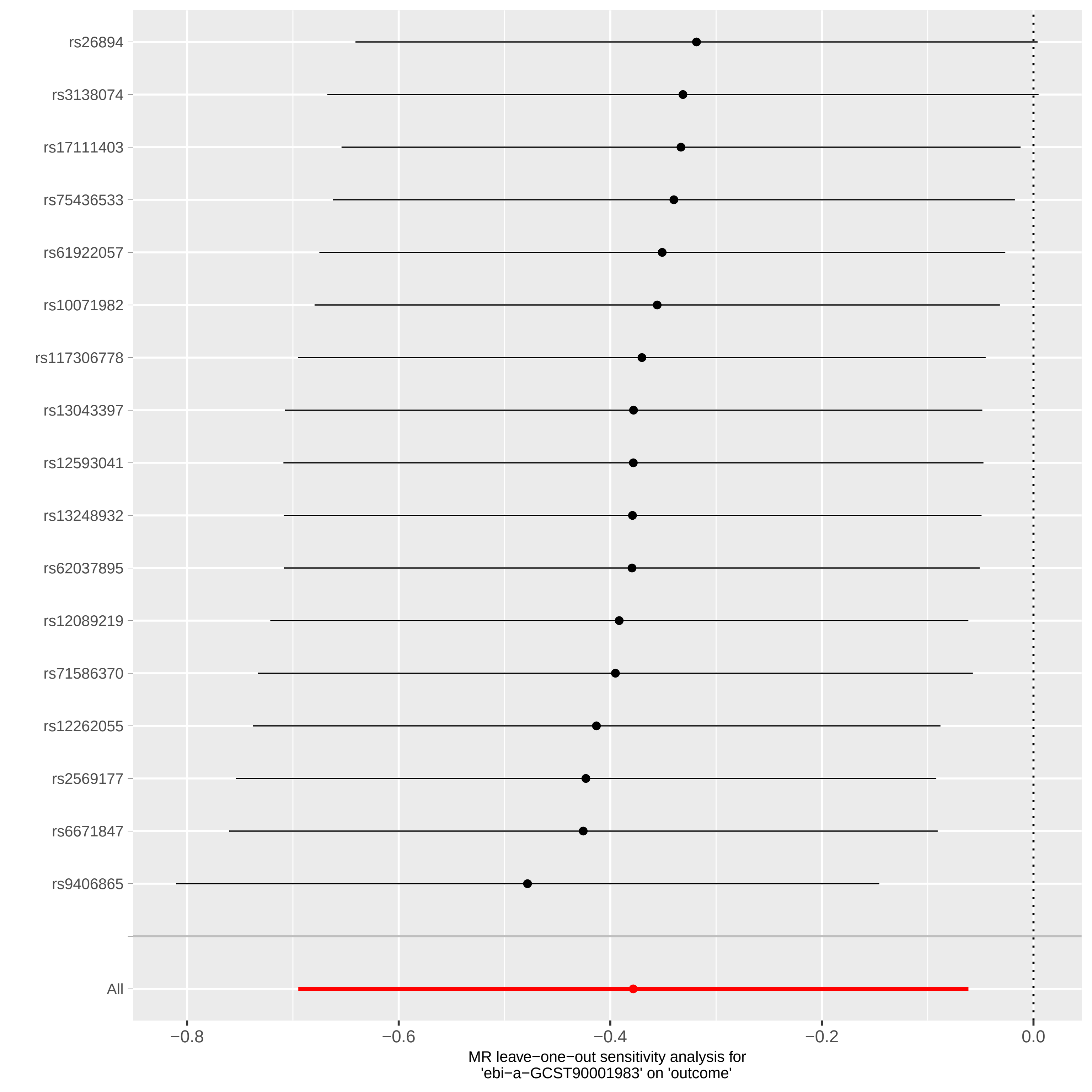
rs26894
rs3138074
rs17111403
rs75436533
rs61922057
rs10071982
rs117306778
rs13043397
rs12593041
rs13248932
rs62037895
rs12089219
rs71586370
rs12262055
rs2569177
rs6671847
rs9406865
All
−0.8
−0.6
−0.4
−0.2
0.0
MR leave−one−out sensitivity analysis for
'ebi−a−GCST90001983' on 'outcome'

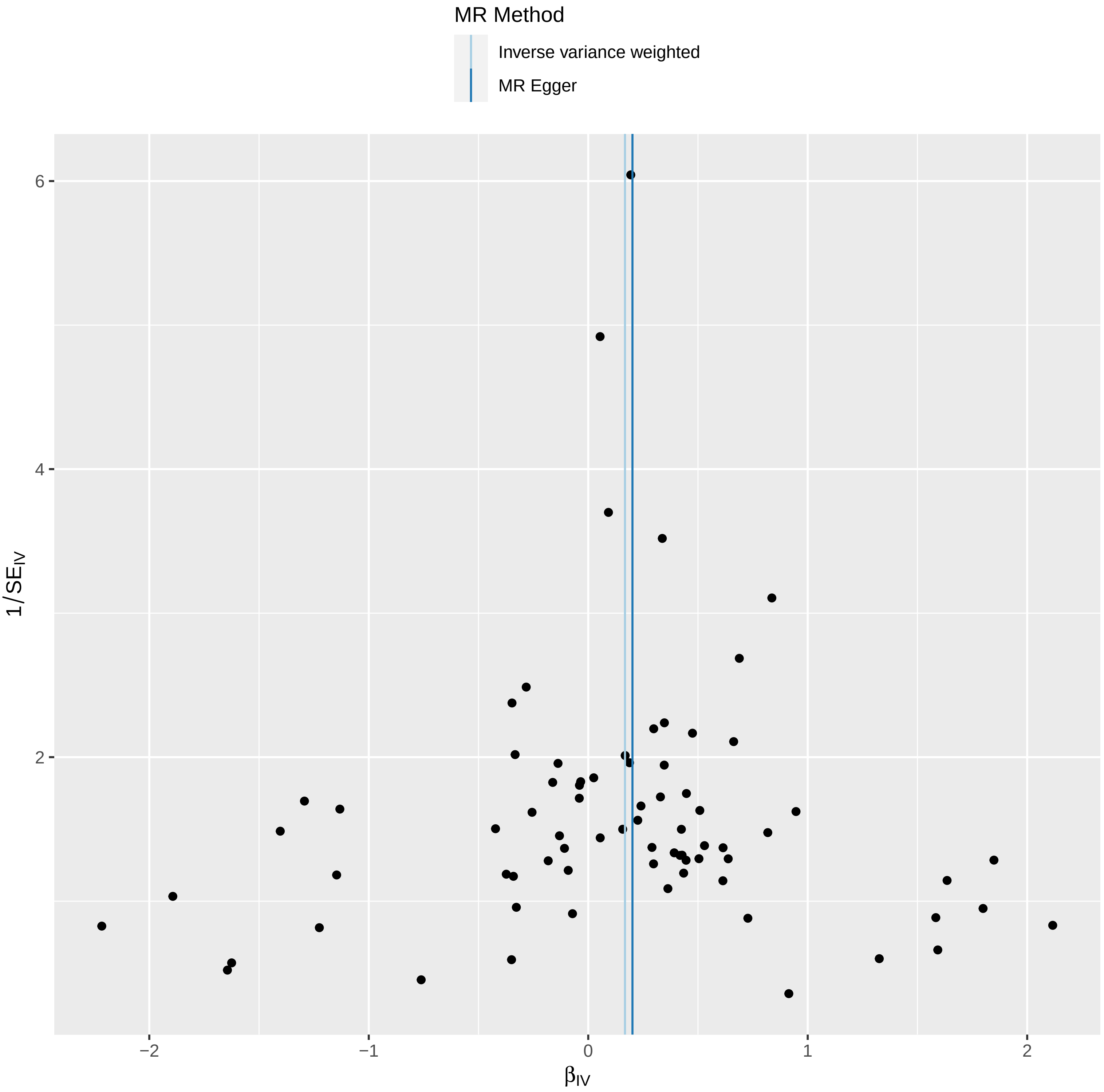

MR Method
Inverse variance weighted
MR Egger
1/SE_IV
6
4
2
−2
−1
0
1
2
β_IV

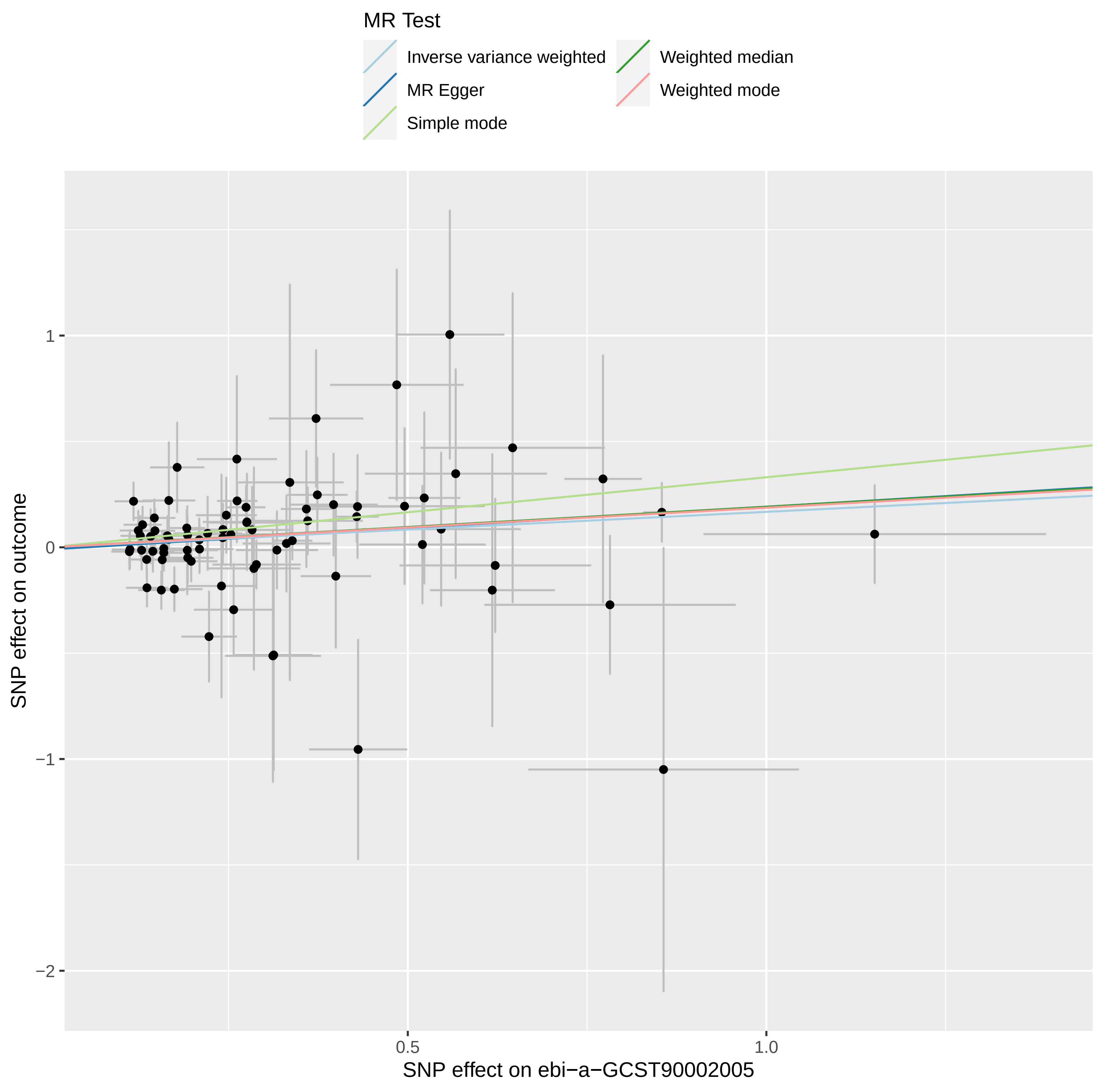

MR Test
Inverse variance weighted
MR Egger
Simple mode
Weighted median
Weighted mode
SNP effect on outcome
SNP effect on ebi−a−GCST90002005
1
0
−1
−2
0.5
1.0

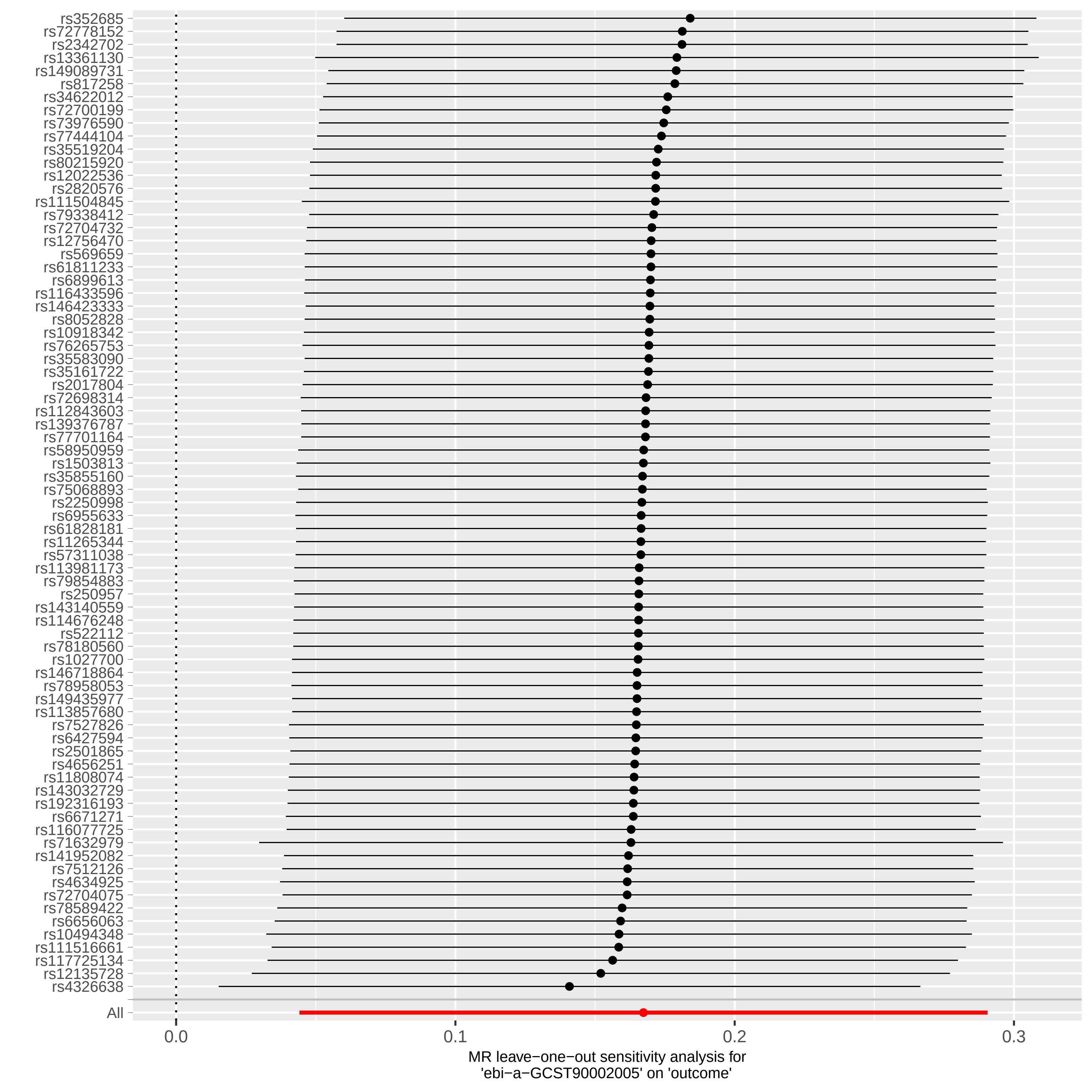

rs352685
rs72778152
rs2342702
rs13361130
rs149089731
rs817258
rs34622012
rs72700199
rs73976590
rs77444104
rs35519204
rs80215920
rs12022536
rs2820576
rs111504845
rs79338412
rs72704732
rs12756470
rs569659
rs61811233
rs6899613
rs116433596
rs146423333
rs8052828
rs10918342
rs76265753
rs35583090
rs35161722
rs2017804
rs72698314
rs112843603
rs139376787
rs77701164
rs58950959
rs1503813
rs35855160
rs75068893
rs2250998
rs6955633
rs61828181
rs11265344
rs57311038
rs113981173
rs79854883
rs250957
rs143140559
rs114676248
rs522112
rs78180560
rs1027700
rs146718864
rs78958053
rs149435977
rs113857680
rs7527826
rs6427594
rs2501865
rs4656251
rs11808074
rs143032729
rs192316193
rs6671271
rs116077725
rs71632979
rs141952082
rs7512126
rs4634925
rs72704075
rs78589422
rs6656063
rs10494348
rs111516661
rs117725134
rs12135728
rs4326638
All
0.0
0.1
0.2
0.3
MR leave−one−out sensitivity analysis for
'ebi−a−GCST90002005' on 'outcome'

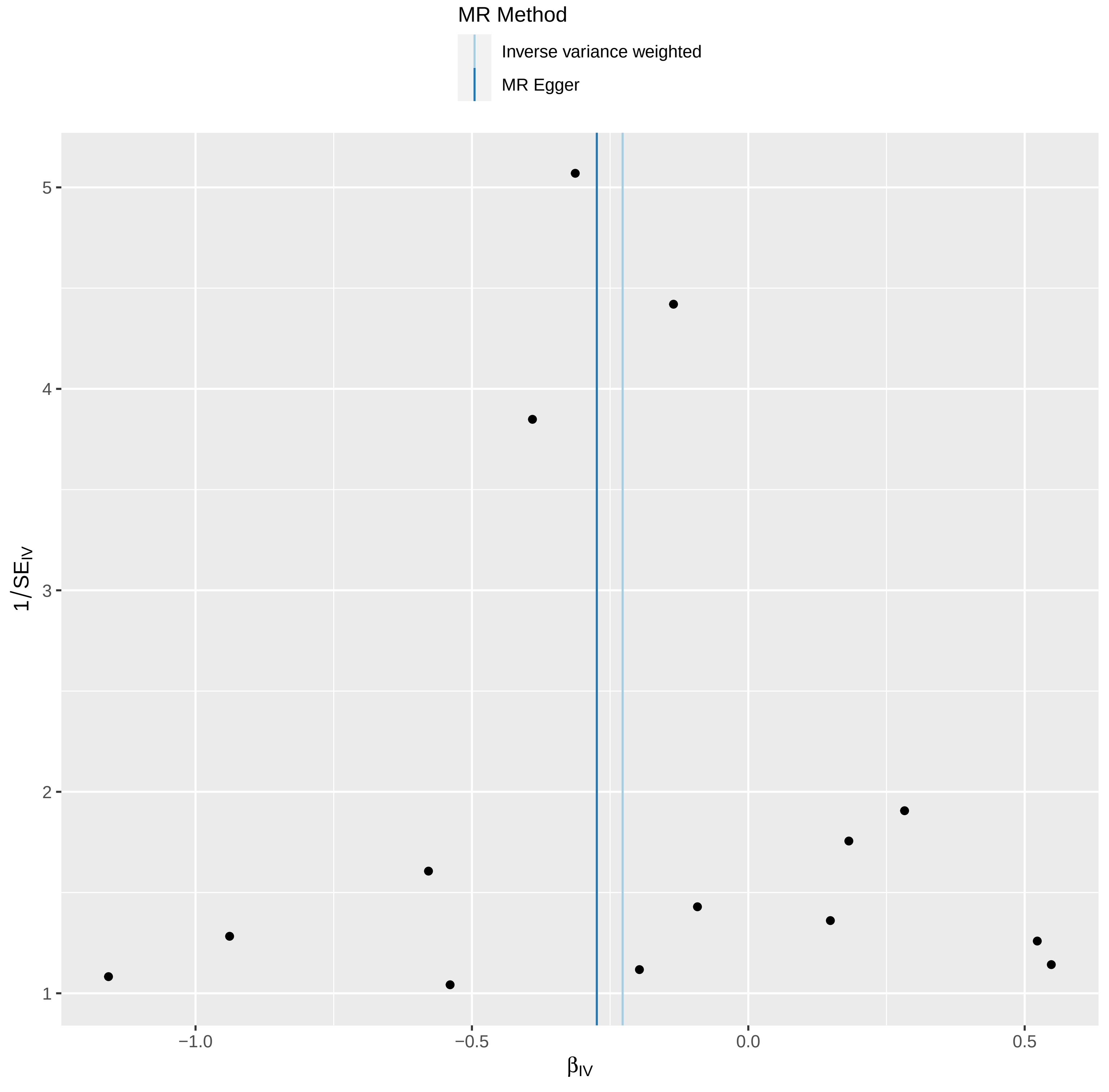
MR Method
Inverse variance weighted
MR Egger
5
4
3
2
1
1/SE_IV
−1.0
−0.5
0.0
0.5
β_IV

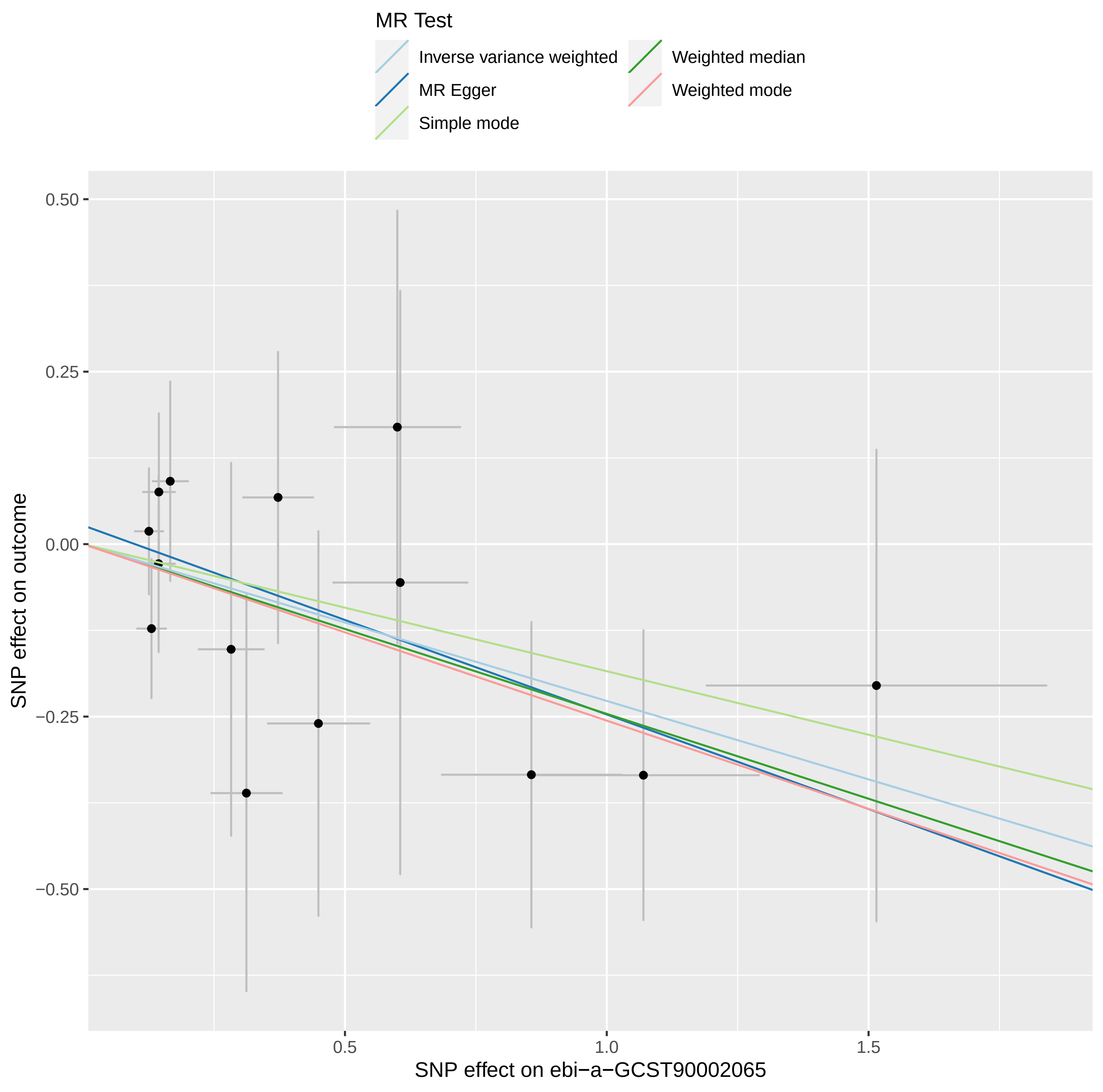
MR Test
Inverse variance weighted
MR Egger
Simple mode
Weighted median
Weighted mode
0.50
0.25
0.00
−0.25
−0.50
SNP effect on outcome
0.5
1.0
1.5
SNP effect on ebi−a−GCST90002065

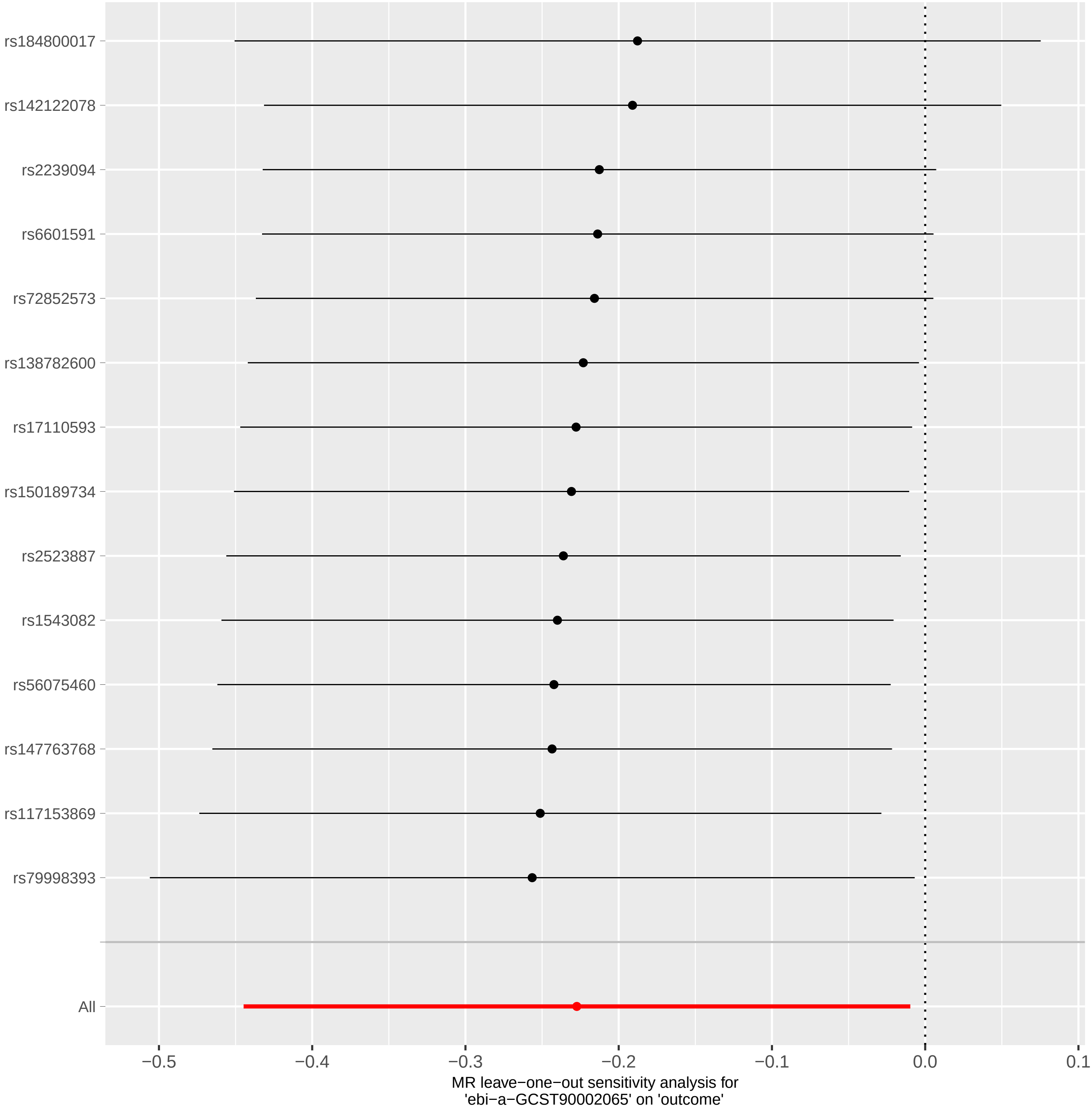
rs184800017
rs142122078
rs2239094
rs6601591
rs72852573
rs138782600
rs17110593
rs150189734
rs2523887
rs1543082
rs56075460
rs147763768
rs117153869
rs79998393
All
−0.5
−0.4
−0.3
−0.2
−0.1
0.0
0.1
MR leave−one−out sensitivity analysis for
'ebi−a−GCST90002065' on 'outcome'

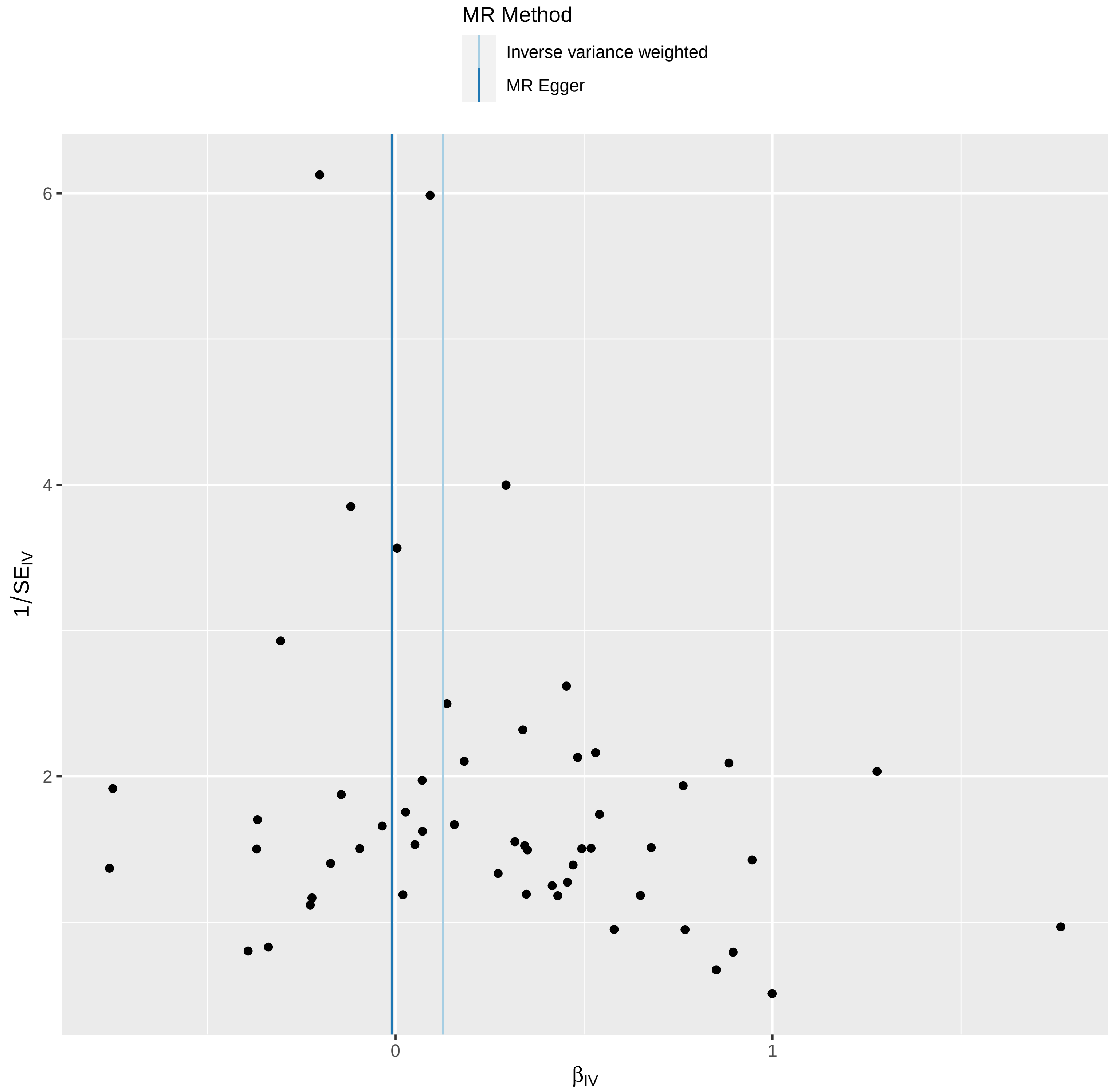
MR Method
Inverse variance weighted
MR Egger
6
4
2
1/SE_IV
0
1
β_IV

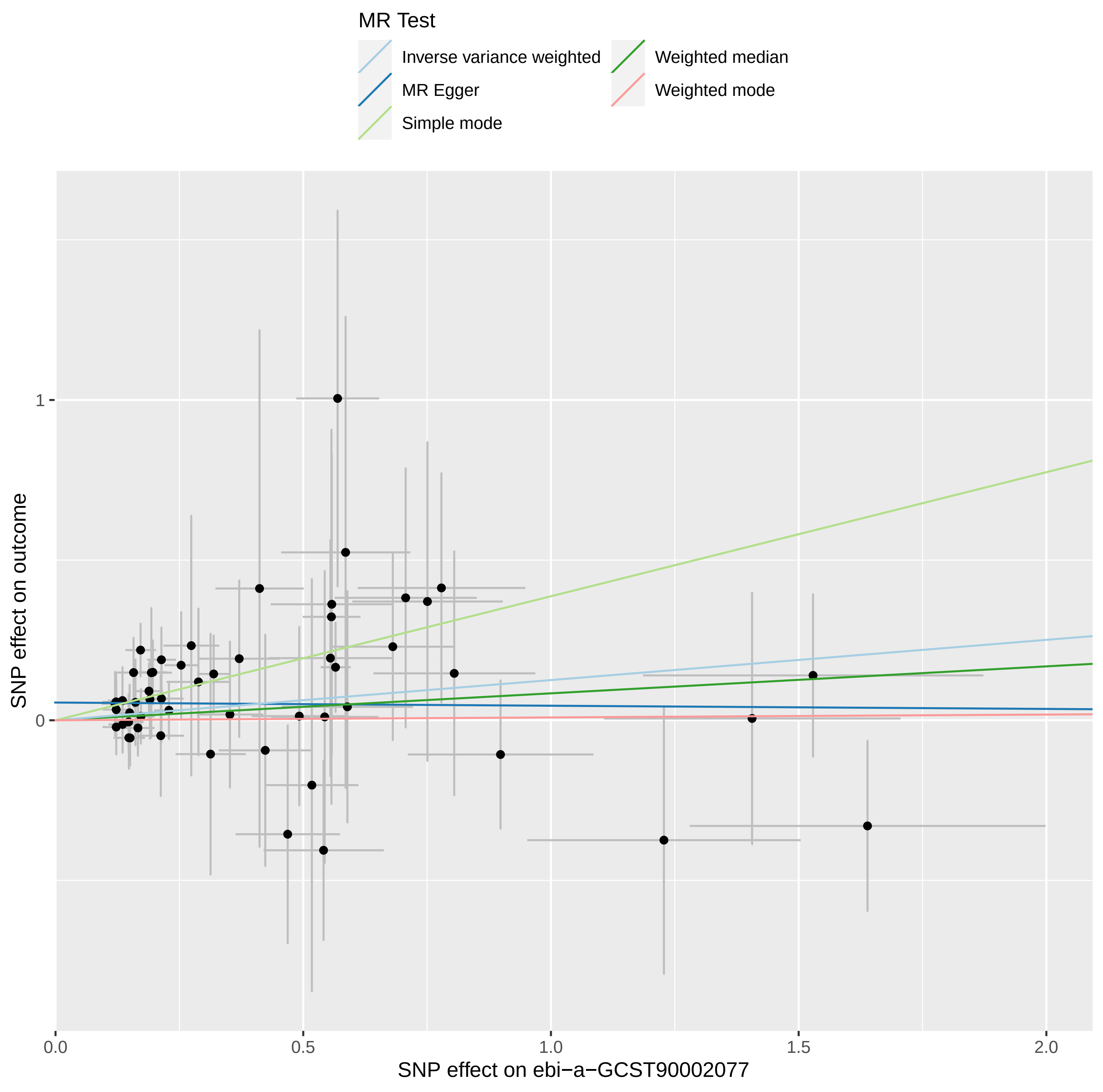
MR Test
Inverse variance weighted
MR Egger
Simple mode
Weighted median
Weighted mode
1
0
0.0
0.5
1.0
1.5
2.0
SNP effect on outcome
SNP effect on ebi−a−GCST90002077

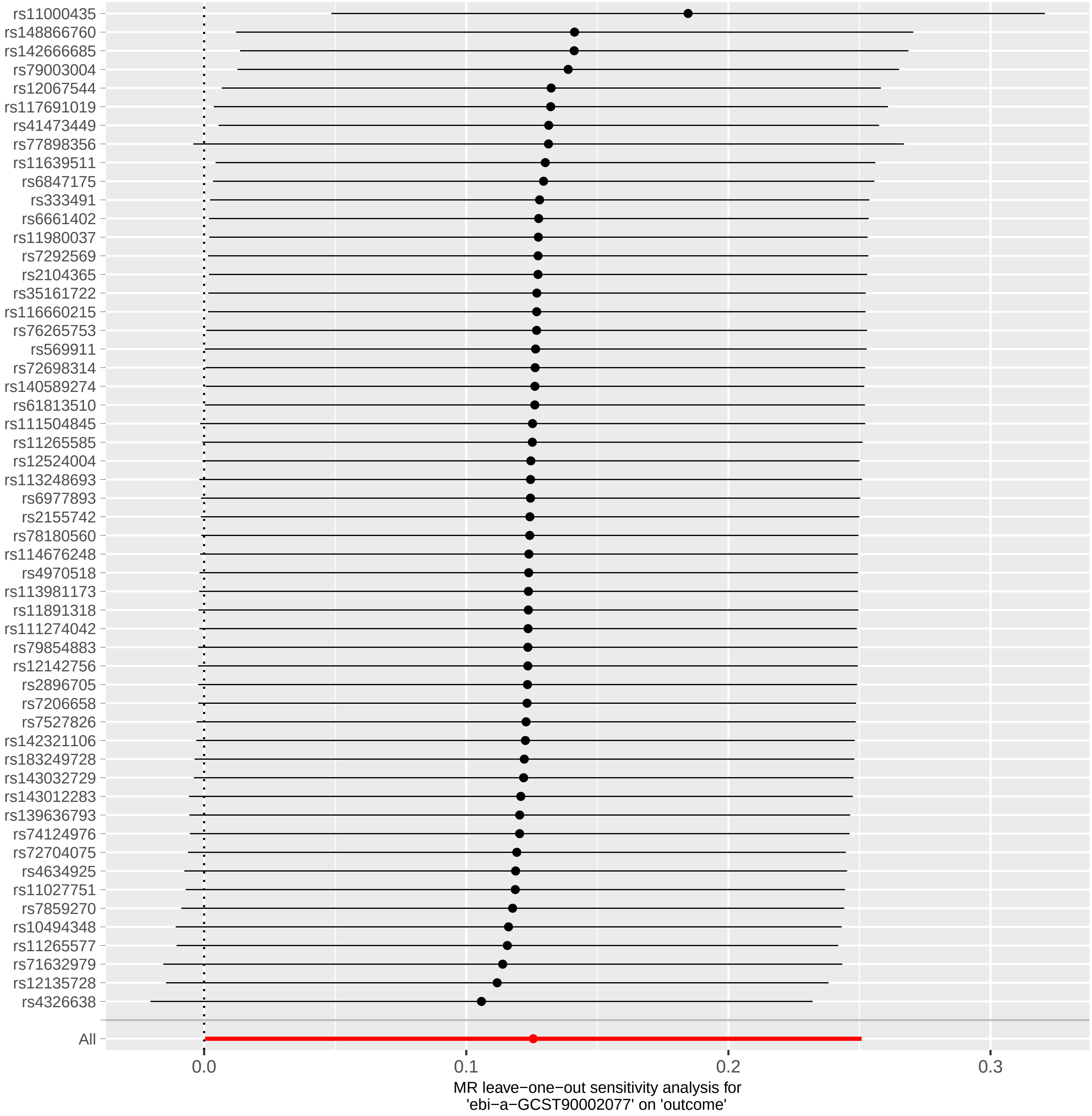

rs11000435
rs148866760
rs142666685
rs79003004
rs12067544
rs117691019
rs41473449
rs77898356
rs11639511
rs6847175
rs333491
rs6661402
rs11980037
rs7292569
rs2104365
rs35161722
rs116660215
rs76265753
rs569911
rs72698314
rs140589274
rs61813510
rs111504845
rs11265585
rs12524004
rs113248693
rs6977893
rs2155742
rs78180560
rs114676248
rs4970518
rs113981173
rs11891318
rs111274042
rs79854883
rs12142756
rs2896705
rs7206658
rs7527826
rs142321106
rs183249728
rs143032729
rs143012283
rs139636793
rs74124976
rs72704075
rs4634925
rs11027751
rs7859270
rs10494348
rs11265577
rs71632979
rs12135728
rs4326638
All
0.0
0.1
0.2
0.3
MR leave−one−out sensitivity analysis for
'ebi−a−GCST90002077' on 'outcome'

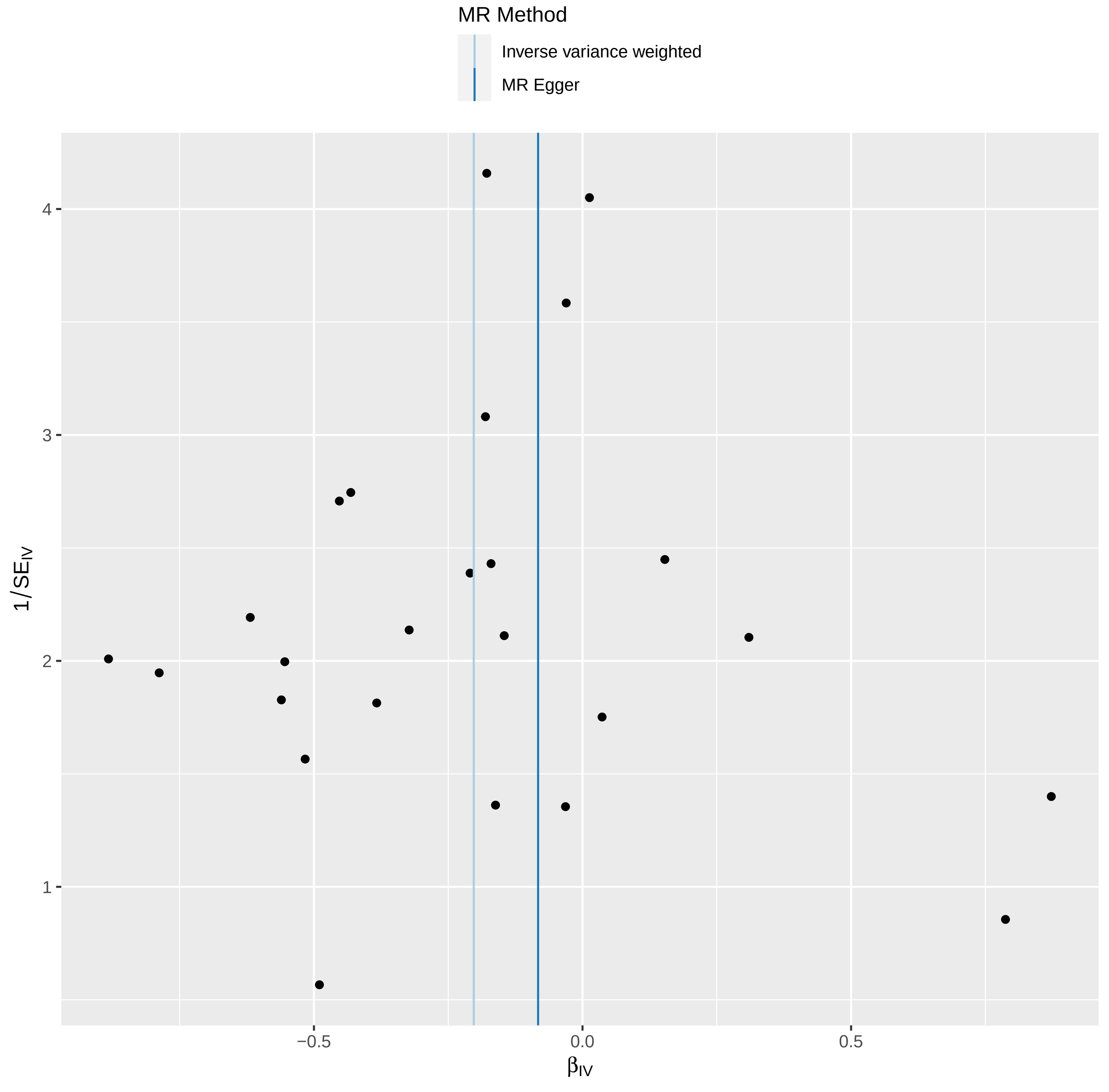
MR Method
Inverse variance weighted
MR Egger
4
3
2
1
1/SE$_{IV}$
−0.5
0.0
0.5
β$_{IV}$

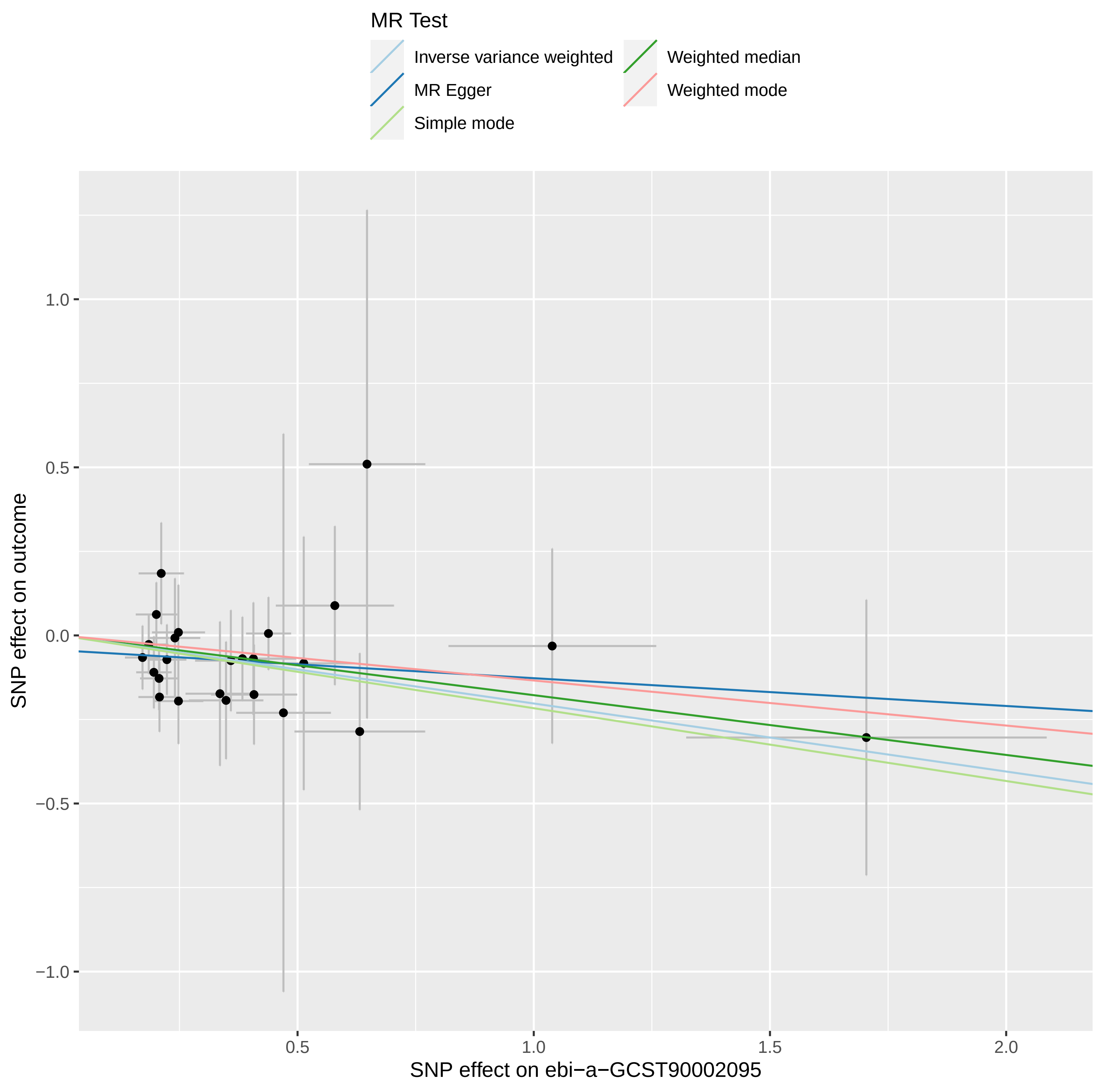
MR Test
Inverse variance weighted
MR Egger
Simple mode
Weighted median
Weighted mode
1.0
0.5
0.0
−0.5
−1.0
SNP effect on outcome
0.5
1.0
1.5
2.0
SNP effect on ebi−a−GCST90002095

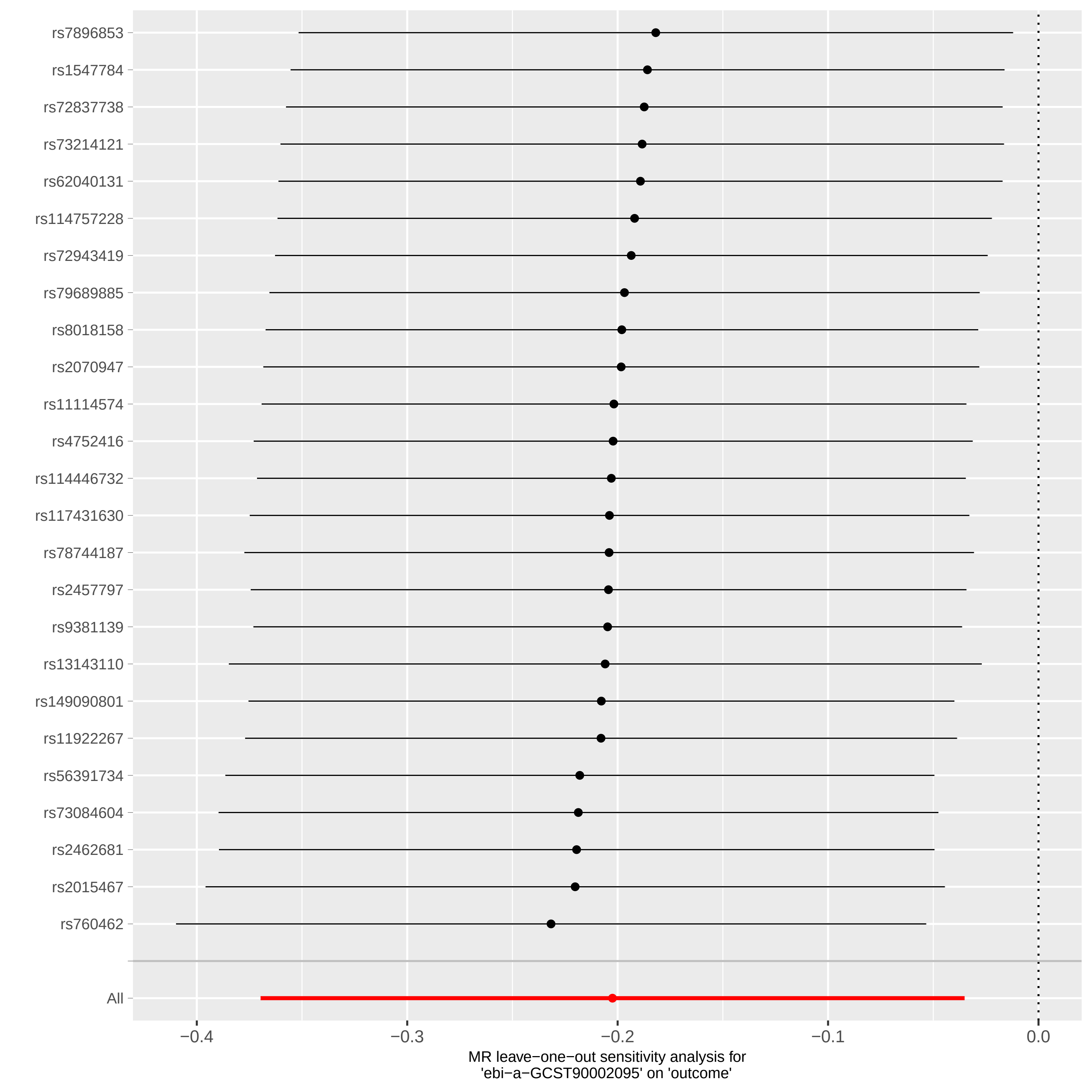
rs7896853
rs1547784
rs72837738
rs73214121
rs62040131
rs114757228
rs72943419
rs79689885
rs8018158
rs2070947
rs11114574
rs4752416
rs114446732
rs117431630
rs78744187
rs2457797
rs9381139
rs13143110
rs149090801
rs11922267
rs56391734
rs73084604
rs2462681
rs2015467
rs760462
All
−0.4
−0.3
−0.2
−0.1
0.0
MR leave−one−out sensitivity analysis for
'ebi−a−GCST90002095' on 'outcome'

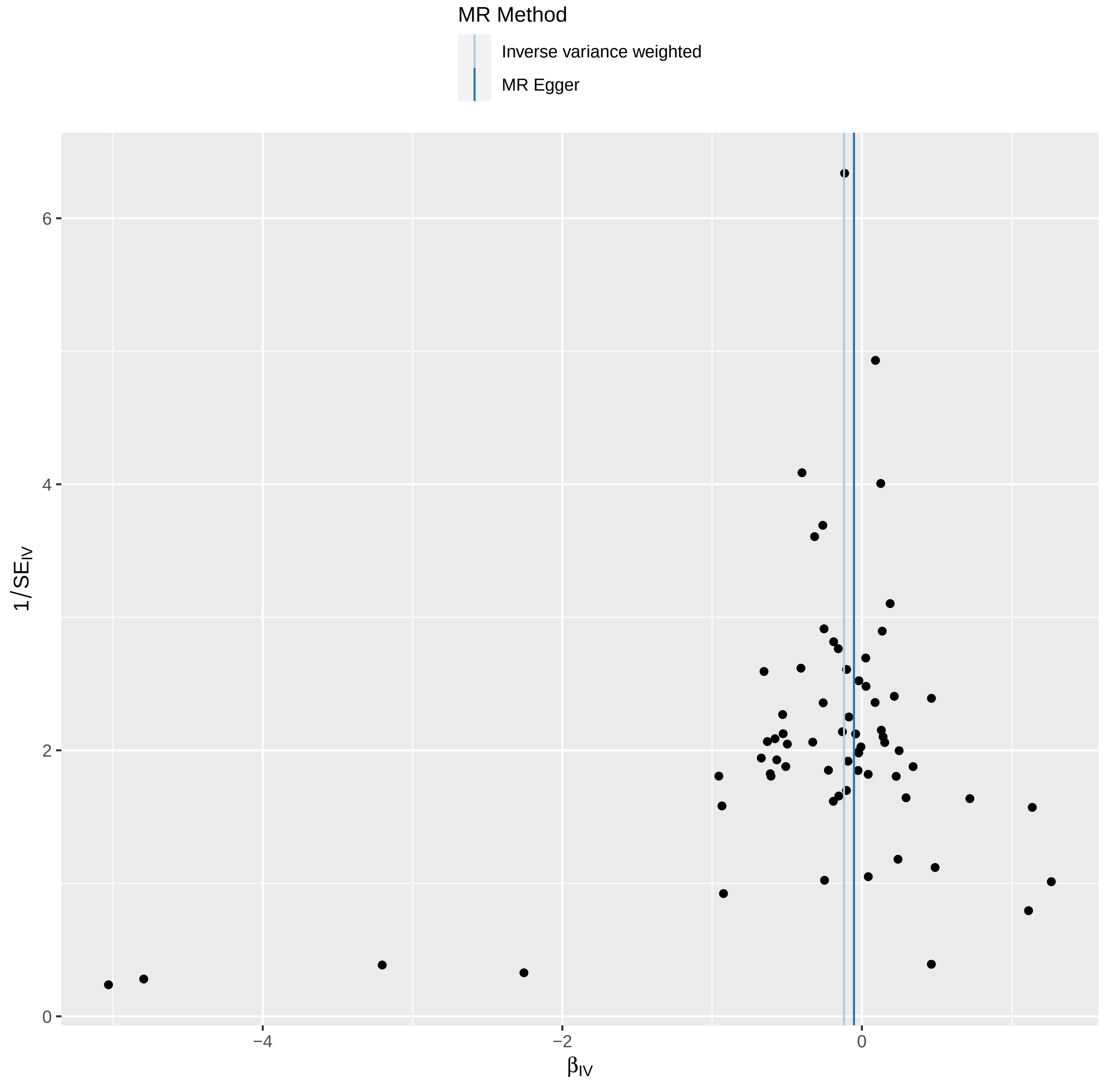
MR Method
Inverse variance weighted
MR Egger
1/SE_IV
β_IV
6
4
2
0
−4
−2
0

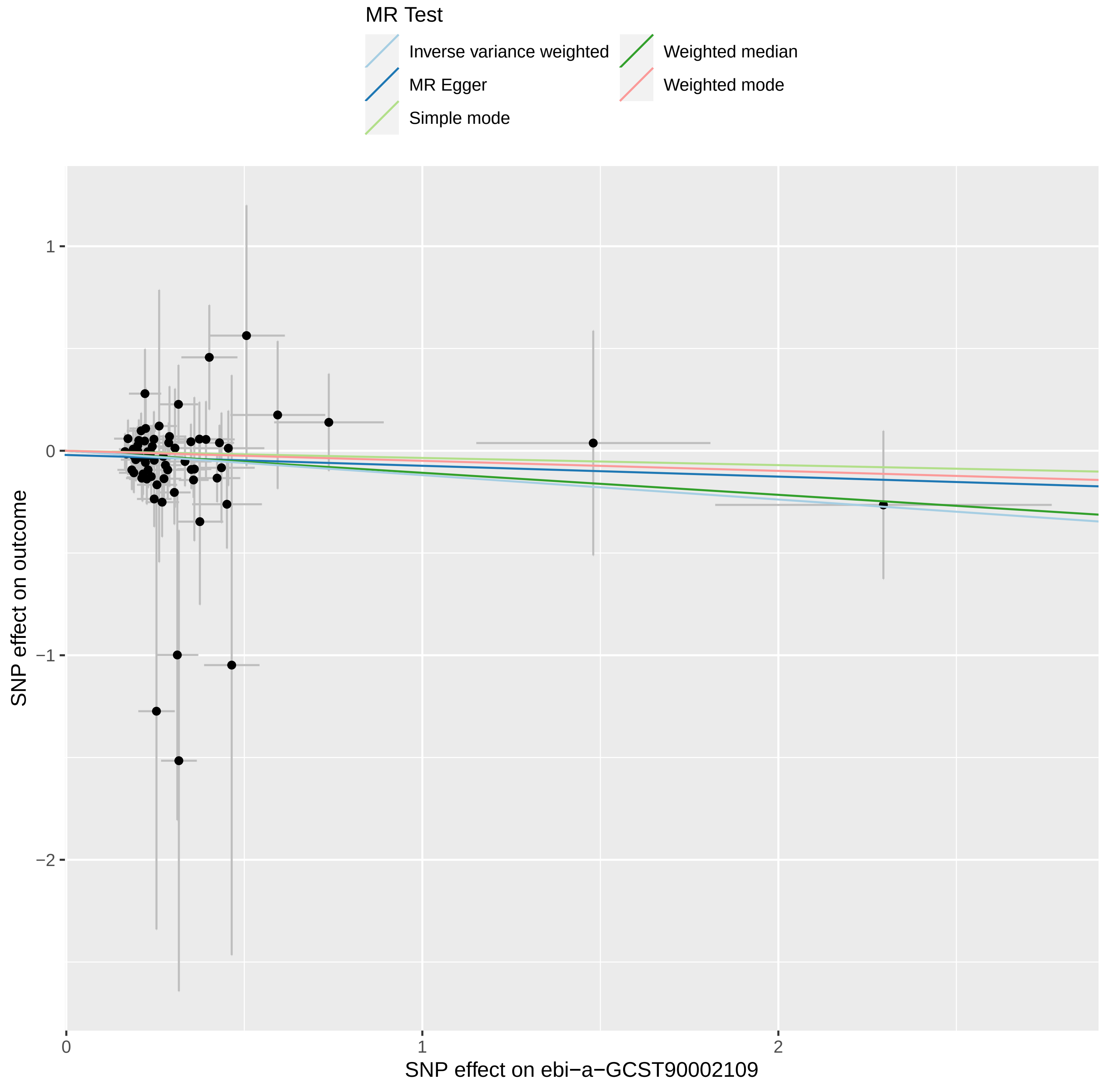

MR Test
Inverse variance weighted
MR Egger
Simple mode
Weighted median
Weighted mode
1
0
−1
−2
0
1
2
SNP effect on outcome
SNP effect on ebi−a−GCST90002109

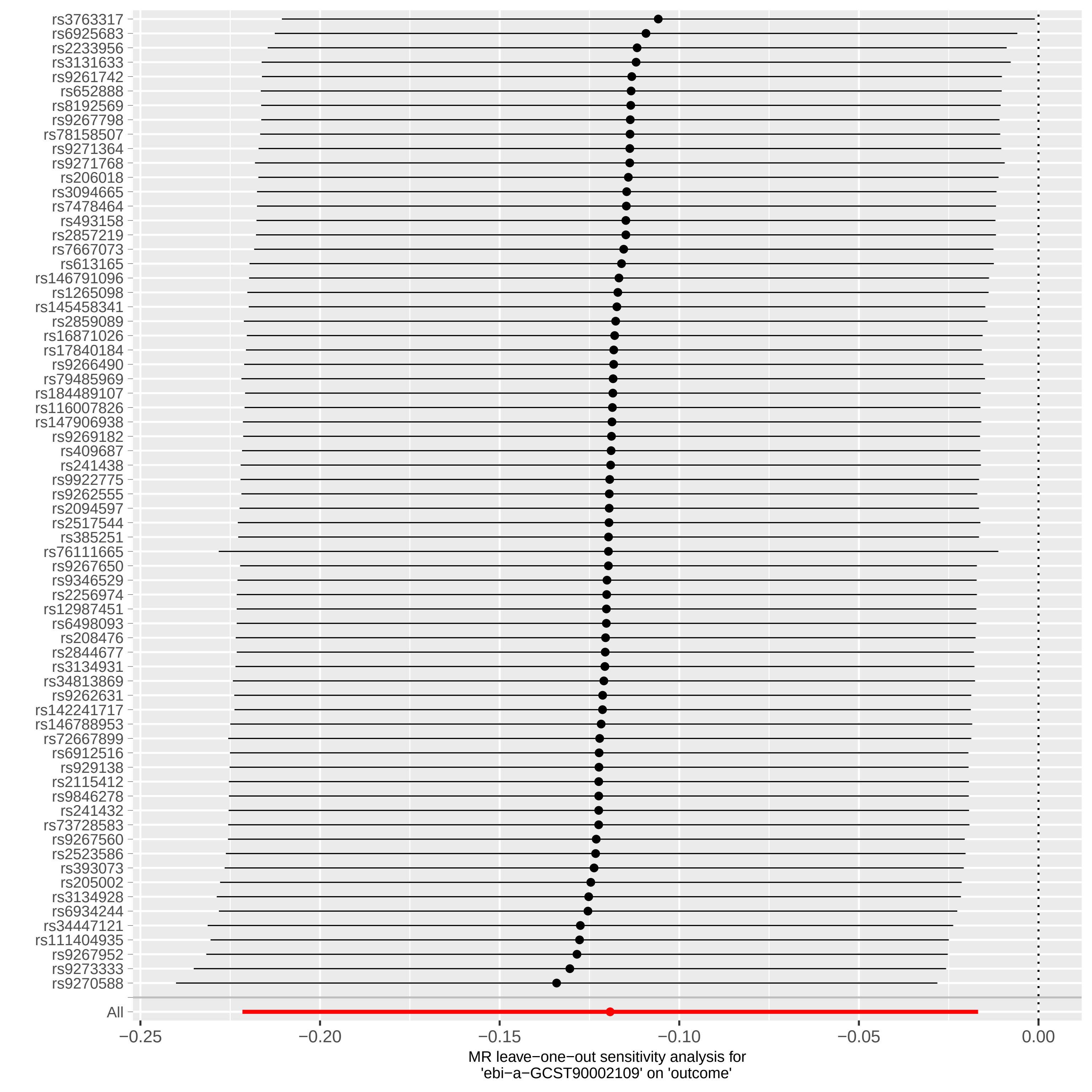
rs3763317
rs6925683
rs2233956
rs3131633
rs9261742
rs652888
rs8192569
rs9267798
rs78158507
rs9271364
rs9271768
rs206018
rs3094665
rs7478464
rs493158
rs2857219
rs7667073
rs613165
rs146791096
rs1265098
rs145458341
rs2859089
rs16871026
rs17840184
rs9266490
rs79485969
rs184489107
rs116007826
rs147906938
rs9269182
rs409687
rs241438
rs9922775
rs9262555
rs2094597
rs2517544
rs385251
rs76111665
rs9267650
rs9346529
rs2256974
rs12987451
rs6498093
rs208476
rs2844677
rs3134931
rs34813869
rs9262631
rs142241717
rs146788953
rs72667899
rs6912516
rs929138
rs2115412
rs9846278
rs241432
rs73728583
rs9267560
rs2523586
rs393073
rs205002
rs3134928
rs6934244
rs34447121
rs111404935
rs9267952
rs9273333
rs9270588
All
−0.25
−0.20
−0.15
−0.10
−0.05
0.00
MR leave−one−out sensitivity analysis for
'ebi−a−GCST90002109' on 'outcome'
